# *atpE* gene as a new useful specific molecular target to quantify *Mycobacterium* in environmental samples

**DOI:** 10.1186/1471-2180-13-277

**Published:** 2013-12-03

**Authors:** Nicolas Radomski, Adélaïde Roguet, Françoise S Lucas, Frédéric J Veyrier, Emmanuelle Cambau, Héberte Accrombessi, Régis Moilleron, Marcel A Behr, Laurent Moulin

**Affiliations:** 1 Laboratoire Eau Environnement Systèmes Urbains (Leesu) UMR MA 102-AgroParisTech, Université Paris-Est, 6-8 avenue Blaise Pascal Cité, Descartes, FR 77455, Champs sur Marne, France; 2 Research Institute of the McGill University Health Centre, 1650 Cedar Avenue, Montreal H3G 1A4, QC, Canada; 3 Département Infection et Epidémiologie, Infections Bactériennes Invasives, Institut Pasteur, 28 Rue du Dr. Roux, Paris, F 75015, France; 4 Laboratoire associé du Centre national de référence des mycobactéries et de la résistance aux antituberculeux, AP-HP, Groupe Hospitalier Saint Louis-Lariboisière, Université Paris Diderot EA3964, FR 75475, Paris, France; 5 Eau de Paris, Direction Recherche et Développement Qualité de l'Eau (DRDQE), 33 avenue Jean Jaurès, FR 94200, Ivry-sur-Seine, France

**Keywords:** Mycobacteria, *atpE* gene, Environmental samples

## Abstract

**Background:**

The environment is the likely source of many pathogenic mycobacterial species but detection of mycobacteria by bacteriological tools is generally difficult and time-consuming. Consequently, several molecular targets based on the sequences of housekeeping genes, non-functional RNA and structural ribosomal RNAs have been proposed for the detection and identification of mycobacteria in clinical or environmental samples. While certain of these targets were proposed as specific for this genus, most are prone to false positive results in complex environmental samples that include related, but distinct, bacterial genera. Nowadays the increased number of sequenced genomes and the availability of software for genomic comparison provide tools to develop novel, mycobacteria-specific targets, and the associated molecular probes and primers. Consequently, we conducted an *in silico* search for proteins exclusive to *Mycobacterium* spp. genomes in order to design sensitive and specific molecular targets.

**Results:**

Among the 3989 predicted proteins from *M. tuberculosis* H37Rv, only 11 proteins showed 80% to 100% of similarity with *Mycobacterium* spp. genomes, and less than 50% of similarity with genomes of closely related *Corynebacterium*, *Nocardia* and *Rhodococcus* genera. Based on DNA sequence alignments, we designed primer pairs and a probe that specifically detect the *atpE* gene of mycobacteria, as verified by quantitative real-time PCR on a collection of mycobacteria and non-mycobacterial species. The real-time PCR method we developed was successfully used to detect mycobacteria in tap water and lake samples.

**Conclusions:**

The results indicate that this real-time PCR method targeting the *atpE* gene can serve for highly specific detection and precise quantification of *Mycobacterium* spp. in environmental samples.

## Background

*Mycobacterium* genus is constituted of several pathogenic species, including the *M. tuberculosis* complex (MTC) responsible for tuberculosis (i.e. *M. tuberculosis*, *M. africanum*, *M. bovis*, *M. canettii*, *M. caprae*, *M. microti* and *M. pinnipedii*), *M. leprae* responsible for leprosy, and non-tuberculous mycobacteria (NTM), which are environmental potentially pathogenic species causing mycobacteriosis [1]. Detection of mycobacteria by bacteriological tools is generally time-consuming and difficult because most pathogenic mycobacteria are slow growing, such that other microorganisms overgrow NTM colonies [2]. Identification of mycobacteria based on metabolic criteria is also problematic as current methods do not allow for proper identification of mycobacterial species and sub-species. Consequently, molecular tools have been developed using *rrs*, *gyrA*, *gyrB*, *hsp65*, *recA*, *rpoB*, *sodA* genes and 16S-23S internal transcribed spacer (ITS) genes, to detect and/or identify mycobacteria species by sequence analysis [3,4]. In order to detect *Mycobacterium* genus in clinical and environmental samples, several studies have proposed targeting different loci of the 16S rRNA gene [5-17], or other housekeeping genes such as *gyrB *[18], *rpoB*[19], and *hsp65*[20]. Nevertheless, in a recent study comparing several primers commonly used for mycobacterial detection or identification, we demonstrated that most of these primers present either a high specificity (i.e. the proportion of true negatives that are correctly identified by the test) but a low sensitivity (i.e. the proportion of true positives that are correctly identified by the test), or conversely a high sensitivity but a low specificity [17]. Indeed, some of these methods fail to detect several mycobacterial species by PCR, while other primers lead to detection of closely related genera [17] which also belong to the *Corynebacterium*, *Nocardia*, *Rhodococcus*, *Mycobacterium* (CNM) group [21] and which are commonly present in water and soil samples. Consequently, new strategies must be used in order to design *Mycobacterium* genus targets with high levels of specificity and sensitivity that will be useful for studying mycobacteria in their habitat.

As new mycobacterial sequences are added into genetic databases, our knowledge of mycobacterial genomes is increasing and this may help to design new primers and probes that will be both specific and sensitive. Since the whole sequencing of the first mycobacterial genome in 1998 [22] by Sanger sequencing method (*M. tuberculosis* H37Rv), the number of mycobacterial sequences has considerably increased due to advances in sequencing capacity and the appearance of high throughput sequencing techniques [23]. Today, GenBank database provides access to whole genomes of seven other strains of the MTC (*M. tuberculosis* and *M. bovis* species), two strains of *M. leprae*, and eleven species and subspecies of pathogenic (P) and non-pathogenic (NP) NTM: *M. abscessus* (P), *M. avium* (P), *M. avium* subsp. *paratuberculosis* (P), *M. gilvum* (NP), *M. marinum* (P), *M. smegmatis* (NP), *Mycobacterium* sp. JLS (NP), *Mycobacterium* sp. KMS (NP), *Mycobacterium* sp. MCS (NP), *M. ulcerans* (P), *M. vanbaalenii* (NP), [24-26]. Moreover, three whole genomes of other NTM species were sequenced and are currently assembled (*M. intracellulare*, *M. kansasii*, *M. parascrofulaceum*). This increasing number of completely sequenced mycobacterial genomes led to the development of the MycoHit software, which permits gene- and protein-level comparisons across mycobacteria species, [27]. This software was originally developed to detect horizontal gene transfers and mutations among whole mycobacterial genomes [27]. However, MycoHit should also be useful for developing new primers and probes for mycobacteria detection and quantification in environmental and clinical samples.

In this paper, we used this tool for screening sensitive and specific targets of *Mycobacterium* spp.. We compared *in silico* proteins of whole mycobacterial genomes with those of non-mycobacterial genomes using the MycoHit software, in order to find conserved sequences among mycobacteria that will not be shared with non-mycobacterial species. Based on the screening results a primer pair and a probe targeting the *atpE* gene were designed and tested by real-time PCR. This novel target proved to be totally specific and sensitive. It also offers the advantage of targeting a gene present as a single copy in the genome. Thus this new real-time PCR method appears promising for water quality survey, and should be useful for studying the ecology of mycobacteria in aquatic, terrestrial and urban environments.

## Results

### Specificity of genes commonly used for mycobacterial detection/identification

**Figure 1:**
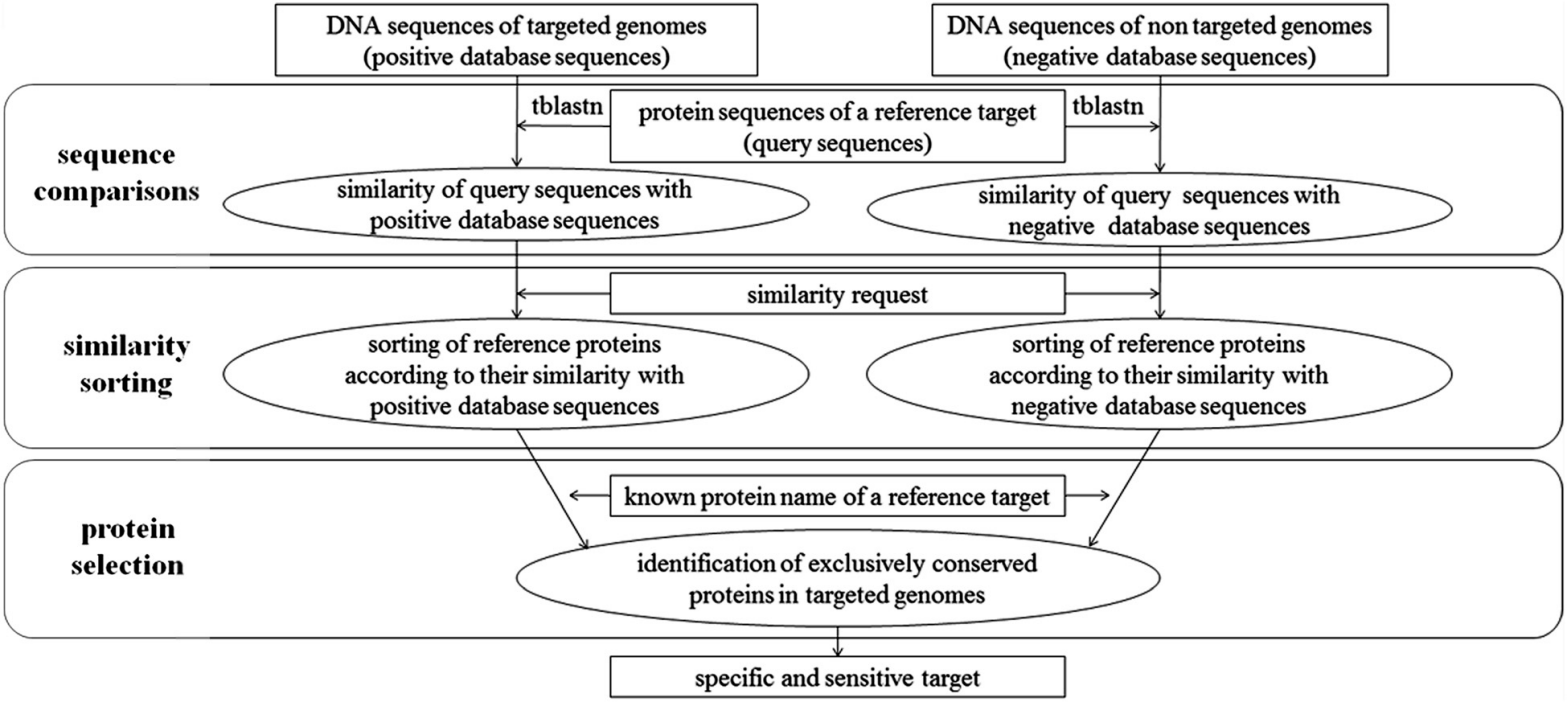
**Strategy used to identify sensitive and specific targets in *Mycobacterium *spp. whole genomes based on MycoHit software.** DNA sequences of targeted mycobacterial genomes include *M. tuberculosis* H37Ra (CP000611.1), *M. tuberculosis* CDC 1551 (AE000516.2), *M. tuberculosis* KZN 1435 (CP001658.1), *M. bovis* AF2122/97 (BX248333.1), *M. ulcerans* Agy99 (CP000325.1), *M. marinum* M (CP000854.1), *M. avium* 104 (CP000479.1), *M. paratuberculosis* K10 (AE016958.1), *M. smegmatis* MC2 155 (CP000480.1), *M. abscessus* ATCC 19977 (CU458896.1), *M. gilvum* PYG-GCK (CP000656.1), *M. vanbaalenii* PYR-1 (CP000511.1), *Mycobacterium* sp. JLS (CP000580.1), *Mycobacterium* sp. KMS (CP000518.1), *Mycobacterium* sp. MCS (CP000384.1), and DNA sequences of non-targeted genomes include *Corynebacterium aurimucosum* ATCC 700975 (CP001601.1), *C. diphteriae* NCTC 13129 (BX248353.1), *C. efficiens* YS-314 (BA000035.2), *C. glutamicum* ATCC 13032 (BX927147.1), *C. jeikeium* K411 (NC_007164), *C. kroppenstedtii* DSM 44385 (CP001620.1), *C. urealyticum* DSM 7109 (AM942444.1), *Nocardia farcinica* IFM 10152 (AP006618.1), *Nocardioides* sp. JS614 (CP000509.1), *Rhodococcus erythropolis* PR4 (AP008957.1), *R. jostii* RHA1 (CP000431.1) and *R. opacus* B4 (AP011115.1).

Excluding *rrs* gene and ITS (non-functional RNA elements and structural ribosomal RNAs), and according to our strategy of genome comparison (Figure 1) most of the genes commonly used for mycobacterial species identification (*gyrA*, *gyrB*, *hsp65*, *recA*, *rpoB*, *sodA, groEL1, groEL2*) code for proteins which present similar conformations in non-mycobacterial studied genomes (Additional file 1). Indeed, protein similarity levels of these genes, in comparison with *M. tuberculosis* H37Rv genome, were higher than 80% for the other 15 mycobacterial genomes studied (96 ± 2% for *gyrA*, 94 ± 5% for *gyrB*, 79 ± 5% for *groEL1*, 93 ± 4% for *groEL2* which is an alternative gene name for *hsp65*, 99 ± 1% for *recA*, 96 ± 2% for *rpoB*, 81 ± 33% for *sodA*), and also for the 12 non-mycobacterial genomes studied (86 ± 5% for *gyrA*, 85 ± 5% for *gyrB*, 89 ± 3% for *groEL1*, 96 ± 2% for *groEL2*, 94 ± 3% for *recA*, 88 ± 4% for *rpoB*, 69 ± 22% for *sodA*).

### Selection of exclusively conserved proteins in *Mycobacterium* spp. genomes

**Figure 2:**
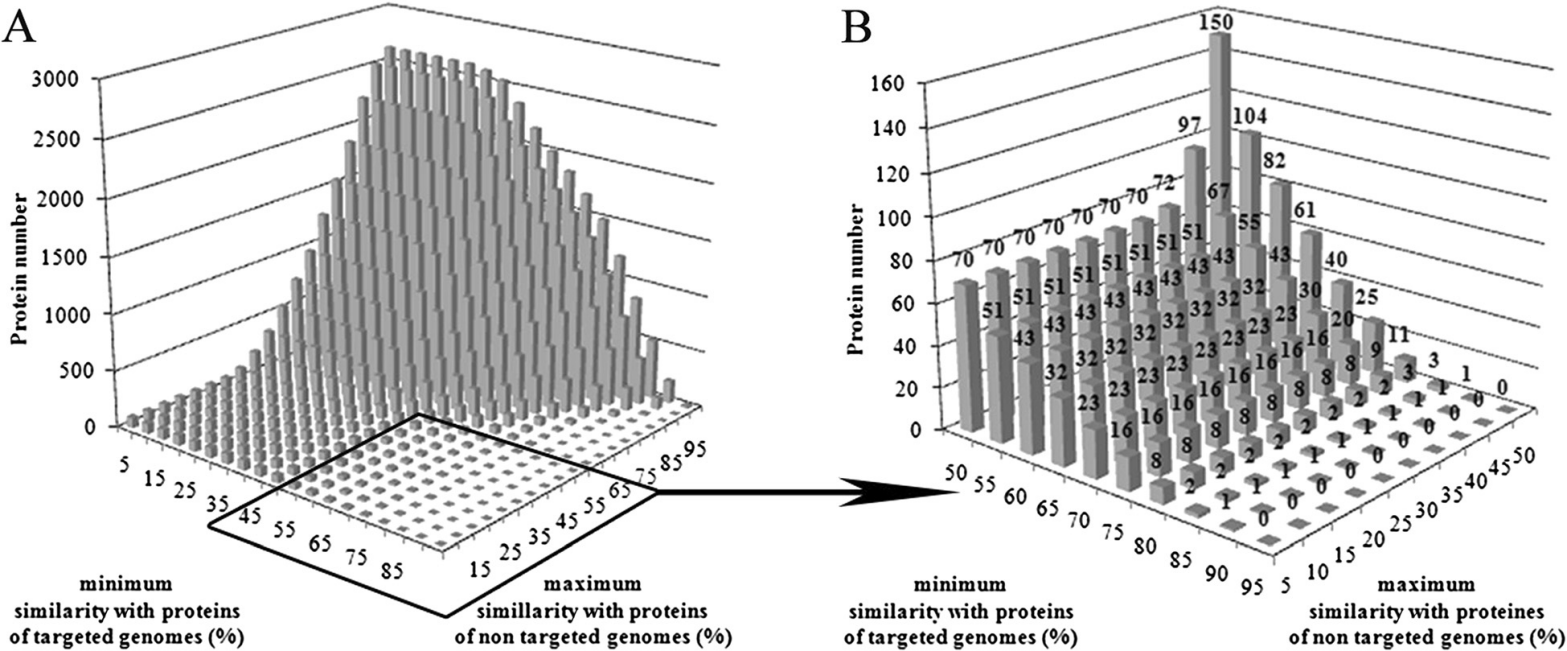
**Total (A) and partial representation (B) of the protein number (vertical axe, number in the top of the bars) of *Mycobacterium tuberculosis *H37Rv genome, according to their similarities with proteins of targeted mycobacterial genomes and proteins of non-targeted genomes (horizontal axes).** Targeted mycobacterial genomes include *M. tuberculosis* H37Ra (CP000611.1), *M. tuberculosis* CDC 1551 (AE000516.2), *M. tuberculosis* KZN 1435 (CP001658.1), *M. bovis* AF2122/97 (BX248333.1), *M. ulcerans* Agy99 (CP000325.1), *M. marinum* M (CP000854.1), *M. avium* 104 (CP000479.1), *M. paratuberculosis* K10 (AE016958.1), *M. smegmatis* MC2 155 (CP000480.1), *M. abscessus* ATCC 19977 (CU458896.1), *M. gilvum* PYG-GCK (CP000656.1), *M. vanbaalenii* PYR-1 (CP000511.1), *Mycobacterium* sp. JLS (CP000580.1), *Mycobacterium* sp. KMS (CP000518.1), *Mycobacterium* sp. MCS (CP000384.1), and non-targeted genomes include *Corynebacterium aurimucosum* ATCC 700975 (CP001601.1), *C. diphteriae* NCTC 13129 (BX248353.1), *C. efficiens* YS-314 (BA000035.2), *C. glutamicum* ATCC 13032 (BX927147.1), *C. jeikeium* K411 (NC_007164), *C. kroppenstedtii* DSM 44385 (CP001620.1), *C. urealyticum* DSM 7109 (AM942444.1), *Nocardia farcinica* IFM 10152 (AP006618.1), *Nocardioides* sp. JS614 (CP000509.1), *Rhodococcus erythropolis* PR4 (AP008957.1), *R. jostii* RHA1 (CP000431.1) and *R. opacus* B4 (AP011115.1).

Among the 3989 predicted proteins of *M. tuberculosis* H37Rv genome (Figure 2A and Additional file 1), about 54.6% (i.e. 2177 proteins) presented protein similarities above 50% with the other studied mycobacterial genomes (n = 15), and only 6.8% of these hypothetical conserved mycobacterial proteins (150 proteins: 150 number in the top of a bar in Figure 2B) displayed similarities less than 50% with the studied non-mycobacterial genomes (n = 12). Consequently, almost half of the *M. tuberculosis* H37Rv predicted proteins are potentially present in the 12 studied genomes of CNM group members. We chose to decrease the number of candidate proteins by restricting the panel of studied proteins to those exclusively conserved in the mycobacterial genomes, focusing on *M. tuberculosis* H37Rv proteins with similarity levels between 80% and 100% in comparison with other mycobacterial genomes (n = 15), and less than 50% similarity levels in comparison with genomes (n = 12) of the other CNM group genera. As a result, among the 3989 predicted proteins of *M. tuberculosis* H37Rv genome (Figure 2A), we selected 11 proteins (11 number in the top of a bar in Figure 2B). Among the 3989 predicted proteins of *M. tuberculosis* H37Rv proteins (Additional file 1), the selected candidate proteins (Table 1), were the subunits C (locus Rv1305) and A (locus Rv1304) of the ATP synthase, the cyclopropane mycolic acid synthase (CMAS) coded by the *cmaA1* gene in *M. tuberculosis* H37Rv (locus Rv3392c), hypothetical PE or PPE family proteins (loci Rv0285 and Rv3022c), proteins coded by *esxG*, *esxH* and *esxR* genes in *M. tuberculosis* H37Rv (loci Rv0287, Rv0288, Rv3019c, respectively), and proteins such as a lipoprotein coding by *lppM* gene (locus Rv2172c), an oxidoreductase (locus Rv0197), and a small secreted protein (locus Rv0236A).

**Table 1:** **Similarity (%) of the most conserved mycobacterial proteins in *Mycobacterium *** **spp., *Corynebacterium *** **spp., *Nocardia *** **spp. and *Rhodococcus *** **spp. genomes, in comparison with *M. tuberculosis *** **H37Rv genome**

**Protein locus (H37Rv genome)**	**Rv1305**	**Rv0236A**	**Rv0197**	**Rv2172c**	**Rv0287**	**Rv0288**	**Rv3019c**	**Rv0285**	**Rv3022c**	**Rv1304**	**Rv3392c**
**protein length (aa)**	**81**	**57**	**762**	**301**	**97**	**96**	**96**	**102**	**81**	**250**	**287**
**gene name**	***atpE***	***-***	***-***	***lppM***	***esxG***	***esxH***	***esxR***	***PE5***	***PPE48***	***atpB***	***cmaA1***
*M. tuberculosis* H37Ra	100	100	99	100	100	100	100	100	100	100	100
*M. tuberculosis* CDC1551	100	100	99	100	100	100	100	100	100	100	99
*M. tuberculosis* KZN 1435	100	100	99	100	100	100	100	100	100	100	100
*M. bovis* AF2122/97	100	100	99	100	100	100	100	100	98	100	100
*M. ulcerans* Agy99	100	96	86	90	96	92	93	93	83	96	87
*M. marinum* M	100	98	90	91	96	89	94	93	82	97	88
*M. avium*104	96	96	91	91	91	89	91	92	83	93	82
*M. paratuberculosis* K10	96	96	91	91	91	89	91	92	85	92	82
*M. smegmatis* MC2 155	93	91	85	83	87	85	85	87	82	84	86
*M. abscessus* ATCC 19977	98	85	85	82	81	81	80	82	81	85	82
*M. gilvum* PYR-GCK	100	91	85	86	88	88	85	85	80	83	81
*M. vanbaalenii* PYR-1	93	91	85	87	89	85	83	82	83	84	81
*Mycobacterium* sp*.* JLS	100	91	85	86	87	86	86	82	82	89	92
*Mycobacterium* sp*.* KMS	100	91	86	86	88	86	86	82	82	89	91
*Mycobacterium* sp*.* MCS	100	91	86	86	88	86	86	82	82	89	91
*C. aurimucosum* ATCC 700975	0	0	0	0	0	0	0	0	0	0	46
*C. diphteriae* NCTC 13129	0	0	0	0	0	0	0	0	0	43	0
*C. efficiens* YS-314	0	0	42	0	0	0	0	0	0	0	0
*C. glutamicum* ATCC 13032	0	0	42	0	0	0	0	0	0	0	47
*C. jeikeium* K411	0	0	0	0	0	0	0	0	0	45	0
*C. kroppenstedtii* DSM 44385	0	0	0	0	0	0	0	0	0	41	47
*C. urealyticum* DSM 7109	0	0	38	0	0	0	0	0	0	44	41
*Nocardioides* sp. JS614	0	0	40	0	0	0	0	0	0	46	46
*N. farcinica* IFM 10152	0	0	42	0	0	0	0	0	0	0	44
*R. erythropolis* PR4	0	0	42	0	0	0	0	0	0	42	48
*R. jostii* RHA1	0	0	44	0	0	0	0	0	0	41	49
*R. opacus* B4	0	0	44	0	0	0	0	0	0	41	50

Protein similarities were sorted (Figure 2) according to the strategy of genome comparison (Figure 1). Only proteins presenting more than 80% of similarity with *Mycobacterium* spp. genomes, and less than 50% of similarity with non-mycobacterial genomes, are shown.

### Mycobacterial molecular target design

Among the 11 selected mycobacterial proteins, protein alignments revealed that the ATP synthase subunit C (locus Rv1305), the oxidoreductase (locus Rv0197), and the small secreted protein (locus Rv0236A), are the less polymorphous among the 14 NTM species studied (Additional file 2) and even absent in other bacteria genus and thus seemed very promising for primers and probes design. The remaining 8 proteins that were selected, namely ATP synthase subunit A, CMAS coded by the *cmaA1* gene, lipoprotein coding by *lppM* gene, as well as PE, PPE and proteins coded by *esx* genes *esxG*, *esxH* and *esxR*, were highly conserved in studies MTC species (*tuberculosis* and *bovis*) but very polymorphous in the 14 NTM species studied (Additional file 1), which did not allow us to design specific mycobacterial primers and probes, according to the rules of primer and probe design (Additional file 3).

DNA sequence alignment of the oxidoreductase and of the small secreted protein did not allow design of PCR primers with a minimal length of 18 oligonucleotides (Additional file 3). Only the DNA sequence alignment of the ATP synthase subunits C allowed designing a PCR primer pair and a probe. We designed the following primers and probe: forward primer FatpE 5′-CGGYGCCGGTATCGGYGA-3′ (Tm = 62°C), with the probe PatpE 5′-ACSGTGATGAAGAACGGBGTRAA-3′ (Tm = 68°C) which might be hydrolyzed by the reverse primer RatpE 5′-CGAAGACGAACARSGCCAT-3′ (Tm = 59°C, 182 bp).

### Real-time PCR validation

Based on standard curve comparisons, our results showed reproducible amplification signals with similar Ct values for each genome equivalents of tested mycobacterial strains: *M. avium*, *M. fortuitum*, *M. intracellulare*, *M. gordonae*, and *M. chelonae* (Table 2). Detection limit was estimated at about 6 genome equivalents for *M. chelonae* by real-time PCR reaction by testing repetition of dilution limits (i.e. EC95 value: more than 95% of positive detection for these genome concentration) whereas quantification limits were estimated at about 100 genome equivalents. In the positive collection all 31 mycobacteria species were positively detected by the real-time PCR method. This collection includes NTM species, *leprae* species and MTC species as *tuberculosis* and *bovis* (Table 3). None of the non-mycobacterial environmental strains and none of the CNM collection strains [17], were detected before the end of the 40 PCR cycles (Table 3). These results indicate a sensibility of 100% (31/31) and a specificity of 100% (0/30).

**Table 2:** **Characteristics of *Mycobacterium avium*, *M. fortuitum*, *M. intracellulare*, and *M. chelonae *** **DNA amplification using real-time PCR targeting *atpE *** **gene (locus Rv1305 in *M. tuberculosis *** **genome)**

**Real-time PCR characteristics**	***M. avium***	***M. fortuitum***	***M. intracellulare***	***M. gordonae***	***M. chelonae***
Correlation coefficient *r*^2^ (%)	93.4	97.4	98.1	99.6	99.8
Efficiencies (%)	119	109	119	97	101
QL (ge/reaction)	<100	<100	<100	ND	<100
DL (95%) (ge/reaction)	ND	ND	ND	ND	6

Ct (cycle threshold) set at 0.02. ND stands for not determined, QL for Quantification Limit, and DL for Detection Limit.

**Table 3:** **Detection of the *atpE *** **gene (locus Rv1305 in *M. tuberculosis *** **genome) in different *Mycobacterium *** **species (25 ± 15 ng of DNA) and non-mycobacterial microorganisms (50 ± 15 ng of DNA)**

	**Microorganism codification**^**a**^	**Microorganism**	**Results**
A	CPS MC13	*M. arupense*	Detected
	CPS MC11	*M. austroafricanum*	Detected
	ATCC 25291^T^	*M. avium* subsp. *avium*	Detected
	CIP 1173/P2	*M. bovis* (BCG)	Detected
	ATCC 19977^T^	*M. chelonae* spp. *abscessus*	Detected
	ATCC 35752^T^	*M. chelonae* spp. *chelonae*	Detected
	CIP 105388 ^T^	*M. gadium*	Detected
	ATCC 14470^T^	*M. gordonae*	Detected
	ATCC 6841^T^	*M. fortuitum* spp. *fortuitum*	Detected
	CPS MC8	*M. insubricum*	Detected
	ATCC 15985^T^	*M. intracellulare*	Detected
	ATCC 12478^T^	*M. kansasii*	Detected
	CIP 105465^T^	*M. lentiflavum*	Detected
	THAI 53	*M. leprae*	Detected
	CPS MC10	*M. llatzerense*	Detected
	ATCC 927^T^	*M. marinum*	Detected
	CIP 105223^T^	*M. mucogenicum*	Detected
	CIP 106811^T^	*M. nonchromogenicum*	Detected
	CPS MC6	*M. psychrotolerans*	Detected
	ATCC 14467^T^	*M. peregrinum*	Detected
	CPS MC9	*M. porcinum*	Detected
	CIP 105416^T^	*M. scrofulaceum*	Detected
	CPS MC7	*M. setense*	Detected
	ATCC 25275^T^	*M. simiae*	Detected
	ATCC 19420^T^	*M. smegmatis*	Detected
	ATCC 35799^T^	*M. szulgai*	Detected
	CIP 104321^T^	*M. terrae*	Detected
	CIP 106368	*M. tusciae*	Detected
	ATCC 25618^T^	*M. tuberculosis* (H37Rv)	Detected
	CPS CR08085632	*M. ulcerans*	Detected
	ATCC 19250^T^	*M. xenopi*	Detected
B	CMR SC10	*Acinetobacter* sp.	ND
	CMR SC9	*Aeromonas* sp.	ND
	CMR SC23	*Arthrobacter* sp.	ND
	CMR SC44	*Aspergillus* sp.	ND
	CMR SC5	*Bacillus* sp.	ND
	CMR SC24	*Brevundimonas* sp.	ND
	ATCC 6871^T^	*C. ammoniagenes*	ND
	ATCC 13032^T^	*C. glutamicum*	ND
	ATCC 10700^T^	*C. pseudodiphtheriticum*	ND
	CMR SC35	*Escherishia* sp.	ND
	CMR SC19	*Flavobacterium* sp.	ND
	ATCC 43504^T^	*Helicobacter pylori*	ND
	CMR SC45	*Kocuria* sp.	ND
	CMR SC31	*Leuclercia* sp.	ND
	CMR SC28	*Leucobacter* sp.	ND
	CMR SC29	*Microbacterium* sp.	ND
	CMR SC3	*Micrococcus* sp.	ND
	DSM 44546^T^	*N. cerradoensis*	ND
	DSM 44490^T^	*N. cummidelens*	ND
	IFM 10152	*N. farcinica*	ND
	CMR SC42	*Penicillium* sp.	ND
	CMR SC1	*Pseudomonas* sp.	ND
	CMR SC26	*Rhodococcus* sp.	ND
	CMR SC34	*Serracia fonticola*	ND
	CMR SC22	*Solibacillus* sp.	ND
	CMR SC12	*Staphylococcus caprae*	ND
	CMR SC6	*Staphylococcus hominis*	ND
	CMR SC46	*Staphylococcus lugdunensis*	ND
	CMR SC49	*Streptomyces* sp.	ND
	CMR SC41	*Trichoderma* sp.	ND

TaqMan® real-time PCR amplification was performed using forward primer FatpE, reverse primer RatpE and probe PatpE in duplicate assays. ND stands for not detected sigmoidal curve.

^a^ATCC: American Type Culture Collection; CPS: Collection de la Pitié-Salpêtrière, Paris, France; ^T^: type strain; CIP: Collection de l′Institut Pasteur, Paris, France; CMR: Collection de Microorganismes de Radomski *et al*. 2010 [17], Paris, France; DSM: DSMZ-Deutsche Sammlung von Mikroorganismen und Zellkulturen, Braunschweig, Germany; IFM: Culture Collection of the Research Centre for Pathogenic Fungi and Microbial Toxicoses, Chiba, Japan.

### Environmental analyses

**Figure 3:**
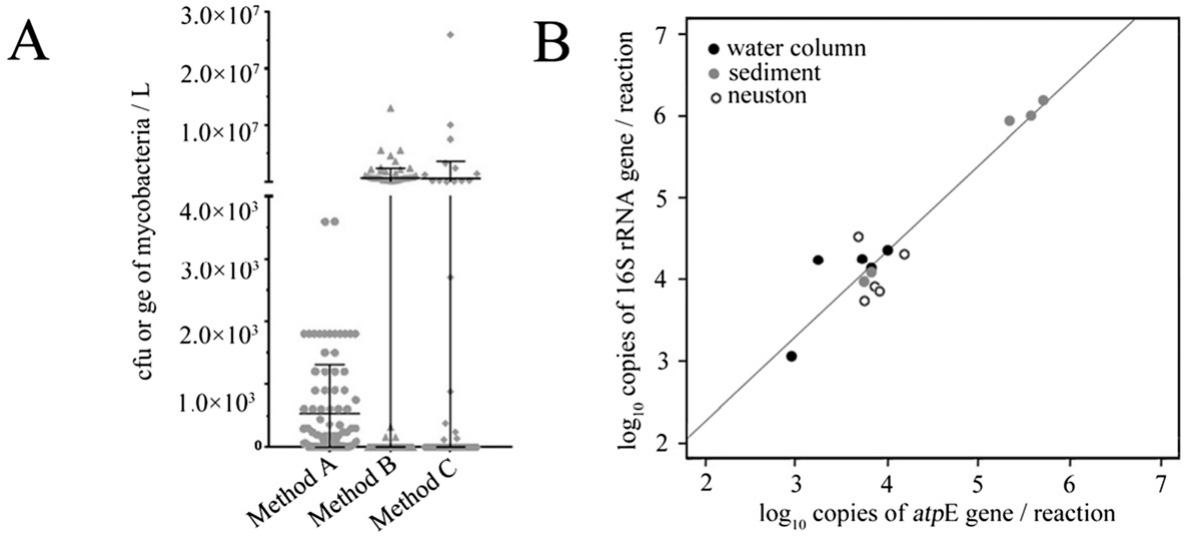
**Mycobacteria quantification in environmental samples and comparison to reference methods. A)** Quantification in drinking water samples (n = 90) was performed by culture method (Method A: Le Dantec *et al*. 2002) [28], and the new real-time PCR targeting the *atpE* gene (locus Rv1305 in *M. tuberculosis* genome) applied to DNA extracted by commercial spin column procedure (Method B: Qiagen kit extraction), or reference DNA extraction procedure (Method C: Radomski *et al*. 2011) [29]. **B)** Quantification in lake samples (n = 15) was performed measured by real-time PCR targeting 16S rRNA (Radomski *et al*. 2010) [17] or *atpE* genes.

In order to compare with culture-based method (Method A) [28], and evaluate the impact of extraction methods on the quantification process by the new real-time PCR, we used two DNA extraction procedures (Method B and C) on water distribution samples: a commercial kit (Method B) and a published phenol-chloroform extraction (Method C) [29]. DNA extraction from tap water significantly influenced the result of mycobacteria detection by *atpE* real-time PCR (Figure 3A). Detection levels from DNA extracted by the kit (Method B) were significantly higher (Wilcoxon signed-rank test, n = 90, p = 0.002) than those from DNA extracted by phenol/chloroform procedure (Method C). The percentage of positive samples was significantly higher (Chi-square test, n = 180, df = 1, p = 0.021) when performing the real-time PCR with the DNA extracted by method B (33/90), compared to method C (19/90). In order to evaluate the new real-time PCR method, we compared the levels of mycobacteria detected in water distribution samples with a published culture method called method A [28]. Using the method A, *Mycobacterium* spp. colonies were obtained from 76% of tap water samples.

Mycobacteria quantification in lake samples by real-time PCR targeting *atpE* gene, shows a vast diversity of mycobacteria concentration, ranging from 10^4^ to 10^6^ ge/L in water column and neuston samples, and 10^5^ to 10^6^ ge/g DW (dry weight) in sediment samples. Comparison with the previously published methods targeting 16S rRNA [17] shows a high correlation between the results (Figure 3B, Correlation test, n = 30, Rs = 0.571, p = 0.028).

## Discussion

Although *gyrA*, *gyrB*, *hsp65*, *recA*, *rpoB*, and *sodA* genes are appropriate for identification purposes [3,4], our results emphasized that these genes seem inappropriate for specific detection of mycobacteria. Indeed, their high similarities with non-mycobacterial genes make specific target design delicate. These new results are in accordance with our previous observations that the molecular targets which were designed based on *gyrB *[18], *rpoB*[19] or *hsp65*[20] genes, had low specificity [17]. For example, the non-related *Helicobacter pylori* show positive amplification with several *Mycobacterium* specific primer pairs [17]. Prospection for more specific targets in mycobacterial genomes seems consequently necessary in order to improve current detection tools based on proteins and/or DNA. The new *atpE* real-time PCR method that we propose is just as specific, but more sensitive than the previously proposed *rrs* real-time PCR method which cannot detect some mycobacterial species [17].

The proposed strategy is aimed at comparing mycobacterial and non-mycobacterial genomic proteins to reference genomic DNA of *M. tuberculosis* H37Rv, sorting proteins according to similarity requests and listing candidate proteins (Figure 1). We chose to perform protein-level comparisons in order to identify exclusively conserved proteins in *Mycobacterium* spp. because non-coding regions, as intergenic regions and insertion sequences, are known to be less conserved than coding regions in *M. tuberculosis* genomes [30]. According to literature, our results emphasized that almost half of the *M. tuberculosis* H37Rv predicted proteins are potentially present in the genomes of CNM group members. More precisely, mycobacteria belong to Actinobacteria which may explain the presence of 48 to 73% shared genes among high G + C content microorganisms [31-34]. In addition, horizontal gene transfers from different bacteria widely present in soil or water, especially *Rhodococcus* sp., *Nocardia* sp. and *Streptomyces* sp. were previously considered to have happened in the *Mycobacterium* genus which may also explain the shared proteins with non-mycobacterial species [24,27,35]. These observations show that CNM group members must be taken into account in order to develop highly specific mycobacterial targets, considering that these bacteria are commonly found in aquatic and terrestrial environments [36,37].

Our study showed that 11 proteins exclusively conserved in the 16 mycobacterial genomes studied could be selected using our genome comparison strategy (i.e. proteins coded by *atpE, atpB*, *cmaA1*, *lppM*, *PE5*, *PPE48*, *esxG*, *esxH* and *esxR* genes, as well as an oxidoreductase and a small secreted protein). Only the *aptE* gene could be used to design primers and a probe for mycobacteria detection. Concerning the other genes, the sequence polymorphism among NTM species did not allow designing molecular targets for *Mycobacterium* spp. detection. However, these genes could be of immunological or pathogenic importance. Indeed, PE and PPE family proteins represent 0.9 to 4.2% of the genome coding capacity of several mycobacteria [22,25,26,35], and are suspected to play a major antigenic role in immune response [38]. PE and PPE family proteins are often associated with mycobacterial *esx* gene clusters, which encode ATP dependent specific secretion system [24] and are required to export specific members of the 6-kDa early secreted antigenic target (ESAT-6) protein family [26]. Together, ATP dependent specific secretion system and ESAT-6 protein family play a major role in the virulence and life cycle of mycobacteria [24,26]. Nevertheless, PE and PPE family proteins, and proteins coded by *esx* gene clusters are very small and polymorphous among genomes of the 11 NTM species compared (Table 1). Mycobacterial cell wall is also important in pathology, and could procure interesting PCR targets. For instance, several studies emphasized that cyclopropanation of the mycolic acids is common among pathogenic mycobacteria but rare among saprophytic species [39]. Although having sufficient length, proteins CMAS coded by the *cmaA1* gene and lipoprotein coded by *lppM* gene in *M. tuberculosis* H37Rv, were also polymorphous among genomes of the 11 NTM species compared (Table 1) and thus could not be used to design a primer pair and a probe (Additional file 2). Nevertheless, polymorphism of mycobacterial mycolic acids is useful for mycobacteria identification [40,41].

The *atpE* gene which codes ATP synthase subunit C in *M. tuberculosis* H37Rv genome (locus Rv1305) is exclusively conserved in the genomes of the 17 mycobacterial species studied (Additional file 2), and its length and relative conservation among mycobacteria make it an adequate molecular target in order to detect *Mycobacterium* genus. It is remarkable to see that the protein coded by *atpE* gene was also the target of the new antimycobacterial compound recently described: diarylquinoline R207910 [42]. This compound shows a specific bactericidal effect on mycobacteria and none in other genera [43]. In addition, our *in vitro* results demonstrated the specificity of the *atpE* gene (locus Rv1305), which codes for the ATP synthase protein subunit C. These results also showed that our strategy of target design based on MycoHit software (Figure 1) gave very useful results for designing highly specific primers and might be applied to other microorganism clusters.

*In vitro* validation of the real-time PCR targeting the *atpE* gene showed a very high specificity and sensitivity, as well as reproducible quantification of different mycobacteria species. The new real-time method was tested on a realistic number of mycobacterial species including several slow and rapid growing NTM, although not all the described mycobacterial species were tested. In addition, application of this real-time PCR method to environmental samples showed that *Mycobacterium* was detected in tap water samples. The discrepancy between the cultural and molecular techniques was previously described for other pathogens, and the lower level of prevalence obtained by the PCR methods was probably due to our concentration and extraction procedures. These protocol steps must be improved to detect low level of NTM even if the used spin column seemed more appropriate for DNA extraction from environmental samples compared to classical phenol-chloroform extraction. Moreover, culture method did not detect higher level of mycobacterial cells compared to the molecular one. Both methods have advantages and drawbacks, and it may explain the differences observed. For instance, molecular methods could detect dead bacteria, or viable but uncultivable bacteria. However, the real-time PCR targeting the *atpE* gene allows more accurate *Mycobacterium* spp. quantification, contrary to culture based method which is subjected to many drawbacks such as decontamination artifact (about 2 log_10_ reduction for *M. chelonae*), slow mycobacteria growth, clumping of mycobacterial cells, high hydrophobicity of mycobacteria and contamination of culture media by other fast growing environmental microorganisms [44].

Comparison of the method targeting *atpE* with previously described method targeting 16S rRNA, [17], showed a high correlation. Moreover the method targeting *atpE* gene presents two major advantages over the method targeting *rrs* gene. First, the new method detects all the tested mycobacterial strains, while the method targeting *rrs* gene cannot detect isolates of *M. celatum*, *M. heckeshornense*, and *M. leprae*[17]. Second, the *atpE* gene is present in a single copy in the *Mycobacterium* genomes, while the 16S rRNA gene is present either in 1 or 2 copies in the genome [45]. When comparing samples it will be simpler to interpret the data with a stable gene copy number, and probably give a better accuracy of the mycobacterial concentration.

One of the limitations of this study is that only 31 mycobacterial species were tested *in vitro* as positive controls whereas more than 150 mycobacterial species have been described so far [1]. To date, we have confirmed the sensitivity of the *atpE* real-time PCR method using a large representative collection of mycobacterial species (31 species, e.g. around 20% of described species), including members of MTC (n = 2), *M. leprae* species (n = 1), slow growing NTM (n = 13), and rapid growing NTM (n = 15). Given the broad diversity of mycobacterial species we have tested in this study, we expect the method to be applicable to all species within the *Mycobacterium* genus. In addition, it is the first time that a sensitive and specific molecular target has been identified based on an *in silico* comparison of 16 mycobacterial (13 species) and 12 non-mycobacterial genomes (4 closely related species).

## Conclusions

In conclusion, although our strategy did not take into account non-coding regions, such as insertion sequences, repetitive units, non-functional RNA, and structural ribosomal RNAs, the comparison of whole bacterial genomes for design of specific primers is a promising approach not only for mycobacteria but also for other cultured bacterial or archaeal groups for which whole sequenced genomes are accumulating in databases. Metagenomic libraries from environmental samples which are increasingly performed in microbial ecology studies [46] could also provide useful data for the design of specific targets toward uncultured Bacteria and *Archaea*. Using these databases, MycoHit, or other new software, may then be used to design new primers for real-time PCR detection or quantification, for *in situ* hybridization and other molecular tools. With this approach we were able to design primer pairs and a probe that target specific mycobacterial *atpE* gene, and could be used to detect and quantify very specifically mycobacteria in environmental samples. Although the *atpE* gene may not be appropriate for microdiversity studies, it appeared to be very useful for specific detection of the genus *Mycobacterium* in environmental samples. More generally, genome comparison used here showed its utility to identify specific genera’s targets, and could be used to identify specific proteins for antimicrobial design as previously emphasized [47].

## Methods

### ***In silico*** comparison strategy

In order to detect *M. tuberculosis* genes, presenting homologue genes in other mycobacterial genomes, and not presenting homologue genes in non-mycobacteria genomes, we used the MycoHit software version 14.17 (Zipped copy of the files and instructions for this application are available in the Behr Research Lab, https://www.mcgill.ca/molepi/) and performed an alignment search with Stand Alone tblastn algorithm as previously described [27]. Stand Alone tblastn algorithm has been chosen because coding sequences are known to be more conserved in mycobacterial genomes than non-coding sequences, as intergenic regions, insertion sequences, or phage sequences [30]. Genome of *M. tuberculosis* H37Rv has been used as a reference of the *Mycobacterium* genus, because it is the most historically described mycobacterial genome [22]. Based on the 3989 predicted proteins from *M. tuberculosis* H37Rv, corresponding to the query sequences used in order to search for matches in the genomic DNA of other organisms (Figure 1), a matrix of 107703 scores (3989 protein sequences blasted against 12 non-mycobacterial genomes and 15 mycobacterial genomes) was obtained. As previously described [27] and according to NCBI procedures [48], expected value was set at e^-10^. Following sequence comparisons, the MycoHit software allowed to sort scores according to similarity requests which were performed on the one hand toward mycobacterial genomes, and on the other hand toward non-mycobacterial genomes (Figure 1). A protein list of the reference target, which can be downloaded from NCBI web site (http://www.ncbi.nlm.nih.gov), allowed identification of the conserved mycobacterial proteins presenting no homology in non-mycobacterial genomes (Figure 1).

### Mycobacterial genome database

In order to perform comparisons of pathogenic (P) and non-pathogenic (NP) mycobacterial genomes with *M. tuberculosis* H37Rv genome using MycoHit software, sequences were obtained at NCBI web site (http://www.ncbi.nlm.nih.gov) using the accession numbers: *M. abscessus* ATCC 19977 (CU458896.1) (P), *M. avium* 104 (CP000479.1) (P), *M. avium* subsp. *paratuberculosis* K10 (AE016958.1) (P), *M. bovis* subsp. *bovis* AF2122/97 (BX248333.1) (P), *M. gilvum* PYR-GCK (CP000656.1) (NP), *M. marinum* M (CP000854.1) (P), *M. smegmatis* MC2 155 (CP000480.1) (NP), *Mycobacterium* sp. JLS (CP000580.1) (NP), *Mycobacterium* sp. KMS (CP000518.1) (NP), *Mycobacterium* sp. MCS (CP000384.1) (NP), *M. tuberculosis* CDC1551 (AE000516.2) (P), *M. tuberculosis* H37Ra (CP000611.1) (NP), *M. tuberculosis* H37Rv (AL123456.2) (P), *M. tuberculosis* KZN 1435 (CP001658.1) (P), *M. ulcerans* Agy99 (CP000325.1) (P), and *M. vanbaalenii* PYR-1 (CP000511.1) (P). In order to avoid data lost during genome comparisons performed by MycoHit software, we have chosen to ignore some mycobacterial genomes. Since the number of coding proteins is much lower compared to other mycobacterial species, *M. leprae* Br4923 (FM211192.1) (P), and *M. leprae* TN (AL450380.1) (P) were ignored in the analysis (e.g. 1604 coding proteins in *M. leprae* Br4923 or 1605 coding proteins in *M. leprae* TN, against 6716 coding proteins in *M. smegmatis* MC2 155) [22,24-26,35]. Genomes of *M. bovis* BCG Pasteur 1173P2 (AM408590.1) (NP) and *M. bovis* BCG Tokyo 172 (AP010918.1) (NP) were also not taken into account, because these vicinal genomes present mutations [49]. Moreover, genomes of *M. intracellulare* ATCC 13950 (ABIN00000000) (P), *M. kansasii* ATCC 12478 (ACBV00000000) (P) and *M. parascrofulaceum* BAA-614 (ADNV00000000) (P) were also not used during MycoHit proceedings, because their genomes were still not assembled at the moment we performed the first screening step of our analysis. Nevertheless, the genomes of *M. leprae*, *M. bovis* BCG, *M. intracellulare*, *M. kansasii* and *M. parascrofulaceum* were used during alignment of nucleic sequences of the most conserved proteins in mycobacterial genomes.

### Non-mycobacterial genome database

We selected non-mycobacterial genomes of species from the CNM group using the following accession numbers: *Corynebacterium aurimucosum* ATCC 700975 (CP001601.1), *C. diphtheriae* NCTC 13129 (BX248353.1), *C. efficiens* YS-314 (BA000035.2), *C. glutamicum* ATCC 13032 (BX927147.1), *C. jeikeium* K411 (NC_007164), *C. kroppenstedtii* DSM 44385 (CP001620.1), *C. urealyticum* DSM 7109 (AM942444.1), *Nocardia farcinica* IFM 10152 (AP006618.1), *Nocardioides* sp. JS614 (CP000509.1), *Rhodococcus erythropolis* PR4 (AP008957.1), *R. jostii* RHA1 (CP000431.1), and *R. opacus* B4 (AP011115.1).

### Primer pair and probe design

In order to check the homology of the selected mycobacterial sequences, the protein and DNA sequences of these selected proteins were aligned using the ClustalW multiple alignment of the BioEdit software 7.0.9.0 with 1000 bootstraps [50]. Primer pair and probe was designed from the best fitted gene sequences (after protein screening and selection) by visual analysis and using the Beacon Designer software version 7.90 (Premier Biosoft International, Palo Alto, Calif.).

### Real-time PCR validation

Reproducibility, sensitivity and specificity of the new real-time PCR method were estimated using DNA from a previously described microorganism collection, and according to Radomski *et al*. protocol [17]. Reproducibility, efficiency, limits of detection and quantification of the real-time PCR methods [44] were estimated by quantification of several tenfold dilutions (10 replications of 400, 100, 40, 20, 4, 0.4 and 0.04 genome equivalent (ge) by reaction) of a known quantity of DNA extracted from four strains: *M. avium*, *M. fortuitum*, *M. intracellulare* and *M. gordonae* (identified from the national French reference laboratory collection). Specificity and sensitivity were estimated against 30 non-mycobacteria (negative) strains and 31 mycobacteria (positive), respectively. The collection contained reference and environmental strains of mycobacteria, as well as, strains of the closely related CNM group, and other non-actinobacteria strains isolated from the environment [17]. Mycobacteria collection included MTC (n = 2) and *leprae* species (n = 1), as well as species of slow growing NTM (n = 13), and rapid growing NTM (n = 15). TaqMan® real-time PCR were performed in duplicate using an ABI7500 real-time PCR system (Applied Biosystems), a Lifetech 7500 software version 2.0.6 (Applied Biosystems) and TaqMan fast virus 1-STEP Master Mix with 6-carboxy-X-rhodamine (ROX) (Applied Biosystems). The TaqMan® probes were labeled (Eurogentec) with the fluorescent dyes 6-carboxyfluorescein (5′ end) and Black Hole Quencher (3′ end). All reactions were performed in a 25 μl reaction mixture volume (2.5 μl of DNA) with 500 nM of forward primer, 500 nM of reverse primer, 50 nM of probe and 5 mM of MgCl_2_. Reverse transcriptase was inactivated immediately (95 °C, 45 s) according to the manufacturer instruction, and real-time PCR consisted in 40 cycles of denaturation (95°C for 3 s), annealing and extension (both steps at 60°C for 30 s). Determinations of cycle threshold were performed by setting the instrument’s threshold line at 0.02 ∆Rn units (fluorescence gain above the baseline divided by the ROX channel signal).

### Environmental analyses

In order to compare the new real-time PCR method to the culture method, 26 tap water distribution points in Paris (France) were sampled between April 2011 and July 2011, corresponding to 90 samples. Briefly, one liter of tap water was sampled in sterile plastic bottle, then centrifuged at 5000 × g for 2h and finally re-suspended in 1 ml of water. Mycobacteria density was estimated by culture (Method A) in all these samples following the procedure previously described by Le Dantec *et al*. [28]. In parallel, DNA was extracted using two different methods: i) a bacterial DNA extraction kit (QIAamp DNA mini kit, Qiagen) according to the manufacturer recommendations (Method B), and ii) a phenol-chloroform extraction procedure according to Radomski *et al*. [29] (Method C). Extracted DNA was 10 fold diluted and mycobacteria density was estimated in duplicate using the new real-time PCR method.

Using environmental samples, the new *atpE* targeting method was also compared a previously described *rrs* targeting method [17]. More precisely, samples collected from water column, sediment, and neuston of two urbanized lakes (Daumesnil Lake, Paris, France, and Créteil Lake, Créteil, France) were analyzed in triplicate. Water samples (column and neuston) were centrifuged 1 h at 7500 × g, and DNA was extracted using a MagNA Pure System (Roche). Sediment samples were lyophilized and DNA was isolated using FastDNA SPIN kit for Soil according to the manufacturer’s instructions (MP Biomedicals, Santa Ana, CA). Statistical analyses were carried out using R software v. 2.15 [51].

## Availability of supporting data

The data sets supporting the results of this article are included within the article and its additional files.

## Abbreviations

ATP: Adenosine triphosphate; CMAS: Cyclopropane mycolic acid synthase; CNM: *Corynebacterium*, *Nocardia*, *Rhodococcus*, *Mycobacterium*; ITS: Internal transcribed spacer; MTC: *Mycobacterium tuberculosis* complex; NP: Non-pathogenic; NTM: Non-tuberculous mycobacteria; P: Pathogenic.

## Competing interests

The authors declare that they have no competing interests.

## Authors’ contributions

NR contributed in the experimental design, data acquisition and interpretation under the supervisions of FL, RM, EC, and LM, and was involved in writing the manuscript. For *in silico* comparisons, FJV provided technical assistance and MAB supervised data interpretation. HA and LM carried out the *in vitro* assays, and participated to statistical analyses and manuscript writing. All authors read and approved the manuscript.

## Supplementary Material

Additional file 1 **Similarities (%) between *Mycobacterium tuberculosis *H37Rv (AL123456.2) proteins and proteins of targeted mycobacterial genomes and proteins of non-targeted genomes.** Targeted mycobacterial genomes include *M. tuberculosis* H37Ra (CP000611.1), *M. tuberculosis* CDC 1551 (AE000516.2), *M. tuberculosis* KZN 1435 (CP001658.1), *M. bovis* AF2122/97 (BX248333.1), *M. ulcerans* Agy99 (CP000325.1), *M. marinum* M (CP000854.1), *M. avium* 104 (CP000479.1), *M. paratuberculosis* K10 (AE016958.1), *M. smegmatis* MC2 155 (CP000480.1), *M. abscessus* ATCC 19977 (CU458896.1), *M. gilvum* PYG-GCK (CP000656.1), *M. vanbaalenii* PYR-1 (CP000511.1), *Mycobacterium* sp. JLS (CP000580.1), *Mycobacterium* sp. KMS (CP000518.1), *Mycobacterium* sp. MCS (CP000384.1), and non-targeted genomes include *Corynebacterium aurimucosum* ATCC 700975 (CP001601.1), *C. diphteriae* NCTC 13129 (BX248353.1), *C. efficiens* YS-314 (BA000035.2), *C. glutamicum* ATCC 13032 (BX927147.1), *C. jeikeium* K411 (NC_007164), *C. kroppenstedtii* DSM 44385 (CP001620.1), *C. urealyticum* DSM 7109 (AM942444.1), *Nocardia farcinica* IFM 10152 (AP006618.1), *Nocardioides* sp. JS614 (CP000509.1), *Rhodococcus erythropolis* PR4 (AP008957.1), *R. jostii* RHA1 (CP000431.1) and *R. opacus* B4 (AP011115.1). Click here for file

Additional file 2 **Protein sequence alignment of conserved proteins in mycobacterial genomes.** Sequences are from genomes of *M. abscessus* ATCC 19977 (CU458896.1), *M. avium* 104 (CP000479.1), *M. avium* subsp. *paratuberculosis* K10 (AE016958.1), *M. bovis* subsp. *bovis* AF2122/97 (BX248333.1), *M. bovis* BCG Pasteur 1173P2 (AM408590.1), *M. bovis* BCG Tokyo 172 (AP010918.1), *M. gilvum* PYR-GCK (CP000656.1), *M. intracellulare* ATCC 13950 (ABIN00000000), *M. kansasii* ATCC 12478 (ACBV00000000), *M. leprae* Br4923 (FM211192.1), *M. leprae* TN (AL450380.1), *M. marinum* M (CP000854.1), *M. parascrofulaceum* BAA-614 (ADNV00000000), *M. smegmatis* MC2 155 (CP000480.1), *Mycobacterium* sp. JLS (CP000580.1), *Mycobacterium* sp. KMS (CP000518.1), *Mycobacterium* sp. MCS (CP000384.1), *M. tuberculosis* CDC1551 (AE000516.2), *M. tuberculosis* H37Ra (CP000611.1), *M. tuberculosis* H37Rv (AL123456.2), *M. tuberculosis* KZN 1435 (CP001658.1), *M. ulcerans* Agy99 (CP000325.1) and *M. vanbaalenii* PYR-1 (CP000511.1). Click here for file

Additional file 3 **DNA sequence alignment of conserved proteins in mycobacterial genomes.** Sequences are from genomes of *M. abscessus* ATCC 19977 (CU458896.1), *M. avium* 104 (CP000479.1), *M. avium* subsp. *paratuberculosis* K10 (AE016958.1), *M. bovis* subsp. *bovis* AF2122/97 (BX248333.1), *M. bovis* BCG Pasteur 1173P2 (AM408590.1), *M. bovis* BCG Tokyo 172 (AP010918.1), *M. gilvum* PYR-GCK (CP000656.1), *M. intracellulare* ATCC 13950 (ABIN00000000), *M. kansasii* ATCC 12478 (ACBV00000000), *M. leprae* Br4923 (FM211192.1), *M. leprae* TN (AL450380.1), *M. marinum* M (CP000854.1), *M. parascrofulaceum* BAA-614 (ADNV00000000), *M. smegmatis* MC2 155 (CP000480.1), *Mycobacterium* sp. JLS (CP000580.1), *Mycobacterium* sp. KMS (CP000518.1), *Mycobacterium* sp. MCS (CP000384.1), *M. tuberculosis* CDC1551 (AE000516.2), *M. tuberculosis* H37Ra (CP000611.1), *M. tuberculosis* H37Rv (AL123456.2), *M. tuberculosis* KZN 1435 (CP001658.1), *M. ulcerans* Agy99 (CP000325.1) and *M. vanbaalenii* PYR-1 (CP000511.1). Click here for file
