# Supplementary material for: atpE gene as a new useful specific molecular target to quantify Mycobacterium in environmental samples
Source: BMC Microbiol. 2013 Dec 3;13:277. doi: 10.1186/1471-2180-13-277 (PMC4219376; doi:10.1186/1471-2180-13-277)
Supplement: Additional file 1 — Similarities (%) between Mycobacterium tuberculosis H37Rv (AL123456.2) proteins and proteins of targeted mycobacterial genomes and proteins of non-targeted genomes. Targeted mycobacterial genomes include M. tuberculosis H37Ra (CP000611.1), M. tuberculosis CDC 1551 (AE000516.2), M. tuberculosis KZN 1435 (CP001658.1), M. bovis AF2122/97 (BX248333.1), M. ulcerans Agy99 (CP000325.1), M. marinum M (CP000854.1), M. avium 104 (CP000479.1), M. paratuberculosis K10 (AE016958.1), M. smegmatis MC2 155 (CP000480.1), M. abscessus ATCC 19977 (CU458896.1), M. gilvum PYG-GCK (CP000656.1), M. vanbaalenii PYR-1 (CP000511.1), Mycobacterium sp. JLS (CP000580.1), Mycobacterium sp. KMS (CP000518.1), Mycobacterium sp. MCS (CP000384.1), and non-targeted genomes include Corynebacterium aurimucosum ATCC 700975 (CP001601.1), C. diphteriae NCTC 13129 (BX248353.1), C. efficiens YS-314 (BA000035.2), C. glutamicum ATCC 13032 (BX927147.1), C. jeikeium K411 (NC_007164), C. kroppenstedtii DSM 44385 (CP001620.1), C. urealyticum DSM 7109 (AM942444.1), Nocardia farcinica IFM 10152 (AP006618.1), Nocardioides sp. JS614 (CP000509.1), Rhodococcus erythropolis PR4 (AP008957.1), R. jostii RHA1 (CP000431.1) and R. opacus B4 (AP011115.1). [file 1471-2180-13-277-S1.pdf]

**Additional file 1 - Similarities (%) between *Mycobacterium tuberculosis* H37Rv (AL123456.2) proteins and proteins of targeted mycobacterial genomes including *M. tuberculosis* H37Ra (CP000611.1), *M. tuberculosis* CDC 1551 (AE000516.2), *M. tuberculosis* KZN 1435 (CP001658.1), *M. bovis* AF2122/97 (BX248333.1), *M. ulcerans* Agy99 (CP000325.1), *M. marinum* M (CP000854.1), *M. avium* 104 (CP000479.1), *M. paratuberculosis* K10 (AE016958.1), *M. smegmatis* MC2 155 (CP000480.1), *M. abscessus* ATCC 19977 (CU458896.1), *M. gilvum* PYG-GCK (CP000656.1), *M. vanbaalenii* PYR-1 (CP000511.1), *Mycobacterium* sp. JLS (CP000580.1), *Mycobacterium* sp. KMS (CP000518.1), *Mycobacterium* sp. MCS (CP000384.1), and proteins of non-targeted genomes including *Corynebacterium aurimucosum* ATCC 700975 (CP001601.1), *C. diphteriae* NCTC 13129 (BX248353.1), *C. efficiens* YS-314 (BA000035.2), *C. glutamicum* ATCC 13032 (BX927147.1), *C. jeikeium* K411 (NC_007164), *C. kroppenstedtii* DSM 44385 (CP001620.1), *C. urealyticum* DSM 7109 (AM942444.1), *Nocardia farcinica* IFM 10152 (AP006618.1), *Nocardioides* sp. JS614 (CP000509.1), *Rhodococcus erythropolis* PR4 (AP008957.1), *R. jostii* RHA1 (CP000431.1) and *R. opacus* B4 (AP011115.1).**

| Rv number | gene name | protein length | *C. aurimucosum* | *C. diphteriae* | *C. efficiens* | *C. glutamicum* | *C. jeikeium* | *C. kroppenstedtii* | *C. urealyticum* | *Nocardioides* | *N. farcinica* | *R. erythropolis* | *R. jostii* | *R. opacus* | *M. abscessus* | *M. paratuberculosis* | *M. marinum* | *M. avium* | *M. bovis* | *M. gilvum* | *Mycobacterium* sp. JLS | *Mycobacterium* sp. KMS | *Mycobacterium* sp. MCS | *M. smegmatis* | *M. tuberculosis CDC1551* | *M. tuberculosis H37Ra* | *M. tuberculosis KZN 1435* | *M. ulcerans* | *M. vanbaalenii* |
|---|---|---|---|---|---|---|---|---|---|---|---|---|---|---|---|---|---|---|---|---|---|---|---|---|---|---|---|---|---|
| Rv0001 | *dnaA* | 507 | 61 | 66 | 66 | 66 | 86 | 84 | 85 | 74 | 76 | 75 | 75 | 76 | 86 | 87 | 87 | 87 | 99 | 87 | 90 | 90 | 90 | 89 | 99 | 99 | 99 | 87 | 88 |
| Rv0002 | *dnaN* | 402 | 68 | 68 | 70 | 70 | 71 | 72 | 70 | 66 | 79 | 81 | 81 | 81 | 83 | 92 | 90 | 92 | 99 | 86 | 86 | 86 | 86 | 86 | 100 | 100 | 100 | 89 | 87 |
| Rv0003 | *recF* | 385 | 67 | 66 | 69 | 71 | 66 | 67 | 62 | 63 | 71 | 71 | 70 | 70 | 79 | 86 | 86 | 86 | 99 | 83 | 82 | 83 | 83 | 81 | 99 | 100 | 99 | 87 | 84 |
| Rv0004 | - | 187 | 50 | 57 | 53 | 57 | 53 | 59 | 43 | 54 | 75 | 76 | 77 | 77 | 84 | 83 | 83 | 83 | 99 | 77 | 82 | 82 | 82 | 79 | 100 | 100 | 100 | 82 | 73 |
| Rv0005 | *gyrB* | 714 | 82 | 82 | 83 | 81 | 85 | 85 | 85 | 74 | 91 | 89 | 90 | 91 | 94 | 92 | 95 | 92 | 100 | 93 | 88 | 88 | 88 | 89 | 100 | 100 | 100 | 95 | 93 |
| Rv0006 | *gyrA* | 838 | 85 | 85 | 84 | 85 | 85 | 86 | 84 | 74 | 91 | 91 | 92 | 91 | 95 | 97 | 96 | 97 | 99 | 94 | 95 | 94 | 94 | 95 | 99 | 100 | 99 | 96 | 94 |
| Rv0007 | - | 304 | 51 | 45 | 44 | 51 | 48 | 0 | 49 | 61 | 50 | 50 | 48 | 44 | 54 | 70 | 72 | 72 | 100 | 60 | 62 | 62 | 62 | 60 | 100 | 100 | 100 | 75 | 60 |
| Rv0008c | - | 145 | 0 | 0 | 0 | 0 | 0 | 0 | 0 | 0 | 0 | 0 | 0 | 0 | 54 | 76 | 83 | 75 | 100 | 70 | 76 | 76 | 76 | 62 | 100 | 100 | 100 | 81 | 72 |
| Rv0009 | *ppiA* | 182 | 84 | 84 | 82 | 86 | 84 | 87 | 81 | 77 | 52 | 85 | 88 | 89 | 91 | 95 | 95 | 96 | 100 | 92 | 94 | 94 | 94 | 94 | 100 | 100 | 100 | 95 | 94 |
| Rv0010c | - | 141 | 0 | 0 | 0 | 0 | 0 | 0 | 0 | 0 | 50 | 49 | 55 | 54 | 65 | 85 | 83 | 83 | 100 | 75 | 83 | 83 | 83 | 78 | 100 | 100 | 100 | 86 | 74 |
| Rv0011c | - | 93 | 61 | 59 | 57 | 57 | 57 | 57 | 57 | 0 | 71 | 75 | 72 | 72 | 79 | 100 | 96 | 100 | 100 | 84 | 88 | 88 | 88 | 84 | 100 | 100 | 100 | 96 | 83 |
| Rv0012 | - | 262 | 0 | 0 | 0 | 0 | 0 | 0 | 0 | 49 | 0 | 68 | 68 | 68 | 76 | 88 | 84 | 88 | 99 | 77 | 82 | 82 | 82 | 81 | 99 | 99 | 99 | 83 | 81 |
| Rv0013 | *trpG* | 232 | 55 | 56 | 58 | 59 | 66 | 73 | 68 | 57 | 81 | 79 | 81 | 77 | 85 | 87 | 87 | 87 | 100 | 80 | 83 | 82 | 82 | 84 | 100 | 100 | 100 | 86 | 87 |
| Rv0014c | *pknB* | 626 | 55 | 56 | 56 | 56 | 53 | 55 | 52 | 58 | 67 | 66 | 68 | 68 | 77 | 92 | 92 | 92 | 100 | 84 | 85 | 85 | 85 | 80 | 100 | 100 | 100 | 94 | 83 |
| Rv0015c | *pknA* | 431 | 57 | 56 | 58 | 57 | 52 | 60 | 54 | 58 | 71 | 75 | 73 | 73 | 84 | 76 | 83 | 89 | 100 | 82 | 84 | 84 | 84 | 90 | 100 | 100 | 99 | 84 | 86 |

| | | | | | | | | | | | | | | | | | | | | | | | | | | | | | |
|---|---|---|---|---|---|---|---|---|---|---|---|---|---|---|---|---|---|---|---|---|---|---|---|---|---|---|---|---|---|
| Rv0016c | *pbpA* | 491 | 56 | 60 | 58 | 58 | 62 | 61 | 60 | 58 | 75 | 76 | 76 | 76 | 79 | 92 | 94 | 92 | 100 | 86 | 87 | 87 | 87 | 88 | 100 | 100 | 100 | 93 | 86 |
| Rv0017c | *rodA* | 469 | 67 | 67 | 66 | 66 | 66 | 68 | 66 | 64 | 78 | 78 | 78 | 78 | 80 | 91 | 94 | 91 | 100 | 87 | 87 | 87 | 87 | 87 | 100 | 100 | 100 | 94 | 87 |
| Rv0018c | *ppp* | 514 | 65 | 61 | 61 | 61 | 58 | 52 | 53 | 48 | 70 | 74 | 71 | 70 | 74 | 84 | 84 | 84 | 99 | 80 | 79 | 79 | 79 | 79 | 99 | 100 | 99 | 89 | 81 |
| Rv0019c | - | 155 | 54 | 55 | 57 | 55 | 55 | 54 | 52 | 59 | 83 | 83 | 83 | 83 | 86 | 95 | 96 | 95 | 100 | 89 | 92 | 92 | 92 | 92 | 100 | 100 | 100 | 96 | 90 |
| Rv0020c | *TB39.8* | 527 | 67 | 76 | 78 | 75 | 46 | 55 | 40 | 51 | 54 | 54 | 53 | 52 | 60 | 76 | 82 | 76 | 98 | 67 | 69 | 69 | 69 | 68 | 98 | 100 | 100 | 84 | 68 |
| Rv0021c | - | 322 | 46 | 0 | 50 | 0 | 52 | 43 | 36 | 49 | 52 | 53 | 62 | 63 | 64 | 51 | 69 | 67 | 99 | 53 | 50 | 49 | 49 | 51 | 100 | 100 | 100 | 69 | 54 |
| Rv0022c | *whiB5* | 139 | 0 | 0 | 0 | 0 | 0 | 0 | 0 | 0 | 0 | 0 | 0 | 0 | 0 | 87 | 86 | 88 | 100 | 0 | 0 | 0 | 0 | 0 | 100 | 100 | 100 | 84 | 0 |
| Rv0023 | - | 256 | 0 | 0 | 0 | 0 | 0 | 0 | 0 | 0 | 0 | 0 | 0 | 0 | 0 | 93 | 91 | 91 | 100 | 0 | 0 | 0 | 0 | 0 | 100 | 100 | 100 | 88 | 0 |
| Rv0024 | - | 281 | 46 | 45 | 67 | 66 | 61 | 50 | 57 | 48 | 45 | 60 | 64 | 61 | 47 | 76 | 76 | 76 | 100 | 43 | 45 | 42 | 42 | 60 | 100 | 100 | 100 | 73 | 44 |
| Rv0025 | - | 120 | 0 | 0 | 0 | 0 | 0 | 0 | 0 | 0 | 0 | 0 | 0 | 0 | 0 | 73 | 77 | 75 | 99 | 0 | 0 | 0 | 0 | 0 | 100 | 100 | 100 | 77 | 0 |
| Rv0026 | - | 448 | 0 | 0 | 0 | 0 | 0 | 0 | 0 | 0 | 0 | 0 | 0 | 0 | 0 | 67 | 65 | 67 | 99 | 0 | 0 | 0 | 0 | 0 | 99 | 100 | 99 | 64 | 0 |
| Rv0027 | - | 105 | 0 | 0 | 0 | 0 | 0 | 0 | 0 | 0 | 0 | 0 | 0 | 0 | 0 | 83 | 90 | 83 | 100 | 0 | 0 | 0 | 0 | 0 | 100 | 100 | 100 | 89 | 0 |
| Rv0028 | - | 101 | 0 | 0 | 0 | 0 | 0 | 0 | 0 | 0 | 0 | 0 | 0 | 0 | 0 | 93 | 91 | 94 | 100 | 0 | 0 | 0 | 0 | 0 | 100 | 100 | 100 | 91 | 0 |
| Rv0029 | - | 365 | 0 | 0 | 0 | 0 | 0 | 0 | 0 | 0 | 0 | 0 | 0 | 0 | 0 | 63 | 66 | 62 | 100 | 0 | 0 | 0 | 0 | 0 | 88 | 100 | 100 | 65 | 0 |
| Rv0030 | - | 109 | 0 | 0 | 0 | 0 | 0 | 0 | 0 | 0 | 0 | 0 | 0 | 0 | 0 | 87 | 87 | 87 | 100 | 0 | 0 | 0 | 0 | 0 | 100 | 100 | 100 | 90 | 0 |
| Rv0031 | - | 70 | 0 | 0 | 0 | 0 | 0 | 0 | 0 | 0 | 0 | 0 | 0 | 0 | 0 | 0 | 0 | 0 | 100 | 0 | 0 | 0 | 0 | 0 | 100 | 100 | 100 | 0 | 0 |
| Rv0032 | *bioF2* | 771 | 0 | 51 | 0 | 0 | 0 | 47 | 0 | 54 | 53 | 54 | 52 | 55 | 55 | 50 | 56 | 50 | 100 | 52 | 51 | 51 | 51 | 52 | 100 | 100 | 100 | 56 | 51 |
| Rv0033 | *acpA* | 87 | 0 | 0 | 0 | 0 | 0 | 0 | 0 | 0 | 0 | 0 | 0 | 0 | 0 | 0 | 0 | 0 | 100 | 0 | 0 | 0 | 0 | 0 | 100 | 100 | 100 | 0 | 0 |
| Rv0034 | - | 131 | 0 | 0 | 0 | 0 | 0 | 0 | 0 | 0 | 0 | 0 | 0 | 0 | 0 | 0 | 0 | 0 | 100 | 0 | 0 | 0 | 0 | 0 | 100 | 100 | 100 | 0 | 0 |
| Rv0035 | *fadD34* | 562 | 42 | 43 | 44 | 43 | 45 | 0 | 43 | 43 | 43 | 42 | 42 | 41 | 45 | 44 | 43 | 45 | 100 | 46 | 43 | 43 | 43 | 46 | 100 | 100 | 99 | 43 | 45 |
| Rv0036c | - | 257 | 0 | 0 | 0 | 0 | 0 | 0 | 0 | 64 | 66 | 71 | 74 | 74 | 77 | 85 | 88 | 85 | 100 | 79 | 80 | 80 | 80 | 82 | 100 | 100 | 100 | 87 | 79 |
| Rv0037c | - | 441 | 0 | 0 | 0 | 0 | 0 | 0 | 0 | 40 | 52 | 60 | 59 | 59 | 73 | 88 | 88 | 88 | 100 | 80 | 80 | 80 | 80 | 79 | 99 | 99 | 100 | 90 | 82 |
| Rv0038 | - | 202 | 62 | 61 | 63 | 62 | 0 | 58 | 0 | 60 | 83 | 83 | 83 | 83 | 89 | 95 | 95 | 95 | 100 | 91 | 93 | 93 | 93 | 90 | 100 | 100 | 100 | 95 | 92 |
| Rv0039c | - | 115 | 0 | 0 | 0 | 0 | 0 | 0 | 0 | 0 | 0 | 0 | 0 | 0 | 47 | 83 | 82 | 83 | 99 | 66 | 63 | 63 | 63 | 60 | 100 | 100 | 100 | 82 | 66 |
| Rv0040c | *mtc28* | 310 | 0 | 0 | 0 | 0 | 0 | 0 | 0 | 0 | 0 | 0 | 0 | 0 | 55 | 69 | 69 | 68 | 100 | 64 | 67 | 67 | 67 | 63 | 100 | 100 | 100 | 70 | 66 |
| Rv0041 | *leuS* | 969 | 77 | 77 | 78 | 78 | 74 | 73 | 72 | 47 | 82 | 82 | 82 | 82 | 85 | 91 | 91 | 91 | 100 | 86 | 87 | 87 | 87 | 88 | 100 | 100 | 100 | 92 | 85 |
| Rv0042c | - | 208 | 53 | 0 | 0 | 0 | 55 | 47 | 54 | 44 | 49 | 47 | 47 | 48 | 54 | 91 | 84 | 91 | 100 | 63 | 81 | 81 | 81 | 80 | 100 | 100 | 100 | 84 | 79 |
| Rv0043c | - | 244 | 0 | 0 | 0 | 0 | 0 | 0 | 0 | 0 | 0 | 51 | 49 | 50 | 0 | 90 | 90 | 90 | 100 | 74 | 74 | 74 | 74 | 79 | 100 | 100 | 100 | 89 | 76 |
| Rv0044c | - | 264 | 0 | 0 | 0 | 0 | 0 | 0 | 0 | 45 | 73 | 76 | 75 | 73 | 62 | 89 | 88 | 89 | 100 | 84 | 85 | 85 | 85 | 87 | 100 | 100 | 100 | 88 | 84 |
| Rv0045c | - | 298 | 0 | 0 | 0 | 0 | 0 | 0 | 0 | 44 | 41 | 40 | 42 | 40 | 43 | 88 | 88 | 88 | 100 | 78 | 77 | 77 | 77 | 77 | 100 | 100 | 100 | 87 | 79 |
| Rv0046c | *ino1* | 367 | 0 | 87 | 86 | 87 | 83 | 85 | 83 | 87 | 94 | 90 | 92 | 93 | 92 | 96 | 91 | 96 | 99 | 92 | 94 | 94 | 94 | 92 | 99 | 100 | 100 | 92 | 92 |
| Rv0047c | - | 180 | 0 | 0 | 0 | 0 | 0 | 0 | 0 | 66 | 90 | 93 | 89 | 89 | 89 | 98 | 97 | 98 | 100 | 92 | 91 | 91 | 91 | 95 | 100 | 100 | 100 | 97 | 93 |
| Rv0048c | - | 289 | 0 | 0 | 0 | 0 | 0 | 0 | 0 | 0 | 47 | 46 | 46 | 49 | 58 | 78 | 87 | 78 | 99 | 67 | 69 | 69 | 69 | 71 | 99 | 100 | 100 | 87 | 67 |
| Rv0049 | - | 137 | 57 | 59 | 57 | 58 | 54 | 56 | 0 | 0 | 81 | 77 | 81 | 81 | 89 | 94 | 94 | 94 | 100 | 90 | 89 | 89 | 89 | 87 | 100 | 100 | 100 | 95 | 91 |
| Rv0050 | *ponA1* | 678 | 66 | 61 | 64 | 65 | 64 | 66 | 65 | 50 | 72 | 73 | 73 | 73 | 82 | 94 | 95 | 94 | 99 | 86 | 87 | 87 | 87 | 87 | 99 | 99 | 99 | 94 | 86 |
| Rv0051 | - | 560 | 54 | 54 | 55 | 54 | 53 | 56 | 53 | 49 | 68 | 70 | 70 | 70 | 80 | 88 | 84 | 88 | 100 | 83 | 83 | 83 | 83 | 81 | 100 | 100 | 100 | 87 | 81 |
| Rv0052 | - | 187 | 52 | 0 | 0 | 0 | 48 | 50 | 0 | 58 | 59 | 48 | 49 | 54 | 59 | 80 | 82 | 80 | 100 | 62 | 59 | 59 | 59 | 58 | 100 | 100 | 100 | 81 | 62 |
| Rv0053 | *rpsF* | 96 | 81 | 82 | 80 | 80 | 82 | 81 | 80 | 85 | 87 | 90 | 92 | 92 | 91 | 97 | 97 | 97 | 100 | 96 | 97 | 97 | 97 | 96 | 100 | 100 | 100 | 96 | 96 |

| | | | | | | | | | | | | | | | | | | | | | | | | | | | | | |
|---|---|---|---|---|---|---|---|---|---|---|---|---|---|---|---|---|---|---|---|---|---|---|---|---|---|---|---|---|---|
| Rv0054 | *ssb* | 164 | 73 | 70 | 57 | 59 | 66 | 68 | 64 | 70 | 83 | 84 | 85 | 85 | 87 | 93 | 89 | 91 | 100 | 88 | 87 | 87 | 87 | 89 | 100 | 100 | 100 | 91 | 89 |
| Rv0055 | *rpsR* | 84 | 71 | 66 | 68 | 68 | 69 | 70 | 71 | 79 | 77 | 78 | 78 | 80 | 91 | 97 | 98 | 97 | 100 | 92 | 95 | 95 | 95 | 95 | 100 | 100 | 100 | 98 | 93 |
| Rv0056 | *rplI* | 152 | 78 | 80 | 74 | 72 | 74 | 70 | 71 | 69 | 82 | 81 | 81 | 82 | 82 | 94 | 95 | 95 | 100 | 86 | 86 | 86 | 86 | 87 | 100 | 100 | 100 | 94 | 85 |
| Rv0057 | - | 173 | 0 | 0 | 0 | 0 | 0 | 0 | 0 | 0 | 0 | 0 | 0 | 0 | 0 | 59 | 58 | 58 | 100 | 0 | 0 | 0 | 0 | 0 | 100 | 100 | 100 | 57 | 0 |
| Rv0058 | *dnaB* | 874 | 80 | 81 | 78 | 77 | 77 | 77 | 77 | 77 | 83 | 88 | 90 | 62 | 95 | 95 | 95 | 95 | 99 | 90 | 94 | 92 | 92 | 79 | 99 | 100 | 99 | 97 | 90 |
| Rv0059 | - | 230 | 0 | 0 | 0 | 0 | 0 | 0 | 0 | 0 | 0 | 0 | 0 | 0 | 0 | 0 | 0 | 0 | 100 | 0 | 0 | 0 | 0 | 0 | 100 | 100 | 99 | 0 | 0 |
| Rv0060 | - | 352 | 0 | 0 | 0 | 0 | 0 | 0 | 0 | 0 | 0 | 0 | 0 | 0 | 0 | 0 | 0 | 0 | 100 | 0 | 0 | 0 | 0 | 0 | 100 | 100 | 100 | 0 | 0 |
| Rv0061 | - | 141 | 0 | 0 | 0 | 0 | 0 | 0 | 0 | 0 | 0 | 0 | 0 | 0 | 0 | 0 | 61 | 0 | 100 | 0 | 0 | 0 | 0 | 0 | 100 | 100 | 100 | 60 | 0 |
| Rv0062 | *celA1* | 380 | 0 | 0 | 0 | 0 | 0 | 0 | 0 | 56 | 0 | 0 | 0 | 0 | 79 | 81 | 81 | 81 | 100 | 70 | 74 | 74 | 74 | 75 | 99 | 100 | 100 | 0 | 72 |
| Rv0063 | - | 479 | 0 | 0 | 0 | 0 | 0 | 0 | 0 | 40 | 39 | 41 | 41 | 42 | 45 | 87 | 91 | 87 | 100 | 40 | 37 | 37 | 37 | 41 | 100 | 100 | 100 | 75 | 50 |
| Rv0064 | - | 979 | 63 | 61 | 61 | 62 | 64 | 64 | 64 | 56 | 74 | 73 | 76 | 77 | 66 | 67 | 67 | 67 | 99 | 66 | 66 | 66 | 66 | 66 | 99 | 99 | 99 | 67 | 66 |
| Rv0065 | - | 133 | 0 | 0 | 0 | 0 | 0 | 0 | 0 | 0 | 0 | 0 | 0 | 0 | 0 | 0 | 0 | 0 | 100 | 55 | 0 | 0 | 0 | 0 | 100 | 100 | 100 | 0 | 0 |
| Rv0066c | *icd2* | 745 | 69 | 72 | 72 | 72 | 72 | 71 | 70 | 72 | 0 | 0 | 91 | 91 | 89 | 91 | 94 | 91 | 99 | 89 | 90 | 89 | 89 | 89 | 100 | 100 | 100 | 94 | 90 |
| Rv0067c | - | 189 | 0 | 0 | 0 | 0 | 52 | 52 | 0 | 55 | 51 | 47 | 51 | 70 | 79 | 53 | 0 | 51 | 100 | 72 | 78 | 78 | 78 | 81 | 100 | 100 | 100 | 0 | 75 |
| Rv0068 | - | 303 | 50 | 46 | 48 | 0 | 43 | 44 | 46 | 57 | 66 | 65 | 68 | 67 | 83 | 83 | 83 | 82 | 100 | 78 | 79 | 79 | 79 | 83 | 100 | 100 | 100 | 83 | 80 |
| Rv0069c | *sdaA* | 461 | 63 | 63 | 0 | 62 | 66 | 68 | 0 | 0 | 0 | 64 | 82 | 80 | 0 | 0 | 89 | 0 | 100 | 64 | 0 | 0 | 0 | 80 | 100 | 100 | 100 | 88 | 0 |
| Rv0070c | *glyA* | 425 | 75 | 77 | 77 | 75 | 76 | 76 | 75 | 54 | 78 | 77 | 87 | 88 | 55 | 76 | 77 | 77 | 99 | 53 | 57 | 57 | 57 | 55 | 100 | 100 | 100 | 95 | 54 |
| Rv0071 | - | 235 | 0 | 0 | 0 | 0 | 0 | 0 | 0 | 0 | 0 | 0 | 0 | 62 | 0 | 0 | 0 | 0 | 98 | 0 | 0 | 0 | 0 | 0 | 98 | 100 | 98 | 0 | 60 |
| Rv0072 | - | 349 | 0 | 40 | 0 | 0 | 0 | 0 | 0 | 45 | 40 | 0 | 40 | 41 | 41 | 0 | 0 | 0 | 100 | 0 | 0 | 0 | 0 | 0 | 100 | 100 | 100 | 0 | 0 |
| Rv0073 | - | 330 | 59 | 59 | 62 | 61 | 63 | 64 | 60 | 64 | 63 | 63 | 63 | 63 | 62 | 62 | 95 | 62 | 91 | 63 | 62 | 62 | 62 | 62 | 100 | 100 | 100 | 59 | 62 |
| Rv0074 | - | 411 | 38 | 0 | 0 | 0 | 0 | 0 | 0 | 39 | 40 | 40 | 39 | 37 | 39 | 44 | 44 | 45 | 100 | 44 | 43 | 43 | 43 | 45 | 100 | 100 | 100 | 43 | 45 |
| Rv0075 | - | 390 | 60 | 62 | 60 | 60 | 60 | 61 | 58 | 53 | 39 | 44 | 45 | 44 | 43 | 52 | 53 | 53 | 100 | 46 | 44 | 44 | 44 | 45 | 100 | 100 | 100 | 53 | 43 |
| Rv0076c | - | 129 | 0 | 0 | 0 | 0 | 0 | 0 | 0 | 0 | 0 | 0 | 0 | 0 | 0 | 67 | 76 | 67 | 100 | 0 | 0 | 0 | 0 | 58 | 100 | 100 | 100 | 0 | 0 |
| Rv0077c | - | 276 | 0 | 0 | 0 | 0 | 0 | 0 | 0 | 40 | 50 | 40 | 53 | 54 | 52 | 88 | 88 | 88 | 100 | 53 | 51 | 51 | 51 | 52 | 100 | 100 | 100 | 86 | 53 |
| Rv0078 | - | 201 | 0 | 0 | 0 | 0 | 0 | 0 | 0 | 51 | 57 | 0 | 0 | 0 | 57 | 90 | 94 | 90 | 100 | 0 | 65 | 65 | 65 | 0 | 100 | 100 | 100 | 92 | 0 |
| Rv0078A | - | 197 | 0 | 0 | 0 | 0 | 0 | 0 | 0 | 0 | 0 | 0 | 0 | 0 | 0 | 0 | 0 | 0 | 100 | 0 | 0 | 0 | 0 | 0 | 100 | 100 | 100 | 0 | 0 |
| Rv0079 | - | 273 | 0 | 0 | 0 | 0 | 0 | 0 | 0 | 0 | 55 | 0 | 0 | 0 | 0 | 0 | 73 | 0 | 100 | 52 | 48 | 48 | 48 | 45 | 100 | 100 | 99 | 71 | 49 |
| Rv0080 | - | 152 | 0 | 0 | 0 | 0 | 0 | 0 | 0 | 47 | 68 | 0 | 0 | 0 | 0 | 0 | 0 | 0 | 99 | 47 | 56 | 56 | 56 | 55 | 100 | 100 | 100 | 0 | 0 |
| Rv0081 | - | 114 | 0 | 0 | 63 | 70 | 0 | 0 | 0 | 70 | 0 | 0 | 0 | 80 | 82 | 0 | 87 | 86 | 100 | 66 | 67 | 67 | 67 | 63 | 100 | 100 | 100 | 87 | 67 |
| Rv0082 | - | 159 | 0 | 0 | 0 | 0 | 0 | 0 | 0 | 78 | 55 | 55 | 56 | 56 | 58 | 58 | 90 | 90 | 100 | 57 | 58 | 58 | 58 | 57 | 100 | 100 | 100 | 84 | 57 |
| Rv0083 | - | 640 | 45 | 43 | 44 | 44 | 46 | 43 | 44 | 61 | 44 | 45 | 43 | 44 | 44 | 42 | 78 | 84 | 99 | 44 | 45 | 46 | 46 | 42 | 100 | 100 | 100 | 77 | 43 |
| Rv0084 | *hycD* | 316 | 0 | 0 | 0 | 0 | 0 | 0 | 0 | 74 | 0 | 0 | 0 | 0 | 0 | 0 | 90 | 91 | 100 | 0 | 0 | 0 | 0 | 0 | 100 | 100 | 100 | 90 | 0 |
| Rv0085 | *hycP* | 220 | 0 | 0 | 0 | 0 | 0 | 0 | 0 | 67 | 0 | 0 | 0 | 0 | 0 | 0 | 84 | 89 | 100 | 0 | 0 | 0 | 0 | 0 | 100 | 100 | 100 | 83 | 0 |
| Rv0086 | *hycQ* | 488 | 44 | 44 | 44 | 43 | 42 | 43 | 40 | 61 | 45 | 45 | 45 | 46 | 45 | 45 | 80 | 84 | 99 | 45 | 42 | 42 | 42 | 45 | 100 | 100 | 100 | 80 | 46 |
| Rv0087 | *hycE* | 492 | 0 | 0 | 0 | 0 | 0 | 0 | 0 | 71 | 38 | 42 | 41 | 41 | 42 | 41 | 80 | 85 | 100 | 40 | 42 | 42 | 42 | 40 | 99 | 100 | 100 | 78 | 40 |
| Rv0088 | - | 224 | 0 | 0 | 0 | 0 | 0 | 0 | 0 | 0 | 0 | 0 | 0 | 0 | 80 | 0 | 88 | 87 | 100 | 65 | 78 | 78 | 78 | 48 | 99 | 100 | 100 | 86 | 49 |
| Rv0089 | - | 197 | 0 | 0 | 0 | 0 | 0 | 0 | 0 | 0 | 54 | 46 | 0 | 0 | 48 | 0 | 82 | 48 | 100 | 0 | 0 | 0 | 0 | 47 | 100 | 100 | 100 | 80 | 46 |
| Rv0090 | - | 256 | 0 | 0 | 0 | 0 | 0 | 0 | 0 | 0 | 0 | 64 | 0 | 0 | 66 | 0 | 87 | 87 | 100 | 0 | 0 | 0 | 0 | 0 | 100 | 100 | 100 | 86 | 0 |

| | | | | | | | | | | | | | | | | | | | | | | | | | | | | | |
|---|---|---|---|---|---|---|---|---|---|---|---|---|---|---|---|---|---|---|---|---|---|---|---|---|---|---|---|---|---|
| Rv0091 | *mtn* | 255 | 0 | 0 | 0 | 0 | 0 | 0 | 0 | 0 | 0 | 0 | 0 | 0 | 0 | 0 | 0 | 0 | 100 | 0 | 0 | 0 | 0 | 74 | 100 | 100 | 100 | 83 | 0 |
| Rv0092 | *ctpA* | 761 | 59 | 63 | 64 | 62 | 63 | 62 | 60 | 62 | 65 | 64 | 66 | 65 | 61 | 60 | 92 | 63 | 99 | 64 | 66 | 65 | 65 | 67 | 100 | 100 | 100 | 91 | 67 |
| Rv0093c | - | 282 | 0 | 0 | 42 | 43 | 40 | 42 | 0 | 0 | 0 | 0 | 0 | 0 | 55 | 58 | 80 | 58 | 99 | 0 | 0 | 0 | 0 | 0 | 100 | 100 | 100 | 78 | 0 |
| Rv0094c | - | 317 | 0 | 51 | 41 | 45 | 48 | 0 | 0 | 0 | 0 | 38 | 54 | 48 | 68 | 74 | 74 | 74 | 98 | 62 | 62 | 61 | 61 | 56 | 99 | 100 | 100 | 74 | 61 |
| Rv0095c | - | 136 | 0 | 0 | 0 | 0 | 0 | 0 | 0 | 0 | 0 | 0 | 0 | 0 | 0 | 64 | 70 | 60 | 95 | 0 | 0 | 0 | 0 | 0 | 100 | 100 | 97 | 70 | 0 |
| Rv0096 | *PPE1* | 463 | 0 | 0 | 0 | 0 | 0 | 0 | 0 | 0 | 0 | 37 | 37 | 0 | 65 | 54 | 74 | 54 | 99 | 50 | 54 | 54 | 54 | 53 | 100 | 100 | 100 | 74 | 51 |
| Rv0097 | - | 289 | 0 | 0 | 0 | 0 | 0 | 0 | 0 | 0 | 40 | 43 | 42 | 41 | 88 | 40 | 96 | 40 | 100 | 0 | 0 | 0 | 0 | 39 | 100 | 100 | 100 | 95 | 0 |
| Rv0098 | - | 183 | 0 | 0 | 0 | 0 | 0 | 0 | 0 | 0 | 0 | 0 | 0 | 0 | 81 | 0 | 0 | 0 | 100 | 0 | 0 | 0 | 0 | 0 | 100 | 100 | 100 | 85 | 0 |
| Rv0099 | *fadD10* | 540 | 41 | 40 | 40 | 46 | 41 | 40 | 41 | 42 | 44 | 43 | 43 | 46 | 75 | 44 | 88 | 44 | 100 | 44 | 42 | 42 | 42 | 43 | 100 | 100 | 100 | 88 | 44 |
| Rv0100 | - | 78 | 0 | 0 | 0 | 0 | 0 | 0 | 0 | 0 | 0 | 0 | 0 | 0 | 84 | 0 | 88 | 0 | 100 | 0 | 0 | 0 | 0 | 0 | 100 | 100 | 100 | 88 | 0 |
| Rv0101 | *nrp* | 2512 | 46 | 48 | 47 | 46 | 44 | 41 | 43 | 40 | 51 | 52 | 52 | 52 | 62 | 64 | 67 | 67 | 100 | 49 | 63 | 64 | 64 | 66 | 100 | 99 | 99 | 75 | 50 |
| Rv0102 | - | 661 | 45 | 45 | 47 | 44 | 49 | 49 | 47 | 53 | 53 | 50 | 52 | 52 | 50 | 50 | 85 | 50 | 100 | 51 | 54 | 54 | 54 | 52 | 100 | 100 | 100 | 83 | 52 |
| Rv0103c | *ctpB* | 752 | 60 | 60 | 63 | 62 | 61 | 63 | 58 | 64 | 65 | 64 | 65 | 64 | 60 | 60 | 89 | 63 | 99 | 64 | 64 | 65 | 65 | 64 | 99 | 100 | 99 | 89 | 66 |
| Rv0104 | - | 504 | 0 | 0 | 0 | 0 | 0 | 0 | 0 | 0 | 0 | 0 | 0 | 0 | 0 | 0 | 83 | 0 | 99 | 0 | 0 | 0 | 0 | 0 | 100 | 100 | 100 | 80 | 0 |
| Rv0105c | *rpmB* | 94 | 77 | 80 | 77 | 79 | 77 | 77 | 76 | 0 | 80 | 79 | 82 | 80 | 90 | 92 | 85 | 85 | 100 | 84 | 85 | 85 | 85 | 85 | 100 | 100 | 100 | 75 | 87 |
| Rv0106 | - | 398 | 44 | 44 | 0 | 42 | 40 | 44 | 0 | 0 | 59 | 61 | 63 | 63 | 79 | 77 | 82 | 79 | 100 | 69 | 73 | 73 | 73 | 74 | 100 | 100 | 100 | 82 | 73 |
| Rv0107c | *ctpI* | 1632 | 46 | 39 | 46 | 48 | 41 | 43 | 41 | 46 | 51 | 50 | 49 | 50 | 41 | 90 | 92 | 90 | 99 | 46 | 46 | 46 | 46 | 46 | 99 | 100 | 99 | 91 | 47 |
| Rv0108c | - | 69 | 0 | 0 | 0 | 0 | 0 | 0 | 0 | 0 | 0 | 0 | 0 | 0 | 0 | 88 | 92 | 88 | 100 | 0 | 0 | 0 | 0 | 0 | 100 | 100 | 100 | 91 | 0 |
| Rv0109 | *PE_PGRS1* | 496 | 41 | 49 | 36 | 36 | 32 | 34 | 36 | 38 | 39 | 38 | 43 | 42 | 46 | 46 | 67 | 38 | 99 | 52 | 52 | 55 | 55 | 48 | 99 | 99 | 99 | 56 | 53 |
| Rv0110 | - | 249 | 57 | 56 | 50 | 50 | 51 | 50 | 40 | 52 | 55 | 57 | 54 | 55 | 58 | 0 | 0 | 0 | 100 | 70 | 78 | 78 | 78 | 75 | 100 | 100 | 100 | 86 | 72 |
| Rv0111 | - | 685 | 51 | 50 | 42 | 46 | 47 | 47 | 49 | 0 | 41 | 39 | 40 | 49 | 43 | 90 | 89 | 90 | 100 | 71 | 73 | 73 | 73 | 71 | 99 | 100 | 100 | 89 | 73 |
| Rv0112 | *gca* | 318 | 42 | 42 | 44 | 42 | 41 | 43 | 41 | 44 | 43 | 43 | 42 | 41 | 40 | 43 | 48 | 40 | 100 | 42 | 44 | 41 | 41 | 40 | 100 | 100 | 100 | 40 | 39 |
| Rv0113 | *gmhA* | 196 | 0 | 0 | 0 | 0 | 0 | 0 | 0 | 0 | 61 | 0 | 51 | 50 | 0 | 65 | 53 | 65 | 100 | 0 | 0 | 0 | 0 | 52 | 100 | 100 | 100 | 0 | 0 |
| Rv0114 | *gmhB* | 190 | 0 | 0 | 0 | 0 | 0 | 0 | 0 | 0 | 54 | 0 | 0 | 0 | 0 | 51 | 0 | 51 | 99 | 0 | 0 | 0 | 0 | 0 | 100 | 100 | 100 | 0 | 0 |
| Rv0115 | *hddA* | 386 | 0 | 0 | 0 | 0 | 0 | 0 | 0 | 0 | 0 | 0 | 0 | 0 | 0 | 53 | 0 | 53 | 99 | 0 | 0 | 0 | 0 | 0 | 100 | 100 | 100 | 0 | 0 |
| Rv0116c | - | 251 | 55 | 56 | 51 | 53 | 58 | 57 | 54 | 53 | 65 | 59 | 65 | 66 | 67 | 82 | 90 | 83 | 100 | 68 | 68 | 68 | 68 | 73 | 100 | 100 | 100 | 90 | 63 |
| Rv0117 | *oxyS* | 314 | 45 | 45 | 44 | 47 | 41 | 44 | 47 | 41 | 46 | 45 | 45 | 45 | 67 | 84 | 89 | 84 | 100 | 56 | 46 | 47 | 47 | 75 | 100 | 100 | 100 | 88 | 56 |
| Rv0118c | *oxcA* | 582 | 46 | 44 | 46 | 47 | 45 | 46 | 45 | 47 | 45 | 45 | 45 | 47 | 46 | 85 | 87 | 81 | 99 | 45 | 47 | 46 | 46 | 79 | 100 | 100 | 100 | 87 | 45 |
| Rv0119 | *fadD7* | 525 | 42 | 42 | 43 | 43 | 44 | 42 | 46 | 45 | 51 | 50 | 51 | 50 | 44 | 78 | 84 | 78 | 100 | 47 | 44 | 44 | 44 | 61 | 100 | 100 | 100 | 83 | 46 |
| Rv0120c | *fusA2* | 714 | 49 | 50 | 50 | 51 | 51 | 50 | 51 | 76 | 70 | 51 | 54 | 54 | 51 | 92 | 93 | 92 | 100 | 51 | 85 | 85 | 85 | 85 | 100 | 100 | 100 | 90 | 52 |
| Rv0121c | - | 144 | 0 | 0 | 0 | 0 | 0 | 0 | 0 | 0 | 0 | 56 | 56 | 56 | 55 | 79 | 78 | 79 | 100 | 71 | 71 | 71 | 71 | 69 | 98 | 100 | 99 | 78 | 72 |
| Rv0122 | - | 122 | 0 | 0 | 0 | 0 | 0 | 0 | 0 | 0 | 0 | 0 | 0 | 0 | 0 | 0 | 0 | 0 | 100 | 0 | 0 | 0 | 0 | 0 | 100 | 100 | 100 | 0 | 0 |
| Rv0123 | - | 122 | 0 | 0 | 0 | 0 | 0 | 0 | 0 | 0 | 0 | 0 | 0 | 0 | 0 | 0 | 0 | 0 | 100 | 0 | 0 | 0 | 0 | 0 | 100 | 100 | 100 | 0 | 0 |
| Rv0124 | *PE_PGRS2* | 487 | 45 | 35 | 37 | 37 | 36 | 33 | 35 | 39 | 39 | 40 | 41 | 40 | 41 | 55 | 39 | 39 | 92 | 48 | 47 | 48 | 48 | 47 | 93 | 99 | 75 | 61 | 47 |
| Rv0125 | *pepA* | 355 | 49 | 50 | 50 | 49 | 50 | 49 | 47 | 49 | 58 | 56 | 60 | 59 | 54 | 81 | 91 | 82 | 100 | 61 | 61 | 61 | 61 | 62 | 100 | 100 | 100 | 90 | 62 |
| Rv0126 | *treS* | 601 | 68 | 44 | 79 | 79 | 0 | 0 | 0 | 86 | 44 | 45 | 85 | 85 | 43 | 95 | 95 | 95 | 100 | 56 | 93 | 93 | 93 | 92 | 100 | 100 | 100 | 95 | 43 |
| Rv0127 | - | 455 | 0 | 0 | 53 | 54 | 0 | 0 | 0 | 53 | 0 | 0 | 59 | 59 | 0 | 83 | 84 | 83 | 99 | 70 | 75 | 75 | 75 | 74 | 99 | 100 | 99 | 83 | 71 |
| Rv0128 | - | 259 | 0 | 0 | 0 | 0 | 52 | 0 | 47 | 0 | 0 | 51 | 50 | 48 | 67 | 82 | 82 | 82 | 100 | 0 | 0 | 0 | 0 | 0 | 100 | 100 | 100 | 84 | 0 |

| | | | | | | | | | | | | | | | | | | | | | | | | | | | | | |
|---|---|---|---|---|---|---|---|---|---|---|---|---|---|---|---|---|---|---|---|---|---|---|---|---|---|---|---|---|---|
| Rv0129c | *fbpC* | 340 | 49 | 49 | 53 | 53 | 56 | 48 | 54 | 0 | 54 | 55 | 55 | 55 | 72 | 90 | 92 | 90 | 100 | 82 | 83 | 83 | 83 | 83 | 100 | 100 | 100 | 92 | 83 |
| Rv0130 | - | 151 | 0 | 0 | 0 | 61 | 0 | 0 | 0 | 72 | 72 | 75 | 73 | 74 | 66 | 96 | 96 | 96 | 100 | 78 | 76 | 76 | 76 | 77 | 100 | 100 | 100 | 95 | 76 |
| Rv0131c | *fadE1* | 447 | 44 | 0 | 44 | 40 | 55 | 46 | 55 | 71 | 79 | 61 | 59 | 72 | 77 | 94 | 95 | 94 | 100 | 75 | 76 | 76 | 76 | 76 | 100 | 100 | 100 | 94 | 76 |
| Rv0132c | *fgd2* | 360 | 0 | 0 | 0 | 0 | 0 | 0 | 0 | 47 | 53 | 53 | 53 | 53 | 52 | 83 | 82 | 82 | 100 | 54 | 54 | 54 | 54 | 53 | 100 | 100 | 100 | 50 | 55 |
| Rv0133 | - | 201 | 0 | 0 | 44 | 47 | 0 | 0 | 0 | 0 | 47 | 65 | 64 | 63 | 48 | 82 | 82 | 82 | 99 | 68 | 75 | 69 | 75 | 66 | 100 | 100 | 100 | 81 | 67 |
| Rv0134 | *ephF* | 300 | 50 | 46 | 53 | 56 | 47 | 42 | 42 | 44 | 40 | 47 | 45 | 45 | 55 | 87 | 87 | 87 | 86 | 54 | 43 | 43 | 43 | 45 | 100 | 100 | 99 | 87 | 52 |
| Rv0135c | - | 201 | 0 | 0 | 0 | 0 | 0 | 0 | 0 | 0 | 63 | 0 | 0 | 44 | 0 | 83 | 83 | 83 | 100 | 72 | 79 | 79 | 79 | 75 | 100 | 100 | 100 | 85 | 74 |
| Rv0136 | *cyp138* | 441 | 0 | 0 | 0 | 0 | 0 | 0 | 0 | 47 | 67 | 43 | 43 | 44 | 75 | 86 | 86 | 86 | 100 | 79 | 78 | 77 | 77 | 79 | 100 | 100 | 100 | 78 | 81 |
| Rv0137c | *msrA* | 182 | 52 | 57 | 53 | 53 | 52 | 52 | 54 | 55 | 88 | 55 | 56 | 56 | 53 | 94 | 95 | 94 | 100 | 89 | 86 | 87 | 87 | 86 | 100 | 100 | 100 | 95 | 90 |
| Rv0138 | - | 167 | 0 | 0 | 0 | 0 | 0 | 0 | 0 | 0 | 68 | 63 | 67 | 65 | 61 | 85 | 92 | 86 | 99 | 83 | 85 | 85 | 85 | 86 | 100 | 100 | 100 | 91 | 83 |
| Rv0139 | - | 340 | 0 | 0 | 0 | 0 | 0 | 42 | 0 | 0 | 49 | 0 | 47 | 48 | 43 | 88 | 88 | 88 | 99 | 76 | 80 | 79 | 79 | 80 | 100 | 100 | 100 | 88 | 79 |
| Rv0140 | - | 126 | 0 | 0 | 0 | 0 | 0 | 0 | 0 | 67 | 0 | 56 | 50 | 54 | 68 | 86 | 84 | 84 | 100 | 76 | 77 | 77 | 77 | 77 | 100 | 100 | 100 | 85 | 76 |
| Rv0141c | - | 136 | 0 | 0 | 0 | 0 | 0 | 0 | 0 | 0 | 0 | 0 | 0 | 0 | 0 | 79 | 89 | 79 | 100 | 0 | 0 | 0 | 0 | 0 | 100 | 100 | 100 | 88 | 0 |
| Rv0142 | - | 308 | 0 | 0 | 0 | 0 | 0 | 0 | 0 | 60 | 67 | 70 | 72 | 72 | 58 | 88 | 88 | 88 | 100 | 74 | 78 | 78 | 78 | 77 | 100 | 100 | 100 | 84 | 76 |
| Rv0143c | - | 492 | 0 | 0 | 0 | 0 | 0 | 45 | 0 | 0 | 0 | 41 | 74 | 74 | 0 | 41 | 92 | 41 | 99 | 40 | 44 | 44 | 44 | 81 | 99 | 100 | 100 | 91 | 39 |
| Rv0144 | - | 280 | 0 | 0 | 0 | 0 | 0 | 0 | 0 | 0 | 0 | 0 | 0 | 0 | 0 | 75 | 80 | 68 | 100 | 65 | 71 | 71 | 71 | 74 | 100 | 100 | 100 | 80 | 68 |
| Rv0145 | - | 317 | 0 | 0 | 0 | 0 | 0 | 0 | 0 | 0 | 46 | 58 | 61 | 0 | 65 | 82 | 83 | 81 | 100 | 68 | 66 | 68 | 68 | 73 | 100 | 100 | 65 | 83 | 67 |
| Rv0146 | - | 310 | 0 | 0 | 0 | 0 | 0 | 0 | 0 | 0 | 50 | 57 | 57 | 0 | 66 | 86 | 88 | 86 | 100 | 74 | 77 | 77 | 77 | 74 | 100 | 100 | 100 | 88 | 74 |
| Rv0147 | - | 506 | 44 | 42 | 44 | 50 | 44 | 44 | 49 | 48 | 48 | 49 | 45 | 62 | 72 | 87 | 88 | 87 | 100 | 80 | 82 | 82 | 82 | 51 | 100 | 100 | 100 | 88 | 81 |
| Rv0148 | - | 286 | 49 | 0 | 50 | 59 | 48 | 51 | 48 | 59 | 58 | 65 | 56 | 59 | 88 | 92 | 96 | 92 | 100 | 89 | 88 | 89 | 89 | 89 | 100 | 100 | 100 | 96 | 89 |
| Rv0149 | - | 322 | 47 | 48 | 47 | 47 | 45 | 44 | 45 | 56 | 61 | 70 | 70 | 62 | 75 | 62 | 90 | 62 | 100 | 77 | 78 | 78 | 78 | 77 | 100 | 100 | 100 | 90 | 78 |
| Rv0150c | - | 95 | 0 | 0 | 0 | 0 | 0 | 0 | 0 | 0 | 0 | 0 | 0 | 0 | 0 | 0 | 0 | 0 | 100 | 0 | 0 | 0 | 0 | 0 | 100 | 100 | 100 | 0 | 0 |
| Rv0151c | *PE1* | 588 | 0 | 0 | 0 | 0 | 0 | 0 | 0 | 0 | 0 | 0 | 0 | 0 | 45 | 64 | 83 | 47 | 99 | 47 | 49 | 49 | 49 | 47 | 99 | 100 | 99 | 62 | 50 |
| Rv0152c | *PE2* | 525 | 0 | 0 | 0 | 0 | 0 | 0 | 0 | 0 | 0 | 0 | 0 | 0 | 48 | 0 | 80 | 0 | 100 | 46 | 49 | 49 | 49 | 45 | 100 | 100 | 99 | 66 | 52 |
| Rv0153c | *ptbB* | 276 | 0 | 0 | 0 | 0 | 0 | 0 | 0 | 46 | 50 | 44 | 45 | 43 | 55 | 67 | 87 | 86 | 99 | 71 | 69 | 69 | 69 | 72 | 100 | 100 | 100 | 86 | 71 |
| Rv0154c | *fadE2* | 403 | 44 | 0 | 45 | 43 | 69 | 47 | 69 | 72 | 73 | 73 | 73 | 73 | 62 | 91 | 92 | 92 | 100 | 88 | 89 | 89 | 89 | 87 | 100 | 100 | 100 | 95 | 87 |
| Rv0155 | *pntAa* | 366 | 44 | 0 | 0 | 0 | 0 | 0 | 58 | 58 | 88 | 86 | 88 | 88 | 92 | 94 | 94 | 94 | 100 | 60 | 60 | 60 | 60 | 82 | 99 | 100 | 100 | 94 | 60 |
| Rv0156 | *pntAb* | 110 | 0 | 0 | 0 | 0 | 0 | 0 | 74 | 66 | 91 | 85 | 90 | 92 | 87 | 96 | 90 | 96 | 99 | 67 | 65 | 65 | 65 | 89 | 100 | 100 | 100 | 90 | 60 |
| Rv0157 | *pntB* | 475 | 0 | 0 | 0 | 0 | 0 | 0 | 60 | 61 | 90 | 89 | 90 | 89 | 82 | 95 | 95 | 96 | 100 | 58 | 58 | 58 | 58 | 83 | 100 | 100 | 100 | 94 | 59 |
| Rv0158 | - | 214 | 0 | 0 | 0 | 0 | 0 | 0 | 0 | 49 | 46 | 46 | 51 | 50 | 80 | 93 | 93 | 93 | 100 | 88 | 85 | 85 | 85 | 86 | 100 | 100 | 100 | 94 | 88 |
| Rv0159c | *PE3* | 468 | 0 | 0 | 0 | 0 | 0 | 0 | 0 | 0 | 0 | 0 | 0 | 0 | 45 | 60 | 69 | 60 | 99 | 50 | 47 | 47 | 47 | 49 | 99 | 100 | 99 | 68 | 49 |
| Rv0160c | *PE4* | 502 | 0 | 0 | 0 | 0 | 0 | 0 | 0 | 0 | 0 | 41 | 0 | 0 | 48 | 64 | 65 | 65 | 99 | 44 | 49 | 44 | 44 | 51 | 100 | 100 | 100 | 82 | 44 |
| Rv0161 | - | 449 | 0 | 0 | 39 | 41 | 0 | 46 | 0 | 45 | 45 | 70 | 70 | 70 | 77 | 87 | 88 | 88 | 100 | 44 | 81 | 81 | 81 | 84 | 100 | 100 | 100 | 89 | 82 |
| Rv0162c | *adhE1* | 383 | 70 | 44 | 46 | 48 | 51 | 49 | 51 | 48 | 75 | 74 | 78 | 78 | 65 | 87 | 87 | 87 | 100 | 52 | 82 | 83 | 83 | 81 | 100 | 100 | 100 | 87 | 49 |
| Rv0163 | - | 151 | 0 | 0 | 0 | 0 | 0 | 0 | 0 | 66 | 70 | 69 | 75 | 70 | 67 | 85 | 81 | 85 | 100 | 59 | 83 | 83 | 83 | 83 | 100 | 100 | 100 | 82 | 60 |
| Rv0164 | *TB18.5* | 161 | 0 | 0 | 0 | 0 | 0 | 0 | 0 | 47 | 0 | 50 | 52 | 52 | 64 | 88 | 90 | 88 | 100 | 80 | 80 | 80 | 80 | 75 | 100 | 100 | 100 | 89 | 80 |
| Rv0165c | - | 264 | 0 | 0 | 0 | 46 | 0 | 0 | 0 | 45 | 42 | 71 | 69 | 69 | 67 | 85 | 80 | 85 | 99 | 73 | 80 | 80 | 80 | 64 | 100 | 100 | 100 | 79 | 79 |
| Rv0166 | *fadD5* | 554 | 47 | 45 | 47 | 52 | 46 | 47 | 49 | 50 | 71 | 75 | 74 | 73 | 84 | 91 | 89 | 90 | 99 | 83 | 83 | 85 | 85 | 84 | 100 | 100 | 100 | 93 | 85 |

| | | | | | | | | | | | | | | | | | | | | | | | | | | | | | |
|---|---|---|---|---|---|---|---|---|---|---|---|---|---|---|---|---|---|---|---|---|---|---|---|---|---|---|---|---|---|
| Rv0167 | *yrbE1A* | 265 | 0 | 0 | 0 | 0 | 0 | 0 | 0 | 67 | 78 | 80 | 79 | 85 | 79 | 98 | 94 | 98 | 100 | 91 | 91 | 91 | 91 | 91 | 100 | 100 | 100 | 94 | 91 |
| Rv0168 | *yrbE1B* | 289 | 0 | 0 | 0 | 0 | 0 | 0 | 0 | 68 | 74 | 71 | 73 | 78 | 75 | 96 | 93 | 93 | 100 | 89 | 87 | 87 | 87 | 83 | 100 | 100 | 100 | 93 | 89 |
| Rv0169 | *mce1A* | 454 | 0 | 0 | 0 | 0 | 0 | 0 | 0 | 51 | 45 | 46 | 48 | 49 | 48 | 81 | 81 | 81 | 99 | 72 | 72 | 72 | 72 | 69 | 100 | 100 | 100 | 81 | 72 |
| Rv0170 | *mce1B* | 346 | 0 | 0 | 0 | 0 | 0 | 0 | 0 | 55 | 55 | 60 | 57 | 62 | 58 | 89 | 89 | 89 | 99 | 80 | 82 | 82 | 82 | 81 | 100 | 100 | 100 | 87 | 81 |
| Rv0171 | *mce1C* | 515 | 0 | 0 | 40 | 38 | 38 | 0 | 34 | 52 | 59 | 54 | 56 | 58 | 53 | 87 | 89 | 88 | 100 | 76 | 76 | 77 | 77 | 77 | 99 | 100 | 100 | 88 | 77 |
| Rv0172 | *mce1D* | 530 | 0 | 0 | 0 | 0 | 0 | 0 | 0 | 52 | 53 | 51 | 55 | 59 | 45 | 88 | 89 | 88 | 100 | 76 | 76 | 76 | 76 | 76 | 100 | 100 | 99 | 89 | 76 |
| Rv0173 | *lprK* | 390 | 0 | 0 | 0 | 0 | 0 | 0 | 0 | 53 | 57 | 61 | 60 | 60 | 55 | 90 | 87 | 90 | 100 | 79 | 82 | 82 | 82 | 81 | 100 | 100 | 100 | 86 | 80 |
| Rv0174 | *mce1F* | 515 | 0 | 0 | 0 | 0 | 0 | 0 | 0 | 52 | 56 | 56 | 48 | 55 | 52 | 87 | 86 | 87 | 99 | 78 | 82 | 82 | 82 | 79 | 99 | 100 | 99 | 86 | 77 |
| Rv0175 | - | 213 | 0 | 0 | 0 | 0 | 0 | 0 | 0 | 0 | 0 | 0 | 50 | 46 | 0 | 72 | 74 | 74 | 100 | 67 | 72 | 67 | 67 | 70 | 93 | 100 | 99 | 75 | 67 |
| Rv0176 | - | 322 | 0 | 0 | 0 | 0 | 0 | 0 | 0 | 50 | 0 | 0 | 0 | 0 | 43 | 69 | 71 | 70 | 99 | 64 | 62 | 62 | 62 | 64 | 100 | 100 | 100 | 70 | 65 |
| Rv0177 | - | 184 | 0 | 0 | 0 | 0 | 0 | 0 | 0 | 0 | 0 | 0 | 0 | 47 | 0 | 76 | 79 | 76 | 100 | 66 | 70 | 70 | 70 | 69 | 100 | 100 | 100 | 79 | 70 |
| Rv0178 | - | 244 | 0 | 0 | 0 | 0 | 0 | 0 | 0 | 0 | 42 | 0 | 0 | 41 | 0 | 70 | 69 | 72 | 100 | 62 | 67 | 68 | 68 | 66 | 100 | 100 | 100 | 71 | 63 |
| Rv0179c | *lprO* | 369 | 0 | 0 | 0 | 0 | 0 | 0 | 0 | 0 | 0 | 0 | 0 | 0 | 71 | 90 | 95 | 90 | 99 | 83 | 83 | 83 | 83 | 82 | 100 | 100 | 100 | 94 | 82 |
| Rv0180c | - | 452 | 0 | 0 | 0 | 0 | 0 | 0 | 0 | 0 | 60 | 66 | 66 | 66 | 0 | 84 | 89 | 84 | 100 | 0 | 0 | 0 | 0 | 0 | 100 | 100 | 100 | 88 | 0 |
| Rv0181c | - | 244 | 0 | 0 | 44 | 53 | 0 | 0 | 0 | 60 | 71 | 76 | 76 | 75 | 74 | 80 | 84 | 80 | 100 | 77 | 75 | 75 | 75 | 76 | 100 | 100 | 99 | 81 | 77 |
| Rv0182c | *sigG* | 370 | 43 | 46 | 42 | 43 | 42 | 44 | 46 | 68 | 54 | 76 | 80 | 81 | 78 | 87 | 83 | 87 | 100 | 77 | 77 | 77 | 77 | 78 | 100 | 100 | 100 | 83 | 75 |
| Rv0183 | - | 279 | 0 | 46 | 0 | 0 | 0 | 38 | 0 | 0 | 56 | 66 | 67 | 65 | 75 | 91 | 93 | 91 | 100 | 78 | 78 | 78 | 78 | 78 | 100 | 100 | 100 | 92 | 81 |
| Rv0184 | - | 249 | 0 | 0 | 0 | 0 | 0 | 0 | 0 | 0 | 67 | 68 | 71 | 71 | 83 | 95 | 95 | 95 | 100 | 85 | 87 | 87 | 87 | 89 | 100 | 100 | 100 | 89 | 87 |
| Rv0185 | - | 169 | 0 | 0 | 0 | 0 | 0 | 0 | 0 | 0 | 59 | 57 | 59 | 60 | 70 | 85 | 85 | 85 | 100 | 74 | 74 | 74 | 74 | 70 | 100 | 100 | 100 | 86 | 76 |
| Rv0186 | *bglS* | 691 | 0 | 0 | 0 | 0 | 0 | 0 | 0 | 40 | 50 | 50 | 50 | 0 | 0 | 84 | 87 | 85 | 99 | 0 | 0 | 0 | 0 | 49 | 100 | 100 | 100 | 86 | 0 |
| Rv0187 | - | 220 | 0 | 0 | 0 | 47 | 0 | 0 | 0 | 49 | 72 | 74 | 55 | 55 | 69 | 81 | 50 | 81 | 100 | 70 | 73 | 73 | 73 | 74 | 100 | 100 | 100 | 49 | 69 |
| Rv0188 | - | 143 | 0 | 0 | 0 | 0 | 0 | 0 | 0 | 0 | 0 | 0 | 72 | 72 | 0 | 83 | 76 | 76 | 100 | 0 | 0 | 0 | 0 | 0 | 100 | 100 | 100 | 0 | 0 |
| Rv0189c | *ilvD* | 575 | 53 | 53 | 86 | 54 | 54 | 54 | 53 | 86 | 91 | 91 | 92 | 90 | 92 | 94 | 95 | 94 | 100 | 90 | 91 | 92 | 92 | 91 | 100 | 99 | 100 | 95 | 93 |
| Rv0190 | - | 96 | 72 | 0 | 71 | 73 | 76 | 77 | 73 | 77 | 81 | 78 | 78 | 78 | 86 | 91 | 95 | 91 | 100 | 90 | 91 | 91 | 91 | 90 | 100 | 100 | 100 | 95 | 89 |
| Rv0191 | - | 413 | 42 | 42 | 40 | 52 | 44 | 44 | 44 | 42 | 46 | 56 | 45 | 45 | 42 | 85 | 88 | 85 | 100 | 43 | 73 | 73 | 73 | 73 | 99 | 100 | 100 | 88 | 43 |
| Rv0192 | - | 366 | 54 | 56 | 58 | 58 | 58 | 58 | 53 | 53 | 67 | 58 | 67 | 67 | 67 | 78 | 78 | 78 | 96 | 76 | 78 | 75 | 75 | 74 | 99 | 100 | 99 | 90 | 77 |
| Rv0192A | - | 100 | 0 | 0 | 0 | 0 | 0 | 0 | 0 | 0 | 0 | 0 | 0 | 0 | 0 | 64 | 0 | 0 | 93 | 0 | 0 | 0 | 0 | 0 | 100 | 100 | 100 | 81 | 0 |
| Rv0193c | - | 615 | 0 | 0 | 0 | 0 | 0 | 0 | 0 | 0 | 0 | 0 | 0 | 0 | 0 | 0 | 0 | 0 | 99 | 0 | 0 | 0 | 0 | 0 | 100 | 100 | 100 | 86 | 0 |
| Rv0194 | - | 1194 | 53 | 56 | 55 | 56 | 50 | 51 | 51 | 55 | 57 | 57 | 60 | 58 | 51 | 50 | 86 | 50 | 99 | 57 | 47 | 47 | 47 | 56 | 99 | 100 | 99 | 85 | 57 |
| Rv0195 | - | 211 | 0 | 0 | 0 | 0 | 0 | 0 | 0 | 46 | 47 | 45 | 46 | 46 | 48 | 50 | 51 | 50 | 100 | 50 | 54 | 50 | 50 | 51 | 100 | 100 | 100 | 51 | 46 |
| Rv0196 | - | 194 | 0 | 0 | 0 | 0 | 0 | 0 | 0 | 0 | 51 | 52 | 50 | 48 | 78 | 85 | 85 | 85 | 100 | 73 | 75 | 75 | 75 | 73 | 100 | 100 | 100 | 88 | 73 |
| Rv0197 | - | 762 | 0 | 0 | 42 | 42 | 0 | 0 | 38 | 40 | 42 | 42 | 44 | 44 | 85 | 91 | 90 | 91 | 99 | 85 | 85 | 86 | 86 | 85 | 99 | 99 | 99 | 86 | 85 |
| Rv0198c | - | 663 | 61 | 59 | 61 | 60 | 59 | 55 | 61 | 67 | 75 | 79 | 80 | 81 | 82 | 0 | 89 | 88 | 100 | 84 | 85 | 86 | 86 | 85 | 100 | 100 | 100 | 89 | 82 |
| Rv0199 | - | 219 | 0 | 0 | 0 | 0 | 0 | 0 | 0 | 0 | 0 | 0 | 0 | 0 | 56 | 0 | 84 | 84 | 100 | 68 | 72 | 72 | 72 | 75 | 100 | 100 | 100 | 84 | 69 |
| Rv0200 | - | 229 | 0 | 0 | 0 | 0 | 0 | 0 | 0 | 0 | 0 | 0 | 0 | 0 | 62 | 0 | 85 | 85 | 100 | 71 | 75 | 75 | 75 | 74 | 100 | 100 | 100 | 85 | 70 |
| Rv0201c | - | 167 | 0 | 0 | 0 | 0 | 0 | 0 | 0 | 0 | 0 | 0 | 0 | 0 | 67 | 63 | 78 | 78 | 100 | 74 | 71 | 71 | 71 | 75 | 100 | 100 | 100 | 82 | 73 |
| Rv0202c | *mmpL11* | 966 | 47 | 50 | 42 | 46 | 49 | 49 | 48 | 50 | 45 | 49 | 48 | 49 | 67 | 84 | 85 | 84 | 100 | 80 | 79 | 78 | 78 | 80 | 100 | 100 | 100 | 85 | 80 |
| Rv0203 | - | 136 | 0 | 0 | 0 | 0 | 0 | 0 | 0 | 0 | 0 | 0 | 0 | 0 | 50 | 82 | 78 | 82 | 100 | 66 | 68 | 68 | 68 | 72 | 100 | 100 | 100 | 78 | 71 |

| | | | | | | | | | | | | | | | | | | | | | | | | | | | | | |
|---|---|---|---|---|---|---|---|---|---|---|---|---|---|---|---|---|---|---|---|---|---|---|---|---|---|---|---|---|---|
| Rv0204c | - | 412 | 49 | 48 | 47 | 48 | 52 | 58 | 54 | 0 | 61 | 58 | 64 | 67 | 77 | 86 | 87 | 85 | 100 | 77 | 81 | 81 | 81 | 80 | 100 | 100 | 100 | 87 | 74 |
| Rv0205 | - | 367 | 51 | 54 | 52 | 55 | 54 | 55 | 57 | 60 | 72 | 71 | 73 | 73 | 74 | 87 | 87 | 87 | 99 | 80 | 83 | 83 | 83 | 83 | 100 | 100 | 100 | 90 | 81 |
| Rv0206c | *mmpL3* | 944 | 57 | 61 | 57 | 62 | 57 | 43 | 59 | 43 | 58 | 63 | 63 | 60 | 68 | 78 | 84 | 78 | 99 | 65 | 70 | 66 | 66 | 72 | 99 | 100 | 100 | 84 | 72 |
| Rv0207c | - | 242 | 75 | 81 | 82 | 80 | 0 | 77 | 77 | 0 | 85 | 80 | 87 | 87 | 85 | 87 | 90 | 86 | 100 | 82 | 91 | 91 | 91 | 85 | 100 | 100 | 100 | 89 | 88 |
| Rv0208c | *trmB* | 263 | 61 | 59 | 61 | 61 | 61 | 60 | 59 | 52 | 69 | 74 | 72 | 72 | 70 | 73 | 74 | 73 | 100 | 70 | 71 | 69 | 69 | 77 | 100 | 100 | 100 | 73 | 70 |
| Rv0209 | - | 361 | 0 | 0 | 0 | 0 | 0 | 0 | 0 | 0 | 41 | 0 | 44 | 38 | 50 | 69 | 64 | 69 | 99 | 53 | 54 | 55 | 55 | 48 | 100 | 100 | 100 | 64 | 56 |
| Rv0210 | - | 492 | 0 | 0 | 0 | 0 | 0 | 0 | 0 | 0 | 39 | 43 | 42 | 44 | 60 | 74 | 74 | 74 | 99 | 57 | 67 | 67 | 67 | 68 | 99 | 100 | 100 | 74 | 57 |
| Rv0211 | *pckA* | 606 | 77 | 78 | 79 | 77 | 80 | 80 | 80 | 77 | 89 | 90 | 88 | 88 | 93 | 93 | 95 | 94 | 100 | 92 | 92 | 92 | 92 | 92 | 100 | 100 | 100 | 95 | 92 |
| Rv0212c | *nadR* | 323 | 0 | 0 | 0 | 0 | 0 | 0 | 0 | 0 | 0 | 0 | 0 | 45 | 0 | 0 | 78 | 0 | 100 | 0 | 0 | 0 | 0 | 0 | 99 | 100 | 100 | 0 | 0 |
| Rv0213c | - | 437 | 0 | 0 | 0 | 0 | 0 | 0 | 0 | 0 | 0 | 0 | 0 | 0 | 0 | 0 | 88 | 0 | 100 | 0 | 0 | 0 | 0 | 0 | 100 | 100 | 100 | 90 | 0 |
| Rv0214 | *fadD4* | 537 | 43 | 41 | 41 | 47 | 45 | 41 | 43 | 50 | 65 | 63 | 63 | 63 | 64 | 89 | 88 | 88 | 99 | 83 | 85 | 84 | 84 | 85 | 100 | 100 | 100 | 90 | 84 |
| Rv0215c | *fadE3* | 357 | 44 | 0 | 42 | 44 | 54 | 52 | 54 | 68 | 54 | 90 | 90 | 91 | 56 | 93 | 93 | 93 | 100 | 90 | 91 | 91 | 91 | 82 | 100 | 100 | 100 | 93 | 89 |
| Rv0216 | - | 337 | 0 | 0 | 0 | 0 | 0 | 0 | 0 | 0 | 0 | 75 | 75 | 77 | 0 | 86 | 85 | 86 | 100 | 76 | 79 | 78 | 78 | 80 | 100 | 100 | 100 | 85 | 78 |
| Rv0217c | *lipW* | 302 | 56 | 0 | 0 | 47 | 45 | 0 | 0 | 50 | 51 | 53 | 50 | 52 | 69 | 82 | 63 | 82 | 99 | 77 | 76 | 77 | 77 | 76 | 100 | 100 | 100 | 61 | 74 |
| Rv0218 | - | 442 | 0 | 0 | 0 | 0 | 0 | 0 | 0 | 0 | 0 | 0 | 78 | 78 | 0 | 48 | 80 | 80 | 99 | 0 | 82 | 81 | 81 | 52 | 99 | 100 | 99 | 84 | 52 |
| Rv0219 | - | 182 | 0 | 0 | 0 | 0 | 0 | 0 | 0 | 0 | 0 | 0 | 73 | 69 | 0 | 0 | 73 | 73 | 100 | 0 | 70 | 72 | 72 | 0 | 100 | 100 | 100 | 75 | 0 |
| Rv0220 | *lipC* | 403 | 0 | 0 | 0 | 41 | 0 | 0 | 0 | 64 | 41 | 45 | 61 | 61 | 42 | 86 | 89 | 86 | 100 | 79 | 80 | 80 | 80 | 80 | 100 | 100 | 100 | 89 | 80 |
| Rv0221 | - | 469 | 0 | 0 | 0 | 0 | 0 | 0 | 0 | 48 | 48 | 47 | 48 | 49 | 51 | 88 | 91 | 88 | 98 | 83 | 84 | 84 | 84 | 84 | 100 | 100 | 100 | 90 | 82 |
| Rv0222 | *echA1* | 262 | 50 | 44 | 51 | 40 | 52 | 53 | 50 | 61 | 73 | 80 | 74 | 76 | 79 | 91 | 94 | 92 | 58 | 80 | 81 | 81 | 81 | 78 | 100 | 100 | 100 | 94 | 82 |
| Rv0223c | - | 487 | 53 | 52 | 56 | 56 | 53 | 52 | 52 | 68 | 77 | 80 | 81 | 80 | 83 | 92 | 92 | 92 | 96 | 83 | 83 | 83 | 83 | 81 | 100 | 100 | 100 | 91 | 82 |
| Rv0224c | - | 254 | 70 | 69 | 68 | 65 | 64 | 0 | 67 | 61 | 74 | 68 | 69 | 69 | 77 | 87 | 90 | 87 | 99 | 81 | 81 | 81 | 81 | 88 | 100 | 100 | 100 | 89 | 80 |
| Rv0225 | - | 384 | 61 | 64 | 68 | 68 | 63 | 42 | 63 | 57 | 75 | 74 | 73 | 73 | 77 | 90 | 90 | 91 | 100 | 86 | 86 | 85 | 85 | 85 | 100 | 100 | 100 | 90 | 87 |
| Rv0226c | - | 576 | 47 | 43 | 45 | 43 | 44 | 0 | 46 | 46 | 58 | 63 | 64 | 64 | 69 | 83 | 81 | 83 | 100 | 69 | 73 | 73 | 73 | 69 | 100 | 100 | 100 | 80 | 72 |
| Rv0227c | - | 421 | 43 | 41 | 38 | 41 | 42 | 39 | 43 | 39 | 55 | 55 | 56 | 57 | 73 | 85 | 85 | 85 | 99 | 80 | 82 | 81 | 81 | 79 | 100 | 100 | 100 | 84 | 79 |
| Rv0228 | - | 407 | 56 | 52 | 52 | 53 | 55 | 0 | 56 | 48 | 66 | 67 | 69 | 69 | 77 | 86 | 86 | 86 | 100 | 80 | 78 | 78 | 78 | 83 | 100 | 100 | 100 | 86 | 80 |
| Rv0229c | - | 226 | 0 | 0 | 0 | 0 | 0 | 0 | 0 | 0 | 0 | 0 | 0 | 0 | 0 | 0 | 0 | 0 | 100 | 50 | 0 | 0 | 0 | 0 | 100 | 100 | 100 | 0 | 0 |
| Rv0230c | *php* | 326 | 0 | 0 | 0 | 0 | 0 | 0 | 0 | 0 | 0 | 73 | 72 | 59 | 0 | 94 | 94 | 95 | 100 | 0 | 88 | 88 | 88 | 87 | 100 | 100 | 100 | 93 | 0 |
| Rv0231 | *fadE4* | 568 | 0 | 0 | 0 | 0 | 0 | 0 | 0 | 0 | 67 | 71 | 69 | 69 | 69 | 89 | 91 | 89 | 100 | 70 | 0 | 0 | 0 | 0 | 100 | 100 | 100 | 91 | 69 |
| Rv0232 | - | 229 | 0 | 0 | 0 | 0 | 0 | 0 | 0 | 0 | 0 | 0 | 0 | 0 | 0 | 92 | 97 | 92 | 100 | 82 | 86 | 86 | 86 | 41 | 100 | 100 | 100 | 97 | 87 |
| Rv0233 | *nrdB* | 314 | 0 | 0 | 0 | 0 | 0 | 0 | 0 | 0 | 0 | 74 | 76 | 76 | 90 | 92 | 0 | 91 | 99 | 90 | 89 | 89 | 89 | 89 | 99 | 100 | 99 | 0 | 91 |
| Rv0234c | *gabD1* | 511 | 56 | 58 | 52 | 54 | 54 | 55 | 56 | 60 | 53 | 61 | 60 | 60 | 90 | 89 | 93 | 89 | 100 | 52 | 52 | 52 | 52 | 61 | 100 | 100 | 100 | 93 | 57 |
| Rv0235c | - | 482 | 0 | 0 | 0 | 0 | 0 | 0 | 0 | 0 | 0 | 0 | 75 | 75 | 0 | 86 | 86 | 86 | 100 | 0 | 0 | 0 | 0 | 81 | 100 | 100 | 100 | 86 | 79 |
| Rv0236A | - | 57 | 0 | 0 | 0 | 0 | 0 | 0 | 0 | 0 | 0 | 0 | 0 | 0 | 85 | 96 | 98 | 96 | 100 | 91 | 91 | 91 | 91 | 91 | 100 | 100 | 100 | 96 | 91 |
| Rv0236c | - | 1400 | 50 | 48 | 51 | 48 | 49 | 0 | 51 | 47 | 64 | 65 | 65 | 64 | 74 | 86 | 86 | 86 | 99 | 78 | 80 | 80 | 80 | 80 | 99 | 100 | 99 | 86 | 79 |
| Rv0237 | *lpqI* | 388 | 54 | 57 | 56 | 58 | 59 | 0 | 57 | 50 | 64 | 68 | 66 | 67 | 69 | 78 | 85 | 79 | 100 | 75 | 74 | 74 | 74 | 76 | 100 | 100 | 100 | 84 | 74 |
| Rv0238 | - | 204 | 48 | 47 | 44 | 45 | 0 | 0 | 0 | 53 | 71 | 75 | 74 | 74 | 79 | 90 | 90 | 90 | 100 | 89 | 88 | 88 | 88 | 87 | 100 | 100 | 100 | 89 | 91 |
| Rv0239 | - | 77 | 0 | 0 | 0 | 0 | 0 | 0 | 0 | 0 | 0 | 0 | 0 | 0 | 0 | 0 | 0 | 0 | 100 | 0 | 0 | 0 | 0 | 0 | 100 | 100 | 100 | 0 | 0 |
| Rv0240 | - | 145 | 0 | 0 | 0 | 0 | 0 | 0 | 0 | 0 | 0 | 0 | 0 | 0 | 0 | 0 | 0 | 0 | 100 | 0 | 0 | 0 | 0 | 0 | 100 | 100 | 100 | 0 | 0 |

| | | | | | | | | | | | | | | | | | | | | | | | | | | | | | |
|---|---|---|---|---|---|---|---|---|---|---|---|---|---|---|---|---|---|---|---|---|---|---|---|---|---|---|---|---|---|
| Rv0241c | - | 280 | 58 | 0 | 0 | 0 | 57 | 56 | 59 | 54 | 69 | 68 | 69 | 69 | 73 | 89 | 92 | 89 | 100 | 86 | 87 | 87 | 87 | 87 | 100 | 100 | 100 | 91 | 87 |
| Rv0242c | *fabG* | 454 | 75 | 51 | 54 | 46 | 76 | 72 | 77 | 68 | 79 | 79 | 79 | 79 | 87 | 94 | 92 | 94 | 100 | 88 | 88 | 87 | 87 | 89 | 100 | 100 | 100 | 92 | 89 |
| Rv0243 | *fadA2* | 440 | 76 | 0 | 48 | 46 | 76 | 78 | 78 | 78 | 81 | 82 | 83 | 83 | 86 | 93 | 93 | 93 | 100 | 90 | 92 | 92 | 92 | 91 | 100 | 100 | 100 | 96 | 90 |
| Rv0244c | *fadE5* | 611 | 0 | 0 | 0 | 0 | 44 | 41 | 44 | 77 | 83 | 85 | 85 | 85 | 89 | 96 | 96 | 96 | 99 | 90 | 92 | 92 | 92 | 90 | 100 | 100 | 99 | 95 | 92 |
| Rv0245 | - | 162 | 0 | 0 | 49 | 50 | 0 | 0 | 0 | 52 | 55 | 51 | 81 | 83 | 81 | 88 | 91 | 88 | 100 | 89 | 86 | 85 | 85 | 89 | 100 | 100 | 100 | 91 | 86 |
| Rv0246 | - | 436 | 0 | 0 | 0 | 0 | 0 | 0 | 0 | 0 | 0 | 0 | 0 | 0 | 0 | 0 | 79 | 0 | 99 | 0 | 0 | 0 | 0 | 0 | 100 | 100 | 100 | 80 | 0 |
| Rv0247c | - | 248 | 0 | 0 | 43 | 42 | 0 | 42 | 41 | 84 | 90 | 43 | 87 | 87 | 87 | 95 | 95 | 95 | 100 | 93 | 90 | 90 | 90 | 92 | 100 | 100 | 100 | 96 | 91 |
| Rv0248c | *sdhA* | 646 | 43 | 44 | 44 | 43 | 43 | 44 | 43 | 76 | 86 | 51 | 86 | 87 | 87 | 91 | 93 | 92 | 100 | 86 | 88 | 88 | 88 | 86 | 100 | 100 | 100 | 93 | 85 |
| Rv0249c | - | 273 | 0 | 0 | 0 | 0 | 0 | 0 | 0 | 67 | 82 | 0 | 79 | 80 | 85 | 80 | 94 | 80 | 100 | 78 | 83 | 83 | 83 | 86 | 100 | 100 | 100 | 94 | 81 |
| Rv0250c | - | 97 | 0 | 0 | 0 | 0 | 0 | 0 | 0 | 0 | 0 | 0 | 55 | 57 | 70 | 89 | 90 | 89 | 100 | 77 | 76 | 76 | 76 | 72 | 100 | 100 | 100 | 92 | 80 |
| Rv0251c | *hsp* | 159 | 0 | 0 | 0 | 0 | 0 | 0 | 0 | 0 | 0 | 0 | 0 | 0 | 69 | 78 | 84 | 78 | 100 | 71 | 76 | 76 | 76 | 72 | 100 | 100 | 100 | 84 | 76 |
| Rv0252 | *nirB* | 853 | 50 | 44 | 50 | 51 | 45 | 44 | 45 | 71 | 80 | 78 | 78 | 78 | 80 | 92 | 93 | 92 | 100 | 86 | 87 | 87 | 87 | 85 | 99 | 100 | 100 | 93 | 86 |
| Rv0253 | *nirD* | 118 | 0 | 0 | 0 | 0 | 0 | 0 | 0 | 62 | 72 | 75 | 72 | 73 | 67 | 84 | 90 | 90 | 100 | 81 | 86 | 86 | 86 | 83 | 100 | 100 | 100 | 95 | 88 |
| Rv0254c | *cobU* | 174 | 0 | 56 | 59 | 63 | 0 | 53 | 0 | 68 | 61 | 64 | 56 | 58 | 59 | 58 | 60 | 57 | 100 | 77 | 59 | 59 | 59 | 59 | 100 | 100 | 100 | 61 | 80 |
| Rv0255c | *cobQ1* | 494 | 0 | 61 | 59 | 0 | 0 | 53 | 0 | 78 | 61 | 62 | 62 | 62 | 61 | 62 | 89 | 62 | 100 | 87 | 63 | 63 | 63 | 61 | 100 | 100 | 100 | 89 | 85 |
| Rv0256c | *PPE2* | 556 | 0 | 0 | 0 | 0 | 0 | 0 | 0 | 0 | 45 | 46 | 46 | 44 | 47 | 67 | 71 | 71 | 100 | 49 | 50 | 50 | 50 | 52 | 99 | 100 | 100 | 67 | 51 |
| Rv0257 | - | 124 | 0 | 0 | 0 | 0 | 0 | 0 | 0 | 0 | 0 | 0 | 0 | 0 | 0 | 86 | 0 | 86 | 99 | 0 | 0 | 0 | 0 | 0 | 100 | 100 | 100 | 0 | 0 |
| Rv0258c | - | 151 | 0 | 0 | 0 | 0 | 0 | 0 | 0 | 0 | 0 | 0 | 0 | 0 | 0 | 94 | 96 | 94 | 100 | 0 | 0 | 0 | 0 | 53 | 100 | 100 | 100 | 96 | 49 |
| Rv0259c | - | 247 | 42 | 0 | 44 | 45 | 0 | 0 | 0 | 46 | 65 | 67 | 65 | 65 | 64 | 77 | 80 | 77 | 99 | 73 | 76 | 77 | 77 | 76 | 99 | 100 | 99 | 79 | 71 |
| Rv0260c | - | 381 | 0 | 0 | 0 | 0 | 0 | 0 | 0 | 60 | 70 | 70 | 69 | 69 | 70 | 86 | 90 | 86 | 100 | 84 | 83 | 83 | 83 | 85 | 100 | 100 | 100 | 89 | 84 |
| Rv0261c | *narK3* | 469 | 0 | 43 | 46 | 45 | 0 | 0 | 0 | 55 | 76 | 76 | 76 | 76 | 75 | 80 | 80 | 80 | 100 | 72 | 89 | 89 | 89 | 88 | 100 | 100 | 100 | 80 | 53 |
| Rv0262c | *aac* | 181 | 0 | 0 | 0 | 0 | 0 | 0 | 0 | 64 | 0 | 0 | 0 | 0 | 72 | 0 | 91 | 0 | 100 | 73 | 79 | 78 | 78 | 74 | 100 | 100 | 100 | 91 | 74 |
| Rv0263c | - | 300 | 53 | 0 | 52 | 52 | 53 | 0 | 49 | 61 | 62 | 62 | 64 | 63 | 82 | 91 | 89 | 91 | 100 | 86 | 85 | 85 | 85 | 88 | 99 | 100 | 100 | 88 | 84 |
| Rv0264c | - | 210 | 56 | 0 | 62 | 59 | 58 | 0 | 54 | 57 | 55 | 59 | 60 | 58 | 62 | 78 | 80 | 78 | 100 | 70 | 73 | 73 | 73 | 74 | 100 | 100 | 99 | 80 | 72 |
| Rv0265c | - | 330 | 42 | 48 | 43 | 50 | 50 | 45 | 50 | 0 | 45 | 50 | 47 | 48 | 65 | 87 | 88 | 87 | 100 | 68 | 71 | 71 | 71 | 72 | 100 | 100 | 100 | 88 | 69 |
| Rv0266c | *oplA* | 1209 | 45 | 0 | 0 | 0 | 0 | 0 | 0 | 50 | 43 | 50 | 48 | 48 | 0 | 0 | 0 | 0 | 100 | 0 | 48 | 48 | 48 | 47 | 98 | 100 | 100 | 0 | 42 |
| Rv0267 | *narU* | 463 | 0 | 45 | 46 | 46 | 0 | 0 | 0 | 60 | 64 | 60 | 59 | 59 | 57 | 90 | 90 | 90 | 100 | 57 | 58 | 58 | 58 | 58 | 100 | 100 | 100 | 90 | 88 |
| Rv0268c | - | 169 | 0 | 0 | 0 | 0 | 0 | 0 | 0 | 0 | 0 | 0 | 0 | 0 | 0 | 0 | 0 | 0 | 100 | 0 | 0 | 0 | 0 | 0 | 100 | 100 | 100 | 0 | 0 |
| Rv0269c | - | 397 | 0 | 0 | 0 | 0 | 0 | 0 | 0 | 60 | 64 | 61 | 61 | 61 | 78 | 85 | 86 | 86 | 100 | 79 | 81 | 80 | 80 | 81 | 100 | 100 | 100 | 84 | 78 |
| Rv0270 | *fadD2* | 560 | 53 | 55 | 46 | 45 | 54 | 52 | 54 | 63 | 66 | 61 | 60 | 61 | 83 | 91 | 91 | 91 | 100 | 87 | 87 | 87 | 87 | 87 | 100 | 100 | 100 | 90 | 89 |
| Rv0271c | *fadE6* | 731 | 0 | 0 | 0 | 0 | 46 | 42 | 42 | 55 | 55 | 61 | 62 | 62 | 80 | 90 | 91 | 89 | 100 | 87 | 88 | 88 | 88 | 88 | 100 | 100 | 100 | 91 | 88 |
| Rv0272c | - | 377 | 0 | 0 | 0 | 0 | 0 | 0 | 0 | 0 | 52 | 0 | 45 | 43 | 80 | 91 | 92 | 92 | 99 | 88 | 54 | 55 | 55 | 90 | 100 | 100 | 100 | 88 | 89 |
| Rv0273c | - | 206 | 0 | 0 | 0 | 0 | 0 | 0 | 0 | 0 | 0 | 49 | 44 | 45 | 77 | 88 | 89 | 89 | 100 | 48 | 48 | 48 | 48 | 78 | 100 | 100 | 100 | 88 | 80 |
| Rv0274 | - | 193 | 0 | 0 | 0 | 0 | 0 | 0 | 0 | 46 | 0 | 49 | 46 | 48 | 86 | 94 | 94 | 94 | 100 | 91 | 0 | 0 | 0 | 87 | 100 | 100 | 100 | 93 | 91 |
| Rv0275c | - | 241 | 0 | 0 | 0 | 0 | 0 | 0 | 0 | 46 | 50 | 57 | 61 | 61 | 65 | 85 | 73 | 85 | 99 | 73 | 78 | 78 | 78 | 74 | 100 | 100 | 100 | 77 | 77 |
| Rv0276 | - | 306 | 0 | 0 | 0 | 0 | 0 | 0 | 0 | 0 | 61 | 65 | 71 | 70 | 69 | 84 | 88 | 85 | 100 | 78 | 83 | 83 | 83 | 78 | 100 | 100 | 100 | 87 | 81 |
| Rv0277c | - | 142 | 0 | 0 | 0 | 0 | 0 | 0 | 0 | 0 | 0 | 0 | 0 | 0 | 0 | 0 | 0 | 0 | 100 | 50 | 0 | 0 | 0 | 0 | 100 | 100 | 99 | 0 | 0 |
| Rv0278c | *PE_PGRS3* | 957 | 47 | 32 | 34 | 35 | 38 | 35 | 40 | 37 | 40 | 37 | 38 | 43 | 43 | 43 | 61 | 32 | 78 | 47 | 46 | 47 | 47 | 45 | 99 | 99 | 87 | 59 | 45 |

| | | | | | | | | | | | | | | | | | | | | | | | | | | | | |
|---|---|---|---|---|---|---|---|---|---|---|---|---|---|---|---|---|---|---|---|---|---|---|---|---|---|---|---|---|---|
| Rv0279c | *PE_PGRS4* | 837 | 44 | 32 | 33 | 35 | 38 | 38 | 40 | 35 | 40 | 38 | 42 | 41 | 37 | 33 | 35 | 35 | 97 | 48 | 44 | 45 | 45 | 42 | 97 | 98 | 99 | 63 | 48 |
| Rv0280 | *PPE3* | 536 | 0 | 0 | 0 | 0 | 0 | 0 | 0 | 0 | 0 | 46 | 48 | 46 | 55 | 73 | 81 | 72 | 99 | 55 | 56 | 56 | 56 | 56 | 99 | 100 | 99 | 81 | 56 |
| Rv0281 | - | 302 | 0 | 0 | 0 | 0 | 0 | 0 | 0 | 0 | 51 | 55 | 57 | 0 | 68 | 85 | 86 | 86 | 100 | 60 | 74 | 74 | 74 | 74 | 100 | 100 | 100 | 85 | 60 |
| Rv0282 | - | 631 | 0 | 0 | 0 | 0 | 0 | 0 | 0 | 0 | 57 | 0 | 0 | 0 | 79 | 91 | 92 | 90 | 99 | 81 | 81 | 81 | 81 | 83 | 100 | 100 | 100 | 91 | 83 |
| Rv0283 | - | 538 | 39 | 45 | 40 | 41 | 49 | 51 | 47 | 0 | 53 | 54 | 0 | 55 | 69 | 80 | 82 | 82 | 100 | 72 | 77 | 77 | 77 | 76 | 100 | 100 | 100 | 83 | 74 |
| Rv0284 | - | 1330 | 46 | 45 | 45 | 46 | 42 | 45 | 42 | 49 | 51 | 52 | 0 | 52 | 81 | 91 | 92 | 93 | 99 | 83 | 85 | 85 | 85 | 85 | 99 | 100 | 99 | 91 | 83 |
| Rv0285 | *PE5* | 102 | 0 | 0 | 0 | 0 | 0 | 0 | 0 | 0 | 0 | 0 | 0 | 0 | 82 | 92 | 93 | 92 | 100 | 85 | 82 | 82 | 82 | 87 | 100 | 100 | 100 | 93 | 82 |
| Rv0286 | *PPE4* | 513 | 0 | 0 | 0 | 0 | 0 | 0 | 0 | 0 | 38 | 37 | 45 | 43 | 60 | 67 | 78 | 67 | 100 | 57 | 62 | 62 | 62 | 61 | 100 | 100 | 100 | 78 | 59 |
| Rv0287 | *esxG* | 97 | 0 | 0 | 0 | 0 | 0 | 0 | 0 | 0 | 0 | 0 | 0 | 0 | 81 | 91 | 96 | 91 | 100 | 88 | 87 | 88 | 88 | 87 | 100 | 100 | 100 | 96 | 89 |
| Rv0288 | *esxH* | 96 | 0 | 0 | 0 | 0 | 0 | 0 | 0 | 0 | 0 | 0 | 0 | 0 | 81 | 89 | 89 | 89 | 100 | 88 | 86 | 86 | 86 | 85 | 100 | 100 | 100 | 92 | 85 |
| Rv0289 | - | 295 | 0 | 0 | 0 | 0 | 0 | 0 | 0 | 0 | 41 | 0 | 42 | 40 | 73 | 84 | 87 | 84 | 100 | 72 | 70 | 70 | 70 | 74 | 100 | 100 | 100 | 86 | 74 |
| Rv0290 | - | 472 | 0 | 0 | 0 | 0 | 0 | 0 | 0 | 0 | 45 | 44 | 0 | 46 | 71 | 88 | 88 | 87 | 99 | 71 | 72 | 72 | 72 | 73 | 100 | 100 | 100 | 88 | 72 |
| Rv0291 | *mycP3* | 461 | 47 | 47 | 44 | 45 | 0 | 41 | 0 | 42 | 58 | 55 | 0 | 53 | 68 | 80 | 83 | 80 | 100 | 70 | 74 | 74 | 74 | 73 | 100 | 100 | 100 | 83 | 71 |
| Rv0292 | - | 331 | 0 | 0 | 0 | 0 | 0 | 0 | 0 | 0 | 0 | 0 | 0 | 0 | 53 | 73 | 71 | 73 | 100 | 60 | 61 | 61 | 61 | 57 | 100 | 100 | 100 | 71 | 59 |
| Rv0293c | - | 400 | 0 | 0 | 0 | 0 | 0 | 0 | 0 | 0 | 0 | 0 | 0 | 0 | 71 | 85 | 85 | 85 | 100 | 77 | 79 | 79 | 79 | 77 | 99 | 100 | 100 | 88 | 79 |
| Rv0294 | *tam* | 261 | 0 | 0 | 0 | 0 | 0 | 0 | 0 | 58 | 0 | 61 | 77 | 78 | 56 | 92 | 92 | 92 | 100 | 75 | 79 | 79 | 79 | 77 | 100 | 100 | 100 | 91 | 78 |
| Rv0295c | - | 267 | 0 | 0 | 0 | 0 | 0 | 0 | 0 | 0 | 0 | 69 | 70 | 69 | 86 | 86 | 85 | 86 | 100 | 0 | 87 | 87 | 87 | 84 | 100 | 100 | 100 | 85 | 0 |
| Rv0296c | - | 465 | 0 | 0 | 0 | 0 | 0 | 0 | 0 | 0 | 0 | 74 | 73 | 73 | 81 | 87 | 86 | 87 | 100 | 0 | 81 | 81 | 81 | 80 | 99 | 100 | 100 | 85 | 0 |
| Rv0297 | *PE_PGRS5* | 591 | 41 | 41 | 36 | 35 | 38 | 36 | 34 | 32 | 40 | 42 | 41 | 42 | 42 | 44 | 38 | 38 | 97 | 50 | 46 | 51 | 51 | 47 | 99 | 100 | 96 | 59 | 50 |
| Rv0298 | - | 75 | 0 | 0 | 0 | 0 | 0 | 0 | 0 | 0 | 0 | 0 | 0 | 0 | 0 | 0 | 0 | 0 | 100 | 0 | 0 | 0 | 0 | 0 | 100 | 100 | 100 | 0 | 0 |
| Rv0299 | - | 100 | 0 | 0 | 0 | 0 | 0 | 0 | 0 | 0 | 0 | 0 | 0 | 0 | 0 | 0 | 0 | 0 | 100 | 0 | 0 | 0 | 0 | 0 | 100 | 100 | 100 | 0 | 0 |
| Rv0300 | - | 73 | 0 | 0 | 0 | 0 | 0 | 0 | 0 | 0 | 0 | 0 | 0 | 0 | 0 | 0 | 0 | 0 | 100 | 0 | 0 | 0 | 0 | 0 | 100 | 100 | 100 | 0 | 0 |
| Rv0301 | - | 141 | 0 | 0 | 0 | 0 | 0 | 0 | 0 | 0 | 0 | 0 | 0 | 0 | 0 | 0 | 0 | 0 | 100 | 75 | 0 | 0 | 0 | 0 | 100 | 100 | 100 | 0 | 0 |
| Rv0302 | - | 210 | 0 | 0 | 0 | 0 | 0 | 0 | 0 | 51 | 46 | 0 | 0 | 0 | 0 | 52 | 49 | 0 | 99 | 45 | 47 | 47 | 47 | 0 | 100 | 100 | 100 | 0 | 49 |
| Rv0303 | - | 302 | 47 | 0 | 0 | 0 | 0 | 0 | 0 | 44 | 44 | 43 | 45 | 45 | 47 | 46 | 48 | 47 | 100 | 49 | 50 | 50 | 50 | 48 | 100 | 100 | 100 | 48 | 46 |
| Rv0304c | *PPE5* | 2204 | 0 | 0 | 0 | 0 | 0 | 0 | 0 | 0 | 35 | 38 | 35 | 34 | 37 | 0 | 66 | 0 | 99 | 35 | 34 | 33 | 33 | 34 | 100 | 100 | 100 | 58 | 35 |
| Rv0305c | *PPE6* | 963 | 0 | 0 | 0 | 0 | 0 | 0 | 32 | 0 | 42 | 38 | 0 | 0 | 49 | 65 | 67 | 65 | 100 | 46 | 45 | 45 | 45 | 51 | 100 | 100 | 98 | 59 | 49 |
| Rv0306 | - | 223 | 0 | 0 | 0 | 0 | 0 | 57 | 0 | 55 | 60 | 56 | 55 | 54 | 57 | 86 | 87 | 86 | 100 | 57 | 67 | 86 | 86 | 86 | 100 | 100 | 100 | 87 | 58 |
| Rv0307c | - | 160 | 0 | 0 | 0 | 0 | 0 | 0 | 0 | 0 | 0 | 0 | 0 | 0 | 0 | 80 | 80 | 80 | 100 | 78 | 70 | 70 | 70 | 71 | 100 | 100 | 100 | 83 | 78 |
| Rv0308 | - | 238 | 0 | 0 | 0 | 0 | 0 | 0 | 0 | 44 | 50 | 0 | 46 | 52 | 62 | 69 | 79 | 69 | 100 | 60 | 66 | 66 | 66 | 63 | 100 | 100 | 100 | 79 | 62 |
| Rv0309 | - | 218 | 0 | 0 | 0 | 0 | 0 | 0 | 0 | 44 | 0 | 0 | 46 | 48 | 75 | 85 | 85 | 85 | 100 | 72 | 78 | 78 | 78 | 80 | 100 | 100 | 100 | 92 | 75 |
| Rv0310c | - | 163 | 0 | 0 | 0 | 0 | 0 | 0 | 0 | 68 | 66 | 65 | 67 | 66 | 64 | 84 | 86 | 84 | 100 | 80 | 79 | 78 | 78 | 84 | 100 | 100 | 100 | 86 | 80 |
| Rv0311 | - | 409 | 0 | 0 | 0 | 0 | 0 | 0 | 0 | 0 | 66 | 67 | 67 | 67 | 83 | 88 | 90 | 88 | 100 | 81 | 82 | 82 | 82 | 82 | 100 | 100 | 100 | 89 | 81 |
| Rv0312 | - | 620 | 0 | 0 | 41 | 0 | 0 | 45 | 0 | 38 | 39 | 41 | 41 | 42 | 50 | 73 | 77 | 69 | 99 | 54 | 58 | 57 | 57 | 56 | 100 | 100 | 100 | 77 | 55 |
| Rv0313 | - | 128 | 0 | 0 | 0 | 0 | 0 | 0 | 0 | 0 | 64 | 0 | 73 | 73 | 0 | 89 | 90 | 90 | 100 | 82 | 72 | 72 | 72 | 76 | 100 | 100 | 100 | 86 | 76 |
| Rv0314c | - | 220 | 0 | 0 | 0 | 0 | 0 | 0 | 0 | 0 | 0 | 0 | 0 | 0 | 57 | 69 | 68 | 69 | 99 | 55 | 57 | 57 | 57 | 51 | 100 | 100 | 100 | 69 | 55 |
| Rv0315 | - | 294 | 0 | 0 | 0 | 0 | 0 | 0 | 0 | 0 | 0 | 0 | 46 | 43 | 73 | 69 | 92 | 69 | 99 | 68 | 65 | 65 | 65 | 68 | 99 | 100 | 99 | 92 | 67 |
| Rv0316 | - | 204 | 0 | 0 | 0 | 52 | 0 | 0 | 0 | 0 | 52 | 0 | 0 | 0 | 0 | 68 | 69 | 69 | 100 | 0 | 0 | 0 | 0 | 0 | 100 | 100 | 100 | 80 | 0 |

| | | | | | | | | | | | | | | | | | | | | | | | | | | | | | |
|---|---|---|---|---|---|---|---|---|---|---|---|---|---|---|---|---|---|---|---|---|---|---|---|---|---|---|---|---|---|
| Rv0317c | *glpQ2* | 256 | 43 | 0 | 45 | 0 | 0 | 0 | 0 | 52 | 0 | 0 | 0 | 0 | 0 | 0 | 82 | 0 | 100 | 0 | 0 | 0 | 0 | 0 | 100 | 100 | 100 | 81 | 0 |
| Rv0318c | - | 264 | 0 | 0 | 0 | 0 | 0 | 0 | 0 | 0 | 0 | 0 | 0 | 0 | 0 | 0 | 84 | 0 | 99 | 0 | 0 | 0 | 0 | 0 | 99 | 100 | 99 | 85 | 0 |
| Rv0319 | *pcp* | 222 | 0 | 0 | 0 | 0 | 0 | 0 | 0 | 0 | 0 | 0 | 0 | 0 | 0 | 0 | 91 | 0 | 100 | 0 | 0 | 0 | 0 | 0 | 100 | 100 | 99 | 83 | 0 |
| Rv0320 | - | 220 | 0 | 0 | 0 | 0 | 0 | 0 | 0 | 46 | 0 | 0 | 0 | 0 | 50 | 75 | 79 | 75 | 100 | 66 | 69 | 69 | 69 | 70 | 100 | 100 | 100 | 77 | 68 |
| Rv0321 | *dcd* | 190 | 85 | 89 | 86 | 87 | 86 | 84 | 90 | 85 | 95 | 94 | 95 | 96 | 94 | 97 | 97 | 97 | 99 | 96 | 96 | 96 | 96 | 94 | 100 | 100 | 100 | 96 | 96 |
| Rv0322 | *udgA* | 443 | 77 | 54 | 77 | 76 | 75 | 74 | 64 | 74 | 82 | 81 | 85 | 84 | 87 | 94 | 94 | 94 | 100 | 88 | 79 | 78 | 78 | 91 | 100 | 100 | 100 | 92 | 90 |
| Rv0323c | - | 223 | 48 | 48 | 46 | 48 | 51 | 0 | 53 | 49 | 47 | 49 | 48 | 48 | 48 | 49 | 50 | 49 | 99 | 48 | 49 | 49 | 49 | 50 | 99 | 99 | 99 | 50 | 45 |
| Rv0324 | - | 226 | 51 | 0 | 0 | 0 | 0 | 0 | 0 | 0 | 69 | 0 | 0 | 0 | 0 | 0 | 0 | 0 | 99 | 0 | 64 | 64 | 64 | 0 | 99 | 100 | 100 | 0 | 0 |
| Rv0325 | - | 74 | 0 | 0 | 0 | 0 | 0 | 0 | 0 | 0 | 0 | 0 | 0 | 0 | 0 | 0 | 0 | 0 | 100 | 0 | 0 | 0 | 0 | 0 | 100 | 100 | 100 | 0 | 0 |
| Rv0326 | - | 151 | 0 | 0 | 0 | 0 | 0 | 0 | 0 | 0 | 0 | 0 | 0 | 0 | 0 | 0 | 0 | 0 | 100 | 0 | 0 | 0 | 0 | 0 | 100 | 100 | 100 | 0 | 0 |
| Rv0327c | *cyp135A1* | 449 | 0 | 0 | 0 | 0 | 0 | 0 | 0 | 65 | 49 | 41 | 42 | 40 | 55 | 48 | 57 | 49 | 100 | 49 | 57 | 57 | 57 | 56 | 100 | 100 | 100 | 49 | 57 |
| Rv0328 | - | 200 | 0 | 0 | 0 | 0 | 0 | 0 | 0 | 0 | 48 | 0 | 50 | 0 | 0 | 0 | 0 | 0 | 100 | 0 | 0 | 0 | 0 | 0 | 100 | 100 | 100 | 0 | 0 |
| Rv0329c | - | 208 | 0 | 0 | 47 | 0 | 0 | 48 | 0 | 0 | 49 | 0 | 0 | 0 | 0 | 0 | 0 | 0 | 99 | 46 | 45 | 45 | 45 | 0 | 100 | 100 | 100 | 0 | 0 |
| Rv0330c | - | 246 | 0 | 0 | 0 | 0 | 0 | 0 | 0 | 0 | 0 | 0 | 0 | 0 | 0 | 0 | 0 | 0 | 100 | 0 | 0 | 0 | 0 | 0 | 100 | 100 | 100 | 0 | 0 |
| Rv0331 | - | 388 | 0 | 0 | 0 | 0 | 0 | 0 | 0 | 46 | 0 | 0 | 0 | 0 | 57 | 0 | 0 | 0 | 100 | 39 | 39 | 39 | 39 | 0 | 99 | 100 | 100 | 0 | 39 |
| Rv0332 | - | 261 | 0 | 0 | 0 | 0 | 0 | 0 | 0 | 45 | 0 | 0 | 0 | 0 | 45 | 83 | 83 | 83 | 99 | 72 | 71 | 71 | 71 | 70 | 100 | 100 | 100 | 82 | 68 |
| Rv0333 | - | 124 | 0 | 0 | 0 | 0 | 0 | 0 | 0 | 0 | 0 | 76 | 73 | 73 | 0 | 91 | 87 | 86 | 100 | 77 | 78 | 78 | 78 | 77 | 100 | 100 | 100 | 87 | 78 |
| Rv0334 | *rmlA* | 288 | 73 | 74 | 74 | 74 | 80 | 79 | 76 | 0 | 88 | 86 | 86 | 87 | 90 | 94 | 95 | 94 | 100 | 91 | 92 | 92 | 92 | 92 | 100 | 100 | 100 | 95 | 94 |
| Rv0335c | *PE6* | 171 | 0 | 0 | 0 | 0 | 0 | 0 | 0 | 0 | 0 | 0 | 0 | 0 | 0 | 61 | 64 | 61 | 100 | 0 | 0 | 0 | 0 | 0 | 100 | 100 | 100 | 64 | 0 |
| Rv0336 | - | 503 | 41 | 51 | 53 | 50 | 49 | 0 | 0 | 42 | 0 | 47 | 42 | 41 | 53 | 76 | 81 | 76 | 99 | 54 | 69 | 69 | 69 | 58 | 100 | 100 | 100 | 63 | 53 |
| Rv0337c | *aspC* | 429 | 84 | 84 | 85 | 86 | 85 | 84 | 87 | 52 | 91 | 92 | 90 | 90 | 90 | 96 | 97 | 96 | 100 | 93 | 91 | 91 | 91 | 93 | 100 | 100 | 100 | 97 | 93 |
| Rv0338c | - | 882 | 52 | 53 | 45 | 43 | 58 | 45 | 57 | 61 | 61 | 61 | 60 | 60 | 64 | 82 | 82 | 87 | 99 | 76 | 78 | 78 | 78 | 76 | 99 | 100 | 99 | 82 | 75 |
| Rv0339c | - | 832 | 0 | 0 | 0 | 0 | 0 | 0 | 0 | 0 | 49 | 49 | 53 | 54 | 61 | 39 | 81 | 38 | 100 | 68 | 71 | 71 | 71 | 70 | 100 | 100 | 100 | 87 | 70 |
| Rv0340 | - | 179 | 0 | 0 | 0 | 0 | 0 | 0 | 0 | 0 | 59 | 0 | 0 | 0 | 52 | 0 | 72 | 0 | 100 | 63 | 73 | 73 | 73 | 54 | 100 | 100 | 100 | 72 | 78 |
| Rv0341 | *iniB* | 479 | 39 | 0 | 39 | 37 | 34 | 0 | 36 | 38 | 40 | 45 | 43 | 43 | 39 | 34 | 52 | 45 | 100 | 41 | 39 | 42 | 42 | 41 | 99 | 100 | 100 | 52 | 40 |
| Rv0342 | *iniA* | 640 | 0 | 0 | 0 | 0 | 41 | 0 | 0 | 0 | 63 | 67 | 65 | 65 | 73 | 0 | 89 | 0 | 99 | 80 | 79 | 79 | 79 | 79 | 100 | 100 | 100 | 88 | 81 |
| Rv0343 | *iniC* | 493 | 0 | 0 | 0 | 0 | 46 | 0 | 0 | 0 | 66 | 67 | 67 | 67 | 76 | 0 | 92 | 0 | 100 | 86 | 87 | 87 | 87 | 87 | 100 | 100 | 100 | 91 | 86 |
| Rv0344c | *lpqJ* | 186 | 0 | 0 | 0 | 0 | 0 | 0 | 0 | 0 | 0 | 0 | 0 | 0 | 51 | 82 | 82 | 82 | 100 | 69 | 67 | 67 | 67 | 70 | 100 | 100 | 100 | 89 | 70 |
| Rv0345 | - | 136 | 0 | 0 | 0 | 0 | 0 | 0 | 0 | 58 | 65 | 56 | 48 | 55 | 53 | 0 | 87 | 0 | 100 | 62 | 60 | 64 | 64 | 59 | 100 | 100 | 100 | 85 | 62 |
| Rv0346c | *ansP2* | 487 | 54 | 50 | 53 | 55 | 49 | 55 | 53 | 39 | 55 | 74 | 75 | 73 | 80 | 53 | 90 | 54 | 100 | 55 | 56 | 56 | 56 | 56 | 100 | 100 | 100 | 78 | 41 |
| Rv0347 | - | 328 | 0 | 0 | 0 | 0 | 0 | 0 | 0 | 0 | 45 | 0 | 0 | 0 | 0 | 0 | 47 | 0 | 100 | 0 | 0 | 0 | 0 | 0 | 100 | 100 | 100 | 49 | 0 |
| Rv0348 | - | 217 | 0 | 0 | 0 | 0 | 0 | 0 | 0 | 0 | 52 | 0 | 0 | 0 | 0 | 0 | 0 | 0 | 100 | 0 | 0 | 0 | 0 | 0 | 100 | 100 | 100 | 0 | 0 |
| Rv0349 | - | 219 | 0 | 0 | 0 | 0 | 0 | 0 | 0 | 0 | 47 | 0 | 0 | 0 | 0 | 0 | 0 | 0 | 100 | 0 | 0 | 0 | 0 | 0 | 100 | 100 | 100 | 0 | 0 |
| Rv0350 | *dnaK* | 625 | 87 | 87 | 89 | 89 | 87 | 88 | 88 | 83 | 91 | 92 | 91 | 91 | 94 | 96 | 97 | 96 | 100 | 95 | 94 | 94 | 94 | 93 | 99 | 100 | 100 | 96 | 95 |
| Rv0351 | *grpE* | 235 | 60 | 69 | 70 | 63 | 57 | 64 | 74 | 58 | 65 | 71 | 63 | 63 | 65 | 81 | 81 | 81 | 99 | 77 | 69 | 69 | 69 | 72 | 100 | 100 | 100 | 80 | 77 |
| Rv0352 | *dnaJ1* | 395 | 74 | 73 | 69 | 70 | 71 | 71 | 70 | 65 | 81 | 80 | 82 | 82 | 87 | 95 | 94 | 95 | 100 | 89 | 92 | 92 | 92 | 91 | 100 | 100 | 100 | 94 | 90 |
| Rv0353 | *hspR* | 126 | 79 | 76 | 72 | 73 | 64 | 78 | 70 | 69 | 84 | 86 | 86 | 85 | 88 | 92 | 93 | 93 | 100 | 87 | 91 | 91 | 91 | 81 | 100 | 100 | 100 | 94 | 88 |
| Rv0354c | *PPE7* | 141 | 0 | 0 | 0 | 0 | 0 | 0 | 0 | 0 | 0 | 0 | 0 | 0 | 0 | 0 | 0 | 0 | 100 | 0 | 0 | 0 | 0 | 0 | 100 | 100 | 100 | 0 | 0 |

| | | | | | | | | | | | | | | | | | | | | | | | | | | | | | |
|---|---|---|---|---|---|---|---|---|---|---|---|---|---|---|---|---|---|---|---|---|---|---|---|---|---|---|---|---|---|
| Rv0355c | *PPE8* | 3300 | 0 | 0 | 0 | 0 | 0 | 0 | 0 | 0 | 34 | 40 | 36 | 35 | 49 | 66 | 66 | 66 | 99 | 38 | 33 | 34 | 34 | 33 | 99 | 99 | 98 | 68 | 50 |
| Rv0356c | - | 214 | 0 | 0 | 0 | 0 | 0 | 0 | 0 | 0 | 69 | 65 | 70 | 68 | 71 | 91 | 90 | 91 | 100 | 75 | 75 | 74 | 74 | 71 | 100 | 100 | 100 | 91 | 75 |
| Rv0357c | *purA* | 432 | 79 | 76 | 77 | 77 | 77 | 79 | 78 | 75 | 89 | 91 | 92 | 92 | 93 | 94 | 96 | 95 | 100 | 93 | 93 | 93 | 93 | 93 | 100 | 100 | 100 | 96 | 92 |
| Rv0358 | - | 215 | 42 | 47 | 49 | 46 | 46 | 42 | 44 | 0 | 62 | 59 | 61 | 62 | 67 | 73 | 78 | 73 | 100 | 64 | 61 | 61 | 61 | 62 | 100 | 100 | 100 | 77 | 61 |
| Rv0359 | - | 259 | 0 | 0 | 0 | 0 | 0 | 0 | 0 | 0 | 67 | 68 | 70 | 70 | 72 | 83 | 83 | 83 | 99 | 81 | 82 | 82 | 82 | 82 | 100 | 100 | 100 | 81 | 82 |
| Rv0360c | - | 145 | 64 | 63 | 65 | 64 | 71 | 64 | 69 | 60 | 74 | 83 | 86 | 86 | 81 | 91 | 90 | 90 | 100 | 87 | 89 | 89 | 89 | 86 | 100 | 100 | 100 | 90 | 86 |
| Rv0361 | - | 275 | 0 | 0 | 0 | 0 | 0 | 0 | 0 | 0 | 46 | 41 | 42 | 42 | 65 | 74 | 70 | 70 | 100 | 64 | 65 | 65 | 65 | 62 | 100 | 100 | 100 | 79 | 63 |
| Rv0362 | *mgtE* | 460 | 52 | 44 | 54 | 54 | 0 | 0 | 57 | 47 | 55 | 0 | 0 | 0 | 45 | 44 | 91 | 45 | 99 | 51 | 47 | 47 | 47 | 76 | 100 | 100 | 100 | 96 | 77 |
| Rv0363c | *fba* | 344 | 83 | 83 | 85 | 84 | 83 | 0 | 83 | 82 | 94 | 91 | 91 | 91 | 92 | 0 | 95 | 0 | 100 | 93 | 94 | 94 | 94 | 95 | 100 | 100 | 100 | 95 | 94 |
| Rv0364 | - | 227 | 61 | 58 | 47 | 64 | 63 | 66 | 57 | 58 | 68 | 67 | 70 | 70 | 76 | 87 | 90 | 87 | 100 | 61 | 61 | 61 | 61 | 61 | 100 | 100 | 100 | 90 | 62 |
| Rv0365c | - | 376 | 56 | 57 | 56 | 55 | 56 | 58 | 55 | 0 | 68 | 68 | 70 | 70 | 73 | 87 | 88 | 88 | 100 | 80 | 82 | 82 | 82 | 85 | 100 | 100 | 100 | 89 | 80 |
| Rv0366c | - | 197 | 0 | 0 | 0 | 0 | 0 | 0 | 0 | 0 | 0 | 0 | 0 | 70 | 0 | 83 | 0 | 83 | 100 | 80 | 77 | 77 | 77 | 79 | 100 | 100 | 99 | 0 | 61 |
| Rv0367c | - | 129 | 0 | 0 | 0 | 0 | 0 | 0 | 0 | 0 | 0 | 0 | 0 | 80 | 0 | 91 | 0 | 92 | 100 | 88 | 86 | 86 | 86 | 87 | 100 | 100 | 100 | 0 | 84 |
| Rv0368c | - | 403 | 0 | 0 | 0 | 0 | 0 | 0 | 0 | 44 | 0 | 0 | 69 | 71 | 0 | 0 | 80 | 0 | 99 | 0 | 68 | 69 | 69 | 69 | 99 | 100 | 100 | 80 | 0 |
| Rv0369c | - | 171 | 0 | 0 | 0 | 0 | 0 | 0 | 0 | 63 | 0 | 0 | 75 | 71 | 0 | 0 | 77 | 0 | 100 | 53 | 74 | 74 | 74 | 71 | 100 | 100 | 100 | 80 | 57 |
| Rv0370c | - | 298 | 0 | 0 | 0 | 0 | 0 | 0 | 0 | 64 | 60 | 59 | 85 | 83 | 61 | 62 | 62 | 62 | 99 | 61 | 84 | 84 | 84 | 85 | 100 | 100 | 100 | 90 | 62 |
| Rv0371c | - | 197 | 0 | 0 | 0 | 0 | 0 | 0 | 0 | 64 | 0 | 0 | 0 | 0 | 0 | 0 | 82 | 0 | 100 | 0 | 0 | 0 | 0 | 0 | 100 | 100 | 100 | 81 | 0 |
| Rv0372c | - | 251 | 0 | 0 | 0 | 0 | 0 | 0 | 0 | 52 | 0 | 0 | 76 | 76 | 0 | 0 | 90 | 0 | 99 | 0 | 85 | 85 | 85 | 84 | 100 | 100 | 100 | 91 | 0 |
| Rv0373c | - | 799 | 0 | 0 | 0 | 0 | 0 | 0 | 0 | 87 | 42 | 49 | 93 | 93 | 40 | 0 | 96 | 0 | 99 | 53 | 94 | 94 | 94 | 94 | 100 | 100 | 100 | 95 | 51 |
| Rv0374c | - | 159 | 0 | 0 | 0 | 0 | 0 | 0 | 0 | 84 | 48 | 60 | 89 | 89 | 52 | 0 | 93 | 0 | 100 | 64 | 92 | 91 | 91 | 89 | 100 | 100 | 100 | 92 | 65 |
| Rv0375c | - | 286 | 0 | 0 | 0 | 0 | 0 | 0 | 0 | 81 | 0 | 47 | 82 | 82 | 0 | 0 | 0 | 0 | 100 | 49 | 85 | 85 | 85 | 88 | 100 | 100 | 100 | 90 | 49 |
| Rv0376c | - | 380 | 0 | 0 | 0 | 0 | 0 | 0 | 0 | 79 | 58 | 62 | 85 | 85 | 61 | 0 | 90 | 0 | 100 | 84 | 85 | 85 | 85 | 87 | 99 | 100 | 100 | 64 | 83 |
| Rv0377 | - | 321 | 0 | 0 | 0 | 0 | 0 | 0 | 0 | 68 | 0 | 43 | 0 | 0 | 0 | 0 | 95 | 0 | 99 | 0 | 84 | 84 | 84 | 83 | 100 | 100 | 0 | 95 | 0 |
| Rv0378 | - | 73 | 0 | 0 | 0 | 0 | 0 | 0 | 0 | 0 | 0 | 0 | 0 | 0 | 0 | 0 | 61 | 0 | 100 | 61 | 55 | 55 | 55 | 53 | 100 | 100 | 57 | 60 | 56 |
| Rv0379 | *secE2* | 71 | 0 | 0 | 0 | 0 | 0 | 0 | 0 | 0 | 0 | 0 | 0 | 0 | 0 | 0 | 100 | 0 | 100 | 0 | 0 | 0 | 0 | 0 | 100 | 100 | 100 | 98 | 0 |
| Rv0380c | - | 183 | 81 | 82 | 83 | 85 | 82 | 82 | 85 | 72 | 83 | 84 | 86 | 85 | 89 | 94 | 92 | 94 | 100 | 92 | 90 | 90 | 90 | 91 | 100 | 100 | 100 | 92 | 95 |
| Rv0381c | - | 302 | 0 | 0 | 0 | 0 | 0 | 0 | 0 | 0 | 0 | 0 | 0 | 0 | 70 | 81 | 82 | 82 | 100 | 75 | 75 | 74 | 74 | 73 | 99 | 100 | 100 | 83 | 74 |
| Rv0382c | *pyrE* | 179 | 81 | 86 | 84 | 87 | 86 | 85 | 84 | 50 | 84 | 86 | 86 | 86 | 90 | 93 | 96 | 94 | 100 | 53 | 50 | 50 | 50 | 51 | 100 | 100 | 100 | 95 | 53 |
| Rv0383c | - | 284 | 44 | 46 | 40 | 44 | 45 | 0 | 0 | 0 | 50 | 53 | 58 | 57 | 78 | 88 | 89 | 88 | 100 | 79 | 80 | 80 | 80 | 84 | 100 | 100 | 100 | 89 | 80 |
| Rv0384c | *clpB* | 848 | 85 | 85 | 86 | 86 | 86 | 84 | 84 | 80 | 90 | 90 | 90 | 90 | 93 | 97 | 97 | 97 | 100 | 96 | 96 | 96 | 96 | 96 | 100 | 100 | 100 | 97 | 96 |
| Rv0385 | - | 390 | 42 | 55 | 49 | 51 | 51 | 44 | 46 | 0 | 59 | 58 | 62 | 62 | 72 | 85 | 85 | 85 | 100 | 77 | 79 | 80 | 80 | 78 | 100 | 100 | 100 | 83 | 78 |
| Rv0386 | - | 1085 | 0 | 0 | 0 | 75 | 0 | 0 | 0 | 37 | 47 | 48 | 50 | 52 | 38 | 53 | 69 | 53 | 99 | 40 | 38 | 35 | 35 | 56 | 100 | 100 | 100 | 56 | 73 |
| Rv0387c | - | 244 | 0 | 0 | 0 | 38 | 0 | 0 | 0 | 0 | 0 | 0 | 0 | 0 | 0 | 0 | 78 | 0 | 100 | 36 | 38 | 0 | 0 | 0 | 100 | 100 | 100 | 39 | 0 |
| Rv0388c | *PPE9* | 180 | 0 | 0 | 0 | 0 | 0 | 0 | 0 | 0 | 0 | 0 | 0 | 0 | 51 | 77 | 77 | 77 | 96 | 51 | 53 | 53 | 53 | 52 | 97 | 97 | 97 | 76 | 54 |
| Rv0389 | *purT* | 419 | 53 | 48 | 61 | 62 | 57 | 58 | 58 | 0 | 0 | 62 | 61 | 62 | 62 | 72 | 70 | 74 | 99 | 61 | 67 | 67 | 67 | 67 | 100 | 100 | 100 | 0 | 61 |
| Rv0390 | - | 140 | 0 | 0 | 0 | 0 | 0 | 0 | 0 | 0 | 72 | 80 | 79 | 80 | 77 | 89 | 83 | 89 | 100 | 81 | 80 | 80 | 80 | 81 | 100 | 100 | 100 | 0 | 82 |
| Rv0391 | *metZ* | 406 | 56 | 50 | 57 | 51 | 52 | 57 | 52 | 52 | 90 | 88 | 87 | 88 | 91 | 96 | 96 | 96 | 100 | 92 | 92 | 92 | 92 | 93 | 100 | 100 | 100 | 56 | 91 |
| Rv0392c | *ndhA* | 470 | 70 | 72 | 71 | 72 | 71 | 69 | 71 | 72 | 80 | 77 | 80 | 79 | 77 | 90 | 91 | 90 | 99 | 81 | 78 | 78 | 78 | 78 | 100 | 100 | 100 | 77 | 80 |

| | | | | | | | | | | | | | | | | | | | | | | | | | | | | | |
|---|---|---|---|---|---|---|---|---|---|---|---|---|---|---|---|---|---|---|---|---|---|---|---|---|---|---|---|---|---|
| Rv0393 | - | 441 | 42 | 53 | 50 | 46 | 42 | 0 | 0 | 38 | 0 | 46 | 39 | 39 | 52 | 68 | 67 | 69 | 100 | 55 | 70 | 70 | 70 | 56 | 100 | 100 | 100 | 66 | 50 |
| Rv0394c | - | 239 | 0 | 0 | 0 | 0 | 0 | 0 | 0 | 0 | 0 | 0 | 0 | 0 | 0 | 0 | 0 | 0 | 100 | 0 | 0 | 0 | 0 | 0 | 100 | 100 | 100 | 0 | 0 |
| Rv0395 | - | 134 | 0 | 0 | 0 | 0 | 0 | 0 | 0 | 0 | 0 | 0 | 0 | 0 | 0 | 0 | 0 | 0 | 99 | 0 | 0 | 0 | 0 | 0 | 99 | 100 | 99 | 0 | 0 |
| Rv0396 | - | 130 | 0 | 0 | 0 | 0 | 0 | 0 | 0 | 0 | 0 | 0 | 0 | 0 | 0 | 0 | 0 | 0 | 100 | 0 | 0 | 0 | 0 | 0 | 96 | 100 | 100 | 0 | 0 |
| Rv0397 | - | 122 | 0 | 0 | 0 | 0 | 0 | 0 | 0 | 0 | 0 | 0 | 0 | 0 | 0 | 66 | 60 | 67 | 100 | 56 | 58 | 58 | 58 | 48 | 100 | 100 | 100 | 60 | 51 |
| Rv0398c | - | 213 | 0 | 0 | 0 | 0 | 0 | 0 | 0 | 0 | 0 | 0 | 0 | 0 | 0 | 69 | 69 | 69 | 100 | 0 | 0 | 0 | 0 | 0 | 100 | 100 | 100 | 62 | 0 |
| Rv0399c | *lpqK* | 409 | 43 | 0 | 45 | 0 | 0 | 0 | 0 | 0 | 43 | 51 | 77 | 52 | 50 | 0 | 88 | 0 | 100 | 45 | 78 | 78 | 78 | 50 | 100 | 100 | 99 | 0 | 81 |
| Rv0400c | *fadE7* | 395 | 69 | 0 | 71 | 46 | 74 | 49 | 49 | 78 | 75 | 78 | 79 | 90 | 77 | 92 | 92 | 92 | 100 | 75 | 79 | 79 | 79 | 80 | 100 | 100 | 99 | 92 | 77 |
| Rv0401 | - | 123 | 54 | 58 | 0 | 0 | 49 | 54 | 57 | 54 | 64 | 61 | 66 | 66 | 80 | 83 | 85 | 83 | 100 | 78 | 77 | 77 | 77 | 82 | 100 | 100 | 100 | 85 | 78 |
| Rv0402c | *mmpL1* | 958 | 41 | 51 | 43 | 54 | 38 | 51 | 42 | 46 | 52 | 47 | 41 | 41 | 76 | 76 | 86 | 74 | 75 | 75 | 75 | 75 | 75 | 75 | 100 | 100 | 100 | 86 | 75 |
| Rv0403c | *mmpS1* | 142 | 0 | 0 | 0 | 0 | 0 | 0 | 0 | 0 | 0 | 0 | 0 | 0 | 65 | 68 | 70 | 70 | 100 | 62 | 60 | 60 | 60 | 64 | 99 | 100 | 100 | 80 | 64 |
| Rv0404 | *fadD30* | 585 | 49 | 50 | 49 | 49 | 50 | 38 | 50 | 38 | 49 | 50 | 50 | 50 | 57 | 59 | 74 | 59 | 99 | 63 | 61 | 61 | 61 | 60 | 100 | 100 | 99 | 62 | 62 |
| Rv0405 | *pks6* | 1402 | 52 | 51 | 53 | 52 | 50 | 0 | 48 | 40 | 50 | 49 | 57 | 57 | 55 | 61 | 68 | 61 | 99 | 55 | 56 | 56 | 56 | 55 | 100 | 100 | 100 | 61 | 57 |
| Rv0406c | - | 272 | 0 | 0 | 50 | 0 | 0 | 0 | 0 | 0 | 46 | 54 | 55 | 0 | 66 | 85 | 79 | 85 | 100 | 71 | 71 | 71 | 71 | 71 | 100 | 100 | 100 | 80 | 72 |
| Rv0407 | *fgd1* | 336 | 0 | 0 | 0 | 0 | 0 | 0 | 0 | 48 | 90 | 90 | 91 | 91 | 92 | 94 | 95 | 94 | 100 | 93 | 93 | 93 | 93 | 94 | 100 | 100 | 100 | 95 | 93 |
| Rv0408 | *pta* | 690 | 63 | 64 | 58 | 65 | 70 | 74 | 75 | 72 | 79 | 79 | 79 | 79 | 83 | 87 | 87 | 87 | 99 | 84 | 84 | 84 | 84 | 86 | 100 | 100 | 100 | 85 | 84 |
| Rv0409 | *ackA* | 385 | 68 | 70 | 68 | 68 | 69 | 68 | 70 | 65 | 68 | 72 | 72 | 73 | 76 | 88 | 89 | 88 | 100 | 80 | 78 | 78 | 78 | 79 | 100 | 100 | 100 | 88 | 80 |
| Rv0410c | *pknG* | 750 | 61 | 59 | 59 | 55 | 60 | 56 | 61 | 49 | 67 | 66 | 68 | 67 | 82 | 91 | 93 | 91 | 100 | 87 | 88 | 88 | 88 | 88 | 100 | 100 | 100 | 92 | 87 |
| Rv0411c | *glnH* | 328 | 59 | 55 | 56 | 57 | 56 | 54 | 51 | 46 | 67 | 66 | 68 | 68 | 76 | 91 | 93 | 91 | 100 | 85 | 83 | 83 | 83 | 82 | 100 | 100 | 100 | 93 | 83 |
| Rv0412c | - | 439 | 52 | 53 | 54 | 53 | 51 | 51 | 50 | 0 | 54 | 58 | 58 | 57 | 77 | 91 | 94 | 91 | 99 | 86 | 86 | 86 | 86 | 87 | 99 | 100 | 99 | 94 | 85 |
| Rv0413 | *mutT3* | 217 | 63 | 0 | 64 | 62 | 59 | 53 | 63 | 0 | 65 | 66 | 63 | 63 | 71 | 85 | 85 | 85 | 100 | 83 | 79 | 79 | 79 | 84 | 100 | 100 | 100 | 80 | 86 |
| Rv0414c | *thiE* | 222 | 44 | 43 | 47 | 45 | 46 | 45 | 46 | 47 | 76 | 78 | 76 | 76 | 80 | 89 | 89 | 89 | 100 | 82 | 84 | 85 | 85 | 82 | 100 | 100 | 100 | 87 | 83 |
| Rv0415 | *thiO* | 340 | 47 | 51 | 53 | 46 | 47 | 48 | 47 | 52 | 65 | 70 | 69 | 68 | 77 | 92 | 89 | 92 | 99 | 84 | 85 | 85 | 85 | 88 | 100 | 100 | 99 | 89 | 88 |
| Rv0416 | *thiS* | 68 | 0 | 0 | 0 | 0 | 0 | 0 | 0 | 0 | 0 | 0 | 0 | 0 | 0 | 80 | 86 | 80 | 100 | 70 | 76 | 76 | 76 | 71 | 100 | 100 | 100 | 86 | 76 |
| Rv0417 | *thiG* | 252 | 77 | 78 | 82 | 78 | 74 | 78 | 75 | 73 | 87 | 88 | 90 | 88 | 91 | 93 | 94 | 94 | 99 | 91 | 92 | 92 | 92 | 91 | 99 | 100 | 99 | 96 | 91 |
| Rv0418 | *lpqL* | 500 | 0 | 0 | 0 | 0 | 0 | 0 | 0 | 0 | 0 | 65 | 65 | 65 | 71 | 88 | 88 | 88 | 100 | 76 | 76 | 76 | 76 | 75 | 100 | 100 | 100 | 90 | 78 |
| Rv0419 | *lpqM* | 498 | 0 | 0 | 0 | 0 | 0 | 0 | 0 | 0 | 59 | 59 | 58 | 57 | 58 | 86 | 89 | 86 | 99 | 68 | 64 | 64 | 64 | 68 | 100 | 100 | 100 | 88 | 67 |
| Rv0420c | - | 136 | 0 | 0 | 0 | 0 | 0 | 0 | 0 | 0 | 0 | 0 | 0 | 0 | 0 | 57 | 58 | 58 | 99 | 0 | 60 | 60 | 60 | 55 | 100 | 100 | 100 | 72 | 0 |
| Rv0421c | - | 209 | 0 | 0 | 0 | 0 | 0 | 0 | 0 | 45 | 44 | 57 | 63 | 62 | 77 | 88 | 90 | 88 | 100 | 81 | 82 | 82 | 82 | 80 | 100 | 100 | 100 | 87 | 82 |
| Rv0422c | *thiD* | 265 | 53 | 52 | 53 | 56 | 54 | 56 | 51 | 53 | 76 | 78 | 78 | 78 | 84 | 91 | 87 | 90 | 100 | 84 | 83 | 83 | 83 | 81 | 100 | 100 | 100 | 87 | 84 |
| Rv0423c | *thiC* | 547 | 82 | 82 | 83 | 82 | 79 | 83 | 81 | 73 | 86 | 86 | 87 | 87 | 91 | 94 | 95 | 94 | 100 | 88 | 0 | 0 | 0 | 90 | 100 | 100 | 100 | 95 | 88 |
| Rv0424c | - | 91 | 0 | 0 | 0 | 0 | 0 | 0 | 0 | 0 | 0 | 63 | 75 | 73 | 0 | 88 | 87 | 88 | 100 | 0 | 59 | 0 | 0 | 0 | 100 | 100 | 100 | 87 | 59 |
| Rv0425c | *ctpH* | 1539 | 41 | 39 | 46 | 49 | 43 | 43 | 42 | 41 | 49 | 61 | 66 | 65 | 44 | 49 | 80 | 49 | 99 | 43 | 62 | 43 | 43 | 46 | 100 | 100 | 100 | 84 | 61 |
| Rv0426c | - | 147 | 0 | 0 | 0 | 0 | 0 | 0 | 0 | 0 | 0 | 58 | 62 | 71 | 0 | 81 | 87 | 81 | 100 | 0 | 69 | 0 | 0 | 0 | 100 | 100 | 100 | 87 | 68 |
| Rv0427c | *xthA* | 291 | 59 | 63 | 68 | 68 | 61 | 64 | 62 | 72 | 75 | 81 | 79 | 77 | 80 | 89 | 94 | 89 | 100 | 82 | 83 | 83 | 83 | 81 | 100 | 100 | 100 | 93 | 80 |
| Rv0428c | - | 302 | 48 | 46 | 48 | 45 | 49 | 44 | 46 | 38 | 49 | 51 | 52 | 51 | 57 | 61 | 74 | 61 | 99 | 62 | 64 | 64 | 64 | 64 | 100 | 100 | 100 | 69 | 62 |
| Rv0429c | *def* | 197 | 71 | 74 | 71 | 72 | 74 | 70 | 67 | 51 | 80 | 82 | 78 | 78 | 85 | 91 | 91 | 91 | 99 | 88 | 88 | 88 | 88 | 87 | 100 | 100 | 100 | 91 | 90 |
| Rv0430 | - | 102 | 62 | 0 | 0 | 0 | 60 | 0 | 69 | 80 | 84 | 97 | 97 | 93 | 80 | 99 | 98 | 99 | 100 | 95 | 93 | 93 | 93 | 95 | 100 | 100 | 100 | 99 | 94 |

| | | | | | | | | | | | | | | | | | | | | | | | | | | | | | |
|---|---|---|---|---|---|---|---|---|---|---|---|---|---|---|---|---|---|---|---|---|---|---|---|---|---|---|---|---|---|
| Rv0431 | - | 164 | 0 | 0 | 0 | 0 | 0 | 0 | 0 | 0 | 50 | 46 | 51 | 53 | 67 | 81 | 82 | 81 | 98 | 70 | 69 | 69 | 69 | 74 | 100 | 100 | 100 | 81 | 73 |
| Rv0432 | *sodC* | 240 | 0 | 48 | 53 | 0 | 0 | 0 | 0 | 0 | 62 | 67 | 66 | 69 | 71 | 82 | 79 | 82 | 100 | 72 | 73 | 73 | 73 | 75 | 100 | 100 | 100 | 78 | 73 |
| Rv0433 | - | 376 | 67 | 70 | 67 | 69 | 68 | 69 | 65 | 77 | 75 | 76 | 75 | 75 | 87 | 92 | 94 | 92 | 100 | 90 | 91 | 91 | 91 | 90 | 100 | 100 | 100 | 94 | 90 |
| Rv0434 | - | 217 | 0 | 0 | 0 | 0 | 0 | 0 | 0 | 0 | 0 | 63 | 60 | 60 | 66 | 77 | 77 | 77 | 99 | 75 | 71 | 71 | 71 | 71 | 100 | 100 | 100 | 77 | 76 |
| Rv0435c | - | 728 | 51 | 49 | 56 | 57 | 57 | 61 | 58 | 59 | 78 | 82 | 81 | 81 | 84 | 91 | 93 | 91 | 100 | 85 | 85 | 85 | 85 | 84 | 100 | 100 | 100 | 92 | 85 |
| Rv0436c | *pssA* | 286 | 0 | 0 | 0 | 0 | 0 | 0 | 0 | 0 | 71 | 73 | 71 | 70 | 73 | 91 | 92 | 91 | 99 | 76 | 79 | 79 | 79 | 77 | 99 | 100 | 100 | 91 | 80 |
| Rv0437c | *psd* | 231 | 0 | 0 | 0 | 0 | 0 | 0 | 0 | 0 | 74 | 72 | 72 | 71 | 74 | 85 | 86 | 85 | 100 | 80 | 78 | 79 | 79 | 80 | 100 | 100 | 100 | 86 | 80 |
| Rv0438c | *moeA2* | 405 | 49 | 53 | 52 | 52 | 52 | 46 | 51 | 59 | 56 | 54 | 59 | 59 | 74 | 86 | 86 | 86 | 100 | 77 | 78 | 77 | 77 | 79 | 100 | 100 | 100 | 85 | 77 |
| Rv0439c | - | 311 | 48 | 42 | 45 | 0 | 40 | 0 | 41 | 54 | 63 | 65 | 65 | 64 | 79 | 86 | 89 | 85 | 100 | 78 | 81 | 81 | 81 | 79 | 100 | 100 | 100 | 89 | 78 |
| Rv0440 | *groEL* | 540 | 87 | 87 | 88 | 88 | 88 | 88 | 87 | 89 | 93 | 92 | 93 | 93 | 95 | 95 | 95 | 95 | 100 | 94 | 95 | 95 | 95 | 95 | 100 | 100 | 100 | 95 | 95 |
| Rv0441c | - | 142 | 0 | 0 | 0 | 0 | 0 | 0 | 0 | 53 | 0 | 0 | 0 | 0 | 0 | 76 | 80 | 76 | 100 | 58 | 0 | 0 | 0 | 65 | 100 | 100 | 100 | 79 | 57 |
| Rv0442c | *PPE10* | 487 | 0 | 0 | 0 | 36 | 0 | 0 | 0 | 0 | 48 | 39 | 37 | 37 | 54 | 83 | 76 | 83 | 99 | 53 | 52 | 52 | 52 | 56 | 100 | 100 | 100 | 76 | 56 |
| Rv0443 | - | 171 | 0 | 0 | 0 | 0 | 0 | 54 | 0 | 71 | 67 | 0 | 64 | 66 | 75 | 94 | 94 | 93 | 100 | 84 | 86 | 86 | 86 | 89 | 100 | 100 | 100 | 94 | 85 |
| Rv0444c | - | 232 | 0 | 0 | 0 | 0 | 0 | 0 | 0 | 45 | 0 | 57 | 58 | 59 | 0 | 0 | 87 | 0 | 99 | 72 | 72 | 72 | 72 | 0 | 100 | 100 | 100 | 87 | 69 |
| Rv0445c | *sigK* | 187 | 55 | 57 | 0 | 0 | 56 | 0 | 0 | 63 | 67 | 72 | 71 | 72 | 45 | 44 | 87 | 44 | 100 | 81 | 80 | 79 | 79 | 44 | 100 | 100 | 100 | 87 | 81 |
| Rv0446c | - | 256 | 0 | 0 | 0 | 0 | 0 | 0 | 0 | 62 | 0 | 74 | 74 | 73 | 0 | 0 | 0 | 0 | 100 | 0 | 43 | 43 | 43 | 0 | 100 | 100 | 99 | 85 | 0 |
| Rv0447c | *ufaA1* | 427 | 45 | 41 | 41 | 46 | 42 | 45 | 43 | 66 | 50 | 68 | 67 | 67 | 45 | 43 | 81 | 43 | 100 | 43 | 45 | 45 | 45 | 45 | 100 | 100 | 100 | 81 | 45 |
| Rv0448c | - | 221 | 0 | 0 | 0 | 0 | 0 | 0 | 0 | 52 | 0 | 65 | 68 | 68 | 0 | 0 | 88 | 0 | 100 | 69 | 67 | 67 | 67 | 0 | 100 | 100 | 100 | 86 | 73 |
| Rv0449c | - | 439 | 0 | 0 | 0 | 0 | 0 | 0 | 0 | 70 | 0 | 68 | 72 | 72 | 0 | 0 | 0 | 0 | 100 | 0 | 0 | 0 | 0 | 0 | 100 | 100 | 100 | 86 | 0 |
| Rv0450c | *mmpL4* | 967 | 42 | 41 | 43 | 43 | 40 | 53 | 42 | 46 | 55 | 50 | 43 | 43 | 78 | 80 | 91 | 79 | 99 | 79 | 79 | 79 | 79 | 78 | 99 | 100 | 100 | 87 | 80 |
| Rv0451c | *mmpS4* | 140 | 0 | 0 | 0 | 0 | 0 | 0 | 0 | 0 | 0 | 0 | 0 | 0 | 75 | 80 | 92 | 80 | 100 | 65 | 70 | 70 | 70 | 77 | 100 | 100 | 100 | 71 | 70 |
| Rv0452 | - | 236 | 0 | 0 | 0 | 0 | 0 | 0 | 0 | 0 | 0 | 44 | 0 | 0 | 81 | 86 | 91 | 86 | 100 | 0 | 0 | 0 | 0 | 0 | 100 | 100 | 100 | 0 | 0 |
| Rv0453 | *PPE11* | 518 | 0 | 0 | 0 | 0 | 0 | 0 | 0 | 0 | 43 | 46 | 45 | 43 | 55 | 63 | 65 | 63 | 100 | 50 | 53 | 53 | 53 | 53 | 99 | 100 | 100 | 64 | 52 |
| Rv0454 | - | 116 | 0 | 0 | 0 | 0 | 0 | 0 | 0 | 0 | 0 | 0 | 0 | 0 | 0 | 0 | 0 | 0 | 100 | 0 | 0 | 0 | 0 | 0 | 100 | 100 | 100 | 0 | 0 |
| Rv0455c | - | 148 | 0 | 0 | 0 | 0 | 0 | 0 | 0 | 0 | 0 | 0 | 0 | 0 | 72 | 88 | 88 | 85 | 100 | 70 | 67 | 66 | 66 | 65 | 100 | 100 | 100 | 88 | 65 |
| Rv0456A | - | 93 | 0 | 0 | 0 | 0 | 0 | 0 | 0 | 0 | 0 | 0 | 0 | 0 | 0 | 0 | 0 | 0 | 100 | 0 | 0 | 0 | 0 | 0 | 100 | 100 | 100 | 0 | 80 |
| Rv0456c | *echA2* | 304 | 42 | 0 | 42 | 0 | 44 | 41 | 44 | 42 | 51 | 59 | 54 | 49 | 53 | 96 | 96 | 96 | 100 | 45 | 90 | 90 | 90 | 53 | 100 | 100 | 100 | 96 | 91 |
| Rv0457c | - | 673 | 39 | 60 | 61 | 60 | 40 | 49 | 41 | 47 | 70 | 72 | 74 | 74 | 78 | 87 | 86 | 87 | 100 | 83 | 83 | 83 | 83 | 79 | 99 | 100 | 100 | 85 | 83 |
| Rv0458 | - | 507 | 81 | 80 | 82 | 82 | 80 | 52 | 82 | 83 | 84 | 89 | 90 | 90 | 90 | 93 | 94 | 93 | 100 | 91 | 91 | 91 | 91 | 92 | 100 | 100 | 100 | 94 | 91 |
| Rv0459 | - | 163 | 0 | 0 | 0 | 0 | 0 | 0 | 0 | 0 | 65 | 63 | 68 | 67 | 71 | 84 | 83 | 85 | 100 | 76 | 77 | 77 | 77 | 78 | 100 | 100 | 100 | 82 | 81 |
| Rv0460 | - | 79 | 0 | 0 | 0 | 0 | 0 | 0 | 0 | 0 | 0 | 0 | 0 | 0 | 78 | 86 | 88 | 88 | 100 | 77 | 77 | 77 | 77 | 0 | 100 | 100 | 100 | 89 | 77 |
| Rv0461 | - | 174 | 0 | 0 | 0 | 0 | 0 | 0 | 0 | 0 | 0 | 0 | 0 | 0 | 49 | 65 | 68 | 65 | 100 | 52 | 54 | 54 | 54 | 0 | 100 | 100 | 100 | 82 | 55 |
| Rv0462 | *lpd* | 464 | 78 | 79 | 74 | 74 | 77 | 79 | 77 | 77 | 86 | 87 | 88 | 87 | 88 | 94 | 94 | 95 | 100 | 90 | 91 | 92 | 92 | 91 | 100 | 100 | 100 | 94 | 92 |
| Rv0463 | - | 97 | 0 | 0 | 0 | 0 | 0 | 0 | 0 | 0 | 0 | 0 | 0 | 0 | 0 | 68 | 74 | 68 | 100 | 0 | 54 | 54 | 54 | 56 | 100 | 100 | 100 | 74 | 55 |
| Rv0464c | - | 190 | 0 | 0 | 0 | 0 | 48 | 0 | 0 | 56 | 60 | 62 | 61 | 58 | 66 | 78 | 86 | 78 | 100 | 63 | 65 | 65 | 65 | 66 | 100 | 100 | 100 | 86 | 65 |
| Rv0465c | - | 474 | 72 | 70 | 72 | 71 | 72 | 70 | 70 | 0 | 85 | 87 | 86 | 86 | 92 | 95 | 95 | 95 | 99 | 90 | 92 | 92 | 92 | 93 | 100 | 100 | 100 | 96 | 90 |
| Rv0466 | - | 264 | 0 | 0 | 0 | 0 | 0 | 0 | 0 | 0 | 57 | 59 | 57 | 58 | 79 | 94 | 95 | 94 | 100 | 85 | 86 | 86 | 86 | 86 | 100 | 100 | 100 | 94 | 85 |
| Rv0467 | *icl* | 428 | 42 | 0 | 90 | 90 | 90 | 90 | 89 | 0 | 93 | 91 | 92 | 92 | 92 | 98 | 96 | 98 | 100 | 95 | 96 | 96 | 96 | 96 | 100 | 100 | 100 | 95 | 96 |

| | | | | | | | | | | | | | | | | | | | | | | | | | | | | | |
|---|---|---|---|---|---|---|---|---|---|---|---|---|---|---|---|---|---|---|---|---|---|---|---|---|---|---|---|---|---|
| Rv0468 | *fadB2* | 286 | 45 | 0 | 58 | 0 | 75 | 77 | 75 | 75 | 84 | 84 | 84 | 83 | 88 | 95 | 96 | 95 | 100 | 87 | 91 | 91 | 91 | 91 | 100 | 100 | 99 | 96 | 87 |
| Rv0469 | *umaA* | 286 | 49 | 0 | 0 | 50 | 0 | 48 | 44 | 48 | 47 | 51 | 50 | 49 | 84 | 93 | 95 | 93 | 100 | 75 | 74 | 74 | 74 | 85 | 100 | 100 | 100 | 95 | 84 |
| Rv0470A | - | 146 | 0 | 0 | 0 | 0 | 0 | 0 | 0 | 0 | 0 | 0 | 0 | 0 | 0 | 0 | 0 | 0 | 100 | 0 | 0 | 0 | 0 | 0 | 100 | 100 | 100 | 0 | 0 |
| Rv0470c | *pcaA* | 287 | 46 | 0 | 0 | 48 | 0 | 45 | 44 | 48 | 43 | 45 | 48 | 47 | 77 | 89 | 89 | 89 | 100 | 80 | 82 | 82 | 82 | 82 | 100 | 100 | 100 | 93 | 80 |
| Rv0471c | - | 162 | 0 | 0 | 0 | 0 | 0 | 0 | 0 | 0 | 0 | 0 | 0 | 0 | 0 | 0 | 0 | 0 | 100 | 0 | 0 | 0 | 0 | 0 | 100 | 100 | 100 | 0 | 0 |
| Rv0472c | - | 234 | 0 | 0 | 0 | 0 | 0 | 0 | 0 | 46 | 68 | 74 | 72 | 72 | 87 | 93 | 93 | 93 | 100 | 89 | 93 | 93 | 93 | 91 | 99 | 100 | 99 | 96 | 91 |
| Rv0473 | - | 456 | 60 | 66 | 63 | 64 | 64 | 65 | 65 | 59 | 76 | 78 | 79 | 77 | 83 | 92 | 90 | 94 | 100 | 82 | 87 | 87 | 87 | 89 | 100 | 100 | 100 | 90 | 88 |
| Rv0474 | - | 140 | 0 | 0 | 0 | 0 | 0 | 0 | 0 | 75 | 81 | 78 | 80 | 80 | 84 | 93 | 89 | 93 | 100 | 85 | 83 | 83 | 83 | 81 | 100 | 100 | 100 | 87 | 84 |
| Rv0475 | *hbhA* | 199 | 0 | 0 | 0 | 0 | 0 | 0 | 0 | 0 | 49 | 50 | 50 | 50 | 70 | 86 | 93 | 89 | 100 | 74 | 82 | 82 | 82 | 72 | 99 | 100 | 100 | 92 | 82 |
| Rv0476 | - | 87 | 0 | 0 | 0 | 0 | 0 | 0 | 0 | 63 | 68 | 67 | 67 | 67 | 68 | 93 | 93 | 93 | 100 | 83 | 75 | 75 | 75 | 74 | 100 | 100 | 100 | 91 | 83 |
| Rv0477 | - | 148 | 0 | 0 | 0 | 0 | 0 | 0 | 0 | 0 | 59 | 0 | 0 | 0 | 64 | 73 | 73 | 73 | 100 | 57 | 70 | 70 | 70 | 73 | 100 | 100 | 100 | 73 | 60 |
| Rv0478 | *deoC* | 224 | 71 | 71 | 67 | 65 | 61 | 73 | 70 | 49 | 76 | 76 | 76 | 77 | 79 | 88 | 86 | 88 | 100 | 79 | 76 | 76 | 76 | 80 | 100 | 100 | 100 | 86 | 81 |
| Rv0479c | - | 348 | 0 | 0 | 0 | 0 | 0 | 0 | 0 | 0 | 53 | 46 | 53 | 53 | 49 | 74 | 83 | 74 | 100 | 64 | 62 | 62 | 62 | 66 | 100 | 100 | 100 | 83 | 66 |
| Rv0480c | - | 280 | 56 | 59 | 60 | 61 | 53 | 55 | 54 | 56 | 58 | 53 | 74 | 67 | 72 | 86 | 84 | 85 | 100 | 73 | 76 | 76 | 76 | 76 | 100 | 100 | 100 | 83 | 74 |
| Rv0481c | - | 174 | 0 | 0 | 0 | 0 | 0 | 0 | 0 | 0 | 47 | 53 | 50 | 51 | 66 | 82 | 89 | 81 | 100 | 68 | 74 | 74 | 74 | 72 | 100 | 100 | 100 | 89 | 68 |
| Rv0482 | *murB* | 369 | 59 | 60 | 63 | 64 | 54 | 53 | 58 | 59 | 70 | 66 | 65 | 65 | 68 | 82 | 83 | 82 | 100 | 75 | 76 | 76 | 76 | 78 | 100 | 100 | 100 | 82 | 78 |
| Rv0483 | *lprQ* | 451 | 49 | 49 | 51 | 49 | 52 | 51 | 48 | 48 | 67 | 71 | 69 | 71 | 76 | 87 | 90 | 87 | 100 | 77 | 78 | 77 | 77 | 78 | 100 | 100 | 100 | 89 | 80 |
| Rv0484c | - | 251 | 69 | 71 | 71 | 71 | 65 | 73 | 62 | 74 | 82 | 87 | 87 | 86 | 87 | 94 | 93 | 94 | 100 | 86 | 88 | 88 | 88 | 89 | 100 | 100 | 100 | 92 | 86 |
| Rv0485 | - | 438 | 42 | 39 | 45 | 42 | 45 | 0 | 0 | 43 | 66 | 66 | 68 | 67 | 80 | 88 | 88 | 88 | 100 | 78 | 78 | 78 | 78 | 81 | 100 | 100 | 100 | 88 | 78 |
| Rv0486 | - | 480 | 65 | 69 | 67 | 67 | 63 | 67 | 63 | 70 | 75 | 80 | 79 | 76 | 82 | 92 | 85 | 92 | 100 | 78 | 81 | 81 | 81 | 83 | 100 | 100 | 100 | 85 | 81 |
| Rv0487 | - | 183 | 0 | 0 | 0 | 0 | 66 | 71 | 69 | 67 | 75 | 75 | 78 | 78 | 82 | 88 | 87 | 88 | 100 | 84 | 85 | 85 | 85 | 86 | 100 | 100 | 100 | 87 | 84 |
| Rv0488 | - | 201 | 50 | 50 | 54 | 55 | 0 | 0 | 0 | 63 | 63 | 67 | 65 | 66 | 69 | 0 | 0 | 0 | 100 | 72 | 73 | 73 | 73 | 72 | 100 | 100 | 100 | 0 | 73 |
| Rv0489 | *gpm1* | 249 | 80 | 81 | 79 | 79 | 83 | 80 | 81 | 80 | 83 | 84 | 86 | 85 | 88 | 94 | 95 | 94 | 100 | 90 | 89 | 90 | 90 | 89 | 100 | 100 | 100 | 95 | 89 |
| Rv0490 | *senX3* | 410 | 65 | 65 | 63 | 65 | 66 | 66 | 59 | 57 | 74 | 73 | 71 | 73 | 76 | 90 | 90 | 90 | 100 | 85 | 82 | 81 | 81 | 83 | 100 | 100 | 100 | 89 | 86 |
| Rv0491 | *regX3* | 227 | 86 | 89 | 86 | 86 | 84 | 84 | 86 | 84 | 91 | 94 | 93 | 93 | 96 | 99 | 99 | 99 | 100 | 96 | 96 | 96 | 96 | 96 | 100 | 100 | 100 | 99 | 97 |
| Rv0492A | - | 109 | 0 | 0 | 0 | 0 | 0 | 0 | 0 | 0 | 0 | 0 | 0 | 0 | 0 | 0 | 0 | 0 | 100 | 0 | 0 | 0 | 0 | 0 | 100 | 100 | 100 | 88 | 0 |
| Rv0492c | - | 629 | 0 | 0 | 0 | 0 | 0 | 0 | 41 | 39 | 47 | 0 | 37 | 41 | 37 | 0 | 89 | 39 | 99 | 0 | 0 | 0 | 0 | 40 | 100 | 100 | 100 | 88 | 0 |
| Rv0493c | - | 329 | 0 | 0 | 0 | 0 | 0 | 0 | 0 | 0 | 0 | 0 | 0 | 0 | 0 | 77 | 82 | 78 | 99 | 37 | 0 | 0 | 0 | 0 | 100 | 100 | 99 | 81 | 0 |
| Rv0494 | - | 242 | 0 | 0 | 0 | 0 | 0 | 0 | 0 | 47 | 45 | 48 | 50 | 51 | 49 | 43 | 89 | 50 | 100 | 46 | 47 | 47 | 47 | 45 | 100 | 100 | 100 | 89 | 50 |
| Rv0495c | - | 296 | 62 | 57 | 60 | 58 | 62 | 60 | 54 | 63 | 77 | 80 | 81 | 80 | 89 | 92 | 93 | 92 | 100 | 90 | 91 | 91 | 91 | 85 | 100 | 100 | 100 | 93 | 86 |
| Rv0496 | - | 328 | 75 | 46 | 78 | 79 | 76 | 80 | 73 | 66 | 88 | 85 | 86 | 86 | 90 | 92 | 92 | 92 | 100 | 88 | 91 | 91 | 91 | 92 | 100 | 100 | 100 | 92 | 93 |
| Rv0497 | - | 310 | 42 | 0 | 0 | 0 | 0 | 0 | 0 | 0 | 52 | 44 | 44 | 46 | 39 | 67 | 64 | 67 | 100 | 49 | 50 | 49 | 49 | 49 | 100 | 100 | 100 | 64 | 46 |
| Rv0498 | - | 280 | 0 | 0 | 0 | 0 | 0 | 0 | 0 | 62 | 73 | 79 | 78 | 78 | 87 | 95 | 96 | 95 | 100 | 88 | 90 | 90 | 90 | 88 | 100 | 100 | 100 | 96 | 89 |
| Rv0499 | - | 291 | 0 | 0 | 0 | 0 | 0 | 0 | 0 | 44 | 48 | 61 | 60 | 59 | 65 | 82 | 79 | 82 | 100 | 74 | 73 | 73 | 73 | 74 | 100 | 100 | 100 | 80 | 71 |
| Rv0500 | *proC* | 295 | 55 | 58 | 57 | 58 | 59 | 61 | 58 | 59 | 66 | 69 | 69 | 68 | 76 | 88 | 88 | 88 | 100 | 83 | 83 | 83 | 83 | 82 | 100 | 100 | 100 | 88 | 83 |
| Rv0500A | - | 78 | 78 | 78 | 86 | 94 | 77 | 84 | 77 | 80 | 81 | 79 | 78 | 78 | 79 | 87 | 85 | 87 | 100 | 76 | 84 | 84 | 84 | 87 | 100 | 100 | 100 | 85 | 77 |
| Rv0500B | - | 33 | 100 | 100 | 100 | 100 | 100 | 100 | 100 | 100 | 100 | 100 | 100 | 100 | 100 | 100 | 100 | 100 | 100 | 100 | 100 | 100 | 100 | 100 | 100 | 100 | 100 | 100 | 100 |
| Rv0501 | *galE2* | 376 | 0 | 0 | 0 | 0 | 0 | 0 | 0 | 65 | 70 | 75 | 74 | 74 | 84 | 91 | 94 | 91 | 100 | 85 | 88 | 89 | 89 | 85 | 100 | 100 | 100 | 94 | 86 |

| | | | | | | | | | | | | | | | | | | | | | | | | | | | | |
|---|---|---|---|---|---|---|---|---|---|---|---|---|---|---|---|---|---|---|---|---|---|---|---|---|---|---|---|---|---|
| Rv0502 | - | 358 | 0 | 0 | 0 | 0 | 0 | 43 | 0 | 63 | 76 | 69 | 71 | 72 | 78 | 95 | 92 | 95 | 100 | 85 | 87 | 87 | 87 | 85 | 100 | 100 | 100 | 92 | 87 |
| Rv0503c | *cmaA2* | 302 | 48 | 0 | 0 | 51 | 0 | 47 | 43 | 48 | 46 | 49 | 45 | 47 | 70 | 88 | 90 | 88 | 100 | 73 | 73 | 73 | 73 | 76 | 100 | 100 | 100 | 89 | 73 |
| Rv0504c | - | 166 | 0 | 0 | 0 | 0 | 0 | 0 | 0 | 0 | 57 | 60 | 58 | 58 | 74 | 93 | 89 | 93 | 100 | 77 | 81 | 81 | 81 | 79 | 100 | 100 | 100 | 89 | 77 |
| Rv0505c | *serB1* | 373 | 75 | 73 | 72 | 74 | 74 | 0 | 69 | 72 | 87 | 76 | 86 | 86 | 76 | 83 | 83 | 83 | 100 | 84 | 88 | 88 | 88 | 79 | 100 | 100 | 100 | 90 | 78 |
| Rv0506 | *mmpS2* | 147 | 0 | 0 | 0 | 0 | 0 | 0 | 0 | 0 | 0 | 0 | 0 | 0 | 57 | 65 | 82 | 60 | 100 | 55 | 61 | 61 | 61 | 58 | 100 | 100 | 100 | 57 | 58 |
| Rv0507 | *mmpL2* | 968 | 44 | 43 | 41 | 54 | 44 | 57 | 45 | 50 | 54 | 57 | 54 | 53 | 74 | 79 | 93 | 75 | 99 | 76 | 75 | 75 | 75 | 76 | 99 | 100 | 99 | 74 | 76 |
| Rv0508 | - | 97 | 56 | 62 | 61 | 0 | 0 | 57 | 57 | 56 | 76 | 74 | 72 | 75 | 75 | 85 | 86 | 85 | 100 | 59 | 80 | 80 | 80 | 75 | 100 | 100 | 100 | 86 | 81 |
| Rv0509 | *hemA* | 468 | 62 | 64 | 63 | 63 | 62 | 60 | 65 | 59 | 80 | 80 | 82 | 82 | 83 | 91 | 91 | 91 | 100 | 85 | 86 | 86 | 86 | 86 | 100 | 100 | 100 | 91 | 86 |
| Rv0510 | *hemC* | 309 | 69 | 68 | 67 | 67 | 60 | 61 | 61 | 69 | 84 | 84 | 85 | 85 | 88 | 93 | 96 | 93 | 100 | 90 | 91 | 91 | 91 | 90 | 100 | 100 | 100 | 95 | 90 |
| Rv0511 | *hemD* | 565 | 65 | 63 | 67 | 68 | 64 | 67 | 65 | 60 | 78 | 79 | 78 | 78 | 84 | 93 | 93 | 93 | 100 | 86 | 88 | 88 | 88 | 87 | 100 | 100 | 100 | 93 | 86 |
| Rv0512 | *hemB* | 329 | 79 | 77 | 77 | 76 | 77 | 75 | 78 | 79 | 84 | 85 | 84 | 83 | 86 | 94 | 93 | 94 | 99 | 85 | 91 | 91 | 91 | 91 | 100 | 100 | 100 | 93 | 88 |
| Rv0513 | - | 182 | 0 | 0 | 0 | 0 | 0 | 0 | 0 | 0 | 62 | 60 | 65 | 64 | 63 | 82 | 83 | 81 | 100 | 75 | 75 | 75 | 75 | 76 | 100 | 100 | 100 | 81 | 75 |
| Rv0514 | - | 99 | 0 | 0 | 0 | 0 | 0 | 0 | 0 | 0 | 0 | 0 | 0 | 0 | 0 | 76 | 73 | 76 | 100 | 63 | 58 | 58 | 58 | 63 | 100 | 100 | 100 | 73 | 63 |
| Rv0515 | - | 503 | 41 | 51 | 53 | 50 | 49 | 0 | 0 | 42 | 0 | 47 | 42 | 41 | 53 | 75 | 75 | 75 | 100 | 54 | 68 | 69 | 69 | 58 | 100 | 100 | 100 | 63 | 53 |
| Rv0516c | - | 158 | 0 | 0 | 0 | 0 | 0 | 0 | 0 | 0 | 0 | 0 | 0 | 0 | 0 | 70 | 70 | 70 | 100 | 61 | 60 | 60 | 60 | 58 | 100 | 100 | 100 | 71 | 60 |
| Rv0517 | - | 436 | 41 | 0 | 41 | 0 | 42 | 0 | 42 | 40 | 40 | 0 | 0 | 0 | 83 | 86 | 86 | 86 | 99 | 80 | 77 | 77 | 77 | 79 | 100 | 100 | 100 | 86 | 79 |
| Rv0518 | - | 231 | 0 | 0 | 0 | 0 | 0 | 0 | 0 | 0 | 0 | 0 | 0 | 0 | 72 | 81 | 81 | 81 | 100 | 64 | 72 | 72 | 72 | 68 | 100 | 100 | 100 | 77 | 71 |
| Rv0519c | - | 300 | 0 | 0 | 0 | 0 | 0 | 0 | 0 | 0 | 0 | 0 | 0 | 0 | 68 | 86 | 86 | 86 | 100 | 66 | 72 | 72 | 72 | 72 | 100 | 100 | 100 | 90 | 67 |
| Rv0520 | - | 116 | 0 | 0 | 0 | 0 | 0 | 0 | 0 | 0 | 0 | 0 | 0 | 0 | 0 | 0 | 88 | 0 | 100 | 0 | 0 | 0 | 0 | 0 | 100 | 100 | 100 | 88 | 0 |
| Rv0521 | - | 101 | 0 | 0 | 0 | 0 | 0 | 0 | 0 | 0 | 0 | 0 | 0 | 0 | 0 | 0 | 0 | 0 | 100 | 0 | 0 | 0 | 0 | 0 | 100 | 100 | 100 | 68 | 0 |
| Rv0522 | *gabP* | 434 | 60 | 60 | 60 | 61 | 59 | 59 | 58 | 42 | 58 | 71 | 78 | 79 | 78 | 86 | 87 | 86 | 100 | 80 | 78 | 78 | 78 | 76 | 100 | 100 | 100 | 67 | 41 |
| Rv0523c | - | 131 | 0 | 0 | 0 | 0 | 0 | 0 | 0 | 0 | 57 | 50 | 60 | 64 | 76 | 89 | 57 | 89 | 100 | 64 | 63 | 63 | 63 | 76 | 100 | 100 | 100 | 0 | 80 |
| Rv0524 | *hemL* | 462 | 71 | 71 | 71 | 73 | 72 | 70 | 71 | 68 | 81 | 77 | 80 | 79 | 78 | 87 | 88 | 88 | 100 | 86 | 85 | 85 | 85 | 84 | 100 | 100 | 100 | 88 | 86 |
| Rv0525 | - | 202 | 65 | 63 | 65 | 63 | 66 | 61 | 66 | 66 | 81 | 77 | 76 | 76 | 79 | 92 | 94 | 92 | 100 | 71 | 75 | 75 | 75 | 76 | 99 | 100 | 100 | 94 | 72 |
| Rv0526 | - | 216 | 61 | 66 | 59 | 61 | 63 | 60 | 62 | 52 | 75 | 81 | 81 | 81 | 79 | 89 | 89 | 89 | 100 | 86 | 83 | 83 | 83 | 86 | 100 | 100 | 100 | 89 | 82 |
| Rv0527 | *ccdA* | 259 | 70 | 70 | 72 | 72 | 75 | 73 | 74 | 60 | 75 | 78 | 77 | 77 | 82 | 92 | 92 | 92 | 100 | 88 | 88 | 88 | 88 | 88 | 100 | 100 | 100 | 91 | 85 |
| Rv0528 | - | 529 | 63 | 67 | 65 | 65 | 67 | 66 | 65 | 53 | 74 | 77 | 75 | 75 | 80 | 89 | 90 | 89 | 100 | 82 | 82 | 82 | 82 | 86 | 99 | 100 | 100 | 90 | 83 |
| Rv0529 | *ccsA* | 324 | 59 | 63 | 60 | 65 | 57 | 72 | 58 | 58 | 72 | 75 | 73 | 73 | 78 | 89 | 89 | 89 | 100 | 84 | 84 | 84 | 84 | 85 | 100 | 100 | 100 | 89 | 85 |
| Rv0530 | - | 405 | 0 | 0 | 0 | 0 | 0 | 0 | 0 | 41 | 65 | 71 | 73 | 67 | 72 | 75 | 81 | 75 | 100 | 76 | 76 | 76 | 76 | 73 | 99 | 100 | 100 | 76 | 77 |
| Rv0531 | - | 105 | 0 | 0 | 65 | 0 | 0 | 64 | 0 | 0 | 67 | 72 | 69 | 70 | 75 | 77 | 87 | 77 | 100 | 79 | 80 | 80 | 80 | 74 | 100 | 100 | 100 | 87 | 78 |
| Rv0532 | *PE_PGRS6* | 594 | 46 | 35 | 36 | 34 | 37 | 38 | 37 | 36 | 41 | 42 | 47 | 45 | 43 | 45 | 56 | 35 | 92 | 47 | 47 | 45 | 45 | 48 | 95 | 99 | 95 | 59 | 44 |
| Rv0533c | *fabH* | 335 | 0 | 0 | 0 | 0 | 0 | 0 | 0 | 64 | 68 | 70 | 71 | 70 | 86 | 87 | 90 | 87 | 100 | 40 | 41 | 41 | 41 | 42 | 100 | 100 | 100 | 90 | 0 |
| Rv0534c | *menA* | 292 | 68 | 71 | 72 | 71 | 71 | 68 | 71 | 69 | 82 | 80 | 79 | 80 | 79 | 89 | 89 | 89 | 100 | 86 | 88 | 88 | 88 | 88 | 100 | 100 | 100 | 89 | 88 |
| Rv0535 | *pnp* | 264 | 0 | 0 | 0 | 0 | 0 | 0 | 0 | 67 | 74 | 0 | 75 | 75 | 78 | 84 | 85 | 85 | 100 | 0 | 84 | 84 | 84 | 85 | 100 | 100 | 100 | 88 | 85 |
| Rv0536 | *galE3* | 346 | 41 | 43 | 42 | 39 | 40 | 42 | 41 | 0 | 42 | 41 | 72 | 73 | 48 | 92 | 92 | 92 | 100 | 42 | 84 | 84 | 84 | 82 | 100 | 100 | 100 | 91 | 84 |
| Rv0537c | - | 477 | 0 | 0 | 0 | 0 | 0 | 0 | 0 | 0 | 0 | 0 | 0 | 0 | 0 | 66 | 70 | 66 | 100 | 0 | 58 | 0 | 0 | 58 | 100 | 100 | 100 | 69 | 0 |
| Rv0538 | - | 548 | 39 | 38 | 41 | 35 | 32 | 34 | 30 | 34 | 42 | 47 | 43 | 36 | 38 | 60 | 71 | 61 | 99 | 42 | 46 | 40 | 40 | 51 | 99 | 100 | 99 | 71 | 44 |
| Rv0539 | - | 210 | 45 | 47 | 48 | 49 | 0 | 0 | 49 | 47 | 45 | 0 | 76 | 77 | 48 | 90 | 92 | 90 | 100 | 50 | 86 | 86 | 86 | 86 | 100 | 100 | 100 | 92 | 80 |

| | | | | | | | | | | | | | | | | | | | | | | | | | | | | | |
|---|---|---|---|---|---|---|---|---|---|---|---|---|---|---|---|---|---|---|---|---|---|---|---|---|---|---|---|---|---|
| Rv0540 | - | 220 | 0 | 0 | 0 | 0 | 0 | 0 | 0 | 0 | 0 | 0 | 64 | 62 | 0 | 83 | 82 | 82 | 100 | 0 | 71 | 72 | 72 | 74 | 100 | 100 | 100 | 82 | 71 |
| Rv0541c | - | 449 | 0 | 0 | 0 | 0 | 0 | 0 | 0 | 0 | 0 | 0 | 71 | 68 | 0 | 84 | 86 | 84 | 100 | 0 | 79 | 79 | 79 | 76 | 100 | 100 | 100 | 85 | 76 |
| Rv0542c | *menE* | 362 | 54 | 58 | 57 | 54 | 49 | 52 | 50 | 54 | 69 | 71 | 70 | 70 | 70 | 85 | 86 | 84 | 100 | 75 | 76 | 76 | 76 | 77 | 100 | 100 | 100 | 85 | 77 |
| Rv0543c | - | 100 | 0 | 0 | 0 | 57 | 0 | 0 | 0 | 0 | 70 | 68 | 70 | 68 | 77 | 88 | 88 | 88 | 100 | 70 | 69 | 69 | 69 | 73 | 100 | 100 | 100 | 90 | 72 |
| Rv0544c | - | 92 | 0 | 0 | 0 | 0 | 0 | 60 | 0 | 0 | 0 | 0 | 0 | 0 | 0 | 80 | 80 | 80 | 100 | 0 | 0 | 0 | 0 | 0 | 100 | 100 | 100 | 73 | 0 |
| Rv0545c | *pitA* | 417 | 54 | 56 | 76 | 75 | 69 | 73 | 54 | 67 | 79 | 82 | 79 | 78 | 70 | 87 | 90 | 87 | 99 | 70 | 72 | 72 | 72 | 72 | 99 | 100 | 99 | 87 | 71 |
| Rv0546c | - | 128 | 0 | 0 | 0 | 0 | 0 | 0 | 0 | 0 | 73 | 78 | 74 | 74 | 82 | 90 | 88 | 90 | 100 | 83 | 81 | 81 | 81 | 79 | 100 | 100 | 100 | 88 | 83 |
| Rv0547c | - | 294 | 48 | 50 | 45 | 51 | 44 | 50 | 47 | 62 | 54 | 60 | 63 | 63 | 73 | 88 | 89 | 88 | 100 | 80 | 80 | 80 | 80 | 82 | 100 | 100 | 100 | 89 | 80 |
| Rv0548c | *menB* | 314 | 81 | 86 | 80 | 82 | 82 | 84 | 83 | 85 | 91 | 90 | 90 | 90 | 88 | 96 | 96 | 96 | 100 | 92 | 90 | 90 | 90 | 90 | 100 | 100 | 100 | 96 | 90 |
| Rv0549c | - | 137 | 47 | 0 | 0 | 0 | 0 | 0 | 0 | 0 | 0 | 0 | 0 | 0 | 0 | 0 | 0 | 0 | 100 | 47 | 0 | 0 | 0 | 0 | 100 | 100 | 100 | 0 | 0 |
| Rv0550c | - | 88 | 0 | 0 | 0 | 0 | 0 | 0 | 0 | 0 | 0 | 0 | 0 | 0 | 0 | 0 | 0 | 0 | 100 | 0 | 0 | 0 | 0 | 0 | 100 | 100 | 100 | 0 | 0 |
| Rv0551c | *fadD8* | 571 | 42 | 41 | 41 | 47 | 44 | 45 | 44 | 83 | 48 | 69 | 69 | 69 | 83 | 90 | 92 | 90 | 100 | 86 | 90 | 90 | 90 | 89 | 100 | 100 | 100 | 91 | 92 |
| Rv0552 | - | 534 | 42 | 0 | 0 | 0 | 0 | 0 | 0 | 43 | 66 | 73 | 72 | 72 | 62 | 88 | 91 | 89 | 100 | 55 | 85 | 85 | 85 | 84 | 100 | 100 | 100 | 90 | 55 |
| Rv0553 | *menC* | 326 | 66 | 65 | 66 | 66 | 61 | 63 | 55 | 63 | 64 | 72 | 75 | 76 | 80 | 87 | 87 | 88 | 100 | 80 | 84 | 83 | 83 | 85 | 100 | 100 | 100 | 87 | 83 |
| Rv0554 | *bpoC* | 262 | 0 | 0 | 0 | 42 | 0 | 0 | 0 | 0 | 61 | 59 | 60 | 61 | 73 | 89 | 92 | 90 | 100 | 82 | 82 | 82 | 82 | 82 | 100 | 100 | 100 | 91 | 82 |
| Rv0555 | *menD* | 554 | 51 | 53 | 55 | 55 | 57 | 52 | 51 | 53 | 77 | 77 | 76 | 76 | 85 | 91 | 92 | 91 | 100 | 86 | 88 | 88 | 88 | 86 | 100 | 100 | 100 | 92 | 87 |
| Rv0556 | - | 171 | 56 | 55 | 53 | 55 | 51 | 52 | 56 | 0 | 68 | 74 | 67 | 66 | 84 | 86 | 91 | 87 | 99 | 83 | 81 | 81 | 81 | 82 | 99 | 100 | 99 | 88 | 75 |
| Rv0557 | *pimB* | 378 | 66 | 54 | 64 | 65 | 63 | 69 | 64 | 61 | 72 | 76 | 74 | 74 | 77 | 92 | 90 | 92 | 100 | 86 | 85 | 85 | 85 | 85 | 100 | 100 | 100 | 89 | 86 |
| Rv0558 | *ubiE* | 234 | 77 | 76 | 79 | 79 | 76 | 75 | 78 | 72 | 83 | 81 | 82 | 82 | 85 | 94 | 93 | 94 | 100 | 87 | 86 | 86 | 86 | 89 | 100 | 100 | 100 | 93 | 88 |
| Rv0559c | - | 112 | 0 | 0 | 0 | 0 | 0 | 0 | 0 | 0 | 0 | 0 | 0 | 0 | 64 | 66 | 90 | 66 | 100 | 66 | 70 | 70 | 70 | 70 | 100 | 100 | 100 | 90 | 69 |
| Rv0560c | - | 241 | 0 | 0 | 0 | 0 | 0 | 0 | 0 | 0 | 0 | 0 | 69 | 70 | 77 | 78 | 79 | 78 | 100 | 69 | 66 | 66 | 66 | 75 | 100 | 100 | 100 | 76 | 69 |
| Rv0561c | - | 408 | 67 | 66 | 62 | 64 | 54 | 0 | 61 | 73 | 73 | 75 | 77 | 78 | 80 | 87 | 94 | 87 | 100 | 80 | 86 | 86 | 86 | 82 | 100 | 100 | 100 | 93 | 81 |
| Rv0562 | *grcC1* | 335 | 66 | 65 | 65 | 65 | 67 | 64 | 66 | 62 | 75 | 76 | 76 | 76 | 80 | 86 | 89 | 85 | 100 | 85 | 87 | 87 | 87 | 86 | 100 | 100 | 100 | 89 | 84 |
| Rv0563 | *htpX* | 286 | 0 | 0 | 0 | 0 | 44 | 0 | 0 | 56 | 84 | 87 | 87 | 88 | 88 | 95 | 97 | 95 | 100 | 91 | 94 | 94 | 94 | 93 | 100 | 100 | 100 | 96 | 91 |
| Rv0564c | *gpsA* | 341 | 57 | 57 | 60 | 60 | 59 | 55 | 59 | 55 | 53 | 67 | 69 | 69 | 86 | 92 | 95 | 95 | 100 | 92 | 93 | 93 | 93 | 92 | 100 | 100 | 100 | 95 | 91 |
| Rv0565c | - | 486 | 43 | 0 | 0 | 0 | 54 | 0 | 0 | 66 | 64 | 66 | 68 | 67 | 66 | 86 | 87 | 86 | 100 | 78 | 80 | 80 | 80 | 80 | 99 | 100 | 100 | 65 | 78 |
| Rv0566c | - | 163 | 0 | 0 | 0 | 0 | 0 | 0 | 0 | 83 | 86 | 84 | 85 | 86 | 94 | 96 | 96 | 96 | 100 | 92 | 93 | 93 | 93 | 93 | 100 | 100 | 100 | 96 | 95 |
| Rv0567 | - | 339 | 0 | 0 | 0 | 0 | 0 | 0 | 0 | 0 | 0 | 0 | 43 | 43 | 0 | 0 | 88 | 0 | 99 | 44 | 45 | 45 | 45 | 43 | 100 | 100 | 100 | 88 | 42 |
| Rv0568 | *cyp135B1* | 472 | 0 | 0 | 0 | 0 | 0 | 0 | 0 | 58 | 50 | 42 | 45 | 44 | 79 | 52 | 52 | 52 | 100 | 78 | 84 | 85 | 85 | 79 | 100 | 100 | 100 | 50 | 82 |
| Rv0569 | - | 88 | 0 | 0 | 0 | 0 | 0 | 0 | 0 | 62 | 0 | 0 | 0 | 0 | 0 | 0 | 0 | 0 | 100 | 0 | 88 | 88 | 88 | 71 | 100 | 100 | 100 | 0 | 0 |
| Rv0570 | *nrdZ* | 692 | 38 | 41 | 40 | 40 | 40 | 38 | 41 | 47 | 41 | 40 | 39 | 40 | 39 | 39 | 39 | 39 | 100 | 39 | 87 | 87 | 87 | 39 | 99 | 100 | 100 | 39 | 88 |
| Rv0571c | - | 443 | 0 | 0 | 0 | 0 | 0 | 0 | 0 | 63 | 63 | 66 | 66 | 66 | 56 | 60 | 78 | 60 | 100 | 58 | 62 | 62 | 62 | 64 | 100 | 100 | 100 | 77 | 81 |
| Rv0572c | - | 113 | 0 | 0 | 0 | 0 | 0 | 0 | 0 | 0 | 0 | 0 | 0 | 0 | 0 | 0 | 0 | 0 | 99 | 0 | 0 | 0 | 0 | 0 | 99 | 100 | 99 | 0 | 84 |
| Rv0573c | - | 463 | 51 | 53 | 53 | 52 | 53 | 52 | 51 | 54 | 50 | 51 | 51 | 52 | 51 | 52 | 52 | 52 | 100 | 53 | 53 | 53 | 53 | 54 | 100 | 100 | 100 | 53 | 53 |
| Rv0574c | - | 380 | 0 | 0 | 0 | 0 | 0 | 0 | 0 | 41 | 0 | 0 | 67 | 0 | 0 | 0 | 0 | 0 | 99 | 0 | 0 | 0 | 0 | 0 | 100 | 100 | 100 | 0 | 0 |
| Rv0575c | - | 388 | 0 | 0 | 0 | 41 | 0 | 0 | 0 | 0 | 44 | 0 | 50 | 52 | 51 | 51 | 88 | 51 | 100 | 0 | 48 | 48 | 48 | 71 | 100 | 100 | 100 | 49 | 53 |
| Rv0576 | - | 434 | 0 | 0 | 0 | 0 | 0 | 0 | 0 | 0 | 55 | 0 | 75 | 76 | 58 | 60 | 85 | 59 | 99 | 0 | 66 | 66 | 66 | 59 | 100 | 100 | 100 | 84 | 62 |
| Rv0577 | *TB27.3* | 261 | 44 | 43 | 44 | 45 | 45 | 39 | 45 | 47 | 46 | 54 | 74 | 75 | 50 | 0 | 0 | 0 | 100 | 46 | 0 | 0 | 0 | 46 | 100 | 100 | 100 | 43 | 47 |

| | | | | | | | | | | | | | | | | | | | | | | | | | | | | | |
|---|---|---|---|---|---|---|---|---|---|---|---|---|---|---|---|---|---|---|---|---|---|---|---|---|---|---|---|---|---|
| Rv0578c | *PE_PGRS7* | 1306 | 44 | 38 | 36 | 34 | 40 | 39 | 38 | 32 | 37 | 38 | 38 | 37 | 38 | 31 | 68 | 31 | 99 | 52 | 54 | 54 | 54 | 48 | 99 | 99 | 99 | 57 | 50 |
| Rv0579 | - | 252 | 0 | 0 | 0 | 0 | 0 | 0 | 0 | 0 | 72 | 0 | 65 | 64 | 0 | 85 | 86 | 84 | 100 | 0 | 0 | 0 | 0 | 0 | 100 | 100 | 100 | 85 | 82 |
| Rv0580c | - | 163 | 0 | 0 | 0 | 0 | 0 | 0 | 0 | 0 | 0 | 0 | 47 | 0 | 0 | 0 | 84 | 0 | 100 | 71 | 70 | 70 | 70 | 0 | 100 | 100 | 100 | 85 | 71 |
| Rv0581 | - | 71 | 0 | 0 | 0 | 0 | 0 | 0 | 0 | 0 | 0 | 0 | 0 | 0 | 0 | 0 | 0 | 0 | 100 | 0 | 0 | 0 | 0 | 0 | 100 | 100 | 100 | 0 | 0 |
| Rv0582 | - | 135 | 0 | 0 | 0 | 0 | 0 | 0 | 0 | 0 | 54 | 0 | 0 | 0 | 0 | 0 | 0 | 0 | 100 | 0 | 0 | 0 | 0 | 0 | 100 | 100 | 100 | 0 | 0 |
| Rv0583c | *lpqN* | 228 | 0 | 0 | 0 | 0 | 0 | 0 | 0 | 0 | 0 | 0 | 0 | 0 | 43 | 79 | 81 | 77 | 100 | 62 | 65 | 66 | 66 | 67 | 100 | 100 | 100 | 80 | 64 |
| Rv0584 | - | 877 | 0 | 0 | 0 | 0 | 0 | 0 | 0 | 0 | 0 | 0 | 0 | 0 | 0 | 78 | 79 | 79 | 99 | 0 | 0 | 0 | 0 | 0 | 99 | 100 | 100 | 81 | 0 |
| Rv0585c | - | 795 | 43 | 0 | 0 | 0 | 40 | 0 | 0 | 0 | 79 | 76 | 0 | 0 | 0 | 86 | 88 | 86 | 100 | 84 | 83 | 82 | 82 | 85 | 99 | 100 | 100 | 88 | 85 |
| Rv0586 | - | 240 | 0 | 0 | 0 | 0 | 0 | 0 | 0 | 70 | 71 | 67 | 66 | 66 | 61 | 78 | 47 | 80 | 100 | 45 | 75 | 75 | 75 | 73 | 100 | 100 | 100 | 47 | 78 |
| Rv0587 | *yrbE2A* | 265 | 0 | 0 | 0 | 0 | 0 | 0 | 0 | 67 | 79 | 79 | 77 | 84 | 76 | 95 | 89 | 95 | 100 | 88 | 90 | 90 | 90 | 87 | 100 | 100 | 100 | 89 | 89 |
| Rv0588 | *yrbE2B* | 295 | 0 | 0 | 0 | 0 | 0 | 0 | 0 | 68 | 72 | 74 | 71 | 78 | 74 | 93 | 94 | 94 | 100 | 84 | 83 | 83 | 83 | 82 | 99 | 100 | 100 | 85 | 86 |
| Rv0589 | *mce2A* | 404 | 0 | 0 | 0 | 0 | 0 | 0 | 0 | 46 | 53 | 51 | 54 | 54 | 52 | 89 | 75 | 89 | 99 | 82 | 77 | 78 | 78 | 76 | 99 | 100 | 99 | 74 | 80 |
| Rv0590 | *mce2B* | 275 | 0 | 0 | 0 | 0 | 0 | 0 | 0 | 56 | 61 | 66 | 64 | 67 | 62 | 87 | 80 | 87 | 100 | 81 | 83 | 83 | 83 | 81 | 100 | 100 | 100 | 80 | 81 |
| Rv0590A | - | 84 | 0 | 0 | 0 | 0 | 0 | 0 | 0 | 0 | 0 | 61 | 0 | 61 | 0 | 91 | 77 | 90 | 100 | 83 | 86 | 86 | 86 | 82 | 100 | 100 | 100 | 77 | 82 |
| Rv0591 | *mce2C* | 481 | 0 | 0 | 37 | 0 | 0 | 0 | 0 | 54 | 50 | 54 | 54 | 56 | 52 | 84 | 80 | 84 | 99 | 74 | 75 | 75 | 75 | 77 | 99 | 100 | 100 | 80 | 76 |
| Rv0592 | *mce2D* | 508 | 0 | 0 | 0 | 0 | 0 | 0 | 0 | 54 | 55 | 52 | 58 | 62 | 49 | 88 | 78 | 88 | 93 | 81 | 82 | 82 | 82 | 79 | 100 | 100 | 100 | 78 | 81 |
| Rv0593 | *lprL* | 402 | 0 | 0 | 0 | 0 | 0 | 0 | 0 | 51 | 52 | 55 | 56 | 58 | 54 | 86 | 75 | 86 | 99 | 81 | 81 | 81 | 81 | 77 | 100 | 100 | 100 | 74 | 80 |
| Rv0594 | *mce2F* | 516 | 0 | 0 | 0 | 0 | 0 | 0 | 0 | 49 | 53 | 54 | 54 | 60 | 52 | 89 | 80 | 89 | 100 | 77 | 81 | 81 | 81 | 77 | 100 | 100 | 100 | 80 | 76 |
| Rv0595c | - | 130 | 0 | 0 | 0 | 0 | 0 | 0 | 0 | 0 | 0 | 0 | 0 | 0 | 0 | 55 | 0 | 0 | 100 | 0 | 0 | 0 | 0 | 0 | 100 | 100 | 100 | 0 | 0 |
| Rv0596c | - | 85 | 0 | 0 | 0 | 0 | 0 | 0 | 0 | 0 | 0 | 0 | 0 | 0 | 0 | 0 | 0 | 0 | 100 | 0 | 0 | 0 | 0 | 0 | 100 | 100 | 100 | 0 | 0 |
| Rv0597c | - | 411 | 55 | 43 | 0 | 0 | 0 | 0 | 0 | 42 | 62 | 0 | 0 | 0 | 0 | 0 | 0 | 0 | 100 | 0 | 0 | 0 | 0 | 0 | 100 | 100 | 100 | 0 | 0 |
| Rv0598c | - | 137 | 0 | 0 | 0 | 0 | 0 | 0 | 0 | 0 | 0 | 0 | 0 | 0 | 0 | 0 | 0 | 0 | 100 | 0 | 59 | 61 | 61 | 0 | 100 | 100 | 100 | 0 | 0 |
| Rv0599c | - | 78 | 0 | 0 | 0 | 0 | 0 | 0 | 0 | 0 | 0 | 0 | 0 | 0 | 0 | 0 | 0 | 0 | 100 | 77 | 82 | 82 | 82 | 0 | 100 | 100 | 100 | 0 | 0 |
| Rv0600c | - | 168 | 48 | 47 | 51 | 47 | 51 | 53 | 49 | 50 | 51 | 50 | 51 | 51 | 49 | 51 | 51 | 51 | 100 | 47 | 54 | 54 | 54 | 48 | 100 | 100 | 100 | 50 | 52 |
| Rv0601c | - | 156 | 59 | 53 | 54 | 54 | 0 | 0 | 53 | 61 | 54 | 60 | 66 | 53 | 52 | 61 | 55 | 61 | 100 | 54 | 55 | 55 | 55 | 53 | 100 | 100 | 100 | 55 | 57 |
| Rv0602c | *tcrA* | 253 | 56 | 56 | 60 | 57 | 55 | 58 | 60 | 64 | 59 | 63 | 64 | 60 | 60 | 64 | 64 | 64 | 100 | 61 | 59 | 59 | 59 | 58 | 100 | 100 | 100 | 62 | 60 |
| Rv0603 | - | 103 | 0 | 0 | 0 | 0 | 0 | 0 | 0 | 55 | 0 | 0 | 0 | 0 | 0 | 0 | 0 | 0 | 99 | 0 | 0 | 0 | 0 | 0 | 100 | 100 | 100 | 0 | 0 |
| Rv0604 | *lpqO* | 316 | 0 | 0 | 0 | 0 | 0 | 0 | 0 | 0 | 0 | 0 | 0 | 0 | 0 | 0 | 0 | 0 | 99 | 0 | 0 | 0 | 0 | 0 | 100 | 100 | 100 | 0 | 0 |
| Rv0605 | - | 202 | 0 | 0 | 0 | 0 | 0 | 0 | 0 | 0 | 0 | 0 | 0 | 0 | 0 | 0 | 0 | 0 | 100 | 0 | 0 | 0 | 0 | 0 | 100 | 100 | 100 | 0 | 0 |
| Rv0606 | - | 247 | 0 | 0 | 0 | 0 | 0 | 0 | 0 | 44 | 0 | 0 | 54 | 0 | 0 | 0 | 0 | 0 | 100 | 0 | 0 | 0 | 0 | 0 | 100 | 100 | 100 | 0 | 0 |
| Rv0607 | - | 128 | 0 | 0 | 0 | 0 | 0 | 0 | 0 | 0 | 0 | 0 | 0 | 0 | 0 | 0 | 0 | 0 | 100 | 59 | 0 | 0 | 0 | 0 | 100 | 100 | 100 | 0 | 56 |
| Rv0608 | - | 81 | 0 | 0 | 0 | 0 | 0 | 0 | 0 | 0 | 0 | 0 | 0 | 0 | 0 | 0 | 0 | 0 | 100 | 0 | 0 | 0 | 0 | 0 | 100 | 100 | 100 | 0 | 81 |
| Rv0609 | - | 133 | 0 | 0 | 0 | 0 | 0 | 0 | 0 | 0 | 0 | 0 | 0 | 0 | 0 | 0 | 0 | 0 | 100 | 0 | 0 | 0 | 0 | 60 | 100 | 100 | 100 | 0 | 91 |
| Rv0609A | - | 75 | 0 | 0 | 0 | 0 | 0 | 0 | 0 | 0 | 0 | 0 | 0 | 0 | 0 | 0 | 0 | 0 | 100 | 0 | 0 | 0 | 0 | 0 | 100 | 100 | 100 | 0 | 0 |
| Rv0610c | - | 385 | 0 | 0 | 0 | 0 | 0 | 0 | 0 | 0 | 0 | 0 | 0 | 0 | 0 | 0 | 0 | 0 | 100 | 0 | 0 | 0 | 0 | 0 | 100 | 100 | 100 | 0 | 0 |
| Rv0611c | - | 127 | 0 | 0 | 0 | 0 | 0 | 0 | 0 | 0 | 0 | 0 | 0 | 0 | 0 | 0 | 0 | 0 | 100 | 0 | 0 | 0 | 0 | 0 | 100 | 100 | 100 | 0 | 0 |
| Rv0612 | - | 201 | 0 | 0 | 0 | 0 | 0 | 0 | 0 | 0 | 0 | 0 | 0 | 0 | 0 | 0 | 0 | 0 | 100 | 0 | 0 | 0 | 0 | 0 | 100 | 100 | 100 | 0 | 0 |
| Rv0613c | - | 855 | 0 | 0 | 0 | 0 | 0 | 0 | 0 | 0 | 0 | 49 | 71 | 48 | 0 | 0 | 0 | 0 | 100 | 0 | 0 | 0 | 0 | 68 | 100 | 100 | 99 | 0 | 72 |

| | | | | | | | | | | | | | | | | | | | | | | | | | | | | |
|---|---|---|---|---|---|---|---|---|---|---|---|---|---|---|---|---|---|---|---|---|---|---|---|---|---|---|---|---|
| Rv0614 | - | 330 | 0 | 0 | 0 | 0 | 0 | 0 | 0 | 0 | 0 | 0 | 0 | 0 | 0 | 77 | 52 | 52 | 100 | 0 | 0 | 0 | 0 | 0 | 100 | 100 | 100 | 61 | 0 |
| Rv0615 | - | 80 | 0 | 0 | 0 | 0 | 0 | 0 | 0 | 0 | 0 | 0 | 0 | 0 | 0 | 0 | 61 | 0 | 100 | 0 | 0 | 0 | 0 | 0 | 100 | 100 | 100 | 0 | 0 |
| Rv0616c | - | 88 | 0 | 0 | 0 | 0 | 0 | 0 | 0 | 0 | 0 | 0 | 0 | 0 | 0 | 0 | 0 | 0 | 100 | 0 | 0 | 0 | 0 | 0 | 100 | 100 | 100 | 0 | 0 |
| Rv0617 | - | 133 | 0 | 0 | 0 | 0 | 0 | 0 | 0 | 0 | 0 | 0 | 0 | 0 | 0 | 0 | 0 | 0 | 100 | 0 | 0 | 0 | 0 | 0 | 100 | 100 | 100 | 0 | 0 |
| Rv0618 | *galTa* | 231 | 0 | 57 | 0 | 0 | 61 | 57 | 0 | 0 | 63 | 61 | 63 | 65 | 61 | 81 | 83 | 82 | 99 | 0 | 0 | 0 | 0 | 80 | 100 | 100 | 100 | 83 | 0 |
| Rv0619 | *galTb* | 181 | 0 | 57 | 0 | 0 | 65 | 58 | 0 | 0 | 64 | 71 | 68 | 68 | 69 | 85 | 85 | 85 | 99 | 0 | 0 | 0 | 0 | 81 | 99 | 100 | 100 | 85 | 0 |
| Rv0620 | *galK* | 363 | 0 | 51 | 0 | 0 | 54 | 49 | 0 | 54 | 56 | 56 | 56 | 56 | 56 | 79 | 79 | 79 | 99 | 0 | 0 | 0 | 0 | 77 | 100 | 100 | 100 | 78 | 0 |
| Rv0621 | - | 354 | 0 | 0 | 0 | 0 | 0 | 0 | 0 | 0 | 0 | 0 | 0 | 0 | 0 | 73 | 0 | 0 | 100 | 64 | 43 | 42 | 42 | 41 | 100 | 100 | 100 | 0 | 65 |
| Rv0622 | - | 315 | 0 | 0 | 0 | 0 | 0 | 0 | 0 | 0 | 0 | 0 | 0 | 0 | 0 | 0 | 0 | 0 | 100 | 74 | 0 | 0 | 0 | 58 | 100 | 100 | 100 | 0 | 76 |
| Rv0623 | - | 84 | 0 | 0 | 0 | 0 | 0 | 0 | 0 | 0 | 0 | 0 | 0 | 0 | 0 | 0 | 0 | 0 | 100 | 0 | 0 | 0 | 0 | 92 | 100 | 100 | 100 | 0 | 0 |
| Rv0624 | - | 131 | 0 | 0 | 0 | 0 | 0 | 0 | 0 | 0 | 0 | 0 | 0 | 0 | 0 | 0 | 0 | 0 | 100 | 0 | 0 | 0 | 0 | 88 | 100 | 100 | 100 | 0 | 62 |
| Rv0625c | - | 246 | 44 | 45 | 43 | 0 | 40 | 45 | 50 | 0 | 0 | 43 | 42 | 41 | 0 | 83 | 81 | 83 | 100 | 76 | 76 | 76 | 76 | 82 | 100 | 100 | 100 | 46 | 80 |
| Rv0626 | - | 86 | 0 | 0 | 0 | 0 | 0 | 0 | 0 | 0 | 0 | 0 | 0 | 0 | 0 | 91 | 0 | 0 | 100 | 0 | 0 | 0 | 0 | 0 | 100 | 100 | 100 | 0 | 0 |
| Rv0627 | - | 135 | 0 | 0 | 0 | 0 | 0 | 0 | 0 | 0 | 0 | 0 | 0 | 0 | 0 | 91 | 0 | 0 | 100 | 0 | 0 | 0 | 0 | 0 | 100 | 100 | 100 | 0 | 0 |
| Rv0628c | - | 383 | 0 | 0 | 0 | 0 | 0 | 0 | 0 | 0 | 0 | 0 | 0 | 0 | 0 | 0 | 0 | 0 | 100 | 0 | 0 | 0 | 0 | 0 | 100 | 100 | 100 | 0 | 0 |
| Rv0629c | *recD* | 575 | 0 | 0 | 0 | 0 | 0 | 0 | 0 | 65 | 57 | 61 | 57 | 57 | 78 | 78 | 77 | 78 | 99 | 77 | 77 | 77 | 77 | 79 | 100 | 100 | 100 | 77 | 77 |
| Rv0630c | *recB* | 1094 | 34 | 37 | 38 | 36 | 37 | 0 | 42 | 61 | 56 | 56 | 58 | 57 | 72 | 75 | 75 | 75 | 99 | 72 | 74 | 74 | 74 | 75 | 100 | 100 | 100 | 74 | 74 |
| Rv0631c | *recC* | 1097 | 0 | 0 | 0 | 0 | 0 | 0 | 0 | 64 | 58 | 58 | 59 | 59 | 76 | 79 | 79 | 79 | 99 | 75 | 78 | 77 | 77 | 76 | 99 | 100 | 99 | 77 | 76 |
| Rv0632c | *echA3* | 231 | 0 | 0 | 0 | 0 | 0 | 0 | 0 | 45 | 44 | 44 | 45 | 40 | 44 | 90 | 91 | 90 | 100 | 72 | 71 | 71 | 71 | 73 | 100 | 100 | 100 | 91 | 71 |
| Rv0633c | - | 279 | 0 | 0 | 0 | 0 | 0 | 0 | 0 | 0 | 0 | 0 | 0 | 0 | 0 | 85 | 85 | 85 | 98 | 0 | 0 | 0 | 0 | 67 | 100 | 100 | 100 | 85 | 0 |
| Rv0634A | - | 83 | 0 | 0 | 0 | 0 | 0 | 0 | 0 | 0 | 0 | 0 | 0 | 0 | 0 | 73 | 73 | 73 | 100 | 0 | 0 | 0 | 0 | 0 | 100 | 100 | 100 | 74 | 0 |
| Rv0634B | *rpmG* | 55 | 0 | 0 | 0 | 0 | 0 | 0 | 0 | 85 | 94 | 98 | 98 | 98 | 94 | 100 | 100 | 100 | 100 | 100 | 98 | 98 | 98 | 96 | 100 | 100 | 100 | 96 | 100 |
| Rv0634c | - | 237 | 48 | 43 | 42 | 47 | 48 | 52 | 51 | 48 | 84 | 47 | 46 | 50 | 45 | 91 | 94 | 92 | 100 | 90 | 90 | 90 | 90 | 86 | 100 | 100 | 100 | 93 | 90 |
| Rv0635 | - | 158 | 0 | 0 | 0 | 0 | 0 | 0 | 0 | 0 | 68 | 65 | 67 | 67 | 77 | 91 | 91 | 91 | 100 | 82 | 84 | 83 | 83 | 86 | 100 | 98 | 100 | 88 | 82 |
| Rv0636 | - | 142 | 0 | 0 | 0 | 0 | 0 | 0 | 0 | 55 | 79 | 77 | 78 | 78 | 90 | 96 | 99 | 96 | 99 | 94 | 90 | 91 | 91 | 90 | 100 | 100 | 100 | 99 | 95 |
| Rv0637 | - | 166 | 0 | 0 | 0 | 0 | 0 | 0 | 0 | 0 | 64 | 64 | 64 | 64 | 68 | 89 | 91 | 89 | 100 | 76 | 80 | 81 | 81 | 83 | 100 | 100 | 100 | 91 | 77 |
| Rv0638 | *secE* | 161 | 0 | 0 | 57 | 52 | 56 | 54 | 50 | 0 | 53 | 53 | 54 | 54 | 58 | 72 | 75 | 71 | 100 | 62 | 62 | 61 | 61 | 64 | 100 | 100 | 100 | 75 | 59 |
| Rv0639 | *nusG* | 238 | 62 | 62 | 60 | 66 | 61 | 64 | 65 | 71 | 80 | 81 | 83 | 83 | 79 | 88 | 93 | 88 | 100 | 77 | 77 | 78 | 78 | 85 | 100 | 100 | 100 | 92 | 79 |
| Rv0640 | *rplK* | 142 | 86 | 88 | 89 | 88 | 85 | 88 | 85 | 83 | 93 | 93 | 93 | 93 | 95 | 95 | 98 | 95 | 100 | 94 | 95 | 95 | 95 | 96 | 100 | 100 | 100 | 98 | 94 |
| Rv0641 | *rplA* | 235 | 85 | 87 | 86 | 86 | 85 | 82 | 87 | 82 | 90 | 92 | 92 | 92 | 90 | 96 | 97 | 96 | 99 | 94 | 94 | 94 | 94 | 94 | 100 | 100 | 100 | 96 | 94 |
| Rv0642c | *mmaA4* | 301 | 45 | 0 | 0 | 49 | 0 | 48 | 43 | 47 | 44 | 47 | 48 | 48 | 72 | 95 | 94 | 95 | 99 | 83 | 82 | 82 | 82 | 73 | 100 | 100 | 100 | 94 | 83 |
| Rv0643c | *mmaA3* | 293 | 45 | 0 | 0 | 48 | 0 | 49 | 43 | 48 | 45 | 49 | 48 | 48 | 73 | 80 | 88 | 80 | 100 | 76 | 81 | 80 | 80 | 83 | 100 | 100 | 100 | 88 | 76 |
| Rv0644c | *mmaA2* | 287 | 45 | 0 | 0 | 46 | 0 | 47 | 44 | 47 | 43 | 47 | 48 | 47 | 77 | 86 | 90 | 86 | 99 | 81 | 84 | 84 | 84 | 86 | 100 | 100 | 100 | 90 | 81 |
| Rv0645c | *mmaA1* | 286 | 45 | 0 | 0 | 47 | 0 | 51 | 41 | 47 | 46 | 51 | 51 | 47 | 78 | 86 | 94 | 86 | 100 | 71 | 73 | 74 | 74 | 79 | 100 | 100 | 100 | 92 | 79 |
| Rv0646c | *lipG* | 301 | 0 | 0 | 0 | 0 | 0 | 0 | 0 | 55 | 0 | 42 | 0 | 0 | 71 | 88 | 83 | 87 | 100 | 79 | 78 | 79 | 79 | 78 | 100 | 100 | 100 | 83 | 78 |
| Rv0647c | - | 488 | 0 | 0 | 0 | 0 | 0 | 0 | 0 | 51 | 42 | 49 | 49 | 52 | 83 | 93 | 93 | 93 | 99 | 91 | 91 | 91 | 91 | 91 | 100 | 100 | 100 | 93 | 92 |
| Rv0648 | - | 1215 | 0 | 0 | 0 | 0 | 0 | 0 | 0 | 0 | 0 | 0 | 0 | 0 | 38 | 82 | 84 | 82 | 99 | 72 | 75 | 76 | 76 | 75 | 99 | 100 | 100 | 83 | 73 |
| Rv0649 | *fabD2* | 224 | 0 | 0 | 0 | 0 | 0 | 0 | 0 | 0 | 0 | 0 | 0 | 0 | 0 | 81 | 84 | 82 | 100 | 65 | 65 | 66 | 66 | 64 | 100 | 100 | 100 | 82 | 63 |

| | | | | | | | | | | | | | | | | | | | | | | | | | | | | | |
|---|---|---|---|---|---|---|---|---|---|---|---|---|---|---|---|---|---|---|---|---|---|---|---|---|---|---|---|---|---|
| Rv0650 | - | 302 | 47 | 44 | 45 | 46 | 47 | 43 | 46 | 48 | 59 | 61 | 64 | 64 | 0 | 80 | 85 | 80 | 100 | 77 | 80 | 80 | 80 | 79 | 100 | 100 | 100 | 85 | 78 |
| Rv0651 | *rplJ* | 178 | 72 | 79 | 78 | 78 | 78 | 71 | 77 | 72 | 90 | 88 | 88 | 88 | 90 | 95 | 93 | 94 | 100 | 89 | 93 | 93 | 93 | 93 | 100 | 100 | 100 | 93 | 87 |
| Rv0652 | *rplL* | 130 | 83 | 85 | 86 | 85 | 82 | 82 | 83 | 84 | 87 | 89 | 90 | 90 | 93 | 96 | 98 | 96 | 100 | 93 | 93 | 93 | 93 | 93 | 100 | 100 | 100 | 96 | 94 |
| Rv0653c | - | 231 | 0 | 0 | 0 | 0 | 0 | 0 | 0 | 0 | 43 | 0 | 53 | 0 | 74 | 81 | 82 | 82 | 100 | 58 | 59 | 59 | 59 | 0 | 100 | 100 | 100 | 78 | 0 |
| Rv0654 | - | 501 | 0 | 0 | 0 | 0 | 0 | 0 | 0 | 0 | 43 | 43 | 43 | 43 | 74 | 83 | 85 | 83 | 100 | 70 | 51 | 51 | 51 | 50 | 100 | 100 | 100 | 84 | 69 |
| Rv0655 | *mkl* | 359 | 53 | 56 | 55 | 57 | 54 | 53 | 53 | 82 | 86 | 89 | 89 | 91 | 92 | 96 | 97 | 96 | 100 | 93 | 91 | 94 | 94 | 91 | 100 | 100 | 99 | 97 | 93 |
| Rv0656c | - | 127 | 0 | 0 | 0 | 0 | 0 | 0 | 0 | 0 | 0 | 0 | 0 | 0 | 0 | 0 | 0 | 0 | 100 | 0 | 0 | 0 | 0 | 0 | 100 | 100 | 100 | 0 | 0 |
| Rv0657c | - | 51 | 0 | 0 | 0 | 0 | 0 | 0 | 0 | 0 | 0 | 0 | 0 | 0 | 0 | 0 | 0 | 0 | 100 | 0 | 0 | 0 | 0 | 0 | 100 | 100 | 100 | 0 | 0 |
| Rv0658c | - | 238 | 0 | 0 | 0 | 0 | 0 | 0 | 0 | 0 | 0 | 0 | 0 | 0 | 0 | 0 | 86 | 0 | 99 | 0 | 0 | 0 | 0 | 75 | 99 | 99 | 99 | 85 | 80 |
| Rv0659c | - | 102 | 0 | 0 | 0 | 0 | 0 | 0 | 0 | 0 | 0 | 0 | 0 | 0 | 0 | 0 | 0 | 0 | 100 | 0 | 0 | 0 | 0 | 0 | 100 | 100 | 100 | 0 | 0 |
| Rv0660c | - | 81 | 0 | 0 | 0 | 0 | 0 | 0 | 0 | 0 | 0 | 0 | 0 | 0 | 0 | 0 | 0 | 0 | 100 | 0 | 0 | 0 | 0 | 0 | 100 | 100 | 100 | 0 | 0 |
| Rv0661c | - | 145 | 0 | 0 | 0 | 0 | 0 | 0 | 0 | 0 | 0 | 0 | 0 | 0 | 0 | 0 | 0 | 0 | 100 | 0 | 0 | 0 | 0 | 0 | 100 | 100 | 100 | 0 | 0 |
| Rv0662c | - | 122 | 0 | 0 | 0 | 0 | 0 | 0 | 0 | 0 | 0 | 0 | 0 | 0 | 0 | 0 | 0 | 0 | 100 | 0 | 0 | 0 | 0 | 0 | 100 | 100 | 100 | 0 | 0 |
| Rv0663 | *atsD* | 787 | 0 | 0 | 43 | 0 | 0 | 0 | 0 | 67 | 0 | 77 | 57 | 72 | 54 | 54 | 54 | 54 | 99 | 53 | 79 | 79 | 79 | 54 | 100 | 100 | 100 | 77 | 78 |
| Rv0664 | - | 90 | 0 | 0 | 0 | 0 | 0 | 0 | 0 | 0 | 0 | 0 | 0 | 0 | 0 | 0 | 0 | 0 | 100 | 0 | 0 | 0 | 0 | 0 | 100 | 100 | 100 | 0 | 0 |
| Rv0665 | - | 112 | 0 | 0 | 0 | 0 | 0 | 0 | 0 | 0 | 0 | 0 | 0 | 0 | 0 | 55 | 0 | 0 | 100 | 0 | 0 | 0 | 0 | 0 | 100 | 100 | 100 | 0 | 0 |
| Rv0666 | - | 57 | 0 | 0 | 0 | 0 | 0 | 0 | 0 | 0 | 0 | 0 | 0 | 0 | 0 | 0 | 0 | 0 | 100 | 0 | 0 | 0 | 0 | 0 | 100 | 100 | 100 | 0 | 0 |
| Rv0667 | *rpoB* | 1172 | 85 | 87 | 86 | 86 | 88 | 85 | 87 | 85 | 93 | 93 | 93 | 93 | 94 | 95 | 95 | 95 | 100 | 94 | 95 | 94 | 94 | 95 | 100 | 100 | 99 | 96 | 96 |
| Rv0668 | *rpoC* | 1316 | 83 | 84 | 83 | 83 | 86 | 84 | 85 | 82 | 94 | 92 | 93 | 93 | 94 | 98 | 97 | 98 | 100 | 94 | 95 | 95 | 95 | 95 | 99 | 100 | 100 | 97 | 95 |
| Rv0669c | - | 637 | 0 | 0 | 0 | 0 | 54 | 0 | 0 | 0 | 59 | 58 | 59 | 0 | 0 | 0 | 87 | 0 | 99 | 0 | 0 | 0 | 0 | 0 | 100 | 100 | 100 | 84 | 0 |
| Rv0670 | *end* | 252 | 0 | 0 | 0 | 0 | 0 | 0 | 0 | 68 | 66 | 0 | 66 | 67 | 0 | 88 | 94 | 88 | 100 | 89 | 88 | 88 | 88 | 88 | 100 | 100 | 100 | 94 | 89 |
| Rv0671 | *lpqP* | 280 | 41 | 46 | 42 | 0 | 41 | 39 | 41 | 0 | 0 | 0 | 0 | 0 | 0 | 59 | 77 | 60 | 100 | 0 | 0 | 0 | 0 | 49 | 100 | 100 | 100 | 61 | 0 |
| Rv0672 | *fadE8* | 542 | 0 | 0 | 0 | 0 | 45 | 41 | 44 | 67 | 76 | 78 | 79 | 80 | 90 | 94 | 95 | 94 | 100 | 89 | 92 | 92 | 92 | 92 | 100 | 100 | 100 | 94 | 91 |
| Rv0673 | *echA4* | 312 | 42 | 0 | 42 | 0 | 0 | 0 | 0 | 43 | 78 | 60 | 79 | 43 | 86 | 96 | 95 | 96 | 100 | 44 | 94 | 95 | 95 | 94 | 100 | 100 | 100 | 94 | 91 |
| Rv0674 | - | 240 | 0 | 0 | 0 | 0 | 0 | 0 | 0 | 41 | 0 | 40 | 0 | 0 | 67 | 83 | 84 | 84 | 100 | 79 | 80 | 80 | 80 | 78 | 100 | 100 | 100 | 87 | 82 |
| Rv0675 | *echA5* | 263 | 53 | 46 | 47 | 0 | 53 | 51 | 54 | 75 | 80 | 74 | 73 | 73 | 84 | 93 | 94 | 93 | 100 | 89 | 88 | 88 | 88 | 85 | 100 | 100 | 100 | 93 | 89 |
| Rv0676c | *mmpL5* | 964 | 46 | 41 | 44 | 43 | 41 | 55 | 44 | 51 | 56 | 50 | 49 | 56 | 81 | 85 | 95 | 85 | 99 | 86 | 88 | 88 | 88 | 84 | 100 | 100 | 100 | 95 | 86 |
| Rv0677c | *mmpS5* | 142 | 0 | 0 | 0 | 0 | 0 | 0 | 0 | 0 | 0 | 0 | 0 | 0 | 67 | 74 | 74 | 74 | 100 | 87 | 87 | 87 | 87 | 78 | 100 | 100 | 100 | 96 | 88 |
| Rv0678 | - | 165 | 0 | 0 | 0 | 0 | 0 | 0 | 0 | 0 | 0 | 0 | 45 | 0 | 51 | 0 | 89 | 55 | 100 | 0 | 50 | 50 | 50 | 0 | 100 | 100 | 100 | 90 | 46 |
| Rv0679c | - | 165 | 0 | 0 | 0 | 0 | 0 | 0 | 0 | 0 | 0 | 0 | 0 | 0 | 56 | 72 | 77 | 72 | 100 | 60 | 63 | 63 | 63 | 61 | 100 | 100 | 100 | 78 | 77 |
| Rv0680c | - | 124 | 0 | 0 | 0 | 0 | 0 | 0 | 0 | 0 | 0 | 0 | 0 | 0 | 72 | 82 | 82 | 82 | 100 | 73 | 76 | 76 | 76 | 74 | 100 | 100 | 100 | 84 | 83 |
| Rv0681 | - | 196 | 0 | 0 | 0 | 0 | 0 | 0 | 0 | 0 | 0 | 51 | 57 | 57 | 72 | 98 | 94 | 98 | 100 | 87 | 89 | 89 | 89 | 90 | 100 | 100 | 100 | 94 | 87 |
| Rv0682 | *rpsL* | 124 | 95 | 96 | 96 | 96 | 96 | 98 | 94 | 96 | 98 | 96 | 96 | 96 | 98 | 99 | 99 | 99 | 100 | 98 | 99 | 99 | 99 | 100 | 100 | 100 | 100 | 98 | 98 |
| Rv0683 | *rpsG* | 156 | 88 | 91 | 90 | 90 | 92 | 89 | 92 | 91 | 94 | 95 | 96 | 95 | 97 | 98 | 100 | 98 | 100 | 97 | 97 | 97 | 97 | 99 | 100 | 100 | 100 | 98 | 97 |
| Rv0684 | *fusA1* | 701 | 86 | 87 | 86 | 86 | 87 | 85 | 86 | 83 | 91 | 90 | 90 | 90 | 93 | 96 | 96 | 96 | 100 | 93 | 94 | 94 | 94 | 93 | 100 | 100 | 100 | 95 | 94 |
| Rv0685 | *tuf* | 396 | 89 | 91 | 91 | 91 | 91 | 90 | 91 | 90 | 96 | 94 | 95 | 95 | 96 | 98 | 98 | 98 | 100 | 98 | 98 | 98 | 98 | 98 | 100 | 100 | 100 | 98 | 98 |
| Rv0686 | - | 265 | 0 | 0 | 0 | 0 | 0 | 0 | 0 | 0 | 0 | 0 | 0 | 0 | 67 | 84 | 84 | 84 | 99 | 79 | 77 | 77 | 77 | 74 | 100 | 100 | 100 | 92 | 78 |
| Rv0687 | *fabG* | 275 | 46 | 0 | 48 | 49 | 48 | 49 | 47 | 50 | 82 | 76 | 73 | 72 | 76 | 89 | 92 | 89 | 100 | 82 | 83 | 83 | 83 | 85 | 100 | 100 | 100 | 91 | 82 |

| | | | | | | | | | | | | | | | | | | | | | | | | | | | | | |
|---|---|---|---|---|---|---|---|---|---|---|---|---|---|---|---|---|---|---|---|---|---|---|---|---|---|---|---|---|---|
| Rv0688 | - | 406 | 49 | 47 | 53 | 61 | 45 | 50 | 53 | 57 | 69 | 74 | 77 | 76 | 76 | 88 | 90 | 88 | 100 | 85 | 84 | 84 | 84 | 85 | 99 | 100 | 100 | 89 | 84 |
| Rv0689c | - | 84 | 0 | 0 | 0 | 0 | 0 | 0 | 0 | 0 | 0 | 0 | 0 | 0 | 0 | 0 | 0 | 0 | 100 | 60 | 51 | 51 | 51 | 58 | 100 | 100 | 100 | 0 | 60 |
| Rv0690c | - | 349 | 0 | 0 | 0 | 0 | 0 | 0 | 0 | 46 | 40 | 45 | 47 | 0 | 0 | 77 | 77 | 77 | 100 | 0 | 0 | 0 | 0 | 0 | 100 | 100 | 100 | 76 | 0 |
| Rv0691c | - | 198 | 0 | 0 | 0 | 0 | 0 | 0 | 0 | 0 | 47 | 70 | 67 | 67 | 71 | 87 | 86 | 87 | 100 | 77 | 81 | 81 | 81 | 77 | 100 | 100 | 100 | 86 | 80 |
| Rv0692 | - | 109 | 0 | 0 | 0 | 0 | 0 | 0 | 0 | 0 | 65 | 78 | 70 | 70 | 73 | 89 | 89 | 89 | 100 | 77 | 73 | 73 | 73 | 73 | 100 | 100 | 100 | 90 | 76 |
| Rv0693 | *pqqE* | 391 | 0 | 43 | 40 | 0 | 0 | 0 | 0 | 45 | 80 | 82 | 83 | 83 | 87 | 91 | 91 | 91 | 100 | 87 | 90 | 90 | 90 | 89 | 100 | 100 | 100 | 92 | 87 |
| Rv0694 | *lldD1* | 396 | 50 | 0 | 51 | 51 | 52 | 52 | 0 | 51 | 83 | 86 | 88 | 88 | 88 | 94 | 95 | 94 | 100 | 90 | 92 | 92 | 92 | 90 | 100 | 100 | 100 | 95 | 90 |
| Rv0695 | - | 251 | 0 | 0 | 0 | 0 | 0 | 0 | 0 | 0 | 57 | 65 | 66 | 65 | 66 | 84 | 83 | 84 | 100 | 71 | 72 | 72 | 72 | 74 | 100 | 100 | 100 | 82 | 74 |
| Rv0696 | - | 470 | 0 | 0 | 45 | 0 | 0 | 0 | 0 | 0 | 63 | 63 | 65 | 65 | 81 | 89 | 90 | 89 | 100 | 84 | 84 | 84 | 84 | 83 | 100 | 100 | 99 | 90 | 84 |
| Rv0697 | - | 479 | 44 | 43 | 0 | 0 | 44 | 44 | 43 | 42 | 53 | 55 | 55 | 54 | 63 | 82 | 82 | 82 | 100 | 71 | 71 | 71 | 71 | 69 | 100 | 100 | 100 | 83 | 71 |
| Rv0698 | - | 203 | 0 | 0 | 0 | 0 | 0 | 0 | 0 | 0 | 0 | 0 | 0 | 0 | 0 | 0 | 0 | 0 | 98 | 0 | 0 | 0 | 0 | 0 | 100 | 100 | 100 | 0 | 0 |
| Rv0699 | - | 73 | 0 | 0 | 0 | 0 | 0 | 0 | 0 | 0 | 0 | 0 | 0 | 0 | 0 | 0 | 0 | 0 | 100 | 0 | 0 | 0 | 0 | 0 | 100 | 100 | 100 | 0 | 0 |
| Rv0700 | *rpsJ* | 101 | 97 | 97 | 97 | 97 | 98 | 99 | 97 | 93 | 98 | 98 | 98 | 98 | 99 | 100 | 100 | 100 | 100 | 99 | 99 | 99 | 99 | 99 | 100 | 100 | 100 | 100 | 99 |
| Rv0701 | *rplC* | 217 | 80 | 81 | 81 | 81 | 81 | 83 | 81 | 76 | 89 | 90 | 89 | 89 | 95 | 97 | 98 | 97 | 100 | 94 | 95 | 95 | 95 | 94 | 100 | 100 | 100 | 97 | 95 |
| Rv0702 | *rplD* | 223 | 79 | 81 | 81 | 83 | 80 | 78 | 83 | 75 | 82 | 84 | 82 | 83 | 88 | 94 | 94 | 94 | 100 | 91 | 92 | 92 | 92 | 90 | 100 | 100 | 100 | 92 | 93 |
| Rv0703 | *rplW* | 100 | 85 | 82 | 87 | 86 | 91 | 87 | 88 | 82 | 89 | 89 | 88 | 88 | 98 | 97 | 97 | 97 | 100 | 93 | 95 | 95 | 95 | 94 | 100 | 100 | 100 | 96 | 93 |
| Rv0704 | *rplB* | 280 | 87 | 87 | 87 | 86 | 88 | 87 | 87 | 90 | 91 | 94 | 95 | 95 | 96 | 96 | 97 | 97 | 100 | 96 | 96 | 96 | 96 | 96 | 100 | 100 | 100 | 98 | 96 |
| Rv0705 | *rpsS* | 93 | 93 | 94 | 94 | 94 | 92 | 94 | 93 | 89 | 94 | 97 | 97 | 97 | 100 | 97 | 100 | 97 | 100 | 100 | 100 | 100 | 100 | 100 | 100 | 100 | 100 | 100 | 100 |
| Rv0706 | *rplV* | 197 | 65 | 87 | 68 | 68 | 66 | 63 | 66 | 61 | 77 | 64 | 68 | 68 | 76 | 87 | 84 | 87 | 100 | 79 | 71 | 72 | 72 | 90 | 100 | 100 | 100 | 83 | 76 |
| Rv0707 | *rpsC* | 274 | 80 | 83 | 84 | 84 | 85 | 85 | 82 | 79 | 94 | 94 | 94 | 94 | 94 | 94 | 97 | 94 | 100 | 94 | 95 | 95 | 95 | 94 | 100 | 100 | 100 | 97 | 92 |
| Rv0708 | *rplP* | 138 | 82 | 83 | 83 | 82 | 82 | 84 | 81 | 88 | 92 | 90 | 90 | 90 | 95 | 97 | 99 | 97 | 100 | 97 | 97 | 97 | 97 | 97 | 100 | 100 | 100 | 99 | 97 |
| Rv0709 | *rpmC* | 77 | 79 | 80 | 83 | 83 | 81 | 81 | 85 | 82 | 86 | 85 | 85 | 85 | 88 | 97 | 93 | 97 | 100 | 90 | 96 | 96 | 96 | 96 | 100 | 100 | 100 | 93 | 90 |
| Rv0710 | *rpsQ* | 136 | 81 | 77 | 83 | 82 | 76 | 78 | 82 | 82 | 93 | 89 | 90 | 90 | 85 | 86 | 77 | 86 | 100 | 86 | 75 | 74 | 74 | 90 | 100 | 100 | 100 | 76 | 88 |
| Rv0711 | *atsA* | 787 | 0 | 0 | 45 | 0 | 0 | 0 | 0 | 61 | 0 | 67 | 69 | 70 | 66 | 93 | 93 | 93 | 99 | 87 | 87 | 88 | 88 | 88 | 100 | 100 | 100 | 69 | 87 |
| Rv0712 | - | 299 | 0 | 0 | 51 | 0 | 0 | 0 | 0 | 39 | 46 | 63 | 63 | 65 | 79 | 86 | 85 | 85 | 100 | 82 | 84 | 83 | 83 | 80 | 100 | 100 | 100 | 63 | 80 |
| Rv0713 | - | 313 | 0 | 0 | 0 | 0 | 0 | 0 | 0 | 0 | 0 | 0 | 49 | 49 | 63 | 74 | 89 | 74 | 100 | 62 | 0 | 0 | 0 | 0 | 100 | 100 | 100 | 89 | 0 |
| Rv0714 | *rplN* | 122 | 92 | 94 | 93 | 93 | 88 | 94 | 88 | 97 | 99 | 98 | 99 | 99 | 98 | 100 | 100 | 100 | 100 | 97 | 97 | 97 | 97 | 98 | 100 | 100 | 100 | 100 | 97 |
| Rv0715 | *rplX* | 105 | 87 | 86 | 86 | 85 | 89 | 88 | 87 | 69 | 90 | 92 | 92 | 92 | 94 | 96 | 99 | 96 | 100 | 91 | 96 | 96 | 96 | 95 | 100 | 100 | 100 | 99 | 90 |
| Rv0716 | *rplE* | 187 | 86 | 85 | 86 | 87 | 86 | 88 | 87 | 82 | 90 | 89 | 88 | 88 | 88 | 96 | 96 | 97 | 100 | 88 | 92 | 92 | 92 | 90 | 100 | 100 | 100 | 96 | 89 |
| Rv0717 | *rpsN* | 61 | 68 | 72 | 70 | 73 | 0 | 70 | 70 | 86 | 90 | 93 | 91 | 91 | 95 | 96 | 96 | 96 | 100 | 91 | 90 | 90 | 90 | 95 | 100 | 100 | 100 | 96 | 91 |
| Rv0718 | *rpsH* | 132 | 85 | 92 | 88 | 90 | 90 | 90 | 86 | 84 | 96 | 96 | 96 | 96 | 98 | 100 | 99 | 100 | 100 | 99 | 99 | 99 | 99 | 99 | 100 | 100 | 100 | 99 | 98 |
| Rv0719 | *rplF* | 179 | 83 | 86 | 85 | 86 | 85 | 86 | 86 | 80 | 89 | 91 | 92 | 92 | 96 | 98 | 96 | 98 | 100 | 94 | 95 | 95 | 95 | 95 | 100 | 100 | 100 | 96 | 94 |
| Rv0720 | *rplR* | 122 | 81 | 82 | 81 | 81 | 80 | 80 | 79 | 81 | 81 | 82 | 82 | 83 | 91 | 93 | 94 | 93 | 100 | 88 | 90 | 90 | 90 | 90 | 100 | 100 | 100 | 92 | 88 |
| Rv0721 | *rpsE* | 220 | 76 | 83 | 84 | 83 | 79 | 83 | 78 | 86 | 87 | 86 | 88 | 88 | 88 | 94 | 92 | 94 | 100 | 88 | 91 | 91 | 91 | 90 | 100 | 100 | 100 | 92 | 89 |
| Rv0722 | *rpmD* | 65 | 0 | 70 | 77 | 77 | 77 | 81 | 79 | 68 | 82 | 80 | 82 | 82 | 91 | 85 | 85 | 85 | 100 | 91 | 85 | 84 | 84 | 91 | 100 | 100 | 100 | 95 | 89 |
| Rv0723 | *rplO* | 146 | 77 | 79 | 81 | 82 | 81 | 78 | 79 | 84 | 83 | 84 | 83 | 85 | 86 | 95 | 93 | 95 | 99 | 90 | 90 | 90 | 90 | 92 | 100 | 100 | 100 | 93 | 90 |
| Rv0724 | *sppA* | 623 | 0 | 0 | 0 | 0 | 0 | 0 | 0 | 0 | 0 | 0 | 44 | 46 | 76 | 86 | 89 | 85 | 100 | 82 | 84 | 84 | 84 | 83 | 100 | 100 | 100 | 87 | 82 |
| Rv0724A | - | 111 | 0 | 0 | 0 | 0 | 0 | 0 | 0 | 0 | 0 | 0 | 0 | 0 | 50 | 83 | 81 | 80 | 100 | 70 | 75 | 76 | 76 | 66 | 100 | 100 | 99 | 80 | 71 |

| | | | | | | | | | | | | | | | | | | | | | | | | | | | | | |
|---|---|---|---|---|---|---|---|---|---|---|---|---|---|---|---|---|---|---|---|---|---|---|---|---|---|---|---|---|---|
| Rv0725c | - | 301 | 0 | 0 | 0 | 0 | 0 | 0 | 0 | 0 | 47 | 59 | 60 | 0 | 73 | 88 | 86 | 88 | 100 | 79 | 84 | 83 | 83 | 79 | 100 | 100 | 99 | 86 | 82 |
| Rv0726c | - | 367 | 0 | 0 | 0 | 0 | 0 | 0 | 0 | 0 | 48 | 57 | 56 | 0 | 63 | 78 | 79 | 79 | 100 | 67 | 68 | 71 | 71 | 63 | 100 | 100 | 100 | 76 | 61 |
| Rv0727c | *fucA* | 218 | 0 | 0 | 0 | 0 | 0 | 0 | 0 | 51 | 44 | 0 | 0 | 0 | 42 | 88 | 92 | 89 | 99 | 0 | 0 | 0 | 0 | 50 | 99 | 100 | 99 | 92 | 44 |
| Rv0728c | *serA2* | 326 | 48 | 47 | 49 | 49 | 49 | 49 | 47 | 51 | 50 | 50 | 50 | 50 | 49 | 90 | 87 | 90 | 99 | 56 | 53 | 53 | 53 | 56 | 100 | 100 | 100 | 87 | 54 |
| Rv0729 | *xylB* | 448 | 0 | 40 | 0 | 37 | 0 | 0 | 0 | 43 | 0 | 0 | 39 | 40 | 0 | 83 | 86 | 83 | 99 | 39 | 43 | 43 | 43 | 44 | 100 | 100 | 100 | 85 | 38 |
| Rv0730 | - | 242 | 0 | 0 | 0 | 0 | 0 | 0 | 0 | 0 | 0 | 62 | 62 | 60 | 82 | 87 | 87 | 87 | 100 | 73 | 72 | 72 | 72 | 83 | 100 | 100 | 100 | 92 | 68 |
| Rv0731c | - | 318 | 0 | 0 | 0 | 0 | 0 | 0 | 0 | 0 | 44 | 55 | 56 | 0 | 65 | 75 | 82 | 75 | 100 | 72 | 72 | 72 | 72 | 73 | 100 | 100 | 100 | 72 | 71 |
| Rv0732 | *secY* | 441 | 80 | 81 | 79 | 80 | 79 | 80 | 80 | 77 | 87 | 89 | 86 | 86 | 91 | 97 | 97 | 97 | 100 | 91 | 93 | 93 | 93 | 94 | 100 | 100 | 100 | 97 | 91 |
| Rv0733 | *adk* | 181 | 77 | 76 | 79 | 77 | 77 | 78 | 76 | 70 | 81 | 82 | 83 | 83 | 86 | 95 | 95 | 96 | 100 | 85 | 88 | 88 | 88 | 89 | 100 | 100 | 100 | 95 | 87 |
| Rv0734 | *mapA* | 266 | 74 | 76 | 74 | 75 | 71 | 71 | 75 | 66 | 86 | 86 | 86 | 86 | 86 | 92 | 92 | 92 | 100 | 88 | 86 | 86 | 86 | 88 | 100 | 100 | 100 | 91 | 89 |
| Rv0735 | *sigL* | 177 | 47 | 48 | 45 | 47 | 46 | 47 | 50 | 67 | 71 | 45 | 76 | 76 | 75 | 95 | 90 | 95 | 100 | 81 | 79 | 79 | 79 | 75 | 100 | 100 | 100 | 89 | 84 |
| Rv0736 | - | 250 | 0 | 0 | 0 | 0 | 0 | 0 | 0 | 49 | 55 | 0 | 52 | 51 | 50 | 77 | 77 | 77 | 100 | 59 | 59 | 59 | 59 | 61 | 100 | 100 | 100 | 76 | 61 |
| Rv0737 | - | 165 | 59 | 0 | 0 | 0 | 0 | 0 | 0 | 59 | 71 | 72 | 72 | 73 | 74 | 95 | 95 | 95 | 100 | 91 | 91 | 92 | 92 | 93 | 100 | 100 | 100 | 93 | 93 |
| Rv0738 | - | 182 | 0 | 0 | 0 | 0 | 0 | 0 | 0 | 40 | 39 | 0 | 46 | 48 | 49 | 42 | 81 | 42 | 100 | 0 | 43 | 43 | 43 | 0 | 100 | 100 | 100 | 81 | 39 |
| Rv0739 | - | 268 | 0 | 0 | 0 | 0 | 0 | 0 | 0 | 0 | 0 | 0 | 0 | 0 | 0 | 50 | 55 | 53 | 100 | 0 | 0 | 0 | 0 | 0 | 100 | 100 | 100 | 57 | 0 |
| Rv0740 | - | 175 | 0 | 0 | 0 | 0 | 0 | 0 | 0 | 0 | 0 | 0 | 0 | 0 | 0 | 0 | 0 | 0 | 100 | 0 | 0 | 0 | 0 | 0 | 100 | 100 | 100 | 0 | 0 |
| Rv0741 | - | 104 | 0 | 0 | 0 | 0 | 0 | 0 | 0 | 66 | 0 | 0 | 59 | 0 | 0 | 0 | 0 | 0 | 100 | 56 | 0 | 0 | 0 | 0 | 100 | 100 | 100 | 0 | 0 |
| Rv0742 | *PE_PGRS8* | 175 | 0 | 0 | 0 | 0 | 40 | 0 | 41 | 0 | 0 | 0 | 0 | 0 | 0 | 60 | 77 | 55 | 100 | 40 | 41 | 40 | 40 | 0 | 100 | 100 | 100 | 76 | 60 |
| Rv0743c | - | 185 | 0 | 0 | 0 | 0 | 0 | 0 | 0 | 61 | 0 | 0 | 0 | 0 | 0 | 0 | 0 | 0 | 100 | 0 | 0 | 0 | 0 | 0 | 100 | 100 | 100 | 0 | 57 |
| Rv0744c | - | 168 | 0 | 0 | 0 | 0 | 0 | 0 | 0 | 0 | 0 | 0 | 0 | 0 | 0 | 0 | 0 | 0 | 100 | 0 | 0 | 0 | 0 | 0 | 100 | 100 | 100 | 0 | 0 |
| Rv0745 | - | 175 | 0 | 0 | 0 | 0 | 0 | 0 | 0 | 0 | 74 | 72 | 76 | 77 | 0 | 77 | 81 | 77 | 100 | 0 | 0 | 0 | 0 | 0 | 100 | 100 | 100 | 48 | 0 |
| Rv0746 | *PE_PGRS9* | 783 | 43 | 31 | 33 | 39 | 33 | 35 | 40 | 38 | 41 | 38 | 39 | 42 | 39 | 32 | 69 | 33 | 89 | 51 | 47 | 49 | 49 | 42 | 99 | 99 | 99 | 65 | 51 |
| Rv0747 | *PE_PGRS10* | 801 | 44 | 43 | 42 | 33 | 38 | 39 | 35 | 36 | 39 | 37 | 39 | 40 | 39 | 48 | 59 | 34 | 86 | 49 | 49 | 49 | 49 | 40 | 97 | 99 | 91 | 56 | 51 |
| Rv0748 | - | 85 | 0 | 0 | 0 | 0 | 0 | 0 | 0 | 0 | 0 | 0 | 0 | 0 | 0 | 0 | 0 | 0 | 100 | 0 | 0 | 0 | 0 | 0 | 100 | 100 | 100 | 0 | 0 |
| Rv0749 | - | 142 | 0 | 0 | 0 | 0 | 0 | 0 | 0 | 0 | 0 | 0 | 0 | 0 | 0 | 0 | 0 | 0 | 100 | 50 | 0 | 0 | 0 | 0 | 100 | 100 | 100 | 0 | 0 |
| Rv0749A | - | 45 | 0 | 0 | 0 | 0 | 0 | 0 | 0 | 0 | 0 | 0 | 0 | 0 | 0 | 0 | 0 | 0 | 100 | 0 | 0 | 0 | 0 | 0 | 100 | 100 | 100 | 0 | 0 |
| Rv0750 | - | 81 | 0 | 0 | 0 | 0 | 0 | 0 | 0 | 0 | 0 | 0 | 0 | 0 | 0 | 0 | 0 | 0 | 100 | 0 | 0 | 0 | 0 | 0 | 100 | 100 | 100 | 0 | 0 |
| Rv0751c | *mmsB* | 294 | 65 | 43 | 0 | 0 | 0 | 0 | 0 | 59 | 70 | 73 | 72 | 72 | 67 | 86 | 89 | 86 | 100 | 81 | 84 | 84 | 84 | 83 | 100 | 100 | 100 | 88 | 80 |
| Rv0752c | *fadE9* | 390 | 45 | 0 | 46 | 43 | 59 | 56 | 57 | 58 | 75 | 78 | 79 | 78 | 73 | 94 | 94 | 94 | 99 | 88 | 88 | 88 | 88 | 85 | 100 | 100 | 100 | 94 | 89 |
| Rv0753c | *mmsA* | 510 | 77 | 45 | 47 | 66 | 49 | 49 | 47 | 67 | 80 | 81 | 81 | 81 | 82 | 96 | 94 | 96 | 99 | 92 | 91 | 91 | 91 | 91 | 99 | 100 | 100 | 94 | 92 |
| Rv0754 | *PE_PGRS11* | 584 | 38 | 0 | 41 | 0 | 0 | 0 | 37 | 0 | 45 | 36 | 39 | 57 | 39 | 70 | 70 | 70 | 100 | 59 | 57 | 57 | 57 | 56 | 99 | 100 | 100 | 71 | 63 |
| Rv0755A | - | 61 | 0 | 0 | 0 | 0 | 0 | 0 | 0 | 0 | 0 | 0 | 0 | 0 | 0 | 94 | 0 | 0 | 100 | 0 | 0 | 0 | 0 | 0 | 100 | 100 | 100 | 0 | 0 |
| Rv0755c | *PPE12* | 645 | 40 | 0 | 0 | 0 | 0 | 0 | 0 | 0 | 44 | 35 | 34 | 37 | 42 | 69 | 61 | 69 | 100 | 32 | 37 | 40 | 40 | 37 | 100 | 100 | 100 | 61 | 32 |
| Rv0756c | - | 241 | 0 | 0 | 0 | 0 | 0 | 0 | 0 | 0 | 0 | 54 | 52 | 52 | 61 | 90 | 90 | 90 | 100 | 80 | 80 | 80 | 80 | 69 | 99 | 100 | 100 | 92 | 81 |
| Rv0757 | *phoP* | 247 | 81 | 81 | 81 | 81 | 76 | 77 | 78 | 77 | 85 | 89 | 90 | 90 | 91 | 95 | 97 | 96 | 100 | 94 | 97 | 97 | 97 | 97 | 100 | 99 | 100 | 97 | 95 |
| Rv0758 | *phoR* | 485 | 60 | 58 | 59 | 59 | 55 | 57 | 60 | 50 | 64 | 70 | 72 | 72 | 74 | 85 | 83 | 85 | 99 | 82 | 83 | 83 | 83 | 83 | 99 | 100 | 99 | 83 | 82 |
| Rv0759c | - | 110 | 64 | 62 | 64 | 64 | 62 | 64 | 64 | 0 | 76 | 77 | 77 | 78 | 78 | 93 | 91 | 92 | 100 | 94 | 88 | 89 | 89 | 94 | 100 | 100 | 100 | 91 | 93 |
| Rv0760c | - | 139 | 0 | 0 | 0 | 0 | 0 | 0 | 0 | 51 | 56 | 55 | 54 | 52 | 53 | 89 | 92 | 92 | 100 | 82 | 85 | 86 | 86 | 88 | 100 | 100 | 100 | 92 | 85 |

| | | | | | | | | | | | | | | | | | | | | | | | | | | | | | |
|---|---|---|---|---|---|---|---|---|---|---|---|---|---|---|---|---|---|---|---|---|---|---|---|---|---|---|---|---|---|
| Rv0761c | *adhB* | 375 | 52 | 44 | 46 | 46 | 49 | 47 | 49 | 47 | 80 | 82 | 82 | 82 | 75 | 94 | 94 | 94 | 100 | 89 | 92 | 92 | 92 | 91 | 100 | 100 | 100 | 95 | 89 |
| Rv0762c | - | 181 | 0 | 0 | 0 | 0 | 0 | 0 | 0 | 0 | 77 | 76 | 77 | 76 | 85 | 80 | 81 | 80 | 100 | 78 | 79 | 79 | 79 | 81 | 100 | 100 | 100 | 81 | 78 |
| Rv0763c | - | 68 | 0 | 0 | 0 | 0 | 0 | 0 | 0 | 0 | 72 | 72 | 78 | 76 | 80 | 89 | 89 | 89 | 100 | 80 | 79 | 79 | 79 | 80 | 100 | 100 | 100 | 89 | 79 |
| Rv0764c | *cyp51* | 451 | 0 | 0 | 0 | 0 | 47 | 0 | 0 | 42 | 85 | 85 | 86 | 86 | 90 | 90 | 90 | 90 | 100 | 89 | 89 | 89 | 89 | 89 | 100 | 100 | 100 | 90 | 89 |
| Rv0765c | - | 275 | 50 | 51 | 44 | 52 | 46 | 49 | 47 | 47 | 70 | 70 | 68 | 68 | 89 | 82 | 81 | 82 | 100 | 81 | 80 | 80 | 80 | 84 | 100 | 100 | 100 | 80 | 80 |
| Rv0766c | *cyp123* | 402 | 0 | 0 | 47 | 49 | 43 | 0 | 0 | 59 | 74 | 75 | 78 | 78 | 85 | 83 | 84 | 80 | 100 | 83 | 85 | 85 | 85 | 87 | 100 | 100 | 100 | 84 | 85 |
| Rv0767c | - | 213 | 0 | 0 | 0 | 0 | 0 | 0 | 0 | 0 | 63 | 62 | 58 | 59 | 75 | 80 | 80 | 80 | 100 | 76 | 83 | 83 | 83 | 78 | 100 | 100 | 100 | 83 | 80 |
| Rv0768 | *aldA* | 489 | 53 | 50 | 54 | 54 | 52 | 54 | 51 | 58 | 80 | 81 | 80 | 80 | 87 | 92 | 92 | 92 | 100 | 87 | 91 | 90 | 90 | 88 | 100 | 100 | 100 | 91 | 88 |
| Rv0769 | - | 248 | 48 | 46 | 57 | 52 | 49 | 50 | 47 | 54 | 80 | 81 | 84 | 85 | 87 | 88 | 88 | 88 | 99 | 84 | 84 | 84 | 84 | 85 | 100 | 100 | 100 | 93 | 84 |
| Rv0770 | - | 295 | 46 | 48 | 0 | 0 | 0 | 0 | 0 | 44 | 74 | 78 | 76 | 75 | 79 | 79 | 80 | 80 | 99 | 79 | 77 | 77 | 77 | 80 | 100 | 100 | 99 | 80 | 78 |
| Rv0771 | - | 144 | 0 | 0 | 0 | 0 | 0 | 0 | 0 | 68 | 61 | 77 | 72 | 72 | 78 | 75 | 76 | 75 | 100 | 78 | 77 | 77 | 77 | 77 | 100 | 100 | 100 | 75 | 78 |
| Rv0772 | *purD* | 422 | 71 | 73 | 74 | 74 | 71 | 72 | 71 | 66 | 81 | 83 | 82 | 81 | 83 | 90 | 92 | 91 | 100 | 88 | 89 | 89 | 89 | 90 | 100 | 100 | 100 | 92 | 87 |
| Rv0773c | *ggtA* | 512 | 0 | 0 | 43 | 45 | 0 | 43 | 0 | 47 | 45 | 0 | 47 | 46 | 44 | 89 | 88 | 88 | 100 | 45 | 52 | 52 | 52 | 46 | 100 | 100 | 100 | 85 | 45 |
| Rv0774c | - | 303 | 0 | 0 | 0 | 0 | 0 | 0 | 0 | 0 | 0 | 0 | 0 | 0 | 72 | 86 | 90 | 86 | 99 | 72 | 77 | 77 | 77 | 77 | 100 | 100 | 100 | 89 | 71 |
| Rv0775 | - | 207 | 0 | 0 | 0 | 0 | 0 | 0 | 0 | 0 | 67 | 71 | 70 | 70 | 72 | 93 | 98 | 93 | 100 | 84 | 80 | 81 | 81 | 83 | 100 | 100 | 100 | 98 | 83 |
| Rv0776c | - | 259 | 0 | 0 | 0 | 0 | 0 | 0 | 0 | 67 | 0 | 0 | 0 | 0 | 0 | 74 | 75 | 75 | 100 | 69 | 68 | 68 | 68 | 70 | 100 | 100 | 100 | 81 | 69 |
| Rv0777 | *purB* | 472 | 81 | 83 | 81 | 81 | 80 | 82 | 79 | 78 | 87 | 84 | 85 | 85 | 88 | 93 | 90 | 94 | 99 | 91 | 90 | 90 | 90 | 91 | 100 | 100 | 100 | 90 | 92 |
| Rv0778 | *cyp126* | 414 | 0 | 0 | 43 | 45 | 40 | 0 | 0 | 53 | 53 | 54 | 56 | 56 | 50 | 89 | 87 | 89 | 100 | 63 | 85 | 85 | 85 | 85 | 100 | 100 | 100 | 87 | 85 |
| Rv0779c | - | 206 | 0 | 0 | 0 | 0 | 0 | 0 | 0 | 0 | 0 | 0 | 0 | 0 | 78 | 82 | 84 | 82 | 100 | 77 | 81 | 81 | 81 | 81 | 100 | 100 | 100 | 83 | 80 |
| Rv0780 | *hemH* | 297 | 78 | 79 | 80 | 79 | 80 | 76 | 81 | 70 | 67 | 81 | 82 | 81 | 85 | 92 | 92 | 92 | 100 | 85 | 86 | 86 | 86 | 85 | 100 | 100 | 100 | 93 | 83 |
| Rv0781 | *ptrBa* | 236 | 68 | 69 | 65 | 63 | 68 | 67 | 72 | 71 | 78 | 76 | 78 | 77 | 80 | 87 | 93 | 87 | 98 | 89 | 80 | 80 | 80 | 78 | 98 | 100 | 98 | 93 | 81 |
| Rv0782 | *ptrBb* | 552 | 68 | 72 | 71 | 70 | 65 | 67 | 67 | 68 | 77 | 77 | 76 | 77 | 79 | 87 | 91 | 87 | 100 | 84 | 86 | 87 | 87 | 85 | 100 | 100 | 100 | 90 | 85 |
| Rv0783c | *emrB* | 540 | 49 | 49 | 48 | 50 | 46 | 46 | 47 | 65 | 67 | 67 | 68 | 68 | 52 | 79 | 77 | 79 | 100 | 47 | 47 | 48 | 48 | 48 | 99 | 100 | 100 | 77 | 48 |
| Rv0784 | - | 228 | 53 | 0 | 53 | 57 | 47 | 0 | 0 | 0 | 70 | 69 | 73 | 73 | 78 | 92 | 89 | 92 | 100 | 78 | 79 | 79 | 79 | 84 | 100 | 100 | 100 | 89 | 82 |
| Rv0785 | - | 566 | 67 | 0 | 0 | 0 | 72 | 0 | 70 | 77 | 77 | 78 | 77 | 77 | 84 | 90 | 74 | 90 | 99 | 87 | 72 | 72 | 72 | 88 | 99 | 100 | 99 | 64 | 86 |
| Rv0786c | - | 129 | 51 | 0 | 48 | 48 | 0 | 0 | 0 | 59 | 74 | 79 | 79 | 80 | 80 | 93 | 91 | 93 | 100 | 86 | 81 | 80 | 80 | 88 | 100 | 100 | 100 | 0 | 86 |
| Rv0787 | - | 319 | 0 | 0 | 0 | 0 | 0 | 0 | 0 | 0 | 46 | 0 | 59 | 64 | 62 | 74 | 74 | 74 | 100 | 60 | 65 | 65 | 65 | 55 | 100 | 100 | 100 | 76 | 60 |
| Rv0787A | - | 79 | 86 | 0 | 86 | 87 | 79 | 82 | 81 | 78 | 86 | 86 | 88 | 88 | 83 | 93 | 97 | 93 | 100 | 89 | 90 | 90 | 90 | 94 | 100 | 100 | 100 | 97 | 91 |
| Rv0788 | *purQ* | 224 | 81 | 43 | 76 | 79 | 81 | 81 | 77 | 79 | 87 | 85 | 87 | 87 | 90 | 95 | 95 | 95 | 100 | 90 | 92 | 92 | 92 | 90 | 100 | 100 | 100 | 95 | 91 |
| Rv0789c | - | 199 | 0 | 0 | 0 | 0 | 0 | 0 | 0 | 0 | 0 | 0 | 0 | 0 | 0 | 67 | 66 | 67 | 100 | 0 | 0 | 0 | 0 | 0 | 100 | 100 | 100 | 66 | 0 |
| Rv0790c | - | 242 | 0 | 0 | 0 | 0 | 0 | 0 | 0 | 0 | 0 | 0 | 0 | 0 | 45 | 0 | 77 | 0 | 100 | 47 | 45 | 45 | 45 | 0 | 100 | 100 | 99 | 76 | 0 |
| Rv0791c | - | 347 | 42 | 0 | 42 | 40 | 0 | 0 | 40 | 60 | 49 | 48 | 49 | 48 | 49 | 51 | 51 | 51 | 100 | 48 | 49 | 49 | 49 | 52 | 100 | 100 | 99 | 75 | 50 |
| Rv0792c | - | 269 | 49 | 46 | 46 | 48 | 0 | 0 | 0 | 45 | 46 | 48 | 46 | 45 | 45 | 84 | 79 | 85 | 100 | 46 | 46 | 47 | 47 | 47 | 97 | 100 | 100 | 79 | 47 |
| Rv0793 | - | 101 | 0 | 0 | 0 | 0 | 0 | 0 | 0 | 0 | 0 | 0 | 0 | 0 | 0 | 0 | 74 | 0 | 100 | 0 | 0 | 0 | 0 | 0 | 0 | 100 | 100 | 73 | 0 |
| Rv0794c | - | 499 | 44 | 43 | 43 | 42 | 44 | 45 | 44 | 51 | 61 | 59 | 59 | 59 | 43 | 44 | 43 | 43 | 100 | 45 | 44 | 44 | 44 | 78 | 100 | 100 | 100 | 86 | 44 |
| Rv0795 | - | 108 | 65 | 0 | 66 | 0 | 61 | 0 | 60 | 76 | 0 | 0 | 0 | 0 | 59 | 63 | 66 | 66 | 100 | 82 | 96 | 77 | 77 | 84 | 100 | 100 | 100 | 0 | 62 |
| Rv0796 | - | 312 | 58 | 49 | 61 | 51 | 63 | 0 | 61 | 56 | 0 | 0 | 51 | 52 | 66 | 59 | 66 | 66 | 100 | 66 | 90 | 67 | 67 | 66 | 100 | 100 | 100 | 0 | 65 |
| Rv0797 | - | 364 | 0 | 0 | 45 | 38 | 40 | 0 | 0 | 0 | 0 | 0 | 57 | 0 | 0 | 80 | 45 | 81 | 100 | 74 | 0 | 39 | 39 | 45 | 100 | 100 | 99 | 0 | 39 |

| | | | | | | | | | | | | | | | | | | | | | | | | | | | | | |
|---|---|---|---|---|---|---|---|---|---|---|---|---|---|---|---|---|---|---|---|---|---|---|---|---|---|---|---|---|---|
| Rv0798c | *cfp29* | 265 | 0 | 0 | 0 | 0 | 0 | 0 | 0 | 0 | 81 | 82 | 73 | 74 | 88 | 94 | 97 | 94 | 100 | 92 | 0 | 0 | 0 | 92 | 100 | 100 | 100 | 97 | 90 |
| Rv0799c | - | 335 | 0 | 0 | 0 | 0 | 0 | 0 | 0 | 0 | 67 | 74 | 70 | 71 | 76 | 87 | 90 | 86 | 100 | 82 | 0 | 0 | 0 | 84 | 100 | 100 | 100 | 90 | 84 |
| Rv0800 | *pepC* | 433 | 58 | 56 | 58 | 58 | 0 | 52 | 0 | 0 | 77 | 78 | 76 | 77 | 76 | 88 | 87 | 88 | 99 | 81 | 82 | 82 | 82 | 83 | 99 | 100 | 99 | 85 | 81 |
| Rv0801 | - | 115 | 0 | 0 | 0 | 0 | 0 | 0 | 0 | 48 | 54 | 62 | 69 | 69 | 54 | 82 | 82 | 81 | 100 | 71 | 73 | 73 | 73 | 71 | 100 | 100 | 100 | 81 | 72 |
| Rv0802c | - | 218 | 47 | 0 | 0 | 0 | 0 | 0 | 0 | 56 | 0 | 52 | 0 | 0 | 0 | 0 | 87 | 0 | 100 | 0 | 0 | 0 | 0 | 0 | 100 | 100 | 100 | 87 | 0 |
| Rv0803 | *purL* | 754 | 75 | 37 | 75 | 76 | 76 | 72 | 77 | 76 | 89 | 89 | 90 | 90 | 92 | 95 | 96 | 96 | 100 | 88 | 92 | 92 | 92 | 93 | 100 | 100 | 100 | 94 | 91 |
| Rv0804 | - | 209 | 0 | 0 | 0 | 0 | 0 | 0 | 0 | 0 | 46 | 51 | 48 | 47 | 61 | 73 | 84 | 73 | 100 | 62 | 71 | 71 | 71 | 70 | 100 | 100 | 100 | 83 | 65 |
| Rv0805 | - | 318 | 0 | 0 | 0 | 0 | 0 | 0 | 0 | 66 | 77 | 75 | 76 | 75 | 0 | 0 | 89 | 0 | 100 | 0 | 0 | 0 | 0 | 0 | 100 | 100 | 100 | 89 | 0 |
| Rv0806c | *cpsY* | 532 | 0 | 0 | 0 | 0 | 0 | 0 | 0 | 52 | 69 | 71 | 72 | 72 | 0 | 0 | 91 | 0 | 100 | 0 | 0 | 0 | 0 | 0 | 100 | 100 | 100 | 91 | 0 |
| Rv0807 | - | 129 | 69 | 70 | 70 | 69 | 60 | 63 | 59 | 57 | 67 | 71 | 72 | 73 | 74 | 80 | 85 | 80 | 100 | 76 | 76 | 76 | 76 | 78 | 100 | 100 | 100 | 84 | 77 |
| Rv0808 | *purF* | 527 | 80 | 83 | 82 | 80 | 77 | 80 | 77 | 78 | 88 | 87 | 87 | 87 | 91 | 96 | 96 | 96 | 100 | 92 | 92 | 92 | 92 | 92 | 100 | 100 | 100 | 95 | 93 |
| Rv0809 | *purM* | 364 | 82 | 83 | 80 | 83 | 82 | 81 | 81 | 73 | 91 | 90 | 89 | 89 | 86 | 94 | 94 | 94 | 100 | 93 | 95 | 95 | 95 | 94 | 100 | 100 | 100 | 94 | 94 |
| Rv0810c | - | 60 | 68 | 61 | 68 | 67 | 74 | 68 | 64 | 67 | 69 | 64 | 69 | 69 | 76 | 82 | 80 | 82 | 100 | 83 | 81 | 81 | 81 | 78 | 100 | 100 | 100 | 80 | 85 |
| Rv0811c | - | 368 | 54 | 54 | 54 | 56 | 52 | 51 | 52 | 49 | 69 | 70 | 72 | 72 | 69 | 87 | 89 | 87 | 100 | 80 | 80 | 79 | 79 | 80 | 100 | 100 | 100 | 87 | 81 |
| Rv0812 | - | 289 | 52 | 57 | 51 | 51 | 62 | 60 | 62 | 39 | 62 | 69 | 69 | 69 | 73 | 89 | 89 | 88 | 100 | 82 | 79 | 79 | 79 | 78 | 100 | 100 | 100 | 88 | 78 |
| Rv0813c | - | 226 | 61 | 62 | 64 | 63 | 66 | 65 | 62 | 52 | 72 | 75 | 79 | 76 | 83 | 91 | 90 | 94 | 100 | 88 | 88 | 88 | 88 | 85 | 100 | 100 | 100 | 92 | 90 |
| Rv0814c | *sseC2* | 100 | 0 | 0 | 0 | 0 | 0 | 0 | 0 | 69 | 83 | 82 | 79 | 79 | 91 | 96 | 95 | 96 | 100 | 94 | 93 | 93 | 93 | 92 | 100 | 100 | 100 | 95 | 93 |
| Rv0815c | *cysA2* | 277 | 59 | 61 | 64 | 63 | 59 | 60 | 43 | 86 | 90 | 87 | 86 | 89 | 92 | 93 | 93 | 93 | 100 | 90 | 91 | 91 | 91 | 93 | 100 | 100 | 100 | 93 | 92 |
| Rv0816c | *thiX* | 140 | 0 | 0 | 0 | 0 | 0 | 0 | 0 | 0 | 56 | 53 | 51 | 51 | 63 | 75 | 75 | 75 | 100 | 67 | 68 | 68 | 68 | 66 | 100 | 100 | 100 | 70 | 69 |
| Rv0817c | - | 270 | 0 | 0 | 0 | 0 | 43 | 0 | 0 | 0 | 50 | 53 | 54 | 54 | 72 | 83 | 85 | 83 | 100 | 73 | 75 | 75 | 75 | 77 | 100 | 100 | 100 | 85 | 75 |
| Rv0818 | - | 255 | 52 | 53 | 57 | 55 | 56 | 69 | 48 | 73 | 85 | 85 | 85 | 85 | 84 | 90 | 94 | 90 | 99 | 88 | 85 | 85 | 85 | 89 | 100 | 100 | 100 | 93 | 84 |
| Rv0819 | - | 315 | 46 | 45 | 48 | 45 | 43 | 47 | 44 | 53 | 61 | 59 | 58 | 57 | 61 | 79 | 79 | 79 | 100 | 67 | 70 | 70 | 70 | 70 | 100 | 100 | 100 | 78 | 68 |
| Rv0820 | *phoT* | 258 | 85 | 83 | 87 | 87 | 87 | 86 | 86 | 81 | 90 | 91 | 91 | 92 | 91 | 96 | 97 | 96 | 99 | 93 | 93 | 93 | 93 | 93 | 100 | 100 | 100 | 97 | 93 |
| Rv0821c | *phoY2* | 213 | 64 | 63 | 63 | 66 | 62 | 66 | 60 | 62 | 85 | 87 | 88 | 88 | 93 | 96 | 96 | 96 | 100 | 94 | 96 | 96 | 96 | 93 | 100 | 100 | 100 | 98 | 94 |
| Rv0822c | - | 684 | 47 | 45 | 43 | 44 | 44 | 48 | 45 | 45 | 54 | 54 | 57 | 59 | 66 | 74 | 74 | 74 | 99 | 68 | 68 | 69 | 69 | 65 | 100 | 100 | 100 | 80 | 66 |
| Rv0823c | - | 389 | 78 | 75 | 78 | 78 | 80 | 76 | 79 | 67 | 75 | 83 | 84 | 84 | 85 | 92 | 93 | 93 | 99 | 88 | 88 | 88 | 88 | 87 | 100 | 100 | 100 | 92 | 88 |
| Rv0824c | *desA1* | 338 | 0 | 0 | 0 | 0 | 0 | 0 | 0 | 0 | 73 | 74 | 76 | 76 | 82 | 91 | 97 | 91 | 100 | 85 | 89 | 89 | 89 | 91 | 99 | 100 | 100 | 96 | 86 |
| Rv0825c | - | 213 | 0 | 0 | 0 | 0 | 0 | 0 | 0 | 44 | 51 | 65 | 67 | 68 | 70 | 88 | 90 | 88 | 100 | 80 | 86 | 86 | 86 | 84 | 100 | 100 | 99 | 90 | 81 |
| Rv0826 | - | 351 | 0 | 0 | 0 | 0 | 0 | 0 | 0 | 0 | 58 | 79 | 76 | 76 | 84 | 87 | 88 | 88 | 99 | 76 | 78 | 78 | 78 | 78 | 100 | 100 | 100 | 90 | 76 |
| Rv0827c | - | 130 | 0 | 0 | 0 | 71 | 0 | 68 | 0 | 0 | 58 | 85 | 89 | 88 | 87 | 87 | 91 | 87 | 100 | 90 | 83 | 82 | 82 | 52 | 100 | 100 | 100 | 91 | 90 |
| Rv0828c | - | 140 | 0 | 0 | 49 | 0 | 0 | 0 | 0 | 0 | 0 | 0 | 0 | 0 | 77 | 78 | 81 | 78 | 100 | 0 | 0 | 0 | 0 | 0 | 100 | 100 | 100 | 86 | 0 |
| Rv0829 | - | 96 | 0 | 0 | 0 | 0 | 0 | 0 | 0 | 0 | 0 | 0 | 0 | 0 | 0 | 0 | 0 | 0 | 98 | 0 | 0 | 0 | 0 | 0 | 98 | 100 | 98 | 0 | 0 |
| Rv0830 | - | 301 | 0 | 0 | 0 | 0 | 0 | 0 | 0 | 0 | 50 | 56 | 56 | 0 | 61 | 86 | 88 | 84 | 100 | 65 | 68 | 68 | 68 | 66 | 100 | 100 | 99 | 86 | 64 |
| Rv0831c | - | 271 | 0 | 0 | 0 | 0 | 0 | 0 | 0 | 0 | 58 | 0 | 0 | 0 | 0 | 0 | 0 | 0 | 100 | 0 | 0 | 0 | 0 | 0 | 100 | 100 | 100 | 90 | 0 |
| Rv0832 | *PE_PGRS12* | 137 | 0 | 0 | 0 | 0 | 0 | 0 | 0 | 0 | 0 | 0 | 0 | 0 | 0 | 58 | 76 | 60 | 100 | 0 | 0 | 0 | 0 | 0 | 100 | 100 | 100 | 76 | 0 |
| Rv0833 | *PE_PGRS13* | 749 | 46 | 44 | 34 | 34 | 37 | 34 | 38 | 36 | 41 | 40 | 42 | 41 | 42 | 33 | 64 | 34 | 95 | 53 | 48 | 50 | 50 | 43 | 99 | 99 | 99 | 62 | 52 |
| Rv0834c | *PE_PGRS14* | 882 | 43 | 32 | 36 | 33 | 37 | 36 | 37 | 37 | 39 | 38 | 40 | 40 | 41 | 47 | 63 | 31 | 91 | 50 | 46 | 45 | 45 | 41 | 99 | 100 | 100 | 51 | 48 |
| Rv0835 | *lpqQ* | 214 | 0 | 0 | 0 | 0 | 0 | 0 | 0 | 0 | 0 | 0 | 0 | 0 | 0 | 54 | 55 | 55 | 99 | 0 | 0 | 0 | 0 | 0 | 100 | 100 | 100 | 74 | 0 |

| | | | | | | | | | | | | | | | | | | | | | | | | | | | | |
|---|---|---|---|---|---|---|---|---|---|---|---|---|---|---|---|---|---|---|---|---|---|---|---|---|---|---|---|---|
| Rv0836c | - | 217 | 0 | 51 | 0 | 51 | 0 | 0 | 0 | 0 | 0 | 0 | 0 | 0 | 0 | 0 | 0 | 0 | 100 | 0 | 0 | 0 | 0 | 0 | 100 | 100 | 100 | 0 | 0 |
| Rv0837c | - | 342 | 0 | 48 | 0 | 49 | 0 | 0 | 0 | 0 | 0 | 0 | 0 | 0 | 0 | 0 | 0 | 0 | 100 | 0 | 0 | 0 | 0 | 0 | 100 | 100 | 100 | 0 | 0 |
| Rv0838 | *lpqR* | 256 | 0 | 0 | 0 | 0 | 0 | 0 | 0 | 0 | 0 | 0 | 0 | 0 | 79 | 83 | 75 | 84 | 99 | 77 | 0 | 0 | 0 | 73 | 100 | 100 | 100 | 74 | 75 |
| Rv0839 | - | 270 | 0 | 0 | 0 | 0 | 0 | 0 | 50 | 44 | 44 | 0 | 74 | 72 | 54 | 84 | 87 | 84 | 100 | 0 | 54 | 54 | 54 | 57 | 100 | 100 | 100 | 87 | 39 |
| Rv0840c | *pip* | 286 | 0 | 0 | 0 | 0 | 0 | 0 | 0 | 0 | 0 | 0 | 0 | 38 | 0 | 81 | 84 | 81 | 100 | 0 | 0 | 0 | 0 | 57 | 100 | 100 | 100 | 82 | 0 |
| Rv0841 | - | 80 | 0 | 0 | 0 | 0 | 0 | 0 | 0 | 0 | 0 | 0 | 0 | 0 | 0 | 0 | 72 | 0 | 98 | 0 | 0 | 0 | 0 | 0 | 100 | 100 | 100 | 69 | 0 |
| Rv0842 | - | 430 | 0 | 0 | 0 | 0 | 0 | 0 | 0 | 42 | 0 | 0 | 0 | 0 | 47 | 0 | 0 | 0 | 100 | 38 | 0 | 0 | 0 | 0 | 100 | 100 | 100 | 73 | 0 |
| Rv0843 | - | 334 | 0 | 0 | 0 | 0 | 0 | 49 | 0 | 48 | 45 | 46 | 45 | 44 | 45 | 53 | 63 | 53 | 99 | 0 | 47 | 47 | 47 | 44 | 100 | 100 | 100 | 54 | 47 |
| Rv0844c | *narL* | 216 | 56 | 57 | 54 | 54 | 54 | 50 | 44 | 56 | 58 | 81 | 85 | 85 | 85 | 92 | 94 | 92 | 99 | 84 | 53 | 54 | 54 | 86 | 100 | 100 | 100 | 94 | 56 |
| Rv0845 | - | 425 | 47 | 41 | 0 | 43 | 0 | 0 | 41 | 48 | 43 | 47 | 50 | 50 | 47 | 81 | 82 | 82 | 99 | 50 | 45 | 44 | 44 | 50 | 100 | 100 | 100 | 81 | 48 |
| Rv0846c | - | 504 | 0 | 55 | 39 | 56 | 55 | 56 | 0 | 59 | 0 | 52 | 57 | 58 | 57 | 76 | 74 | 74 | 100 | 0 | 73 | 0 | 0 | 0 | 100 | 100 | 100 | 78 | 73 |
| Rv0847 | *lpqS* | 130 | 0 | 0 | 0 | 0 | 0 | 0 | 0 | 0 | 0 | 0 | 0 | 0 | 0 | 0 | 71 | 0 | 99 | 50 | 45 | 45 | 45 | 0 | 100 | 100 | 100 | 70 | 46 |
| Rv0848 | *cysK2* | 372 | 48 | 47 | 46 | 49 | 48 | 45 | 47 | 46 | 45 | 46 | 46 | 64 | 80 | 46 | 46 | 46 | 99 | 80 | 83 | 82 | 82 | 45 | 99 | 100 | 99 | 87 | 82 |
| Rv0849 | - | 419 | 0 | 52 | 0 | 0 | 0 | 50 | 0 | 0 | 0 | 51 | 0 | 59 | 76 | 0 | 85 | 0 | 100 | 79 | 81 | 81 | 81 | 0 | 100 | 100 | 100 | 85 | 78 |
| Rv0850 | - | 110 | 0 | 0 | 0 | 0 | 0 | 0 | 0 | 0 | 0 | 0 | 0 | 0 | 0 | 0 | 0 | 0 | 100 | 0 | 0 | 0 | 0 | 0 | 100 | 100 | 99 | 0 | 0 |
| Rv0851c | - | 275 | 48 | 47 | 51 | 50 | 50 | 52 | 46 | 55 | 55 | 48 | 55 | 55 | 53 | 86 | 85 | 86 | 99 | 54 | 50 | 50 | 50 | 55 | 100 | 100 | 100 | 84 | 59 |
| Rv0852 | *fadD16* | 278 | 0 | 0 | 0 | 0 | 0 | 0 | 0 | 0 | 0 | 0 | 0 | 0 | 0 | 86 | 85 | 86 | 100 | 64 | 67 | 67 | 67 | 66 | 100 | 100 | 100 | 78 | 66 |
| Rv0853c | *pdc* | 560 | 55 | 42 | 41 | 42 | 40 | 72 | 41 | 41 | 41 | 40 | 40 | 41 | 40 | 92 | 92 | 92 | 100 | 42 | 42 | 42 | 42 | 85 | 100 | 100 | 100 | 92 | 41 |
| Rv0854 | - | 147 | 0 | 0 | 0 | 0 | 0 | 0 | 0 | 53 | 58 | 65 | 67 | 67 | 82 | 87 | 88 | 88 | 100 | 77 | 79 | 79 | 79 | 76 | 100 | 100 | 100 | 84 | 77 |
| Rv0855 | *far* | 359 | 64 | 0 | 42 | 0 | 46 | 0 | 39 | 65 | 70 | 72 | 72 | 72 | 80 | 90 | 95 | 90 | 100 | 70 | 68 | 68 | 68 | 86 | 100 | 100 | 100 | 92 | 72 |
| Rv0856 | - | 134 | 0 | 0 | 0 | 0 | 0 | 0 | 0 | 0 | 0 | 62 | 61 | 61 | 60 | 85 | 90 | 85 | 100 | 74 | 77 | 77 | 77 | 59 | 100 | 100 | 100 | 90 | 75 |
| Rv0857 | - | 157 | 0 | 0 | 0 | 0 | 0 | 0 | 0 | 45 | 50 | 61 | 60 | 60 | 61 | 86 | 91 | 88 | 100 | 76 | 77 | 77 | 77 | 60 | 100 | 100 | 100 | 74 | 75 |
| Rv0858c | - | 397 | 64 | 70 | 68 | 67 | 41 | 64 | 43 | 67 | 74 | 75 | 73 | 73 | 78 | 91 | 91 | 91 | 100 | 84 | 84 | 85 | 85 | 84 | 100 | 100 | 100 | 91 | 86 |
| Rv0859 | *fadA* | 403 | 56 | 0 | 54 | 50 | 79 | 79 | 79 | 81 | 88 | 87 | 88 | 88 | 93 | 95 | 96 | 94 | 99 | 92 | 92 | 93 | 93 | 92 | 99 | 100 | 99 | 96 | 93 |
| Rv0860 | *fadB* | 720 | 48 | 0 | 43 | 0 | 70 | 71 | 71 | 75 | 83 | 83 | 83 | 83 | 86 | 91 | 92 | 92 | 99 | 88 | 90 | 89 | 89 | 89 | 99 | 100 | 100 | 94 | 88 |
| Rv0861c | *ercc3* | 542 | 84 | 87 | 87 | 88 | 88 | 87 | 87 | 86 | 94 | 93 | 91 | 91 | 95 | 98 | 97 | 98 | 99 | 96 | 96 | 96 | 96 | 97 | 99 | 100 | 100 | 97 | 97 |
| Rv0862c | - | 756 | 47 | 49 | 52 | 52 | 40 | 38 | 42 | 50 | 62 | 63 | 62 | 63 | 69 | 88 | 88 | 88 | 99 | 75 | 78 | 78 | 78 | 78 | 99 | 100 | 100 | 87 | 76 |
| Rv0863 | - | 93 | 0 | 56 | 65 | 0 | 66 | 67 | 70 | 0 | 59 | 66 | 68 | 69 | 68 | 95 | 95 | 95 | 100 | 70 | 77 | 77 | 77 | 84 | 100 | 100 | 100 | 93 | 80 |
| Rv0864 | *moaC* | 167 | 66 | 66 | 71 | 69 | 64 | 0 | 66 | 72 | 73 | 75 | 69 | 70 | 77 | 89 | 89 | 89 | 100 | 80 | 87 | 87 | 87 | 83 | 100 | 100 | 100 | 88 | 82 |
| Rv0865 | *mog* | 160 | 59 | 58 | 57 | 56 | 57 | 55 | 54 | 72 | 69 | 64 | 66 | 69 | 79 | 90 | 91 | 90 | 100 | 80 | 80 | 80 | 80 | 81 | 100 | 100 | 100 | 91 | 81 |
| Rv0866 | *moaE2* | 141 | 57 | 62 | 57 | 58 | 60 | 0 | 58 | 63 | 61 | 62 | 65 | 63 | 77 | 90 | 90 | 90 | 100 | 82 | 85 | 85 | 85 | 81 | 100 | 100 | 100 | 90 | 82 |
| Rv0867c | *rpfA* | 407 | 59 | 66 | 51 | 59 | 59 | 56 | 69 | 62 | 70 | 68 | 68 | 68 | 52 | 75 | 66 | 66 | 67 | 57 | 58 | 58 | 58 | 57 | 100 | 100 | 100 | 79 | 56 |
| Rv0868c | *moaD2* | 92 | 56 | 0 | 0 | 0 | 0 | 0 | 0 | 0 | 0 | 0 | 0 | 0 | 66 | 77 | 80 | 79 | 100 | 74 | 73 | 73 | 73 | 70 | 100 | 100 | 100 | 77 | 75 |
| Rv0869c | *moaA* | 360 | 64 | 67 | 66 | 69 | 63 | 0 | 65 | 70 | 68 | 67 | 66 | 66 | 78 | 91 | 89 | 91 | 100 | 81 | 81 | 81 | 81 | 81 | 100 | 100 | 100 | 88 | 80 |
| Rv0870c | - | 129 | 78 | 77 | 79 | 77 | 0 | 0 | 0 | 0 | 82 | 89 | 90 | 90 | 0 | 96 | 97 | 96 | 99 | 93 | 92 | 92 | 92 | 94 | 100 | 100 | 100 | 97 | 92 |
| Rv0871 | *cspB* | 135 | 72 | 69 | 71 | 69 | 72 | 73 | 68 | 73 | 86 | 88 | 91 | 91 | 86 | 94 | 94 | 94 | 100 | 91 | 89 | 89 | 89 | 91 | 100 | 100 | 100 | 93 | 92 |
| Rv0872c | *PE_PGRS15* | 606 | 42 | 45 | 36 | 33 | 35 | 33 | 36 | 36 | 38 | 38 | 42 | 40 | 42 | 36 | 66 | 35 | 99 | 55 | 51 | 51 | 51 | 47 | 100 | 100 | 100 | 66 | 53 |
| Rv0873 | *fadE10* | 650 | 42 | 0 | 45 | 41 | 50 | 50 | 47 | 53 | 54 | 54 | 88 | 88 | 51 | 93 | 92 | 92 | 100 | 52 | 52 | 49 | 49 | 50 | 100 | 100 | 100 | 92 | 52 |

| | | | | | | | | | | | | | | | | | | | | | | | | | | | | |
|---|---|---|---|---|---|---|---|---|---|---|---|---|---|---|---|---|---|---|---|---|---|---|---|---|---|---|---|---|
| Rv0874c | - | 386 | 0 | 0 | 0 | 0 | 0 | 0 | 0 | 0 | 0 | 0 | 0 | 0 | 0 | 0 | 0 | 0 | 100 | 0 | 0 | 0 | 0 | 0 | 100 | 100 | 100 | 0 | 0 |
| Rv0875c | - | 162 | 0 | 0 | 0 | 0 | 0 | 0 | 0 | 0 | 51 | 51 | 53 | 53 | 57 | 86 | 86 | 86 | 100 | 66 | 76 | 76 | 76 | 77 | 100 | 100 | 100 | 87 | 67 |
| Rv0876c | - | 548 | 0 | 0 | 0 | 0 | 0 | 54 | 0 | 52 | 66 | 70 | 72 | 72 | 80 | 89 | 92 | 89 | 99 | 81 | 83 | 83 | 83 | 84 | 100 | 100 | 100 | 91 | 83 |
| Rv0877 | - | 262 | 54 | 55 | 0 | 0 | 53 | 0 | 55 | 58 | 73 | 74 | 77 | 77 | 77 | 88 | 88 | 89 | 100 | 81 | 85 | 85 | 85 | 85 | 100 | 100 | 100 | 87 | 83 |
| Rv0878c | *PPE13* | 443 | 0 | 0 | 0 | 0 | 0 | 0 | 0 | 0 | 39 | 36 | 38 | 36 | 52 | 60 | 68 | 61 | 99 | 47 | 50 | 48 | 48 | 54 | 100 | 99 | 99 | 71 | 48 |
| Rv0879c | - | 91 | 0 | 0 | 0 | 0 | 0 | 0 | 0 | 0 | 63 | 0 | 0 | 0 | 73 | 76 | 84 | 76 | 100 | 73 | 70 | 70 | 70 | 74 | 100 | 100 | 100 | 84 | 70 |
| Rv0880 | - | 143 | 0 | 0 | 0 | 0 | 0 | 0 | 0 | 62 | 74 | 73 | 76 | 76 | 82 | 91 | 96 | 90 | 100 | 50 | 83 | 83 | 83 | 78 | 100 | 100 | 100 | 95 | 54 |
| Rv0881 | - | 288 | 67 | 69 | 70 | 70 | 62 | 68 | 66 | 60 | 78 | 78 | 78 | 78 | 83 | 91 | 89 | 88 | 99 | 85 | 83 | 84 | 84 | 82 | 99 | 100 | 99 | 88 | 84 |
| Rv0882 | - | 94 | 0 | 0 | 0 | 0 | 0 | 0 | 0 | 0 | 64 | 63 | 67 | 70 | 73 | 85 | 84 | 85 | 100 | 87 | 82 | 82 | 82 | 83 | 100 | 100 | 100 | 84 | 88 |
| Rv0883c | - | 253 | 60 | 45 | 53 | 52 | 47 | 56 | 47 | 41 | 64 | 59 | 64 | 65 | 71 | 83 | 81 | 81 | 100 | 74 | 72 | 72 | 72 | 75 | 99 | 100 | 100 | 84 | 73 |
| Rv0884c | *serC* | 376 | 75 | 75 | 76 | 75 | 74 | 73 | 76 | 77 | 84 | 84 | 83 | 83 | 86 | 92 | 93 | 92 | 100 | 88 | 90 | 90 | 90 | 88 | 100 | 100 | 100 | 93 | 89 |
| Rv0885 | - | 340 | 0 | 0 | 0 | 0 | 0 | 0 | 0 | 0 | 72 | 80 | 47 | 47 | 79 | 94 | 95 | 94 | 100 | 89 | 88 | 88 | 88 | 89 | 100 | 100 | 100 | 95 | 89 |
| Rv0886 | *fprB* | 575 | 54 | 51 | 55 | 54 | 49 | 54 | 48 | 57 | 67 | 67 | 62 | 62 | 70 | 86 | 86 | 86 | 100 | 79 | 80 | 80 | 80 | 83 | 100 | 100 | 100 | 86 | 81 |
| Rv0887c | - | 152 | 0 | 0 | 0 | 0 | 0 | 0 | 0 | 46 | 59 | 54 | 48 | 50 | 80 | 91 | 91 | 91 | 100 | 80 | 84 | 85 | 85 | 81 | 100 | 100 | 100 | 93 | 78 |
| Rv0888 | - | 490 | 0 | 0 | 0 | 0 | 0 | 0 | 0 | 0 | 0 | 0 | 0 | 0 | 0 | 0 | 79 | 0 | 100 | 43 | 0 | 0 | 0 | 0 | 100 | 100 | 100 | 74 | 0 |
| Rv0889c | *citA* | 373 | 48 | 45 | 46 | 44 | 44 | 45 | 45 | 80 | 93 | 93 | 93 | 93 | 92 | 96 | 97 | 96 | 100 | 93 | 96 | 96 | 96 | 95 | 100 | 100 | 100 | 94 | 94 |
| Rv0890c | - | 882 | 0 | 57 | 0 | 60 | 0 | 0 | 0 | 0 | 44 | 51 | 50 | 53 | 0 | 38 | 65 | 36 | 99 | 47 | 38 | 38 | 38 | 39 | 99 | 99 | 99 | 50 | 68 |
| Rv0891c | - | 285 | 0 | 0 | 0 | 0 | 0 | 0 | 0 | 0 | 0 | 0 | 0 | 0 | 0 | 53 | 53 | 53 | 99 | 55 | 50 | 51 | 51 | 55 | 99 | 100 | 99 | 55 | 69 |
| Rv0892 | - | 495 | 52 | 0 | 43 | 46 | 44 | 42 | 41 | 56 | 56 | 54 | 57 | 57 | 67 | 55 | 84 | 55 | 100 | 54 | 57 | 57 | 57 | 55 | 100 | 100 | 100 | 82 | 66 |
| Rv0893c | - | 325 | 0 | 0 | 0 | 0 | 0 | 0 | 0 | 0 | 45 | 54 | 57 | 0 | 68 | 76 | 76 | 78 | 100 | 60 | 72 | 72 | 72 | 71 | 100 | 100 | 100 | 75 | 60 |
| Rv0894 | - | 393 | 0 | 0 | 0 | 0 | 0 | 0 | 0 | 0 | 57 | 58 | 57 | 57 | 0 | 0 | 66 | 0 | 100 | 0 | 0 | 0 | 0 | 0 | 100 | 100 | 100 | 58 | 68 |
| Rv0895 | - | 505 | 0 | 0 | 0 | 0 | 0 | 0 | 0 | 57 | 55 | 55 | 65 | 64 | 52 | 55 | 56 | 55 | 100 | 51 | 54 | 54 | 54 | 56 | 100 | 100 | 100 | 54 | 52 |
| Rv0896 | *gltA* | 431 | 77 | 78 | 77 | 77 | 76 | 77 | 78 | 69 | 85 | 86 | 86 | 86 | 89 | 94 | 93 | 93 | 99 | 91 | 89 | 89 | 89 | 92 | 100 | 100 | 100 | 97 | 90 |
| Rv0897c | - | 535 | 0 | 0 | 0 | 0 | 0 | 0 | 0 | 64 | 64 | 0 | 40 | 40 | 70 | 83 | 84 | 83 | 100 | 76 | 76 | 76 | 76 | 78 | 100 | 100 | 99 | 84 | 77 |
| Rv0898c | - | 87 | 0 | 0 | 0 | 0 | 0 | 0 | 0 | 63 | 61 | 0 | 68 | 0 | 0 | 84 | 88 | 84 | 100 | 71 | 76 | 76 | 76 | 76 | 100 | 100 | 100 | 88 | 75 |
| Rv0899 | *ompA* | 326 | 0 | 0 | 0 | 0 | 0 | 0 | 0 | 0 | 0 | 0 | 0 | 0 | 0 | 0 | 0 | 0 | 100 | 0 | 0 | 0 | 0 | 0 | 100 | 100 | 100 | 78 | 0 |
| Rv0900 | - | 50 | 0 | 0 | 0 | 0 | 0 | 0 | 0 | 0 | 0 | 0 | 0 | 0 | 0 | 0 | 0 | 0 | 100 | 0 | 0 | 0 | 0 | 0 | 100 | 100 | 100 | 0 | 0 |
| Rv0901 | - | 175 | 0 | 0 | 0 | 0 | 0 | 0 | 0 | 0 | 0 | 0 | 0 | 0 | 0 | 0 | 56 | 0 | 100 | 0 | 0 | 0 | 0 | 0 | 100 | 100 | 100 | 59 | 0 |
| Rv0902c | *prrB* | 446 | 48 | 44 | 46 | 48 | 41 | 40 | 44 | 50 | 61 | 59 | 60 | 60 | 73 | 89 | 97 | 91 | 100 | 86 | 90 | 90 | 90 | 90 | 100 | 100 | 100 | 97 | 89 |
| Rv0903c | *prrA* | 236 | 64 | 64 | 62 | 65 | 62 | 62 | 63 | 65 | 83 | 85 | 80 | 79 | 94 | 98 | 98 | 98 | 100 | 96 | 96 | 96 | 96 | 95 | 100 | 100 | 100 | 97 | 95 |
| Rv0904c | *accD3* | 495 | 38 | 64 | 62 | 64 | 39 | 62 | 40 | 71 | 72 | 79 | 77 | 76 | 38 | 89 | 80 | 80 | 100 | 83 | 84 | 84 | 84 | 84 | 100 | 100 | 100 | 87 | 84 |
| Rv0905 | *echA6* | 243 | 47 | 56 | 54 | 40 | 59 | 56 | 55 | 46 | 68 | 70 | 69 | 69 | 80 | 96 | 96 | 96 | 100 | 83 | 83 | 83 | 83 | 84 | 100 | 100 | 100 | 96 | 84 |
| Rv0906 | - | 372 | 0 | 0 | 0 | 0 | 0 | 0 | 0 | 0 | 66 | 68 | 69 | 69 | 72 | 84 | 91 | 85 | 100 | 76 | 77 | 77 | 77 | 78 | 100 | 100 | 100 | 90 | 77 |
| Rv0907 | - | 532 | 0 | 0 | 0 | 0 | 0 | 0 | 0 | 0 | 68 | 71 | 74 | 73 | 41 | 83 | 86 | 83 | 99 | 73 | 73 | 73 | 73 | 74 | 99 | 99 | 99 | 85 | 72 |
| Rv0908 | *ctpE* | 797 | 43 | 44 | 42 | 42 | 65 | 70 | 65 | 44 | 79 | 80 | 80 | 80 | 82 | 90 | 91 | 90 | 100 | 86 | 87 | 87 | 87 | 87 | 99 | 100 | 100 | 91 | 85 |
| Rv0909 | - | 59 | 0 | 0 | 0 | 0 | 0 | 0 | 0 | 0 | 0 | 0 | 0 | 0 | 0 | 76 | 84 | 76 | 98 | 72 | 0 | 0 | 0 | 74 | 100 | 100 | 100 | 84 | 78 |
| Rv0910 | - | 144 | 0 | 0 | 0 | 0 | 0 | 0 | 0 | 0 | 60 | 66 | 62 | 62 | 78 | 91 | 91 | 91 | 100 | 86 | 83 | 83 | 83 | 86 | 100 | 100 | 100 | 90 | 83 |
| Rv0911 | - | 257 | 49 | 51 | 50 | 51 | 48 | 46 | 48 | 59 | 51 | 63 | 60 | 60 | 65 | 0 | 85 | 0 | 100 | 52 | 0 | 0 | 0 | 74 | 100 | 100 | 100 | 85 | 77 |

| | | | | | | | | | | | | | | | | | | | | | | | | | | | | |
|---|---|---|---|---|---|---|---|---|---|---|---|---|---|---|---|---|---|---|---|---|---|---|---|---|---|---|---|---|
| Rv0912 | - | 149 | 0 | 0 | 0 | 0 | 0 | 0 | 0 | 0 | 0 | 0 | 0 | 0 | 62 | 83 | 83 | 83 | 100 | 68 | 71 | 71 | 71 | 73 | 100 | 100 | 100 | 81 | 71 |
| Rv0913c | - | 502 | 0 | 0 | 0 | 0 | 0 | 0 | 0 | 0 | 45 | 86 | 45 | 46 | 41 | 42 | 41 | 41 | 99 | 84 | 80 | 80 | 80 | 85 | 100 | 100 | 100 | 91 | 80 |
| Rv0914c | - | 412 | 0 | 0 | 40 | 0 | 0 | 0 | 0 | 46 | 40 | 81 | 43 | 45 | 43 | 43 | 92 | 47 | 100 | 85 | 81 | 81 | 81 | 86 | 100 | 100 | 100 | 91 | 83 |
| Rv0915c | *PPE14* | 423 | 0 | 0 | 0 | 0 | 0 | 0 | 0 | 0 | 38 | 44 | 36 | 37 | 42 | 55 | 78 | 55 | 100 | 53 | 41 | 41 | 41 | 41 | 100 | 100 | 100 | 62 | 43 |
| Rv0916c | *PE7* | 99 | 0 | 0 | 0 | 0 | 0 | 0 | 0 | 0 | 0 | 0 | 0 | 0 | 0 | 68 | 68 | 68 | 100 | 0 | 0 | 0 | 0 | 0 | 100 | 100 | 100 | 68 | 0 |
| Rv0917 | *betP* | 593 | 58 | 60 | 58 | 60 | 61 | 59 | 59 | 61 | 61 | 78 | 62 | 61 | 0 | 0 | 92 | 0 | 100 | 62 | 60 | 60 | 60 | 63 | 100 | 100 | 100 | 92 | 57 |
| Rv0918 | - | 158 | 0 | 0 | 0 | 0 | 0 | 0 | 0 | 0 | 0 | 0 | 0 | 0 | 0 | 0 | 0 | 0 | 99 | 78 | 0 | 0 | 0 | 0 | 100 | 100 | 100 | 0 | 0 |
| Rv0919 | - | 166 | 0 | 0 | 0 | 0 | 0 | 0 | 0 | 0 | 0 | 0 | 0 | 0 | 0 | 0 | 0 | 0 | 100 | 84 | 0 | 0 | 0 | 0 | 100 | 100 | 100 | 0 | 0 |
| Rv0920c | - | 439 | 64 | 53 | 59 | 0 | 61 | 0 | 61 | 48 | 0 | 43 | 79 | 49 | 0 | 49 | 69 | 69 | 100 | 80 | 79 | 79 | 79 | 50 | 100 | 100 | 100 | 82 | 67 |
| Rv0921 | - | 193 | 0 | 0 | 0 | 0 | 0 | 0 | 0 | 0 | 0 | 0 | 0 | 0 | 0 | 0 | 0 | 0 | 100 | 0 | 0 | 0 | 0 | 0 | 100 | 100 | 100 | 0 | 0 |
| Rv0922 | - | 550 | 0 | 0 | 0 | 0 | 0 | 0 | 0 | 42 | 41 | 0 | 52 | 38 | 0 | 0 | 0 | 0 | 100 | 0 | 0 | 0 | 0 | 0 | 100 | 100 | 100 | 0 | 0 |
| Rv0923c | - | 354 | 0 | 0 | 0 | 0 | 0 | 0 | 0 | 0 | 0 | 0 | 0 | 0 | 0 | 84 | 85 | 83 | 100 | 82 | 82 | 82 | 82 | 79 | 100 | 100 | 100 | 80 | 83 |
| Rv0924c | *mntH* | 428 | 70 | 0 | 0 | 0 | 70 | 73 | 0 | 41 | 0 | 78 | 76 | 77 | 79 | 87 | 92 | 87 | 100 | 83 | 82 | 82 | 82 | 83 | 100 | 100 | 100 | 92 | 0 |
| Rv0925c | - | 245 | 0 | 0 | 0 | 0 | 0 | 0 | 0 | 51 | 49 | 0 | 88 | 87 | 56 | 0 | 93 | 0 | 100 | 49 | 50 | 50 | 50 | 50 | 100 | 100 | 100 | 92 | 54 |
| Rv0926c | - | 358 | 0 | 0 | 0 | 0 | 0 | 0 | 0 | 66 | 78 | 58 | 71 | 71 | 78 | 92 | 96 | 92 | 100 | 85 | 87 | 87 | 87 | 86 | 100 | 100 | 100 | 84 | 87 |
| Rv0927c | - | 263 | 50 | 44 | 50 | 50 | 54 | 46 | 50 | 72 | 80 | 55 | 54 | 54 | 79 | 94 | 95 | 95 | 100 | 92 | 89 | 89 | 89 | 89 | 100 | 100 | 100 | 93 | 91 |
| Rv0928 | *pstS3* | 370 | 53 | 58 | 55 | 56 | 58 | 57 | 52 | 47 | 77 | 64 | 63 | 64 | 83 | 86 | 89 | 86 | 100 | 79 | 79 | 79 | 79 | 61 | 100 | 100 | 100 | 89 | 80 |
| Rv0929 | *pstC2* | 324 | 68 | 65 | 68 | 70 | 68 | 69 | 61 | 62 | 84 | 75 | 76 | 76 | 86 | 91 | 93 | 92 | 100 | 86 | 86 | 86 | 86 | 75 | 100 | 100 | 100 | 93 | 86 |
| Rv0930 | *pstA1* | 308 | 72 | 71 | 70 | 72 | 69 | 68 | 64 | 69 | 82 | 77 | 77 | 77 | 86 | 92 | 91 | 92 | 99 | 85 | 85 | 85 | 85 | 77 | 99 | 99 | 99 | 91 | 84 |
| Rv0931c | *pknD* | 664 | 59 | 54 | 51 | 53 | 46 | 56 | 46 | 46 | 64 | 58 | 55 | 51 | 59 | 76 | 78 | 77 | 100 | 65 | 68 | 68 | 68 | 71 | 100 | 100 | 100 | 75 | 66 |
| Rv0932c | *pstS2* | 370 | 55 | 57 | 56 | 56 | 57 | 55 | 52 | 48 | 75 | 64 | 63 | 64 | 79 | 85 | 84 | 86 | 100 | 78 | 76 | 76 | 76 | 61 | 100 | 100 | 100 | 83 | 79 |
| Rv0933 | *pstB* | 276 | 64 | 60 | 64 | 61 | 65 | 64 | 64 | 63 | 63 | 64 | 63 | 63 | 62 | 63 | 64 | 62 | 100 | 62 | 62 | 62 | 62 | 61 | 100 | 100 | 100 | 64 | 62 |
| Rv0934 | *pstS1* | 374 | 41 | 44 | 42 | 42 | 41 | 44 | 42 | 42 | 42 | 44 | 43 | 42 | 44 | 44 | 44 | 44 | 99 | 45 | 44 | 44 | 44 | 46 | 100 | 100 | 100 | 44 | 45 |
| Rv0935 | *pstC1* | 338 | 50 | 50 | 50 | 48 | 50 | 49 | 53 | 53 | 48 | 49 | 48 | 49 | 52 | 51 | 52 | 52 | 100 | 50 | 49 | 49 | 49 | 51 | 100 | 100 | 100 | 52 | 50 |
| Rv0936 | *pstA2* | 301 | 48 | 47 | 49 | 50 | 48 | 50 | 49 | 52 | 51 | 52 | 51 | 51 | 48 | 46 | 49 | 46 | 100 | 47 | 47 | 47 | 47 | 49 | 100 | 100 | 100 | 49 | 47 |
| Rv0937c | - | 273 | 0 | 0 | 0 | 0 | 0 | 0 | 0 | 68 | 73 | 67 | 69 | 68 | 89 | 94 | 94 | 94 | 100 | 91 | 92 | 92 | 92 | 91 | 100 | 100 | 100 | 93 | 89 |
| Rv0938 | - | 759 | 0 | 0 | 0 | 0 | 0 | 0 | 0 | 63 | 62 | 69 | 69 | 70 | 76 | 87 | 88 | 86 | 99 | 79 | 79 | 80 | 80 | 81 | 99 | 100 | 99 | 88 | 80 |
| Rv0939 | - | 644 | 45 | 44 | 45 | 45 | 47 | 45 | 42 | 51 | 45 | 52 | 50 | 47 | 50 | 87 | 88 | 87 | 100 | 46 | 46 | 46 | 46 | 48 | 100 | 100 | 100 | 89 | 43 |
| Rv0940c | - | 288 | 0 | 0 | 0 | 0 | 0 | 0 | 0 | 78 | 84 | 60 | 63 | 63 | 89 | 93 | 93 | 93 | 100 | 92 | 91 | 91 | 91 | 91 | 100 | 100 | 100 | 93 | 90 |
| Rv0941c | - | 257 | 0 | 0 | 0 | 0 | 0 | 0 | 0 | 45 | 49 | 0 | 0 | 0 | 0 | 78 | 85 | 78 | 100 | 60 | 0 | 0 | 0 | 67 | 100 | 100 | 100 | 85 | 64 |
| Rv0942 | - | 92 | 0 | 0 | 0 | 0 | 0 | 0 | 0 | 0 | 0 | 0 | 0 | 0 | 0 | 0 | 0 | 0 | 100 | 0 | 0 | 0 | 0 | 0 | 100 | 100 | 100 | 0 | 0 |
| Rv0943c | - | 346 | 46 | 0 | 0 | 0 | 0 | 0 | 0 | 53 | 40 | 45 | 50 | 50 | 40 | 74 | 72 | 73 | 100 | 38 | 40 | 40 | 40 | 62 | 100 | 100 | 100 | 72 | 36 |
| Rv0944 | - | 158 | 41 | 52 | 48 | 51 | 48 | 0 | 45 | 68 | 81 | 81 | 83 | 82 | 84 | 89 | 88 | 89 | 100 | 85 | 87 | 87 | 87 | 90 | 100 | 100 | 100 | 87 | 88 |
| Rv0945 | - | 253 | 48 | 48 | 52 | 50 | 53 | 51 | 53 | 49 | 74 | 72 | 71 | 72 | 80 | 94 | 93 | 94 | 99 | 86 | 92 | 91 | 91 | 86 | 100 | 100 | 100 | 92 | 86 |
| Rv0946c | *pgi* | 553 | 74 | 75 | 73 | 73 | 71 | 76 | 66 | 0 | 84 | 83 | 86 | 86 | 89 | 95 | 95 | 95 | 100 | 89 | 91 | 91 | 91 | 91 | 100 | 100 | 100 | 94 | 89 |
| Rv0948c | - | 105 | 75 | 79 | 77 | 78 | 78 | 75 | 74 | 0 | 85 | 80 | 89 | 89 | 88 | 84 | 90 | 84 | 100 | 89 | 81 | 81 | 81 | 78 | 100 | 100 | 99 | 90 | 74 |
| Rv0949 | *uvrD1* | 771 | 73 | 74 | 74 | 74 | 69 | 69 | 75 | 69 | 79 | 78 | 78 | 79 | 82 | 94 | 93 | 94 | 100 | 90 | 89 | 88 | 88 | 89 | 100 | 100 | 100 | 93 | 90 |
| Rv0950c | - | 332 | 58 | 63 | 59 | 60 | 56 | 55 | 61 | 47 | 61 | 63 | 64 | 64 | 62 | 78 | 79 | 76 | 100 | 62 | 64 | 64 | 64 | 63 | 100 | 100 | 99 | 78 | 61 |

| | | | | | | | | | | | | | | | | | | | | | | | | | | | | | |
|---|---|---|---|---|---|---|---|---|---|---|---|---|---|---|---|---|---|---|---|---|---|---|---|---|---|---|---|---|---|
| Rv0951 | *sucC* | 387 | 71 | 0 | 75 | 75 | 0 | 0 | 0 | 77 | 91 | 88 | 88 | 88 | 89 | 93 | 95 | 93 | 99 | 93 | 92 | 92 | 92 | 92 | 100 | 100 | 100 | 95 | 93 |
| Rv0952 | *sucD* | 303 | 79 | 0 | 83 | 84 | 0 | 0 | 0 | 84 | 93 | 91 | 93 | 93 | 93 | 95 | 95 | 95 | 100 | 92 | 94 | 94 | 94 | 93 | 100 | 100 | 100 | 95 | 92 |
| Rv0953c | - | 282 | 0 | 0 | 46 | 45 | 0 | 0 | 44 | 77 | 82 | 80 | 84 | 84 | 83 | 92 | 93 | 92 | 100 | 91 | 90 | 90 | 90 | 90 | 100 | 100 | 100 | 92 | 92 |
| Rv0954 | - | 303 | 0 | 0 | 0 | 0 | 0 | 0 | 0 | 0 | 39 | 45 | 41 | 43 | 42 | 70 | 80 | 70 | 100 | 51 | 56 | 56 | 56 | 49 | 99 | 100 | 100 | 79 | 50 |
| Rv0955 | - | 455 | 43 | 47 | 46 | 46 | 42 | 47 | 47 | 38 | 55 | 52 | 53 | 53 | 70 | 80 | 80 | 81 | 100 | 73 | 74 | 74 | 74 | 73 | 100 | 100 | 100 | 79 | 73 |
| Rv0956 | *purN* | 215 | 66 | 68 | 69 | 71 | 71 | 62 | 66 | 66 | 77 | 77 | 75 | 76 | 81 | 89 | 89 | 89 | 100 | 81 | 83 | 83 | 83 | 81 | 100 | 100 | 100 | 89 | 83 |
| Rv0957 | *purH* | 523 | 73 | 78 | 79 | 78 | 78 | 75 | 79 | 75 | 81 | 81 | 82 | 82 | 82 | 92 | 92 | 92 | 100 | 85 | 88 | 88 | 88 | 88 | 100 | 100 | 100 | 91 | 85 |
| Rv0958 | - | 459 | 0 | 0 | 78 | 77 | 0 | 0 | 0 | 82 | 82 | 86 | 86 | 86 | 0 | 95 | 95 | 95 | 99 | 93 | 93 | 93 | 93 | 93 | 99 | 100 | 99 | 96 | 92 |
| Rv0959 | - | 672 | 0 | 0 | 71 | 69 | 0 | 0 | 0 | 78 | 73 | 82 | 83 | 83 | 0 | 95 | 94 | 95 | 100 | 91 | 92 | 92 | 92 | 92 | 99 | 100 | 100 | 94 | 90 |
| Rv0960 | - | 127 | 0 | 0 | 0 | 0 | 0 | 0 | 0 | 0 | 0 | 0 | 0 | 0 | 0 | 0 | 0 | 0 | 100 | 51 | 0 | 0 | 0 | 0 | 100 | 100 | 100 | 0 | 0 |
| Rv0961 | - | 115 | 0 | 0 | 0 | 0 | 0 | 0 | 0 | 0 | 0 | 0 | 0 | 0 | 0 | 0 | 0 | 0 | 100 | 0 | 0 | 0 | 0 | 0 | 100 | 100 | 100 | 0 | 0 |
| Rv0962c | *lprP* | 224 | 0 | 0 | 0 | 0 | 0 | 0 | 0 | 0 | 0 | 0 | 0 | 0 | 0 | 0 | 0 | 0 | 99 | 0 | 0 | 0 | 0 | 0 | 99 | 100 | 99 | 0 | 0 |
| Rv0963c | - | 266 | 46 | 0 | 0 | 40 | 0 | 0 | 46 | 0 | 0 | 50 | 47 | 47 | 0 | 42 | 52 | 41 | 100 | 0 | 43 | 43 | 43 | 48 | 100 | 100 | 100 | 52 | 41 |
| Rv0964c | - | 160 | 0 | 0 | 0 | 0 | 0 | 0 | 0 | 0 | 0 | 0 | 0 | 0 | 0 | 0 | 0 | 0 | 99 | 0 | 0 | 0 | 0 | 0 | 99 | 100 | 99 | 0 | 0 |
| Rv0965c | - | 139 | 0 | 0 | 0 | 0 | 0 | 0 | 0 | 0 | 0 | 0 | 0 | 0 | 0 | 0 | 54 | 0 | 99 | 0 | 0 | 0 | 0 | 0 | 100 | 100 | 100 | 54 | 0 |
| Rv0966c | - | 200 | 0 | 0 | 43 | 44 | 0 | 0 | 0 | 52 | 42 | 0 | 0 | 48 | 78 | 88 | 89 | 88 | 99 | 80 | 78 | 78 | 78 | 71 | 99 | 99 | 99 | 89 | 79 |
| Rv0967 | - | 119 | 62 | 0 | 0 | 55 | 0 | 0 | 0 | 0 | 0 | 0 | 0 | 0 | 0 | 60 | 90 | 60 | 100 | 0 | 61 | 61 | 61 | 61 | 100 | 100 | 100 | 90 | 58 |
| Rv0968 | - | 98 | 0 | 0 | 0 | 0 | 0 | 0 | 0 | 0 | 0 | 0 | 0 | 64 | 0 | 62 | 73 | 62 | 100 | 0 | 0 | 0 | 0 | 68 | 100 | 100 | 100 | 68 | 0 |
| Rv0969 | *ctpV* | 770 | 61 | 64 | 66 | 64 | 63 | 64 | 61 | 59 | 66 | 66 | 66 | 67 | 62 | 59 | 87 | 62 | 100 | 64 | 64 | 64 | 64 | 66 | 100 | 100 | 100 | 84 | 66 |
| Rv0970 | - | 210 | 0 | 0 | 0 | 0 | 0 | 0 | 0 | 0 | 0 | 0 | 0 | 0 | 0 | 52 | 75 | 52 | 100 | 0 | 0 | 0 | 0 | 0 | 100 | 100 | 100 | 54 | 0 |
| Rv0971c | *echA7* | 269 | 45 | 42 | 44 | 40 | 45 | 43 | 44 | 61 | 73 | 75 | 74 | 74 | 78 | 88 | 87 | 87 | 100 | 83 | 82 | 82 | 82 | 84 | 100 | 100 | 100 | 88 | 84 |
| Rv0972c | *fadE12* | 388 | 47 | 0 | 47 | 0 | 49 | 48 | 61 | 80 | 86 | 84 | 82 | 84 | 87 | 95 | 97 | 96 | 99 | 92 | 92 | 92 | 92 | 92 | 100 | 100 | 99 | 97 | 94 |
| Rv0973c | *accA2* | 667 | 57 | 50 | 58 | 51 | 55 | 49 | 49 | 63 | 77 | 79 | 77 | 76 | 77 | 87 | 90 | 87 | 99 | 80 | 81 | 81 | 81 | 79 | 100 | 100 | 100 | 90 | 82 |
| Rv0974c | *accD2* | 529 | 47 | 52 | 52 | 51 | 64 | 52 | 49 | 83 | 87 | 89 | 90 | 89 | 92 | 95 | 96 | 96 | 99 | 91 | 94 | 93 | 93 | 93 | 99 | 100 | 99 | 97 | 92 |
| Rv0975c | *fadE13* | 382 | 46 | 0 | 47 | 44 | 53 | 56 | 56 | 71 | 86 | 87 | 86 | 86 | 91 | 95 | 93 | 96 | 100 | 90 | 92 | 92 | 92 | 91 | 100 | 100 | 100 | 93 | 91 |
| Rv0976c | - | 560 | 0 | 0 | 0 | 0 | 0 | 0 | 0 | 68 | 83 | 83 | 84 | 83 | 86 | 91 | 93 | 91 | 100 | 83 | 88 | 88 | 88 | 85 | 100 | 100 | 100 | 93 | 87 |
| Rv0977 | *PE_PGRS16* | 923 | 43 | 43 | 38 | 33 | 33 | 34 | 35 | 35 | 38 | 40 | 40 | 41 | 41 | 47 | 53 | 35 | 100 | 57 | 54 | 55 | 55 | 43 | 100 | 99 | 100 | 49 | 54 |
| Rv0978c | *PE_PGRS17* | 331 | 0 | 0 | 0 | 0 | 38 | 0 | 36 | 0 | 46 | 0 | 63 | 61 | 38 | 50 | 63 | 45 | 95 | 58 | 52 | 59 | 59 | 51 | 95 | 100 | 95 | 55 | 49 |
| Rv0979A | *rpmF* | 57 | 75 | 75 | 75 | 75 | 74 | 76 | 76 | 0 | 79 | 81 | 84 | 79 | 85 | 91 | 87 | 89 | 100 | 84 | 89 | 89 | 89 | 82 | 100 | 100 | 100 | 85 | 85 |
| Rv0979c | - | 64 | 0 | 0 | 0 | 0 | 0 | 0 | 0 | 0 | 0 | 0 | 0 | 0 | 0 | 0 | 0 | 0 | 100 | 0 | 0 | 0 | 0 | 0 | 100 | 100 | 100 | 0 | 0 |
| Rv0980c | *PE_PGRS18* | 457 | 45 | 42 | 40 | 37 | 38 | 0 | 39 | 42 | 50 | 35 | 60 | 64 | 45 | 48 | 65 | 46 | 99 | 54 | 48 | 44 | 44 | 46 | 96 | 100 | 99 | 58 | 44 |
| Rv0981 | *mprA* | 230 | 83 | 86 | 83 | 83 | 85 | 67 | 85 | 82 | 92 | 93 | 92 | 92 | 95 | 98 | 98 | 98 | 99 | 93 | 95 | 95 | 95 | 94 | 100 | 100 | 100 | 96 | 95 |
| Rv0982 | *mprB* | 504 | 61 | 58 | 60 | 62 | 57 | 44 | 54 | 58 | 68 | 73 | 73 | 72 | 79 | 89 | 90 | 89 | 99 | 83 | 87 | 87 | 87 | 86 | 100 | 100 | 100 | 90 | 84 |
| Rv0983 | *pepD* | 464 | 58 | 60 | 56 | 66 | 56 | 59 | 55 | 63 | 67 | 61 | 64 | 64 | 70 | 79 | 81 | 79 | 99 | 72 | 75 | 75 | 75 | 75 | 99 | 100 | 99 | 81 | 73 |
| Rv0984 | *moaB2* | 181 | 69 | 76 | 68 | 78 | 76 | 73 | 73 | 66 | 83 | 84 | 86 | 86 | 87 | 96 | 97 | 97 | 100 | 89 | 91 | 91 | 91 | 91 | 100 | 100 | 100 | 97 | 90 |
| Rv0985c | *mscL* | 151 | 64 | 63 | 62 | 66 | 65 | 60 | 66 | 51 | 65 | 64 | 59 | 61 | 65 | 84 | 86 | 84 | 100 | 77 | 79 | 78 | 78 | 74 | 100 | 100 | 100 | 86 | 74 |
| Rv0986 | - | 248 | 63 | 66 | 64 | 64 | 63 | 65 | 62 | 63 | 65 | 65 | 65 | 63 | 63 | 64 | 62 | 64 | 100 | 61 | 60 | 60 | 60 | 61 | 100 | 100 | 100 | 61 | 63 |
| Rv0987 | - | 855 | 43 | 43 | 40 | 41 | 0 | 41 | 41 | 42 | 43 | 43 | 42 | 43 | 0 | 40 | 0 | 40 | 99 | 0 | 0 | 0 | 0 | 0 | 99 | 100 | 100 | 0 | 42 |

| | | | | | | | | | | | | | | | | | | | | | | | | | | | | |
|---|---|---|---|---|---|---|---|---|---|---|---|---|---|---|---|---|---|---|---|---|---|---|---|---|---|---|---|---|
| Rv0988 | - | 386 | 0 | 0 | 0 | 0 | 0 | 0 | 0 | 0 | 0 | 0 | 0 | 0 | 0 | 0 | 0 | 0 | 99 | 0 | 0 | 0 | 0 | 0 | 100 | 100 | 100 | 0 | 0 |
| Rv0989c | *grcC2* | 325 | 57 | 57 | 58 | 58 | 58 | 54 | 58 | 55 | 63 | 63 | 64 | 63 | 67 | 72 | 69 | 72 | 100 | 72 | 73 | 73 | 73 | 74 | 99 | 100 | 100 | 69 | 72 |
| Rv0990c | - | 218 | 47 | 0 | 47 | 46 | 0 | 0 | 0 | 53 | 57 | 55 | 61 | 61 | 71 | 80 | 84 | 80 | 100 | 74 | 77 | 76 | 76 | 78 | 100 | 100 | 99 | 82 | 76 |
| Rv0991c | - | 110 | 0 | 0 | 0 | 0 | 0 | 0 | 0 | 70 | 72 | 68 | 71 | 70 | 77 | 81 | 86 | 81 | 100 | 78 | 77 | 79 | 79 | 75 | 100 | 100 | 100 | 86 | 79 |
| Rv0992c | - | 197 | 48 | 47 | 48 | 47 | 53 | 43 | 50 | 51 | 59 | 60 | 61 | 59 | 61 | 85 | 77 | 86 | 100 | 70 | 69 | 69 | 69 | 69 | 100 | 100 | 100 | 78 | 69 |
| Rv0993 | *galU* | 306 | 73 | 73 | 74 | 75 | 72 | 71 | 76 | 0 | 85 | 87 | 86 | 86 | 89 | 92 | 95 | 92 | 100 | 93 | 92 | 92 | 92 | 90 | 100 | 100 | 100 | 95 | 92 |
| Rv0994 | *moeA1* | 426 | 74 | 75 | 76 | 75 | 72 | 69 | 70 | 52 | 84 | 84 | 85 | 85 | 85 | 92 | 94 | 92 | 99 | 90 | 90 | 90 | 90 | 89 | 100 | 100 | 100 | 94 | 90 |
| Rv0995 | *rimJ* | 203 | 60 | 59 | 60 | 60 | 64 | 60 | 56 | 57 | 76 | 79 | 80 | 79 | 87 | 94 | 93 | 95 | 100 | 89 | 86 | 86 | 86 | 83 | 100 | 100 | 100 | 93 | 89 |
| Rv0996 | - | 358 | 42 | 45 | 43 | 44 | 41 | 47 | 40 | 0 | 44 | 45 | 45 | 45 | 61 | 74 | 68 | 75 | 99 | 59 | 66 | 66 | 66 | 62 | 100 | 100 | 100 | 69 | 62 |
| Rv0997 | - | 143 | 0 | 0 | 0 | 0 | 0 | 0 | 0 | 0 | 0 | 0 | 0 | 0 | 0 | 0 | 0 | 0 | 100 | 0 | 0 | 0 | 0 | 0 | 100 | 100 | 100 | 63 | 0 |
| Rv0998 | - | 333 | 0 | 0 | 0 | 0 | 0 | 0 | 0 | 47 | 51 | 52 | 53 | 53 | 68 | 85 | 85 | 85 | 100 | 75 | 75 | 75 | 75 | 73 | 100 | 100 | 100 | 87 | 77 |
| Rv0999 | - | 252 | 0 | 0 | 0 | 0 | 0 | 0 | 0 | 0 | 0 | 0 | 0 | 0 | 57 | 79 | 81 | 79 | 100 | 67 | 65 | 64 | 64 | 73 | 100 | 100 | 100 | 81 | 66 |
| Rv1000c | - | 205 | 0 | 0 | 0 | 0 | 0 | 0 | 0 | 73 | 75 | 73 | 72 | 74 | 77 | 91 | 92 | 91 | 100 | 82 | 85 | 85 | 85 | 83 | 100 | 100 | 100 | 91 | 81 |
| Rv1001 | *arcA* | 402 | 53 | 0 | 0 | 0 | 0 | 58 | 0 | 71 | 80 | 85 | 84 | 84 | 84 | 94 | 93 | 94 | 100 | 86 | 87 | 87 | 87 | 88 | 100 | 100 | 100 | 93 | 86 |
| Rv1002c | - | 503 | 61 | 60 | 62 | 60 | 60 | 66 | 59 | 49 | 74 | 78 | 76 | 77 | 80 | 91 | 93 | 91 | 100 | 90 | 89 | 88 | 88 | 86 | 100 | 100 | 100 | 93 | 89 |
| Rv1003 | - | 285 | 65 | 69 | 67 | 66 | 63 | 69 | 64 | 69 | 75 | 75 | 74 | 73 | 75 | 87 | 87 | 87 | 100 | 78 | 82 | 82 | 82 | 80 | 100 | 100 | 100 | 87 | 80 |
| Rv1004c | - | 419 | 0 | 0 | 39 | 41 | 34 | 36 | 34 | 36 | 36 | 0 | 35 | 35 | 36 | 41 | 58 | 41 | 100 | 37 | 36 | 36 | 36 | 37 | 98 | 100 | 100 | 58 | 37 |
| Rv1005c | *pabB* | 458 | 46 | 51 | 51 | 48 | 50 | 56 | 44 | 40 | 47 | 46 | 45 | 46 | 70 | 82 | 84 | 82 | 100 | 79 | 76 | 77 | 77 | 72 | 99 | 100 | 100 | 85 | 74 |
| Rv1006 | - | 567 | 0 | 0 | 0 | 0 | 0 | 0 | 0 | 0 | 0 | 0 | 0 | 0 | 0 | 80 | 79 | 79 | 99 | 0 | 0 | 0 | 0 | 0 | 100 | 100 | 100 | 84 | 0 |
| Rv1007c | *metG* | 519 | 43 | 44 | 42 | 43 | 43 | 41 | 41 | 41 | 80 | 81 | 82 | 82 | 84 | 90 | 91 | 90 | 99 | 85 | 85 | 85 | 85 | 86 | 99 | 100 | 99 | 90 | 81 |
| Rv1008 | *tatD* | 264 | 60 | 60 | 61 | 60 | 58 | 61 | 60 | 60 | 79 | 78 | 80 | 79 | 82 | 88 | 91 | 89 | 99 | 86 | 85 | 85 | 85 | 85 | 99 | 100 | 99 | 90 | 88 |
| Rv1009 | *rpfB* | 362 | 54 | 55 | 52 | 51 | 54 | 52 | 54 | 52 | 65 | 69 | 68 | 68 | 79 | 92 | 92 | 92 | 99 | 81 | 85 | 85 | 85 | 85 | 100 | 100 | 100 | 91 | 83 |
| Rv1010 | *ksgA* | 317 | 66 | 68 | 70 | 72 | 64 | 69 | 63 | 65 | 74 | 71 | 72 | 72 | 84 | 89 | 89 | 89 | 100 | 85 | 89 | 89 | 89 | 84 | 100 | 100 | 100 | 89 | 85 |
| Rv1011 | *ispE* | 306 | 58 | 60 | 56 | 56 | 55 | 62 | 59 | 63 | 80 | 79 | 78 | 78 | 85 | 93 | 95 | 93 | 100 | 89 | 86 | 86 | 86 | 87 | 100 | 100 | 100 | 95 | 89 |
| Rv1012 | - | 97 | 0 | 0 | 0 | 0 | 0 | 0 | 0 | 0 | 0 | 0 | 0 | 0 | 0 | 0 | 0 | 0 | 100 | 0 | 0 | 0 | 0 | 0 | 100 | 100 | 100 | 0 | 0 |
| Rv1013 | *pks16* | 544 | 43 | 47 | 45 | 43 | 46 | 37 | 41 | 43 | 75 | 77 | 79 | 79 | 87 | 97 | 92 | 97 | 100 | 94 | 96 | 96 | 96 | 95 | 100 | 100 | 100 | 92 | 95 |
| Rv1014c | *pth* | 191 | 70 | 66 | 69 | 69 | 67 | 67 | 67 | 71 | 79 | 79 | 80 | 79 | 83 | 94 | 92 | 94 | 100 | 84 | 90 | 90 | 90 | 91 | 100 | 100 | 100 | 92 | 85 |
| Rv1015c | *rplY* | 215 | 65 | 62 | 68 | 65 | 66 | 67 | 63 | 70 | 76 | 74 | 74 | 75 | 82 | 85 | 86 | 85 | 100 | 81 | 82 | 82 | 82 | 81 | 100 | 100 | 100 | 86 | 81 |
| Rv1016c | *lpqT* | 226 | 0 | 0 | 0 | 0 | 0 | 0 | 0 | 0 | 0 | 0 | 0 | 0 | 49 | 77 | 73 | 77 | 98 | 69 | 70 | 70 | 70 | 73 | 100 | 100 | 100 | 73 | 73 |
| Rv1017c | *prsA* | 326 | 79 | 83 | 83 | 83 | 83 | 84 | 83 | 78 | 89 | 89 | 89 | 89 | 92 | 98 | 97 | 98 | 100 | 91 | 93 | 93 | 93 | 93 | 100 | 100 | 100 | 96 | 92 |
| Rv1018c | *glmU* | 495 | 73 | 74 | 76 | 76 | 75 | 69 | 74 | 70 | 73 | 75 | 76 | 77 | 82 | 89 | 89 | 89 | 99 | 86 | 86 | 86 | 86 | 84 | 100 | 100 | 100 | 88 | 84 |
| Rv1019 | - | 197 | 75 | 74 | 73 | 76 | 0 | 53 | 0 | 51 | 83 | 87 | 87 | 87 | 92 | 98 | 98 | 98 | 100 | 94 | 98 | 98 | 98 | 95 | 100 | 100 | 100 | 99 | 94 |
| Rv1020 | *mfd* | 1234 | 70 | 71 | 71 | 71 | 70 | 71 | 69 | 67 | 80 | 81 | 80 | 81 | 83 | 92 | 92 | 93 | 100 | 87 | 88 | 88 | 88 | 88 | 100 | 100 | 100 | 91 | 87 |
| Rv1021 | - | 325 | 59 | 56 | 48 | 49 | 72 | 51 | 47 | 50 | 57 | 59 | 60 | 61 | 73 | 85 | 77 | 85 | 100 | 81 | 81 | 81 | 81 | 83 | 100 | 99 | 100 | 77 | 81 |
| Rv1022 | *lpqU* | 243 | 56 | 60 | 62 | 62 | 57 | 56 | 57 | 60 | 65 | 61 | 65 | 66 | 73 | 89 | 92 | 88 | 99 | 81 | 81 | 81 | 81 | 81 | 100 | 100 | 100 | 92 | 82 |
| Rv1023 | *eno* | 429 | 81 | 83 | 81 | 82 | 83 | 81 | 81 | 85 | 90 | 89 | 89 | 89 | 91 | 96 | 95 | 96 | 99 | 92 | 92 | 92 | 92 | 94 | 100 | 100 | 100 | 95 | 93 |
| Rv1024 | - | 228 | 0 | 43 | 45 | 45 | 44 | 51 | 41 | 44 | 51 | 49 | 47 | 47 | 64 | 77 | 79 | 77 | 100 | 65 | 68 | 68 | 68 | 67 | 100 | 100 | 100 | 79 | 63 |
| Rv1025 | - | 155 | 72 | 68 | 71 | 70 | 71 | 72 | 69 | 61 | 81 | 80 | 79 | 79 | 89 | 91 | 91 | 91 | 100 | 86 | 88 | 88 | 88 | 88 | 100 | 100 | 100 | 92 | 87 |

| | | | | | | | | | | | | | | | | | | | | | | | | | | | | |
|---|---|---|---|---|---|---|---|---|---|---|---|---|---|---|---|---|---|---|---|---|---|---|---|---|---|---|---|---|---|
| Rv1026 | - | 319 | 66 | 66 | 66 | 65 | 65 | 64 | 64 | 60 | 73 | 73 | 76 | 76 | 77 | 85 | 87 | 84 | 100 | 80 | 80 | 80 | 80 | 78 | 100 | 100 | 100 | 85 | 79 |
| Rv1027c | *kdpE* | 226 | 59 | 58 | 61 | 63 | 70 | 60 | 63 | 73 | 84 | 90 | 90 | 90 | 88 | 96 | 91 | 96 | 100 | 58 | 92 | 92 | 92 | 92 | 100 | 100 | 100 | 58 | 91 |
| Rv1028A | *kdpF* | 30 | 0 | 0 | 0 | 0 | 0 | 0 | 0 | 0 | 0 | 0 | 0 | 0 | 0 | 0 | 0 | 0 | 100 | 0 | 0 | 0 | 0 | 0 | 100 | 100 | 100 | 0 | 0 |
| Rv1028c | *kdpD* | 860 | 48 | 49 | 49 | 48 | 55 | 43 | 35 | 59 | 75 | 80 | 81 | 81 | 75 | 89 | 89 | 89 | 99 | 49 | 87 | 87 | 87 | 86 | 99 | 100 | 99 | 44 | 88 |
| Rv1029 | *kdpA* | 571 | 0 | 0 | 0 | 0 | 64 | 0 | 0 | 0 | 65 | 64 | 64 | 65 | 65 | 66 | 66 | 66 | 100 | 0 | 65 | 65 | 65 | 67 | 100 | 100 | 100 | 0 | 67 |
| Rv1030 | *kdpB* | 709 | 44 | 44 | 44 | 48 | 73 | 45 | 46 | 45 | 77 | 78 | 78 | 79 | 76 | 71 | 79 | 77 | 100 | 47 | 78 | 78 | 78 | 80 | 100 | 100 | 100 | 45 | 79 |
| Rv1031 | *kdpC* | 189 | 0 | 0 | 0 | 0 | 60 | 0 | 0 | 0 | 50 | 54 | 55 | 55 | 56 | 48 | 48 | 48 | 100 | 0 | 69 | 69 | 69 | 68 | 100 | 100 | 100 | 0 | 72 |
| Rv1032c | *trcS* | 509 | 57 | 52 | 48 | 51 | 53 | 51 | 49 | 50 | 53 | 46 | 46 | 52 | 54 | 83 | 86 | 82 | 100 | 51 | 54 | 54 | 54 | 68 | 100 | 100 | 100 | 86 | 68 |
| Rv1033c | *trcR* | 257 | 70 | 69 | 69 | 70 | 69 | 69 | 68 | 73 | 69 | 68 | 68 | 68 | 78 | 91 | 95 | 91 | 100 | 75 | 77 | 77 | 77 | 83 | 100 | 100 | 100 | 95 | 86 |
| Rv1034c | - | 129 | 0 | 0 | 0 | 0 | 0 | 0 | 0 | 0 | 0 | 0 | 0 | 0 | 0 | 0 | 0 | 0 | 100 | 0 | 0 | 0 | 0 | 0 | 100 | 100 | 100 | 0 | 0 |
| Rv1035c | - | 228 | 0 | 0 | 0 | 0 | 0 | 0 | 0 | 0 | 0 | 0 | 0 | 0 | 0 | 0 | 0 | 0 | 100 | 0 | 0 | 0 | 0 | 0 | 100 | 100 | 100 | 0 | 0 |
| Rv1036c | - | 112 | 0 | 0 | 0 | 0 | 0 | 0 | 0 | 0 | 0 | 0 | 0 | 0 | 0 | 0 | 0 | 0 | 100 | 0 | 0 | 0 | 0 | 0 | 100 | 100 | 100 | 0 | 0 |
| Rv1037c | *esxI* | 94 | 0 | 0 | 0 | 0 | 0 | 0 | 0 | 0 | 0 | 0 | 0 | 0 | 0 | 91 | 93 | 91 | 98 | 0 | 0 | 0 | 0 | 0 | 100 | 100 | 100 | 93 | 0 |
| Rv1038c | *esxJ* | 98 | 0 | 0 | 0 | 0 | 0 | 0 | 0 | 0 | 0 | 0 | 0 | 0 | 0 | 93 | 93 | 93 | 100 | 0 | 0 | 0 | 0 | 0 | 100 | 100 | 100 | 96 | 0 |
| Rv1039c | *PPE15* | 391 | 0 | 0 | 0 | 0 | 0 | 0 | 33 | 0 | 38 | 41 | 37 | 40 | 41 | 66 | 66 | 66 | 100 | 44 | 44 | 44 | 44 | 42 | 100 | 100 | 99 | 85 | 44 |
| Rv1040c | *PE8* | 275 | 0 | 0 | 0 | 0 | 0 | 0 | 0 | 0 | 40 | 0 | 0 | 0 | 0 | 58 | 84 | 58 | 100 | 0 | 43 | 43 | 43 | 0 | 100 | 100 | 100 | 84 | 0 |
| Rv1041c | - | 287 | 0 | 0 | 0 | 0 | 0 | 0 | 0 | 0 | 51 | 0 | 0 | 0 | 0 | 0 | 0 | 0 | 100 | 50 | 0 | 0 | 0 | 0 | 100 | 100 | 100 | 0 | 50 |
| Rv1042c | - | 135 | 0 | 0 | 0 | 0 | 0 | 0 | 0 | 0 | 63 | 0 | 0 | 0 | 0 | 0 | 0 | 0 | 100 | 61 | 0 | 0 | 0 | 0 | 100 | 100 | 100 | 0 | 61 |
| Rv1043c | - | 341 | 0 | 0 | 0 | 0 | 0 | 0 | 0 | 0 | 0 | 51 | 49 | 49 | 0 | 0 | 0 | 0 | 99 | 0 | 0 | 0 | 0 | 0 | 100 | 100 | 100 | 0 | 0 |
| Rv1044 | - | 207 | 0 | 42 | 0 | 0 | 0 | 0 | 0 | 0 | 41 | 41 | 0 | 0 | 0 | 0 | 0 | 46 | 100 | 0 | 0 | 0 | 0 | 0 | 100 | 100 | 100 | 0 | 0 |
| Rv1045 | - | 293 | 0 | 0 | 0 | 0 | 0 | 0 | 0 | 0 | 0 | 0 | 0 | 0 | 0 | 0 | 0 | 0 | 99 | 0 | 0 | 0 | 0 | 0 | 100 | 100 | 100 | 0 | 0 |
| Rv1046c | - | 174 | 0 | 0 | 0 | 0 | 0 | 0 | 0 | 0 | 0 | 0 | 0 | 0 | 0 | 0 | 0 | 0 | 100 | 0 | 0 | 0 | 0 | 0 | 100 | 100 | 100 | 0 | 0 |
| Rv1047 | - | 415 | 45 | 66 | 74 | 0 | 73 | 0 | 69 | 59 | 0 | 40 | 87 | 58 | 0 | 60 | 0 | 79 | 99 | 89 | 42 | 79 | 79 | 59 | 100 | 100 | 100 | 43 | 89 |
| Rv1048c | - | 371 | 0 | 0 | 0 | 0 | 0 | 0 | 0 | 0 | 0 | 0 | 0 | 0 | 0 | 0 | 0 | 0 | 100 | 0 | 0 | 0 | 0 | 0 | 100 | 100 | 100 | 0 | 0 |
| Rv1049 | - | 148 | 0 | 0 | 0 | 0 | 0 | 0 | 0 | 0 | 0 | 0 | 0 | 0 | 0 | 0 | 0 | 0 | 100 | 0 | 0 | 0 | 0 | 0 | 100 | 100 | 100 | 0 | 0 |
| Rv1050 | - | 301 | 57 | 57 | 56 | 54 | 53 | 53 | 55 | 58 | 52 | 52 | 52 | 52 | 51 | 52 | 55 | 55 | 100 | 47 | 50 | 55 | 55 | 50 | 100 | 100 | 100 | 51 | 46 |
| Rv1051c | - | 251 | 0 | 0 | 0 | 0 | 0 | 0 | 0 | 0 | 0 | 0 | 0 | 0 | 0 | 0 | 91 | 75 | 100 | 67 | 93 | 95 | 95 | 0 | 100 | 100 | 100 | 0 | 0 |
| Rv1052 | - | 129 | 0 | 0 | 0 | 0 | 0 | 0 | 0 | 0 | 0 | 0 | 0 | 0 | 0 | 0 | 0 | 0 | 100 | 0 | 0 | 0 | 0 | 0 | 100 | 100 | 100 | 0 | 0 |
| Rv1053c | - | 91 | 0 | 0 | 0 | 0 | 0 | 0 | 0 | 0 | 0 | 0 | 0 | 0 | 0 | 0 | 0 | 0 | 100 | 0 | 0 | 0 | 0 | 0 | 100 | 100 | 100 | 0 | 0 |
| Rv1054 | - | 104 | 0 | 0 | 0 | 0 | 0 | 0 | 0 | 0 | 0 | 0 | 0 | 0 | 0 | 0 | 0 | 0 | 100 | 81 | 91 | 0 | 0 | 0 | 100 | 100 | 100 | 0 | 0 |
| Rv1055 | - | 44 | 0 | 0 | 0 | 0 | 0 | 0 | 0 | 0 | 0 | 0 | 0 | 0 | 0 | 0 | 0 | 0 | 100 | 0 | 0 | 0 | 0 | 0 | 100 | 100 | 100 | 0 | 0 |
| Rv1056 | - | 254 | 0 | 0 | 0 | 0 | 0 | 0 | 0 | 51 | 0 | 53 | 50 | 51 | 55 | 89 | 89 | 89 | 100 | 49 | 51 | 51 | 51 | 52 | 100 | 100 | 100 | 42 | 81 |
| Rv1057 | - | 393 | 0 | 0 | 0 | 0 | 0 | 0 | 0 | 0 | 0 | 0 | 43 | 47 | 0 | 68 | 71 | 67 | 100 | 51 | 47 | 47 | 47 | 51 | 100 | 100 | 100 | 72 | 47 |
| Rv1058 | *fadD14* | 543 | 59 | 57 | 60 | 60 | 55 | 54 | 54 | 49 | 70 | 70 | 63 | 65 | 77 | 91 | 90 | 91 | 100 | 73 | 85 | 85 | 85 | 84 | 100 | 100 | 100 | 91 | 63 |
| Rv1059 | - | 354 | 0 | 0 | 0 | 0 | 0 | 0 | 0 | 45 | 69 | 50 | 44 | 45 | 47 | 74 | 95 | 74 | 100 | 83 | 86 | 86 | 86 | 84 | 100 | 100 | 100 | 95 | 84 |
| Rv1060 | - | 157 | 0 | 0 | 0 | 0 | 0 | 0 | 0 | 0 | 0 | 0 | 0 | 0 | 0 | 69 | 75 | 69 | 100 | 75 | 0 | 0 | 0 | 0 | 100 | 100 | 100 | 74 | 75 |
| Rv1061 | - | 287 | 0 | 0 | 0 | 0 | 0 | 0 | 0 | 0 | 65 | 0 | 68 | 68 | 0 | 85 | 82 | 85 | 100 | 77 | 42 | 42 | 42 | 0 | 100 | 100 | 100 | 81 | 0 |
| Rv1062 | - | 285 | 0 | 0 | 0 | 0 | 0 | 0 | 0 | 45 | 0 | 64 | 41 | 66 | 44 | 82 | 84 | 83 | 100 | 76 | 78 | 78 | 78 | 77 | 100 | 100 | 100 | 82 | 75 |

| | | | | | | | | | | | | | | | | | | | | | | | | | | | | | |
|---|---|---|---|---|---|---|---|---|---|---|---|---|---|---|---|---|---|---|---|---|---|---|---|---|---|---|---|---|---|
| Rv1063c | - | 360 | 0 | 0 | 0 | 0 | 0 | 0 | 0 | 61 | 0 | 0 | 71 | 70 | 68 | 84 | 87 | 84 | 100 | 76 | 80 | 80 | 80 | 79 | 100 | 100 | 100 | 87 | 77 |
| Rv1064c | *lpqV* | 139 | 0 | 0 | 0 | 0 | 0 | 0 | 0 | 0 | 0 | 0 | 0 | 0 | 53 | 70 | 70 | 72 | 100 | 60 | 56 | 56 | 56 | 58 | 100 | 100 | 100 | 69 | 60 |
| Rv1065 | - | 188 | 0 | 0 | 0 | 0 | 0 | 0 | 0 | 0 | 71 | 75 | 76 | 76 | 81 | 88 | 87 | 88 | 100 | 81 | 78 | 78 | 78 | 75 | 100 | 100 | 100 | 87 | 76 |
| Rv1066 | - | 131 | 0 | 0 | 0 | 0 | 0 | 0 | 0 | 0 | 83 | 77 | 81 | 82 | 77 | 90 | 90 | 90 | 100 | 85 | 90 | 90 | 90 | 87 | 100 | 100 | 100 | 88 | 86 |
| Rv1067c | *PE_PGRS19* | 667 | 43 | 34 | 35 | 33 | 33 | 39 | 38 | 35 | 39 | 41 | 41 | 41 | 41 | 43 | 60 | 36 | 98 | 52 | 51 | 52 | 52 | 48 | 99 | 100 | 99 | 55 | 52 |
| Rv1068c | *PE_PGRS20* | 463 | 44 | 40 | 39 | 38 | 35 | 37 | 38 | 36 | 48 | 44 | 39 | 39 | 48 | 46 | 64 | 39 | 73 | 54 | 51 | 51 | 51 | 50 | 87 | 99 | 100 | 63 | 48 |
| Rv1069c | - | 587 | 51 | 0 | 48 | 49 | 50 | 47 | 0 | 0 | 59 | 56 | 68 | 67 | 79 | 85 | 85 | 85 | 100 | 82 | 82 | 82 | 82 | 80 | 99 | 100 | 100 | 90 | 81 |
| Rv1070c | *echA8* | 257 | 74 | 46 | 59 | 44 | 73 | 72 | 73 | 64 | 81 | 84 | 85 | 85 | 87 | 95 | 95 | 95 | 100 | 91 | 87 | 87 | 87 | 88 | 100 | 100 | 100 | 95 | 90 |
| Rv1071c | *echA9* | 345 | 64 | 54 | 55 | 55 | 52 | 52 | 48 | 61 | 68 | 72 | 70 | 71 | 79 | 87 | 89 | 87 | 100 | 79 | 82 | 82 | 82 | 81 | 100 | 100 | 100 | 88 | 81 |
| Rv1072 | - | 278 | 66 | 69 | 68 | 70 | 66 | 70 | 67 | 52 | 71 | 74 | 74 | 74 | 79 | 91 | 92 | 91 | 100 | 82 | 84 | 84 | 84 | 76 | 100 | 100 | 100 | 91 | 83 |
| Rv1073 | - | 283 | 0 | 0 | 46 | 0 | 0 | 0 | 0 | 42 | 0 | 0 | 56 | 58 | 64 | 70 | 70 | 70 | 100 | 65 | 75 | 75 | 75 | 69 | 100 | 100 | 98 | 71 | 70 |
| Rv1074c | *fadA3* | 405 | 64 | 0 | 59 | 56 | 53 | 51 | 53 | 84 | 88 | 86 | 88 | 89 | 89 | 95 | 97 | 96 | 100 | 90 | 90 | 90 | 90 | 90 | 100 | 100 | 100 | 96 | 89 |
| Rv1075c | - | 314 | 0 | 0 | 0 | 0 | 0 | 0 | 0 | 53 | 66 | 66 | 64 | 70 | 72 | 83 | 84 | 83 | 100 | 82 | 80 | 80 | 80 | 76 | 100 | 100 | 99 | 90 | 82 |
| Rv1076 | *lipU* | 297 | 45 | 0 | 0 | 0 | 42 | 0 | 0 | 46 | 52 | 52 | 58 | 58 | 71 | 89 | 93 | 89 | 100 | 80 | 78 | 78 | 78 | 80 | 100 | 100 | 100 | 92 | 81 |
| Rv1077 | *cbs* | 464 | 58 | 58 | 58 | 57 | 59 | 82 | 60 | 78 | 90 | 91 | 90 | 90 | 92 | 95 | 96 | 95 | 100 | 94 | 94 | 94 | 94 | 93 | 100 | 100 | 100 | 95 | 94 |
| Rv1078 | *pra* | 240 | 0 | 0 | 0 | 0 | 0 | 0 | 0 | 46 | 51 | 48 | 56 | 59 | 63 | 80 | 82 | 80 | 99 | 61 | 59 | 57 | 57 | 60 | 100 | 100 | 100 | 86 | 61 |
| Rv1079 | *metB* | 388 | 73 | 50 | 76 | 77 | 74 | 82 | 48 | 77 | 85 | 85 | 85 | 85 | 87 | 91 | 92 | 91 | 100 | 90 | 88 | 88 | 88 | 88 | 100 | 100 | 100 | 91 | 88 |
| Rv1080c | *greA* | 164 | 70 | 73 | 70 | 71 | 79 | 81 | 79 | 64 | 93 | 95 | 95 | 95 | 96 | 98 | 98 | 98 | 100 | 98 | 96 | 96 | 96 | 97 | 100 | 100 | 100 | 98 | 97 |
| Rv1081c | - | 144 | 47 | 54 | 56 | 56 | 50 | 49 | 51 | 0 | 60 | 56 | 57 | 58 | 65 | 79 | 86 | 80 | 100 | 68 | 73 | 73 | 73 | 70 | 100 | 100 | 100 | 86 | 71 |
| Rv1082 | *mca* | 288 | 69 | 70 | 72 | 73 | 72 | 72 | 66 | 72 | 80 | 80 | 80 | 80 | 79 | 89 | 89 | 89 | 100 | 87 | 89 | 89 | 89 | 87 | 100 | 100 | 100 | 90 | 88 |
| Rv1083 | - | 88 | 0 | 0 | 0 | 0 | 0 | 0 | 0 | 0 | 78 | 77 | 69 | 68 | 75 | 78 | 76 | 76 | 100 | 72 | 65 | 65 | 65 | 79 | 100 | 100 | 100 | 76 | 59 |
| Rv1084 | - | 673 | 0 | 0 | 0 | 0 | 0 | 0 | 0 | 62 | 74 | 73 | 74 | 73 | 77 | 88 | 87 | 88 | 100 | 79 | 82 | 83 | 83 | 80 | 100 | 100 | 100 | 87 | 81 |
| Rv1085c | - | 242 | 55 | 55 | 53 | 54 | 53 | 55 | 55 | 59 | 66 | 72 | 73 | 73 | 75 | 76 | 76 | 76 | 100 | 82 | 79 | 79 | 79 | 78 | 100 | 100 | 100 | 90 | 81 |
| Rv1086 | - | 262 | 71 | 75 | 73 | 73 | 71 | 65 | 71 | 68 | 79 | 77 | 80 | 80 | 87 | 93 | 94 | 93 | 99 | 90 | 88 | 88 | 88 | 91 | 100 | 100 | 100 | 94 | 90 |
| Rv1087 | *PE_PGRS21* | 767 | 45 | 33 | 33 | 33 | 35 | 37 | 39 | 35 | 40 | 40 | 45 | 42 | 44 | 34 | 53 | 33 | 96 | 50 | 50 | 53 | 53 | 48 | 97 | 100 | 82 | 57 | 50 |
| Rv1087A | - | 106 | 0 | 50 | 49 | 50 | 47 | 0 | 0 | 0 | 43 | 0 | 0 | 0 | 48 | 48 | 48 | 48 | 100 | 48 | 59 | 59 | 59 | 58 | 100 | 100 | 100 | 48 | 48 |
| Rv1088 | *PE9* | 144 | 0 | 0 | 0 | 0 | 0 | 0 | 0 | 0 | 0 | 0 | 0 | 0 | 0 | 73 | 70 | 73 | 100 | 0 | 0 | 0 | 0 | 0 | 100 | 100 | 100 | 70 | 0 |
| Rv1089 | *PE10* | 120 | 0 | 0 | 0 | 0 | 0 | 0 | 0 | 0 | 0 | 0 | 0 | 0 | 0 | 0 | 57 | 0 | 100 | 0 | 0 | 0 | 0 | 0 | 100 | 100 | 100 | 54 | 0 |
| Rv1089A | *celA2a* | 34 | 0 | 0 | 0 | 0 | 0 | 0 | 0 | 0 | 0 | 0 | 0 | 0 | 0 | 0 | 0 | 0 | 100 | 0 | 0 | 0 | 0 | 0 | 100 | 100 | 100 | 0 | 0 |
| Rv1090 | *celA2b* | 151 | 0 | 0 | 0 | 0 | 0 | 0 | 0 | 0 | 0 | 0 | 0 | 0 | 0 | 0 | 0 | 0 | 100 | 0 | 0 | 0 | 0 | 0 | 100 | 100 | 100 | 0 | 0 |
| Rv1091 | *PE_PGRS22* | 853 | 44 | 45 | 38 | 32 | 36 | 32 | 40 | 36 | 40 | 41 | 43 | 43 | 39 | 33 | 59 | 36 | 99 | 52 | 47 | 50 | 50 | 44 | 82 | 99 | 82 | 58 | 51 |
| Rv1092c | *coaA* | 312 | 80 | 75 | 77 | 80 | 77 | 77 | 80 | 71 | 90 | 87 | 88 | 88 | 90 | 97 | 98 | 97 | 100 | 92 | 94 | 94 | 94 | 93 | 100 | 100 | 100 | 98 | 92 |
| Rv1093 | *glyA* | 426 | 85 | 84 | 86 | 85 | 84 | 81 | 84 | 59 | 85 | 85 | 87 | 86 | 58 | 94 | 95 | 94 | 99 | 57 | 57 | 57 | 57 | 57 | 99 | 100 | 99 | 95 | 56 |
| Rv1094 | *desA2* | 275 | 0 | 0 | 0 | 0 | 0 | 0 | 0 | 0 | 56 | 53 | 57 | 58 | 76 | 90 | 90 | 90 | 100 | 86 | 87 | 87 | 87 | 87 | 100 | 100 | 100 | 90 | 87 |
| Rv1095 | *phoH2* | 433 | 80 | 53 | 79 | 78 | 80 | 54 | 63 | 85 | 91 | 91 | 92 | 92 | 94 | 97 | 97 | 97 | 100 | 95 | 95 | 95 | 95 | 96 | 100 | 100 | 100 | 94 | 95 |
| Rv1096 | - | 291 | 50 | 56 | 0 | 0 | 0 | 0 | 0 | 53 | 49 | 45 | 43 | 0 | 77 | 90 | 90 | 90 | 99 | 61 | 47 | 47 | 47 | 48 | 100 | 100 | 100 | 91 | 76 |
| Rv1097c | - | 293 | 0 | 0 | 0 | 0 | 0 | 0 | 0 | 0 | 0 | 0 | 0 | 0 | 0 | 66 | 73 | 66 | 99 | 38 | 46 | 45 | 45 | 41 | 100 | 100 | 100 | 74 | 40 |
| Rv1098c | *fumC* | 474 | 82 | 86 | 84 | 83 | 85 | 84 | 81 | 74 | 87 | 88 | 89 | 89 | 90 | 94 | 95 | 94 | 100 | 91 | 92 | 92 | 92 | 92 | 100 | 100 | 100 | 94 | 92 |

| | | | | | | | | | | | | | | | | | | | | | | | | | | | | | |
|---|---|---|---|---|---|---|---|---|---|---|---|---|---|---|---|---|---|---|---|---|---|---|---|---|---|---|---|---|---|
| Rv1099c | *glpX* | 328 | 79 | 79 | 81 | 81 | 81 | 81 | 80 | 81 | 89 | 91 | 92 | 92 | 92 | 97 | 96 | 97 | 100 | 92 | 94 | 94 | 94 | 92 | 100 | 100 | 100 | 96 | 95 |
| Rv1100 | - | 233 | 45 | 43 | 44 | 46 | 43 | 49 | 46 | 0 | 52 | 56 | 57 | 56 | 63 | 79 | 83 | 79 | 100 | 73 | 70 | 70 | 70 | 70 | 100 | 100 | 100 | 83 | 72 |
| Rv1101c | - | 385 | 0 | 0 | 0 | 0 | 0 | 0 | 0 | 0 | 0 | 0 | 0 | 0 | 77 | 0 | 89 | 0 | 100 | 68 | 80 | 80 | 80 | 82 | 100 | 100 | 100 | 88 | 69 |
| Rv1102c | - | 103 | 0 | 0 | 0 | 0 | 0 | 0 | 0 | 0 | 0 | 0 | 0 | 0 | 0 | 0 | 0 | 0 | 99 | 0 | 0 | 0 | 0 | 0 | 100 | 100 | 100 | 0 | 0 |
| Rv1103c | - | 106 | 0 | 0 | 0 | 0 | 0 | 0 | 0 | 0 | 0 | 0 | 0 | 0 | 0 | 0 | 0 | 0 | 100 | 0 | 0 | 0 | 0 | 0 | 96 | 100 | 100 | 0 | 0 |
| Rv1104 | - | 229 | 53 | 52 | 53 | 54 | 53 | 52 | 43 | 0 | 64 | 70 | 69 | 68 | 61 | 83 | 79 | 82 | 100 | 80 | 79 | 79 | 79 | 73 | 99 | 100 | 100 | 76 | 77 |
| Rv1105 | - | 171 | 0 | 50 | 0 | 0 | 44 | 0 | 0 | 0 | 55 | 61 | 64 | 64 | 51 | 84 | 86 | 84 | 100 | 72 | 69 | 69 | 69 | 71 | 100 | 100 | 100 | 85 | 71 |
| Rv1106c | - | 370 | 0 | 0 | 44 | 44 | 0 | 43 | 0 | 0 | 47 | 53 | 43 | 42 | 47 | 91 | 91 | 92 | 100 | 88 | 87 | 87 | 87 | 86 | 100 | 100 | 100 | 91 | 85 |
| Rv1107c | *xseB* | 85 | 71 | 71 | 70 | 76 | 64 | 64 | 68 | 70 | 81 | 80 | 79 | 78 | 76 | 87 | 87 | 87 | 100 | 86 | 82 | 82 | 82 | 81 | 100 | 100 | 100 | 85 | 82 |
| Rv1108c | *xseA* | 415 | 72 | 76 | 74 | 76 | 70 | 67 | 71 | 66 | 77 | 79 | 79 | 79 | 84 | 92 | 90 | 92 | 100 | 87 | 82 | 82 | 82 | 86 | 100 | 100 | 100 | 89 | 86 |
| Rv1109c | - | 212 | 0 | 0 | 0 | 0 | 0 | 0 | 0 | 0 | 47 | 51 | 53 | 52 | 58 | 81 | 79 | 81 | 100 | 68 | 69 | 69 | 69 | 73 | 100 | 100 | 100 | 79 | 73 |
| Rv1110 | *ispH* | 335 | 82 | 79 | 78 | 80 | 80 | 0 | 78 | 79 | 86 | 91 | 88 | 88 | 90 | 93 | 94 | 94 | 100 | 91 | 91 | 91 | 91 | 92 | 100 | 100 | 100 | 94 | 91 |
| Rv1111c | - | 327 | 0 | 0 | 0 | 0 | 0 | 0 | 0 | 0 | 46 | 45 | 46 | 44 | 49 | 54 | 61 | 54 | 100 | 49 | 52 | 53 | 53 | 50 | 99 | 100 | 100 | 58 | 52 |
| Rv1112 | - | 357 | 81 | 82 | 84 | 84 | 84 | 84 | 83 | 79 | 90 | 88 | 89 | 89 | 92 | 94 | 95 | 95 | 100 | 92 | 93 | 93 | 93 | 92 | 100 | 100 | 100 | 95 | 93 |
| Rv1113 | - | 65 | 0 | 0 | 0 | 0 | 0 | 0 | 0 | 0 | 0 | 0 | 0 | 0 | 0 | 88 | 88 | 88 | 100 | 0 | 0 | 0 | 0 | 0 | 100 | 100 | 100 | 0 | 0 |
| Rv1114 | - | 124 | 0 | 0 | 0 | 0 | 0 | 0 | 0 | 0 | 0 | 0 | 0 | 0 | 0 | 78 | 0 | 78 | 100 | 0 | 0 | 0 | 0 | 0 | 100 | 100 | 100 | 0 | 0 |
| Rv1115 | - | 232 | 52 | 0 | 0 | 0 | 0 | 0 | 0 | 0 | 0 | 47 | 0 | 0 | 60 | 69 | 80 | 68 | 99 | 68 | 68 | 68 | 68 | 70 | 100 | 100 | 100 | 69 | 67 |
| Rv1116 | - | 61 | 0 | 0 | 0 | 0 | 0 | 0 | 0 | 0 | 0 | 0 | 0 | 0 | 0 | 0 | 0 | 0 | 100 | 0 | 0 | 0 | 0 | 0 | 100 | 100 | 100 | 0 | 0 |
| Rv1116A | - | 91 | 0 | 0 | 0 | 0 | 0 | 0 | 0 | 0 | 0 | 0 | 0 | 0 | 0 | 0 | 0 | 0 | 100 | 0 | 0 | 0 | 0 | 0 | 100 | 100 | 100 | 70 | 0 |
| Rv1117 | - | 107 | 60 | 64 | 66 | 66 | 66 | 66 | 63 | 0 | 0 | 0 | 72 | 72 | 0 | 94 | 95 | 95 | 100 | 0 | 0 | 0 | 0 | 75 | 100 | 100 | 100 | 97 | 0 |
| Rv1118c | - | 286 | 0 | 0 | 0 | 0 | 0 | 0 | 0 | 59 | 67 | 68 | 61 | 62 | 85 | 75 | 90 | 75 | 100 | 81 | 85 | 85 | 85 | 86 | 100 | 100 | 100 | 90 | 83 |
| Rv1119c | - | 49 | 0 | 0 | 0 | 0 | 0 | 0 | 0 | 0 | 0 | 0 | 0 | 0 | 0 | 0 | 62 | 0 | 100 | 0 | 0 | 0 | 0 | 0 | 100 | 100 | 100 | 64 | 0 |
| Rv1120c | - | 164 | 0 | 0 | 0 | 0 | 0 | 0 | 0 | 0 | 0 | 0 | 0 | 0 | 0 | 89 | 88 | 89 | 99 | 81 | 75 | 75 | 75 | 0 | 100 | 100 | 100 | 87 | 81 |
| Rv1121 | *zwf1* | 466 | 52 | 53 | 53 | 52 | 52 | 53 | 52 | 52 | 52 | 53 | 53 | 52 | 53 | 85 | 86 | 85 | 100 | 54 | 53 | 53 | 53 | 53 | 100 | 100 | 100 | 88 | 53 |
| Rv1122 | *gnd2* | 340 | 46 | 47 | 46 | 47 | 46 | 47 | 46 | 55 | 71 | 59 | 60 | 59 | 59 | 92 | 92 | 92 | 100 | 58 | 56 | 56 | 56 | 59 | 100 | 100 | 100 | 92 | 58 |
| Rv1123c | *bpoB* | 302 | 0 | 0 | 0 | 0 | 0 | 0 | 0 | 0 | 62 | 0 | 63 | 58 | 56 | 84 | 89 | 85 | 100 | 74 | 81 | 81 | 81 | 79 | 100 | 100 | 100 | 88 | 75 |
| Rv1124 | *ephC* | 316 | 0 | 0 | 0 | 0 | 0 | 0 | 0 | 39 | 44 | 41 | 43 | 43 | 56 | 85 | 85 | 85 | 100 | 61 | 82 | 82 | 82 | 58 | 100 | 100 | 100 | 87 | 61 |
| Rv1125 | - | 414 | 0 | 0 | 0 | 0 | 0 | 0 | 0 | 0 | 48 | 54 | 0 | 0 | 56 | 78 | 78 | 78 | 99 | 64 | 69 | 68 | 68 | 66 | 91 | 100 | 100 | 78 | 67 |
| Rv1126c | - | 201 | 0 | 0 | 0 | 0 | 0 | 0 | 0 | 0 | 0 | 60 | 48 | 57 | 0 | 77 | 82 | 78 | 100 | 0 | 73 | 0 | 0 | 0 | 100 | 100 | 100 | 82 | 0 |
| Rv1127c | *ppdK* | 490 | 0 | 0 | 0 | 0 | 0 | 0 | 0 | 50 | 0 | 62 | 0 | 63 | 0 | 85 | 84 | 85 | 99 | 39 | 78 | 0 | 0 | 39 | 99 | 100 | 100 | 85 | 0 |
| Rv1128c | - | 451 | 0 | 0 | 45 | 43 | 50 | 0 | 0 | 37 | 0 | 39 | 55 | 51 | 66 | 79 | 79 | 79 | 99 | 64 | 62 | 62 | 62 | 57 | 99 | 100 | 100 | 79 | 64 |
| Rv1129c | - | 486 | 64 | 64 | 63 | 62 | 62 | 62 | 62 | 0 | 72 | 77 | 65 | 73 | 78 | 78 | 77 | 78 | 100 | 64 | 65 | 65 | 65 | 78 | 100 | 100 | 100 | 77 | 64 |
| Rv1130 | - | 526 | 77 | 0 | 78 | 78 | 82 | 0 | 80 | 37 | 0 | 81 | 0 | 81 | 84 | 84 | 84 | 84 | 99 | 0 | 0 | 0 | 0 | 86 | 100 | 100 | 100 | 84 | 0 |
| Rv1131 | *gltA1* | 393 | 75 | 51 | 71 | 71 | 70 | 51 | 71 | 49 | 52 | 82 | 80 | 85 | 87 | 86 | 87 | 86 | 100 | 50 | 52 | 52 | 52 | 85 | 100 | 100 | 100 | 87 | 51 |
| Rv1132 | - | 576 | 0 | 0 | 0 | 0 | 0 | 0 | 0 | 0 | 57 | 60 | 60 | 60 | 83 | 89 | 89 | 89 | 99 | 85 | 84 | 84 | 84 | 84 | 100 | 100 | 100 | 92 | 85 |
| Rv1133c | *metE* | 759 | 65 | 0 | 66 | 67 | 68 | 59 | 68 | 0 | 63 | 82 | 81 | 81 | 86 | 91 | 91 | 91 | 100 | 0 | 0 | 0 | 0 | 83 | 100 | 100 | 100 | 91 | 0 |
| Rv1134 | - | 78 | 0 | 0 | 0 | 0 | 0 | 0 | 0 | 0 | 0 | 0 | 0 | 0 | 0 | 0 | 0 | 0 | 100 | 0 | 0 | 0 | 0 | 0 | 100 | 100 | 100 | 0 | 0 |
| Rv1135A | - | 80 | 0 | 0 | 0 | 0 | 0 | 0 | 0 | 78 | 78 | 83 | 80 | 80 | 72 | 90 | 89 | 90 | 100 | 87 | 87 | 87 | 87 | 87 | 100 | 100 | 98 | 89 | 87 |

| | | | | | | | | | | | | | | | | | | | | | | | | | | | | | |
|---|---|---|---|---|---|---|---|---|---|---|---|---|---|---|---|---|---|---|---|---|---|---|---|---|---|---|---|---|---|
| Rv1135c | *PPE16* | 618 | 0 | 0 | 0 | 0 | 0 | 0 | 0 | 0 | 50 | 39 | 0 | 0 | 50 | 64 | 76 | 64 | 100 | 47 | 54 | 54 | 54 | 54 | 99 | 100 | 100 | 75 | 51 |
| Rv1136 | - | 113 | 55 | 0 | 58 | 0 | 52 | 55 | 55 | 55 | 68 | 70 | 71 | 73 | 59 | 85 | 86 | 85 | 100 | 84 | 84 | 84 | 84 | 83 | 100 | 100 | 100 | 84 | 84 |
| Rv1137c | - | 122 | 0 | 0 | 0 | 0 | 0 | 0 | 0 | 0 | 0 | 0 | 0 | 0 | 0 | 65 | 52 | 65 | 100 | 0 | 0 | 0 | 0 | 0 | 100 | 100 | 100 | 52 | 0 |
| Rv1138c | - | 338 | 39 | 39 | 39 | 0 | 36 | 0 | 37 | 41 | 38 | 39 | 40 | 40 | 0 | 90 | 89 | 90 | 100 | 60 | 60 | 61 | 61 | 58 | 100 | 100 | 100 | 89 | 61 |
| Rv1139c | - | 166 | 0 | 0 | 0 | 0 | 0 | 0 | 0 | 0 | 0 | 0 | 0 | 0 | 0 | 86 | 87 | 87 | 100 | 78 | 79 | 79 | 79 | 73 | 100 | 100 | 100 | 93 | 77 |
| Rv1140 | - | 282 | 0 | 0 | 0 | 0 | 0 | 0 | 44 | 59 | 55 | 0 | 0 | 0 | 0 | 79 | 79 | 79 | 100 | 73 | 64 | 64 | 62 | 78 | 100 | 100 | 100 | 79 | 73 |
| Rv1141c | *echA11* | 268 | 48 | 42 | 47 | 0 | 48 | 46 | 48 | 60 | 55 | 57 | 66 | 66 | 53 | 82 | 77 | 82 | 100 | 68 | 71 | 71 | 71 | 73 | 100 | 100 | 100 | 77 | 74 |
| Rv1142c | *echA10* | 268 | 48 | 48 | 47 | 38 | 47 | 45 | 50 | 59 | 60 | 58 | 65 | 63 | 54 | 82 | 79 | 82 | 100 | 71 | 73 | 73 | 73 | 76 | 100 | 100 | 100 | 79 | 76 |
| Rv1143 | *mcr* | 360 | 66 | 0 | 44 | 0 | 48 | 0 | 40 | 65 | 72 | 74 | 74 | 74 | 70 | 89 | 90 | 89 | 100 | 81 | 83 | 83 | 83 | 83 | 100 | 100 | 100 | 90 | 82 |
| Rv1144 | - | 250 | 64 | 0 | 48 | 46 | 50 | 48 | 45 | 66 | 80 | 84 | 82 | 82 | 66 | 89 | 94 | 89 | 100 | 86 | 86 | 86 | 86 | 89 | 100 | 100 | 100 | 94 | 86 |
| Rv1145 | *mmpL13a* | 303 | 48 | 52 | 50 | 49 | 54 | 51 | 50 | 44 | 58 | 53 | 53 | 54 | 70 | 87 | 86 | 88 | 97 | 76 | 72 | 72 | 72 | 74 | 100 | 100 | 100 | 86 | 75 |
| Rv1146 | *mmpL13b* | 470 | 49 | 48 | 48 | 46 | 49 | 43 | 48 | 49 | 62 | 48 | 52 | 52 | 74 | 87 | 88 | 88 | 100 | 80 | 82 | 81 | 81 | 77 | 100 | 100 | 100 | 86 | 81 |
| Rv1147 | - | 216 | 0 | 0 | 0 | 0 | 0 | 0 | 0 | 46 | 49 | 0 | 50 | 50 | 62 | 80 | 82 | 81 | 100 | 78 | 77 | 78 | 78 | 72 | 100 | 100 | 100 | 82 | 77 |
| Rv1148c | - | 482 | 43 | 47 | 42 | 45 | 40 | 0 | 0 | 36 | 0 | 41 | 55 | 52 | 68 | 73 | 76 | 73 | 100 | 59 | 58 | 58 | 58 | 55 | 99 | 100 | 100 | 76 | 58 |
| Rv1149 | - | 135 | 0 | 0 | 0 | 0 | 0 | 0 | 0 | 0 | 63 | 0 | 0 | 0 | 0 | 0 | 0 | 0 | 100 | 61 | 0 | 0 | 0 | 0 | 100 | 100 | 100 | 0 | 61 |
| Rv1151c | - | 237 | 65 | 0 | 62 | 66 | 65 | 64 | 64 | 55 | 52 | 43 | 47 | 46 | 68 | 91 | 92 | 91 | 100 | 82 | 82 | 82 | 82 | 81 | 100 | 100 | 100 | 92 | 84 |
| Rv1152 | - | 121 | 0 | 0 | 0 | 0 | 0 | 0 | 0 | 63 | 67 | 65 | 64 | 64 | 0 | 92 | 98 | 93 | 100 | 81 | 83 | 83 | 83 | 78 | 100 | 100 | 100 | 95 | 82 |
| Rv1153c | *omt* | 282 | 0 | 0 | 0 | 0 | 0 | 0 | 0 | 0 | 0 | 0 | 60 | 62 | 69 | 46 | 89 | 47 | 99 | 72 | 78 | 78 | 78 | 77 | 100 | 100 | 100 | 68 | 73 |
| Rv1154c | - | 213 | 0 | 0 | 0 | 0 | 0 | 0 | 0 | 0 | 0 | 66 | 65 | 65 | 72 | 86 | 86 | 86 | 99 | 45 | 72 | 73 | 73 | 76 | 99 | 100 | 99 | 79 | 0 |
| Rv1155 | - | 147 | 0 | 0 | 0 | 0 | 0 | 0 | 0 | 53 | 59 | 69 | 67 | 69 | 63 | 95 | 96 | 95 | 99 | 86 | 91 | 91 | 91 | 87 | 100 | 100 | 100 | 95 | 84 |
| Rv1156 | - | 195 | 0 | 0 | 0 | 0 | 0 | 0 | 0 | 73 | 74 | 78 | 77 | 77 | 84 | 92 | 93 | 92 | 100 | 87 | 89 | 89 | 89 | 87 | 100 | 100 | 100 | 91 | 85 |
| Rv1157c | - | 371 | 49 | 45 | 59 | 61 | 46 | 0 | 46 | 35 | 50 | 48 | 48 | 48 | 39 | 76 | 77 | 77 | 100 | 65 | 67 | 67 | 67 | 71 | 98 | 100 | 100 | 74 | 65 |
| Rv1158c | - | 227 | 46 | 40 | 42 | 41 | 41 | 38 | 40 | 0 | 40 | 40 | 42 | 43 | 48 | 68 | 72 | 68 | 100 | 44 | 43 | 43 | 43 | 40 | 78 | 100 | 100 | 72 | 44 |
| Rv1159 | *pimE* | 431 | 48 | 50 | 48 | 48 | 47 | 49 | 45 | 0 | 69 | 44 | 69 | 71 | 77 | 85 | 88 | 85 | 100 | 82 | 81 | 81 | 81 | 80 | 100 | 100 | 100 | 87 | 80 |
| Rv1159A | *phhB* | 94 | 0 | 0 | 0 | 52 | 0 | 0 | 0 | 0 | 67 | 64 | 73 | 72 | 76 | 87 | 87 | 87 | 100 | 82 | 86 | 85 | 85 | 87 | 100 | 100 | 100 | 82 | 83 |
| Rv1160 | *mutT2* | 141 | 52 | 47 | 0 | 52 | 51 | 0 | 50 | 55 | 0 | 64 | 65 | 66 | 66 | 83 | 85 | 85 | 100 | 69 | 71 | 72 | 72 | 71 | 100 | 100 | 100 | 80 | 68 |
| Rv1161 | *narG* | 1232 | 0 | 70 | 70 | 69 | 0 | 0 | 37 | 76 | 74 | 0 | 0 | 85 | 0 | 91 | 0 | 92 | 99 | 87 | 87 | 87 | 87 | 87 | 100 | 100 | 100 | 0 | 88 |
| Rv1162 | *narH* | 558 | 0 | 74 | 74 | 73 | 0 | 0 | 50 | 80 | 76 | 0 | 0 | 86 | 0 | 89 | 62 | 89 | 100 | 89 | 89 | 89 | 89 | 86 | 99 | 100 | 100 | 60 | 89 |
| Rv1163 | *narJ* | 201 | 0 | 50 | 51 | 54 | 0 | 0 | 0 | 57 | 59 | 0 | 0 | 75 | 0 | 83 | 84 | 84 | 100 | 71 | 74 | 75 | 75 | 74 | 100 | 100 | 100 | 0 | 75 |
| Rv1164 | *narI* | 246 | 0 | 67 | 68 | 69 | 0 | 0 | 0 | 65 | 69 | 0 | 0 | 83 | 0 | 78 | 78 | 78 | 99 | 84 | 84 | 84 | 84 | 84 | 100 | 100 | 100 | 0 | 84 |
| Rv1165 | *typA* | 628 | 78 | 79 | 78 | 78 | 81 | 79 | 79 | 78 | 86 | 87 | 88 | 88 | 92 | 93 | 93 | 93 | 100 | 92 | 91 | 91 | 91 | 92 | 100 | 100 | 100 | 93 | 92 |
| Rv1166 | *lpqW* | 635 | 42 | 48 | 48 | 45 | 40 | 43 | 41 | 0 | 63 | 63 | 64 | 63 | 74 | 90 | 90 | 89 | 100 | 80 | 82 | 82 | 82 | 83 | 100 | 100 | 100 | 89 | 79 |
| Rv1167c | - | 201 | 0 | 0 | 0 | 0 | 0 | 0 | 0 | 49 | 45 | 0 | 0 | 0 | 44 | 83 | 82 | 81 | 100 | 48 | 43 | 47 | 47 | 49 | 100 | 100 | 100 | 80 | 49 |
| Rv1168c | *PPE17* | 346 | 0 | 0 | 0 | 0 | 0 | 0 | 0 | 0 | 0 | 0 | 0 | 0 | 40 | 46 | 45 | 45 | 100 | 48 | 44 | 44 | 44 | 45 | 100 | 100 | 100 | 46 | 43 |
| Rv1169c | *PE11* | 100 | 0 | 0 | 0 | 0 | 0 | 0 | 0 | 0 | 0 | 0 | 0 | 0 | 0 | 61 | 61 | 61 | 100 | 0 | 0 | 0 | 0 | 0 | 100 | 100 | 100 | 62 | 0 |
| Rv1170 | *mshB* | 303 | 48 | 51 | 53 | 52 | 49 | 48 | 54 | 54 | 62 | 65 | 68 | 66 | 64 | 81 | 87 | 81 | 100 | 73 | 71 | 70 | 70 | 77 | 100 | 100 | 100 | 87 | 73 |
| Rv1171 | - | 146 | 0 | 0 | 0 | 0 | 0 | 0 | 0 | 0 | 0 | 55 | 48 | 50 | 55 | 77 | 76 | 77 | 100 | 66 | 76 | 76 | 76 | 65 | 100 | 100 | 100 | 75 | 66 |
| Rv1172c | *PE12* | 308 | 0 | 0 | 0 | 0 | 0 | 0 | 0 | 0 | 0 | 0 | 0 | 0 | 46 | 51 | 76 | 51 | 99 | 0 | 0 | 0 | 0 | 0 | 100 | 100 | 100 | 73 | 0 |

| | | | | | | | | | | | | | | | | | | | | | | | | | | | | | |
|---|---|---|---|---|---|---|---|---|---|---|---|---|---|---|---|---|---|---|---|---|---|---|---|---|---|---|---|---|---|
| Rv1173 | *fbiC* | 856 | 0 | 0 | 0 | 0 | 0 | 0 | 0 | 77 | 84 | 85 | 86 | 86 | 90 | 92 | 93 | 93 | 100 | 92 | 92 | 92 | 92 | 92 | 100 | 100 | 100 | 93 | 91 |
| Rv1174c | *TB8.4* | 110 | 0 | 0 | 0 | 0 | 0 | 0 | 0 | 0 | 54 | 0 | 0 | 0 | 70 | 79 | 89 | 79 | 100 | 0 | 63 | 63 | 63 | 63 | 100 | 100 | 100 | 88 | 49 |
| Rv1175c | *fadH* | 674 | 47 | 48 | 47 | 44 | 61 | 0 | 63 | 74 | 80 | 80 | 81 | 81 | 81 | 42 | 93 | 42 | 99 | 42 | 42 | 42 | 42 | 89 | 99 | 100 | 99 | 92 | 43 |
| Rv1176c | - | 189 | 0 | 0 | 0 | 0 | 0 | 0 | 0 | 60 | 66 | 67 | 67 | 66 | 65 | 50 | 50 | 50 | 100 | 42 | 56 | 0 | 0 | 51 | 100 | 100 | 100 | 88 | 57 |
| Rv1177 | *fdxC* | 108 | 80 | 79 | 82 | 83 | 84 | 82 | 81 | 83 | 82 | 83 | 86 | 86 | 88 | 96 | 96 | 96 | 100 | 95 | 95 | 95 | 95 | 93 | 100 | 100 | 100 | 96 | 94 |
| Rv1178 | - | 362 | 76 | 72 | 78 | 78 | 78 | 76 | 77 | 74 | 81 | 81 | 80 | 81 | 83 | 91 | 92 | 91 | 99 | 87 | 87 | 87 | 87 | 87 | 99 | 100 | 100 | 91 | 88 |
| Rv1179c | - | 939 | 0 | 0 | 0 | 0 | 43 | 0 | 0 | 0 | 0 | 0 | 0 | 0 | 0 | 0 | 89 | 0 | 100 | 0 | 0 | 0 | 0 | 0 | 100 | 100 | 100 | 86 | 0 |
| Rv1180 | *pks3* | 488 | 56 | 58 | 60 | 59 | 57 | 0 | 57 | 41 | 58 | 56 | 61 | 63 | 77 | 89 | 80 | 88 | 100 | 67 | 82 | 82 | 82 | 81 | 100 | 100 | 100 | 76 | 68 |
| Rv1181 | *pks4* | 1582 | 46 | 48 | 49 | 47 | 48 | 43 | 51 | 45 | 44 | 49 | 43 | 43 | 73 | 82 | 79 | 82 | 99 | 44 | 77 | 78 | 78 | 75 | 100 | 100 | 100 | 75 | 44 |
| Rv1182 | *papA3* | 472 | 0 | 0 | 0 | 0 | 0 | 43 | 0 | 0 | 53 | 44 | 46 | 46 | 73 | 76 | 76 | 76 | 100 | 73 | 71 | 71 | 71 | 74 | 100 | 100 | 100 | 66 | 74 |
| Rv1183 | *mmpL10* | 1002 | 42 | 47 | 57 | 60 | 44 | 64 | 46 | 52 | 55 | 59 | 45 | 45 | 76 | 75 | 69 | 77 | 100 | 75 | 76 | 76 | 76 | 75 | 100 | 100 | 100 | 49 | 76 |
| Rv1184c | - | 359 | 0 | 0 | 0 | 0 | 0 | 0 | 0 | 0 | 0 | 0 | 0 | 0 | 59 | 63 | 46 | 63 | 100 | 62 | 61 | 61 | 61 | 61 | 100 | 100 | 100 | 46 | 61 |
| Rv1185c | *fadD21* | 578 | 51 | 53 | 50 | 50 | 53 | 37 | 51 | 40 | 55 | 56 | 56 | 56 | 74 | 81 | 77 | 82 | 100 | 74 | 73 | 74 | 74 | 73 | 100 | 100 | 100 | 77 | 74 |
| Rv1186c | - | 538 | 0 | 0 | 38 | 0 | 0 | 0 | 0 | 0 | 0 | 58 | 58 | 59 | 0 | 86 | 87 | 87 | 99 | 41 | 76 | 76 | 76 | 80 | 99 | 100 | 99 | 88 | 77 |
| Rv1187 | *rocA* | 543 | 43 | 42 | 41 | 45 | 42 | 42 | 42 | 76 | 83 | 82 | 84 | 84 | 85 | 93 | 91 | 92 | 100 | 46 | 88 | 88 | 88 | 88 | 100 | 100 | 100 | 91 | 87 |
| Rv1188 | - | 329 | 0 | 0 | 0 | 0 | 0 | 0 | 0 | 67 | 70 | 70 | 71 | 72 | 78 | 81 | 85 | 80 | 100 | 0 | 78 | 78 | 78 | 76 | 100 | 100 | 100 | 84 | 80 |
| Rv1189 | *sigI* | 290 | 46 | 46 | 46 | 46 | 50 | 0 | 0 | 51 | 52 | 50 | 52 | 50 | 60 | 61 | 52 | 60 | 100 | 49 | 48 | 48 | 48 | 50 | 100 | 100 | 100 | 47 | 48 |
| Rv1190 | - | 292 | 0 | 0 | 0 | 0 | 0 | 44 | 0 | 0 | 43 | 0 | 48 | 47 | 44 | 0 | 40 | 47 | 100 | 0 | 0 | 0 | 0 | 42 | 100 | 100 | 100 | 40 | 0 |
| Rv1191 | - | 304 | 0 | 0 | 0 | 0 | 0 | 46 | 0 | 72 | 0 | 42 | 82 | 82 | 0 | 87 | 95 | 87 | 100 | 82 | 84 | 84 | 84 | 86 | 100 | 100 | 100 | 94 | 82 |
| Rv1192 | - | 275 | 0 | 0 | 0 | 0 | 0 | 0 | 0 | 50 | 0 | 59 | 0 | 0 | 0 | 0 | 0 | 0 | 100 | 0 | 0 | 0 | 0 | 0 | 100 | 100 | 100 | 77 | 0 |
| Rv1193 | *fadD36* | 473 | 54 | 54 | 53 | 53 | 53 | 53 | 51 | 54 | 79 | 77 | 80 | 80 | 81 | 90 | 90 | 91 | 100 | 83 | 87 | 87 | 87 | 87 | 100 | 100 | 100 | 90 | 85 |
| Rv1194c | - | 421 | 0 | 0 | 0 | 0 | 0 | 0 | 0 | 40 | 54 | 44 | 56 | 54 | 57 | 0 | 86 | 0 | 100 | 38 | 0 | 0 | 0 | 0 | 100 | 100 | 100 | 85 | 0 |
| Rv1195 | *PE13* | 99 | 0 | 0 | 0 | 0 | 0 | 0 | 0 | 0 | 0 | 0 | 0 | 0 | 0 | 82 | 81 | 82 | 100 | 0 | 0 | 0 | 0 | 0 | 100 | 100 | 100 | 79 | 0 |
| Rv1196 | *PPE18* | 391 | 0 | 0 | 0 | 0 | 0 | 0 | 0 | 0 | 37 | 0 | 39 | 39 | 51 | 57 | 56 | 56 | 99 | 52 | 45 | 45 | 45 | 51 | 100 | 89 | 100 | 70 | 54 |
| Rv1197 | *esxK* | 98 | 0 | 0 | 0 | 0 | 0 | 0 | 0 | 0 | 0 | 0 | 0 | 0 | 0 | 93 | 96 | 93 | 98 | 0 | 0 | 0 | 0 | 0 | 100 | 100 | 100 | 96 | 0 |
| Rv1198 | *esxL* | 94 | 0 | 0 | 0 | 0 | 0 | 0 | 0 | 0 | 0 | 0 | 0 | 0 | 0 | 91 | 91 | 91 | 98 | 0 | 0 | 0 | 0 | 0 | 97 | 100 | 98 | 93 | 0 |
| Rv1199c | - | 415 | 45 | 66 | 74 | 0 | 73 | 0 | 69 | 59 | 0 | 40 | 87 | 58 | 0 | 60 | 0 | 79 | 100 | 89 | 42 | 79 | 79 | 59 | 99 | 100 | 99 | 43 | 89 |
| Rv1200 | - | 425 | 58 | 52 | 56 | 56 | 0 | 59 | 57 | 37 | 58 | 68 | 68 | 67 | 66 | 77 | 80 | 77 | 100 | 57 | 59 | 59 | 59 | 57 | 100 | 100 | 100 | 80 | 58 |
| Rv1201c | - | 317 | 76 | 76 | 75 | 73 | 73 | 77 | 0 | 75 | 79 | 80 | 81 | 82 | 84 | 93 | 96 | 93 | 100 | 90 | 87 | 87 | 87 | 83 | 100 | 100 | 100 | 95 | 90 |
| Rv1202 | *dapE* | 354 | 68 | 67 | 69 | 71 | 63 | 63 | 0 | 67 | 77 | 82 | 81 | 82 | 84 | 90 | 94 | 91 | 100 | 86 | 89 | 89 | 89 | 87 | 100 | 100 | 100 | 94 | 85 |
| Rv1203c | - | 194 | 0 | 0 | 0 | 0 | 0 | 0 | 0 | 0 | 48 | 0 | 46 | 49 | 45 | 73 | 75 | 75 | 100 | 64 | 66 | 66 | 66 | 67 | 100 | 100 | 100 | 82 | 67 |
| Rv1204c | - | 562 | 0 | 0 | 0 | 0 | 0 | 0 | 0 | 0 | 51 | 0 | 50 | 50 | 49 | 48 | 67 | 62 | 100 | 62 | 59 | 59 | 59 | 57 | 100 | 100 | 100 | 67 | 58 |
| Rv1205 | - | 187 | 59 | 57 | 0 | 50 | 57 | 43 | 58 | 0 | 75 | 76 | 77 | 77 | 79 | 81 | 90 | 81 | 100 | 83 | 85 | 85 | 85 | 84 | 100 | 100 | 100 | 90 | 81 |
| Rv1206 | *fadD6* | 597 | 37 | 0 | 37 | 37 | 39 | 39 | 42 | 41 | 72 | 72 | 72 | 71 | 79 | 89 | 89 | 89 | 100 | 80 | 80 | 79 | 79 | 83 | 100 | 100 | 100 | 90 | 80 |
| Rv1207 | *folP2* | 318 | 67 | 68 | 69 | 70 | 44 | 60 | 45 | 71 | 72 | 79 | 79 | 80 | 86 | 86 | 89 | 86 | 100 | 88 | 85 | 89 | 89 | 87 | 100 | 100 | 100 | 89 | 87 |
| Rv1208 | - | 324 | 64 | 60 | 61 | 64 | 0 | 0 | 0 | 64 | 76 | 78 | 77 | 77 | 84 | 89 | 86 | 88 | 100 | 83 | 82 | 82 | 82 | 81 | 100 | 100 | 100 | 86 | 84 |
| Rv1209 | - | 122 | 0 | 0 | 0 | 54 | 0 | 0 | 0 | 0 | 64 | 72 | 74 | 75 | 75 | 85 | 84 | 84 | 100 | 72 | 78 | 81 | 81 | 73 | 100 | 100 | 100 | 80 | 73 |
| Rv1210 | *tagA* | 204 | 62 | 66 | 63 | 66 | 57 | 60 | 52 | 61 | 77 | 75 | 80 | 78 | 80 | 83 | 90 | 84 | 100 | 78 | 80 | 80 | 80 | 79 | 100 | 100 | 100 | 90 | 79 |

| | | | | | | | | | | | | | | | | | | | | | | | | | | | | |
|---|---|---|---|---|---|---|---|---|---|---|---|---|---|---|---|---|---|---|---|---|---|---|---|---|---|---|---|---|
| Rv1211 | - | 75 | 68 | 77 | 83 | 83 | 0 | 80 | 0 | 88 | 88 | 92 | 96 | 96 | 96 | 97 | 95 | 95 | 100 | 100 | 96 | 96 | 96 | 100 | 100 | 100 | 100 | 93 | 92 |
| Rv1212c | - | 387 | 73 | 74 | 73 | 73 | 72 | 71 | 45 | 66 | 74 | 79 | 80 | 80 | 82 | 88 | 90 | 88 | 100 | 80 | 84 | 84 | 84 | 85 | 100 | 100 | 100 | 91 | 82 |
| Rv1213 | *glgC* | 404 | 78 | 82 | 83 | 83 | 85 | 84 | 44 | 70 | 90 | 92 | 91 | 91 | 94 | 98 | 98 | 98 | 100 | 96 | 97 | 97 | 97 | 97 | 100 | 100 | 100 | 97 | 96 |
| Rv1214c | *PE14* | 110 | 0 | 0 | 0 | 0 | 0 | 0 | 0 | 0 | 0 | 0 | 0 | 0 | 0 | 68 | 80 | 72 | 82 | 0 | 0 | 0 | 0 | 0 | 100 | 100 | 100 | 79 | 0 |
| Rv1215c | - | 561 | 0 | 0 | 0 | 0 | 0 | 0 | 0 | 41 | 38 | 46 | 47 | 41 | 36 | 85 | 86 | 86 | 100 | 0 | 37 | 37 | 37 | 69 | 100 | 100 | 100 | 66 | 36 |
| Rv1216c | - | 224 | 0 | 0 | 0 | 0 | 0 | 0 | 0 | 0 | 0 | 0 | 0 | 0 | 72 | 75 | 77 | 75 | 100 | 0 | 68 | 75 | 75 | 70 | 100 | 100 | 100 | 76 | 72 |
| Rv1217c | - | 548 | 0 | 0 | 52 | 42 | 0 | 0 | 0 | 55 | 70 | 56 | 75 | 75 | 47 | 83 | 89 | 83 | 99 | 50 | 48 | 48 | 48 | 50 | 100 | 100 | 100 | 87 | 48 |
| Rv1218c | - | 311 | 53 | 55 | 75 | 63 | 51 | 52 | 49 | 78 | 82 | 74 | 83 | 84 | 73 | 92 | 90 | 93 | 100 | 71 | 71 | 71 | 71 | 71 | 99 | 100 | 100 | 49 | 73 |
| Rv1219c | - | 212 | 0 | 0 | 0 | 0 | 0 | 0 | 0 | 0 | 68 | 0 | 69 | 69 | 74 | 89 | 90 | 90 | 100 | 82 | 86 | 85 | 85 | 83 | 100 | 100 | 100 | 50 | 87 |
| Rv1220c | - | 215 | 50 | 53 | 49 | 49 | 46 | 51 | 43 | 64 | 74 | 71 | 72 | 72 | 84 | 92 | 92 | 92 | 100 | 88 | 85 | 87 | 87 | 89 | 100 | 100 | 100 | 92 | 88 |
| Rv1221 | *sigE* | 257 | 81 | 77 | 80 | 82 | 77 | 70 | 72 | 70 | 93 | 92 | 83 | 83 | 81 | 88 | 89 | 88 | 100 | 93 | 84 | 83 | 83 | 91 | 100 | 100 | 100 | 88 | 90 |
| Rv1222 | - | 154 | 0 | 52 | 0 | 0 | 0 | 50 | 0 | 0 | 64 | 74 | 80 | 79 | 72 | 91 | 92 | 91 | 100 | 76 | 87 | 85 | 85 | 89 | 99 | 100 | 100 | 92 | 78 |
| Rv1223 | *htrA* | 528 | 57 | 48 | 49 | 59 | 52 | 53 | 52 | 52 | 66 | 65 | 64 | 63 | 80 | 89 | 91 | 89 | 99 | 84 | 84 | 83 | 83 | 80 | 100 | 100 | 96 | 93 | 84 |
| Rv1224 | *tatB* | 131 | 57 | 60 | 58 | 62 | 54 | 0 | 58 | 0 | 68 | 68 | 68 | 81 | 69 | 75 | 82 | 74 | 99 | 74 | 74 | 74 | 74 | 76 | 100 | 100 | 100 | 82 | 76 |
| Rv1225c | - | 276 | 43 | 42 | 43 | 44 | 42 | 44 | 43 | 44 | 45 | 67 | 69 | 69 | 73 | 0 | 0 | 0 | 100 | 77 | 79 | 79 | 79 | 79 | 100 | 100 | 100 | 0 | 79 |
| Rv1226c | - | 487 | 0 | 0 | 0 | 0 | 0 | 0 | 0 | 54 | 55 | 65 | 63 | 64 | 44 | 0 | 0 | 0 | 99 | 74 | 75 | 75 | 75 | 79 | 100 | 100 | 100 | 0 | 75 |
| Rv1227c | - | 177 | 0 | 0 | 0 | 0 | 0 | 0 | 0 | 61 | 64 | 69 | 71 | 71 | 49 | 0 | 0 | 0 | 100 | 81 | 76 | 76 | 76 | 71 | 100 | 100 | 100 | 0 | 80 |
| Rv1228 | *lpqX* | 185 | 0 | 0 | 0 | 0 | 0 | 0 | 0 | 0 | 0 | 0 | 0 | 0 | 0 | 0 | 0 | 0 | 100 | 0 | 0 | 0 | 0 | 0 | 100 | 100 | 100 | 0 | 0 |
| Rv1229c | *mrp* | 390 | 79 | 77 | 79 | 78 | 78 | 79 | 78 | 71 | 86 | 84 | 87 | 87 | 86 | 93 | 91 | 93 | 100 | 88 | 91 | 91 | 91 | 91 | 100 | 100 | 100 | 91 | 88 |
| Rv1230c | - | 411 | 46 | 46 | 41 | 54 | 48 | 42 | 44 | 45 | 50 | 50 | 51 | 52 | 63 | 78 | 78 | 78 | 99 | 62 | 67 | 67 | 67 | 67 | 99 | 100 | 99 | 78 | 63 |
| Rv1231c | - | 180 | 0 | 74 | 69 | 72 | 70 | 69 | 68 | 67 | 72 | 81 | 82 | 82 | 80 | 83 | 84 | 83 | 100 | 78 | 75 | 75 | 75 | 76 | 100 | 100 | 100 | 0 | 76 |
| Rv1232c | - | 435 | 0 | 69 | 68 | 68 | 66 | 69 | 68 | 67 | 80 | 81 | 78 | 78 | 81 | 91 | 93 | 91 | 99 | 85 | 85 | 85 | 85 | 84 | 99 | 100 | 99 | 0 | 84 |
| Rv1233c | - | 198 | 0 | 0 | 0 | 0 | 0 | 0 | 0 | 43 | 48 | 48 | 44 | 46 | 50 | 61 | 68 | 60 | 99 | 50 | 52 | 50 | 50 | 50 | 100 | 100 | 100 | 43 | 58 |
| Rv1234 | - | 175 | 0 | 74 | 75 | 79 | 0 | 70 | 0 | 71 | 84 | 83 | 84 | 83 | 82 | 95 | 95 | 95 | 100 | 91 | 92 | 92 | 92 | 92 | 100 | 100 | 100 | 0 | 89 |
| Rv1235 | *lpqY* | 468 | 39 | 0 | 41 | 42 | 0 | 0 | 42 | 42 | 43 | 64 | 65 | 65 | 79 | 85 | 87 | 86 | 100 | 45 | 84 | 84 | 84 | 83 | 100 | 100 | 100 | 92 | 46 |
| Rv1236 | *sugA* | 307 | 57 | 0 | 57 | 57 | 0 | 50 | 56 | 56 | 60 | 86 | 88 | 88 | 86 | 91 | 89 | 91 | 100 | 70 | 88 | 88 | 88 | 86 | 100 | 100 | 100 | 90 | 69 |
| Rv1237 | *sugB* | 274 | 67 | 50 | 68 | 67 | 0 | 48 | 64 | 58 | 66 | 89 | 88 | 88 | 90 | 95 | 95 | 95 | 100 | 76 | 94 | 94 | 94 | 93 | 100 | 100 | 100 | 93 | 76 |
| Rv1238 | *sugC* | 393 | 60 | 67 | 71 | 61 | 54 | 60 | 63 | 60 | 61 | 82 | 81 | 81 | 83 | 92 | 92 | 92 | 100 | 69 | 85 | 85 | 85 | 83 | 100 | 100 | 100 | 90 | 84 |
| Rv1239c | *corA* | 366 | 61 | 65 | 53 | 53 | 62 | 60 | 0 | 62 | 72 | 74 | 74 | 75 | 77 | 87 | 91 | 87 | 99 | 77 | 78 | 78 | 78 | 80 | 100 | 100 | 100 | 92 | 78 |
| Rv1240 | *mdh* | 329 | 70 | 69 | 71 | 68 | 69 | 71 | 67 | 92 | 80 | 78 | 76 | 77 | 0 | 95 | 96 | 95 | 100 | 75 | 0 | 0 | 0 | 0 | 100 | 100 | 100 | 95 | 76 |
| Rv1241 | - | 86 | 0 | 0 | 0 | 0 | 0 | 0 | 0 | 0 | 0 | 0 | 0 | 0 | 0 | 0 | 0 | 0 | 100 | 0 | 0 | 0 | 0 | 0 | 100 | 100 | 100 | 0 | 0 |
| Rv1242 | - | 143 | 0 | 0 | 0 | 0 | 0 | 0 | 0 | 0 | 0 | 0 | 0 | 0 | 0 | 0 | 0 | 0 | 100 | 52 | 0 | 0 | 0 | 0 | 100 | 100 | 100 | 0 | 0 |
| Rv1243c | *PE_PGRS23* | 562 | 44 | 40 | 35 | 35 | 36 | 36 | 38 | 35 | 38 | 39 | 42 | 42 | 45 | 43 | 56 | 56 | 100 | 48 | 50 | 50 | 50 | 48 | 100 | 100 | 100 | 59 | 49 |
| Rv1244 | *lpqZ* | 286 | 0 | 41 | 44 | 0 | 0 | 0 | 0 | 0 | 46 | 45 | 48 | 50 | 59 | 80 | 79 | 80 | 100 | 69 | 69 | 69 | 69 | 69 | 100 | 100 | 100 | 79 | 68 |
| Rv1245c | - | 276 | 48 | 46 | 51 | 51 | 52 | 51 | 46 | 75 | 79 | 81 | 81 | 82 | 85 | 90 | 89 | 90 | 100 | 88 | 89 | 89 | 89 | 90 | 100 | 100 | 100 | 88 | 89 |
| Rv1246c | - | 97 | 0 | 0 | 0 | 0 | 0 | 0 | 0 | 57 | 0 | 0 | 0 | 72 | 0 | 0 | 0 | 0 | 100 | 0 | 0 | 0 | 0 | 0 | 100 | 100 | 100 | 0 | 0 |
| Rv1247c | - | 89 | 0 | 0 | 0 | 0 | 0 | 0 | 0 | 59 | 0 | 0 | 0 | 60 | 0 | 0 | 0 | 0 | 100 | 0 | 0 | 0 | 0 | 0 | 100 | 100 | 100 | 0 | 0 |
| Rv1248c | *kgd* | 1231 | 73 | 73 | 74 | 74 | 72 | 71 | 74 | 70 | 82 | 84 | 84 | 84 | 88 | 93 | 93 | 93 | 99 | 86 | 88 | 88 | 88 | 91 | 100 | 100 | 99 | 93 | 88 |

| | | | | | | | | | | | | | | | | | | | | | | | | | | | | | |
|---|---|---|---|---|---|---|---|---|---|---|---|---|---|---|---|---|---|---|---|---|---|---|---|---|---|---|---|---|---|
| Rv1249c | - | 262 | 56 | 51 | 54 | 56 | 50 | 47 | 0 | 0 | 67 | 65 | 63 | 62 | 71 | 79 | 79 | 79 | 99 | 75 | 80 | 80 | 80 | 79 | 100 | 100 | 100 | 83 | 75 |
| Rv1250 | - | 579 | 58 | 57 | 58 | 59 | 62 | 63 | 61 | 69 | 67 | 73 | 73 | 72 | 67 | 78 | 78 | 78 | 99 | 76 | 71 | 72 | 72 | 76 | 100 | 100 | 100 | 72 | 69 |
| Rv1251c | - | 1139 | 0 | 0 | 0 | 0 | 0 | 0 | 0 | 0 | 51 | 66 | 66 | 67 | 71 | 85 | 85 | 85 | 100 | 74 | 78 | 78 | 78 | 75 | 100 | 100 | 100 | 85 | 75 |
| Rv1252c | *lprE* | 202 | 0 | 0 | 0 | 0 | 0 | 0 | 0 | 0 | 0 | 0 | 0 | 0 | 60 | 79 | 82 | 78 | 100 | 74 | 73 | 73 | 73 | 71 | 100 | 100 | 100 | 82 | 72 |
| Rv1253 | *deaD* | 563 | 70 | 72 | 74 | 73 | 71 | 73 | 72 | 73 | 78 | 81 | 82 | 83 | 83 | 89 | 89 | 89 | 100 | 84 | 83 | 83 | 83 | 85 | 100 | 100 | 100 | 89 | 84 |
| Rv1254 | - | 383 | 47 | 46 | 49 | 50 | 45 | 0 | 47 | 53 | 50 | 52 | 49 | 49 | 76 | 88 | 87 | 87 | 100 | 81 | 86 | 86 | 86 | 83 | 100 | 100 | 100 | 87 | 84 |
| Rv1255c | - | 202 | 0 | 0 | 0 | 0 | 0 | 0 | 0 | 61 | 47 | 65 | 67 | 65 | 62 | 81 | 87 | 81 | 58 | 75 | 76 | 76 | 76 | 75 | 100 | 100 | 100 | 87 | 76 |
| Rv1256c | *cyp130* | 405 | 0 | 0 | 46 | 48 | 39 | 0 | 0 | 78 | 51 | 79 | 78 | 79 | 80 | 89 | 90 | 89 | 50 | 85 | 85 | 85 | 85 | 86 | 100 | 100 | 99 | 88 | 84 |
| Rv1257c | - | 455 | 38 | 0 | 42 | 42 | 0 | 59 | 0 | 74 | 80 | 80 | 81 | 81 | 81 | 94 | 93 | 94 | 51 | 88 | 87 | 87 | 87 | 88 | 100 | 100 | 100 | 93 | 87 |
| Rv1258c | - | 419 | 0 | 40 | 42 | 41 | 0 | 0 | 0 | 41 | 42 | 71 | 71 | 70 | 75 | 80 | 81 | 81 | 100 | 81 | 78 | 78 | 78 | 74 | 100 | 100 | 100 | 84 | 81 |
| Rv1259 | - | 299 | 0 | 0 | 0 | 0 | 0 | 0 | 0 | 67 | 62 | 0 | 67 | 60 | 0 | 84 | 88 | 84 | 100 | 75 | 78 | 78 | 78 | 76 | 100 | 100 | 100 | 88 | 75 |
| Rv1260 | - | 372 | 0 | 0 | 0 | 0 | 0 | 0 | 38 | 0 | 54 | 0 | 49 | 54 | 61 | 89 | 90 | 89 | 100 | 0 | 56 | 56 | 56 | 57 | 100 | 100 | 100 | 90 | 55 |
| Rv1261c | - | 149 | 0 | 0 | 0 | 0 | 0 | 0 | 0 | 47 | 68 | 58 | 66 | 55 | 67 | 78 | 82 | 78 | 100 | 78 | 84 | 83 | 83 | 79 | 100 | 100 | 100 | 82 | 79 |
| Rv1262c | - | 144 | 0 | 43 | 53 | 0 | 0 | 0 | 0 | 57 | 61 | 58 | 60 | 59 | 77 | 90 | 90 | 90 | 99 | 85 | 83 | 83 | 83 | 86 | 100 | 100 | 100 | 88 | 86 |
| Rv1263 | *amiB2* | 462 | 43 | 53 | 46 | 43 | 55 | 44 | 54 | 62 | 46 | 58 | 57 | 58 | 57 | 89 | 91 | 89 | 100 | 56 | 57 | 57 | 57 | 57 | 100 | 100 | 100 | 90 | 57 |
| Rv1264 | - | 397 | 0 | 0 | 0 | 0 | 0 | 0 | 0 | 48 | 42 | 48 | 52 | 50 | 44 | 84 | 88 | 85 | 99 | 76 | 79 | 79 | 79 | 76 | 100 | 100 | 100 | 88 | 77 |
| Rv1265 | - | 226 | 0 | 0 | 0 | 0 | 0 | 0 | 0 | 0 | 78 | 82 | 75 | 82 | 75 | 83 | 80 | 88 | 100 | 0 | 84 | 84 | 84 | 76 | 100 | 100 | 100 | 84 | 82 |
| Rv1266c | *pknH* | 626 | 55 | 51 | 44 | 52 | 44 | 46 | 42 | 50 | 55 | 59 | 68 | 69 | 60 | 72 | 83 | 82 | 93 | 67 | 57 | 57 | 57 | 64 | 100 | 100 | 100 | 81 | 68 |
| Rv1267c | *embR* | 388 | 0 | 0 | 0 | 0 | 0 | 0 | 0 | 43 | 62 | 49 | 69 | 51 | 0 | 85 | 85 | 85 | 100 | 44 | 46 | 47 | 47 | 51 | 100 | 100 | 100 | 94 | 52 |
| Rv1268c | - | 232 | 0 | 0 | 0 | 0 | 0 | 0 | 0 | 0 | 0 | 0 | 0 | 0 | 0 | 55 | 86 | 55 | 100 | 49 | 0 | 0 | 0 | 0 | 100 | 100 | 100 | 86 | 0 |
| Rv1269c | - | 124 | 0 | 0 | 0 | 0 | 0 | 0 | 0 | 0 | 56 | 0 | 0 | 0 | 0 | 0 | 90 | 0 | 100 | 0 | 0 | 0 | 0 | 0 | 100 | 100 | 100 | 89 | 0 |
| Rv1270c | *lprA* | 244 | 0 | 0 | 0 | 0 | 0 | 0 | 0 | 0 | 46 | 0 | 0 | 0 | 50 | 56 | 56 | 56 | 99 | 55 | 56 | 56 | 56 | 55 | 100 | 100 | 100 | 88 | 56 |
| Rv1271c | - | 113 | 0 | 0 | 0 | 0 | 0 | 0 | 0 | 0 | 0 | 0 | 0 | 0 | 0 | 62 | 86 | 62 | 100 | 57 | 0 | 0 | 0 | 0 | 100 | 100 | 100 | 85 | 57 |
| Rv1272c | - | 631 | 70 | 51 | 52 | 71 | 48 | 53 | 51 | 71 | 73 | 74 | 72 | 73 | 83 | 86 | 88 | 85 | 100 | 84 | 85 | 85 | 85 | 85 | 100 | 100 | 100 | 87 | 83 |
| Rv1273c | - | 582 | 66 | 48 | 48 | 70 | 46 | 48 | 46 | 73 | 71 | 73 | 71 | 72 | 78 | 89 | 88 | 88 | 100 | 83 | 84 | 84 | 84 | 83 | 99 | 100 | 100 | 90 | 82 |
| Rv1274 | *lprB* | 185 | 0 | 0 | 0 | 0 | 0 | 0 | 0 | 0 | 51 | 53 | 56 | 56 | 65 | 88 | 93 | 88 | 99 | 73 | 81 | 81 | 81 | 78 | 100 | 100 | 100 | 93 | 77 |
| Rv1275 | *lprC* | 180 | 0 | 0 | 0 | 0 | 0 | 0 | 0 | 0 | 56 | 53 | 58 | 55 | 71 | 93 | 93 | 93 | 100 | 85 | 86 | 86 | 86 | 85 | 100 | 100 | 100 | 92 | 87 |
| Rv1276c | - | 158 | 0 | 0 | 48 | 48 | 0 | 0 | 0 | 50 | 64 | 68 | 68 | 68 | 67 | 78 | 82 | 77 | 100 | 69 | 69 | 70 | 70 | 73 | 100 | 100 | 100 | 82 | 72 |
| Rv1277 | - | 417 | 57 | 56 | 54 | 56 | 53 | 49 | 53 | 0 | 43 | 0 | 0 | 0 | 78 | 88 | 88 | 88 | 100 | 80 | 80 | 80 | 80 | 80 | 100 | 100 | 100 | 90 | 77 |
| Rv1278 | - | 875 | 49 | 44 | 45 | 46 | 42 | 41 | 42 | 38 | 36 | 35 | 38 | 0 | 65 | 82 | 82 | 82 | 100 | 67 | 68 | 68 | 68 | 67 | 100 | 100 | 100 | 82 | 68 |
| Rv1279 | - | 528 | 54 | 54 | 0 | 0 | 54 | 53 | 53 | 52 | 77 | 46 | 78 | 78 | 75 | 44 | 88 | 50 | 100 | 52 | 51 | 51 | 51 | 50 | 100 | 100 | 100 | 88 | 49 |
| Rv1280c | *oppA* | 591 | 52 | 41 | 41 | 39 | 39 | 55 | 41 | 39 | 70 | 38 | 70 | 72 | 38 | 90 | 88 | 90 | 100 | 77 | 82 | 82 | 82 | 80 | 100 | 100 | 100 | 88 | 77 |
| Rv1281c | *oppD* | 612 | 71 | 57 | 58 | 57 | 58 | 70 | 57 | 64 | 75 | 59 | 77 | 77 | 56 | 90 | 91 | 90 | 100 | 85 | 85 | 85 | 85 | 86 | 100 | 100 | 100 | 91 | 84 |
| Rv1282c | *oppC* | 291 | 65 | 52 | 54 | 52 | 0 | 64 | 50 | 53 | 75 | 54 | 82 | 82 | 51 | 92 | 94 | 91 | 100 | 83 | 86 | 86 | 86 | 86 | 100 | 100 | 100 | 94 | 85 |
| Rv1283c | *oppB* | 325 | 59 | 53 | 52 | 52 | 0 | 57 | 48 | 52 | 78 | 49 | 79 | 80 | 48 | 93 | 93 | 93 | 100 | 87 | 91 | 91 | 91 | 89 | 100 | 100 | 100 | 92 | 87 |
| Rv1284 | - | 163 | 0 | 0 | 0 | 0 | 0 | 0 | 0 | 49 | 0 | 88 | 89 | 89 | 80 | 90 | 90 | 90 | 100 | 87 | 90 | 90 | 90 | 89 | 100 | 100 | 100 | 93 | 89 |
| Rv1285 | *cysD* | 332 | 77 | 0 | 71 | 70 | 71 | 71 | 73 | 79 | 81 | 80 | 80 | 80 | 81 | 93 | 96 | 93 | 100 | 95 | 88 | 88 | 88 | 93 | 99 | 100 | 100 | 96 | 94 |
| Rv1286 | *cysN* | 614 | 70 | 50 | 67 | 69 | 65 | 63 | 63 | 77 | 75 | 74 | 74 | 75 | 73 | 93 | 96 | 93 | 100 | 91 | 91 | 91 | 91 | 92 | 100 | 100 | 100 | 95 | 92 |

| | | | | | | | | | | | | | | | | | | | | | | | | | | | | | |
|---|---|---|---|---|---|---|---|---|---|---|---|---|---|---|---|---|---|---|---|---|---|---|---|---|---|---|---|---|---|
| Rv1287 | - | 161 | 0 | 0 | 0 | 0 | 0 | 0 | 0 | 60 | 56 | 66 | 65 | 65 | 0 | 93 | 95 | 92 | 100 | 89 | 88 | 88 | 88 | 89 | 100 | 100 | 100 | 94 | 90 |
| Rv1288 | - | 456 | 0 | 0 | 0 | 0 | 0 | 46 | 0 | 0 | 55 | 40 | 0 | 0 | 88 | 0 | 0 | 0 | 99 | 0 | 0 | 0 | 0 | 90 | 100 | 100 | 100 | 0 | 0 |
| Rv1289 | - | 210 | 0 | 0 | 0 | 0 | 0 | 0 | 0 | 0 | 0 | 0 | 0 | 0 | 0 | 0 | 91 | 0 | 100 | 0 | 0 | 0 | 0 | 0 | 100 | 100 | 100 | 0 | 0 |
| Rv1290A | - | 104 | 0 | 0 | 0 | 0 | 0 | 0 | 0 | 0 | 0 | 0 | 0 | 0 | 0 | 0 | 0 | 0 | 100 | 0 | 0 | 0 | 0 | 0 | 100 | 100 | 100 | 0 | 0 |
| Rv1290c | - | 521 | 0 | 0 | 46 | 44 | 0 | 0 | 0 | 0 | 0 | 44 | 0 | 0 | 0 | 0 | 88 | 0 | 100 | 50 | 52 | 52 | 52 | 0 | 99 | 100 | 100 | 87 | 47 |
| Rv1291c | - | 111 | 0 | 0 | 0 | 0 | 0 | 0 | 0 | 0 | 0 | 0 | 0 | 0 | 0 | 57 | 91 | 55 | 100 | 0 | 0 | 0 | 0 | 0 | 100 | 100 | 100 | 91 | 0 |
| Rv1292 | *argS* | 550 | 78 | 80 | 79 | 79 | 78 | 77 | 77 | 71 | 82 | 86 | 84 | 85 | 86 | 94 | 94 | 91 | 100 | 90 | 89 | 90 | 90 | 89 | 100 | 100 | 99 | 93 | 91 |
| Rv1293 | *lysA* | 447 | 69 | 73 | 72 | 73 | 70 | 71 | 70 | 67 | 84 | 87 | 87 | 87 | 86 | 90 | 94 | 90 | 100 | 88 | 88 | 88 | 88 | 89 | 100 | 100 | 100 | 93 | 87 |
| Rv1294 | *thrA* | 441 | 74 | 72 | 74 | 76 | 75 | 74 | 75 | 75 | 79 | 84 | 84 | 84 | 88 | 94 | 94 | 94 | 100 | 92 | 93 | 93 | 93 | 93 | 100 | 100 | 100 | 95 | 93 |
| Rv1295 | *thrC* | 360 | 46 | 41 | 41 | 42 | 79 | 0 | 0 | 72 | 82 | 84 | 83 | 83 | 89 | 95 | 96 | 95 | 100 | 93 | 92 | 93 | 93 | 91 | 100 | 100 | 100 | 96 | 93 |
| Rv1296 | *thrB* | 316 | 65 | 63 | 64 | 64 | 61 | 67 | 63 | 57 | 68 | 72 | 73 | 73 | 75 | 85 | 87 | 85 | 100 | 79 | 83 | 83 | 83 | 83 | 100 | 100 | 100 | 86 | 80 |
| Rv1297 | *rho* | 602 | 67 | 73 | 68 | 73 | 70 | 65 | 71 | 68 | 71 | 67 | 70 | 71 | 76 | 86 | 86 | 86 | 100 | 72 | 78 | 78 | 78 | 75 | 100 | 100 | 100 | 85 | 75 |
| Rv1298 | *rpmE* | 80 | 56 | 55 | 56 | 55 | 55 | 54 | 54 | 75 | 89 | 86 | 86 | 86 | 85 | 91 | 94 | 91 | 100 | 86 | 85 | 85 | 85 | 85 | 100 | 100 | 100 | 94 | 86 |
| Rv1299 | *prfA* | 357 | 76 | 79 | 81 | 80 | 80 | 80 | 80 | 75 | 87 | 89 | 88 | 88 | 92 | 94 | 94 | 93 | 100 | 89 | 91 | 91 | 91 | 90 | 100 | 100 | 100 | 94 | 90 |
| Rv1300 | *hemK* | 325 | 55 | 56 | 61 | 57 | 47 | 45 | 43 | 52 | 64 | 60 | 60 | 61 | 66 | 81 | 81 | 79 | 99 | 70 | 67 | 67 | 67 | 69 | 99 | 100 | 99 | 81 | 75 |
| Rv1301 | - | 217 | 76 | 76 | 76 | 76 | 70 | 74 | 70 | 67 | 78 | 78 | 80 | 80 | 80 | 90 | 90 | 91 | 99 | 87 | 86 | 86 | 86 | 85 | 100 | 100 | 100 | 90 | 88 |
| Rv1302 | *rfe* | 404 | 69 | 74 | 72 | 69 | 71 | 71 | 72 | 64 | 76 | 77 | 80 | 80 | 90 | 97 | 97 | 97 | 99 | 91 | 94 | 94 | 94 | 92 | 100 | 100 | 100 | 97 | 92 |
| Rv1303 | - | 161 | 0 | 0 | 0 | 0 | 0 | 0 | 0 | 0 | 0 | 0 | 0 | 0 | 76 | 86 | 83 | 86 | 100 | 81 | 83 | 83 | 83 | 78 | 100 | 100 | 100 | 83 | 81 |
| Rv1304 | *atpB* | 250 | 0 | 43 | 0 | 0 | 45 | 41 | 44 | 46 | 0 | 42 | 41 | 41 | 85 | 92 | 97 | 93 | 100 | 83 | 89 | 89 | 89 | 84 | 100 | 100 | 100 | 96 | 84 |
| Rv1305 | *atpE* | 81 | 0 | 0 | 0 | 0 | 0 | 0 | 0 | 0 | 0 | 0 | 0 | 0 | 98 | 96 | 100 | 96 | 100 | 100 | 100 | 100 | 100 | 93 | 100 | 100 | 100 | 100 | 93 |
| Rv1306 | *atpF* | 171 | 0 | 56 | 0 | 0 | 57 | 52 | 55 | 51 | 52 | 51 | 54 | 54 | 72 | 88 | 92 | 88 | 100 | 78 | 74 | 74 | 74 | 75 | 100 | 100 | 100 | 91 | 76 |
| Rv1307 | *atpH* | 446 | 53 | 51 | 51 | 50 | 51 | 50 | 54 | 53 | 52 | 49 | 56 | 56 | 69 | 87 | 87 | 87 | 100 | 74 | 75 | 75 | 75 | 75 | 100 | 100 | 100 | 86 | 74 |
| Rv1308 | *atpA* | 549 | 82 | 84 | 83 | 83 | 84 | 84 | 83 | 80 | 82 | 83 | 83 | 83 | 90 | 96 | 96 | 96 | 100 | 93 | 93 | 93 | 93 | 92 | 100 | 100 | 100 | 96 | 92 |
| Rv1309 | *atpG* | 305 | 68 | 68 | 64 | 66 | 69 | 67 | 70 | 69 | 72 | 72 | 71 | 71 | 84 | 91 | 94 | 91 | 100 | 86 | 87 | 87 | 87 | 86 | 100 | 100 | 100 | 93 | 86 |
| Rv1310 | *atpD* | 486 | 86 | 84 | 86 | 83 | 86 | 86 | 84 | 81 | 86 | 87 | 87 | 87 | 97 | 98 | 96 | 96 | 100 | 95 | 95 | 95 | 95 | 96 | 100 | 100 | 100 | 96 | 96 |
| Rv1311 | *atpC* | 121 | 67 | 65 | 66 | 66 | 65 | 65 | 67 | 57 | 65 | 62 | 63 | 63 | 85 | 94 | 94 | 94 | 100 | 88 | 88 | 88 | 88 | 87 | 100 | 100 | 100 | 89 | 87 |
| Rv1312 | - | 147 | 54 | 57 | 55 | 55 | 54 | 0 | 51 | 0 | 72 | 73 | 70 | 70 | 81 | 95 | 96 | 95 | 99 | 87 | 91 | 91 | 91 | 89 | 100 | 100 | 100 | 96 | 88 |
| Rv1313c | - | 444 | 0 | 0 | 0 | 0 | 0 | 0 | 0 | 65 | 0 | 0 | 58 | 59 | 0 | 0 | 0 | 0 | 98 | 48 | 0 | 0 | 0 | 46 | 100 | 100 | 100 | 0 | 0 |
| Rv1314c | - | 193 | 0 | 81 | 79 | 75 | 0 | 77 | 0 | 77 | 85 | 86 | 85 | 84 | 83 | 89 | 90 | 90 | 100 | 88 | 86 | 86 | 86 | 88 | 100 | 100 | 100 | 87 | 89 |
| Rv1315 | *murA* | 418 | 79 | 79 | 78 | 78 | 82 | 80 | 77 | 45 | 85 | 84 | 86 | 86 | 93 | 98 | 98 | 98 | 100 | 95 | 95 | 95 | 95 | 95 | 100 | 100 | 100 | 98 | 96 |
| Rv1316c | *ogt* | 165 | 54 | 54 | 50 | 55 | 62 | 49 | 53 | 61 | 52 | 63 | 68 | 66 | 56 | 90 | 87 | 90 | 100 | 78 | 79 | 80 | 80 | 79 | 100 | 100 | 100 | 87 | 78 |
| Rv1317c | *alkA* | 496 | 0 | 0 | 0 | 0 | 0 | 0 | 0 | 64 | 61 | 51 | 52 | 53 | 60 | 87 | 89 | 87 | 99 | 80 | 83 | 83 | 83 | 85 | 100 | 100 | 100 | 88 | 84 |
| Rv1318c | - | 541 | 55 | 55 | 54 | 56 | 56 | 61 | 55 | 0 | 56 | 64 | 66 | 65 | 72 | 85 | 86 | 86 | 100 | 80 | 82 | 82 | 82 | 78 | 100 | 100 | 99 | 81 | 81 |
| Rv1319c | - | 535 | 56 | 56 | 55 | 56 | 57 | 54 | 55 | 0 | 54 | 64 | 64 | 63 | 72 | 81 | 83 | 81 | 100 | 79 | 80 | 80 | 80 | 78 | 99 | 100 | 100 | 78 | 79 |
| Rv1320c | - | 567 | 55 | 54 | 55 | 55 | 57 | 54 | 57 | 0 | 54 | 64 | 66 | 65 | 71 | 78 | 78 | 78 | 99 | 75 | 78 | 78 | 78 | 72 | 99 | 100 | 99 | 78 | 76 |
| Rv1321 | - | 226 | 78 | 84 | 83 | 81 | 75 | 73 | 73 | 78 | 84 | 87 | 89 | 88 | 87 | 96 | 95 | 96 | 99 | 94 | 91 | 91 | 91 | 90 | 99 | 100 | 99 | 95 | 93 |
| Rv1322 | - | 98 | 58 | 52 | 50 | 51 | 0 | 0 | 0 | 0 | 59 | 68 | 62 | 62 | 74 | 82 | 83 | 84 | 100 | 75 | 79 | 79 | 79 | 80 | 100 | 100 | 100 | 85 | 75 |
| Rv1322A | - | 152 | 0 | 73 | 70 | 73 | 0 | 73 | 0 | 81 | 80 | 76 | 84 | 84 | 81 | 87 | 89 | 87 | 100 | 86 | 86 | 86 | 86 | 85 | 100 | 100 | 100 | 89 | 87 |

| | | | | | | | | | | | | | | | | | | | | | | | | | | | | | |
|---|---|---|---|---|---|---|---|---|---|---|---|---|---|---|---|---|---|---|---|---|---|---|---|---|---|---|---|---|---|
| Rv1323 | *fadA4* | 389 | 66 | 0 | 66 | 53 | 67 | 69 | 66 | 80 | 84 | 85 | 85 | 85 | 86 | 95 | 95 | 95 | 100 | 90 | 93 | 93 | 93 | 93 | 100 | 100 | 100 | 94 | 91 |
| Rv1324 | - | 304 | 60 | 56 | 57 | 57 | 52 | 54 | 58 | 50 | 66 | 67 | 64 | 64 | 71 | 85 | 88 | 85 | 100 | 75 | 79 | 79 | 79 | 81 | 100 | 100 | 100 | 88 | 77 |
| Rv1325c | *PE_PGRS24* | 603 | 38 | 50 | 36 | 35 | 33 | 32 | 37 | 38 | 38 | 42 | 43 | 43 | 40 | 51 | 62 | 52 | 99 | 49 | 47 | 47 | 47 | 46 | 99 | 100 | 100 | 61 | 54 |
| Rv1326c | *glgB* | 731 | 73 | 72 | 71 | 71 | 70 | 73 | 0 | 70 | 78 | 81 | 81 | 81 | 86 | 93 | 91 | 91 | 99 | 88 | 88 | 88 | 88 | 89 | 99 | 100 | 100 | 93 | 90 |
| Rv1327c | *glgE* | 701 | 68 | 70 | 71 | 70 | 69 | 69 | 0 | 62 | 75 | 75 | 74 | 74 | 80 | 91 | 90 | 92 | 100 | 83 | 83 | 83 | 83 | 85 | 100 | 100 | 100 | 90 | 84 |
| Rv1328 | *glgP* | 863 | 38 | 0 | 64 | 63 | 37 | 0 | 0 | 70 | 81 | 78 | 78 | 77 | 82 | 89 | 91 | 89 | 99 | 84 | 88 | 88 | 88 | 87 | 100 | 100 | 100 | 91 | 85 |
| Rv1329c | *dinG* | 664 | 65 | 67 | 67 | 69 | 64 | 64 | 64 | 62 | 71 | 72 | 73 | 73 | 77 | 89 | 87 | 89 | 100 | 83 | 83 | 83 | 83 | 79 | 100 | 100 | 100 | 87 | 83 |
| Rv1330c | - | 448 | 72 | 72 | 74 | 75 | 73 | 73 | 74 | 70 | 80 | 80 | 78 | 76 | 82 | 88 | 85 | 87 | 99 | 80 | 83 | 83 | 83 | 83 | 99 | 100 | 99 | 84 | 80 |
| Rv1331 | *clpS* | 101 | 73 | 84 | 81 | 84 | 71 | 71 | 71 | 71 | 88 | 78 | 78 | 79 | 83 | 90 | 90 | 90 | 100 | 82 | 87 | 87 | 87 | 88 | 100 | 100 | 100 | 90 | 85 |
| Rv1332 | - | 218 | 50 | 49 | 50 | 50 | 50 | 47 | 49 | 45 | 64 | 67 | 64 | 63 | 75 | 88 | 89 | 89 | 100 | 76 | 75 | 78 | 78 | 81 | 100 | 100 | 100 | 89 | 75 |
| Rv1333 | - | 344 | 0 | 57 | 56 | 55 | 54 | 0 | 53 | 0 | 70 | 70 | 69 | 70 | 69 | 85 | 84 | 85 | 100 | 79 | 82 | 82 | 82 | 81 | 100 | 100 | 100 | 84 | 80 |
| Rv1334 | - | 146 | 0 | 0 | 0 | 0 | 0 | 0 | 0 | 73 | 88 | 0 | 91 | 91 | 89 | 0 | 89 | 0 | 100 | 91 | 91 | 91 | 91 | 87 | 100 | 100 | 100 | 89 | 89 |
| Rv1335 | - | 93 | 0 | 0 | 0 | 0 | 0 | 0 | 0 | 76 | 80 | 0 | 82 | 82 | 88 | 0 | 91 | 0 | 100 | 91 | 88 | 88 | 88 | 90 | 100 | 100 | 100 | 90 | 89 |
| Rv1336 | *cysM* | 323 | 55 | 55 | 54 | 54 | 53 | 54 | 53 | 82 | 86 | 56 | 84 | 83 | 93 | 56 | 85 | 56 | 100 | 91 | 90 | 90 | 90 | 92 | 100 | 100 | 100 | 85 | 89 |
| Rv1337 | - | 240 | 59 | 58 | 56 | 56 | 0 | 0 | 60 | 0 | 61 | 63 | 68 | 68 | 72 | 82 | 83 | 83 | 100 | 75 | 81 | 81 | 81 | 82 | 100 | 100 | 100 | 86 | 79 |
| Rv1338 | *murI* | 271 | 79 | 80 | 83 | 83 | 77 | 69 | 78 | 74 | 43 | 43 | 42 | 44 | 90 | 93 | 95 | 93 | 100 | 91 | 90 | 91 | 91 | 92 | 100 | 100 | 100 | 95 | 90 |
| Rv1339 | - | 273 | 59 | 65 | 64 | 63 | 57 | 58 | 56 | 62 | 75 | 78 | 79 | 79 | 84 | 91 | 92 | 91 | 100 | 88 | 88 | 88 | 88 | 85 | 100 | 100 | 100 | 92 | 88 |
| Rv1340 | *rph* | 259 | 77 | 77 | 78 | 78 | 78 | 79 | 77 | 79 | 90 | 88 | 88 | 88 | 91 | 97 | 96 | 96 | 99 | 90 | 91 | 91 | 91 | 93 | 100 | 100 | 100 | 96 | 91 |
| Rv1341 | - | 204 | 71 | 68 | 72 | 67 | 66 | 58 | 68 | 62 | 81 | 80 | 81 | 80 | 80 | 90 | 89 | 89 | 100 | 86 | 86 | 86 | 86 | 88 | 100 | 100 | 100 | 89 | 86 |
| Rv1342c | - | 120 | 52 | 52 | 58 | 62 | 53 | 57 | 52 | 0 | 72 | 61 | 78 | 78 | 76 | 81 | 87 | 81 | 100 | 78 | 78 | 78 | 78 | 83 | 100 | 100 | 100 | 87 | 78 |
| Rv1343c | *lprD* | 126 | 76 | 57 | 57 | 71 | 52 | 41 | 53 | 0 | 64 | 65 | 66 | 63 | 70 | 85 | 85 | 85 | 99 | 71 | 76 | 75 | 75 | 74 | 100 | 100 | 100 | 85 | 73 |
| Rv1344 | - | 106 | 0 | 0 | 0 | 0 | 0 | 0 | 0 | 0 | 0 | 0 | 0 | 0 | 0 | 78 | 0 | 90 | 100 | 76 | 88 | 88 | 88 | 81 | 100 | 100 | 100 | 0 | 87 |
| Rv1345 | *fadD33* | 521 | 41 | 42 | 45 | 44 | 45 | 41 | 43 | 46 | 48 | 49 | 50 | 50 | 50 | 79 | 50 | 80 | 100 | 74 | 75 | 75 | 75 | 77 | 98 | 100 | 100 | 50 | 76 |
| Rv1346 | *fadE14* | 386 | 41 | 0 | 41 | 0 | 46 | 44 | 46 | 45 | 45 | 45 | 44 | 45 | 44 | 90 | 43 | 90 | 100 | 45 | 85 | 85 | 85 | 87 | 100 | 100 | 100 | 43 | 87 |
| Rv1347c | - | 210 | 0 | 0 | 0 | 0 | 47 | 53 | 0 | 0 | 53 | 0 | 0 | 61 | 57 | 71 | 65 | 87 | 100 | 74 | 73 | 73 | 73 | 73 | 100 | 100 | 99 | 65 | 72 |
| Rv1348 | - | 859 | 52 | 52 | 49 | 51 | 52 | 48 | 45 | 49 | 61 | 55 | 51 | 51 | 76 | 86 | 85 | 86 | 100 | 51 | 51 | 51 | 51 | 83 | 100 | 100 | 100 | 84 | 51 |
| Rv1349 | - | 579 | 50 | 51 | 46 | 47 | 50 | 48 | 56 | 50 | 56 | 52 | 47 | 47 | 72 | 86 | 86 | 86 | 100 | 51 | 46 | 45 | 45 | 82 | 100 | 100 | 100 | 86 | 50 |
| Rv1350 | *fabG* | 247 | 55 | 44 | 57 | 54 | 55 | 58 | 53 | 63 | 61 | 81 | 76 | 77 | 61 | 95 | 95 | 95 | 100 | 89 | 93 | 93 | 93 | 91 | 100 | 100 | 100 | 95 | 91 |
| Rv1351 | - | 109 | 0 | 0 | 0 | 0 | 0 | 0 | 0 | 0 | 0 | 0 | 0 | 0 | 0 | 0 | 0 | 0 | 100 | 0 | 0 | 0 | 0 | 0 | 100 | 100 | 100 | 0 | 0 |
| Rv1352 | - | 123 | 0 | 0 | 0 | 0 | 0 | 0 | 0 | 0 | 0 | 0 | 0 | 0 | 0 | 61 | 61 | 61 | 100 | 61 | 0 | 0 | 0 | 60 | 100 | 100 | 100 | 79 | 64 |
| Rv1353c | - | 261 | 0 | 0 | 0 | 0 | 0 | 0 | 0 | 0 | 41 | 0 | 46 | 0 | 63 | 84 | 85 | 84 | 100 | 80 | 76 | 76 | 76 | 83 | 100 | 100 | 99 | 85 | 80 |
| Rv1354c | - | 623 | 0 | 0 | 0 | 48 | 0 | 0 | 0 | 54 | 71 | 51 | 67 | 68 | 44 | 70 | 0 | 72 | 100 | 78 | 76 | 76 | 76 | 75 | 100 | 100 | 100 | 0 | 78 |
| Rv1355c | *moeY* | 715 | 47 | 54 | 49 | 57 | 0 | 0 | 41 | 48 | 63 | 0 | 67 | 68 | 0 | 73 | 69 | 73 | 99 | 66 | 71 | 71 | 71 | 69 | 100 | 100 | 100 | 70 | 70 |
| Rv1356c | - | 263 | 0 | 0 | 0 | 0 | 0 | 0 | 0 | 0 | 59 | 0 | 61 | 61 | 0 | 72 | 72 | 72 | 100 | 60 | 66 | 66 | 66 | 67 | 100 | 100 | 100 | 0 | 66 |
| Rv1357c | - | 307 | 0 | 0 | 0 | 42 | 0 | 0 | 0 | 54 | 49 | 54 | 55 | 55 | 0 | 51 | 0 | 53 | 100 | 53 | 56 | 56 | 56 | 51 | 100 | 100 | 100 | 0 | 51 |
| Rv1358 | - | 1159 | 0 | 0 | 0 | 0 | 0 | 0 | 0 | 41 | 47 | 58 | 47 | 53 | 0 | 47 | 66 | 47 | 99 | 0 | 38 | 38 | 38 | 53 | 99 | 100 | 100 | 48 | 67 |
| Rv1359 | - | 250 | 0 | 0 | 0 | 0 | 0 | 0 | 0 | 0 | 0 | 0 | 0 | 0 | 0 | 46 | 46 | 46 | 99 | 0 | 0 | 0 | 0 | 48 | 100 | 100 | 100 | 48 | 60 |
| Rv1360 | - | 340 | 0 | 0 | 0 | 0 | 0 | 0 | 0 | 44 | 63 | 59 | 42 | 66 | 70 | 59 | 60 | 59 | 100 | 75 | 79 | 79 | 79 | 79 | 100 | 100 | 100 | 59 | 75 |

| | | | | | | | | | | | | | | | | | | | | | | | | | | | | | |
|---|---|---|---|---|---|---|---|---|---|---|---|---|---|---|---|---|---|---|---|---|---|---|---|---|---|---|---|---|---|
| Rv1361c | *PPE19* | 396 | 0 | 0 | 0 | 0 | 0 | 0 | 0 | 0 | 0 | 40 | 0 | 0 | 43 | 55 | 70 | 56 | 95 | 56 | 52 | 52 | 52 | 51 | 99 | 100 | 100 | 69 | 47 |
| Rv1362c | - | 220 | 0 | 0 | 0 | 0 | 0 | 0 | 0 | 46 | 46 | 48 | 59 | 60 | 44 | 74 | 79 | 79 | 99 | 66 | 67 | 66 | 66 | 64 | 100 | 100 | 100 | 78 | 63 |
| Rv1363c | - | 261 | 0 | 0 | 0 | 0 | 0 | 0 | 0 | 48 | 53 | 42 | 64 | 61 | 49 | 69 | 76 | 69 | 99 | 63 | 64 | 64 | 64 | 64 | 95 | 100 | 100 | 76 | 63 |
| Rv1364c | - | 653 | 0 | 0 | 45 | 0 | 0 | 0 | 0 | 49 | 45 | 0 | 43 | 0 | 0 | 86 | 88 | 87 | 99 | 49 | 47 | 47 | 47 | 44 | 99 | 100 | 99 | 88 | 49 |
| Rv1365c | *rsfA* | 128 | 0 | 0 | 0 | 0 | 0 | 0 | 0 | 0 | 51 | 0 | 0 | 0 | 0 | 75 | 70 | 74 | 100 | 0 | 0 | 0 | 0 | 0 | 100 | 100 | 100 | 69 | 0 |
| Rv1366 | - | 273 | 53 | 0 | 0 | 0 | 0 | 0 | 0 | 0 | 0 | 0 | 0 | 0 | 0 | 0 | 0 | 0 | 100 | 0 | 0 | 0 | 0 | 0 | 100 | 100 | 100 | 0 | 0 |
| Rv1367c | - | 377 | 0 | 0 | 0 | 0 | 0 | 0 | 0 | 0 | 38 | 48 | 48 | 49 | 62 | 91 | 92 | 91 | 100 | 80 | 80 | 80 | 80 | 81 | 100 | 100 | 100 | 92 | 81 |
| Rv1368 | *lprF* | 261 | 0 | 0 | 0 | 0 | 0 | 0 | 0 | 0 | 67 | 0 | 0 | 0 | 49 | 52 | 52 | 52 | 100 | 51 | 54 | 54 | 54 | 50 | 100 | 100 | 100 | 76 | 48 |
| Rv1369c | - | 294 | 58 | 49 | 61 | 51 | 63 | 0 | 61 | 56 | 0 | 0 | 53 | 52 | 66 | 60 | 66 | 68 | 100 | 66 | 90 | 67 | 67 | 66 | 100 | 100 | 100 | 0 | 65 |
| Rv1370c | - | 108 | 65 | 0 | 66 | 0 | 61 | 0 | 60 | 76 | 0 | 0 | 0 | 0 | 59 | 63 | 66 | 66 | 100 | 82 | 96 | 77 | 77 | 84 | 100 | 100 | 100 | 0 | 62 |
| Rv1371 | - | 489 | 0 | 0 | 0 | 0 | 0 | 0 | 0 | 0 | 85 | 0 | 0 | 0 | 0 | 0 | 91 | 0 | 100 | 0 | 0 | 0 | 0 | 0 | 100 | 100 | 100 | 91 | 0 |
| Rv1372 | - | 393 | 0 | 0 | 0 | 0 | 0 | 0 | 0 | 0 | 80 | 40 | 0 | 0 | 0 | 44 | 86 | 44 | 100 | 45 | 47 | 47 | 47 | 45 | 100 | 100 | 100 | 85 | 47 |
| Rv1373 | - | 326 | 0 | 0 | 0 | 0 | 0 | 0 | 0 | 0 | 0 | 0 | 0 | 0 | 0 | 0 | 71 | 0 | 96 | 0 | 0 | 0 | 0 | 0 | 99 | 100 | 100 | 70 | 0 |
| Rv1374c | - | 152 | 0 | 0 | 0 | 0 | 0 | 0 | 0 | 0 | 0 | 0 | 0 | 0 | 0 | 0 | 0 | 0 | 99 | 0 | 0 | 0 | 0 | 0 | 99 | 100 | 99 | 0 | 0 |
| Rv1375 | - | 439 | 0 | 0 | 0 | 0 | 0 | 0 | 0 | 0 | 0 | 0 | 0 | 0 | 0 | 0 | 80 | 0 | 99 | 0 | 0 | 0 | 0 | 0 | 100 | 100 | 100 | 80 | 0 |
| Rv1376 | - | 497 | 0 | 0 | 0 | 0 | 0 | 0 | 0 | 0 | 0 | 0 | 0 | 0 | 0 | 0 | 78 | 0 | 99 | 0 | 0 | 0 | 0 | 0 | 100 | 100 | 100 | 78 | 0 |
| Rv1377c | - | 212 | 0 | 0 | 0 | 0 | 0 | 0 | 0 | 56 | 45 | 51 | 54 | 59 | 61 | 54 | 54 | 54 | 100 | 54 | 52 | 52 | 52 | 72 | 100 | 100 | 100 | 83 | 50 |
| Rv1378c | - | 475 | 0 | 0 | 0 | 0 | 0 | 0 | 0 | 59 | 0 | 62 | 65 | 64 | 74 | 80 | 82 | 79 | 99 | 71 | 83 | 79 | 79 | 72 | 99 | 100 | 99 | 87 | 68 |
| Rv1379 | *pyrR* | 193 | 79 | 77 | 79 | 80 | 74 | 78 | 77 | 71 | 84 | 84 | 86 | 86 | 86 | 90 | 88 | 90 | 100 | 89 | 85 | 85 | 85 | 88 | 100 | 100 | 100 | 88 | 89 |
| Rv1380 | *pyrB* | 319 | 77 | 80 | 83 | 82 | 80 | 82 | 82 | 76 | 86 | 89 | 90 | 90 | 90 | 94 | 93 | 94 | 100 | 92 | 93 | 93 | 93 | 91 | 100 | 100 | 100 | 92 | 91 |
| Rv1381 | *pyrC* | 430 | 73 | 75 | 74 | 74 | 75 | 76 | 76 | 69 | 86 | 86 | 83 | 85 | 88 | 95 | 94 | 95 | 99 | 88 | 92 | 92 | 92 | 92 | 100 | 100 | 100 | 94 | 89 |
| Rv1382 | - | 165 | 0 | 0 | 0 | 0 | 0 | 0 | 0 | 0 | 74 | 67 | 67 | 68 | 67 | 83 | 83 | 83 | 100 | 76 | 76 | 76 | 76 | 73 | 100 | 100 | 100 | 80 | 77 |
| Rv1383 | *carA* | 376 | 76 | 80 | 77 | 76 | 79 | 75 | 73 | 73 | 82 | 82 | 84 | 83 | 85 | 93 | 91 | 93 | 99 | 89 | 89 | 89 | 89 | 89 | 100 | 100 | 100 | 91 | 90 |
| Rv1384 | *carB* | 1115 | 84 | 84 | 84 | 84 | 83 | 83 | 83 | 78 | 89 | 87 | 88 | 88 | 92 | 96 | 96 | 96 | 99 | 92 | 93 | 93 | 93 | 94 | 100 | 100 | 100 | 96 | 93 |
| Rv1385 | *pyrF* | 274 | 66 | 66 | 67 | 66 | 66 | 59 | 63 | 63 | 71 | 69 | 69 | 69 | 77 | 85 | 85 | 85 | 100 | 81 | 83 | 82 | 82 | 76 | 100 | 100 | 100 | 84 | 81 |
| Rv1386 | *PE15* | 102 | 0 | 0 | 0 | 0 | 0 | 0 | 0 | 0 | 0 | 0 | 0 | 0 | 73 | 80 | 80 | 80 | 100 | 80 | 81 | 81 | 81 | 81 | 100 | 100 | 100 | 78 | 78 |
| Rv1387 | *PPE20* | 539 | 0 | 0 | 0 | 0 | 0 | 0 | 0 | 0 | 0 | 47 | 44 | 43 | 51 | 60 | 62 | 60 | 99 | 47 | 50 | 50 | 50 | 50 | 100 | 100 | 100 | 62 | 48 |
| Rv1388 | *mihF* | 190 | 88 | 85 | 88 | 88 | 93 | 91 | 92 | 77 | 94 | 94 | 96 | 96 | 77 | 99 | 88 | 99 | 100 | 96 | 99 | 99 | 99 | 80 | 100 | 100 | 100 | 87 | 99 |
| Rv1389 | *gmk* | 208 | 76 | 74 | 77 | 74 | 75 | 76 | 71 | 69 | 78 | 79 | 70 | 76 | 79 | 89 | 87 | 89 | 100 | 82 | 84 | 83 | 83 | 82 | 100 | 100 | 100 | 86 | 82 |
| Rv1390 | *rpoZ* | 110 | 81 | 79 | 72 | 85 | 87 | 83 | 88 | 88 | 98 | 95 | 88 | 89 | 80 | 94 | 94 | 94 | 100 | 86 | 84 | 84 | 84 | 86 | 100 | 100 | 100 | 94 | 84 |
| Rv1391 | *dfp* | 418 | 74 | 70 | 72 | 72 | 75 | 65 | 70 | 71 | 83 | 83 | 85 | 85 | 88 | 94 | 94 | 92 | 100 | 89 | 89 | 89 | 89 | 91 | 100 | 100 | 100 | 94 | 90 |
| Rv1392 | *metK* | 403 | 77 | 79 | 80 | 80 | 80 | 80 | 79 | 80 | 87 | 87 | 86 | 86 | 90 | 96 | 97 | 96 | 99 | 92 | 92 | 92 | 92 | 91 | 100 | 100 | 100 | 97 | 92 |
| Rv1393c | - | 492 | 50 | 0 | 45 | 49 | 42 | 42 | 48 | 56 | 76 | 62 | 54 | 58 | 65 | 89 | 90 | 90 | 100 | 78 | 84 | 84 | 84 | 59 | 100 | 100 | 100 | 89 | 82 |
| Rv1394c | *cyp132* | 461 | 0 | 0 | 0 | 0 | 0 | 0 | 0 | 46 | 50 | 43 | 43 | 42 | 48 | 42 | 41 | 41 | 99 | 45 | 43 | 43 | 43 | 43 | 99 | 100 | 99 | 46 | 43 |
| Rv1395 | - | 344 | 0 | 0 | 0 | 0 | 0 | 0 | 0 | 0 | 54 | 0 | 39 | 41 | 44 | 0 | 40 | 0 | 100 | 44 | 44 | 44 | 44 | 0 | 100 | 100 | 100 | 61 | 0 |
| Rv1396c | *PE_PGRS25* | 576 | 44 | 34 | 35 | 34 | 34 | 39 | 38 | 38 | 38 | 40 | 42 | 43 | 42 | 46 | 34 | 34 | 86 | 51 | 55 | 55 | 55 | 44 | 92 | 100 | 73 | 60 | 54 |
| Rv1397c | - | 133 | 0 | 0 | 0 | 0 | 0 | 0 | 0 | 0 | 0 | 0 | 0 | 0 | 0 | 0 | 0 | 0 | 99 | 0 | 0 | 0 | 0 | 0 | 100 | 100 | 100 | 0 | 0 |
| Rv1398c | - | 85 | 0 | 0 | 0 | 0 | 0 | 0 | 0 | 0 | 0 | 0 | 0 | 0 | 0 | 0 | 0 | 0 | 100 | 0 | 0 | 0 | 0 | 0 | 100 | 100 | 100 | 0 | 0 |

| | | | | | | | | | | | | | | | | | | | | | | | | | | | | | |
|---|---|---|---|---|---|---|---|---|---|---|---|---|---|---|---|---|---|---|---|---|---|---|---|---|---|---|---|---|---|
| Rv1399c | *lipH* | 319 | 50 | 50 | 0 | 53 | 55 | 0 | 0 | 56 | 54 | 55 | 54 | 55 | 66 | 70 | 86 | 70 | 100 | 69 | 69 | 69 | 69 | 70 | 100 | 100 | 100 | 85 | 68 |
| Rv1400c | *lipI* | 320 | 48 | 0 | 0 | 49 | 51 | 0 | 0 | 56 | 54 | 56 | 56 | 60 | 68 | 84 | 86 | 84 | 100 | 78 | 78 | 78 | 78 | 79 | 99 | 100 | 100 | 86 | 79 |
| Rv1401 | - | 200 | 0 | 0 | 0 | 0 | 0 | 0 | 0 | 0 | 0 | 0 | 0 | 0 | 0 | 80 | 84 | 81 | 100 | 73 | 74 | 74 | 74 | 71 | 100 | 100 | 100 | 83 | 76 |
| Rv1402 | *priA* | 655 | 61 | 63 | 63 | 62 | 57 | 57 | 60 | 59 | 66 | 70 | 70 | 70 | 76 | 86 | 88 | 86 | 100 | 80 | 80 | 80 | 80 | 80 | 99 | 100 | 100 | 88 | 80 |
| Rv1403c | - | 274 | 0 | 0 | 0 | 0 | 0 | 0 | 0 | 0 | 52 | 66 | 70 | 69 | 0 | 70 | 86 | 70 | 100 | 62 | 67 | 66 | 66 | 66 | 100 | 100 | 100 | 85 | 63 |
| Rv1404 | - | 160 | 0 | 0 | 0 | 0 | 0 | 0 | 0 | 52 | 48 | 0 | 0 | 51 | 76 | 86 | 88 | 86 | 100 | 0 | 60 | 60 | 60 | 50 | 100 | 100 | 100 | 87 | 0 |
| Rv1405c | - | 274 | 0 | 0 | 0 | 0 | 0 | 0 | 0 | 57 | 45 | 65 | 70 | 69 | 0 | 84 | 84 | 84 | 100 | 61 | 70 | 70 | 70 | 68 | 100 | 100 | 100 | 90 | 64 |
| Rv1406 | *fmt* | 312 | 66 | 67 | 69 | 67 | 60 | 62 | 59 | 70 | 73 | 75 | 73 | 73 | 73 | 89 | 90 | 89 | 100 | 81 | 83 | 83 | 83 | 87 | 100 | 100 | 100 | 89 | 84 |
| Rv1407 | *fmu* | 457 | 63 | 65 | 66 | 66 | 63 | 65 | 63 | 62 | 71 | 74 | 73 | 73 | 78 | 88 | 90 | 88 | 100 | 84 | 86 | 86 | 86 | 85 | 100 | 100 | 100 | 89 | 83 |
| Rv1408 | *rpe* | 232 | 71 | 76 | 77 | 75 | 72 | 73 | 71 | 70 | 88 | 88 | 89 | 89 | 91 | 91 | 93 | 91 | 100 | 91 | 91 | 91 | 91 | 91 | 100 | 100 | 100 | 93 | 90 |
| Rv1409 | *ribG* | 339 | 60 | 58 | 60 | 62 | 59 | 65 | 60 | 63 | 0 | 70 | 74 | 73 | 80 | 88 | 94 | 88 | 99 | 84 | 84 | 84 | 84 | 86 | 100 | 100 | 100 | 94 | 83 |
| Rv1410c | - | 518 | 44 | 44 | 46 | 47 | 46 | 43 | 43 | 57 | 46 | 45 | 43 | 44 | 77 | 90 | 93 | 90 | 100 | 81 | 83 | 83 | 83 | 81 | 100 | 100 | 100 | 93 | 83 |
| Rv1411c | *lprG* | 236 | 0 | 0 | 0 | 0 | 0 | 0 | 0 | 43 | 51 | 0 | 0 | 0 | 58 | 82 | 89 | 82 | 100 | 67 | 68 | 68 | 68 | 68 | 100 | 100 | 100 | 88 | 68 |
| Rv1412 | *ribC* | 201 | 71 | 72 | 71 | 72 | 64 | 61 | 66 | 68 | 82 | 87 | 87 | 87 | 85 | 90 | 92 | 90 | 100 | 86 | 88 | 88 | 88 | 84 | 100 | 100 | 100 | 92 | 85 |
| Rv1413 | - | 171 | 0 | 0 | 0 | 52 | 0 | 0 | 0 | 63 | 0 | 0 | 0 | 0 | 61 | 0 | 0 | 0 | 100 | 0 | 0 | 0 | 0 | 0 | 99 | 100 | 100 | 0 | 0 |
| Rv1414 | - | 133 | 0 | 0 | 0 | 64 | 0 | 0 | 0 | 63 | 0 | 0 | 0 | 0 | 66 | 0 | 0 | 0 | 100 | 0 | 0 | 0 | 0 | 0 | 100 | 100 | 100 | 0 | 0 |
| Rv1415 | *ribA2* | 425 | 76 | 75 | 80 | 79 | 78 | 75 | 73 | 77 | 90 | 93 | 92 | 92 | 95 | 99 | 98 | 99 | 100 | 96 | 95 | 95 | 95 | 96 | 100 | 100 | 100 | 98 | 96 |
| Rv1416 | *ribH* | 154 | 72 | 74 | 76 | 76 | 64 | 74 | 66 | 73 | 78 | 79 | 80 | 81 | 85 | 92 | 92 | 92 | 100 | 87 | 87 | 87 | 87 | 89 | 100 | 100 | 100 | 91 | 92 |
| Rv1417 | - | 154 | 58 | 59 | 51 | 52 | 53 | 54 | 52 | 0 | 65 | 67 | 67 | 67 | 79 | 87 | 89 | 86 | 100 | 82 | 83 | 83 | 83 | 81 | 100 | 100 | 100 | 89 | 77 |
| Rv1418 | *lprH* | 228 | 0 | 0 | 0 | 0 | 0 | 0 | 0 | 0 | 0 | 0 | 0 | 0 | 0 | 0 | 71 | 0 | 100 | 0 | 0 | 0 | 0 | 0 | 100 | 100 | 100 | 71 | 0 |
| Rv1419 | - | 157 | 0 | 0 | 0 | 0 | 0 | 0 | 0 | 0 | 0 | 0 | 0 | 0 | 0 | 0 | 0 | 0 | 100 | 0 | 0 | 0 | 0 | 0 | 100 | 100 | 100 | 82 | 0 |
| Rv1420 | *uvrC* | 646 | 73 | 74 | 75 | 74 | 71 | 70 | 72 | 76 | 82 | 82 | 82 | 81 | 88 | 94 | 93 | 94 | 100 | 87 | 88 | 89 | 89 | 90 | 100 | 100 | 100 | 93 | 87 |
| Rv1421 | - | 301 | 65 | 66 | 67 | 68 | 67 | 66 | 69 | 67 | 74 | 76 | 78 | 78 | 88 | 92 | 90 | 92 | 100 | 88 | 88 | 88 | 88 | 88 | 100 | 100 | 100 | 90 | 89 |
| Rv1422 | - | 342 | 71 | 70 | 69 | 72 | 71 | 72 | 69 | 60 | 82 | 80 | 78 | 77 | 81 | 90 | 93 | 91 | 99 | 89 | 90 | 90 | 90 | 89 | 100 | 100 | 100 | 93 | 88 |
| Rv1423 | *whiA* | 325 | 77 | 76 | 81 | 80 | 76 | 71 | 78 | 83 | 94 | 93 | 94 | 94 | 95 | 98 | 99 | 98 | 100 | 96 | 96 | 96 | 96 | 96 | 100 | 100 | 100 | 98 | 95 |
| Rv1424c | - | 253 | 0 | 0 | 0 | 0 | 0 | 0 | 0 | 0 | 0 | 0 | 0 | 0 | 0 | 0 | 0 | 0 | 100 | 0 | 0 | 0 | 0 | 0 | 100 | 100 | 100 | 0 | 0 |
| Rv1425 | - | 459 | 0 | 0 | 0 | 0 | 0 | 0 | 0 | 52 | 56 | 56 | 58 | 58 | 86 | 91 | 96 | 92 | 99 | 81 | 57 | 57 | 57 | 56 | 100 | 100 | 100 | 96 | 56 |
| Rv1426c | *lipO* | 420 | 0 | 0 | 0 | 0 | 0 | 0 | 0 | 57 | 39 | 39 | 60 | 59 | 43 | 86 | 87 | 86 | 100 | 74 | 74 | 74 | 74 | 70 | 98 | 100 | 100 | 86 | 74 |
| Rv1427c | *fadD12* | 535 | 54 | 52 | 43 | 43 | 53 | 48 | 50 | 61 | 58 | 56 | 57 | 56 | 59 | 90 | 91 | 90 | 100 | 86 | 84 | 82 | 82 | 85 | 100 | 100 | 100 | 90 | 84 |
| Rv1428c | - | 275 | 0 | 0 | 0 | 0 | 0 | 0 | 0 | 51 | 53 | 51 | 50 | 50 | 48 | 91 | 93 | 91 | 100 | 81 | 83 | 83 | 83 | 81 | 100 | 100 | 100 | 93 | 79 |
| Rv1429 | - | 422 | 0 | 0 | 0 | 0 | 0 | 0 | 0 | 44 | 45 | 47 | 46 | 52 | 42 | 0 | 50 | 0 | 100 | 49 | 0 | 0 | 0 | 0 | 99 | 100 | 100 | 50 | 0 |
| Rv1430 | *PE16* | 528 | 0 | 0 | 0 | 0 | 0 | 0 | 0 | 0 | 0 | 41 | 0 | 0 | 50 | 68 | 68 | 68 | 100 | 48 | 49 | 49 | 49 | 51 | 100 | 100 | 100 | 60 | 47 |
| Rv1431 | - | 589 | 0 | 0 | 0 | 0 | 0 | 0 | 0 | 0 | 57 | 60 | 59 | 60 | 62 | 83 | 90 | 83 | 99 | 61 | 63 | 63 | 63 | 62 | 99 | 100 | 100 | 91 | 62 |
| Rv1432 | - | 473 | 76 | 0 | 59 | 59 | 0 | 59 | 0 | 43 | 73 | 80 | 81 | 83 | 73 | 86 | 92 | 87 | 100 | 56 | 54 | 54 | 54 | 57 | 100 | 100 | 100 | 92 | 56 |
| Rv1433 | - | 271 | 50 | 52 | 50 | 52 | 50 | 48 | 47 | 50 | 56 | 56 | 61 | 61 | 72 | 87 | 85 | 87 | 100 | 71 | 64 | 63 | 63 | 57 | 100 | 100 | 100 | 67 | 64 |
| Rv1434 | - | 45 | 0 | 0 | 0 | 0 | 0 | 0 | 0 | 0 | 0 | 0 | 0 | 0 | 0 | 0 | 0 | 0 | 100 | 0 | 0 | 0 | 0 | 0 | 95 | 100 | 100 | 0 | 0 |
| Rv1435c | - | 202 | 0 | 0 | 40 | 42 | 36 | 0 | 39 | 0 | 42 | 0 | 39 | 0 | 47 | 69 | 69 | 69 | 96 | 50 | 41 | 41 | 41 | 54 | 96 | 100 | 96 | 61 | 44 |
| Rv1436 | *gap* | 339 | 74 | 80 | 76 | 75 | 79 | 73 | 74 | 79 | 92 | 89 | 92 | 92 | 93 | 96 | 95 | 96 | 100 | 92 | 93 | 93 | 93 | 94 | 100 | 100 | 100 | 95 | 92 |

| | | | | | | | | | | | | | | | | | | | | | | | | | | | | | |
|---|---|---|---|---|---|---|---|---|---|---|---|---|---|---|---|---|---|---|---|---|---|---|---|---|---|---|---|---|---|
| Rv1437 | *pgk* | 412 | 74 | 77 | 76 | 75 | 77 | 73 | 77 | 67 | 82 | 80 | 81 | 81 | 84 | 88 | 91 | 88 | 100 | 86 | 85 | 85 | 85 | 87 | 100 | 100 | 100 | 90 | 85 |
| Rv1438 | *tpiA* | 261 | 78 | 81 | 83 | 82 | 79 | 82 | 84 | 77 | 88 | 86 | 86 | 86 | 91 | 96 | 94 | 95 | 100 | 89 | 92 | 92 | 92 | 89 | 100 | 100 | 100 | 94 | 91 |
| Rv1439c | - | 141 | 0 | 0 | 0 | 0 | 0 | 0 | 0 | 0 | 0 | 0 | 0 | 0 | 0 | 0 | 0 | 0 | 100 | 0 | 0 | 0 | 0 | 0 | 100 | 100 | 100 | 0 | 0 |
| Rv1440 | *secG* | 77 | 80 | 79 | 78 | 80 | 83 | 80 | 79 | 71 | 88 | 86 | 88 | 86 | 93 | 98 | 100 | 98 | 100 | 98 | 96 | 96 | 96 | 96 | 100 | 100 | 100 | 100 | 98 |
| Rv1441c | *PE_PGRS26* | 491 | 42 | 45 | 36 | 38 | 34 | 36 | 36 | 38 | 39 | 42 | 41 | 41 | 45 | 46 | 68 | 38 | 99 | 52 | 47 | 46 | 46 | 51 | 99 | 100 | 92 | 57 | 52 |
| Rv1442 | *bisC* | 766 | 0 | 0 | 0 | 0 | 0 | 0 | 46 | 36 | 37 | 76 | 75 | 75 | 80 | 38 | 37 | 38 | 100 | 78 | 80 | 80 | 80 | 81 | 100 | 100 | 100 | 38 | 77 |
| Rv1443c | - | 161 | 0 | 0 | 0 | 0 | 0 | 0 | 0 | 0 | 0 | 0 | 78 | 77 | 63 | 0 | 61 | 0 | 100 | 0 | 0 | 0 | 0 | 0 | 100 | 100 | 100 | 62 | 0 |
| Rv1444c | - | 136 | 0 | 0 | 0 | 0 | 0 | 0 | 0 | 0 | 0 | 0 | 57 | 0 | 0 | 85 | 92 | 85 | 100 | 67 | 65 | 65 | 65 | 69 | 100 | 100 | 100 | 92 | 70 |
| Rv1445c | *devB* | 247 | 56 | 58 | 58 | 61 | 57 | 60 | 59 | 60 | 72 | 70 | 69 | 68 | 78 | 86 | 86 | 86 | 100 | 78 | 78 | 78 | 78 | 79 | 100 | 100 | 100 | 90 | 77 |
| Rv1446c | *opcA* | 303 | 58 | 58 | 61 | 60 | 58 | 50 | 60 | 59 | 75 | 78 | 75 | 76 | 79 | 91 | 93 | 91 | 99 | 79 | 82 | 82 | 82 | 83 | 99 | 100 | 99 | 92 | 80 |
| Rv1447c | *zwf2* | 514 | 81 | 78 | 82 | 83 | 80 | 82 | 80 | 82 | 87 | 88 | 90 | 90 | 91 | 95 | 95 | 95 | 100 | 90 | 92 | 92 | 92 | 93 | 100 | 100 | 100 | 94 | 91 |
| Rv1448c | *tal* | 373 | 74 | 75 | 76 | 77 | 76 | 67 | 70 | 76 | 84 | 82 | 85 | 84 | 84 | 91 | 91 | 91 | 99 | 87 | 88 | 88 | 88 | 88 | 99 | 100 | 100 | 91 | 90 |
| Rv1449c | *tkt* | 700 | 77 | 76 | 77 | 76 | 77 | 76 | 76 | 75 | 80 | 79 | 82 | 82 | 87 | 93 | 93 | 93 | 99 | 91 | 91 | 91 | 91 | 89 | 99 | 100 | 99 | 94 | 91 |
| Rv1450c | *PE_PGRS27* | 1329 | 45 | 31 | 36 | 32 | 37 | 38 | 39 | 31 | 36 | 39 | 37 | 38 | 36 | 31 | 60 | 30 | 92 | 49 | 53 | 53 | 53 | 47 | 93 | 93 | 93 | 57 | 50 |
| Rv1451 | *ctaB* | 308 | 67 | 71 | 70 | 70 | 67 | 70 | 67 | 67 | 82 | 80 | 79 | 79 | 84 | 88 | 86 | 88 | 99 | 86 | 88 | 88 | 88 | 89 | 100 | 100 | 100 | 86 | 86 |
| Rv1452c | *PE_PGRS28* | 741 | 42 | 33 | 38 | 33 | 35 | 35 | 37 | 36 | 40 | 39 | 43 | 40 | 39 | 33 | 34 | 34 | 90 | 52 | 53 | 49 | 49 | 44 | 97 | 100 | 97 | 54 | 50 |
| Rv1453 | - | 421 | 0 | 0 | 0 | 0 | 0 | 0 | 0 | 40 | 50 | 43 | 51 | 49 | 51 | 0 | 83 | 0 | 99 | 41 | 0 | 0 | 0 | 0 | 99 | 100 | 99 | 82 | 0 |
| Rv1454c | *qor* | 328 | 59 | 44 | 58 | 62 | 42 | 64 | 42 | 48 | 74 | 75 | 73 | 72 | 72 | 84 | 83 | 83 | 100 | 78 | 82 | 81 | 81 | 82 | 100 | 100 | 100 | 90 | 81 |
| Rv1455 | - | 287 | 0 | 0 | 0 | 0 | 0 | 0 | 0 | 41 | 0 | 46 | 0 | 0 | 63 | 53 | 76 | 52 | 100 | 63 | 64 | 64 | 64 | 61 | 99 | 100 | 100 | 76 | 66 |
| Rv1456c | - | 310 | 61 | 58 | 63 | 62 | 63 | 64 | 62 | 55 | 79 | 77 | 81 | 80 | 81 | 89 | 87 | 89 | 100 | 85 | 83 | 83 | 82 | 80 | 100 | 100 | 100 | 87 | 84 |
| Rv1457c | - | 261 | 62 | 60 | 61 | 58 | 58 | 61 | 63 | 58 | 76 | 78 | 76 | 76 | 82 | 89 | 90 | 90 | 100 | 88 | 88 | 87 | 87 | 89 | 100 | 100 | 100 | 93 | 88 |
| Rv1458c | - | 313 | 68 | 69 | 64 | 67 | 69 | 66 | 64 | 65 | 85 | 79 | 82 | 82 | 90 | 95 | 95 | 95 | 100 | 90 | 93 | 93 | 93 | 92 | 100 | 100 | 100 | 95 | 92 |
| Rv1459c | - | 591 | 53 | 53 | 53 | 52 | 53 | 54 | 54 | 43 | 67 | 67 | 69 | 67 | 78 | 85 | 83 | 85 | 100 | 80 | 79 | 79 | 79 | 81 | 100 | 100 | 100 | 83 | 80 |
| Rv1460 | - | 268 | 67 | 66 | 67 | 67 | 61 | 61 | 64 | 58 | 69 | 71 | 69 | 69 | 72 | 84 | 88 | 84 | 99 | 77 | 80 | 80 | 80 | 77 | 100 | 100 | 100 | 89 | 80 |
| Rv1461 | - | 846 | 89 | 89 | 88 | 89 | 90 | 87 | 92 | 91 | 92 | 91 | 91 | 90 | 94 | 96 | 96 | 96 | 100 | 94 | 95 | 95 | 95 | 95 | 100 | 100 | 100 | 95 | 94 |
| Rv1462 | - | 397 | 70 | 72 | 70 | 71 | 65 | 64 | 68 | 66 | 81 | 81 | 83 | 84 | 84 | 91 | 90 | 91 | 100 | 86 | 86 | 87 | 87 | 85 | 100 | 100 | 100 | 90 | 80 |
| Rv1463 | - | 266 | 80 | 83 | 82 | 80 | 83 | 82 | 83 | 84 | 90 | 91 | 89 | 90 | 90 | 95 | 95 | 95 | 100 | 90 | 88 | 88 | 88 | 91 | 100 | 100 | 100 | 94 | 90 |
| Rv1464 | *csd* | 417 | 77 | 74 | 77 | 76 | 77 | 76 | 78 | 69 | 82 | 84 | 82 | 81 | 84 | 92 | 93 | 92 | 100 | 87 | 91 | 91 | 91 | 90 | 100 | 100 | 100 | 92 | 87 |
| Rv1465 | - | 162 | 75 | 81 | 82 | 83 | 80 | 78 | 77 | 72 | 89 | 88 | 85 | 88 | 82 | 87 | 83 | 87 | 100 | 89 | 82 | 88 | 88 | 90 | 100 | 100 | 100 | 83 | 88 |
| Rv1466 | - | 115 | 76 | 82 | 78 | 83 | 71 | 79 | 73 | 82 | 85 | 82 | 86 | 86 | 90 | 99 | 97 | 99 | 100 | 94 | 93 | 93 | 93 | 94 | 100 | 100 | 100 | 97 | 96 |
| Rv1467c | *fadE15* | 609 | 0 | 0 | 0 | 0 | 45 | 43 | 46 | 69 | 77 | 80 | 78 | 78 | 75 | 91 | 95 | 91 | 100 | 75 | 77 | 77 | 77 | 77 | 100 | 100 | 100 | 95 | 75 |
| Rv1468c | *PE_PGRS29* | 370 | 43 | 42 | 36 | 35 | 37 | 0 | 38 | 37 | 42 | 44 | 41 | 39 | 43 | 55 | 75 | 56 | 100 | 52 | 52 | 49 | 49 | 52 | 100 | 100 | 100 | 65 | 53 |
| Rv1469 | *ctpD* | 657 | 52 | 49 | 55 | 55 | 54 | 65 | 54 | 54 | 71 | 49 | 50 | 50 | 54 | 50 | 88 | 49 | 100 | 55 | 55 | 56 | 56 | 75 | 100 | 100 | 100 | 88 | 73 |
| Rv1470 | *trxA* | 124 | 61 | 62 | 59 | 58 | 60 | 58 | 62 | 66 | 68 | 67 | 68 | 68 | 67 | 68 | 83 | 68 | 100 | 69 | 72 | 72 | 72 | 65 | 100 | 100 | 100 | 83 | 70 |
| Rv1471 | *trxB1* | 123 | 71 | 71 | 72 | 66 | 68 | 64 | 69 | 78 | 74 | 81 | 84 | 84 | 82 | 89 | 90 | 89 | 100 | 85 | 89 | 89 | 89 | 85 | 100 | 100 | 100 | 89 | 88 |
| Rv1472 | *echA12* | 285 | 53 | 44 | 52 | 0 | 53 | 53 | 53 | 63 | 51 | 60 | 86 | 61 | 81 | 94 | 94 | 94 | 99 | 82 | 90 | 90 | 90 | 89 | 100 | 100 | 100 | 94 | 89 |
| Rv1473 | - | 542 | 81 | 82 | 81 | 81 | 81 | 82 | 81 | 77 | 89 | 88 | 89 | 89 | 92 | 95 | 96 | 95 | 100 | 92 | 94 | 94 | 94 | 94 | 100 | 100 | 100 | 96 | 94 |
| Rv1473A | - | 63 | 0 | 0 | 0 | 0 | 0 | 0 | 0 | 0 | 70 | 0 | 0 | 0 | 73 | 86 | 92 | 86 | 98 | 81 | 86 | 86 | 86 | 81 | 100 | 100 | 100 | 92 | 85 |

| | | | | | | | | | | | | | | | | | | | | | | | | | | | | | |
|---|---|---|---|---|---|---|---|---|---|---|---|---|---|---|---|---|---|---|---|---|---|---|---|---|---|---|---|---|---|
| Rv1474c | - | 187 | 64 | 71 | 71 | 71 | 64 | 62 | 64 | 0 | 85 | 81 | 82 | 82 | 87 | 95 | 96 | 95 | 100 | 93 | 90 | 90 | 90 | 91 | 100 | 100 | 100 | 96 | 93 |
| Rv1475c | *acn* | 943 | 81 | 81 | 82 | 82 | 84 | 82 | 83 | 81 | 88 | 87 | 88 | 87 | 88 | 93 | 94 | 93 | 100 | 90 | 91 | 90 | 90 | 90 | 100 | 100 | 100 | 94 | 88 |
| Rv1476 | - | 186 | 0 | 0 | 0 | 0 | 0 | 0 | 0 | 0 | 56 | 60 | 61 | 61 | 71 | 86 | 86 | 86 | 100 | 63 | 74 | 74 | 74 | 66 | 100 | 100 | 100 | 85 | 65 |
| Rv1477 | - | 472 | 63 | 47 | 46 | 45 | 43 | 63 | 45 | 48 | 51 | 51 | 52 | 52 | 67 | 86 | 88 | 86 | 100 | 70 | 73 | 73 | 73 | 70 | 100 | 100 | 100 | 89 | 71 |
| Rv1478 | - | 241 | 60 | 62 | 64 | 52 | 53 | 58 | 62 | 51 | 59 | 63 | 66 | 55 | 69 | 85 | 90 | 85 | 100 | 75 | 77 | 76 | 76 | 77 | 100 | 100 | 100 | 90 | 76 |
| Rv1479 | *moxR1* | 377 | 0 | 0 | 0 | 0 | 0 | 0 | 0 | 61 | 88 | 84 | 85 | 84 | 88 | 93 | 95 | 93 | 100 | 89 | 86 | 86 | 86 | 87 | 100 | 100 | 100 | 95 | 89 |
| Rv1480 | - | 317 | 0 | 0 | 0 | 0 | 0 | 0 | 0 | 0 | 87 | 83 | 85 | 85 | 90 | 93 | 93 | 93 | 100 | 89 | 89 | 89 | 89 | 89 | 100 | 100 | 100 | 93 | 89 |
| Rv1481 | - | 335 | 0 | 0 | 0 | 0 | 0 | 0 | 0 | 0 | 73 | 78 | 79 | 79 | 84 | 96 | 98 | 96 | 100 | 93 | 91 | 91 | 91 | 95 | 99 | 100 | 100 | 97 | 93 |
| Rv1482c | - | 280 | 0 | 0 | 45 | 0 | 42 | 0 | 0 | 44 | 0 | 49 | 58 | 59 | 71 | 82 | 67 | 84 | 100 | 69 | 73 | 74 | 74 | 73 | 100 | 100 | 100 | 66 | 62 |
| Rv1483 | *fabG1* | 247 | 50 | 45 | 53 | 51 | 51 | 48 | 50 | 71 | 83 | 84 | 84 | 84 | 85 | 93 | 96 | 93 | 100 | 91 | 93 | 93 | 93 | 90 | 100 | 100 | 100 | 95 | 94 |
| Rv1484 | *inhA* | 269 | 0 | 0 | 0 | 0 | 0 | 0 | 0 | 67 | 73 | 76 | 77 | 77 | 95 | 93 | 97 | 93 | 100 | 94 | 92 | 92 | 92 | 94 | 100 | 100 | 100 | 97 | 94 |
| Rv1485 | *hemH* | 344 | 65 | 60 | 63 | 60 | 53 | 64 | 53 | 59 | 69 | 69 | 70 | 71 | 76 | 85 | 84 | 84 | 100 | 78 | 80 | 81 | 81 | 78 | 100 | 100 | 100 | 87 | 78 |
| Rv1486c | - | 288 | 37 | 0 | 0 | 0 | 0 | 0 | 41 | 0 | 54 | 52 | 57 | 56 | 65 | 83 | 83 | 83 | 99 | 71 | 76 | 76 | 76 | 75 | 99 | 100 | 99 | 81 | 73 |
| Rv1487 | - | 144 | 0 | 55 | 64 | 62 | 57 | 60 | 58 | 56 | 65 | 69 | 71 | 71 | 70 | 84 | 89 | 85 | 100 | 83 | 82 | 82 | 82 | 81 | 100 | 100 | 100 | 88 | 83 |
| Rv1488 | - | 381 | 44 | 80 | 80 | 80 | 78 | 77 | 81 | 70 | 85 | 87 | 88 | 88 | 89 | 97 | 96 | 97 | 100 | 91 | 92 | 92 | 92 | 91 | 100 | 100 | 100 | 95 | 92 |
| Rv1489 | - | 118 | 0 | 0 | 0 | 0 | 0 | 0 | 0 | 0 | 0 | 0 | 0 | 0 | 71 | 83 | 89 | 83 | 100 | 72 | 72 | 71 | 71 | 70 | 100 | 100 | 100 | 86 | 76 |
| Rv1489A | - | 76 | 0 | 0 | 0 | 0 | 0 | 0 | 0 | 0 | 74 | 69 | 67 | 73 | 81 | 88 | 86 | 88 | 100 | 78 | 80 | 80 | 80 | 80 | 100 | 100 | 100 | 86 | 80 |
| Rv1490 | - | 435 | 0 | 0 | 0 | 0 | 0 | 0 | 0 | 0 | 0 | 0 | 0 | 0 | 0 | 0 | 0 | 59 | 100 | 0 | 56 | 0 | 0 | 0 | 100 | 100 | 100 | 0 | 0 |
| Rv1491c | - | 252 | 58 | 59 | 60 | 60 | 57 | 64 | 53 | 55 | 61 | 68 | 68 | 63 | 74 | 76 | 80 | 76 | 100 | 70 | 72 | 72 | 72 | 74 | 100 | 100 | 100 | 80 | 73 |
| Rv1492 | *mutA* | 615 | 0 | 57 | 59 | 58 | 0 | 59 | 0 | 50 | 64 | 66 | 66 | 67 | 71 | 83 | 84 | 84 | 100 | 76 | 79 | 79 | 79 | 76 | 100 | 100 | 100 | 84 | 79 |
| Rv1493 | *mutB* | 750 | 0 | 85 | 84 | 86 | 0 | 81 | 0 | 81 | 89 | 87 | 90 | 90 | 92 | 94 | 94 | 94 | 100 | 92 | 92 | 92 | 92 | 93 | 100 | 100 | 100 | 93 | 93 |
| Rv1494 | - | 100 | 0 | 0 | 0 | 0 | 0 | 0 | 0 | 0 | 0 | 0 | 0 | 0 | 0 | 0 | 0 | 0 | 100 | 0 | 0 | 0 | 0 | 0 | 99 | 100 | 100 | 0 | 0 |
| Rv1495 | - | 105 | 0 | 0 | 0 | 0 | 0 | 0 | 0 | 0 | 0 | 0 | 0 | 0 | 0 | 0 | 0 | 0 | 100 | 0 | 0 | 0 | 0 | 0 | 100 | 100 | 100 | 0 | 0 |
| Rv1496 | - | 334 | 0 | 76 | 74 | 78 | 0 | 78 | 0 | 75 | 84 | 85 | 83 | 85 | 87 | 90 | 92 | 90 | 100 | 90 | 89 | 89 | 89 | 90 | 100 | 100 | 100 | 92 | 90 |
| Rv1497 | *lipL* | 429 | 0 | 0 | 0 | 0 | 0 | 0 | 0 | 0 | 59 | 55 | 56 | 56 | 62 | 85 | 85 | 85 | 100 | 76 | 74 | 74 | 74 | 74 | 100 | 100 | 100 | 88 | 76 |
| Rv1498A | - | 70 | 0 | 0 | 0 | 0 | 0 | 0 | 0 | 81 | 0 | 80 | 79 | 80 | 0 | 0 | 94 | 0 | 100 | 0 | 0 | 0 | 0 | 0 | 100 | 100 | 100 | 94 | 0 |
| Rv1498c | - | 205 | 0 | 0 | 0 | 0 | 0 | 0 | 0 | 0 | 0 | 0 | 0 | 0 | 0 | 0 | 0 | 0 | 99 | 0 | 0 | 0 | 0 | 0 | 99 | 100 | 99 | 0 | 0 |
| Rv1499 | - | 132 | 0 | 0 | 0 | 0 | 0 | 0 | 0 | 0 | 0 | 0 | 0 | 0 | 0 | 0 | 0 | 0 | 100 | 0 | 0 | 0 | 0 | 0 | 100 | 100 | 100 | 0 | 0 |
| Rv1500 | - | 342 | 0 | 0 | 0 | 0 | 0 | 0 | 0 | 0 | 43 | 45 | 0 | 0 | 0 | 0 | 87 | 0 | 100 | 0 | 0 | 0 | 0 | 0 | 100 | 100 | 100 | 90 | 0 |
| Rv1501 | - | 273 | 0 | 0 | 0 | 0 | 0 | 0 | 0 | 0 | 42 | 0 | 0 | 0 | 0 | 51 | 51 | 51 | 100 | 0 | 0 | 0 | 0 | 0 | 100 | 100 | 100 | 48 | 0 |
| Rv1502 | - | 299 | 0 | 0 | 0 | 0 | 0 | 0 | 0 | 0 | 0 | 0 | 0 | 0 | 0 | 0 | 83 | 0 | 98 | 0 | 0 | 0 | 0 | 0 | 99 | 100 | 99 | 0 | 0 |
| Rv1503c | - | 182 | 0 | 0 | 0 | 0 | 0 | 0 | 0 | 0 | 61 | 59 | 0 | 0 | 0 | 0 | 0 | 0 | 100 | 0 | 0 | 0 | 0 | 0 | 100 | 100 | 100 | 0 | 0 |
| Rv1504c | - | 199 | 0 | 0 | 0 | 47 | 0 | 0 | 0 | 49 | 65 | 66 | 53 | 53 | 53 | 0 | 92 | 0 | 100 | 0 | 0 | 0 | 0 | 0 | 100 | 100 | 100 | 0 | 0 |
| Rv1505c | - | 221 | 0 | 0 | 0 | 0 | 0 | 0 | 0 | 0 | 0 | 0 | 0 | 0 | 0 | 0 | 94 | 0 | 100 | 0 | 0 | 0 | 0 | 0 | 99 | 100 | 100 | 0 | 0 |
| Rv1506c | - | 166 | 0 | 0 | 0 | 0 | 0 | 0 | 0 | 0 | 0 | 0 | 0 | 0 | 0 | 0 | 0 | 0 | 0 | 0 | 0 | 0 | 0 | 0 | 100 | 100 | 100 | 0 | 0 |
| Rv1507A | - | 167 | 0 | 0 | 0 | 0 | 0 | 0 | 0 | 0 | 0 | 0 | 0 | 0 | 0 | 0 | 0 | 0 | 0 | 0 | 0 | 0 | 0 | 0 | 100 | 100 | 100 | 0 | 0 |
| Rv1507c | - | 231 | 0 | 0 | 0 | 0 | 0 | 0 | 0 | 0 | 0 | 0 | 0 | 0 | 0 | 0 | 93 | 0 | 0 | 0 | 0 | 0 | 0 | 0 | 100 | 100 | 100 | 0 | 0 |
| Rv1508A | - | 120 | 0 | 0 | 0 | 0 | 0 | 0 | 0 | 0 | 0 | 80 | 80 | 0 | 0 | 80 | 0 | 0 | 0 | 80 | 0 | 80 | 80 | 0 | 100 | 100 | 100 | 0 | 0 |

| | | | | | | | | | | | | | | | | | | | | | | | | | | | | | |
|---|---|---|---|---|---|---|---|---|---|---|---|---|---|---|---|---|---|---|---|---|---|---|---|---|---|---|---|---|---|
| Rv1508c | - | 599 | 0 | 0 | 0 | 0 | 0 | 0 | 0 | 0 | 0 | 0 | 0 | 0 | 0 | 0 | 69 | 0 | 0 | 0 | 0 | 0 | 0 | 0 | 100 | 100 | 100 | 0 | 0 |
| Rv1509 | - | 293 | 0 | 0 | 0 | 0 | 0 | 0 | 0 | 0 | 0 | 0 | 0 | 0 | 0 | 0 | 0 | 0 | 0 | 0 | 0 | 0 | 0 | 0 | 100 | 100 | 100 | 0 | 0 |
| Rv1510 | - | 432 | 0 | 0 | 53 | 52 | 50 | 0 | 50 | 0 | 0 | 38 | 0 | 0 | 69 | 82 | 82 | 82 | 81 | 75 | 80 | 80 | 80 | 78 | 81 | 100 | 100 | 82 | 77 |
| Rv1511 | *gmdA* | 340 | 42 | 46 | 46 | 44 | 0 | 42 | 39 | 81 | 42 | 90 | 91 | 46 | 42 | 95 | 43 | 47 | 43 | 88 | 46 | 87 | 87 | 43 | 100 | 100 | 100 | 43 | 46 |
| Rv1512 | *epiA* | 322 | 0 | 0 | 44 | 43 | 0 | 40 | 0 | 38 | 41 | 82 | 81 | 41 | 43 | 91 | 42 | 44 | 45 | 83 | 0 | 80 | 80 | 44 | 100 | 100 | 100 | 42 | 0 |
| Rv1513 | - | 243 | 0 | 0 | 0 | 0 | 0 | 0 | 0 | 0 | 0 | 0 | 0 | 0 | 0 | 76 | 0 | 0 | 78 | 0 | 0 | 44 | 44 | 0 | 100 | 100 | 100 | 0 | 44 |
| Rv1514c | - | 262 | 0 | 0 | 0 | 0 | 0 | 0 | 0 | 0 | 0 | 0 | 0 | 0 | 0 | 61 | 0 | 0 | 47 | 50 | 0 | 0 | 0 | 0 | 100 | 100 | 100 | 0 | 0 |
| Rv1515c | - | 298 | 0 | 0 | 0 | 0 | 0 | 0 | 0 | 0 | 0 | 0 | 0 | 0 | 0 | 0 | 0 | 0 | 0 | 0 | 0 | 0 | 0 | 0 | 100 | 100 | 100 | 0 | 0 |
| Rv1516c | - | 336 | 0 | 0 | 0 | 49 | 0 | 0 | 0 | 0 | 0 | 0 | 0 | 0 | 0 | 0 | 0 | 73 | 76 | 0 | 0 | 0 | 0 | 0 | 100 | 100 | 100 | 0 | 0 |
| Rv1517 | - | 254 | 0 | 0 | 0 | 0 | 0 | 0 | 0 | 0 | 0 | 0 | 0 | 0 | 50 | 52 | 62 | 50 | 99 | 50 | 50 | 50 | 50 | 55 | 99 | 100 | 100 | 61 | 49 |
| Rv1518 | - | 319 | 0 | 0 | 0 | 0 | 0 | 0 | 0 | 0 | 0 | 45 | 53 | 0 | 0 | 0 | 0 | 0 | 99 | 0 | 0 | 0 | 0 | 0 | 100 | 100 | 100 | 0 | 47 |
| Rv1519 | - | 89 | 0 | 0 | 0 | 0 | 0 | 0 | 0 | 0 | 0 | 0 | 0 | 0 | 0 | 0 | 0 | 0 | 100 | 0 | 0 | 0 | 0 | 0 | 100 | 100 | 100 | 0 | 0 |
| Rv1520 | - | 346 | 0 | 0 | 0 | 0 | 0 | 0 | 0 | 0 | 0 | 0 | 0 | 0 | 0 | 0 | 0 | 69 | 100 | 0 | 0 | 0 | 0 | 0 | 100 | 100 | 100 | 0 | 0 |
| Rv1521 | *fadD25* | 583 | 50 | 54 | 52 | 52 | 53 | 36 | 51 | 38 | 55 | 54 | 54 | 54 | 72 | 77 | 83 | 74 | 99 | 72 | 74 | 74 | 74 | 72 | 100 | 100 | 100 | 78 | 70 |
| Rv1522c | *mmpL12* | 1146 | 44 | 45 | 56 | 56 | 43 | 62 | 41 | 52 | 52 | 54 | 46 | 47 | 67 | 65 | 69 | 69 | 99 | 68 | 67 | 70 | 70 | 66 | 100 | 100 | 100 | 49 | 68 |
| Rv1523 | - | 347 | 0 | 0 | 54 | 49 | 0 | 0 | 0 | 45 | 52 | 0 | 45 | 0 | 70 | 79 | 63 | 0 | 100 | 49 | 53 | 51 | 51 | 67 | 100 | 100 | 100 | 65 | 53 |
| Rv1524 | - | 414 | 0 | 0 | 0 | 0 | 0 | 43 | 0 | 0 | 0 | 0 | 40 | 0 | 72 | 70 | 74 | 79 | 100 | 0 | 67 | 74 | 74 | 73 | 100 | 100 | 100 | 66 | 0 |
| Rv1525 | *wbbL2* | 261 | 0 | 0 | 0 | 0 | 0 | 0 | 0 | 0 | 0 | 0 | 0 | 0 | 0 | 0 | 79 | 0 | 100 | 0 | 0 | 0 | 0 | 0 | 100 | 100 | 100 | 78 | 0 |
| Rv1526c | - | 426 | 0 | 0 | 0 | 0 | 0 | 44 | 0 | 0 | 0 | 0 | 42 | 0 | 66 | 67 | 70 | 70 | 100 | 0 | 64 | 68 | 68 | 68 | 100 | 100 | 100 | 61 | 0 |
| Rv1527c | *pks5* | 2108 | 49 | 51 | 52 | 52 | 50 | 43 | 48 | 44 | 46 | 52 | 46 | 45 | 76 | 79 | 83 | 80 | 99 | 52 | 79 | 80 | 80 | 77 | 79 | 100 | 100 | 77 | 48 |
| Rv1528c | *papA4* | 165 | 0 | 0 | 0 | 0 | 0 | 0 | 0 | 0 | 0 | 0 | 0 | 0 | 0 | 61 | 69 | 0 | 100 | 0 | 59 | 59 | 59 | 0 | 100 | 100 | 100 | 64 | 0 |
| Rv1529 | *fadD24* | 584 | 51 | 54 | 51 | 50 | 52 | 37 | 51 | 42 | 53 | 54 | 55 | 54 | 69 | 77 | 73 | 73 | 100 | 69 | 73 | 73 | 73 | 71 | 100 | 100 | 100 | 77 | 70 |
| Rv1530 | *adh* | 367 | 42 | 44 | 41 | 42 | 42 | 42 | 43 | 42 | 45 | 44 | 42 | 41 | 74 | 45 | 72 | 45 | 100 | 73 | 74 | 74 | 74 | 42 | 100 | 100 | 100 | 45 | 46 |
| Rv1531 | - | 188 | 0 | 0 | 0 | 0 | 0 | 0 | 0 | 54 | 0 | 0 | 0 | 0 | 72 | 85 | 90 | 85 | 100 | 72 | 72 | 72 | 72 | 47 | 100 | 100 | 100 | 49 | 51 |
| Rv1532c | - | 144 | 0 | 0 | 0 | 0 | 0 | 0 | 0 | 0 | 50 | 0 | 0 | 0 | 51 | 53 | 53 | 53 | 100 | 0 | 0 | 0 | 0 | 47 | 100 | 100 | 100 | 51 | 0 |
| Rv1533 | - | 375 | 42 | 0 | 39 | 0 | 42 | 40 | 44 | 66 | 64 | 64 | 64 | 64 | 64 | 91 | 91 | 92 | 100 | 66 | 65 | 65 | 65 | 66 | 100 | 100 | 100 | 63 | 67 |
| Rv1534 | - | 225 | 0 | 0 | 0 | 0 | 0 | 0 | 55 | 0 | 47 | 44 | 0 | 43 | 60 | 47 | 46 | 47 | 100 | 44 | 45 | 45 | 45 | 45 | 100 | 100 | 100 | 46 | 44 |
| Rv1535 | - | 78 | 0 | 0 | 0 | 0 | 0 | 0 | 0 | 0 | 0 | 0 | 0 | 0 | 0 | 74 | 77 | 75 | 100 | 0 | 0 | 0 | 0 | 0 | 100 | 100 | 100 | 77 | 0 |
| Rv1536 | *ileS* | 1041 | 73 | 76 | 75 | 75 | 72 | 75 | 73 | 73 | 78 | 80 | 78 | 79 | 85 | 92 | 91 | 92 | 99 | 88 | 89 | 89 | 89 | 88 | 100 | 100 | 100 | 91 | 87 |
| Rv1537 | *dinX* | 468 | 64 | 67 | 66 | 67 | 63 | 65 | 59 | 57 | 77 | 73 | 75 | 75 | 77 | 87 | 88 | 87 | 100 | 81 | 85 | 85 | 85 | 84 | 100 | 100 | 99 | 87 | 83 |
| Rv1538c | *ansA* | 326 | 50 | 52 | 52 | 52 | 41 | 52 | 46 | 0 | 0 | 56 | 55 | 56 | 62 | 78 | 81 | 78 | 100 | 71 | 72 | 73 | 73 | 70 | 100 | 100 | 100 | 81 | 72 |
| Rv1539 | *lspA* | 202 | 61 | 62 | 56 | 61 | 56 | 60 | 64 | 59 | 73 | 78 | 73 | 72 | 89 | 87 | 87 | 87 | 100 | 85 | 85 | 85 | 85 | 86 | 100 | 100 | 100 | 91 | 89 |
| Rv1540 | - | 308 | 72 | 79 | 81 | 82 | 78 | 77 | 76 | 75 | 85 | 87 | 86 | 86 | 87 | 95 | 94 | 95 | 100 | 92 | 93 | 93 | 93 | 92 | 100 | 100 | 100 | 93 | 91 |
| Rv1541c | *lprI* | 197 | 0 | 0 | 0 | 0 | 0 | 0 | 0 | 0 | 0 | 0 | 0 | 0 | 0 | 0 | 72 | 0 | 100 | 0 | 0 | 0 | 0 | 0 | 100 | 100 | 100 | 72 | 0 |
| Rv1542c | *glbN* | 136 | 0 | 0 | 0 | 0 | 0 | 0 | 0 | 0 | 0 | 0 | 0 | 0 | 0 | 92 | 92 | 92 | 100 | 57 | 87 | 87 | 87 | 86 | 100 | 100 | 100 | 90 | 62 |
| Rv1543 | - | 341 | 52 | 46 | 51 | 49 | 48 | 48 | 49 | 66 | 61 | 56 | 60 | 60 | 63 | 89 | 93 | 91 | 100 | 65 | 59 | 59 | 59 | 62 | 100 | 100 | 100 | 89 | 64 |
| Rv1544 | - | 267 | 52 | 55 | 56 | 57 | 61 | 47 | 51 | 53 | 60 | 62 | 62 | 63 | 58 | 90 | 93 | 90 | 100 | 60 | 55 | 58 | 58 | 55 | 100 | 100 | 100 | 93 | 76 |
| Rv1545 | - | 75 | 0 | 0 | 0 | 0 | 0 | 0 | 0 | 0 | 0 | 0 | 0 | 0 | 0 | 0 | 0 | 0 | 100 | 0 | 0 | 0 | 0 | 0 | 100 | 100 | 100 | 0 | 0 |

| | | | | | | | | | | | | | | | | | | | | | | | | | | | | | |
|---|---|---|---|---|---|---|---|---|---|---|---|---|---|---|---|---|---|---|---|---|---|---|---|---|---|---|---|---|---|
| Rv1546 | - | 143 | 0 | 0 | 0 | 0 | 0 | 0 | 0 | 0 | 72 | 69 | 65 | 65 | 60 | 88 | 88 | 88 | 100 | 73 | 74 | 74 | 74 | 61 | 100 | 100 | 100 | 88 | 71 |
| Rv1547 | *dnaE* | 1184 | 78 | 78 | 79 | 79 | 78 | 77 | 78 | 70 | 87 | 86 | 88 | 88 | 90 | 95 | 95 | 94 | 100 | 91 | 91 | 91 | 91 | 92 | 100 | 100 | 100 | 94 | 92 |
| Rv1548c | *PPE21* | 678 | 0 | 0 | 0 | 0 | 0 | 0 | 0 | 0 | 44 | 38 | 36 | 34 | 51 | 71 | 71 | 71 | 99 | 34 | 47 | 46 | 46 | 52 | 99 | 100 | 99 | 60 | 53 |
| Rv1549 | *fadD11.1* | 175 | 0 | 0 | 0 | 0 | 0 | 0 | 0 | 51 | 75 | 78 | 69 | 67 | 71 | 90 | 95 | 90 | 100 | 49 | 45 | 45 | 45 | 52 | 100 | 100 | 100 | 95 | 52 |
| Rv1550 | *fadD11* | 571 | 54 | 53 | 53 | 55 | 56 | 52 | 54 | 61 | 67 | 72 | 74 | 64 | 63 | 88 | 91 | 88 | 99 | 56 | 55 | 55 | 55 | 55 | 100 | 100 | 100 | 90 | 55 |
| Rv1551 | *plsB1* | 621 | 0 | 0 | 0 | 0 | 0 | 0 | 0 | 0 | 54 | 58 | 59 | 59 | 0 | 91 | 93 | 91 | 100 | 51 | 51 | 51 | 51 | 51 | 100 | 100 | 100 | 92 | 51 |
| Rv1552 | *frdA* | 583 | 45 | 46 | 43 | 44 | 45 | 45 | 45 | 53 | 61 | 61 | 61 | 61 | 62 | 62 | 62 | 62 | 100 | 62 | 61 | 61 | 61 | 61 | 100 | 100 | 100 | 61 | 61 |
| Rv1553 | *frdB* | 247 | 44 | 44 | 44 | 44 | 41 | 47 | 44 | 50 | 56 | 54 | 55 | 55 | 55 | 56 | 55 | 56 | 100 | 54 | 56 | 56 | 56 | 56 | 100 | 100 | 100 | 56 | 55 |
| Rv1554 | *frdC* | 126 | 0 | 0 | 0 | 0 | 0 | 0 | 0 | 0 | 0 | 0 | 0 | 0 | 0 | 0 | 0 | 0 | 100 | 0 | 0 | 0 | 0 | 0 | 100 | 100 | 100 | 0 | 0 |
| Rv1555 | *frdD* | 125 | 0 | 0 | 0 | 0 | 0 | 0 | 0 | 0 | 0 | 0 | 0 | 0 | 0 | 0 | 0 | 0 | 100 | 0 | 0 | 0 | 0 | 0 | 100 | 100 | 100 | 0 | 0 |
| Rv1556 | - | 202 | 0 | 0 | 0 | 0 | 0 | 0 | 0 | 57 | 48 | 49 | 45 | 46 | 78 | 87 | 87 | 87 | 100 | 77 | 52 | 54 | 54 | 49 | 100 | 100 | 100 | 88 | 50 |
| Rv1557 | *mmpL6* | 397 | 42 | 50 | 56 | 57 | 42 | 56 | 45 | 59 | 52 | 48 | 49 | 50 | 83 | 86 | 88 | 86 | 99 | 80 | 79 | 79 | 79 | 83 | 100 | 100 | 99 | 76 | 82 |
| Rv1558 | - | 148 | 0 | 0 | 0 | 0 | 0 | 0 | 0 | 64 | 86 | 79 | 79 | 80 | 82 | 90 | 93 | 90 | 100 | 71 | 87 | 87 | 87 | 69 | 100 | 100 | 100 | 93 | 70 |
| Rv1559 | *ilvA* | 429 | 74 | 74 | 73 | 72 | 55 | 53 | 51 | 53 | 77 | 75 | 76 | 76 | 83 | 93 | 94 | 93 | 100 | 90 | 92 | 92 | 92 | 88 | 100 | 100 | 100 | 94 | 90 |
| Rv1560 | - | 72 | 0 | 0 | 0 | 0 | 0 | 0 | 0 | 0 | 0 | 0 | 0 | 0 | 0 | 0 | 0 | 0 | 100 | 0 | 0 | 0 | 0 | 0 | 100 | 100 | 100 | 0 | 0 |
| Rv1561 | - | 134 | 0 | 0 | 0 | 0 | 0 | 0 | 0 | 0 | 0 | 0 | 0 | 0 | 0 | 0 | 0 | 0 | 100 | 0 | 0 | 0 | 0 | 0 | 100 | 100 | 100 | 0 | 0 |
| Rv1562c | *treZ* | 580 | 41 | 45 | 62 | 62 | 60 | 59 | 0 | 63 | 76 | 73 | 75 | 74 | 39 | 85 | 84 | 86 | 99 | 84 | 80 | 81 | 81 | 81 | 100 | 100 | 100 | 84 | 84 |
| Rv1563c | *treY* | 765 | 0 | 0 | 60 | 60 | 59 | 56 | 0 | 58 | 65 | 63 | 64 | 65 | 0 | 85 | 85 | 85 | 100 | 79 | 76 | 76 | 76 | 72 | 100 | 100 | 100 | 85 | 77 |
| Rv1564c | *treX* | 721 | 79 | 79 | 80 | 81 | 76 | 77 | 0 | 74 | 83 | 80 | 81 | 81 | 85 | 89 | 91 | 89 | 100 | 89 | 88 | 88 | 88 | 89 | 100 | 100 | 99 | 91 | 88 |
| Rv1565c | - | 729 | 49 | 52 | 51 | 52 | 50 | 41 | 48 | 0 | 60 | 60 | 61 | 60 | 73 | 83 | 88 | 83 | 100 | 81 | 82 | 82 | 82 | 85 | 100 | 100 | 100 | 87 | 82 |
| Rv1566c | - | 230 | 61 | 62 | 64 | 65 | 57 | 60 | 55 | 0 | 61 | 62 | 65 | 65 | 62 | 83 | 86 | 80 | 100 | 59 | 57 | 56 | 56 | 57 | 100 | 100 | 100 | 86 | 59 |
| Rv1567c | - | 94 | 0 | 0 | 0 | 0 | 0 | 0 | 0 | 0 | 0 | 0 | 0 | 0 | 0 | 78 | 87 | 78 | 100 | 0 | 0 | 0 | 0 | 0 | 100 | 100 | 100 | 87 | 0 |
| Rv1568 | *bioA* | 437 | 45 | 66 | 69 | 68 | 44 | 62 | 45 | 69 | 75 | 75 | 77 | 77 | 80 | 87 | 89 | 88 | 100 | 81 | 82 | 82 | 82 | 82 | 100 | 100 | 100 | 89 | 82 |
| Rv1569 | *bioF1* | 386 | 0 | 56 | 0 | 0 | 0 | 50 | 0 | 62 | 77 | 79 | 79 | 80 | 81 | 90 | 90 | 90 | 100 | 85 | 83 | 83 | 83 | 85 | 100 | 100 | 99 | 89 | 84 |
| Rv1570 | *bioD* | 226 | 0 | 54 | 57 | 52 | 0 | 56 | 0 | 51 | 59 | 62 | 65 | 65 | 68 | 81 | 82 | 82 | 99 | 73 | 75 | 73 | 73 | 72 | 99 | 100 | 99 | 82 | 75 |
| Rv1571 | - | 169 | 0 | 0 | 0 | 0 | 0 | 0 | 0 | 0 | 0 | 57 | 57 | 57 | 0 | 83 | 83 | 83 | 100 | 66 | 67 | 67 | 67 | 73 | 100 | 100 | 100 | 85 | 63 |
| Rv1572c | - | 34 | 0 | 0 | 0 | 0 | 0 | 0 | 0 | 0 | 0 | 0 | 0 | 0 | 0 | 0 | 0 | 0 | 100 | 0 | 0 | 0 | 0 | 0 | 100 | 100 | 96 | 0 | 0 |
| Rv1573 | - | 136 | 0 | 0 | 0 | 0 | 0 | 0 | 0 | 0 | 0 | 0 | 0 | 0 | 0 | 0 | 0 | 0 | 100 | 0 | 0 | 0 | 0 | 0 | 100 | 100 | 0 | 0 | 0 |
| Rv1574 | - | 103 | 0 | 0 | 0 | 0 | 0 | 0 | 0 | 0 | 0 | 0 | 0 | 0 | 0 | 0 | 0 | 0 | 100 | 0 | 0 | 0 | 0 | 0 | 100 | 100 | 0 | 0 | 0 |
| Rv1575 | - | 166 | 0 | 0 | 0 | 0 | 0 | 0 | 0 | 0 | 0 | 0 | 0 | 0 | 0 | 0 | 0 | 0 | 78 | 0 | 0 | 0 | 0 | 0 | 98 | 98 | 75 | 0 | 0 |
| Rv1576c | - | 473 | 0 | 0 | 0 | 0 | 0 | 0 | 0 | 0 | 0 | 60 | 0 | 0 | 0 | 0 | 0 | 0 | 99 | 0 | 0 | 0 | 0 | 0 | 94 | 100 | 94 | 0 | 58 |
| Rv1577c | - | 170 | 0 | 48 | 0 | 0 | 0 | 0 | 0 | 0 | 0 | 44 | 0 | 51 | 0 | 0 | 0 | 0 | 100 | 0 | 0 | 0 | 0 | 0 | 100 | 100 | 94 | 0 | 0 |
| Rv1578c | - | 156 | 0 | 0 | 0 | 0 | 0 | 0 | 0 | 0 | 62 | 0 | 0 | 0 | 0 | 0 | 0 | 0 | 100 | 0 | 0 | 0 | 0 | 0 | 100 | 100 | 58 | 0 | 0 |
| Rv1579c | - | 104 | 0 | 0 | 0 | 0 | 0 | 0 | 0 | 0 | 0 | 0 | 0 | 0 | 0 | 0 | 0 | 0 | 100 | 0 | 0 | 0 | 0 | 0 | 100 | 100 | 0 | 0 | 0 |
| Rv1580c | - | 90 | 0 | 0 | 0 | 0 | 0 | 0 | 0 | 0 | 0 | 0 | 0 | 0 | 0 | 0 | 0 | 0 | 100 | 0 | 0 | 0 | 0 | 0 | 100 | 100 | 0 | 0 | 0 |
| Rv1581c | - | 131 | 0 | 0 | 0 | 0 | 0 | 0 | 0 | 0 | 0 | 0 | 0 | 0 | 0 | 0 | 0 | 0 | 100 | 0 | 0 | 0 | 0 | 0 | 100 | 100 | 0 | 0 | 0 |
| Rv1582c | - | 471 | 0 | 0 | 0 | 0 | 0 | 0 | 0 | 0 | 0 | 0 | 0 | 0 | 0 | 0 | 0 | 0 | 100 | 0 | 0 | 0 | 0 | 0 | 100 | 100 | 0 | 0 | 0 |
| Rv1583c | - | 132 | 0 | 0 | 0 | 0 | 0 | 0 | 0 | 0 | 0 | 0 | 0 | 0 | 0 | 58 | 0 | 0 | 99 | 0 | 0 | 0 | 0 | 0 | 100 | 100 | 84 | 0 | 60 |

| | | | | | | | | | | | | | | | | | | | | | | | | | | | | | |
|---|---|---|---|---|---|---|---|---|---|---|---|---|---|---|---|---|---|---|---|---|---|---|---|---|---|---|---|---|---|
| Rv1584c | - | 73 | 0 | 0 | 0 | 0 | 0 | 0 | 0 | 0 | 0 | 0 | 0 | 0 | 0 | 0 | 0 | 0 | 100 | 0 | 0 | 70 | 68 | 0 | 100 | 100 | 0 | 0 | 0 |
| Rv1585c | - | 171 | 0 | 0 | 0 | 0 | 0 | 0 | 0 | 0 | 0 | 0 | 0 | 0 | 0 | 0 | 0 | 0 | 100 | 0 | 0 | 0 | 0 | 0 | 100 | 100 | 0 | 0 | 0 |
| Rv1586c | - | 469 | 0 | 0 | 0 | 0 | 0 | 0 | 42 | 50 | 0 | 52 | 0 | 0 | 0 | 0 | 47 | 49 | 100 | 0 | 0 | 48 | 76 | 0 | 100 | 100 | 0 | 0 | 51 |
| Rv1587c | - | 333 | 40 | 51 | 0 | 47 | 48 | 0 | 0 | 0 | 0 | 38 | 44 | 43 | 68 | 58 | 54 | 59 | 98 | 51 | 64 | 63 | 63 | 57 | 99 | 100 | 98 | 72 | 64 |
| Rv1588c | - | 222 | 0 | 0 | 0 | 0 | 0 | 0 | 0 | 0 | 0 | 0 | 0 | 49 | 60 | 68 | 68 | 68 | 98 | 62 | 54 | 54 | 54 | 52 | 98 | 100 | 99 | 75 | 59 |
| Rv1589 | *bioB* | 349 | 83 | 87 | 83 | 86 | 82 | 85 | 83 | 56 | 93 | 92 | 93 | 93 | 92 | 94 | 95 | 94 | 100 | 91 | 94 | 94 | 94 | 94 | 100 | 100 | 100 | 95 | 94 |
| Rv1590 | - | 79 | 79 | 75 | 85 | 79 | 58 | 68 | 70 | 0 | 74 | 71 | 71 | 74 | 76 | 88 | 89 | 89 | 100 | 87 | 82 | 82 | 82 | 88 | 100 | 100 | 100 | 89 | 81 |
| Rv1591 | - | 221 | 0 | 0 | 0 | 0 | 0 | 0 | 0 | 0 | 0 | 44 | 0 | 0 | 61 | 74 | 78 | 74 | 100 | 67 | 67 | 67 | 67 | 67 | 100 | 100 | 99 | 77 | 65 |
| Rv1592c | - | 446 | 37 | 42 | 46 | 43 | 47 | 0 | 41 | 0 | 68 | 52 | 49 | 50 | 75 | 89 | 91 | 89 | 100 | 81 | 82 | 82 | 82 | 81 | 100 | 100 | 100 | 91 | 80 |
| Rv1593c | - | 236 | 0 | 52 | 51 | 52 | 0 | 0 | 40 | 0 | 75 | 78 | 80 | 79 | 82 | 92 | 92 | 92 | 100 | 85 | 87 | 87 | 87 | 88 | 100 | 100 | 100 | 92 | 86 |
| Rv1594 | *nadA* | 349 | 0 | 76 | 45 | 44 | 82 | 0 | 43 | 44 | 88 | 88 | 86 | 87 | 89 | 96 | 96 | 96 | 100 | 89 | 91 | 91 | 91 | 90 | 100 | 100 | 100 | 94 | 93 |
| Rv1595 | *nadB* | 527 | 41 | 55 | 42 | 42 | 40 | 40 | 50 | 59 | 65 | 68 | 68 | 67 | 73 | 85 | 86 | 85 | 100 | 80 | 80 | 80 | 80 | 78 | 100 | 100 | 99 | 86 | 79 |
| Rv1596 | *nadC* | 285 | 0 | 62 | 61 | 57 | 61 | 0 | 58 | 68 | 83 | 83 | 83 | 82 | 83 | 89 | 91 | 89 | 100 | 82 | 87 | 87 | 87 | 86 | 100 | 100 | 100 | 90 | 85 |
| Rv1597 | - | 252 | 0 | 0 | 0 | 0 | 0 | 0 | 0 | 0 | 0 | 75 | 84 | 78 | 0 | 81 | 88 | 80 | 99 | 0 | 0 | 0 | 0 | 0 | 99 | 100 | 99 | 88 | 0 |
| Rv1598c | - | 136 | 0 | 0 | 0 | 0 | 0 | 0 | 0 | 0 | 53 | 0 | 53 | 57 | 67 | 85 | 86 | 86 | 100 | 71 | 70 | 70 | 70 | 76 | 100 | 100 | 100 | 85 | 77 |
| Rv1599 | *hisD* | 438 | 74 | 76 | 75 | 75 | 75 | 76 | 74 | 72 | 82 | 84 | 85 | 85 | 86 | 89 | 92 | 89 | 100 | 88 | 83 | 83 | 83 | 89 | 100 | 100 | 100 | 91 | 88 |
| Rv1600 | *hisC1* | 380 | 75 | 79 | 78 | 80 | 76 | 71 | 77 | 65 | 83 | 85 | 84 | 83 | 84 | 93 | 93 | 93 | 100 | 87 | 88 | 88 | 88 | 88 | 100 | 100 | 100 | 90 | 86 |
| Rv1601 | *hisB* | 210 | 75 | 75 | 75 | 75 | 79 | 81 | 82 | 77 | 83 | 88 | 90 | 89 | 92 | 97 | 97 | 97 | 100 | 92 | 90 | 90 | 90 | 89 | 100 | 100 | 100 | 95 | 91 |
| Rv1602 | *hisH* | 206 | 74 | 75 | 73 | 73 | 65 | 59 | 70 | 75 | 78 | 75 | 76 | 77 | 81 | 88 | 88 | 88 | 100 | 88 | 89 | 89 | 89 | 86 | 100 | 100 | 100 | 87 | 88 |
| Rv1603 | *hisA* | 245 | 80 | 79 | 79 | 79 | 83 | 83 | 82 | 76 | 89 | 87 | 89 | 89 | 89 | 96 | 96 | 96 | 100 | 94 | 95 | 95 | 95 | 90 | 100 | 100 | 100 | 96 | 94 |
| Rv1604 | *impA* | 270 | 52 | 59 | 59 | 58 | 50 | 55 | 50 | 44 | 72 | 71 | 72 | 72 | 78 | 85 | 84 | 86 | 99 | 77 | 81 | 81 | 81 | 81 | 99 | 100 | 99 | 85 | 79 |
| Rv1605 | *hisF* | 267 | 84 | 84 | 85 | 85 | 84 | 83 | 82 | 83 | 89 | 90 | 90 | 91 | 90 | 95 | 96 | 95 | 99 | 93 | 93 | 93 | 93 | 93 | 100 | 100 | 100 | 96 | 94 |
| Rv1606 | *hisI* | 115 | 72 | 77 | 72 | 71 | 79 | 77 | 77 | 77 | 83 | 84 | 82 | 82 | 80 | 93 | 94 | 92 | 100 | 93 | 85 | 85 | 85 | 90 | 100 | 100 | 99 | 94 | 90 |
| Rv1607 | *chaA* | 360 | 0 | 0 | 51 | 52 | 0 | 0 | 0 | 0 | 77 | 0 | 80 | 79 | 0 | 86 | 91 | 86 | 100 | 0 | 0 | 0 | 0 | 0 | 100 | 100 | 100 | 90 | 0 |
| Rv1608c | *bcpB* | 154 | 53 | 56 | 55 | 54 | 54 | 48 | 54 | 52 | 75 | 74 | 75 | 75 | 83 | 90 | 90 | 90 | 100 | 87 | 85 | 84 | 84 | 85 | 100 | 100 | 100 | 90 | 85 |
| Rv1609 | *trpE* | 516 | 54 | 53 | 50 | 52 | 73 | 74 | 72 | 55 | 82 | 82 | 83 | 83 | 88 | 92 | 92 | 92 | 100 | 91 | 90 | 90 | 90 | 90 | 100 | 100 | 100 | 92 | 91 |
| Rv1610 | - | 235 | 43 | 47 | 48 | 48 | 43 | 0 | 43 | 0 | 50 | 50 | 52 | 52 | 64 | 84 | 77 | 73 | 100 | 64 | 67 | 67 | 67 | 64 | 100 | 100 | 100 | 75 | 63 |
| Rv1611 | *trpC* | 272 | 63 | 69 | 52 | 51 | 78 | 82 | 76 | 70 | 90 | 89 | 89 | 90 | 93 | 97 | 96 | 97 | 100 | 92 | 93 | 93 | 93 | 94 | 99 | 100 | 100 | 96 | 93 |
| Rv1612 | *trpB* | 410 | 73 | 72 | 72 | 73 | 80 | 80 | 79 | 76 | 89 | 88 | 88 | 88 | 89 | 93 | 95 | 93 | 100 | 90 | 92 | 92 | 92 | 92 | 100 | 100 | 100 | 95 | 91 |
| Rv1613 | *trpA* | 270 | 47 | 49 | 48 | 47 | 70 | 71 | 66 | 69 | 81 | 81 | 80 | 81 | 78 | 90 | 91 | 91 | 100 | 85 | 90 | 91 | 91 | 86 | 100 | 100 | 100 | 90 | 88 |
| Rv1614 | *lgt* | 468 | 70 | 68 | 63 | 66 | 64 | 67 | 65 | 60 | 60 | 68 | 67 | 75 | 79 | 69 | 71 | 69 | 100 | 67 | 67 | 67 | 67 | 62 | 100 | 100 | 100 | 83 | 71 |
| Rv1615 | - | 146 | 0 | 0 | 0 | 0 | 0 | 0 | 0 | 56 | 67 | 62 | 59 | 65 | 69 | 0 | 87 | 0 | 100 | 64 | 76 | 76 | 76 | 77 | 100 | 100 | 99 | 87 | 77 |
| Rv1616 | - | 132 | 0 | 0 | 0 | 0 | 0 | 0 | 0 | 50 | 0 | 53 | 51 | 54 | 48 | 78 | 77 | 77 | 100 | 70 | 71 | 71 | 71 | 69 | 100 | 100 | 100 | 78 | 68 |
| Rv1617 | *pykA* | 472 | 82 | 81 | 84 | 83 | 84 | 85 | 84 | 72 | 89 | 88 | 90 | 89 | 91 | 96 | 97 | 96 | 100 | 91 | 93 | 93 | 93 | 93 | 100 | 100 | 100 | 97 | 92 |
| Rv1618 | *tesB1* | 300 | 47 | 48 | 47 | 48 | 46 | 0 | 48 | 52 | 71 | 69 | 72 | 72 | 79 | 93 | 93 | 92 | 99 | 83 | 83 | 83 | 83 | 84 | 99 | 100 | 99 | 93 | 86 |
| Rv1619 | - | 484 | 40 | 46 | 0 | 0 | 43 | 43 | 0 | 0 | 42 | 43 | 64 | 60 | 70 | 80 | 83 | 80 | 100 | 43 | 45 | 45 | 45 | 47 | 100 | 100 | 100 | 83 | 48 |
| Rv1620c | *cydC* | 576 | 43 | 51 | 59 | 58 | 39 | 41 | 54 | 48 | 60 | 67 | 68 | 67 | 67 | 82 | 81 | 81 | 100 | 66 | 69 | 69 | 69 | 68 | 100 | 100 | 100 | 80 | 66 |
| Rv1621c | *cydD* | 527 | 44 | 53 | 56 | 54 | 43 | 45 | 49 | 55 | 61 | 68 | 68 | 68 | 69 | 84 | 85 | 85 | 100 | 72 | 75 | 75 | 75 | 72 | 100 | 100 | 100 | 83 | 71 |

| | | | | | | | | | | | | | | | | | | | | | | | | | | | | | |
|---|---|---|---|---|---|---|---|---|---|---|---|---|---|---|---|---|---|---|---|---|---|---|---|---|---|---|---|---|---|
| Rv1622c | *cydB* | 346 | 0 | 67 | 63 | 64 | 0 | 0 | 62 | 63 | 70 | 74 | 72 | 71 | 74 | 89 | 91 | 89 | 100 | 80 | 79 | 78 | 78 | 78 | 100 | 100 | 100 | 90 | 80 |
| Rv1623c | *cydA* | 485 | 0 | 75 | 77 | 75 | 0 | 0 | 68 | 65 | 79 | 78 | 77 | 77 | 81 | 86 | 90 | 86 | 100 | 82 | 85 | 85 | 85 | 86 | 100 | 100 | 100 | 89 | 84 |
| Rv1624c | - | 195 | 0 | 0 | 0 | 0 | 0 | 52 | 0 | 0 | 70 | 60 | 59 | 60 | 77 | 87 | 87 | 87 | 99 | 80 | 70 | 70 | 70 | 83 | 100 | 100 | 100 | 90 | 81 |
| Rv1625c | *cya* | 443 | 49 | 47 | 49 | 50 | 0 | 53 | 48 | 0 | 51 | 52 | 41 | 39 | 46 | 81 | 81 | 81 | 100 | 72 | 73 | 73 | 73 | 79 | 100 | 100 | 100 | 89 | 73 |
| Rv1626 | - | 205 | 55 | 52 | 53 | 54 | 54 | 55 | 54 | 81 | 89 | 87 | 88 | 88 | 91 | 96 | 96 | 96 | 100 | 93 | 92 | 92 | 92 | 93 | 100 | 100 | 100 | 96 | 93 |
| Rv1627c | - | 402 | 0 | 0 | 39 | 0 | 0 | 0 | 0 | 81 | 48 | 82 | 48 | 48 | 93 | 95 | 95 | 95 | 100 | 93 | 96 | 96 | 96 | 95 | 100 | 100 | 100 | 94 | 94 |
| Rv1628c | - | 163 | 0 | 0 | 0 | 0 | 0 | 0 | 0 | 63 | 0 | 64 | 0 | 0 | 83 | 89 | 89 | 89 | 100 | 84 | 87 | 86 | 86 | 90 | 100 | 100 | 100 | 93 | 74 |
| Rv1629 | *polA* | 904 | 71 | 71 | 71 | 71 | 68 | 67 | 68 | 74 | 85 | 84 | 85 | 85 | 88 | 92 | 93 | 92 | 100 | 90 | 88 | 89 | 89 | 91 | 100 | 100 | 100 | 93 | 90 |
| Rv1630 | *rpsA* | 481 | 90 | 91 | 91 | 90 | 91 | 92 | 91 | 86 | 94 | 92 | 92 | 92 | 95 | 97 | 97 | 97 | 99 | 95 | 95 | 95 | 95 | 96 | 100 | 100 | 100 | 97 | 96 |
| Rv1631 | *coaE* | 407 | 62 | 63 | 64 | 66 | 60 | 67 | 60 | 66 | 61 | 69 | 69 | 69 | 71 | 87 | 87 | 87 | 100 | 78 | 73 | 73 | 73 | 81 | 100 | 100 | 100 | 85 | 76 |
| Rv1632c | - | 147 | 59 | 53 | 59 | 59 | 60 | 45 | 0 | 0 | 71 | 71 | 74 | 75 | 80 | 89 | 86 | 90 | 100 | 76 | 83 | 83 | 83 | 82 | 100 | 100 | 100 | 86 | 77 |
| Rv1633 | *uvrB* | 698 | 86 | 86 | 86 | 86 | 88 | 84 | 86 | 83 | 94 | 93 | 94 | 94 | 94 | 97 | 97 | 97 | 100 | 95 | 96 | 96 | 96 | 96 | 100 | 100 | 100 | 96 | 95 |
| Rv1634 | - | 471 | 42 | 41 | 43 | 42 | 45 | 44 | 42 | 42 | 58 | 68 | 61 | 56 | 68 | 78 | 87 | 81 | 99 | 46 | 73 | 73 | 73 | 76 | 100 | 100 | 100 | 87 | 77 |
| Rv1635c | - | 556 | 0 | 0 | 0 | 0 | 0 | 0 | 0 | 0 | 0 | 0 | 51 | 51 | 0 | 78 | 83 | 79 | 100 | 51 | 0 | 0 | 0 | 0 | 100 | 100 | 100 | 83 | 0 |
| Rv1636 | *TB15.3* | 146 | 64 | 65 | 65 | 67 | 67 | 65 | 66 | 0 | 80 | 81 | 79 | 79 | 84 | 94 | 97 | 95 | 100 | 88 | 88 | 88 | 88 | 88 | 100 | 100 | 100 | 97 | 89 |
| Rv1637c | - | 264 | 59 | 58 | 57 | 56 | 58 | 54 | 55 | 54 | 74 | 70 | 74 | 76 | 72 | 80 | 81 | 81 | 100 | 82 | 85 | 84 | 84 | 83 | 100 | 100 | 100 | 85 | 83 |
| Rv1638 | *uvrA* | 972 | 82 | 83 | 83 | 84 | 83 | 82 | 83 | 83 | 91 | 93 | 92 | 92 | 94 | 96 | 96 | 96 | 100 | 92 | 94 | 94 | 94 | 94 | 100 | 100 | 100 | 96 | 93 |
| Rv1638A | - | 85 | 0 | 0 | 0 | 0 | 0 | 0 | 0 | 0 | 73 | 75 | 73 | 71 | 70 | 77 | 81 | 77 | 100 | 66 | 72 | 72 | 72 | 74 | 100 | 100 | 100 | 83 | 77 |
| Rv1639c | - | 489 | 48 | 44 | 0 | 46 | 46 | 51 | 0 | 0 | 0 | 44 | 42 | 44 | 69 | 80 | 82 | 81 | 100 | 70 | 67 | 67 | 67 | 71 | 100 | 100 | 99 | 82 | 72 |
| Rv1640c | *lysS* | 1172 | 55 | 54 | 58 | 58 | 59 | 67 | 58 | 64 | 75 | 73 | 72 | 74 | 77 | 87 | 87 | 87 | 99 | 83 | 84 | 83 | 83 | 85 | 100 | 100 | 100 | 90 | 83 |
| Rv1641 | *infC* | 201 | 88 | 88 | 90 | 89 | 89 | 88 | 89 | 85 | 90 | 89 | 90 | 89 | 91 | 93 | 94 | 94 | 100 | 91 | 90 | 90 | 90 | 94 | 100 | 100 | 100 | 99 | 93 |
| Rv1642 | *rpmI* | 64 | 73 | 73 | 76 | 73 | 73 | 80 | 75 | 72 | 83 | 79 | 79 | 79 | 89 | 95 | 93 | 95 | 100 | 92 | 89 | 89 | 89 | 89 | 100 | 100 | 100 | 93 | 92 |
| Rv1643 | *rplT* | 129 | 89 | 89 | 88 | 88 | 86 | 88 | 88 | 79 | 93 | 87 | 90 | 90 | 95 | 97 | 99 | 97 | 100 | 93 | 96 | 96 | 96 | 97 | 100 | 100 | 100 | 99 | 97 |
| Rv1644 | *tsnR* | 260 | 65 | 66 | 67 | 68 | 66 | 59 | 65 | 62 | 72 | 69 | 70 | 70 | 72 | 85 | 86 | 86 | 99 | 78 | 80 | 80 | 80 | 77 | 99 | 100 | 99 | 85 | 81 |
| Rv1645c | - | 351 | 0 | 0 | 0 | 0 | 0 | 0 | 0 | 0 | 58 | 68 | 67 | 67 | 68 | 89 | 92 | 89 | 100 | 88 | 87 | 86 | 86 | 88 | 100 | 100 | 100 | 91 | 89 |
| Rv1646 | *PE17* | 310 | 0 | 0 | 0 | 0 | 0 | 0 | 0 | 0 | 0 | 0 | 0 | 0 | 0 | 57 | 46 | 46 | 100 | 0 | 0 | 0 | 0 | 0 | 100 | 100 | 100 | 69 | 0 |
| Rv1647 | - | 316 | 0 | 0 | 0 | 0 | 0 | 0 | 0 | 0 | 64 | 67 | 68 | 68 | 71 | 87 | 88 | 83 | 99 | 82 | 75 | 73 | 73 | 80 | 99 | 100 | 99 | 88 | 76 |
| Rv1648 | - | 268 | 0 | 0 | 0 | 0 | 0 | 0 | 0 | 0 | 0 | 46 | 45 | 0 | 60 | 73 | 81 | 73 | 100 | 0 | 48 | 49 | 49 | 66 | 100 | 100 | 100 | 80 | 0 |
| Rv1649 | *pheS* | 341 | 75 | 78 | 77 | 78 | 80 | 78 | 80 | 69 | 84 | 86 | 86 | 86 | 88 | 91 | 91 | 91 | 99 | 88 | 89 | 89 | 89 | 89 | 100 | 100 | 100 | 90 | 88 |
| Rv1650 | *pheT* | 831 | 63 | 62 | 65 | 64 | 63 | 65 | 63 | 60 | 73 | 75 | 72 | 71 | 77 | 89 | 88 | 89 | 99 | 83 | 85 | 85 | 85 | 84 | 100 | 100 | 100 | 87 | 85 |
| Rv1651c | *PE_PGRS30* | 1011 | 47 | 33 | 34 | 33 | 37 | 38 | 37 | 35 | 39 | 38 | 43 | 40 | 39 | 46 | 58 | 36 | 99 | 47 | 49 | 51 | 51 | 42 | 99 | 100 | 99 | 52 | 51 |
| Rv1652 | *argC* | 352 | 67 | 72 | 73 | 74 | 65 | 66 | 65 | 73 | 78 | 78 | 77 | 77 | 84 | 90 | 91 | 90 | 100 | 88 | 89 | 89 | 89 | 87 | 100 | 100 | 100 | 91 | 88 |
| Rv1653 | *argJ* | 404 | 70 | 69 | 70 | 71 | 71 | 67 | 71 | 65 | 86 | 84 | 84 | 84 | 85 | 90 | 91 | 90 | 100 | 90 | 89 | 90 | 90 | 89 | 100 | 100 | 100 | 91 | 89 |
| Rv1654 | *argB* | 294 | 79 | 79 | 80 | 81 | 78 | 78 | 78 | 78 | 85 | 87 | 84 | 85 | 90 | 96 | 96 | 96 | 100 | 91 | 92 | 91 | 91 | 94 | 99 | 100 | 100 | 93 | 91 |
| Rv1655 | *argD* | 400 | 68 | 66 | 69 | 68 | 66 | 63 | 68 | 70 | 77 | 79 | 79 | 79 | 81 | 88 | 88 | 88 | 100 | 88 | 87 | 87 | 87 | 86 | 100 | 100 | 100 | 89 | 87 |
| Rv1656 | *argF* | 307 | 75 | 75 | 77 | 76 | 73 | 75 | 73 | 71 | 86 | 87 | 88 | 88 | 89 | 89 | 94 | 89 | 100 | 88 | 89 | 89 | 89 | 89 | 100 | 100 | 100 | 93 | 89 |
| Rv1657 | *argR* | 170 | 70 | 64 | 68 | 72 | 62 | 70 | 63 | 67 | 85 | 89 | 86 | 85 | 90 | 88 | 85 | 84 | 100 | 87 | 89 | 89 | 89 | 91 | 100 | 100 | 100 | 85 | 90 |
| Rv1658 | *argG* | 398 | 84 | 86 | 85 | 84 | 85 | 85 | 86 | 46 | 89 | 92 | 92 | 92 | 95 | 97 | 97 | 97 | 100 | 96 | 95 | 95 | 95 | 96 | 100 | 100 | 100 | 96 | 95 |

| | | | | | | | | | | | | | | | | | | | | | | | | | | | | | |
|---|---|---|---|---|---|---|---|---|---|---|---|---|---|---|---|---|---|---|---|---|---|---|---|---|---|---|---|---|---|
| Rv1659 | *argH* | 470 | 82 | 82 | 86 | 85 | 84 | 82 | 84 | 80 | 88 | 87 | 87 | 87 | 87 | 94 | 95 | 94 | 100 | 92 | 91 | 92 | 92 | 91 | 100 | 100 | 100 | 95 | 92 |
| Rv1660 | *pks10* | 353 | 0 | 0 | 0 | 0 | 0 | 0 | 0 | 0 | 43 | 0 | 0 | 0 | 0 | 90 | 94 | 90 | 100 | 81 | 81 | 81 | 81 | 81 | 100 | 100 | 100 | 86 | 83 |
| Rv1661 | *pks7* | 2126 | 52 | 49 | 52 | 52 | 47 | 44 | 50 | 46 | 49 | 48 | 62 | 59 | 51 | 79 | 79 | 80 | 99 | 51 | 51 | 51 | 51 | 51 | 100 | 100 | 100 | 78 | 51 |
| Rv1662 | *pks8* | 1602 | 50 | 51 | 51 | 51 | 48 | 47 | 48 | 54 | 51 | 50 | 58 | 56 | 54 | 71 | 77 | 78 | 99 | 58 | 50 | 50 | 50 | 58 | 99 | 100 | 99 | 75 | 58 |
| Rv1663 | *pks17* | 502 | 0 | 0 | 0 | 0 | 0 | 0 | 0 | 0 | 44 | 44 | 64 | 64 | 50 | 73 | 75 | 73 | 100 | 50 | 50 | 50 | 50 | 50 | 100 | 100 | 100 | 74 | 49 |
| Rv1664 | *pks9* | 1017 | 52 | 51 | 51 | 51 | 50 | 0 | 49 | 42 | 52 | 51 | 57 | 57 | 52 | 64 | 76 | 73 | 99 | 54 | 55 | 56 | 56 | 54 | 100 | 100 | 100 | 75 | 53 |
| Rv1665 | *pks11* | 353 | 0 | 0 | 0 | 0 | 0 | 0 | 0 | 0 | 43 | 0 | 0 | 0 | 0 | 86 | 87 | 87 | 100 | 82 | 80 | 80 | 80 | 80 | 100 | 100 | 100 | 91 | 83 |
| Rv1666c | *cyp139* | 430 | 0 | 0 | 0 | 0 | 0 | 0 | 0 | 43 | 44 | 45 | 43 | 45 | 44 | 83 | 84 | 84 | 100 | 45 | 47 | 48 | 48 | 48 | 100 | 100 | 100 | 82 | 48 |
| Rv1667c | - | 217 | 57 | 61 | 58 | 60 | 60 | 58 | 58 | 60 | 62 | 61 | 61 | 64 | 66 | 85 | 88 | 86 | 100 | 63 | 72 | 61 | 61 | 84 | 100 | 100 | 100 | 88 | 82 |
| Rv1668c | - | 372 | 67 | 68 | 66 | 67 | 68 | 69 | 68 | 72 | 68 | 67 | 68 | 68 | 65 | 91 | 92 | 91 | 100 | 69 | 81 | 69 | 69 | 89 | 100 | 100 | 100 | 92 | 84 |
| Rv1669 | - | 120 | 0 | 0 | 0 | 0 | 0 | 0 | 0 | 0 | 0 | 0 | 0 | 0 | 0 | 0 | 0 | 0 | 100 | 0 | 0 | 0 | 0 | 0 | 100 | 100 | 100 | 0 | 0 |
| Rv1670 | - | 115 | 0 | 0 | 0 | 0 | 0 | 0 | 0 | 70 | 67 | 0 | 0 | 0 | 0 | 0 | 81 | 0 | 100 | 0 | 0 | 0 | 0 | 0 | 99 | 100 | 100 | 80 | 0 |
| Rv1671 | - | 130 | 0 | 0 | 0 | 0 | 0 | 0 | 0 | 0 | 0 | 0 | 0 | 0 | 0 | 0 | 0 | 0 | 100 | 0 | 0 | 0 | 0 | 0 | 100 | 100 | 100 | 0 | 0 |
| Rv1672c | - | 443 | 36 | 0 | 38 | 37 | 0 | 0 | 0 | 40 | 52 | 58 | 59 | 58 | 0 | 0 | 56 | 0 | 100 | 38 | 55 | 55 | 55 | 53 | 100 | 100 | 100 | 55 | 0 |
| Rv1673c | - | 310 | 0 | 0 | 0 | 0 | 0 | 0 | 0 | 0 | 0 | 0 | 0 | 0 | 0 | 0 | 0 | 0 | 100 | 0 | 0 | 0 | 0 | 0 | 100 | 100 | 100 | 0 | 0 |
| Rv1674c | - | 218 | 63 | 62 | 64 | 0 | 0 | 0 | 0 | 0 | 64 | 0 | 0 | 0 | 0 | 0 | 0 | 0 | 100 | 0 | 71 | 71 | 71 | 0 | 100 | 100 | 99 | 0 | 0 |
| Rv1675c | - | 244 | 47 | 0 | 46 | 45 | 0 | 0 | 0 | 63 | 49 | 46 | 47 | 47 | 47 | 47 | 77 | 47 | 99 | 47 | 48 | 48 | 48 | 47 | 100 | 100 | 100 | 76 | 72 |
| Rv1676 | - | 234 | 0 | 0 | 0 | 0 | 0 | 0 | 0 | 0 | 60 | 0 | 0 | 0 | 0 | 0 | 63 | 0 | 100 | 0 | 0 | 0 | 0 | 57 | 100 | 100 | 100 | 62 | 77 |
| Rv1677 | *dsbF* | 182 | 0 | 0 | 0 | 0 | 0 | 0 | 0 | 48 | 0 | 0 | 0 | 0 | 72 | 62 | 68 | 61 | 100 | 0 | 0 | 0 | 0 | 59 | 100 | 100 | 100 | 67 | 0 |
| Rv1678 | - | 300 | 0 | 0 | 0 | 0 | 0 | 0 | 0 | 0 | 0 | 0 | 0 | 0 | 0 | 0 | 0 | 0 | 100 | 0 | 0 | 0 | 0 | 0 | 100 | 100 | 100 | 0 | 72 |
| Rv1679 | *fadE16* | 373 | 0 | 0 | 0 | 0 | 47 | 46 | 49 | 45 | 44 | 46 | 47 | 47 | 46 | 44 | 44 | 44 | 100 | 46 | 40 | 45 | 45 | 45 | 100 | 100 | 100 | 45 | 81 |
| Rv1680 | - | 274 | 0 | 0 | 0 | 0 | 0 | 0 | 0 | 0 | 0 | 0 | 0 | 0 | 0 | 0 | 0 | 0 | 99 | 0 | 0 | 0 | 0 | 0 | 100 | 100 | 100 | 0 | 86 |
| Rv1681 | *moeX* | 330 | 0 | 0 | 0 | 0 | 0 | 0 | 0 | 0 | 46 | 0 | 0 | 0 | 0 | 0 | 42 | 0 | 100 | 0 | 0 | 0 | 0 | 0 | 100 | 100 | 100 | 42 | 80 |
| Rv1682 | - | 305 | 0 | 0 | 0 | 0 | 0 | 0 | 0 | 45 | 56 | 0 | 0 | 0 | 76 | 56 | 0 | 56 | 100 | 0 | 0 | 0 | 0 | 0 | 100 | 100 | 100 | 0 | 0 |
| Rv1683 | - | 999 | 0 | 0 | 0 | 0 | 0 | 0 | 0 | 43 | 68 | 73 | 72 | 72 | 78 | 90 | 92 | 90 | 100 | 84 | 86 | 86 | 86 | 84 | 100 | 100 | 100 | 91 | 84 |
| Rv1684 | - | 74 | 0 | 71 | 70 | 70 | 65 | 75 | 0 | 0 | 78 | 83 | 79 | 77 | 75 | 89 | 85 | 89 | 100 | 81 | 79 | 79 | 79 | 82 | 100 | 100 | 100 | 85 | 76 |
| Rv1685c | - | 207 | 0 | 0 | 0 | 0 | 0 | 53 | 0 | 61 | 65 | 69 | 70 | 73 | 75 | 87 | 88 | 87 | 100 | 55 | 86 | 86 | 86 | 87 | 100 | 100 | 100 | 83 | 86 |
| Rv1686c | - | 226 | 0 | 0 | 0 | 66 | 0 | 65 | 0 | 67 | 77 | 77 | 78 | 77 | 0 | 93 | 93 | 93 | 99 | 0 | 88 | 88 | 88 | 88 | 100 | 100 | 100 | 93 | 87 |
| Rv1687c | - | 255 | 53 | 58 | 55 | 71 | 55 | 65 | 55 | 67 | 72 | 70 | 74 | 72 | 53 | 87 | 80 | 87 | 100 | 51 | 82 | 83 | 83 | 83 | 100 | 100 | 100 | 79 | 79 |
| Rv1688 | *mpg* | 203 | 55 | 0 | 54 | 57 | 52 | 50 | 50 | 59 | 65 | 70 | 69 | 70 | 74 | 86 | 85 | 86 | 100 | 79 | 78 | 78 | 78 | 74 | 100 | 100 | 100 | 85 | 78 |
| Rv1689 | *tyrS* | 424 | 76 | 75 | 81 | 82 | 76 | 79 | 74 | 68 | 85 | 86 | 86 | 86 | 87 | 93 | 93 | 93 | 100 | 89 | 89 | 90 | 90 | 88 | 100 | 100 | 100 | 93 | 89 |
| Rv1690 | *lprJ* | 127 | 0 | 0 | 0 | 0 | 0 | 0 | 0 | 0 | 0 | 0 | 0 | 0 | 0 | 77 | 76 | 76 | 100 | 0 | 58 | 58 | 58 | 57 | 100 | 100 | 100 | 74 | 54 |
| Rv1691 | - | 250 | 58 | 64 | 67 | 58 | 63 | 65 | 57 | 59 | 69 | 71 | 70 | 70 | 81 | 82 | 89 | 82 | 100 | 75 | 84 | 84 | 84 | 80 | 100 | 100 | 100 | 88 | 86 |
| Rv1692 | - | 353 | 50 | 57 | 57 | 56 | 53 | 64 | 56 | 54 | 69 | 66 | 68 | 68 | 68 | 83 | 83 | 83 | 100 | 75 | 74 | 75 | 75 | 75 | 100 | 100 | 100 | 81 | 77 |
| Rv1693 | - | 58 | 0 | 0 | 0 | 0 | 0 | 0 | 0 | 0 | 0 | 0 | 0 | 0 | 0 | 83 | 83 | 83 | 100 | 0 | 0 | 0 | 0 | 0 | 100 | 100 | 100 | 86 | 0 |
| Rv1694 | *tlyA* | 268 | 68 | 68 | 66 | 66 | 69 | 68 | 72 | 71 | 70 | 72 | 73 | 73 | 78 | 86 | 86 | 86 | 100 | 81 | 83 | 83 | 83 | 83 | 100 | 100 | 100 | 86 | 83 |
| Rv1695 | *ppnK* | 307 | 67 | 66 | 69 | 72 | 68 | 68 | 68 | 70 | 74 | 77 | 76 | 77 | 87 | 94 | 95 | 94 | 100 | 91 | 92 | 92 | 92 | 91 | 100 | 100 | 100 | 95 | 93 |
| Rv1696 | *recN* | 587 | 62 | 63 | 64 | 63 | 60 | 62 | 60 | 59 | 73 | 72 | 72 | 72 | 76 | 83 | 87 | 83 | 100 | 79 | 78 | 78 | 78 | 73 | 99 | 100 | 100 | 87 | 80 |

| | | | | | | | | | | | | | | | | | | | | | | | | | | | | | |
|---|---|---|---|---|---|---|---|---|---|---|---|---|---|---|---|---|---|---|---|---|---|---|---|---|---|---|---|---|---|
| Rv1697 | - | 393 | 59 | 63 | 62 | 60 | 63 | 61 | 62 | 56 | 77 | 81 | 82 | 82 | 85 | 94 | 97 | 94 | 100 | 89 | 88 | 88 | 88 | 89 | 100 | 100 | 100 | 97 | 89 |
| Rv1698 | - | 314 | 48 | 51 | 50 | 50 | 48 | 48 | 44 | 43 | 62 | 67 | 65 | 65 | 72 | 85 | 87 | 86 | 100 | 77 | 78 | 78 | 78 | 78 | 100 | 100 | 100 | 87 | 78 |
| Rv1699 | *pyrG* | 586 | 0 | 0 | 82 | 83 | 84 | 86 | 0 | 82 | 88 | 90 | 91 | 90 | 92 | 94 | 94 | 94 | 100 | 92 | 91 | 90 | 90 | 91 | 99 | 100 | 100 | 94 | 93 |
| Rv1700 | - | 207 | 65 | 62 | 65 | 63 | 61 | 63 | 0 | 56 | 69 | 70 | 64 | 63 | 74 | 87 | 86 | 87 | 99 | 76 | 81 | 81 | 81 | 78 | 100 | 100 | 100 | 85 | 79 |
| Rv1701 | *xerD* | 311 | 68 | 72 | 71 | 69 | 69 | 71 | 68 | 77 | 78 | 83 | 85 | 46 | 87 | 94 | 95 | 94 | 100 | 92 | 94 | 94 | 94 | 93 | 100 | 100 | 100 | 95 | 92 |
| Rv1702c | - | 454 | 0 | 0 | 44 | 43 | 45 | 0 | 0 | 0 | 0 | 40 | 52 | 49 | 66 | 76 | 75 | 75 | 100 | 61 | 57 | 57 | 57 | 56 | 100 | 100 | 100 | 74 | 60 |
| Rv1703c | - | 196 | 0 | 0 | 0 | 0 | 0 | 0 | 0 | 0 | 0 | 0 | 0 | 0 | 80 | 89 | 90 | 89 | 100 | 82 | 0 | 0 | 0 | 47 | 100 | 100 | 99 | 90 | 84 |
| Rv1704c | *cycA* | 556 | 56 | 52 | 76 | 77 | 51 | 56 | 60 | 39 | 58 | 79 | 77 | 78 | 60 | 54 | 54 | 54 | 99 | 54 | 57 | 57 | 57 | 79 | 99 | 100 | 99 | 87 | 38 |
| Rv1705c | *PPE22* | 385 | 0 | 0 | 0 | 0 | 0 | 0 | 0 | 0 | 36 | 38 | 37 | 36 | 42 | 62 | 79 | 62 | 100 | 41 | 41 | 41 | 41 | 41 | 99 | 100 | 100 | 79 | 42 |
| Rv1706A | - | 55 | 0 | 0 | 0 | 0 | 0 | 0 | 0 | 0 | 0 | 0 | 0 | 0 | 0 | 0 | 0 | 0 | 100 | 0 | 0 | 0 | 0 | 0 | 100 | 100 | 100 | 0 | 0 |
| Rv1706c | *PPE23* | 394 | 0 | 0 | 0 | 0 | 0 | 0 | 0 | 0 | 40 | 0 | 36 | 0 | 44 | 52 | 62 | 51 | 100 | 51 | 41 | 41 | 41 | 42 | 100 | 100 | 100 | 62 | 44 |
| Rv1707 | - | 486 | 49 | 0 | 51 | 52 | 50 | 48 | 50 | 41 | 47 | 51 | 48 | 50 | 48 | 47 | 47 | 47 | 100 | 50 | 50 | 50 | 50 | 51 | 100 | 100 | 100 | 49 | 48 |
| Rv1708 | - | 318 | 83 | 82 | 83 | 83 | 80 | 80 | 86 | 80 | 82 | 89 | 90 | 89 | 89 | 90 | 93 | 90 | 99 | 85 | 88 | 88 | 88 | 87 | 100 | 100 | 100 | 93 | 85 |
| Rv1709 | - | 278 | 75 | 73 | 76 | 76 | 72 | 72 | 71 | 70 | 75 | 79 | 81 | 81 | 83 | 90 | 92 | 90 | 100 | 87 | 87 | 87 | 87 | 89 | 100 | 100 | 100 | 92 | 89 |
| Rv1710 | - | 231 | 68 | 72 | 68 | 75 | 65 | 72 | 66 | 63 | 74 | 75 | 78 | 78 | 81 | 91 | 89 | 92 | 100 | 82 | 83 | 83 | 83 | 85 | 100 | 100 | 100 | 89 | 85 |
| Rv1711 | - | 254 | 75 | 76 | 76 | 73 | 76 | 76 | 76 | 67 | 79 | 79 | 81 | 81 | 82 | 94 | 92 | 95 | 100 | 89 | 92 | 92 | 92 | 91 | 100 | 100 | 100 | 92 | 88 |
| Rv1712 | *cmk* | 230 | 72 | 69 | 69 | 69 | 69 | 64 | 72 | 64 | 73 | 77 | 75 | 76 | 80 | 84 | 87 | 85 | 100 | 79 | 85 | 85 | 85 | 85 | 100 | 100 | 100 | 87 | 85 |
| Rv1713 | *engA* | 463 | 77 | 77 | 78 | 77 | 77 | 76 | 77 | 79 | 84 | 82 | 84 | 84 | 87 | 92 | 93 | 92 | 100 | 88 | 89 | 89 | 89 | 89 | 100 | 100 | 100 | 93 | 89 |
| Rv1714 | - | 270 | 46 | 0 | 49 | 48 | 46 | 48 | 48 | 54 | 50 | 55 | 56 | 57 | 66 | 50 | 50 | 50 | 100 | 54 | 56 | 56 | 56 | 54 | 100 | 100 | 100 | 51 | 53 |
| Rv1715 | *fadB3* | 304 | 47 | 0 | 54 | 0 | 50 | 47 | 51 | 54 | 51 | 52 | 50 | 50 | 60 | 47 | 51 | 47 | 99 | 53 | 51 | 51 | 51 | 50 | 100 | 100 | 100 | 51 | 53 |
| Rv1716 | - | 276 | 0 | 0 | 0 | 0 | 0 | 0 | 0 | 0 | 36 | 0 | 45 | 38 | 82 | 0 | 0 | 0 | 99 | 0 | 0 | 0 | 0 | 44 | 99 | 100 | 99 | 0 | 0 |
| Rv1717 | - | 116 | 0 | 0 | 0 | 0 | 0 | 0 | 0 | 0 | 0 | 0 | 0 | 0 | 56 | 0 | 0 | 0 | 100 | 0 | 0 | 0 | 0 | 0 | 100 | 100 | 100 | 0 | 0 |
| Rv1718 | - | 272 | 0 | 0 | 0 | 0 | 0 | 0 | 0 | 50 | 84 | 49 | 0 | 50 | 0 | 0 | 0 | 0 | 100 | 0 | 44 | 44 | 44 | 49 | 100 | 100 | 100 | 0 | 0 |
| Rv1719 | - | 259 | 0 | 0 | 49 | 49 | 0 | 0 | 0 | 50 | 48 | 47 | 52 | 52 | 54 | 48 | 50 | 48 | 100 | 50 | 47 | 47 | 47 | 50 | 100 | 100 | 100 | 43 | 52 |
| Rv1720c | - | 129 | 0 | 0 | 0 | 0 | 0 | 0 | 0 | 0 | 0 | 0 | 0 | 0 | 0 | 0 | 0 | 0 | 100 | 54 | 0 | 0 | 0 | 0 | 100 | 100 | 100 | 0 | 0 |
| Rv1721c | - | 75 | 0 | 0 | 0 | 0 | 0 | 0 | 0 | 0 | 0 | 0 | 0 | 0 | 0 | 0 | 0 | 0 | 100 | 0 | 0 | 0 | 0 | 0 | 100 | 100 | 100 | 0 | 0 |
| Rv1722 | - | 494 | 0 | 0 | 0 | 0 | 0 | 0 | 0 | 0 | 0 | 0 | 0 | 0 | 0 | 90 | 90 | 90 | 100 | 0 | 0 | 0 | 0 | 0 | 100 | 100 | 100 | 0 | 0 |
| Rv1723 | - | 415 | 0 | 0 | 0 | 0 | 0 | 0 | 0 | 0 | 0 | 0 | 60 | 0 | 0 | 88 | 89 | 88 | 100 | 0 | 72 | 72 | 72 | 0 | 100 | 100 | 100 | 0 | 0 |
| Rv1724c | - | 139 | 0 | 0 | 0 | 0 | 0 | 0 | 0 | 0 | 0 | 0 | 0 | 0 | 0 | 0 | 0 | 0 | 100 | 0 | 0 | 0 | 0 | 0 | 100 | 100 | 100 | 0 | 0 |
| Rv1725c | - | 236 | 0 | 0 | 0 | 0 | 0 | 0 | 0 | 0 | 61 | 56 | 63 | 60 | 56 | 0 | 52 | 0 | 100 | 47 | 49 | 49 | 49 | 45 | 100 | 100 | 100 | 52 | 48 |
| Rv1726 | - | 461 | 0 | 0 | 0 | 0 | 0 | 0 | 0 | 47 | 45 | 56 | 55 | 56 | 40 | 39 | 41 | 39 | 99 | 42 | 39 | 39 | 39 | 47 | 100 | 100 | 99 | 41 | 40 |
| Rv1727 | - | 189 | 0 | 0 | 0 | 0 | 0 | 0 | 0 | 0 | 48 | 41 | 46 | 49 | 44 | 47 | 47 | 47 | 100 | 0 | 44 | 44 | 44 | 44 | 100 | 100 | 100 | 41 | 42 |
| Rv1728c | - | 256 | 0 | 0 | 0 | 0 | 0 | 0 | 0 | 52 | 0 | 0 | 0 | 0 | 46 | 75 | 76 | 73 | 100 | 76 | 79 | 79 | 79 | 80 | 100 | 100 | 100 | 76 | 78 |
| Rv1729c | - | 312 | 0 | 0 | 0 | 0 | 0 | 0 | 0 | 0 | 52 | 54 | 55 | 0 | 66 | 78 | 78 | 77 | 100 | 76 | 74 | 73 | 73 | 70 | 100 | 100 | 100 | 78 | 74 |
| Rv1730c | - | 517 | 41 | 0 | 0 | 45 | 0 | 0 | 0 | 37 | 46 | 55 | 46 | 46 | 44 | 44 | 45 | 44 | 100 | 45 | 46 | 46 | 46 | 44 | 99 | 100 | 100 | 45 | 47 |
| Rv1731 | *gabD2* | 518 | 63 | 51 | 65 | 65 | 65 | 67 | 67 | 66 | 77 | 80 | 81 | 80 | 54 | 52 | 94 | 52 | 100 | 54 | 56 | 56 | 56 | 84 | 100 | 100 | 100 | 94 | 55 |
| Rv1732c | - | 182 | 0 | 0 | 0 | 0 | 0 | 0 | 0 | 0 | 0 | 0 | 0 | 0 | 0 | 0 | 94 | 0 | 100 | 0 | 0 | 0 | 0 | 0 | 100 | 100 | 100 | 94 | 0 |
| Rv1733c | - | 210 | 0 | 0 | 0 | 0 | 0 | 0 | 0 | 0 | 43 | 44 | 50 | 48 | 0 | 0 | 50 | 56 | 99 | 49 | 53 | 53 | 53 | 57 | 99 | 100 | 99 | 0 | 49 |

| | | | | | | | | | | | | | | | | | | | | | | | | | | | | |
|---|---|---|---|---|---|---|---|---|---|---|---|---|---|---|---|---|---|---|---|---|---|---|---|---|---|---|---|---|
| Rv1734c | - | 80 | 0 | 0 | 0 | 0 | 0 | 0 | 0 | 0 | 0 | 0 | 0 | 0 | 0 | 0 | 0 | 0 | 98 | 0 | 80 | 80 | 80 | 0 | 100 | 100 | 100 | 0 | 83 |
| Rv1735c | - | 165 | 0 | 0 | 0 | 0 | 0 | 0 | 0 | 70 | 0 | 0 | 61 | 70 | 0 | 0 | 0 | 0 | 100 | 0 | 0 | 0 | 0 | 0 | 100 | 100 | 100 | 0 | 0 |
| Rv1736c | *narX* | 652 | 0 | 80 | 78 | 78 | 0 | 0 | 0 | 84 | 84 | 0 | 0 | 83 | 0 | 92 | 0 | 92 | 99 | 94 | 95 | 95 | 95 | 94 | 100 | 100 | 100 | 0 | 95 |
| Rv1737c | *narK2* | 395 | 0 | 46 | 47 | 0 | 0 | 0 | 0 | 59 | 37 | 39 | 39 | 39 | 40 | 39 | 39 | 39 | 100 | 88 | 88 | 88 | 88 | 88 | 100 | 100 | 100 | 39 | 89 |
| Rv1738 | - | 94 | 0 | 0 | 0 | 0 | 0 | 0 | 0 | 69 | 0 | 77 | 70 | 66 | 0 | 0 | 0 | 0 | 100 | 0 | 0 | 0 | 0 | 0 | 100 | 100 | 100 | 73 | 76 |
| Rv1739c | - | 560 | 44 | 0 | 70 | 70 | 45 | 41 | 46 | 52 | 46 | 57 | 57 | 58 | 46 | 45 | 44 | 45 | 99 | 77 | 44 | 44 | 44 | 45 | 99 | 100 | 99 | 44 | 76 |
| Rv1740 | - | 70 | 0 | 0 | 0 | 0 | 0 | 0 | 0 | 0 | 0 | 0 | 0 | 0 | 0 | 0 | 0 | 0 | 100 | 0 | 0 | 0 | 0 | 88 | 100 | 100 | 98 | 0 | 0 |
| Rv1741 | - | 82 | 0 | 0 | 0 | 0 | 0 | 0 | 0 | 0 | 0 | 0 | 0 | 0 | 0 | 0 | 0 | 0 | 98 | 0 | 0 | 0 | 0 | 48 | 100 | 100 | 100 | 0 | 0 |
| Rv1742 | - | 245 | 0 | 0 | 0 | 0 | 0 | 0 | 0 | 0 | 0 | 66 | 0 | 0 | 0 | 0 | 84 | 0 | 100 | 0 | 0 | 0 | 0 | 49 | 100 | 100 | 100 | 84 | 53 |
| Rv1743 | *pknE* | 566 | 52 | 50 | 48 | 58 | 54 | 57 | 55 | 52 | 67 | 70 | 63 | 61 | 52 | 77 | 81 | 66 | 99 | 70 | 60 | 60 | 60 | 61 | 100 | 100 | 100 | 81 | 67 |
| Rv1744c | - | 133 | 0 | 0 | 0 | 0 | 0 | 0 | 0 | 0 | 0 | 0 | 0 | 0 | 0 | 0 | 0 | 0 | 100 | 71 | 63 | 63 | 63 | 0 | 100 | 100 | 100 | 0 | 76 |
| Rv1745c | *idi* | 203 | 58 | 62 | 60 | 60 | 0 | 0 | 0 | 0 | 54 | 0 | 67 | 0 | 0 | 0 | 56 | 0 | 99 | 61 | 0 | 0 | 0 | 0 | 100 | 100 | 100 | 61 | 60 |
| Rv1746 | *pknF* | 476 | 55 | 53 | 44 | 45 | 52 | 54 | 53 | 49 | 50 | 46 | 55 | 52 | 54 | 71 | 88 | 71 | 99 | 70 | 64 | 64 | 64 | 73 | 100 | 100 | 100 | 70 | 65 |
| Rv1747 | - | 865 | 52 | 52 | 54 | 52 | 49 | 54 | 58 | 49 | 63 | 53 | 74 | 74 | 71 | 87 | 86 | 86 | 100 | 80 | 82 | 81 | 81 | 81 | 100 | 100 | 100 | 89 | 81 |
| Rv1748 | - | 243 | 0 | 0 | 0 | 0 | 0 | 0 | 0 | 0 | 0 | 69 | 0 | 0 | 0 | 74 | 79 | 74 | 100 | 72 | 0 | 0 | 0 | 61 | 100 | 100 | 100 | 78 | 71 |
| Rv1749c | - | 185 | 0 | 0 | 0 | 0 | 0 | 0 | 0 | 0 | 0 | 0 | 0 | 0 | 0 | 0 | 82 | 0 | 100 | 0 | 0 | 0 | 0 | 0 | 100 | 100 | 100 | 82 | 0 |
| Rv1750c | *fadD1* | 532 | 38 | 43 | 43 | 44 | 41 | 42 | 43 | 41 | 74 | 62 | 64 | 65 | 73 | 87 | 88 | 87 | 100 | 78 | 76 | 76 | 76 | 75 | 100 | 100 | 100 | 89 | 77 |
| Rv1751 | - | 460 | 0 | 0 | 0 | 0 | 0 | 0 | 0 | 41 | 39 | 0 | 42 | 39 | 72 | 60 | 60 | 60 | 99 | 80 | 39 | 39 | 39 | 74 | 100 | 100 | 100 | 86 | 79 |
| Rv1752 | - | 149 | 0 | 0 | 0 | 0 | 0 | 0 | 0 | 0 | 0 | 0 | 0 | 57 | 0 | 0 | 77 | 73 | 100 | 0 | 0 | 0 | 0 | 0 | 100 | 100 | 100 | 81 | 0 |
| Rv1753c | *PPE24* | 1053 | 0 | 0 | 0 | 0 | 0 | 0 | 0 | 0 | 41 | 39 | 37 | 38 | 50 | 67 | 74 | 67 | 86 | 49 | 48 | 49 | 49 | 46 | 95 | 100 | 81 | 80 | 46 |
| Rv1754c | - | 563 | 52 | 0 | 0 | 0 | 57 | 0 | 54 | 0 | 56 | 63 | 57 | 58 | 65 | 70 | 82 | 70 | 100 | 56 | 56 | 66 | 66 | 68 | 100 | 100 | 99 | 82 | 49 |
| Rv1755c | *plcD* | 280 | 0 | 0 | 0 | 0 | 0 | 0 | 0 | 45 | 0 | 53 | 70 | 70 | 51 | 0 | 83 | 0 | 100 | 0 | 0 | 0 | 0 | 0 | 100 | 100 | 82 | 83 | 0 |
| Rv1756c | - | 294 | 58 | 49 | 61 | 51 | 63 | 0 | 61 | 56 | 0 | 0 | 53 | 52 | 66 | 60 | 66 | 68 | 100 | 66 | 90 | 67 | 67 | 66 | 100 | 100 | 100 | 0 | 65 |
| Rv1757c | - | 108 | 65 | 0 | 66 | 0 | 61 | 0 | 60 | 76 | 0 | 0 | 0 | 0 | 59 | 63 | 66 | 66 | 100 | 82 | 96 | 77 | 77 | 84 | 100 | 100 | 100 | 0 | 62 |
| Rv1758 | *cut1* | 174 | 0 | 0 | 0 | 0 | 0 | 0 | 0 | 0 | 0 | 0 | 62 | 0 | 65 | 75 | 75 | 75 | 98 | 72 | 69 | 69 | 69 | 73 | 100 | 100 | 100 | 74 | 64 |
| Rv1759c | *wag22* | 914 | 42 | 31 | 34 | 32 | 35 | 36 | 41 | 35 | 39 | 37 | 40 | 39 | 44 | 31 | 59 | 32 | 100 | 50 | 48 | 48 | 48 | 41 | 100 | 100 | 99 | 59 | 48 |
| Rv1760 | - | 502 | 0 | 0 | 0 | 0 | 0 | 0 | 0 | 63 | 60 | 67 | 69 | 69 | 73 | 77 | 84 | 77 | 99 | 67 | 72 | 72 | 72 | 74 | 99 | 100 | 99 | 88 | 74 |
| Rv1761c | - | 127 | 0 | 0 | 0 | 0 | 0 | 0 | 0 | 0 | 0 | 0 | 0 | 0 | 0 | 0 | 79 | 0 | 100 | 0 | 0 | 0 | 0 | 0 | 100 | 100 | 100 | 80 | 0 |
| Rv1762c | - | 262 | 0 | 0 | 0 | 0 | 0 | 0 | 0 | 0 | 0 | 0 | 0 | 0 | 0 | 0 | 93 | 0 | 100 | 0 | 0 | 0 | 0 | 0 | 100 | 100 | 100 | 95 | 0 |
| Rv1763 | - | 108 | 65 | 0 | 66 | 0 | 61 | 0 | 60 | 76 | 0 | 0 | 0 | 0 | 59 | 63 | 82 | 66 | 100 | 82 | 96 | 77 | 77 | 84 | 100 | 100 | 100 | 0 | 62 |
| Rv1764 | - | 294 | 58 | 49 | 61 | 51 | 63 | 0 | 61 | 56 | 0 | 0 | 53 | 52 | 66 | 60 | 66 | 68 | 100 | 66 | 90 | 67 | 67 | 66 | 100 | 100 | 100 | 0 | 65 |
| Rv1765A | - | 71 | 77 | 0 | 56 | 0 | 75 | 0 | 74 | 62 | 0 | 0 | 0 | 0 | 0 | 0 | 57 | 57 | 98 | 60 | 64 | 56 | 56 | 60 | 100 | 100 | 100 | 0 | 54 |
| Rv1765c | - | 365 | 0 | 0 | 0 | 0 | 0 | 0 | 0 | 47 | 0 | 43 | 39 | 38 | 0 | 83 | 85 | 85 | 100 | 76 | 80 | 79 | 79 | 75 | 100 | 100 | 100 | 0 | 76 |
| Rv1766 | - | 89 | 66 | 64 | 78 | 75 | 0 | 0 | 0 | 79 | 66 | 89 | 0 | 88 | 87 | 77 | 93 | 77 | 100 | 92 | 89 | 89 | 89 | 92 | 100 | 100 | 100 | 93 | 92 |
| Rv1767 | - | 119 | 0 | 0 | 0 | 0 | 0 | 0 | 0 | 74 | 0 | 0 | 0 | 0 | 0 | 0 | 87 | 0 | 100 | 0 | 0 | 0 | 0 | 0 | 100 | 100 | 100 | 87 | 82 |
| Rv1768 | *PE_PGRS31* | 618 | 34 | 43 | 38 | 35 | 36 | 36 | 38 | 37 | 37 | 39 | 42 | 42 | 42 | 44 | 81 | 34 | 99 | 47 | 53 | 48 | 48 | 45 | 99 | 100 | 100 | 59 | 45 |
| Rv1769 | - | 414 | 56 | 0 | 0 | 0 | 56 | 50 | 57 | 57 | 58 | 58 | 56 | 58 | 57 | 0 | 88 | 0 | 100 | 0 | 0 | 0 | 0 | 0 | 99 | 100 | 100 | 88 | 0 |
| Rv1770 | - | 428 | 0 | 0 | 0 | 0 | 0 | 0 | 0 | 0 | 0 | 0 | 0 | 0 | 0 | 0 | 0 | 0 | 100 | 0 | 0 | 0 | 0 | 0 | 99 | 100 | 100 | 84 | 0 |

| | | | | | | | | | | | | | | | | | | | | | | | | | | | | | |
|---|---|---|---|---|---|---|---|---|---|---|---|---|---|---|---|---|---|---|---|---|---|---|---|---|---|---|---|---|---|
| Rv1771 | - | 428 | 52 | 0 | 0 | 0 | 53 | 0 | 0 | 0 | 64 | 60 | 60 | 61 | 60 | 0 | 87 | 0 | 100 | 0 | 0 | 0 | 0 | 0 | 100 | 100 | 100 | 86 | 0 |
| Rv1772 | - | 103 | 0 | 0 | 0 | 0 | 0 | 0 | 0 | 0 | 0 | 0 | 0 | 58 | 0 | 75 | 0 | 75 | 100 | 0 | 0 | 0 | 0 | 0 | 100 | 100 | 100 | 0 | 0 |
| Rv1773c | - | 248 | 0 | 0 | 49 | 44 | 42 | 41 | 0 | 46 | 47 | 45 | 49 | 48 | 46 | 44 | 47 | 47 | 100 | 46 | 45 | 45 | 45 | 48 | 100 | 100 | 100 | 43 | 75 |
| Rv1774 | - | 446 | 0 | 0 | 0 | 0 | 0 | 0 | 0 | 40 | 47 | 47 | 43 | 42 | 37 | 43 | 45 | 43 | 100 | 44 | 40 | 40 | 40 | 48 | 100 | 100 | 100 | 45 | 79 |
| Rv1775 | - | 272 | 0 | 0 | 0 | 0 | 0 | 0 | 0 | 0 | 0 | 0 | 0 | 0 | 46 | 0 | 0 | 0 | 100 | 72 | 0 | 0 | 0 | 41 | 100 | 100 | 100 | 0 | 73 |
| Rv1776c | - | 186 | 0 | 0 | 0 | 0 | 0 | 0 | 0 | 51 | 0 | 60 | 52 | 59 | 57 | 65 | 83 | 65 | 100 | 65 | 68 | 69 | 69 | 73 | 100 | 100 | 100 | 83 | 69 |
| Rv1777 | *cyp144* | 434 | 0 | 0 | 42 | 43 | 41 | 0 | 0 | 46 | 48 | 65 | 46 | 63 | 62 | 75 | 88 | 75 | 99 | 77 | 78 | 78 | 78 | 80 | 100 | 100 | 100 | 87 | 74 |
| Rv1778c | - | 149 | 0 | 0 | 0 | 0 | 0 | 0 | 0 | 0 | 0 | 0 | 0 | 0 | 0 | 0 | 93 | 0 | 99 | 0 | 0 | 0 | 0 | 0 | 100 | 100 | 100 | 92 | 0 |
| Rv1779c | - | 597 | 0 | 0 | 0 | 0 | 0 | 0 | 0 | 0 | 54 | 0 | 0 | 0 | 0 | 67 | 77 | 68 | 100 | 0 | 0 | 0 | 0 | 0 | 99 | 100 | 100 | 77 | 0 |
| Rv1780 | - | 187 | 0 | 0 | 0 | 0 | 0 | 0 | 0 | 0 | 51 | 0 | 0 | 0 | 79 | 94 | 94 | 94 | 100 | 84 | 83 | 83 | 83 | 84 | 100 | 100 | 100 | 95 | 84 |
| Rv1781c | *malQ* | 724 | 60 | 59 | 60 | 62 | 55 | 58 | 0 | 0 | 0 | 0 | 64 | 63 | 0 | 88 | 85 | 88 | 100 | 78 | 79 | 79 | 79 | 78 | 100 | 100 | 100 | 85 | 78 |
| Rv1782 | - | 506 | 39 | 46 | 39 | 56 | 48 | 36 | 47 | 0 | 54 | 54 | 0 | 55 | 54 | 93 | 92 | 93 | 99 | 56 | 54 | 54 | 54 | 54 | 100 | 100 | 100 | 92 | 55 |
| Rv1783 | - | 435 | 48 | 47 | 41 | 45 | 43 | 44 | 44 | 44 | 50 | 49 | 0 | 46 | 45 | 94 | 96 | 94 | 100 | 44 | 43 | 43 | 43 | 44 | 100 | 100 | 100 | 96 | 44 |
| Rv1784 | - | 932 | 46 | 46 | 47 | 46 | 43 | 47 | 43 | 51 | 52 | 51 | 0 | 52 | 52 | 94 | 96 | 94 | 100 | 48 | 50 | 50 | 50 | 50 | 100 | 100 | 100 | 96 | 48 |
| Rv1785c | *cyp143* | 393 | 0 | 0 | 42 | 42 | 0 | 0 | 0 | 48 | 49 | 50 | 53 | 53 | 44 | 83 | 85 | 84 | 100 | 76 | 82 | 73 | 73 | 47 | 100 | 100 | 100 | 85 | 74 |
| Rv1786 | - | 67 | 0 | 0 | 0 | 0 | 0 | 0 | 0 | 0 | 0 | 0 | 0 | 0 | 0 | 93 | 92 | 93 | 100 | 0 | 90 | 0 | 0 | 0 | 100 | 100 | 100 | 92 | 0 |
| Rv1787 | *PPE25* | 365 | 0 | 0 | 0 | 0 | 0 | 0 | 0 | 0 | 40 | 0 | 39 | 39 | 50 | 63 | 63 | 63 | 99 | 50 | 50 | 50 | 50 | 51 | 99 | 100 | 99 | 63 | 51 |
| Rv1788 | *PE18* | 99 | 0 | 0 | 0 | 0 | 0 | 0 | 0 | 0 | 0 | 0 | 0 | 0 | 0 | 91 | 93 | 93 | 100 | 0 | 0 | 0 | 0 | 0 | 100 | 100 | 100 | 94 | 0 |
| Rv1789 | *PPE26* | 393 | 0 | 0 | 0 | 0 | 0 | 0 | 0 | 0 | 36 | 41 | 36 | 55 | 44 | 70 | 74 | 70 | 100 | 44 | 43 | 43 | 43 | 44 | 100 | 100 | 100 | 67 | 44 |
| Rv1790 | *PPE27* | 350 | 0 | 0 | 0 | 0 | 0 | 0 | 0 | 0 | 38 | 0 | 36 | 39 | 39 | 59 | 60 | 59 | 99 | 51 | 51 | 51 | 51 | 51 | 100 | 100 | 100 | 59 | 52 |
| Rv1791 | *PE19* | 99 | 0 | 0 | 0 | 0 | 0 | 0 | 0 | 0 | 0 | 0 | 0 | 0 | 0 | 91 | 96 | 94 | 100 | 0 | 0 | 0 | 0 | 0 | 100 | 100 | 100 | 94 | 0 |
| Rv1793 | *esxN* | 94 | 0 | 0 | 0 | 0 | 0 | 0 | 0 | 0 | 0 | 0 | 0 | 0 | 0 | 97 | 100 | 97 | 100 | 0 | 0 | 0 | 0 | 0 | 100 | 100 | 100 | 100 | 0 |
| Rv1794 | - | 300 | 0 | 0 | 0 | 0 | 0 | 0 | 0 | 0 | 0 | 0 | 0 | 0 | 0 | 97 | 99 | 97 | 100 | 0 | 0 | 0 | 0 | 0 | 100 | 100 | 100 | 99 | 0 |
| Rv1795 | - | 503 | 0 | 0 | 0 | 0 | 0 | 0 | 0 | 0 | 43 | 43 | 0 | 40 | 42 | 88 | 92 | 89 | 99 | 41 | 41 | 41 | 41 | 39 | 100 | 100 | 100 | 92 | 41 |
| Rv1796 | *mycP5* | 585 | 46 | 68 | 39 | 43 | 0 | 0 | 0 | 38 | 51 | 50 | 31 | 56 | 52 | 82 | 90 | 81 | 100 | 54 | 54 | 54 | 54 | 54 | 100 | 100 | 100 | 89 | 54 |
| Rv1797 | - | 406 | 0 | 0 | 0 | 0 | 0 | 0 | 0 | 0 | 40 | 40 | 42 | 40 | 0 | 76 | 77 | 77 | 100 | 0 | 0 | 0 | 0 | 0 | 100 | 100 | 100 | 82 | 0 |
| Rv1798 | - | 610 | 0 | 0 | 0 | 0 | 0 | 0 | 0 | 0 | 53 | 0 | 0 | 0 | 47 | 94 | 98 | 94 | 100 | 52 | 48 | 48 | 48 | 50 | 100 | 100 | 100 | 97 | 51 |
| Rv1799 | *lppT* | 63 | 0 | 0 | 0 | 0 | 0 | 0 | 0 | 0 | 0 | 0 | 0 | 0 | 0 | 0 | 0 | 0 | 100 | 0 | 0 | 0 | 0 | 0 | 100 | 100 | 100 | 0 | 0 |
| Rv1800 | *PPE28* | 655 | 0 | 0 | 0 | 0 | 0 | 0 | 0 | 0 | 0 | 40 | 0 | 0 | 43 | 54 | 81 | 54 | 99 | 43 | 48 | 48 | 48 | 48 | 99 | 100 | 100 | 80 | 44 |
| Rv1801 | *PPE29* | 423 | 0 | 0 | 0 | 0 | 0 | 0 | 34 | 0 | 38 | 37 | 32 | 33 | 43 | 61 | 64 | 61 | 100 | 49 | 41 | 42 | 42 | 45 | 100 | 100 | 100 | 63 | 40 |
| Rv1802 | *PPE30* | 463 | 0 | 0 | 0 | 0 | 0 | 0 | 0 | 0 | 37 | 39 | 37 | 35 | 49 | 64 | 65 | 65 | 100 | 42 | 53 | 53 | 53 | 41 | 99 | 100 | 100 | 64 | 42 |
| Rv1803c | *PE_PGRS32* | 639 | 36 | 45 | 36 | 33 | 34 | 32 | 35 | 34 | 40 | 40 | 42 | 40 | 39 | 52 | 66 | 66 | 99 | 51 | 46 | 51 | 51 | 50 | 98 | 100 | 100 | 83 | 53 |
| Rv1804c | - | 108 | 0 | 0 | 0 | 0 | 0 | 0 | 0 | 0 | 0 | 0 | 0 | 0 | 0 | 71 | 71 | 71 | 100 | 0 | 0 | 0 | 0 | 0 | 100 | 100 | 100 | 57 | 0 |
| Rv1805c | - | 115 | 0 | 0 | 0 | 0 | 0 | 0 | 0 | 0 | 0 | 0 | 0 | 0 | 0 | 0 | 53 | 0 | 100 | 0 | 0 | 0 | 0 | 0 | 100 | 100 | 100 | 53 | 0 |
| Rv1806 | *PE20* | 99 | 0 | 0 | 0 | 0 | 0 | 0 | 0 | 0 | 0 | 0 | 0 | 0 | 0 | 72 | 74 | 73 | 100 | 0 | 0 | 0 | 0 | 0 | 100 | 100 | 100 | 74 | 0 |
| Rv1807 | *PPE31* | 399 | 0 | 0 | 0 | 0 | 0 | 0 | 38 | 0 | 42 | 42 | 38 | 39 | 39 | 60 | 61 | 61 | 99 | 40 | 40 | 40 | 40 | 41 | 99 | 100 | 99 | 77 | 39 |
| Rv1808 | *PPE32* | 409 | 0 | 0 | 0 | 0 | 0 | 0 | 37 | 0 | 39 | 38 | 37 | 36 | 40 | 73 | 71 | 73 | 99 | 47 | 40 | 40 | 40 | 41 | 100 | 100 | 100 | 71 | 41 |
| Rv1809 | *PPE33* | 468 | 0 | 0 | 0 | 0 | 0 | 0 | 0 | 0 | 0 | 0 | 0 | 0 | 53 | 75 | 62 | 75 | 99 | 44 | 42 | 42 | 42 | 43 | 100 | 100 | 100 | 61 | 41 |

| | | | | | | | | | | | | | | | | | | | | | | | | | | | | | |
|---|---|---|---|---|---|---|---|---|---|---|---|---|---|---|---|---|---|---|---|---|---|---|---|---|---|---|---|---|---|
| Rv1810 | - | 118 | 0 | 0 | 0 | 0 | 0 | 0 | 0 | 0 | 0 | 0 | 0 | 0 | 0 | 76 | 76 | 76 | 100 | 0 | 0 | 0 | 0 | 0 | 100 | 100 | 100 | 59 | 0 |
| Rv1811 | *mgtC* | 234 | 0 | 0 | 0 | 0 | 0 | 0 | 0 | 0 | 0 | 59 | 0 | 60 | 59 | 84 | 90 | 84 | 100 | 0 | 57 | 57 | 57 | 0 | 100 | 100 | 100 | 90 | 0 |
| Rv1812c | - | 400 | 42 | 41 | 43 | 43 | 40 | 41 | 41 | 44 | 43 | 42 | 79 | 57 | 46 | 42 | 83 | 42 | 99 | 39 | 45 | 45 | 45 | 43 | 99 | 100 | 99 | 83 | 45 |
| Rv1813c | - | 143 | 0 | 0 | 0 | 0 | 0 | 0 | 0 | 0 | 0 | 0 | 0 | 0 | 0 | 0 | 0 | 0 | 100 | 0 | 0 | 0 | 0 | 0 | 100 | 100 | 100 | 51 | 0 |
| Rv1814 | *erg3* | 300 | 0 | 0 | 0 | 0 | 0 | 0 | 0 | 0 | 62 | 79 | 83 | 80 | 0 | 88 | 88 | 89 | 100 | 86 | 87 | 87 | 87 | 87 | 100 | 100 | 100 | 88 | 86 |
| Rv1815 | - | 221 | 0 | 0 | 0 | 0 | 46 | 0 | 0 | 0 | 0 | 0 | 0 | 0 | 55 | 80 | 79 | 80 | 99 | 64 | 70 | 71 | 71 | 70 | 99 | 99 | 99 | 73 | 64 |
| Rv1816 | - | 234 | 0 | 0 | 0 | 0 | 0 | 0 | 0 | 53 | 58 | 62 | 62 | 61 | 48 | 87 | 0 | 87 | 100 | 74 | 71 | 73 | 73 | 73 | 100 | 100 | 100 | 0 | 76 |
| Rv1817 | - | 487 | 0 | 0 | 0 | 38 | 0 | 0 | 0 | 82 | 55 | 57 | 57 | 57 | 86 | 85 | 95 | 86 | 100 | 82 | 85 | 85 | 85 | 82 | 99 | 100 | 100 | 94 | 83 |
| Rv1818c | *PE_PGRS33* | 498 | 42 | 46 | 38 | 35 | 36 | 39 | 38 | 39 | 41 | 44 | 44 | 44 | 44 | 55 | 64 | 47 | 99 | 50 | 47 | 49 | 49 | 47 | 90 | 100 | 100 | 57 | 49 |
| Rv1819c | - | 639 | 47 | 47 | 48 | 41 | 42 | 46 | 47 | 41 | 65 | 46 | 66 | 65 | 67 | 87 | 92 | 87 | 99 | 75 | 76 | 76 | 76 | 77 | 100 | 100 | 100 | 92 | 74 |
| Rv1820 | *ilvG* | 547 | 43 | 41 | 41 | 41 | 41 | 42 | 41 | 64 | 42 | 41 | 42 | 42 | 43 | 89 | 90 | 89 | 100 | 44 | 48 | 48 | 48 | 48 | 100 | 100 | 100 | 90 | 43 |
| Rv1821 | *secA2* | 808 | 68 | 67 | 68 | 67 | 70 | 67 | 68 | 52 | 54 | 53 | 53 | 49 | 86 | 94 | 92 | 94 | 100 | 89 | 91 | 91 | 91 | 90 | 100 | 100 | 100 | 92 | 90 |
| Rv1822 | *pgsA2* | 209 | 0 | 52 | 50 | 49 | 0 | 59 | 0 | 69 | 64 | 64 | 64 | 64 | 76 | 84 | 84 | 84 | 100 | 77 | 80 | 80 | 80 | 82 | 100 | 100 | 100 | 84 | 75 |
| Rv1823 | - | 307 | 0 | 0 | 0 | 0 | 0 | 0 | 0 | 44 | 0 | 55 | 60 | 59 | 71 | 84 | 89 | 84 | 100 | 75 | 74 | 74 | 74 | 71 | 100 | 100 | 100 | 88 | 75 |
| Rv1824 | - | 121 | 0 | 0 | 0 | 0 | 0 | 0 | 0 | 81 | 0 | 93 | 92 | 92 | 93 | 97 | 91 | 97 | 100 | 93 | 93 | 93 | 93 | 94 | 100 | 100 | 100 | 91 | 92 |
| Rv1825 | - | 292 | 0 | 0 | 0 | 0 | 0 | 0 | 0 | 53 | 0 | 70 | 64 | 64 | 80 | 80 | 86 | 80 | 100 | 75 | 76 | 76 | 76 | 72 | 100 | 100 | 99 | 86 | 79 |
| Rv1826 | *gcvH* | 134 | 69 | 0 | 0 | 0 | 67 | 65 | 68 | 71 | 71 | 78 | 80 | 80 | 82 | 92 | 91 | 92 | 99 | 83 | 85 | 85 | 85 | 83 | 100 | 100 | 100 | 91 | 83 |
| Rv1827 | *cfp17* | 162 | 82 | 80 | 80 | 81 | 86 | 86 | 86 | 66 | 80 | 87 | 86 | 86 | 88 | 94 | 95 | 94 | 100 | 87 | 95 | 95 | 95 | 88 | 100 | 100 | 100 | 94 | 88 |
| Rv1828 | - | 247 | 61 | 63 | 61 | 61 | 64 | 60 | 64 | 63 | 67 | 70 | 73 | 72 | 79 | 91 | 93 | 92 | 100 | 83 | 82 | 82 | 82 | 83 | 100 | 100 | 100 | 92 | 87 |
| Rv1829 | - | 164 | 0 | 55 | 48 | 47 | 0 | 52 | 0 | 73 | 85 | 86 | 87 | 87 | 95 | 97 | 98 | 97 | 99 | 96 | 97 | 97 | 97 | 96 | 100 | 100 | 100 | 98 | 95 |
| Rv1830 | - | 225 | 76 | 77 | 76 | 76 | 75 | 67 | 71 | 72 | 89 | 82 | 85 | 85 | 88 | 91 | 91 | 91 | 100 | 89 | 89 | 89 | 89 | 89 | 100 | 100 | 100 | 92 | 89 |
| Rv1831 | - | 85 | 0 | 0 | 0 | 0 | 0 | 0 | 0 | 0 | 0 | 0 | 0 | 0 | 0 | 60 | 86 | 60 | 100 | 0 | 0 | 0 | 0 | 0 | 100 | 100 | 100 | 86 | 0 |
| Rv1832 | *gcvB* | 941 | 67 | 0 | 0 | 0 | 66 | 66 | 67 | 73 | 85 | 85 | 85 | 85 | 87 | 93 | 93 | 93 | 99 | 89 | 88 | 88 | 88 | 90 | 100 | 100 | 100 | 93 | 88 |
| Rv1833c | - | 286 | 0 | 0 | 0 | 0 | 0 | 0 | 0 | 50 | 71 | 51 | 43 | 41 | 48 | 46 | 89 | 46 | 100 | 43 | 45 | 45 | 45 | 43 | 100 | 100 | 100 | 88 | 43 |
| Rv1834 | - | 288 | 0 | 0 | 0 | 0 | 0 | 0 | 0 | 38 | 0 | 0 | 0 | 0 | 50 | 43 | 91 | 43 | 99 | 41 | 0 | 0 | 0 | 51 | 100 | 100 | 100 | 89 | 42 |
| Rv1835c | - | 628 | 0 | 0 | 0 | 0 | 0 | 0 | 0 | 42 | 36 | 38 | 37 | 38 | 37 | 37 | 37 | 35 | 100 | 0 | 76 | 76 | 76 | 38 | 98 | 100 | 100 | 45 | 46 |
| Rv1836c | - | 677 | 0 | 0 | 0 | 0 | 0 | 0 | 0 | 0 | 49 | 46 | 51 | 51 | 61 | 79 | 77 | 79 | 99 | 67 | 67 | 67 | 67 | 66 | 100 | 100 | 100 | 77 | 68 |
| Rv1837c | *glcB* | 741 | 0 | 0 | 73 | 73 | 74 | 74 | 75 | 0 | 80 | 81 | 80 | 80 | 85 | 89 | 90 | 89 | 100 | 85 | 87 | 87 | 87 | 86 | 100 | 100 | 100 | 90 | 84 |
| Rv1838c | - | 131 | 0 | 0 | 0 | 0 | 0 | 0 | 0 | 0 | 0 | 0 | 0 | 0 | 0 | 0 | 0 | 0 | 100 | 0 | 0 | 0 | 0 | 0 | 100 | 100 | 100 | 0 | 0 |
| Rv1839c | - | 87 | 0 | 0 | 0 | 0 | 0 | 0 | 0 | 0 | 0 | 0 | 0 | 0 | 0 | 0 | 0 | 0 | 100 | 0 | 0 | 0 | 0 | 0 | 100 | 100 | 100 | 0 | 0 |
| Rv1840c | *PE_PGRS34* | 515 | 36 | 42 | 36 | 36 | 37 | 35 | 35 | 39 | 39 | 39 | 41 | 42 | 41 | 55 | 69 | 61 | 88 | 47 | 47 | 47 | 47 | 46 | 100 | 100 | 100 | 69 | 44 |
| Rv1841c | - | 345 | 53 | 64 | 62 | 64 | 64 | 66 | 63 | 59 | 71 | 72 | 72 | 72 | 79 | 85 | 85 | 85 | 98 | 82 | 82 | 82 | 82 | 78 | 99 | 100 | 100 | 84 | 83 |
| Rv1842c | - | 455 | 54 | 67 | 67 | 68 | 65 | 68 | 64 | 61 | 73 | 73 | 73 | 73 | 80 | 88 | 90 | 88 | 99 | 78 | 83 | 83 | 83 | 80 | 99 | 100 | 100 | 89 | 79 |
| Rv1843c | *guaB1* | 479 | 52 | 51 | 74 | 75 | 51 | 74 | 51 | 78 | 85 | 86 | 84 | 84 | 85 | 94 | 95 | 94 | 100 | 89 | 91 | 91 | 91 | 92 | 100 | 100 | 100 | 95 | 92 |
| Rv1844c | *gnd1* | 485 | 81 | 80 | 82 | 82 | 80 | 81 | 80 | 50 | 88 | 87 | 87 | 87 | 90 | 91 | 95 | 91 | 99 | 88 | 90 | 90 | 90 | 90 | 99 | 100 | 99 | 94 | 90 |
| Rv1845c | - | 316 | 0 | 0 | 41 | 0 | 0 | 0 | 0 | 0 | 78 | 80 | 80 | 80 | 79 | 93 | 91 | 93 | 100 | 84 | 83 | 83 | 83 | 82 | 100 | 100 | 100 | 91 | 85 |
| Rv1846c | - | 138 | 0 | 0 | 58 | 57 | 0 | 0 | 0 | 0 | 88 | 86 | 90 | 90 | 89 | 92 | 92 | 92 | 99 | 89 | 89 | 89 | 89 | 87 | 100 | 100 | 100 | 91 | 89 |
| Rv1847 | - | 140 | 62 | 58 | 58 | 54 | 58 | 62 | 60 | 0 | 75 | 71 | 70 | 70 | 77 | 85 | 82 | 85 | 100 | 81 | 82 | 82 | 82 | 81 | 100 | 100 | 100 | 82 | 83 |

| | | | | | | | | | | | | | | | | | | | | | | | | | | | | | |
|---|---|---|---|---|---|---|---|---|---|---|---|---|---|---|---|---|---|---|---|---|---|---|---|---|---|---|---|---|---|
| Rv1848 | *ureA* | 100 | 0 | 0 | 84 | 84 | 0 | 81 | 85 | 0 | 95 | 81 | 92 | 92 | 94 | 0 | 96 | 0 | 100 | 94 | 95 | 95 | 95 | 95 | 100 | 100 | 100 | 96 | 93 |
| Rv1849 | *ureB* | 104 | 0 | 0 | 56 | 64 | 0 | 59 | 58 | 0 | 72 | 71 | 75 | 76 | 79 | 0 | 82 | 0 | 100 | 81 | 83 | 83 | 83 | 82 | 100 | 100 | 100 | 82 | 83 |
| Rv1850 | *ureC* | 577 | 0 | 0 | 74 | 73 | 0 | 67 | 73 | 0 | 89 | 75 | 90 | 89 | 88 | 0 | 0 | 0 | 100 | 89 | 89 | 89 | 89 | 89 | 100 | 100 | 100 | 93 | 88 |
| Rv1851 | *ureF* | 211 | 0 | 0 | 0 | 0 | 0 | 0 | 0 | 0 | 71 | 48 | 75 | 75 | 73 | 0 | 86 | 0 | 100 | 80 | 75 | 74 | 74 | 80 | 100 | 100 | 100 | 86 | 78 |
| Rv1852 | *ureG* | 224 | 0 | 0 | 68 | 67 | 0 | 64 | 70 | 0 | 89 | 69 | 88 | 89 | 91 | 0 | 92 | 0 | 100 | 88 | 88 | 88 | 88 | 89 | 100 | 100 | 100 | 92 | 89 |
| Rv1853 | *ureD* | 208 | 0 | 0 | 0 | 0 | 0 | 0 | 0 | 0 | 60 | 0 | 59 | 60 | 65 | 0 | 82 | 0 | 100 | 73 | 70 | 70 | 70 | 74 | 100 | 100 | 100 | 81 | 73 |
| Rv1854c | *ndh* | 463 | 71 | 73 | 72 | 71 | 71 | 72 | 71 | 75 | 78 | 79 | 78 | 79 | 86 | 94 | 94 | 94 | 99 | 89 | 91 | 91 | 91 | 90 | 100 | 100 | 100 | 95 | 90 |
| Rv1855c | - | 307 | 0 | 0 | 0 | 0 | 0 | 0 | 0 | 59 | 57 | 75 | 75 | 84 | 76 | 84 | 91 | 84 | 100 | 81 | 80 | 80 | 80 | 80 | 100 | 100 | 100 | 91 | 79 |
| Rv1856c | - | 225 | 0 | 0 | 0 | 0 | 0 | 0 | 0 | 0 | 0 | 47 | 0 | 43 | 68 | 91 | 91 | 91 | 99 | 76 | 80 | 80 | 80 | 80 | 100 | 100 | 100 | 90 | 77 |
| Rv1857 | *modA* | 261 | 51 | 52 | 49 | 51 | 56 | 0 | 47 | 62 | 55 | 67 | 68 | 66 | 64 | 76 | 75 | 75 | 100 | 66 | 68 | 67 | 67 | 72 | 100 | 100 | 100 | 80 | 66 |
| Rv1858 | *modB* | 264 | 61 | 63 | 62 | 62 | 74 | 0 | 59 | 84 | 67 | 79 | 83 | 83 | 83 | 92 | 91 | 92 | 100 | 82 | 93 | 93 | 93 | 84 | 100 | 100 | 100 | 90 | 82 |
| Rv1859 | *modC* | 369 | 53 | 50 | 51 | 52 | 57 | 51 | 52 | 68 | 58 | 64 | 65 | 67 | 64 | 83 | 84 | 83 | 99 | 69 | 73 | 73 | 73 | 69 | 100 | 100 | 100 | 84 | 69 |
| Rv1860 | *apa* | 325 | 48 | 45 | 36 | 0 | 35 | 0 | 37 | 0 | 38 | 0 | 37 | 35 | 53 | 66 | 63 | 63 | 99 | 61 | 64 | 64 | 64 | 60 | 99 | 100 | 99 | 75 | 59 |
| Rv1861 | - | 101 | 59 | 71 | 72 | 70 | 67 | 73 | 70 | 0 | 77 | 66 | 71 | 72 | 67 | 75 | 73 | 78 | 100 | 73 | 0 | 0 | 0 | 74 | 100 | 100 | 100 | 71 | 74 |
| Rv1862 | *adhA* | 346 | 44 | 46 | 44 | 47 | 43 | 43 | 44 | 49 | 76 | 75 | 77 | 78 | 41 | 89 | 91 | 89 | 100 | 85 | 84 | 84 | 84 | 82 | 100 | 100 | 100 | 90 | 85 |
| Rv1863c | - | 256 | 0 | 0 | 64 | 0 | 0 | 0 | 0 | 0 | 83 | 82 | 83 | 83 | 80 | 93 | 93 | 93 | 100 | 87 | 89 | 89 | 89 | 91 | 100 | 100 | 100 | 95 | 89 |
| Rv1864c | - | 251 | 63 | 0 | 63 | 64 | 0 | 0 | 61 | 59 | 55 | 61 | 60 | 49 | 69 | 0 | 88 | 0 | 100 | 70 | 76 | 76 | 76 | 74 | 100 | 100 | 100 | 87 | 69 |
| Rv1865c | - | 286 | 47 | 45 | 49 | 48 | 49 | 50 | 55 | 56 | 75 | 75 | 77 | 58 | 59 | 86 | 89 | 86 | 100 | 57 | 57 | 57 | 57 | 58 | 100 | 100 | 100 | 89 | 59 |
| Rv1866 | - | 778 | 0 | 0 | 44 | 0 | 43 | 0 | 0 | 46 | 47 | 45 | 61 | 61 | 47 | 84 | 84 | 84 | 100 | 54 | 54 | 54 | 54 | 54 | 100 | 100 | 100 | 86 | 55 |
| Rv1867 | - | 494 | 0 | 0 | 0 | 0 | 0 | 0 | 0 | 0 | 53 | 47 | 72 | 72 | 68 | 88 | 88 | 88 | 100 | 82 | 80 | 80 | 80 | 80 | 100 | 100 | 100 | 87 | 80 |
| Rv1868 | - | 699 | 0 | 0 | 0 | 0 | 0 | 0 | 0 | 0 | 0 | 0 | 0 | 0 | 0 | 80 | 87 | 80 | 100 | 0 | 0 | 0 | 0 | 0 | 100 | 100 | 100 | 86 | 0 |
| Rv1869c | - | 411 | 49 | 50 | 53 | 71 | 49 | 48 | 50 | 61 | 74 | 72 | 77 | 76 | 56 | 86 | 61 | 86 | 100 | 60 | 59 | 59 | 59 | 85 | 100 | 100 | 100 | 60 | 55 |
| Rv1870c | - | 211 | 0 | 0 | 0 | 0 | 0 | 0 | 0 | 0 | 0 | 0 | 0 | 69 | 0 | 80 | 0 | 80 | 100 | 77 | 77 | 77 | 77 | 0 | 100 | 100 | 100 | 0 | 0 |
| Rv1871c | - | 129 | 0 | 0 | 0 | 0 | 0 | 0 | 0 | 0 | 0 | 50 | 73 | 73 | 60 | 90 | 91 | 90 | 100 | 72 | 74 | 74 | 74 | 0 | 100 | 100 | 100 | 91 | 74 |
| Rv1872c | *lldD2* | 414 | 68 | 0 | 75 | 75 | 76 | 76 | 0 | 81 | 85 | 54 | 71 | 54 | 52 | 91 | 92 | 91 | 99 | 51 | 51 | 51 | 51 | 51 | 100 | 100 | 100 | 91 | 51 |
| Rv1873 | - | 145 | 0 | 0 | 0 | 0 | 0 | 0 | 0 | 0 | 0 | 0 | 0 | 0 | 0 | 83 | 0 | 82 | 100 | 66 | 77 | 77 | 77 | 74 | 100 | 100 | 100 | 0 | 71 |
| Rv1874 | - | 228 | 0 | 0 | 0 | 0 | 0 | 0 | 0 | 56 | 58 | 0 | 0 | 0 | 60 | 85 | 84 | 85 | 100 | 76 | 77 | 78 | 78 | 72 | 100 | 100 | 100 | 83 | 75 |
| Rv1875 | - | 147 | 0 | 0 | 0 | 0 | 0 | 0 | 0 | 0 | 0 | 0 | 0 | 80 | 72 | 91 | 91 | 91 | 100 | 81 | 69 | 0 | 0 | 51 | 100 | 100 | 100 | 87 | 80 |
| Rv1876 | *bfrA* | 159 | 0 | 0 | 0 | 0 | 0 | 0 | 0 | 0 | 0 | 0 | 84 | 84 | 0 | 95 | 97 | 95 | 100 | 90 | 94 | 94 | 94 | 94 | 100 | 100 | 100 | 95 | 93 |
| Rv1877 | - | 687 | 56 | 59 | 57 | 57 | 58 | 55 | 60 | 58 | 63 | 62 | 53 | 53 | 69 | 74 | 75 | 74 | 100 | 71 | 73 | 73 | 73 | 72 | 100 | 100 | 100 | 74 | 72 |
| Rv1878 | *glnA3* | 450 | 47 | 41 | 42 | 42 | 45 | 45 | 43 | 41 | 41 | 62 | 63 | 62 | 42 | 88 | 86 | 88 | 99 | 77 | 81 | 81 | 81 | 78 | 100 | 100 | 99 | 81 | 81 |
| Rv1879 | - | 378 | 0 | 0 | 0 | 0 | 0 | 0 | 0 | 0 | 0 | 0 | 0 | 0 | 0 | 83 | 84 | 82 | 100 | 78 | 61 | 61 | 61 | 62 | 100 | 100 | 100 | 84 | 60 |
| Rv1880c | *cyp140* | 438 | 0 | 0 | 44 | 45 | 49 | 0 | 0 | 49 | 60 | 48 | 50 | 47 | 57 | 85 | 90 | 86 | 100 | 79 | 77 | 77 | 77 | 77 | 100 | 100 | 100 | 89 | 80 |
| Rv1881c | *lppE* | 140 | 0 | 0 | 0 | 0 | 0 | 0 | 0 | 0 | 0 | 0 | 0 | 0 | 0 | 76 | 79 | 76 | 100 | 0 | 52 | 52 | 52 | 0 | 100 | 100 | 100 | 79 | 45 |
| Rv1882c | - | 277 | 46 | 45 | 51 | 52 | 47 | 49 | 44 | 52 | 50 | 61 | 54 | 50 | 56 | 89 | 89 | 89 | 100 | 64 | 81 | 80 | 80 | 44 | 100 | 100 | 100 | 88 | 62 |
| Rv1883c | - | 153 | 0 | 0 | 0 | 52 | 0 | 0 | 0 | 62 | 45 | 45 | 48 | 48 | 69 | 83 | 83 | 83 | 96 | 51 | 76 | 78 | 78 | 0 | 96 | 100 | 96 | 87 | 0 |
| Rv1884c | *rpfC* | 176 | 57 | 61 | 58 | 61 | 58 | 58 | 64 | 67 | 52 | 59 | 58 | 69 | 79 | 76 | 73 | 76 | 100 | 68 | 61 | 61 | 61 | 66 | 100 | 100 | 99 | 74 | 64 |
| Rv1885c | - | 199 | 0 | 0 | 0 | 0 | 0 | 0 | 0 | 0 | 0 | 0 | 46 | 47 | 0 | 76 | 76 | 76 | 100 | 60 | 61 | 62 | 62 | 63 | 100 | 100 | 100 | 76 | 64 |

| | | | | | | | | | | | | | | | | | | | | | | | | | | | | |
|---|---|---|---|---|---|---|---|---|---|---|---|---|---|---|---|---|---|---|---|---|---|---|---|---|---|---|---|---|---|
| Rv1886c | *fbpB* | 325 | 49 | 49 | 50 | 48 | 52 | 47 | 51 | 0 | 52 | 55 | 53 | 53 | 72 | 92 | 92 | 92 | 100 | 82 | 80 | 80 | 80 | 82 | 100 | 100 | 100 | 94 | 83 |
| Rv1887 | - | 380 | 0 | 0 | 0 | 0 | 0 | 0 | 0 | 0 | 0 | 0 | 0 | 0 | 44 | 68 | 68 | 68 | 100 | 50 | 45 | 46 | 46 | 45 | 100 | 100 | 100 | 77 | 51 |
| Rv1888A | - | 57 | 0 | 0 | 0 | 0 | 0 | 0 | 0 | 0 | 66 | 71 | 69 | 0 | 78 | 83 | 84 | 83 | 100 | 91 | 82 | 82 | 82 | 85 | 100 | 100 | 100 | 84 | 87 |
| Rv1888c | - | 186 | 0 | 0 | 0 | 0 | 0 | 0 | 0 | 0 | 0 | 0 | 73 | 0 | 0 | 59 | 86 | 60 | 100 | 0 | 0 | 0 | 0 | 0 | 100 | 100 | 100 | 85 | 0 |
| Rv1889c | - | 118 | 0 | 0 | 0 | 0 | 0 | 0 | 0 | 0 | 0 | 56 | 57 | 0 | 74 | 85 | 85 | 85 | 100 | 81 | 82 | 82 | 82 | 81 | 100 | 100 | 100 | 84 | 77 |
| Rv1890c | - | 203 | 0 | 0 | 0 | 0 | 0 | 0 | 0 | 0 | 0 | 0 | 0 | 0 | 60 | 83 | 84 | 82 | 100 | 72 | 0 | 0 | 0 | 66 | 100 | 100 | 100 | 84 | 78 |
| Rv1891 | - | 135 | 0 | 0 | 0 | 0 | 0 | 0 | 0 | 0 | 0 | 0 | 0 | 0 | 0 | 84 | 86 | 84 | 100 | 70 | 67 | 67 | 67 | 70 | 100 | 100 | 100 | 85 | 67 |
| Rv1892 | - | 103 | 0 | 0 | 0 | 0 | 0 | 0 | 0 | 0 | 0 | 0 | 0 | 0 | 0 | 91 | 91 | 91 | 100 | 81 | 88 | 87 | 87 | 87 | 100 | 100 | 100 | 88 | 80 |
| Rv1893 | - | 72 | 0 | 0 | 0 | 0 | 0 | 0 | 0 | 0 | 0 | 0 | 0 | 0 | 0 | 91 | 91 | 91 | 100 | 0 | 0 | 0 | 0 | 0 | 100 | 100 | 100 | 87 | 0 |
| Rv1894c | - | 376 | 41 | 0 | 40 | 0 | 49 | 44 | 44 | 62 | 73 | 59 | 62 | 62 | 93 | 95 | 95 | 95 | 100 | 93 | 94 | 94 | 94 | 95 | 100 | 100 | 100 | 96 | 94 |
| Rv1895 | - | 384 | 44 | 42 | 45 | 44 | 43 | 42 | 42 | 51 | 54 | 50 | 46 | 45 | 45 | 84 | 88 | 84 | 97 | 77 | 45 | 45 | 45 | 50 | 100 | 100 | 100 | 87 | 78 |
| Rv1896c | - | 303 | 0 | 0 | 0 | 0 | 0 | 0 | 0 | 0 | 45 | 65 | 66 | 0 | 63 | 85 | 85 | 85 | 100 | 57 | 56 | 56 | 56 | 57 | 100 | 100 | 100 | 81 | 58 |
| Rv1897c | - | 143 | 61 | 58 | 68 | 69 | 57 | 64 | 51 | 72 | 83 | 0 | 77 | 77 | 89 | 90 | 91 | 90 | 100 | 85 | 82 | 82 | 82 | 85 | 100 | 100 | 99 | 91 | 84 |
| Rv1898 | - | 102 | 72 | 72 | 70 | 69 | 0 | 67 | 0 | 72 | 80 | 0 | 73 | 65 | 0 | 93 | 91 | 93 | 100 | 73 | 0 | 0 | 0 | 73 | 100 | 100 | 100 | 90 | 75 |
| Rv1899c | *lppD* | 343 | 0 | 0 | 0 | 0 | 0 | 0 | 0 | 54 | 40 | 53 | 47 | 55 | 0 | 0 | 92 | 0 | 95 | 53 | 0 | 0 | 0 | 0 | 95 | 100 | 95 | 92 | 0 |
| Rv1900c | *lipJ* | 462 | 0 | 0 | 0 | 0 | 0 | 0 | 0 | 0 | 40 | 0 | 53 | 53 | 0 | 50 | 85 | 85 | 100 | 49 | 57 | 57 | 57 | 50 | 100 | 100 | 100 | 82 | 49 |
| Rv1901 | *cinA* | 430 | 45 | 60 | 53 | 59 | 54 | 56 | 54 | 53 | 52 | 52 | 56 | 54 | 71 | 88 | 90 | 88 | 100 | 84 | 84 | 84 | 84 | 83 | 100 | 100 | 100 | 89 | 83 |
| Rv1902c | *nanT* | 422 | 41 | 38 | 40 | 41 | 41 | 40 | 42 | 50 | 45 | 43 | 45 | 45 | 46 | 94 | 93 | 94 | 100 | 42 | 42 | 42 | 42 | 42 | 100 | 100 | 100 | 93 | 42 |
| Rv1903 | - | 134 | 48 | 0 | 58 | 60 | 0 | 0 | 60 | 59 | 66 | 70 | 69 | 70 | 0 | 92 | 88 | 92 | 100 | 87 | 84 | 85 | 85 | 90 | 100 | 100 | 100 | 87 | 90 |
| Rv1904 | - | 143 | 0 | 0 | 0 | 0 | 0 | 0 | 0 | 0 | 0 | 0 | 0 | 0 | 0 | 74 | 89 | 74 | 99 | 0 | 0 | 0 | 0 | 0 | 100 | 100 | 100 | 89 | 0 |
| Rv1905c | *aao* | 320 | 0 | 0 | 0 | 0 | 0 | 0 | 0 | 52 | 0 | 0 | 0 | 0 | 0 | 77 | 79 | 77 | 99 | 0 | 0 | 0 | 0 | 0 | 100 | 100 | 100 | 79 | 0 |
| Rv1906c | - | 156 | 0 | 0 | 0 | 0 | 0 | 0 | 0 | 0 | 0 | 0 | 0 | 0 | 0 | 60 | 79 | 60 | 100 | 52 | 56 | 56 | 56 | 52 | 100 | 100 | 100 | 77 | 52 |
| Rv1907c | - | 215 | 0 | 0 | 0 | 0 | 0 | 0 | 0 | 0 | 0 | 0 | 0 | 0 | 0 | 0 | 0 | 0 | 100 | 49 | 64 | 63 | 63 | 0 | 100 | 100 | 100 | 0 | 0 |
| Rv1908c | *katG* | 740 | 0 | 0 | 0 | 0 | 0 | 0 | 0 | 78 | 82 | 83 | 85 | 81 | 83 | 79 | 81 | 81 | 99 | 77 | 77 | 77 | 77 | 81 | 100 | 100 | 100 | 82 | 78 |
| Rv1909c | *furA* | 150 | 0 | 0 | 0 | 0 | 0 | 0 | 0 | 85 | 81 | 86 | 89 | 89 | 91 | 88 | 83 | 88 | 100 | 86 | 86 | 86 | 86 | 81 | 100 | 100 | 100 | 83 | 87 |
| Rv1910c | - | 197 | 48 | 0 | 0 | 0 | 46 | 0 | 0 | 45 | 0 | 0 | 0 | 0 | 0 | 68 | 47 | 69 | 100 | 40 | 0 | 0 | 0 | 44 | 100 | 100 | 100 | 47 | 0 |
| Rv1911c | *lppC* | 201 | 44 | 45 | 0 | 45 | 45 | 45 | 42 | 44 | 0 | 44 | 0 | 0 | 40 | 66 | 43 | 66 | 100 | 41 | 41 | 41 | 41 | 0 | 100 | 100 | 100 | 43 | 41 |
| Rv1912c | *fadB5* | 334 | 47 | 44 | 45 | 51 | 42 | 42 | 42 | 49 | 46 | 47 | 47 | 48 | 49 | 46 | 94 | 46 | 99 | 48 | 49 | 49 | 49 | 46 | 100 | 100 | 100 | 93 | 47 |
| Rv1913 | - | 250 | 0 | 0 | 0 | 0 | 0 | 0 | 0 | 48 | 0 | 0 | 0 | 0 | 0 | 84 | 80 | 83 | 100 | 65 | 0 | 0 | 0 | 0 | 100 | 100 | 100 | 80 | 67 |
| Rv1914c | - | 135 | 0 | 0 | 0 | 0 | 0 | 0 | 0 | 0 | 0 | 0 | 0 | 0 | 0 | 0 | 80 | 0 | 100 | 0 | 0 | 0 | 0 | 0 | 100 | 100 | 100 | 83 | 0 |
| Rv1915 | *aceAa* | 367 | 0 | 0 | 51 | 52 | 52 | 51 | 52 | 0 | 52 | 52 | 52 | 52 | 51 | 91 | 95 | 91 | 99 | 88 | 90 | 86 | 86 | 86 | 99 | 100 | 100 | 94 | 77 |
| Rv1916 | *aceAb* | 398 | 0 | 0 | 56 | 56 | 64 | 64 | 57 | 0 | 57 | 51 | 56 | 56 | 55 | 94 | 94 | 94 | 100 | 89 | 88 | 88 | 88 | 87 | 100 | 100 | 100 | 93 | 89 |
| Rv1917c | *PPE34* | 1459 | 0 | 0 | 0 | 0 | 0 | 0 | 0 | 0 | 41 | 39 | 36 | 36 | 48 | 62 | 47 | 50 | 67 | 50 | 45 | 44 | 44 | 49 | 87 | 100 | 91 | 48 | 48 |
| Rv1918c | *PPE35* | 987 | 0 | 0 | 0 | 0 | 0 | 0 | 0 | 0 | 39 | 39 | 38 | 39 | 50 | 70 | 70 | 70 | 73 | 46 | 52 | 47 | 47 | 47 | 100 | 100 | 100 | 78 | 46 |
| Rv1919c | - | 154 | 0 | 0 | 0 | 0 | 0 | 0 | 0 | 0 | 0 | 0 | 0 | 0 | 0 | 88 | 92 | 88 | 100 | 0 | 0 | 0 | 0 | 0 | 100 | 100 | 100 | 92 | 0 |
| Rv1920 | - | 287 | 0 | 0 | 0 | 0 | 0 | 0 | 0 | 49 | 60 | 45 | 44 | 44 | 45 | 54 | 88 | 53 | 100 | 47 | 52 | 52 | 52 | 44 | 100 | 100 | 100 | 86 | 53 |
| Rv1921c | *lppF* | 423 | 0 | 0 | 0 | 0 | 0 | 0 | 0 | 0 | 0 | 64 | 0 | 0 | 73 | 0 | 87 | 0 | 100 | 0 | 0 | 0 | 0 | 0 | 100 | 100 | 100 | 87 | 0 |
| Rv1922 | - | 371 | 46 | 0 | 45 | 0 | 0 | 0 | 0 | 0 | 41 | 43 | 47 | 46 | 45 | 46 | 78 | 46 | 100 | 48 | 40 | 44 | 44 | 45 | 100 | 100 | 100 | 80 | 45 |

| | | | | | | | | | | | | | | | | | | | | | | | | | | | | | |
|---|---|---|---|---|---|---|---|---|---|---|---|---|---|---|---|---|---|---|---|---|---|---|---|---|---|---|---|---|---|
| Rv1923 | *lipD* | 446 | 0 | 0 | 0 | 0 | 0 | 0 | 0 | 0 | 62 | 54 | 53 | 51 | 63 | 74 | 92 | 74 | 99 | 74 | 78 | 78 | 78 | 74 | 100 | 100 | 99 | 92 | 75 |
| Rv1924c | - | 126 | 0 | 0 | 0 | 0 | 0 | 0 | 0 | 0 | 0 | 0 | 0 | 0 | 0 | 0 | 76 | 0 | 100 | 0 | 0 | 0 | 0 | 0 | 100 | 100 | 100 | 75 | 0 |
| Rv1925 | *fadD31* | 620 | 55 | 56 | 57 | 56 | 53 | 0 | 54 | 37 | 69 | 69 | 70 | 70 | 76 | 83 | 87 | 83 | 100 | 70 | 79 | 79 | 79 | 81 | 100 | 100 | 100 | 86 | 78 |
| Rv1926c | *mpt63* | 159 | 0 | 0 | 0 | 0 | 0 | 0 | 0 | 0 | 0 | 0 | 0 | 0 | 59 | 58 | 59 | 59 | 100 | 55 | 63 | 63 | 63 | 65 | 100 | 100 | 100 | 0 | 56 |
| Rv1927 | - | 257 | 0 | 0 | 0 | 0 | 0 | 0 | 0 | 0 | 0 | 0 | 0 | 0 | 0 | 0 | 0 | 0 | 100 | 0 | 0 | 0 | 0 | 0 | 100 | 100 | 100 | 83 | 79 |
| Rv1928c | - | 255 | 51 | 51 | 52 | 53 | 50 | 52 | 50 | 54 | 59 | 57 | 59 | 59 | 54 | 87 | 89 | 87 | 100 | 57 | 58 | 58 | 58 | 56 | 100 | 100 | 100 | 87 | 58 |
| Rv1929c | - | 214 | 48 | 46 | 43 | 0 | 50 | 0 | 0 | 49 | 53 | 49 | 51 | 52 | 0 | 50 | 0 | 50 | 100 | 49 | 47 | 48 | 48 | 52 | 100 | 94 | 100 | 0 | 50 |
| Rv1930c | - | 174 | 0 | 0 | 0 | 0 | 0 | 0 | 0 | 0 | 57 | 0 | 0 | 0 | 75 | 86 | 93 | 86 | 100 | 74 | 72 | 72 | 72 | 75 | 100 | 100 | 100 | 92 | 78 |
| Rv1931c | - | 259 | 0 | 0 | 46 | 0 | 0 | 0 | 0 | 40 | 50 | 45 | 44 | 41 | 54 | 59 | 60 | 60 | 100 | 58 | 56 | 56 | 56 | 58 | 100 | 100 | 100 | 58 | 56 |
| Rv1932 | *tpx* | 165 | 64 | 65 | 67 | 71 | 0 | 0 | 0 | 0 | 0 | 0 | 0 | 0 | 88 | 91 | 92 | 92 | 100 | 84 | 0 | 0 | 0 | 91 | 100 | 100 | 100 | 92 | 88 |
| Rv1933c | *fadE18* | 363 | 0 | 0 | 0 | 0 | 39 | 41 | 39 | 50 | 52 | 51 | 53 | 52 | 48 | 49 | 84 | 50 | 100 | 55 | 53 | 53 | 53 | 52 | 100 | 100 | 100 | 84 | 80 |
| Rv1934c | *fadE17* | 409 | 41 | 0 | 45 | 0 | 43 | 42 | 42 | 61 | 58 | 58 | 60 | 59 | 59 | 59 | 86 | 59 | 100 | 58 | 58 | 58 | 58 | 57 | 99 | 100 | 100 | 85 | 86 |
| Rv1935c | *echA13* | 318 | 44 | 0 | 39 | 0 | 43 | 43 | 42 | 48 | 55 | 55 | 55 | 54 | 45 | 71 | 92 | 71 | 100 | 71 | 74 | 74 | 74 | 72 | 100 | 100 | 100 | 91 | 88 |
| Rv1936 | - | 369 | 0 | 0 | 44 | 0 | 0 | 0 | 0 | 0 | 0 | 45 | 55 | 45 | 45 | 48 | 98 | 48 | 100 | 44 | 48 | 48 | 48 | 47 | 100 | 100 | 100 | 98 | 96 |
| Rv1937 | - | 839 | 0 | 0 | 49 | 50 | 0 | 0 | 0 | 43 | 46 | 47 | 49 | 46 | 44 | 45 | 45 | 45 | 100 | 52 | 49 | 48 | 48 | 47 | 100 | 100 | 100 | 90 | 87 |
| Rv1938 | *ephB* | 356 | 58 | 48 | 58 | 56 | 51 | 47 | 51 | 53 | 44 | 49 | 50 | 49 | 48 | 48 | 94 | 48 | 100 | 46 | 46 | 47 | 47 | 50 | 100 | 100 | 100 | 93 | 92 |
| Rv1939 | - | 171 | 0 | 0 | 45 | 50 | 0 | 0 | 0 | 54 | 57 | 49 | 64 | 66 | 64 | 64 | 88 | 64 | 100 | 63 | 70 | 64 | 64 | 63 | 100 | 100 | 100 | 88 | 82 |
| Rv1940 | *ribA1* | 353 | 54 | 61 | 54 | 60 | 61 | 61 | 61 | 55 | 65 | 58 | 59 | 59 | 64 | 64 | 81 | 64 | 100 | 57 | 57 | 57 | 57 | 64 | 100 | 100 | 99 | 81 | 75 |
| Rv1941 | - | 256 | 46 | 46 | 54 | 53 | 45 | 50 | 43 | 53 | 52 | 61 | 59 | 55 | 54 | 52 | 56 | 56 | 100 | 55 | 56 | 56 | 56 | 57 | 100 | 100 | 100 | 85 | 83 |
| Rv1942c | - | 109 | 0 | 0 | 0 | 0 | 0 | 0 | 0 | 0 | 0 | 0 | 0 | 0 | 0 | 0 | 0 | 0 | 100 | 0 | 0 | 0 | 0 | 0 | 100 | 100 | 100 | 0 | 0 |
| Rv1943c | - | 125 | 0 | 0 | 0 | 0 | 0 | 0 | 0 | 0 | 0 | 0 | 0 | 0 | 0 | 0 | 0 | 0 | 100 | 0 | 0 | 0 | 0 | 0 | 100 | 100 | 100 | 0 | 0 |
| Rv1944c | - | 196 | 0 | 0 | 0 | 0 | 0 | 0 | 0 | 0 | 0 | 0 | 0 | 0 | 0 | 0 | 0 | 0 | 100 | 0 | 0 | 0 | 0 | 0 | 100 | 100 | 100 | 0 | 0 |
| Rv1945 | - | 454 | 43 | 46 | 42 | 44 | 40 | 0 | 0 | 36 | 0 | 39 | 55 | 50 | 68 | 74 | 76 | 74 | 100 | 59 | 58 | 58 | 58 | 54 | 100 | 100 | 100 | 75 | 58 |
| Rv1946c | *lppG* | 150 | 0 | 0 | 0 | 0 | 0 | 0 | 0 | 0 | 0 | 0 | 0 | 0 | 0 | 0 | 0 | 0 | 99 | 0 | 0 | 0 | 0 | 0 | 100 | 100 | 100 | 0 | 0 |
| Rv1947 | - | 133 | 0 | 0 | 0 | 0 | 0 | 0 | 0 | 0 | 0 | 0 | 0 | 0 | 0 | 0 | 0 | 0 | 100 | 0 | 0 | 62 | 62 | 0 | 100 | 100 | 100 | 0 | 0 |
| Rv1948c | - | 116 | 0 | 0 | 0 | 0 | 0 | 0 | 0 | 0 | 0 | 0 | 0 | 0 | 68 | 0 | 0 | 0 | 99 | 0 | 0 | 0 | 0 | 0 | 100 | 100 | 100 | 0 | 0 |
| Rv1949c | - | 319 | 0 | 0 | 0 | 0 | 0 | 0 | 0 | 0 | 0 | 0 | 0 | 0 | 63 | 62 | 58 | 80 | 99 | 0 | 0 | 0 | 0 | 41 | 100 | 100 | 100 | 58 | 0 |
| Rv1950c | - | 63 | 0 | 0 | 0 | 0 | 0 | 0 | 0 | 0 | 0 | 0 | 0 | 0 | 0 | 76 | 84 | 84 | 100 | 0 | 0 | 0 | 0 | 0 | 100 | 100 | 100 | 84 | 0 |
| Rv1951c | - | 98 | 0 | 0 | 0 | 0 | 0 | 0 | 0 | 0 | 0 | 0 | 0 | 0 | 0 | 55 | 69 | 69 | 100 | 0 | 0 | 0 | 0 | 0 | 100 | 100 | 100 | 71 | 0 |
| Rv1952 | - | 71 | 0 | 0 | 0 | 0 | 0 | 0 | 0 | 0 | 0 | 0 | 0 | 0 | 0 | 0 | 0 | 0 | 100 | 0 | 0 | 0 | 0 | 0 | 100 | 100 | 100 | 0 | 0 |
| Rv1953 | - | 103 | 0 | 0 | 0 | 0 | 0 | 0 | 0 | 0 | 60 | 0 | 0 | 0 | 0 | 0 | 0 | 0 | 100 | 0 | 0 | 0 | 0 | 0 | 100 | 100 | 100 | 0 | 0 |
| Rv1954c | - | 173 | 0 | 0 | 0 | 0 | 0 | 0 | 0 | 0 | 0 | 0 | 0 | 0 | 0 | 0 | 0 | 0 | 99 | 0 | 0 | 0 | 0 | 0 | 100 | 100 | 100 | 0 | 0 |
| Rv1955 | - | 170 | 0 | 0 | 0 | 0 | 0 | 0 | 0 | 0 | 0 | 0 | 0 | 0 | 0 | 0 | 0 | 0 | 100 | 0 | 0 | 0 | 0 | 0 | 100 | 100 | 100 | 0 | 0 |
| Rv1956 | - | 149 | 0 | 0 | 0 | 70 | 0 | 0 | 0 | 0 | 0 | 0 | 0 | 0 | 0 | 0 | 0 | 0 | 100 | 0 | 0 | 0 | 0 | 0 | 100 | 100 | 100 | 0 | 0 |
| Rv1957 | - | 181 | 0 | 0 | 0 | 0 | 0 | 0 | 0 | 0 | 0 | 0 | 0 | 0 | 0 | 0 | 0 | 0 | 100 | 0 | 0 | 0 | 0 | 0 | 100 | 100 | 100 | 0 | 0 |
| Rv1958c | - | 204 | 0 | 0 | 0 | 0 | 0 | 0 | 0 | 0 | 0 | 0 | 0 | 0 | 0 | 0 | 0 | 0 | 100 | 0 | 0 | 0 | 0 | 0 | 100 | 100 | 100 | 0 | 0 |
| Rv1959c | - | 98 | 0 | 0 | 0 | 0 | 0 | 0 | 0 | 0 | 0 | 0 | 0 | 0 | 0 | 0 | 0 | 0 | 100 | 0 | 0 | 0 | 0 | 0 | 100 | 100 | 100 | 0 | 0 |
| Rv1960c | - | 83 | 0 | 0 | 0 | 0 | 0 | 0 | 0 | 0 | 0 | 0 | 0 | 0 | 0 | 92 | 0 | 92 | 100 | 0 | 0 | 0 | 0 | 0 | 100 | 100 | 100 | 0 | 0 |

| | | | | | | | | | | | | | | | | | | | | | | | | | | | | |
|---|---|---|---|---|---|---|---|---|---|---|---|---|---|---|---|---|---|---|---|---|---|---|---|---|---|---|---|---|---|
| Rv1961 | - | 164 | 0 | 0 | 0 | 0 | 0 | 0 | 0 | 0 | 0 | 0 | 0 | 0 | 0 | 0 | 0 | 0 | 100 | 0 | 0 | 0 | 0 | 0 | 99 | 100 | 100 | 0 | 0 |
| Rv1962c | - | 135 | 0 | 0 | 0 | 0 | 0 | 0 | 0 | 0 | 0 | 0 | 0 | 0 | 0 | 0 | 0 | 0 | 100 | 0 | 0 | 0 | 0 | 0 | 100 | 100 | 100 | 0 | 57 |
| Rv1963c | *mce3R* | 406 | 0 | 0 | 0 | 0 | 0 | 0 | 0 | 54 | 46 | 50 | 52 | 45 | 50 | 42 | 42 | 42 | 100 | 45 | 44 | 44 | 44 | 46 | 100 | 100 | 100 | 90 | 82 |
| Rv1964 | *yrbE3A* | 265 | 0 | 0 | 0 | 0 | 0 | 0 | 0 | 64 | 73 | 73 | 74 | 72 | 74 | 74 | 74 | 74 | 77 | 80 | 80 | 79 | 79 | 80 | 100 | 100 | 100 | 90 | 83 |
| Rv1965 | *yrbE3B* | 271 | 0 | 0 | 0 | 0 | 0 | 0 | 0 | 69 | 73 | 71 | 72 | 76 | 72 | 75 | 95 | 79 | 74 | 82 | 79 | 81 | 81 | 84 | 100 | 100 | 100 | 93 | 85 |
| Rv1966 | *mce3A* | 425 | 0 | 0 | 0 | 0 | 0 | 0 | 0 | 52 | 55 | 57 | 56 | 56 | 53 | 71 | 74 | 74 | 52 | 76 | 77 | 76 | 76 | 73 | 100 | 100 | 100 | 88 | 82 |
| Rv1967 | *mce3B* | 342 | 0 | 0 | 0 | 0 | 0 | 0 | 0 | 57 | 57 | 60 | 60 | 63 | 56 | 72 | 76 | 76 | 61 | 80 | 78 | 77 | 77 | 78 | 100 | 100 | 99 | 91 | 82 |
| Rv1968 | *mce3C* | 410 | 0 | 0 | 0 | 0 | 0 | 0 | 0 | 59 | 57 | 60 | 63 | 60 | 56 | 62 | 89 | 77 | 56 | 77 | 78 | 77 | 77 | 80 | 100 | 100 | 100 | 88 | 83 |
| Rv1969 | *mce3D* | 423 | 0 | 0 | 0 | 0 | 0 | 0 | 0 | 47 | 58 | 56 | 58 | 64 | 53 | 71 | 89 | 74 | 55 | 72 | 76 | 76 | 76 | 76 | 100 | 100 | 100 | 88 | 81 |
| Rv1970 | *lprM* | 377 | 0 | 0 | 0 | 0 | 0 | 0 | 0 | 55 | 62 | 61 | 59 | 64 | 58 | 76 | 79 | 79 | 57 | 80 | 80 | 77 | 77 | 78 | 100 | 100 | 100 | 92 | 81 |
| Rv1971 | *mce3F* | 437 | 0 | 0 | 0 | 0 | 0 | 0 | 0 | 50 | 58 | 63 | 59 | 60 | 55 | 69 | 88 | 76 | 56 | 74 | 76 | 74 | 74 | 75 | 99 | 100 | 99 | 88 | 74 |
| Rv1972 | - | 191 | 0 | 0 | 0 | 0 | 0 | 0 | 0 | 46 | 55 | 49 | 55 | 58 | 0 | 63 | 80 | 70 | 51 | 67 | 69 | 66 | 66 | 66 | 100 | 100 | 100 | 80 | 70 |
| Rv1973 | - | 160 | 0 | 0 | 0 | 0 | 0 | 0 | 0 | 0 | 51 | 52 | 59 | 64 | 0 | 71 | 80 | 73 | 62 | 73 | 78 | 71 | 71 | 71 | 100 | 100 | 100 | 81 | 71 |
| Rv1974 | - | 125 | 0 | 0 | 0 | 0 | 0 | 0 | 0 | 0 | 0 | 0 | 0 | 0 | 0 | 0 | 63 | 77 | 0 | 68 | 72 | 75 | 75 | 74 | 100 | 100 | 100 | 0 | 71 |
| Rv1975 | - | 221 | 0 | 0 | 0 | 0 | 0 | 0 | 0 | 0 | 0 | 0 | 0 | 0 | 0 | 78 | 85 | 77 | 0 | 83 | 83 | 79 | 79 | 78 | 100 | 100 | 100 | 75 | 84 |
| Rv1976c | - | 135 | 0 | 0 | 0 | 0 | 0 | 0 | 0 | 0 | 0 | 0 | 51 | 52 | 0 | 79 | 81 | 79 | 0 | 0 | 73 | 72 | 72 | 70 | 100 | 100 | 100 | 0 | 77 |
| Rv1977 | - | 348 | 0 | 0 | 0 | 0 | 0 | 0 | 0 | 68 | 0 | 78 | 77 | 77 | 77 | 0 | 0 | 0 | 0 | 0 | 0 | 0 | 0 | 90 | 100 | 100 | 100 | 0 | 0 |
| Rv1978 | - | 282 | 0 | 0 | 0 | 0 | 0 | 0 | 0 | 0 | 0 | 0 | 0 | 0 | 70 | 74 | 0 | 74 | 99 | 63 | 69 | 68 | 68 | 66 | 100 | 100 | 100 | 0 | 66 |
| Rv1979c | - | 481 | 41 | 41 | 39 | 41 | 40 | 39 | 40 | 42 | 43 | 44 | 44 | 43 | 44 | 44 | 45 | 44 | 99 | 45 | 44 | 44 | 44 | 42 | 100 | 100 | 100 | 45 | 44 |
| Rv1980c | *mpt64* | 228 | 0 | 0 | 0 | 0 | 0 | 0 | 0 | 0 | 0 | 0 | 0 | 0 | 58 | 63 | 66 | 66 | 100 | 62 | 64 | 64 | 64 | 64 | 100 | 100 | 100 | 82 | 62 |
| Rv1981c | *nrdF1* | 322 | 82 | 81 | 80 | 80 | 80 | 82 | 79 | 0 | 84 | 81 | 83 | 83 | 84 | 84 | 84 | 84 | 99 | 83 | 83 | 83 | 83 | 82 | 100 | 100 | 100 | 83 | 83 |
| Rv1982c | - | 139 | 0 | 0 | 0 | 0 | 0 | 0 | 0 | 0 | 0 | 0 | 0 | 0 | 0 | 0 | 0 | 0 | 100 | 0 | 0 | 0 | 0 | 54 | 99 | 100 | 100 | 0 | 55 |
| Rv1983 | *PE_PGRS35* | 558 | 0 | 43 | 40 | 36 | 37 | 0 | 40 | 40 | 52 | 43 | 38 | 42 | 42 | 45 | 80 | 43 | 99 | 53 | 56 | 56 | 56 | 48 | 99 | 100 | 100 | 57 | 48 |
| Rv1984c | *cfp21* | 217 | 0 | 0 | 0 | 0 | 0 | 0 | 0 | 0 | 0 | 41 | 55 | 0 | 63 | 87 | 85 | 87 | 100 | 61 | 60 | 60 | 60 | 59 | 100 | 100 | 100 | 58 | 62 |
| Rv1985c | - | 303 | 55 | 60 | 60 | 60 | 0 | 0 | 0 | 56 | 59 | 60 | 58 | 59 | 66 | 0 | 0 | 0 | 100 | 78 | 85 | 85 | 85 | 81 | 100 | 100 | 100 | 0 | 80 |
| Rv1986 | - | 199 | 57 | 56 | 51 | 50 | 0 | 0 | 0 | 65 | 68 | 68 | 70 | 70 | 76 | 0 | 0 | 0 | 100 | 80 | 85 | 85 | 85 | 82 | 100 | 100 | 100 | 0 | 81 |
| Rv1987 | - | 142 | 0 | 0 | 0 | 0 | 0 | 0 | 0 | 0 | 0 | 0 | 0 | 0 | 83 | 86 | 85 | 86 | 100 | 54 | 0 | 0 | 0 | 0 | 100 | 100 | 100 | 84 | 57 |
| Rv1988 | - | 179 | 0 | 0 | 0 | 0 | 46 | 0 | 46 | 0 | 50 | 0 | 0 | 0 | 71 | 0 | 0 | 0 | 100 | 0 | 0 | 0 | 0 | 0 | 100 | 100 | 100 | 0 | 0 |
| Rv1989c | - | 186 | 0 | 0 | 0 | 0 | 0 | 0 | 0 | 0 | 0 | 0 | 0 | 0 | 0 | 0 | 0 | 0 | 100 | 0 | 87 | 0 | 0 | 0 | 100 | 100 | 100 | 0 | 0 |
| Rv1990A | - | 111 | 0 | 0 | 0 | 0 | 0 | 0 | 0 | 0 | 0 | 0 | 0 | 0 | 0 | 0 | 0 | 0 | 100 | 0 | 60 | 0 | 0 | 0 | 100 | 100 | 100 | 0 | 0 |
| Rv1990c | - | 113 | 0 | 0 | 0 | 0 | 0 | 0 | 0 | 0 | 0 | 0 | 0 | 0 | 0 | 0 | 0 | 0 | 100 | 0 | 94 | 0 | 0 | 0 | 100 | 100 | 100 | 0 | 0 |
| Rv1991c | - | 114 | 0 | 0 | 0 | 0 | 0 | 0 | 0 | 0 | 0 | 0 | 0 | 0 | 0 | 0 | 0 | 0 | 100 | 0 | 0 | 0 | 0 | 0 | 100 | 100 | 100 | 0 | 0 |
| Rv1992c | *ctpG* | 771 | 67 | 53 | 69 | 57 | 69 | 57 | 69 | 81 | 56 | 51 | 51 | 51 | 79 | 52 | 87 | 52 | 100 | 84 | 84 | 76 | 76 | 53 | 100 | 100 | 100 | 53 | 82 |
| Rv1993c | - | 90 | 0 | 0 | 0 | 0 | 0 | 0 | 0 | 0 | 0 | 0 | 0 | 72 | 0 | 76 | 75 | 77 | 100 | 0 | 0 | 0 | 0 | 73 | 100 | 100 | 100 | 72 | 0 |
| Rv1994c | - | 118 | 71 | 0 | 74 | 72 | 70 | 68 | 70 | 71 | 77 | 71 | 72 | 72 | 58 | 0 | 53 | 0 | 100 | 76 | 67 | 67 | 67 | 67 | 100 | 100 | 100 | 0 | 72 |
| Rv1995 | - | 255 | 0 | 0 | 0 | 0 | 0 | 0 | 0 | 0 | 0 | 0 | 0 | 0 | 0 | 0 | 77 | 0 | 100 | 0 | 0 | 0 | 0 | 0 | 100 | 100 | 100 | 77 | 0 |
| Rv1996 | - | 317 | 54 | 52 | 54 | 52 | 53 | 56 | 54 | 45 | 57 | 53 | 56 | 55 | 51 | 64 | 75 | 63 | 100 | 63 | 60 | 60 | 60 | 60 | 99 | 100 | 100 | 75 | 60 |
| Rv1997 | *ctpF* | 905 | 48 | 39 | 58 | 57 | 42 | 44 | 42 | 56 | 48 | 52 | 51 | 50 | 43 | 45 | 86 | 45 | 100 | 56 | 81 | 81 | 81 | 62 | 100 | 100 | 100 | 86 | 61 |

| | | | | | | | | | | | | | | | | | | | | | | | | | | | | | |
|---|---|---|---|---|---|---|---|---|---|---|---|---|---|---|---|---|---|---|---|---|---|---|---|---|---|---|---|---|---|
| Rv1998c | - | 258 | 0 | 0 | 42 | 0 | 0 | 0 | 0 | 71 | 45 | 46 | 48 | 49 | 47 | 0 | 46 | 0 | 99 | 46 | 45 | 45 | 45 | 46 | 100 | 100 | 100 | 46 | 46 |
| Rv1999c | - | 440 | 44 | 41 | 44 | 44 | 46 | 44 | 41 | 46 | 76 | 72 | 76 | 74 | 83 | 43 | 43 | 43 | 100 | 81 | 79 | 79 | 79 | 79 | 100 | 100 | 100 | 44 | 81 |
| Rv2000 | - | 537 | 0 | 0 | 0 | 0 | 0 | 0 | 0 | 58 | 0 | 0 | 0 | 0 | 0 | 74 | 83 | 74 | 100 | 67 | 66 | 66 | 66 | 0 | 100 | 100 | 100 | 0 | 0 |
| Rv2001 | - | 250 | 0 | 0 | 0 | 0 | 0 | 0 | 0 | 0 | 62 | 61 | 62 | 62 | 60 | 54 | 86 | 54 | 99 | 72 | 59 | 59 | 59 | 56 | 100 | 100 | 100 | 55 | 55 |
| Rv2002 | *fabG3* | 260 | 48 | 50 | 52 | 55 | 48 | 65 | 49 | 64 | 66 | 64 | 68 | 65 | 64 | 92 | 92 | 92 | 100 | 86 | 78 | 78 | 78 | 78 | 99 | 100 | 100 | 78 | 76 |
| Rv2003c | - | 285 | 0 | 0 | 0 | 0 | 0 | 0 | 0 | 0 | 0 | 0 | 0 | 0 | 0 | 0 | 0 | 0 | 100 | 0 | 0 | 0 | 0 | 0 | 100 | 100 | 100 | 0 | 0 |
| Rv2004c | - | 498 | 0 | 0 | 0 | 0 | 0 | 0 | 0 | 0 | 0 | 58 | 61 | 0 | 69 | 46 | 74 | 48 | 100 | 65 | 63 | 63 | 63 | 68 | 100 | 100 | 100 | 0 | 67 |
| Rv2005c | - | 295 | 58 | 57 | 56 | 56 | 53 | 56 | 56 | 48 | 63 | 61 | 60 | 57 | 56 | 80 | 87 | 79 | 100 | 71 | 70 | 70 | 70 | 65 | 100 | 100 | 100 | 87 | 69 |
| Rv2006 | *otsB1* | 1327 | 45 | 46 | 61 | 46 | 42 | 42 | 43 | 43 | 60 | 71 | 69 | 41 | 47 | 65 | 71 | 64 | 99 | 73 | 72 | 72 | 72 | 73 | 100 | 100 | 100 | 75 | 74 |
| Rv2007c | *fdxA* | 114 | 74 | 72 | 74 | 74 | 72 | 70 | 75 | 77 | 67 | 71 | 70 | 71 | 69 | 83 | 85 | 83 | 100 | 81 | 88 | 88 | 88 | 80 | 100 | 100 | 100 | 79 | 83 |
| Rv2008c | - | 441 | 44 | 0 | 0 | 0 | 0 | 0 | 0 | 40 | 44 | 0 | 0 | 0 | 0 | 0 | 0 | 0 | 100 | 0 | 0 | 0 | 0 | 0 | 100 | 100 | 100 | 0 | 0 |
| Rv2009 | - | 80 | 0 | 0 | 0 | 0 | 0 | 0 | 0 | 0 | 0 | 0 | 0 | 0 | 0 | 0 | 0 | 0 | 100 | 0 | 0 | 0 | 0 | 0 | 100 | 100 | 100 | 0 | 0 |
| Rv2010 | - | 132 | 0 | 0 | 0 | 0 | 0 | 0 | 0 | 0 | 0 | 0 | 0 | 0 | 0 | 0 | 0 | 0 | 100 | 0 | 0 | 0 | 0 | 0 | 100 | 100 | 100 | 0 | 0 |
| Rv2011c | - | 143 | 0 | 0 | 0 | 0 | 0 | 0 | 0 | 0 | 48 | 0 | 0 | 0 | 0 | 0 | 0 | 0 | 100 | 0 | 0 | 0 | 0 | 0 | 100 | 100 | 100 | 0 | 0 |
| Rv2012 | - | 164 | 0 | 0 | 0 | 0 | 0 | 0 | 0 | 0 | 0 | 0 | 0 | 0 | 0 | 0 | 0 | 0 | 100 | 0 | 0 | 0 | 0 | 0 | 100 | 100 | 100 | 0 | 0 |
| Rv2013 | - | 159 | 0 | 0 | 0 | 0 | 0 | 0 | 0 | 75 | 0 | 0 | 0 | 0 | 0 | 48 | 0 | 50 | 100 | 48 | 0 | 0 | 0 | 48 | 100 | 100 | 100 | 0 | 46 |
| Rv2014 | - | 196 | 0 | 0 | 0 | 0 | 0 | 0 | 0 | 73 | 0 | 0 | 0 | 0 | 0 | 46 | 45 | 45 | 100 | 48 | 0 | 0 | 0 | 48 | 100 | 100 | 100 | 0 | 47 |
| Rv2015c | - | 418 | 0 | 0 | 0 | 0 | 0 | 0 | 0 | 43 | 0 | 43 | 37 | 38 | 0 | 82 | 0 | 85 | 100 | 75 | 79 | 79 | 79 | 75 | 100 | 100 | 100 | 0 | 75 |
| Rv2016 | - | 191 | 0 | 0 | 0 | 0 | 0 | 0 | 0 | 0 | 0 | 0 | 0 | 0 | 0 | 0 | 0 | 0 | 100 | 0 | 0 | 0 | 0 | 0 | 99 | 100 | 100 | 0 | 0 |
| Rv2017 | - | 346 | 0 | 44 | 41 | 0 | 0 | 0 | 0 | 48 | 41 | 0 | 0 | 0 | 44 | 0 | 0 | 55 | 99 | 0 | 46 | 0 | 0 | 0 | 99 | 100 | 99 | 0 | 57 |
| Rv2018 | - | 239 | 0 | 0 | 0 | 0 | 0 | 0 | 0 | 61 | 0 | 0 | 0 | 0 | 0 | 0 | 0 | 0 | 99 | 0 | 0 | 0 | 0 | 0 | 100 | 100 | 100 | 0 | 49 |
| Rv2019 | - | 138 | 0 | 0 | 0 | 0 | 0 | 0 | 0 | 53 | 0 | 0 | 0 | 0 | 0 | 0 | 0 | 0 | 100 | 0 | 0 | 0 | 0 | 0 | 100 | 100 | 100 | 0 | 0 |
| Rv2020c | - | 99 | 0 | 82 | 0 | 83 | 0 | 0 | 82 | 0 | 0 | 0 | 0 | 0 | 0 | 0 | 0 | 0 | 100 | 0 | 0 | 0 | 0 | 0 | 100 | 100 | 100 | 93 | 0 |
| Rv2021c | - | 101 | 0 | 0 | 0 | 0 | 0 | 0 | 0 | 0 | 0 | 0 | 0 | 0 | 0 | 68 | 0 | 0 | 100 | 0 | 0 | 0 | 0 | 0 | 100 | 100 | 100 | 0 | 0 |
| Rv2022c | - | 201 | 0 | 0 | 0 | 0 | 0 | 0 | 0 | 0 | 0 | 0 | 0 | 0 | 0 | 54 | 0 | 0 | 100 | 0 | 0 | 0 | 0 | 0 | 100 | 100 | 99 | 0 | 0 |
| Rv2023c | - | 119 | 0 | 0 | 0 | 0 | 0 | 0 | 0 | 0 | 0 | 0 | 0 | 0 | 0 | 0 | 0 | 0 | 100 | 0 | 0 | 0 | 0 | 0 | 100 | 100 | 100 | 0 | 0 |
| Rv2024c | - | 515 | 0 | 73 | 0 | 72 | 0 | 0 | 59 | 0 | 0 | 0 | 0 | 0 | 0 | 0 | 0 | 0 | 99 | 0 | 0 | 0 | 0 | 0 | 99 | 100 | 99 | 0 | 0 |
| Rv2025c | - | 332 | 45 | 42 | 45 | 72 | 48 | 44 | 47 | 44 | 39 | 85 | 84 | 86 | 86 | 56 | 55 | 55 | 100 | 85 | 87 | 87 | 87 | 41 | 100 | 100 | 100 | 56 | 82 |
| Rv2026c | - | 294 | 57 | 54 | 56 | 56 | 54 | 58 | 56 | 50 | 63 | 62 | 60 | 60 | 54 | 82 | 85 | 83 | 100 | 74 | 78 | 78 | 78 | 77 | 100 | 100 | 100 | 85 | 74 |
| Rv2027c | - | 573 | 45 | 46 | 0 | 0 | 0 | 0 | 0 | 57 | 69 | 67 | 74 | 74 | 66 | 60 | 76 | 60 | 100 | 76 | 78 | 78 | 78 | 79 | 100 | 100 | 100 | 77 | 80 |
| Rv2028c | - | 279 | 39 | 40 | 40 | 41 | 42 | 42 | 43 | 46 | 40 | 47 | 45 | 42 | 56 | 55 | 54 | 55 | 100 | 62 | 60 | 60 | 60 | 59 | 100 | 100 | 100 | 54 | 61 |
| Rv2029c | *pfkB* | 339 | 47 | 46 | 47 | 45 | 0 | 45 | 0 | 46 | 48 | 67 | 71 | 48 | 0 | 0 | 0 | 0 | 100 | 73 | 74 | 74 | 74 | 77 | 100 | 100 | 100 | 0 | 76 |
| Rv2030c | - | 681 | 0 | 0 | 0 | 0 | 0 | 0 | 0 | 47 | 58 | 57 | 60 | 58 | 68 | 72 | 89 | 69 | 100 | 85 | 87 | 87 | 87 | 71 | 100 | 100 | 100 | 58 | 85 |
| Rv2031c | *hspX* | 144 | 0 | 0 | 0 | 0 | 0 | 0 | 0 | 0 | 0 | 0 | 0 | 0 | 50 | 59 | 59 | 59 | 100 | 57 | 76 | 76 | 76 | 78 | 100 | 100 | 100 | 0 | 81 |
| Rv2032 | *acg* | 331 | 0 | 0 | 0 | 0 | 0 | 0 | 0 | 44 | 59 | 52 | 59 | 55 | 71 | 66 | 68 | 66 | 99 | 69 | 72 | 72 | 72 | 74 | 100 | 100 | 100 | 67 | 72 |
| Rv2033c | - | 280 | 61 | 60 | 60 | 61 | 62 | 60 | 61 | 75 | 61 | 64 | 64 | 63 | 82 | 88 | 92 | 88 | 100 | 85 | 84 | 84 | 84 | 87 | 100 | 100 | 100 | 92 | 86 |
| Rv2034 | - | 107 | 0 | 0 | 0 | 0 | 0 | 0 | 0 | 58 | 67 | 59 | 57 | 58 | 0 | 59 | 57 | 59 | 100 | 0 | 79 | 79 | 79 | 0 | 100 | 100 | 100 | 57 | 81 |
| Rv2035 | - | 162 | 0 | 0 | 0 | 0 | 0 | 0 | 0 | 0 | 55 | 0 | 0 | 0 | 0 | 0 | 0 | 0 | 100 | 0 | 79 | 80 | 80 | 0 | 100 | 100 | 100 | 0 | 84 |

| | | | | | | | | | | | | | | | | | | | | | | | | | | | | |
|---|---|---|---|---|---|---|---|---|---|---|---|---|---|---|---|---|---|---|---|---|---|---|---|---|---|---|---|---|
| Rv2036 | - | 213 | 0 | 0 | 0 | 0 | 0 | 0 | 0 | 0 | 49 | 45 | 55 | 67 | 65 | 85 | 47 | 85 | 99 | 45 | 77 | 77 | 77 | 46 | 100 | 100 | 100 | 70 | 56 |
| Rv2037c | - | 324 | 0 | 0 | 0 | 0 | 0 | 0 | 0 | 0 | 0 | 0 | 0 | 0 | 0 | 92 | 94 | 92 | 99 | 0 | 0 | 0 | 0 | 0 | 99 | 99 | 99 | 93 | 60 |
| Rv2038c | - | 357 | 68 | 70 | 70 | 68 | 57 | 70 | 70 | 71 | 83 | 79 | 80 | 80 | 62 | 89 | 88 | 89 | 100 | 67 | 72 | 72 | 72 | 72 | 100 | 100 | 100 | 87 | 62 |
| Rv2039c | - | 280 | 47 | 52 | 60 | 61 | 0 | 58 | 46 | 60 | 47 | 58 | 56 | 55 | 61 | 91 | 92 | 91 | 99 | 51 | 46 | 46 | 46 | 61 | 99 | 100 | 99 | 92 | 51 |
| Rv2040c | - | 300 | 50 | 49 | 55 | 57 | 0 | 58 | 48 | 60 | 49 | 58 | 56 | 54 | 53 | 92 | 92 | 92 | 100 | 51 | 52 | 52 | 52 | 62 | 100 | 100 | 100 | 92 | 51 |
| Rv2041c | - | 439 | 0 | 0 | 38 | 38 | 0 | 39 | 0 | 41 | 0 | 39 | 39 | 38 | 39 | 88 | 86 | 88 | 99 | 0 | 39 | 0 | 0 | 41 | 100 | 100 | 100 | 86 | 0 |
| Rv2042c | - | 265 | 0 | 0 | 0 | 0 | 0 | 0 | 0 | 0 | 54 | 51 | 53 | 0 | 52 | 76 | 83 | 76 | 100 | 60 | 59 | 59 | 59 | 58 | 100 | 100 | 100 | 81 | 61 |
| Rv2043c | *pncA* | 186 | 61 | 0 | 64 | 65 | 53 | 61 | 58 | 69 | 77 | 75 | 72 | 73 | 74 | 83 | 78 | 83 | 99 | 76 | 80 | 80 | 80 | 78 | 100 | 100 | 99 | 76 | 76 |
| Rv2044c | - | 105 | 0 | 0 | 0 | 0 | 0 | 0 | 0 | 0 | 55 | 62 | 58 | 59 | 0 | 0 | 0 | 0 | 100 | 0 | 0 | 0 | 0 | 62 | 100 | 100 | 100 | 0 | 0 |
| Rv2045c | *lipT* | 511 | 51 | 47 | 49 | 51 | 49 | 43 | 42 | 52 | 60 | 58 | 61 | 62 | 72 | 89 | 90 | 89 | 100 | 79 | 80 | 80 | 80 | 81 | 100 | 100 | 100 | 90 | 80 |
| Rv2046 | *lppI* | 218 | 0 | 0 | 0 | 0 | 0 | 0 | 0 | 0 | 0 | 0 | 0 | 0 | 54 | 74 | 74 | 74 | 99 | 62 | 62 | 62 | 62 | 57 | 100 | 100 | 100 | 76 | 62 |
| Rv2047c | - | 854 | 0 | 0 | 56 | 55 | 0 | 0 | 0 | 43 | 0 | 44 | 51 | 41 | 0 | 81 | 82 | 80 | 100 | 42 | 47 | 54 | 54 | 58 | 100 | 100 | 100 | 82 | 42 |
| Rv2048c | *pks12* | 4151 | 50 | 49 | 51 | 52 | 48 | 43 | 49 | 42 | 46 | 47 | 57 | 60 | 51 | 87 | 89 | 87 | 99 | 52 | 51 | 51 | 51 | 51 | 99 | 99 | 99 | 89 | 51 |
| Rv2049c | - | 74 | 0 | 63 | 68 | 75 | 0 | 0 | 0 | 0 | 67 | 77 | 82 | 82 | 90 | 83 | 86 | 83 | 100 | 88 | 87 | 87 | 87 | 82 | 100 | 100 | 100 | 88 | 87 |
| Rv2050 | - | 111 | 88 | 83 | 88 | 85 | 87 | 87 | 89 | 65 | 92 | 94 | 94 | 94 | 95 | 100 | 100 | 100 | 100 | 100 | 100 | 100 | 100 | 100 | 100 | 100 | 100 | 100 | 100 |
| Rv2051c | *ppm1* | 874 | 57 | 56 | 52 | 53 | 56 | 54 | 52 | 47 | 60 | 66 | 63 | 60 | 69 | 77 | 77 | 77 | 100 | 71 | 67 | 67 | 67 | 72 | 100 | 100 | 100 | 79 | 72 |
| Rv2052c | - | 534 | 36 | 0 | 0 | 0 | 0 | 0 | 0 | 40 | 69 | 69 | 71 | 70 | 73 | 86 | 88 | 86 | 100 | 77 | 77 | 77 | 77 | 76 | 99 | 100 | 100 | 87 | 75 |
| Rv2053c | *fxsA* | 175 | 0 | 0 | 45 | 0 | 49 | 0 | 45 | 48 | 59 | 50 | 53 | 52 | 59 | 71 | 75 | 71 | 100 | 62 | 64 | 64 | 64 | 58 | 100 | 100 | 100 | 75 | 63 |
| Rv2054 | - | 237 | 0 | 0 | 0 | 0 | 0 | 0 | 0 | 0 | 63 | 58 | 62 | 61 | 42 | 83 | 92 | 84 | 100 | 42 | 81 | 81 | 81 | 85 | 100 | 100 | 100 | 91 | 42 |
| Rv2055c | *rpsR* | 88 | 84 | 76 | 75 | 75 | 77 | 79 | 82 | 78 | 82 | 78 | 79 | 77 | 71 | 84 | 83 | 71 | 100 | 79 | 68 | 68 | 68 | 74 | 100 | 100 | 100 | 83 | 81 |
| Rv2056c | *rpsN* | 101 | 80 | 81 | 82 | 79 | 80 | 81 | 81 | 0 | 81 | 82 | 83 | 82 | 85 | 86 | 0 | 0 | 100 | 82 | 0 | 0 | 0 | 82 | 100 | 100 | 100 | 86 | 84 |
| Rv2057c | *rpmG* | 54 | 94 | 94 | 94 | 92 | 92 | 92 | 92 | 0 | 98 | 96 | 94 | 94 | 96 | 96 | 90 | 94 | 100 | 94 | 96 | 94 | 94 | 96 | 100 | 100 | 100 | 90 | 94 |
| Rv2058c | *rpmB* | 78 | 87 | 89 | 88 | 89 | 89 | 88 | 85 | 0 | 88 | 89 | 88 | 88 | 93 | 93 | 88 | 91 | 100 | 93 | 91 | 91 | 91 | 92 | 100 | 100 | 100 | 88 | 94 |
| Rv2059 | - | 511 | 42 | 0 | 41 | 46 | 49 | 43 | 0 | 41 | 46 | 59 | 51 | 47 | 50 | 73 | 73 | 73 | 100 | 41 | 44 | 43 | 43 | 40 | 100 | 100 | 100 | 73 | 40 |
| Rv2060 | - | 133 | 80 | 49 | 79 | 78 | 81 | 80 | 81 | 51 | 79 | 78 | 82 | 82 | 84 | 91 | 91 | 91 | 100 | 59 | 60 | 60 | 60 | 60 | 100 | 100 | 100 | 90 | 60 |
| Rv2061c | - | 134 | 0 | 0 | 0 | 0 | 0 | 0 | 0 | 0 | 51 | 59 | 58 | 58 | 62 | 89 | 88 | 89 | 100 | 76 | 77 | 77 | 77 | 79 | 100 | 100 | 100 | 86 | 74 |
| Rv2062c | *cobN* | 1194 | 0 | 76 | 0 | 0 | 0 | 75 | 0 | 76 | 82 | 82 | 83 | 84 | 84 | 93 | 93 | 93 | 99 | 88 | 89 | 89 | 89 | 88 | 100 | 100 | 100 | 93 | 89 |
| Rv2063 | - | 77 | 0 | 0 | 0 | 0 | 0 | 0 | 0 | 0 | 0 | 0 | 0 | 0 | 0 | 0 | 0 | 0 | 100 | 0 | 0 | 0 | 0 | 0 | 100 | 100 | 100 | 0 | 0 |
| Rv2064 | *cobG* | 363 | 44 | 54 | 54 | 45 | 0 | 53 | 0 | 51 | 60 | 65 | 68 | 68 | 66 | 81 | 74 | 74 | 100 | 74 | 78 | 78 | 78 | 75 | 100 | 100 | 100 | 81 | 74 |
| Rv2065 | *cobH* | 208 | 0 | 71 | 0 | 0 | 0 | 66 | 0 | 68 | 81 | 77 | 79 | 79 | 87 | 93 | 93 | 93 | 100 | 91 | 92 | 92 | 92 | 90 | 100 | 100 | 100 | 92 | 89 |
| Rv2066 | *cobI* | 508 | 0 | 65 | 0 | 0 | 0 | 70 | 0 | 66 | 74 | 76 | 78 | 78 | 81 | 91 | 92 | 90 | 100 | 87 | 89 | 89 | 89 | 85 | 100 | 100 | 100 | 91 | 88 |
| Rv2067c | - | 407 | 0 | 0 | 0 | 0 | 0 | 0 | 0 | 0 | 0 | 0 | 0 | 0 | 0 | 76 | 75 | 75 | 100 | 0 | 0 | 0 | 0 | 0 | 100 | 100 | 100 | 87 | 0 |
| Rv2068c | *blaC* | 307 | 0 | 0 | 0 | 0 | 0 | 0 | 0 | 0 | 61 | 0 | 52 | 53 | 57 | 0 | 79 | 0 | 100 | 53 | 52 | 52 | 52 | 52 | 100 | 100 | 100 | 78 | 53 |
| Rv2069 | *sigC* | 185 | 66 | 68 | 68 | 66 | 66 | 67 | 64 | 47 | 45 | 47 | 45 | 44 | 78 | 88 | 87 | 89 | 100 | 45 | 47 | 47 | 47 | 43 | 100 | 100 | 100 | 87 | 0 |
| Rv2070c | *cobK* | 244 | 0 | 63 | 0 | 0 | 0 | 60 | 0 | 61 | 63 | 60 | 64 | 63 | 70 | 83 | 84 | 84 | 99 | 58 | 77 | 76 | 76 | 80 | 100 | 100 | 100 | 85 | 59 |
| Rv2071c | *cobM* | 251 | 45 | 71 | 46 | 47 | 45 | 67 | 45 | 65 | 65 | 77 | 78 | 78 | 84 | 88 | 91 | 88 | 100 | 65 | 88 | 87 | 87 | 86 | 100 | 100 | 100 | 91 | 63 |
| Rv2072c | *cobL* | 390 | 0 | 58 | 51 | 48 | 0 | 56 | 0 | 56 | 64 | 62 | 63 | 63 | 69 | 81 | 86 | 80 | 99 | 78 | 80 | 80 | 80 | 79 | 99 | 100 | 99 | 86 | 80 |
| Rv2073c | - | 249 | 64 | 62 | 67 | 63 | 68 | 57 | 66 | 0 | 79 | 80 | 77 | 78 | 76 | 92 | 91 | 90 | 58 | 81 | 80 | 80 | 80 | 80 | 99 | 100 | 100 | 90 | 82 |

| | | | | | | | | | | | | | | | | | | | | | | | | | | | | | |
|---|---|---|---|---|---|---|---|---|---|---|---|---|---|---|---|---|---|---|---|---|---|---|---|---|---|---|---|---|---|
| Rv2074 | - | 137 | 0 | 0 | 0 | 0 | 0 | 0 | 0 | 65 | 73 | 76 | 74 | 73 | 92 | 97 | 97 | 97 | 0 | 91 | 94 | 94 | 94 | 93 | 100 | 100 | 100 | 94 | 92 |
| Rv2075c | - | 487 | 0 | 0 | 0 | 0 | 0 | 0 | 0 | 0 | 0 | 0 | 0 | 0 | 0 | 0 | 80 | 0 | 97 | 0 | 0 | 0 | 0 | 0 | 100 | 100 | 100 | 79 | 0 |
| Rv2076c | - | 83 | 0 | 0 | 0 | 0 | 0 | 0 | 0 | 0 | 0 | 0 | 0 | 0 | 0 | 0 | 67 | 0 | 100 | 0 | 0 | 0 | 0 | 0 | 100 | 100 | 100 | 66 | 0 |
| Rv2077A | - | 99 | 0 | 0 | 0 | 0 | 0 | 0 | 0 | 0 | 0 | 0 | 0 | 0 | 0 | 59 | 73 | 73 | 100 | 0 | 0 | 0 | 0 | 0 | 100 | 100 | 100 | 0 | 0 |
| Rv2077c | - | 323 | 0 | 0 | 0 | 0 | 0 | 0 | 0 | 0 | 0 | 0 | 0 | 0 | 46 | 53 | 58 | 76 | 100 | 46 | 0 | 0 | 0 | 46 | 100 | 100 | 100 | 62 | 45 |
| Rv2078 | - | 104 | 0 | 0 | 0 | 0 | 0 | 0 | 0 | 0 | 0 | 0 | 0 | 0 | 0 | 69 | 68 | 68 | 99 | 0 | 0 | 0 | 0 | 0 | 99 | 100 | 99 | 69 | 0 |
| Rv2079 | - | 656 | 0 | 0 | 0 | 39 | 0 | 0 | 0 | 0 | 0 | 40 | 38 | 38 | 0 | 63 | 71 | 62 | 99 | 62 | 64 | 64 | 64 | 41 | 99 | 100 | 99 | 70 | 63 |
| Rv2080 | *lppJ* | 187 | 0 | 0 | 0 | 0 | 0 | 0 | 0 | 0 | 0 | 0 | 0 | 0 | 0 | 50 | 55 | 50 | 99 | 0 | 54 | 54 | 54 | 0 | 98 | 100 | 100 | 55 | 50 |
| Rv2081c | - | 146 | 0 | 0 | 0 | 0 | 0 | 0 | 0 | 0 | 0 | 0 | 0 | 0 | 0 | 63 | 66 | 60 | 99 | 0 | 0 | 0 | 0 | 0 | 100 | 100 | 99 | 68 | 0 |
| Rv2082 | - | 721 | 37 | 38 | 42 | 39 | 38 | 40 | 41 | 36 | 43 | 38 | 39 | 39 | 34 | 57 | 71 | 58 | 99 | 37 | 37 | 37 | 37 | 37 | 92 | 100 | 99 | 67 | 40 |
| Rv2083 | - | 314 | 0 | 0 | 0 | 0 | 0 | 0 | 34 | 0 | 37 | 0 | 0 | 0 | 0 | 67 | 75 | 67 | 99 | 38 | 35 | 38 | 38 | 0 | 100 | 100 | 100 | 74 | 0 |
| Rv2084 | - | 378 | 0 | 0 | 0 | 0 | 0 | 0 | 0 | 0 | 0 | 0 | 0 | 0 | 0 | 0 | 0 | 0 | 98 | 0 | 0 | 0 | 0 | 0 | 100 | 100 | 100 | 0 | 0 |
| Rv2085 | - | 101 | 0 | 0 | 0 | 0 | 0 | 0 | 0 | 0 | 0 | 0 | 0 | 0 | 0 | 66 | 0 | 0 | 100 | 0 | 0 | 0 | 0 | 0 | 100 | 100 | 100 | 0 | 0 |
| Rv2086 | - | 201 | 0 | 0 | 48 | 0 | 0 | 0 | 0 | 51 | 0 | 0 | 0 | 0 | 0 | 75 | 45 | 47 | 100 | 52 | 42 | 42 | 42 | 45 | 100 | 100 | 100 | 0 | 46 |
| Rv2087 | - | 76 | 0 | 0 | 70 | 0 | 0 | 0 | 0 | 54 | 0 | 0 | 0 | 0 | 0 | 65 | 0 | 0 | 100 | 0 | 0 | 0 | 0 | 0 | 100 | 100 | 100 | 0 | 0 |
| Rv2088 | *pknJ* | 589 | 58 | 50 | 50 | 50 | 56 | 54 | 49 | 54 | 58 | 53 | 59 | 60 | 62 | 54 | 56 | 55 | 100 | 53 | 54 | 54 | 54 | 58 | 100 | 100 | 100 | 56 | 53 |
| Rv2089c | *pepE* | 375 | 59 | 65 | 66 | 67 | 67 | 64 | 54 | 45 | 76 | 79 | 80 | 80 | 83 | 90 | 90 | 90 | 100 | 86 | 87 | 87 | 87 | 87 | 100 | 100 | 100 | 91 | 86 |
| Rv2090 | - | 393 | 53 | 54 | 54 | 53 | 53 | 52 | 53 | 66 | 71 | 74 | 74 | 75 | 78 | 89 | 94 | 90 | 94 | 82 | 86 | 86 | 86 | 85 | 86 | 100 | 99 | 93 | 82 |
| Rv2091c | - | 244 | 0 | 0 | 0 | 0 | 37 | 0 | 0 | 0 | 47 | 44 | 51 | 50 | 61 | 73 | 80 | 74 | 100 | 65 | 66 | 66 | 66 | 62 | 100 | 100 | 100 | 81 | 66 |
| Rv2092c | *helY* | 906 | 63 | 63 | 63 | 64 | 62 | 59 | 61 | 65 | 74 | 73 | 74 | 74 | 79 | 90 | 90 | 90 | 99 | 83 | 86 | 86 | 86 | 86 | 100 | 100 | 100 | 90 | 84 |
| Rv2093c | *tatC* | 308 | 68 | 73 | 68 | 66 | 69 | 68 | 69 | 51 | 79 | 81 | 78 | 82 | 85 | 93 | 93 | 93 | 100 | 83 | 84 | 84 | 84 | 87 | 100 | 100 | 100 | 93 | 85 |
| Rv2094c | *tatA* | 83 | 80 | 65 | 83 | 64 | 64 | 71 | 66 | 0 | 61 | 72 | 65 | 76 | 68 | 81 | 90 | 81 | 100 | 74 | 76 | 76 | 76 | 73 | 100 | 100 | 100 | 90 | 69 |
| Rv2095c | - | 316 | 49 | 49 | 47 | 47 | 46 | 44 | 43 | 49 | 69 | 69 | 67 | 67 | 77 | 88 | 91 | 88 | 99 | 80 | 83 | 83 | 83 | 81 | 100 | 100 | 100 | 91 | 81 |
| Rv2096c | - | 332 | 50 | 47 | 49 | 49 | 46 | 48 | 42 | 55 | 73 | 71 | 70 | 70 | 77 | 90 | 88 | 90 | 100 | 81 | 79 | 79 | 79 | 84 | 100 | 100 | 100 | 88 | 81 |
| Rv2097c | - | 452 | 69 | 68 | 69 | 68 | 68 | 69 | 71 | 85 | 96 | 96 | 96 | 96 | 98 | 98 | 98 | 98 | 100 | 98 | 98 | 98 | 98 | 97 | 100 | 100 | 100 | 98 | 98 |
| Rv2100 | - | 550 | 45 | 47 | 50 | 47 | 44 | 0 | 0 | 38 | 0 | 39 | 34 | 46 | 47 | 53 | 56 | 53 | 100 | 58 | 59 | 60 | 60 | 60 | 100 | 100 | 100 | 53 | 56 |
| Rv2101 | *helZ* | 1013 | 51 | 51 | 58 | 49 | 50 | 49 | 49 | 51 | 54 | 54 | 55 | 55 | 0 | 0 | 0 | 0 | 99 | 0 | 46 | 0 | 0 | 0 | 99 | 100 | 99 | 0 | 0 |
| Rv2102 | - | 238 | 42 | 44 | 44 | 0 | 45 | 0 | 45 | 45 | 46 | 52 | 52 | 51 | 0 | 0 | 0 | 0 | 100 | 0 | 0 | 0 | 0 | 0 | 100 | 100 | 100 | 0 | 0 |
| Rv2103c | - | 144 | 0 | 0 | 0 | 0 | 0 | 0 | 0 | 0 | 0 | 0 | 0 | 0 | 0 | 0 | 0 | 0 | 100 | 83 | 0 | 0 | 0 | 0 | 100 | 100 | 100 | 0 | 0 |
| Rv2104c | - | 84 | 0 | 0 | 0 | 0 | 0 | 0 | 0 | 0 | 0 | 0 | 0 | 0 | 0 | 0 | 0 | 0 | 100 | 90 | 0 | 0 | 0 | 0 | 100 | 100 | 100 | 0 | 0 |
| Rv2105 | - | 108 | 65 | 0 | 66 | 0 | 61 | 0 | 60 | 76 | 0 | 0 | 0 | 0 | 59 | 63 | 82 | 66 | 100 | 82 | 96 | 77 | 77 | 84 | 100 | 100 | 100 | 0 | 62 |
| Rv2106 | - | 312 | 58 | 49 | 61 | 51 | 63 | 0 | 61 | 56 | 0 | 0 | 51 | 52 | 66 | 59 | 66 | 66 | 100 | 66 | 90 | 67 | 67 | 66 | 100 | 100 | 100 | 0 | 65 |
| Rv2107 | *PE22* | 98 | 0 | 0 | 0 | 0 | 0 | 0 | 0 | 0 | 0 | 0 | 0 | 0 | 0 | 57 | 64 | 56 | 100 | 0 | 0 | 0 | 0 | 0 | 100 | 100 | 100 | 60 | 0 |
| Rv2108 | *PPE36* | 243 | 0 | 0 | 0 | 0 | 0 | 0 | 0 | 0 | 0 | 0 | 42 | 42 | 51 | 56 | 59 | 57 | 100 | 54 | 50 | 50 | 50 | 54 | 100 | 100 | 100 | 52 | 51 |
| Rv2109c | *prcA* | 248 | 0 | 0 | 0 | 0 | 0 | 0 | 0 | 72 | 74 | 79 | 78 | 77 | 83 | 90 | 91 | 90 | 99 | 89 | 87 | 87 | 87 | 88 | 99 | 100 | 100 | 91 | 89 |
| Rv2110c | *prcB* | 291 | 0 | 0 | 0 | 0 | 0 | 0 | 0 | 68 | 75 | 78 | 74 | 75 | 81 | 88 | 88 | 88 | 100 | 82 | 83 | 83 | 83 | 87 | 100 | 100 | 100 | 91 | 81 |
| Rv2111c | - | 64 | 0 | 66 | 67 | 70 | 68 | 62 | 67 | 67 | 89 | 85 | 85 | 87 | 85 | 93 | 96 | 93 | 100 | 96 | 93 | 93 | 93 | 98 | 100 | 100 | 100 | 96 | 98 |
| Rv2112c | - | 554 | 65 | 63 | 62 | 63 | 66 | 70 | 65 | 73 | 87 | 91 | 88 | 89 | 94 | 98 | 98 | 97 | 100 | 97 | 94 | 94 | 94 | 97 | 96 | 100 | 99 | 98 | 93 |

| | | | | | | | | | | | | | | | | | | | | | | | | | | | | |
|---|---|---|---|---|---|---|---|---|---|---|---|---|---|---|---|---|---|---|---|---|---|---|---|---|---|---|---|---|
| Rv2113 | - | 397 | 0 | 0 | 0 | 0 | 0 | 0 | 0 | 0 | 0 | 0 | 0 | 0 | 0 | 0 | 72 | 0 | 99 | 62 | 0 | 0 | 0 | 0 | 100 | 100 | 100 | 71 | 65 |
| Rv2114 | - | 207 | 0 | 0 | 0 | 0 | 0 | 0 | 0 | 0 | 0 | 0 | 0 | 0 | 0 | 88 | 85 | 89 | 100 | 77 | 0 | 0 | 0 | 0 | 100 | 100 | 100 | 85 | 78 |
| Rv2115c | - | 609 | 65 | 71 | 69 | 69 | 66 | 68 | 67 | 78 | 89 | 89 | 88 | 88 | 92 | 97 | 97 | 97 | 100 | 93 | 94 | 94 | 94 | 93 | 100 | 100 | 100 | 97 | 93 |
| Rv2116 | *lppK* | 189 | 0 | 0 | 0 | 0 | 0 | 0 | 0 | 0 | 0 | 0 | 0 | 0 | 55 | 66 | 71 | 66 | 100 | 59 | 61 | 61 | 61 | 60 | 100 | 100 | 100 | 69 | 65 |
| Rv2117 | - | 97 | 0 | 0 | 0 | 0 | 0 | 0 | 0 | 0 | 73 | 68 | 72 | 72 | 0 | 93 | 92 | 93 | 100 | 87 | 90 | 90 | 90 | 85 | 100 | 100 | 100 | 91 | 86 |
| Rv2118c | - | 280 | 73 | 71 | 71 | 71 | 71 | 71 | 71 | 68 | 80 | 81 | 82 | 82 | 85 | 93 | 96 | 93 | 100 | 85 | 85 | 85 | 85 | 85 | 100 | 100 | 100 | 94 | 86 |
| Rv2119 | - | 278 | 65 | 62 | 62 | 61 | 66 | 64 | 65 | 69 | 73 | 73 | 79 | 80 | 81 | 88 | 88 | 88 | 100 | 85 | 84 | 84 | 84 | 86 | 100 | 100 | 100 | 87 | 87 |
| Rv2120c | - | 160 | 0 | 0 | 0 | 0 | 0 | 0 | 0 | 0 | 0 | 0 | 0 | 0 | 65 | 82 | 94 | 82 | 100 | 68 | 78 | 78 | 78 | 79 | 100 | 100 | 100 | 0 | 71 |
| Rv2121c | *hisG* | 284 | 71 | 74 | 76 | 74 | 75 | 75 | 75 | 71 | 84 | 85 | 86 | 86 | 85 | 92 | 92 | 92 | 100 | 89 | 89 | 89 | 89 | 90 | 100 | 100 | 100 | 92 | 88 |
| Rv2122c | *hisE* | 93 | 73 | 79 | 85 | 85 | 80 | 82 | 82 | 78 | 86 | 86 | 86 | 86 | 90 | 94 | 95 | 95 | 100 | 91 | 89 | 89 | 89 | 90 | 100 | 100 | 100 | 94 | 91 |
| Rv2123 | *PPE37* | 473 | 0 | 0 | 0 | 0 | 0 | 0 | 0 | 0 | 0 | 38 | 44 | 37 | 54 | 52 | 50 | 50 | 97 | 51 | 54 | 55 | 55 | 55 | 53 | 100 | 99 | 64 | 52 |
| Rv2124c | *metH* | 1192 | 0 | 81 | 82 | 82 | 0 | 78 | 0 | 50 | 90 | 88 | 88 | 88 | 50 | 51 | 51 | 51 | 100 | 50 | 51 | 51 | 51 | 50 | 100 | 100 | 100 | 50 | 50 |
| Rv2125 | - | 292 | 44 | 43 | 43 | 44 | 45 | 45 | 45 | 70 | 80 | 82 | 84 | 84 | 83 | 90 | 92 | 90 | 99 | 88 | 84 | 85 | 85 | 88 | 99 | 100 | 99 | 92 | 88 |
| Rv2126c | *PE_PGRS37* | 256 | 39 | 48 | 39 | 37 | 37 | 37 | 38 | 40 | 44 | 40 | 44 | 41 | 45 | 46 | 60 | 50 | 100 | 57 | 53 | 53 | 53 | 51 | 94 | 100 | 100 | 56 | 57 |
| Rv2127 | *ansP1* | 489 | 53 | 52 | 56 | 56 | 50 | 54 | 54 | 40 | 57 | 77 | 77 | 77 | 80 | 54 | 84 | 52 | 99 | 54 | 53 | 53 | 53 | 53 | 100 | 100 | 100 | 83 | 42 |
| Rv2128 | - | 67 | 0 | 0 | 0 | 0 | 0 | 0 | 0 | 0 | 0 | 0 | 0 | 0 | 0 | 0 | 72 | 0 | 100 | 0 | 0 | 0 | 0 | 0 | 100 | 100 | 100 | 70 | 0 |
| Rv2129c | - | 293 | 47 | 46 | 51 | 48 | 51 | 50 | 46 | 48 | 56 | 57 | 72 | 74 | 78 | 86 | 90 | 86 | 100 | 81 | 80 | 80 | 80 | 80 | 100 | 100 | 100 | 89 | 82 |
| Rv2130c | *cysS* | 414 | 70 | 68 | 70 | 69 | 69 | 66 | 65 | 65 | 83 | 82 | 82 | 81 | 83 | 90 | 91 | 90 | 100 | 85 | 86 | 86 | 86 | 87 | 100 | 100 | 100 | 91 | 87 |
| Rv2131c | *cysQ* | 267 | 64 | 64 | 61 | 62 | 63 | 61 | 62 | 63 | 65 | 66 | 69 | 68 | 76 | 89 | 90 | 90 | 100 | 77 | 78 | 78 | 78 | 78 | 100 | 100 | 100 | 90 | 78 |
| Rv2132 | - | 76 | 0 | 0 | 0 | 0 | 0 | 0 | 0 | 0 | 0 | 0 | 0 | 0 | 0 | 0 | 0 | 0 | 100 | 0 | 0 | 0 | 0 | 0 | 100 | 100 | 100 | 0 | 0 |
| Rv2133c | - | 262 | 0 | 0 | 55 | 54 | 0 | 0 | 0 | 61 | 71 | 71 | 69 | 69 | 74 | 84 | 84 | 84 | 100 | 78 | 79 | 78 | 78 | 80 | 100 | 100 | 100 | 88 | 78 |
| Rv2134c | - | 195 | 0 | 0 | 0 | 0 | 0 | 0 | 0 | 61 | 83 | 86 | 87 | 87 | 92 | 95 | 97 | 95 | 100 | 92 | 92 | 92 | 92 | 88 | 100 | 100 | 100 | 97 | 91 |
| Rv2135c | - | 236 | 44 | 45 | 58 | 57 | 43 | 47 | 48 | 61 | 74 | 75 | 76 | 77 | 79 | 79 | 86 | 79 | 100 | 81 | 81 | 81 | 81 | 81 | 100 | 100 | 100 | 85 | 80 |
| Rv2136c | *uppP* | 276 | 75 | 74 | 75 | 75 | 75 | 76 | 75 | 69 | 70 | 81 | 80 | 81 | 86 | 50 | 94 | 50 | 100 | 87 | 85 | 85 | 85 | 85 | 100 | 100 | 100 | 94 | 86 |
| Rv2137c | - | 137 | 0 | 0 | 0 | 0 | 0 | 0 | 0 | 0 | 63 | 61 | 67 | 63 | 68 | 88 | 88 | 88 | 100 | 79 | 76 | 76 | 76 | 81 | 100 | 100 | 100 | 83 | 81 |
| Rv2138 | *lppL* | 358 | 0 | 42 | 41 | 40 | 42 | 46 | 53 | 0 | 53 | 54 | 50 | 50 | 63 | 80 | 79 | 79 | 99 | 70 | 70 | 70 | 70 | 69 | 100 | 100 | 100 | 79 | 70 |
| Rv2139 | *pyrD* | 357 | 73 | 73 | 75 | 75 | 75 | 68 | 75 | 63 | 72 | 76 | 78 | 78 | 78 | 88 | 89 | 87 | 100 | 83 | 83 | 83 | 83 | 83 | 100 | 100 | 100 | 89 | 82 |
| Rv2140c | *TB18.6* | 176 | 63 | 63 | 68 | 66 | 64 | 66 | 65 | 64 | 77 | 74 | 79 | 78 | 86 | 89 | 91 | 89 | 100 | 82 | 86 | 86 | 86 | 87 | 100 | 100 | 100 | 91 | 83 |
| Rv2141c | - | 448 | 41 | 43 | 41 | 45 | 0 | 0 | 0 | 64 | 85 | 84 | 82 | 83 | 85 | 90 | 93 | 90 | 100 | 87 | 87 | 87 | 87 | 86 | 100 | 100 | 100 | 93 | 87 |
| Rv2142c | - | 105 | 0 | 0 | 0 | 0 | 0 | 0 | 0 | 0 | 0 | 0 | 0 | 0 | 0 | 0 | 0 | 0 | 100 | 0 | 0 | 0 | 0 | 0 | 100 | 100 | 100 | 0 | 0 |
| Rv2143 | - | 352 | 0 | 0 | 0 | 0 | 0 | 0 | 0 | 50 | 61 | 60 | 62 | 61 | 61 | 73 | 73 | 83 | 100 | 73 | 83 | 83 | 83 | 74 | 100 | 100 | 100 | 62 | 77 |
| Rv2144c | - | 118 | 0 | 0 | 0 | 0 | 0 | 0 | 0 | 0 | 0 | 0 | 0 | 0 | 62 | 71 | 65 | 71 | 100 | 60 | 61 | 61 | 61 | 62 | 100 | 100 | 100 | 65 | 68 |
| Rv2145c | *wag31* | 260 | 47 | 53 | 51 | 45 | 49 | 53 | 50 | 54 | 78 | 77 | 77 | 76 | 80 | 91 | 91 | 91 | 100 | 83 | 83 | 84 | 84 | 86 | 100 | 100 | 100 | 91 | 86 |
| Rv2146c | - | 96 | 69 | 64 | 63 | 62 | 67 | 64 | 72 | 70 | 84 | 78 | 77 | 77 | 83 | 97 | 98 | 98 | 100 | 96 | 97 | 97 | 97 | 95 | 100 | 100 | 100 | 97 | 95 |
| Rv2147c | - | 241 | 0 | 48 | 38 | 39 | 42 | 44 | 0 | 55 | 55 | 66 | 67 | 66 | 73 | 89 | 90 | 90 | 100 | 73 | 79 | 79 | 79 | 71 | 100 | 100 | 100 | 93 | 75 |
| Rv2148c | - | 258 | 55 | 49 | 57 | 56 | 55 | 55 | 56 | 54 | 68 | 70 | 66 | 65 | 72 | 82 | 84 | 82 | 100 | 72 | 70 | 70 | 70 | 73 | 100 | 100 | 100 | 84 | 71 |
| Rv2149c | *yfiH* | 250 | 67 | 70 | 71 | 71 | 64 | 70 | 70 | 57 | 77 | 71 | 73 | 73 | 80 | 90 | 82 | 91 | 100 | 80 | 82 | 82 | 82 | 83 | 100 | 100 | 100 | 82 | 82 |
| Rv2150c | *ftsZ* | 379 | 87 | 79 | 90 | 82 | 84 | 86 | 84 | 77 | 80 | 85 | 85 | 85 | 91 | 97 | 97 | 97 | 100 | 90 | 94 | 94 | 94 | 94 | 100 | 100 | 100 | 97 | 92 |

| | | | | | | | | | | | | | | | | | | | | | | | | | | | | | |
|---|---|---|---|---|---|---|---|---|---|---|---|---|---|---|---|---|---|---|---|---|---|---|---|---|---|---|---|---|---|
| Rv2151c | *ftsQ* | 314 | 47 | 51 | 48 | 47 | 45 | 44 | 44 | 53 | 54 | 59 | 59 | 58 | 72 | 81 | 84 | 81 | 100 | 77 | 78 | 78 | 78 | 77 | 100 | 100 | 100 | 82 | 80 |
| Rv2152c | *murC* | 494 | 65 | 66 | 67 | 67 | 65 | 65 | 66 | 61 | 75 | 74 | 74 | 74 | 77 | 90 | 88 | 90 | 100 | 82 | 86 | 86 | 86 | 85 | 100 | 100 | 100 | 88 | 82 |
| Rv2153c | *murG* | 410 | 59 | 63 | 65 | 62 | 66 | 71 | 68 | 67 | 77 | 77 | 76 | 76 | 80 | 84 | 83 | 83 | 99 | 78 | 76 | 76 | 76 | 80 | 100 | 100 | 100 | 84 | 78 |
| Rv2154c | *ftsW* | 524 | 56 | 58 | 52 | 53 | 52 | 56 | 53 | 53 | 68 | 71 | 71 | 71 | 80 | 82 | 84 | 81 | 100 | 80 | 78 | 78 | 78 | 75 | 100 | 100 | 100 | 83 | 79 |
| Rv2155c | *murD* | 486 | 57 | 57 | 58 | 57 | 55 | 56 | 55 | 57 | 63 | 64 | 66 | 66 | 71 | 84 | 83 | 84 | 99 | 77 | 75 | 75 | 75 | 76 | 99 | 100 | 99 | 83 | 75 |
| Rv2156c | *mraY* | 359 | 75 | 75 | 74 | 73 | 77 | 75 | 75 | 66 | 81 | 83 | 86 | 86 | 89 | 95 | 95 | 95 | 100 | 93 | 93 | 93 | 93 | 93 | 100 | 100 | 100 | 95 | 93 |
| Rv2157c | *murF* | 510 | 63 | 61 | 64 | 63 | 61 | 63 | 63 | 54 | 74 | 71 | 72 | 72 | 77 | 86 | 84 | 86 | 100 | 82 | 86 | 86 | 86 | 84 | 99 | 100 | 99 | 84 | 84 |
| Rv2158c | *murE* | 535 | 64 | 64 | 60 | 64 | 59 | 61 | 62 | 60 | 65 | 67 | 67 | 67 | 76 | 84 | 84 | 84 | 100 | 74 | 77 | 78 | 78 | 76 | 100 | 100 | 100 | 84 | 73 |
| Rv2159c | - | 344 | 0 | 0 | 0 | 0 | 0 | 0 | 0 | 0 | 0 | 0 | 0 | 0 | 0 | 0 | 0 | 0 | 100 | 0 | 0 | 0 | 0 | 0 | 100 | 100 | 100 | 0 | 0 |
| Rv2160A | - | 211 | 0 | 0 | 0 | 0 | 0 | 0 | 0 | 62 | 60 | 0 | 0 | 68 | 0 | 0 | 0 | 0 | 99 | 0 | 0 | 0 | 0 | 0 | 100 | 100 | 100 | 0 | 0 |
| Rv2160c | - | 113 | 0 | 0 | 0 | 0 | 0 | 0 | 0 | 53 | 0 | 0 | 0 | 52 | 0 | 0 | 0 | 0 | 99 | 0 | 0 | 0 | 0 | 0 | 100 | 100 | 100 | 0 | 0 |
| Rv2161c | - | 288 | 0 | 0 | 0 | 48 | 0 | 0 | 0 | 54 | 51 | 53 | 52 | 52 | 54 | 53 | 53 | 53 | 100 | 52 | 53 | 53 | 53 | 53 | 100 | 100 | 99 | 52 | 51 |
| Rv2162c | *PE_PGRS38* | 532 | 42 | 42 | 37 | 36 | 37 | 35 | 37 | 36 | 37 | 41 | 41 | 42 | 43 | 62 | 51 | 51 | 87 | 49 | 50 | 50 | 50 | 43 | 100 | 100 | 100 | 61 | 49 |
| Rv2163c | *pbpB* | 679 | 62 | 66 | 61 | 62 | 66 | 68 | 62 | 50 | 73 | 76 | 71 | 78 | 78 | 88 | 88 | 88 | 100 | 81 | 83 | 82 | 82 | 84 | 99 | 100 | 100 | 89 | 83 |
| Rv2164c | - | 384 | 0 | 0 | 38 | 36 | 40 | 38 | 35 | 40 | 45 | 49 | 43 | 43 | 56 | 66 | 67 | 66 | 99 | 62 | 59 | 59 | 59 | 57 | 100 | 100 | 100 | 66 | 61 |
| Rv2165c | *mraW* | 344 | 68 | 67 | 67 | 68 | 65 | 63 | 67 | 67 | 74 | 71 | 75 | 74 | 75 | 87 | 86 | 87 | 100 | 78 | 77 | 77 | 77 | 80 | 100 | 100 | 100 | 86 | 80 |
| Rv2166c | - | 143 | 80 | 80 | 81 | 81 | 79 | 80 | 78 | 64 | 88 | 87 | 88 | 88 | 90 | 97 | 97 | 97 | 100 | 92 | 94 | 94 | 94 | 94 | 100 | 100 | 100 | 97 | 93 |
| Rv2167c | - | 346 | 58 | 49 | 59 | 51 | 61 | 0 | 59 | 64 | 0 | 0 | 49 | 52 | 66 | 58 | 64 | 64 | 100 | 65 | 88 | 64 | 64 | 66 | 100 | 100 | 100 | 0 | 61 |
| Rv2168c | - | 108 | 65 | 0 | 66 | 0 | 61 | 0 | 60 | 76 | 0 | 0 | 0 | 0 | 59 | 63 | 82 | 66 | 100 | 82 | 96 | 77 | 77 | 84 | 100 | 100 | 100 | 0 | 62 |
| Rv2169c | - | 134 | 57 | 55 | 55 | 60 | 53 | 59 | 59 | 51 | 72 | 69 | 69 | 69 | 74 | 89 | 94 | 91 | 100 | 85 | 88 | 88 | 88 | 87 | 100 | 100 | 100 | 94 | 88 |
| Rv2170 | - | 206 | 44 | 51 | 48 | 52 | 55 | 51 | 55 | 0 | 65 | 66 | 65 | 64 | 68 | 82 | 84 | 82 | 100 | 78 | 77 | 76 | 76 | 79 | 100 | 100 | 100 | 84 | 78 |
| Rv2171 | *lppM* | 227 | 0 | 0 | 0 | 0 | 0 | 0 | 0 | 0 | 62 | 64 | 65 | 64 | 74 | 92 | 91 | 92 | 100 | 82 | 81 | 81 | 81 | 83 | 100 | 100 | 100 | 90 | 81 |
| Rv2172c | - | 301 | 0 | 0 | 0 | 0 | 0 | 0 | 0 | 0 | 0 | 0 | 0 | 0 | 82 | 91 | 91 | 91 | 100 | 86 | 86 | 86 | 86 | 83 | 100 | 100 | 100 | 90 | 87 |
| Rv2173 | *idsA2* | 352 | 60 | 60 | 58 | 59 | 55 | 56 | 57 | 58 | 61 | 62 | 62 | 61 | 70 | 83 | 84 | 83 | 100 | 78 | 78 | 78 | 78 | 73 | 100 | 100 | 100 | 84 | 78 |
| Rv2174 | - | 516 | 54 | 55 | 55 | 55 | 53 | 50 | 55 | 48 | 65 | 71 | 71 | 71 | 76 | 86 | 89 | 86 | 100 | 82 | 81 | 81 | 81 | 82 | 100 | 100 | 100 | 89 | 80 |
| Rv2175c | - | 146 | 54 | 54 | 58 | 53 | 54 | 50 | 54 | 47 | 60 | 63 | 61 | 61 | 73 | 92 | 97 | 92 | 99 | 80 | 80 | 81 | 81 | 84 | 100 | 100 | 100 | 97 | 81 |
| Rv2176 | *pknL* | 399 | 60 | 63 | 58 | 58 | 67 | 73 | 69 | 68 | 68 | 64 | 67 | 67 | 71 | 83 | 83 | 83 | 100 | 73 | 76 | 77 | 77 | 73 | 100 | 100 | 100 | 84 | 75 |
| Rv2177c | - | 221 | 0 | 0 | 0 | 0 | 0 | 0 | 0 | 57 | 0 | 0 | 57 | 60 | 0 | 0 | 0 | 0 | 100 | 0 | 0 | 0 | 0 | 0 | 100 | 100 | 99 | 0 | 59 |
| Rv2178c | *aroG* | 462 | 82 | 82 | 83 | 83 | 82 | 81 | 82 | 69 | 90 | 90 | 91 | 91 | 93 | 96 | 96 | 96 | 100 | 93 | 93 | 93 | 93 | 93 | 100 | 100 | 100 | 96 | 93 |
| Rv2179c | - | 168 | 76 | 72 | 75 | 73 | 71 | 72 | 73 | 0 | 76 | 77 | 77 | 77 | 76 | 88 | 89 | 85 | 100 | 87 | 88 | 88 | 88 | 85 | 100 | 100 | 100 | 89 | 87 |
| Rv2180c | - | 295 | 0 | 0 | 0 | 0 | 0 | 0 | 0 | 0 | 64 | 67 | 64 | 64 | 55 | 87 | 85 | 85 | 99 | 75 | 78 | 78 | 78 | 80 | 100 | 100 | 100 | 90 | 77 |
| Rv2181 | - | 427 | 50 | 53 | 55 | 56 | 52 | 51 | 49 | 0 | 56 | 48 | 63 | 62 | 49 | 83 | 87 | 83 | 100 | 73 | 71 | 71 | 71 | 73 | 100 | 100 | 100 | 86 | 77 |
| Rv2182c | - | 247 | 65 | 66 | 64 | 66 | 66 | 65 | 68 | 77 | 77 | 82 | 77 | 78 | 80 | 88 | 93 | 88 | 100 | 82 | 86 | 86 | 86 | 82 | 100 | 100 | 100 | 93 | 82 |
| Rv2183c | - | 131 | 0 | 0 | 0 | 0 | 0 | 0 | 0 | 0 | 50 | 50 | 54 | 54 | 61 | 77 | 83 | 77 | 100 | 64 | 69 | 66 | 66 | 69 | 100 | 100 | 100 | 82 | 71 |
| Rv2184c | - | 379 | 0 | 0 | 0 | 0 | 0 | 0 | 0 | 45 | 54 | 54 | 57 | 57 | 78 | 89 | 90 | 89 | 100 | 84 | 81 | 81 | 81 | 83 | 100 | 100 | 100 | 89 | 82 |
| Rv2185c | *TB16.3* | 144 | 0 | 0 | 0 | 0 | 0 | 0 | 0 | 61 | 71 | 61 | 67 | 66 | 83 | 91 | 96 | 91 | 100 | 86 | 85 | 86 | 86 | 84 | 100 | 100 | 100 | 96 | 87 |
| Rv2186c | - | 129 | 0 | 0 | 0 | 0 | 0 | 0 | 0 | 0 | 74 | 69 | 70 | 70 | 79 | 91 | 92 | 90 | 100 | 79 | 82 | 82 | 82 | 83 | 100 | 100 | 100 | 92 | 77 |
| Rv2187 | *fadD15* | 600 | 64 | 63 | 64 | 64 | 62 | 62 | 64 | 66 | 71 | 75 | 75 | 75 | 82 | 89 | 90 | 89 | 99 | 83 | 85 | 85 | 85 | 86 | 100 | 100 | 100 | 89 | 83 |

| | | | | | | | | | | | | | | | | | | | | | | | | | | | | | |
|---|---|---|---|---|---|---|---|---|---|---|---|---|---|---|---|---|---|---|---|---|---|---|---|---|---|---|---|---|---|
| Rv2188c | - | 385 | 65 | 64 | 64 | 65 | 65 | 65 | 65 | 40 | 74 | 78 | 76 | 77 | 81 | 87 | 92 | 87 | 100 | 81 | 81 | 81 | 81 | 81 | 100 | 100 | 100 | 91 | 80 |
| Rv2189c | - | 257 | 0 | 0 | 0 | 0 | 0 | 0 | 0 | 0 | 48 | 45 | 50 | 50 | 51 | 78 | 78 | 78 | 100 | 54 | 58 | 59 | 59 | 62 | 100 | 100 | 100 | 75 | 59 |
| Rv2190c | - | 385 | 47 | 49 | 47 | 50 | 47 | 50 | 49 | 44 | 59 | 59 | 60 | 59 | 69 | 85 | 87 | 85 | 100 | 73 | 75 | 75 | 75 | 79 | 100 | 100 | 100 | 85 | 74 |
| Rv2191 | - | 645 | 45 | 43 | 44 | 43 | 47 | 51 | 47 | 59 | 67 | 68 | 68 | 69 | 69 | 82 | 82 | 82 | 99 | 76 | 75 | 74 | 74 | 74 | 100 | 100 | 100 | 76 | 77 |
| Rv2192c | *trpD* | 370 | 50 | 49 | 50 | 49 | 63 | 60 | 60 | 63 | 69 | 71 | 73 | 73 | 84 | 87 | 87 | 87 | 100 | 85 | 84 | 84 | 84 | 87 | 100 | 100 | 100 | 87 | 86 |
| Rv2193 | *ctaE* | 203 | 70 | 75 | 77 | 77 | 80 | 78 | 79 | 68 | 88 | 89 | 90 | 90 | 90 | 95 | 97 | 96 | 100 | 92 | 93 | 93 | 93 | 92 | 100 | 100 | 100 | 97 | 91 |
| Rv2194 | *qcrC* | 280 | 71 | 73 | 72 | 71 | 73 | 68 | 71 | 58 | 83 | 83 | 82 | 82 | 88 | 93 | 88 | 93 | 100 | 82 | 82 | 82 | 82 | 87 | 100 | 100 | 100 | 88 | 83 |
| Rv2195 | *qcrA* | 429 | 66 | 68 | 67 | 67 | 66 | 67 | 68 | 50 | 71 | 72 | 71 | 71 | 82 | 86 | 92 | 86 | 100 | 85 | 85 | 85 | 85 | 87 | 100 | 100 | 100 | 92 | 87 |
| Rv2196 | *qcrB* | 549 | 79 | 78 | 80 | 79 | 81 | 80 | 80 | 64 | 86 | 83 | 84 | 84 | 88 | 94 | 94 | 94 | 100 | 89 | 88 | 88 | 88 | 89 | 100 | 100 | 100 | 94 | 89 |
| Rv2197c | - | 214 | 0 | 0 | 0 | 0 | 0 | 0 | 0 | 0 | 0 | 0 | 0 | 0 | 55 | 75 | 75 | 74 | 100 | 54 | 59 | 59 | 59 | 54 | 100 | 100 | 100 | 74 | 58 |
| Rv2198c | *mmpS3* | 299 | 0 | 0 | 0 | 0 | 0 | 0 | 0 | 0 | 46 | 39 | 46 | 46 | 63 | 67 | 80 | 67 | 100 | 68 | 75 | 75 | 75 | 71 | 100 | 100 | 100 | 80 | 70 |
| Rv2199c | - | 139 | 49 | 52 | 52 | 54 | 55 | 47 | 53 | 58 | 82 | 81 | 79 | 79 | 87 | 95 | 97 | 95 | 100 | 88 | 87 | 87 | 87 | 87 | 100 | 100 | 100 | 97 | 87 |
| Rv2200c | *ctaC* | 363 | 63 | 63 | 62 | 64 | 65 | 64 | 66 | 48 | 72 | 73 | 70 | 70 | 82 | 88 | 88 | 88 | 100 | 84 | 81 | 81 | 81 | 85 | 100 | 100 | 100 | 88 | 85 |
| Rv2201 | *asnB* | 652 | 75 | 74 | 75 | 74 | 74 | 74 | 73 | 0 | 79 | 81 | 82 | 82 | 82 | 92 | 92 | 92 | 100 | 86 | 85 | 85 | 85 | 86 | 100 | 100 | 100 | 92 | 86 |
| Rv2202c | *cbhK* | 324 | 38 | 0 | 0 | 0 | 0 | 0 | 0 | 70 | 79 | 78 | 78 | 78 | 84 | 92 | 95 | 91 | 100 | 84 | 87 | 87 | 87 | 86 | 100 | 100 | 100 | 95 | 85 |
| Rv2203 | - | 230 | 0 | 0 | 0 | 0 | 0 | 0 | 0 | 0 | 0 | 0 | 0 | 0 | 53 | 77 | 83 | 77 | 100 | 57 | 65 | 65 | 65 | 67 | 100 | 100 | 100 | 83 | 59 |
| Rv2204c | - | 118 | 85 | 88 | 83 | 87 | 84 | 88 | 85 | 81 | 93 | 91 | 89 | 89 | 89 | 95 | 94 | 95 | 100 | 87 | 92 | 92 | 92 | 94 | 100 | 100 | 100 | 94 | 88 |
| Rv2205c | - | 358 | 45 | 45 | 48 | 47 | 0 | 43 | 45 | 57 | 49 | 71 | 72 | 72 | 75 | 88 | 91 | 88 | 100 | 79 | 47 | 47 | 47 | 50 | 100 | 100 | 100 | 91 | 80 |
| Rv2206 | - | 236 | 54 | 56 | 53 | 50 | 58 | 52 | 62 | 51 | 68 | 66 | 64 | 67 | 71 | 82 | 82 | 82 | 100 | 74 | 75 | 75 | 75 | 72 | 100 | 100 | 100 | 83 | 73 |
| Rv2207 | *cobT* | 361 | 51 | 69 | 69 | 69 | 56 | 71 | 50 | 60 | 77 | 78 | 76 | 75 | 74 | 89 | 89 | 89 | 100 | 82 | 82 | 82 | 82 | 84 | 100 | 100 | 100 | 90 | 84 |
| Rv2208 | *cobS* | 249 | 0 | 46 | 46 | 45 | 0 | 46 | 0 | 50 | 58 | 56 | 61 | 61 | 67 | 78 | 80 | 78 | 99 | 73 | 74 | 74 | 74 | 76 | 100 | 100 | 100 | 80 | 74 |
| Rv2209 | - | 512 | 0 | 0 | 0 | 0 | 0 | 0 | 0 | 0 | 0 | 0 | 0 | 0 | 0 | 0 | 76 | 0 | 100 | 0 | 0 | 0 | 0 | 0 | 100 | 100 | 100 | 75 | 0 |
| Rv2210c | *ilvE* | 368 | 76 | 76 | 76 | 75 | 75 | 75 | 75 | 71 | 82 | 83 | 83 | 82 | 85 | 93 | 92 | 94 | 99 | 90 | 89 | 89 | 89 | 91 | 100 | 100 | 100 | 92 | 91 |
| Rv2211c | *gcvT* | 379 | 56 | 0 | 0 | 0 | 56 | 56 | 56 | 71 | 81 | 80 | 80 | 80 | 83 | 90 | 92 | 90 | 100 | 84 | 85 | 85 | 85 | 88 | 100 | 100 | 100 | 91 | 87 |
| Rv2212 | - | 378 | 0 | 0 | 0 | 0 | 0 | 0 | 0 | 50 | 45 | 50 | 48 | 46 | 49 | 43 | 43 | 43 | 100 | 44 | 46 | 46 | 46 | 79 | 100 | 100 | 100 | 83 | 44 |
| Rv2213 | *pepB* | 515 | 63 | 66 | 63 | 66 | 65 | 0 | 62 | 55 | 71 | 72 | 72 | 72 | 79 | 91 | 90 | 90 | 99 | 80 | 81 | 82 | 82 | 79 | 100 | 100 | 100 | 91 | 81 |
| Rv2214c | *ephD* | 592 | 50 | 46 | 45 | 47 | 50 | 50 | 49 | 51 | 57 | 69 | 66 | 66 | 63 | 88 | 91 | 88 | 100 | 66 | 78 | 78 | 78 | 71 | 100 | 100 | 100 | 91 | 67 |
| Rv2215 | *dlaT* | 553 | 70 | 71 | 71 | 72 | 70 | 68 | 62 | 69 | 78 | 79 | 81 | 80 | 83 | 84 | 83 | 83 | 100 | 77 | 77 | 77 | 77 | 83 | 100 | 100 | 100 | 83 | 82 |
| Rv2216 | - | 301 | 52 | 53 | 57 | 59 | 0 | 53 | 48 | 56 | 74 | 74 | 74 | 75 | 78 | 88 | 90 | 89 | 100 | 84 | 86 | 86 | 86 | 82 | 100 | 100 | 100 | 89 | 84 |
| Rv2217 | *lipB* | 230 | 68 | 71 | 68 | 71 | 73 | 67 | 73 | 72 | 72 | 73 | 73 | 73 | 80 | 85 | 85 | 85 | 99 | 82 | 81 | 81 | 81 | 88 | 100 | 100 | 100 | 84 | 82 |
| Rv2218 | *lipA* | 311 | 81 | 79 | 78 | 78 | 81 | 82 | 81 | 86 | 90 | 89 | 91 | 90 | 91 | 94 | 94 | 94 | 100 | 88 | 91 | 91 | 91 | 91 | 100 | 100 | 100 | 94 | 90 |
| Rv2219 | - | 250 | 60 | 63 | 65 | 63 | 66 | 66 | 64 | 59 | 76 | 75 | 77 | 77 | 81 | 93 | 92 | 92 | 99 | 88 | 92 | 92 | 92 | 88 | 100 | 100 | 99 | 92 | 92 |
| Rv2219A | - | 140 | 58 | 58 | 62 | 60 | 60 | 57 | 60 | 0 | 67 | 61 | 64 | 65 | 73 | 76 | 77 | 77 | 100 | 75 | 76 | 76 | 76 | 78 | 100 | 100 | 100 | 82 | 78 |
| Rv2220 | *glnA1* | 478 | 81 | 79 | 81 | 81 | 83 | 82 | 82 | 83 | 87 | 89 | 89 | 89 | 91 | 94 | 95 | 94 | 100 | 92 | 92 | 92 | 92 | 91 | 100 | 100 | 100 | 94 | 93 |
| Rv2221c | *glnE* | 994 | 67 | 65 | 67 | 68 | 67 | 73 | 65 | 60 | 77 | 78 | 78 | 78 | 81 | 91 | 90 | 91 | 100 | 83 | 83 | 83 | 83 | 84 | 100 | 100 | 100 | 90 | 84 |
| Rv2222c | *glnA2* | 446 | 77 | 78 | 79 | 79 | 83 | 83 | 81 | 77 | 90 | 91 | 90 | 90 | 93 | 97 | 97 | 97 | 100 | 92 | 92 | 92 | 92 | 93 | 100 | 100 | 100 | 95 | 93 |
| Rv2223c | - | 520 | 39 | 0 | 45 | 49 | 0 | 42 | 44 | 51 | 63 | 65 | 61 | 60 | 70 | 85 | 85 | 85 | 100 | 73 | 77 | 77 | 77 | 76 | 100 | 100 | 100 | 85 | 74 |
| Rv2224c | - | 520 | 39 | 0 | 39 | 40 | 0 | 40 | 39 | 50 | 64 | 61 | 62 | 61 | 74 | 90 | 93 | 90 | 100 | 80 | 82 | 82 | 82 | 80 | 100 | 100 | 100 | 93 | 78 |

| | | | | | | | | | | | | | | | | | | | | | | | | | | | | | |
|---|---|---|---|---|---|---|---|---|---|---|---|---|---|---|---|---|---|---|---|---|---|---|---|---|---|---|---|---|---|
| Rv2225 | *panB* | 281 | 68 | 66 | 70 | 66 | 72 | 75 | 69 | 73 | 86 | 84 | 86 | 86 | 83 | 92 | 92 | 91 | 100 | 89 | 90 | 90 | 90 | 86 | 100 | 100 | 100 | 91 | 88 |
| Rv2226 | - | 513 | 48 | 48 | 52 | 50 | 0 | 44 | 0 | 46 | 50 | 46 | 48 | 47 | 69 | 78 | 76 | 77 | 99 | 72 | 75 | 75 | 75 | 74 | 100 | 100 | 100 | 76 | 74 |
| Rv2227 | - | 233 | 0 | 0 | 0 | 0 | 0 | 0 | 0 | 0 | 0 | 73 | 76 | 77 | 0 | 86 | 0 | 86 | 90 | 0 | 0 | 0 | 0 | 86 | 100 | 100 | 100 | 0 | 0 |
| Rv2228c | - | 364 | 61 | 68 | 65 | 68 | 56 | 55 | 55 | 58 | 68 | 72 | 75 | 75 | 80 | 82 | 81 | 81 | 100 | 81 | 82 | 82 | 82 | 82 | 100 | 100 | 100 | 87 | 81 |
| Rv2229c | - | 245 | 46 | 45 | 48 | 46 | 45 | 50 | 0 | 53 | 65 | 66 | 64 | 63 | 71 | 83 | 88 | 83 | 100 | 74 | 78 | 78 | 78 | 79 | 100 | 100 | 100 | 88 | 77 |
| Rv2230c | - | 379 | 70 | 67 | 64 | 64 | 64 | 64 | 63 | 60 | 73 | 73 | 74 | 73 | 77 | 88 | 88 | 88 | 100 | 81 | 83 | 83 | 83 | 84 | 100 | 100 | 100 | 89 | 82 |
| Rv2231c | *cobC* | 364 | 50 | 61 | 56 | 40 | 0 | 51 | 0 | 58 | 68 | 68 | 68 | 69 | 72 | 82 | 80 | 83 | 100 | 41 | 76 | 76 | 76 | 41 | 100 | 100 | 100 | 80 | 40 |
| Rv2232 | - | 291 | 0 | 59 | 56 | 53 | 55 | 0 | 58 | 38 | 0 | 62 | 54 | 53 | 75 | 71 | 76 | 71 | 100 | 68 | 73 | 73 | 73 | 69 | 100 | 100 | 100 | 75 | 67 |
| Rv2234 | *ptpA* | 163 | 61 | 66 | 64 | 62 | 55 | 59 | 58 | 50 | 69 | 69 | 69 | 68 | 81 | 89 | 89 | 89 | 100 | 81 | 79 | 79 | 79 | 78 | 100 | 100 | 100 | 89 | 82 |
| Rv2235 | - | 271 | 56 | 59 | 62 | 61 | 57 | 60 | 59 | 46 | 61 | 65 | 62 | 63 | 73 | 85 | 90 | 85 | 100 | 77 | 82 | 82 | 82 | 78 | 100 | 100 | 100 | 90 | 79 |
| Rv2236c | *cobD* | 313 | 0 | 65 | 62 | 0 | 0 | 63 | 0 | 60 | 70 | 66 | 68 | 67 | 68 | 78 | 84 | 78 | 99 | 73 | 76 | 76 | 76 | 78 | 99 | 100 | 99 | 84 | 73 |
| Rv2237 | - | 255 | 0 | 0 | 0 | 0 | 0 | 0 | 0 | 0 | 64 | 64 | 65 | 63 | 62 | 90 | 93 | 90 | 100 | 61 | 64 | 64 | 64 | 62 | 100 | 100 | 100 | 94 | 62 |
| Rv2238c | *ahpE* | 153 | 45 | 0 | 0 | 0 | 46 | 0 | 45 | 65 | 85 | 82 | 82 | 82 | 79 | 93 | 93 | 93 | 100 | 85 | 91 | 91 | 91 | 82 | 100 | 100 | 100 | 95 | 84 |
| Rv2239c | - | 158 | 68 | 70 | 70 | 73 | 61 | 68 | 65 | 66 | 83 | 84 | 85 | 85 | 89 | 97 | 96 | 97 | 100 | 88 | 86 | 86 | 86 | 88 | 100 | 100 | 100 | 96 | 88 |
| Rv2240c | - | 265 | 0 | 0 | 0 | 0 | 0 | 0 | 0 | 0 | 46 | 0 | 0 | 0 | 43 | 84 | 85 | 84 | 99 | 62 | 55 | 55 | 55 | 63 | 100 | 100 | 100 | 85 | 65 |
| Rv2241 | *aceE* | 901 | 80 | 81 | 81 | 81 | 80 | 81 | 78 | 76 | 86 | 86 | 87 | 87 | 90 | 96 | 94 | 96 | 99 | 92 | 94 | 94 | 94 | 91 | 100 | 100 | 100 | 94 | 93 |
| Rv2242 | - | 414 | 0 | 0 | 0 | 0 | 0 | 0 | 0 | 56 | 76 | 77 | 77 | 77 | 82 | 93 | 93 | 93 | 100 | 85 | 86 | 85 | 86 | 84 | 100 | 100 | 100 | 93 | 85 |
| Rv2243 | *fabD* | 302 | 0 | 40 | 0 | 0 | 0 | 0 | 0 | 63 | 78 | 81 | 80 | 79 | 80 | 91 | 96 | 91 | 100 | 84 | 84 | 84 | 84 | 84 | 100 | 100 | 100 | 95 | 85 |
| Rv2244 | *acpP* | 115 | 0 | 0 | 0 | 65 | 75 | 0 | 56 | 71 | 88 | 90 | 89 | 89 | 92 | 100 | 99 | 100 | 100 | 94 | 96 | 96 | 96 | 95 | 100 | 100 | 100 | 99 | 94 |
| Rv2245 | *kasA* | 416 | 42 | 40 | 41 | 41 | 42 | 0 | 40 | 61 | 84 | 81 | 83 | 83 | 90 | 96 | 97 | 97 | 100 | 93 | 92 | 92 | 92 | 93 | 100 | 100 | 99 | 96 | 91 |
| Rv2246 | *kasB* | 438 | 43 | 41 | 42 | 42 | 41 | 0 | 42 | 61 | 79 | 76 | 77 | 77 | 80 | 93 | 93 | 93 | 100 | 88 | 90 | 90 | 90 | 90 | 100 | 100 | 100 | 95 | 88 |
| Rv2247 | *accD6* | 473 | 59 | 60 | 60 | 59 | 56 | 59 | 55 | 76 | 83 | 85 | 85 | 85 | 93 | 98 | 99 | 98 | 100 | 94 | 95 | 95 | 95 | 95 | 100 | 100 | 100 | 98 | 95 |
| Rv2248 | - | 271 | 0 | 0 | 45 | 0 | 0 | 0 | 0 | 44 | 50 | 43 | 44 | 45 | 66 | 73 | 88 | 45 | 100 | 77 | 81 | 82 | 82 | 64 | 100 | 100 | 100 | 87 | 79 |
| Rv2249c | *glpD1* | 516 | 50 | 48 | 0 | 44 | 0 | 50 | 0 | 64 | 73 | 77 | 77 | 77 | 70 | 88 | 92 | 88 | 100 | 84 | 83 | 83 | 83 | 84 | 100 | 100 | 100 | 91 | 83 |
| Rv2250A | - | 139 | 0 | 0 | 0 | 0 | 0 | 0 | 0 | 54 | 65 | 68 | 64 | 65 | 64 | 78 | 88 | 78 | 100 | 76 | 72 | 72 | 72 | 79 | 100 | 100 | 100 | 89 | 77 |
| Rv2250c | - | 189 | 0 | 0 | 0 | 0 | 0 | 0 | 0 | 52 | 62 | 63 | 64 | 64 | 62 | 84 | 87 | 85 | 99 | 73 | 76 | 76 | 76 | 80 | 100 | 100 | 100 | 87 | 70 |
| Rv2251 | - | 475 | 0 | 0 | 0 | 0 | 0 | 39 | 0 | 69 | 76 | 78 | 75 | 80 | 74 | 85 | 91 | 84 | 98 | 86 | 87 | 87 | 87 | 86 | 98 | 98 | 98 | 91 | 87 |
| Rv2252 | - | 309 | 0 | 0 | 0 | 0 | 0 | 0 | 0 | 60 | 56 | 69 | 69 | 69 | 64 | 88 | 88 | 88 | 100 | 79 | 81 | 81 | 81 | 78 | 97 | 100 | 100 | 86 | 80 |
| Rv2253 | - | 167 | 0 | 0 | 0 | 0 | 0 | 0 | 0 | 0 | 0 | 58 | 58 | 56 | 0 | 0 | 77 | 0 | 100 | 57 | 57 | 57 | 57 | 0 | 100 | 100 | 100 | 85 | 55 |
| Rv2254c | - | 151 | 0 | 0 | 0 | 0 | 0 | 0 | 0 | 0 | 0 | 0 | 0 | 0 | 0 | 0 | 65 | 0 | 99 | 0 | 0 | 0 | 0 | 0 | 100 | 100 | 100 | 65 | 0 |
| Rv2255c | - | 64 | 0 | 0 | 0 | 0 | 0 | 0 | 0 | 0 | 0 | 0 | 0 | 0 | 0 | 0 | 0 | 0 | 100 | 0 | 0 | 0 | 0 | 0 | 100 | 100 | 100 | 0 | 0 |
| Rv2256c | - | 177 | 0 | 0 | 0 | 0 | 0 | 0 | 0 | 62 | 72 | 78 | 81 | 81 | 79 | 97 | 94 | 97 | 99 | 91 | 92 | 92 | 92 | 92 | 100 | 100 | 100 | 94 | 92 |
| Rv2257c | - | 272 | 58 | 65 | 64 | 61 | 56 | 59 | 55 | 0 | 72 | 67 | 69 | 70 | 76 | 85 | 89 | 85 | 100 | 78 | 81 | 81 | 81 | 84 | 100 | 100 | 100 | 88 | 79 |
| Rv2258c | - | 353 | 0 | 0 | 0 | 0 | 0 | 0 | 0 | 0 | 45 | 41 | 44 | 42 | 78 | 85 | 87 | 86 | 100 | 81 | 82 | 82 | 82 | 81 | 100 | 100 | 100 | 87 | 80 |
| Rv2259 | *adhE2* | 361 | 78 | 45 | 77 | 78 | 80 | 77 | 81 | 81 | 90 | 87 | 87 | 88 | 89 | 94 | 95 | 95 | 100 | 90 | 91 | 91 | 91 | 91 | 100 | 100 | 100 | 95 | 90 |
| Rv2260 | - | 211 | 59 | 46 | 69 | 70 | 60 | 70 | 71 | 69 | 79 | 77 | 78 | 78 | 86 | 90 | 90 | 90 | 100 | 88 | 90 | 90 | 90 | 82 | 100 | 100 | 100 | 91 | 89 |
| Rv2261c | - | 140 | 0 | 0 | 0 | 0 | 0 | 0 | 0 | 0 | 0 | 0 | 0 | 0 | 0 | 0 | 81 | 0 | 100 | 0 | 68 | 68 | 68 | 0 | 100 | 100 | 100 | 80 | 0 |
| Rv2262c | - | 360 | 0 | 0 | 0 | 0 | 0 | 0 | 0 | 37 | 39 | 41 | 41 | 41 | 0 | 0 | 76 | 0 | 100 | 39 | 75 | 75 | 75 | 0 | 100 | 100 | 100 | 75 | 0 |

| | | | | | | | | | | | | | | | | | | | | | | | | | | | | | |
|---|---|---|---|---|---|---|---|---|---|---|---|---|---|---|---|---|---|---|---|---|---|---|---|---|---|---|---|---|---|
| Rv2263 | - | 317 | 44 | 45 | 50 | 44 | 51 | 43 | 45 | 50 | 57 | 56 | 55 | 55 | 66 | 76 | 94 | 76 | 100 | 60 | 62 | 62 | 62 | 70 | 100 | 100 | 100 | 94 | 68 |
| Rv2264c | - | 592 | 44 | 38 | 36 | 36 | 35 | 39 | 38 | 35 | 42 | 39 | 43 | 43 | 52 | 62 | 73 | 61 | 100 | 51 | 57 | 57 | 57 | 56 | 100 | 100 | 100 | 75 | 51 |
| Rv2265 | - | 409 | 0 | 0 | 0 | 0 | 0 | 0 | 0 | 61 | 0 | 0 | 0 | 0 | 79 | 0 | 41 | 0 | 100 | 0 | 42 | 43 | 43 | 0 | 100 | 100 | 100 | 41 | 73 |
| Rv2266 | *cyp124* | 428 | 0 | 0 | 47 | 48 | 45 | 0 | 0 | 57 | 61 | 61 | 59 | 60 | 59 | 93 | 92 | 93 | 100 | 84 | 86 | 86 | 86 | 84 | 99 | 100 | 100 | 91 | 83 |
| Rv2267c | - | 388 | 0 | 0 | 0 | 0 | 0 | 0 | 0 | 0 | 39 | 41 | 39 | 40 | 40 | 0 | 40 | 0 | 100 | 38 | 38 | 38 | 38 | 38 | 100 | 100 | 100 | 40 | 40 |
| Rv2268c | *cyp128* | 489 | 0 | 0 | 42 | 44 | 39 | 0 | 0 | 50 | 46 | 46 | 42 | 42 | 47 | 51 | 50 | 51 | 100 | 49 | 48 | 46 | 46 | 47 | 100 | 100 | 100 | 46 | 51 |
| Rv2269c | - | 110 | 0 | 0 | 0 | 0 | 0 | 0 | 0 | 0 | 0 | 0 | 0 | 0 | 0 | 0 | 0 | 0 | 100 | 0 | 0 | 0 | 0 | 0 | 100 | 100 | 100 | 0 | 0 |
| Rv2270 | *lppN* | 175 | 0 | 0 | 0 | 0 | 0 | 0 | 0 | 0 | 0 | 0 | 0 | 0 | 0 | 78 | 78 | 79 | 99 | 0 | 0 | 0 | 0 | 0 | 100 | 100 | 100 | 79 | 0 |
| Rv2271 | - | 99 | 0 | 0 | 0 | 0 | 0 | 0 | 0 | 0 | 0 | 0 | 0 | 0 | 0 | 0 | 0 | 0 | 100 | 0 | 0 | 0 | 0 | 0 | 100 | 100 | 100 | 0 | 0 |
| Rv2272 | - | 122 | 57 | 0 | 51 | 50 | 54 | 0 | 0 | 62 | 57 | 58 | 63 | 63 | 62 | 0 | 87 | 0 | 100 | 58 | 59 | 59 | 59 | 75 | 100 | 100 | 100 | 85 | 59 |
| Rv2273 | - | 109 | 0 | 0 | 0 | 0 | 0 | 0 | 0 | 0 | 0 | 0 | 0 | 0 | 0 | 0 | 81 | 0 | 100 | 0 | 0 | 0 | 0 | 52 | 100 | 100 | 100 | 80 | 0 |
| Rv2274c | - | 105 | 0 | 0 | 0 | 0 | 0 | 0 | 0 | 0 | 0 | 0 | 0 | 0 | 0 | 0 | 0 | 0 | 100 | 0 | 0 | 0 | 0 | 0 | 100 | 100 | 100 | 0 | 0 |
| Rv2275 | - | 289 | 0 | 0 | 0 | 0 | 0 | 0 | 0 | 0 | 0 | 0 | 0 | 0 | 0 | 0 | 0 | 0 | 100 | 0 | 0 | 0 | 0 | 0 | 100 | 100 | 100 | 0 | 0 |
| Rv2276 | *cyp121* | 396 | 0 | 0 | 43 | 42 | 39 | 0 | 0 | 48 | 47 | 43 | 44 | 45 | 44 | 44 | 45 | 44 | 100 | 44 | 47 | 46 | 46 | 47 | 100 | 100 | 100 | 45 | 44 |
| Rv2277c | - | 301 | 0 | 0 | 0 | 0 | 0 | 0 | 0 | 0 | 0 | 0 | 0 | 0 | 0 | 0 | 0 | 0 | 99 | 0 | 0 | 0 | 0 | 0 | 100 | 100 | 100 | 82 | 0 |
| Rv2278 | - | 108 | 65 | 0 | 66 | 0 | 61 | 0 | 60 | 76 | 0 | 0 | 0 | 0 | 59 | 63 | 82 | 66 | 100 | 82 | 96 | 77 | 77 | 84 | 100 | 100 | 100 | 0 | 62 |
| Rv2279 | - | 312 | 58 | 49 | 61 | 51 | 63 | 0 | 61 | 56 | 0 | 0 | 51 | 52 | 66 | 59 | 66 | 66 | 100 | 66 | 90 | 67 | 67 | 66 | 100 | 100 | 100 | 0 | 65 |
| Rv2280 | - | 459 | 0 | 0 | 40 | 42 | 0 | 47 | 0 | 50 | 51 | 52 | 51 | 51 | 51 | 51 | 51 | 51 | 100 | 51 | 49 | 49 | 49 | 50 | 100 | 100 | 100 | 51 | 50 |
| Rv2281 | *pitB* | 552 | 77 | 79 | 51 | 52 | 79 | 54 | 75 | 47 | 52 | 56 | 59 | 57 | 56 | 53 | 94 | 53 | 100 | 56 | 56 | 56 | 56 | 55 | 100 | 100 | 100 | 93 | 55 |
| Rv2282c | - | 312 | 41 | 54 | 44 | 55 | 0 | 50 | 47 | 42 | 56 | 61 | 59 | 58 | 57 | 88 | 88 | 88 | 100 | 45 | 41 | 41 | 41 | 76 | 100 | 100 | 100 | 87 | 43 |
| Rv2283 | - | 64 | 0 | 0 | 0 | 0 | 0 | 0 | 0 | 0 | 0 | 0 | 0 | 0 | 0 | 0 | 0 | 0 | 100 | 0 | 0 | 0 | 0 | 0 | 100 | 100 | 100 | 0 | 0 |
| Rv2284 | *lipM* | 431 | 0 | 0 | 0 | 0 | 0 | 0 | 0 | 58 | 0 | 0 | 55 | 55 | 49 | 87 | 88 | 87 | 100 | 60 | 60 | 60 | 60 | 61 | 100 | 100 | 100 | 87 | 61 |
| Rv2285 | - | 445 | 0 | 0 | 0 | 0 | 0 | 0 | 0 | 49 | 49 | 49 | 48 | 49 | 44 | 49 | 92 | 49 | 100 | 52 | 53 | 53 | 53 | 50 | 100 | 100 | 100 | 92 | 53 |
| Rv2286c | - | 230 | 42 | 43 | 42 | 45 | 41 | 45 | 0 | 45 | 45 | 47 | 46 | 47 | 50 | 46 | 91 | 46 | 99 | 46 | 74 | 74 | 74 | 46 | 100 | 100 | 100 | 91 | 75 |
| Rv2287 | *yjcE* | 542 | 0 | 46 | 0 | 48 | 0 | 48 | 0 | 50 | 46 | 55 | 52 | 52 | 52 | 83 | 84 | 84 | 100 | 47 | 52 | 52 | 52 | 53 | 100 | 100 | 100 | 90 | 48 |
| Rv2288 | - | 125 | 0 | 0 | 0 | 0 | 0 | 0 | 0 | 0 | 0 | 0 | 0 | 0 | 0 | 0 | 0 | 0 | 100 | 0 | 0 | 0 | 0 | 0 | 100 | 100 | 100 | 0 | 0 |
| Rv2289 | *cdh* | 260 | 0 | 0 | 0 | 0 | 0 | 0 | 0 | 0 | 0 | 0 | 0 | 0 | 0 | 0 | 80 | 0 | 100 | 0 | 0 | 0 | 0 | 0 | 100 | 100 | 100 | 80 | 0 |
| Rv2290 | *lppO* | 171 | 0 | 0 | 0 | 0 | 0 | 0 | 0 | 0 | 0 | 0 | 0 | 0 | 0 | 72 | 0 | 73 | 97 | 0 | 0 | 0 | 0 | 0 | 100 | 100 | 100 | 0 | 0 |
| Rv2291 | *sseB* | 284 | 47 | 49 | 50 | 48 | 50 | 53 | 52 | 61 | 63 | 61 | 59 | 59 | 63 | 80 | 80 | 81 | 99 | 70 | 73 | 74 | 74 | 73 | 100 | 100 | 100 | 41 | 71 |
| Rv2292c | - | 74 | 0 | 0 | 0 | 0 | 0 | 0 | 0 | 0 | 0 | 0 | 0 | 0 | 0 | 0 | 0 | 0 | 100 | 0 | 0 | 0 | 0 | 0 | 100 | 100 | 100 | 0 | 0 |
| Rv2293c | - | 246 | 0 | 0 | 0 | 0 | 0 | 0 | 0 | 0 | 0 | 0 | 0 | 0 | 0 | 0 | 0 | 0 | 92 | 0 | 0 | 0 | 0 | 0 | 100 | 100 | 100 | 0 | 0 |
| Rv2294 | - | 407 | 49 | 50 | 47 | 46 | 51 | 47 | 51 | 50 | 39 | 42 | 41 | 41 | 41 | 86 | 87 | 86 | 100 | 42 | 42 | 42 | 42 | 42 | 100 | 100 | 100 | 86 | 43 |
| Rv2295 | - | 212 | 0 | 0 | 0 | 0 | 0 | 0 | 0 | 0 | 0 | 0 | 0 | 0 | 0 | 0 | 0 | 0 | 100 | 0 | 0 | 0 | 0 | 0 | 100 | 100 | 100 | 0 | 0 |
| Rv2296 | - | 300 | 0 | 0 | 0 | 0 | 0 | 0 | 0 | 0 | 43 | 61 | 57 | 56 | 51 | 89 | 90 | 89 | 100 | 64 | 65 | 65 | 65 | 64 | 100 | 100 | 100 | 44 | 67 |
| Rv2297 | - | 150 | 0 | 0 | 0 | 0 | 0 | 0 | 0 | 0 | 0 | 0 | 0 | 0 | 0 | 0 | 0 | 0 | 100 | 0 | 0 | 0 | 0 | 0 | 100 | 100 | 100 | 0 | 0 |
| Rv2298 | - | 323 | 0 | 46 | 46 | 46 | 0 | 47 | 0 | 48 | 49 | 45 | 48 | 45 | 48 | 89 | 94 | 90 | 100 | 0 | 47 | 47 | 47 | 48 | 100 | 100 | 100 | 93 | 44 |
| Rv2299c | *htpG* | 647 | 0 | 0 | 0 | 43 | 0 | 0 | 0 | 0 | 83 | 85 | 87 | 87 | 0 | 94 | 93 | 94 | 100 | 90 | 0 | 0 | 0 | 0 | 100 | 100 | 100 | 92 | 90 |
| Rv2300c | - | 310 | 0 | 0 | 0 | 0 | 0 | 0 | 0 | 0 | 0 | 0 | 0 | 64 | 0 | 0 | 87 | 0 | 100 | 73 | 0 | 0 | 0 | 0 | 100 | 100 | 100 | 85 | 74 |

| | | | | | | | | | | | | | | | | | | | | | | | | | | | | | |
|---|---|---|---|---|---|---|---|---|---|---|---|---|---|---|---|---|---|---|---|---|---|---|---|---|---|---|---|---|---|
| Rv2301 | *cut2* | 230 | 0 | 0 | 0 | 0 | 0 | 0 | 0 | 0 | 0 | 0 | 47 | 0 | 63 | 81 | 82 | 82 | 100 | 79 | 79 | 79 | 79 | 79 | 100 | 100 | 100 | 59 | 73 |
| Rv2302 | - | 80 | 0 | 0 | 0 | 0 | 0 | 0 | 0 | 67 | 0 | 69 | 0 | 0 | 0 | 0 | 0 | 0 | 100 | 0 | 75 | 75 | 75 | 82 | 100 | 100 | 100 | 0 | 0 |
| Rv2303c | - | 307 | 0 | 0 | 0 | 44 | 0 | 0 | 0 | 0 | 0 | 0 | 45 | 48 | 0 | 50 | 48 | 49 | 100 | 47 | 43 | 43 | 43 | 47 | 100 | 100 | 100 | 48 | 45 |
| Rv2304c | - | 69 | 0 | 0 | 0 | 0 | 0 | 0 | 0 | 0 | 0 | 0 | 0 | 0 | 0 | 0 | 0 | 0 | 100 | 0 | 0 | 0 | 0 | 0 | 100 | 100 | 100 | 0 | 0 |
| Rv2305 | - | 429 | 0 | 0 | 0 | 0 | 0 | 0 | 0 | 0 | 0 | 0 | 0 | 0 | 0 | 0 | 0 | 0 | 99 | 0 | 0 | 0 | 0 | 0 | 100 | 100 | 100 | 0 | 0 |
| Rv2306A | - | 197 | 0 | 0 | 0 | 0 | 0 | 0 | 0 | 0 | 0 | 0 | 0 | 0 | 0 | 62 | 85 | 63 | 100 | 74 | 82 | 82 | 82 | 79 | 100 | 100 | 100 | 0 | 69 |
| Rv2306B | - | 144 | 0 | 0 | 0 | 0 | 0 | 0 | 0 | 0 | 0 | 0 | 0 | 0 | 0 | 86 | 76 | 88 | 100 | 85 | 86 | 86 | 86 | 77 | 100 | 100 | 100 | 0 | 86 |
| Rv2307A | - | 63 | 0 | 0 | 0 | 0 | 0 | 0 | 0 | 0 | 0 | 0 | 0 | 0 | 0 | 0 | 0 | 0 | 100 | 0 | 0 | 0 | 0 | 0 | 100 | 100 | 100 | 0 | 0 |
| Rv2307B | - | 143 | 0 | 0 | 0 | 0 | 0 | 0 | 0 | 0 | 0 | 0 | 0 | 0 | 0 | 0 | 0 | 0 | 100 | 0 | 0 | 0 | 0 | 0 | 100 | 100 | 100 | 48 | 46 |
| Rv2307D | - | 60 | 0 | 0 | 0 | 0 | 0 | 0 | 0 | 0 | 0 | 0 | 0 | 0 | 0 | 0 | 0 | 0 | 100 | 0 | 0 | 0 | 0 | 0 | 100 | 100 | 100 | 0 | 0 |
| Rv2307c | - | 281 | 0 | 0 | 0 | 0 | 0 | 0 | 0 | 43 | 0 | 0 | 60 | 59 | 0 | 0 | 0 | 0 | 99 | 51 | 82 | 82 | 82 | 46 | 99 | 100 | 100 | 0 | 0 |
| Rv2308 | - | 238 | 0 | 0 | 0 | 0 | 0 | 0 | 0 | 0 | 0 | 0 | 0 | 0 | 0 | 0 | 0 | 0 | 99 | 0 | 0 | 0 | 0 | 0 | 99 | 100 | 100 | 0 | 0 |
| Rv2309A | - | 95 | 0 | 0 | 0 | 0 | 0 | 0 | 0 | 0 | 0 | 0 | 0 | 0 | 0 | 0 | 0 | 0 | 100 | 0 | 0 | 0 | 0 | 0 | 100 | 100 | 100 | 0 | 0 |
| Rv2309c | - | 151 | 52 | 54 | 0 | 52 | 0 | 53 | 0 | 75 | 56 | 54 | 0 | 59 | 0 | 52 | 53 | 0 | 100 | 57 | 82 | 61 | 56 | 0 | 100 | 100 | 100 | 55 | 0 |
| Rv2310 | - | 114 | 56 | 62 | 0 | 0 | 0 | 0 | 0 | 0 | 60 | 0 | 64 | 77 | 0 | 0 | 0 | 0 | 100 | 0 | 0 | 0 | 0 | 0 | 100 | 100 | 100 | 67 | 0 |
| Rv2311 | - | 174 | 0 | 0 | 0 | 0 | 0 | 0 | 0 | 51 | 0 | 0 | 0 | 0 | 0 | 0 | 0 | 0 | 100 | 0 | 0 | 56 | 56 | 0 | 100 | 100 | 100 | 0 | 55 |
| Rv2312 | - | 89 | 0 | 0 | 0 | 0 | 0 | 0 | 0 | 0 | 0 | 0 | 0 | 0 | 0 | 0 | 0 | 0 | 100 | 83 | 0 | 81 | 81 | 0 | 100 | 100 | 100 | 0 | 0 |
| Rv2313c | - | 284 | 0 | 0 | 0 | 0 | 0 | 0 | 0 | 0 | 0 | 0 | 0 | 0 | 0 | 80 | 84 | 80 | 100 | 68 | 72 | 72 | 72 | 66 | 100 | 100 | 100 | 87 | 69 |
| Rv2314c | - | 457 | 0 | 0 | 0 | 0 | 0 | 0 | 0 | 64 | 76 | 75 | 75 | 74 | 75 | 89 | 89 | 89 | 100 | 81 | 85 | 84 | 84 | 81 | 100 | 100 | 100 | 89 | 81 |
| Rv2315c | - | 505 | 0 | 0 | 0 | 0 | 0 | 0 | 0 | 71 | 82 | 82 | 85 | 84 | 86 | 91 | 92 | 91 | 100 | 85 | 89 | 89 | 89 | 89 | 100 | 100 | 100 | 92 | 88 |
| Rv2316 | *uspA* | 290 | 48 | 44 | 54 | 56 | 0 | 53 | 52 | 61 | 45 | 55 | 53 | 53 | 85 | 93 | 94 | 94 | 100 | 50 | 54 | 54 | 54 | 91 | 100 | 100 | 100 | 92 | 50 |
| Rv2317 | *uspB* | 274 | 48 | 46 | 55 | 55 | 0 | 62 | 48 | 59 | 47 | 52 | 52 | 56 | 85 | 94 | 93 | 93 | 100 | 50 | 48 | 48 | 48 | 86 | 100 | 100 | 100 | 91 | 50 |
| Rv2318 | *uspC* | 440 | 0 | 0 | 0 | 0 | 0 | 0 | 0 | 47 | 0 | 0 | 0 | 0 | 72 | 84 | 87 | 84 | 100 | 0 | 0 | 0 | 0 | 78 | 100 | 100 | 100 | 87 | 0 |
| Rv2319c | - | 292 | 48 | 50 | 51 | 50 | 53 | 48 | 48 | 0 | 0 | 53 | 56 | 52 | 72 | 0 | 85 | 0 | 100 | 54 | 53 | 53 | 53 | 70 | 100 | 100 | 100 | 85 | 43 |
| Rv2320c | *rocE* | 476 | 41 | 42 | 40 | 40 | 42 | 46 | 43 | 57 | 58 | 75 | 68 | 68 | 86 | 90 | 89 | 91 | 100 | 56 | 56 | 56 | 56 | 82 | 100 | 100 | 100 | 89 | 56 |
| Rv2321c | *rocD2* | 181 | 50 | 53 | 52 | 55 | 47 | 48 | 45 | 67 | 50 | 52 | 65 | 64 | 77 | 80 | 84 | 80 | 100 | 78 | 77 | 77 | 77 | 82 | 100 | 100 | 100 | 84 | 80 |
| Rv2322c | *rocD1* | 221 | 45 | 40 | 42 | 41 | 41 | 0 | 44 | 68 | 47 | 47 | 65 | 65 | 78 | 81 | 83 | 82 | 100 | 76 | 77 | 77 | 77 | 79 | 100 | 100 | 100 | 83 | 79 |
| Rv2323c | - | 302 | 0 | 0 | 0 | 0 | 0 | 0 | 0 | 0 | 0 | 0 | 63 | 63 | 74 | 81 | 81 | 81 | 99 | 76 | 73 | 73 | 73 | 73 | 100 | 100 | 100 | 81 | 77 |
| Rv2324 | - | 148 | 0 | 0 | 0 | 0 | 48 | 0 | 0 | 51 | 53 | 53 | 65 | 65 | 86 | 94 | 93 | 92 | 100 | 92 | 93 | 93 | 93 | 91 | 100 | 100 | 100 | 92 | 94 |
| Rv2325c | - | 282 | 0 | 0 | 0 | 0 | 0 | 0 | 0 | 0 | 58 | 61 | 43 | 43 | 73 | 86 | 89 | 86 | 100 | 76 | 79 | 79 | 79 | 74 | 100 | 100 | 100 | 89 | 78 |
| Rv2326c | - | 697 | 44 | 47 | 46 | 49 | 42 | 42 | 41 | 42 | 61 | 61 | 48 | 47 | 72 | 82 | 90 | 81 | 100 | 76 | 80 | 80 | 80 | 81 | 100 | 100 | 100 | 90 | 80 |
| Rv2327 | - | 163 | 0 | 0 | 0 | 0 | 0 | 45 | 0 | 51 | 50 | 53 | 50 | 50 | 63 | 88 | 91 | 88 | 100 | 79 | 76 | 77 | 77 | 82 | 100 | 100 | 100 | 91 | 79 |
| Rv2328 | *PE23* | 382 | 0 | 0 | 0 | 0 | 0 | 0 | 0 | 0 | 0 | 0 | 0 | 0 | 0 | 56 | 51 | 51 | 100 | 0 | 0 | 0 | 0 | 0 | 100 | 100 | 100 | 54 | 0 |
| Rv2329c | *narK1* | 515 | 0 | 45 | 44 | 45 | 0 | 0 | 0 | 51 | 69 | 70 | 69 | 70 | 67 | 71 | 83 | 71 | 100 | 69 | 76 | 76 | 76 | 78 | 100 | 100 | 100 | 76 | 52 |
| Rv2330c | *lppP* | 175 | 0 | 0 | 0 | 0 | 0 | 0 | 0 | 0 | 0 | 0 | 0 | 0 | 0 | 82 | 82 | 82 | 100 | 58 | 68 | 68 | 68 | 63 | 100 | 100 | 100 | 81 | 58 |
| Rv2331 | - | 128 | 0 | 0 | 0 | 0 | 0 | 0 | 0 | 0 | 0 | 0 | 0 | 0 | 0 | 0 | 0 | 0 | 100 | 0 | 0 | 0 | 0 | 0 | 100 | 100 | 100 | 0 | 0 |
| Rv2331A | - | 112 | 0 | 0 | 0 | 0 | 0 | 0 | 0 | 0 | 0 | 0 | 0 | 0 | 0 | 68 | 68 | 68 | 100 | 0 | 0 | 0 | 0 | 0 | 100 | 100 | 100 | 0 | 0 |
| Rv2332 | *mez* | 548 | 0 | 0 | 42 | 43 | 0 | 70 | 0 | 41 | 42 | 42 | 43 | 43 | 42 | 44 | 88 | 44 | 100 | 42 | 42 | 42 | 42 | 43 | 100 | 100 | 100 | 88 | 41 |

| | | | | | | | | | | | | | | | | | | | | | | | | | | | | | |
|---|---|---|---|---|---|---|---|---|---|---|---|---|---|---|---|---|---|---|---|---|---|---|---|---|---|---|---|---|---|
| Rv2333c | - | 537 | 47 | 51 | 57 | 55 | 52 | 55 | 52 | 52 | 52 | 60 | 53 | 53 | 56 | 47 | 89 | 53 | 99 | 56 | 56 | 55 | 55 | 57 | 99 | 100 | 100 | 87 | 56 |
| Rv2334 | *cysK1* | 310 | 74 | 74 | 77 | 76 | 73 | 73 | 75 | 81 | 62 | 79 | 61 | 61 | 62 | 94 | 94 | 94 | 100 | 61 | 61 | 61 | 61 | 60 | 100 | 100 | 98 | 93 | 61 |
| Rv2335 | *cysE* | 229 | 69 | 72 | 69 | 71 | 75 | 73 | 72 | 75 | 0 | 73 | 55 | 53 | 0 | 86 | 85 | 85 | 100 | 0 | 49 | 0 | 0 | 0 | 100 | 100 | 100 | 84 | 0 |
| Rv2336 | - | 322 | 0 | 0 | 0 | 0 | 0 | 0 | 0 | 0 | 0 | 0 | 0 | 0 | 0 | 0 | 0 | 0 | 100 | 0 | 0 | 0 | 0 | 0 | 100 | 100 | 100 | 0 | 0 |
| Rv2337c | - | 372 | 0 | 0 | 0 | 0 | 0 | 0 | 0 | 0 | 40 | 0 | 0 | 0 | 0 | 0 | 0 | 0 | 99 | 0 | 0 | 0 | 0 | 0 | 99 | 100 | 99 | 0 | 0 |
| Rv2338c | *moeW* | 318 | 42 | 49 | 43 | 50 | 48 | 46 | 46 | 53 | 0 | 0 | 51 | 50 | 56 | 0 | 54 | 0 | 100 | 54 | 56 | 56 | 56 | 0 | 100 | 100 | 100 | 54 | 0 |
| Rv2339 | *mmpL9* | 962 | 46 | 46 | 44 | 44 | 40 | 60 | 44 | 50 | 57 | 56 | 55 | 54 | 75 | 78 | 75 | 75 | 99 | 77 | 77 | 77 | 77 | 78 | 100 | 100 | 100 | 77 | 77 |
| Rv2340c | *PE_PGRS39* | 413 | 40 | 0 | 38 | 38 | 35 | 0 | 36 | 34 | 46 | 38 | 39 | 35 | 38 | 41 | 56 | 49 | 100 | 37 | 36 | 36 | 36 | 39 | 100 | 100 | 100 | 56 | 35 |
| Rv2341 | *lppQ* | 139 | 0 | 0 | 0 | 0 | 0 | 0 | 0 | 0 | 0 | 0 | 0 | 0 | 0 | 0 | 0 | 0 | 100 | 0 | 0 | 0 | 0 | 0 | 100 | 100 | 100 | 0 | 0 |
| Rv2342 | - | 85 | 0 | 0 | 0 | 0 | 60 | 66 | 57 | 0 | 67 | 0 | 0 | 0 | 70 | 89 | 86 | 91 | 100 | 77 | 79 | 79 | 79 | 83 | 100 | 100 | 100 | 86 | 78 |
| Rv2343c | *dnaG* | 639 | 73 | 73 | 73 | 73 | 71 | 70 | 70 | 61 | 78 | 80 | 82 | 82 | 86 | 92 | 92 | 92 | 100 | 86 | 87 | 87 | 87 | 89 | 100 | 100 | 100 | 91 | 87 |
| Rv2344c | *dgt* | 431 | 70 | 66 | 70 | 67 | 67 | 68 | 67 | 60 | 75 | 77 | 75 | 75 | 79 | 93 | 93 | 93 | 100 | 82 | 83 | 83 | 83 | 83 | 99 | 100 | 100 | 90 | 83 |
| Rv2345 | - | 660 | 51 | 49 | 52 | 48 | 49 | 45 | 0 | 40 | 60 | 60 | 61 | 61 | 64 | 80 | 86 | 81 | 100 | 70 | 76 | 76 | 76 | 72 | 100 | 100 | 100 | 85 | 71 |
| Rv2346c | *esxO* | 94 | 0 | 0 | 0 | 0 | 0 | 0 | 0 | 0 | 0 | 0 | 0 | 0 | 0 | 95 | 97 | 95 | 97 | 0 | 0 | 0 | 0 | 0 | 100 | 100 | 100 | 97 | 0 |
| Rv2347c | *esxP* | 98 | 0 | 0 | 0 | 0 | 0 | 0 | 0 | 0 | 0 | 0 | 0 | 0 | 0 | 93 | 96 | 93 | 98 | 0 | 0 | 0 | 0 | 0 | 100 | 100 | 100 | 96 | 0 |
| Rv2348c | - | 108 | 0 | 0 | 0 | 0 | 0 | 0 | 0 | 0 | 0 | 0 | 0 | 0 | 0 | 60 | 60 | 60 | 0 | 0 | 0 | 0 | 0 | 0 | 100 | 100 | 100 | 0 | 0 |
| Rv2349c | *plcC* | 508 | 0 | 0 | 0 | 0 | 0 | 0 | 0 | 40 | 0 | 46 | 68 | 68 | 51 | 0 | 92 | 0 | 79 | 0 | 0 | 0 | 0 | 0 | 100 | 100 | 100 | 78 | 0 |
| Rv2350c | *plcB* | 512 | 0 | 0 | 0 | 0 | 0 | 0 | 0 | 42 | 0 | 46 | 68 | 68 | 51 | 0 | 88 | 0 | 80 | 0 | 0 | 0 | 0 | 0 | 100 | 100 | 100 | 83 | 0 |
| Rv2351c | *plcA* | 512 | 0 | 0 | 0 | 0 | 0 | 0 | 0 | 40 | 0 | 45 | 68 | 68 | 50 | 0 | 92 | 0 | 81 | 0 | 0 | 0 | 0 | 0 | 100 | 100 | 100 | 82 | 0 |
| Rv2352c | *PPE38* | 391 | 0 | 0 | 0 | 0 | 38 | 0 | 36 | 0 | 40 | 44 | 39 | 39 | 39 | 55 | 85 | 58 | 73 | 39 | 41 | 41 | 41 | 43 | 100 | 100 | 100 | 75 | 39 |
| Rv2353c | *PPE39* | 354 | 40 | 0 | 0 | 0 | 0 | 52 | 0 | 0 | 41 | 42 | 37 | 0 | 42 | 0 | 0 | 0 | 75 | 36 | 57 | 45 | 45 | 39 | 100 | 100 | 100 | 68 | 37 |
| Rv2354 | - | 108 | 65 | 0 | 66 | 0 | 61 | 0 | 60 | 76 | 0 | 0 | 0 | 0 | 59 | 63 | 66 | 66 | 100 | 82 | 96 | 77 | 77 | 84 | 100 | 100 | 100 | 0 | 62 |
| Rv2355 | - | 312 | 58 | 49 | 61 | 51 | 63 | 0 | 61 | 56 | 0 | 0 | 51 | 52 | 66 | 59 | 66 | 66 | 100 | 66 | 90 | 67 | 67 | 66 | 100 | 100 | 100 | 0 | 65 |
| Rv2356c | *PPE40* | 615 | 37 | 0 | 0 | 0 | 0 | 0 | 0 | 0 | 36 | 37 | 37 | 35 | 46 | 65 | 80 | 65 | 99 | 38 | 37 | 35 | 35 | 34 | 100 | 100 | 99 | 70 | 35 |
| Rv2357c | *glyS* | 463 | 85 | 83 | 85 | 85 | 84 | 84 | 84 | 83 | 88 | 86 | 87 | 87 | 88 | 94 | 96 | 94 | 100 | 90 | 90 | 90 | 90 | 91 | 100 | 100 | 100 | 96 | 90 |
| Rv2358 | - | 135 | 63 | 63 | 64 | 66 | 0 | 0 | 0 | 0 | 87 | 82 | 80 | 81 | 88 | 79 | 79 | 79 | 100 | 85 | 78 | 78 | 78 | 91 | 100 | 100 | 100 | 85 | 85 |
| Rv2359 | *furB* | 130 | 66 | 65 | 67 | 73 | 69 | 68 | 73 | 78 | 84 | 82 | 83 | 82 | 82 | 93 | 92 | 93 | 99 | 86 | 85 | 85 | 85 | 87 | 100 | 100 | 100 | 93 | 90 |
| Rv2360c | - | 142 | 0 | 0 | 0 | 0 | 0 | 0 | 0 | 0 | 54 | 56 | 55 | 53 | 58 | 87 | 85 | 86 | 99 | 71 | 68 | 68 | 68 | 67 | 99 | 100 | 100 | 85 | 75 |
| Rv2361c | - | 296 | 77 | 77 | 79 | 79 | 76 | 76 | 75 | 77 | 74 | 83 | 81 | 80 | 76 | 91 | 90 | 90 | 100 | 86 | 85 | 85 | 85 | 86 | 100 | 100 | 100 | 94 | 85 |
| Rv2362c | *recO* | 265 | 63 | 61 | 64 | 64 | 62 | 55 | 61 | 66 | 79 | 85 | 87 | 86 | 88 | 92 | 92 | 92 | 100 | 88 | 90 | 90 | 90 | 90 | 100 | 100 | 100 | 96 | 88 |
| Rv2363 | *amiA2* | 484 | 46 | 45 | 46 | 45 | 53 | 46 | 52 | 54 | 48 | 71 | 71 | 70 | 75 | 92 | 92 | 92 | 100 | 81 | 83 | 83 | 83 | 84 | 100 | 100 | 100 | 92 | 82 |
| Rv2364c | *era* | 300 | 75 | 78 | 75 | 74 | 78 | 75 | 73 | 77 | 87 | 86 | 86 | 86 | 88 | 94 | 93 | 94 | 100 | 88 | 91 | 91 | 91 | 91 | 100 | 100 | 100 | 92 | 88 |
| Rv2365c | - | 113 | 0 | 0 | 0 | 0 | 0 | 0 | 0 | 67 | 76 | 0 | 73 | 76 | 85 | 0 | 0 | 0 | 100 | 82 | 80 | 80 | 80 | 88 | 100 | 100 | 100 | 70 | 84 |
| Rv2366c | - | 435 | 48 | 70 | 75 | 73 | 73 | 70 | 65 | 71 | 81 | 82 | 84 | 84 | 88 | 91 | 90 | 91 | 100 | 86 | 87 | 87 | 87 | 86 | 100 | 100 | 100 | 91 | 87 |
| Rv2367c | - | 182 | 74 | 82 | 77 | 77 | 77 | 78 | 75 | 69 | 85 | 86 | 86 | 86 | 87 | 97 | 97 | 97 | 100 | 94 | 93 | 93 | 93 | 94 | 100 | 100 | 100 | 97 | 94 |
| Rv2368c | *phoH1* | 352 | 75 | 76 | 77 | 78 | 75 | 73 | 75 | 81 | 85 | 82 | 85 | 85 | 87 | 95 | 95 | 95 | 100 | 88 | 89 | 89 | 89 | 90 | 100 | 100 | 100 | 95 | 88 |
| Rv2369c | - | 100 | 0 | 0 | 0 | 0 | 0 | 0 | 0 | 0 | 0 | 0 | 0 | 0 | 0 | 0 | 0 | 0 | 100 | 0 | 0 | 0 | 0 | 0 | 100 | 100 | 100 | 0 | 0 |
| Rv2370c | - | 437 | 0 | 0 | 0 | 0 | 0 | 0 | 0 | 41 | 52 | 43 | 53 | 50 | 53 | 0 | 79 | 0 | 100 | 40 | 0 | 0 | 0 | 0 | 100 | 100 | 100 | 78 | 0 |

| | | | | | | | | | | | | | | | | | | | | | | | | | | | | | |
|---|---|---|---|---|---|---|---|---|---|---|---|---|---|---|---|---|---|---|---|---|---|---|---|---|---|---|---|---|---|
| Rv2371 | *PE_PGRS40* | 61 | 0 | 0 | 0 | 0 | 0 | 0 | 0 | 0 | 0 | 0 | 0 | 0 | 0 | 67 | 77 | 67 | 100 | 0 | 0 | 0 | 0 | 0 | 100 | 100 | 100 | 78 | 0 |
| Rv2372c | - | 262 | 66 | 63 | 63 | 63 | 59 | 61 | 56 | 56 | 68 | 66 | 71 | 70 | 68 | 84 | 84 | 84 | 100 | 76 | 79 | 79 | 79 | 78 | 100 | 100 | 100 | 87 | 78 |
| Rv2373c | *dnaJ2* | 382 | 72 | 71 | 71 | 71 | 68 | 69 | 72 | 66 | 81 | 82 | 84 | 83 | 87 | 94 | 93 | 94 | 100 | 88 | 87 | 87 | 87 | 87 | 100 | 100 | 100 | 93 | 87 |
| Rv2374c | *hrcA* | 343 | 75 | 75 | 73 | 74 | 71 | 73 | 70 | 72 | 86 | 87 | 87 | 87 | 90 | 97 | 96 | 97 | 100 | 93 | 94 | 94 | 94 | 95 | 100 | 100 | 100 | 96 | 94 |
| Rv2375 | - | 105 | 0 | 0 | 0 | 0 | 0 | 0 | 0 | 75 | 0 | 91 | 89 | 89 | 88 | 95 | 95 | 95 | 100 | 90 | 97 | 97 | 97 | 96 | 100 | 100 | 100 | 93 | 95 |
| Rv2376c | *cfp2* | 168 | 0 | 0 | 0 | 0 | 0 | 0 | 0 | 0 | 0 | 0 | 0 | 0 | 57 | 76 | 77 | 77 | 100 | 55 | 54 | 54 | 54 | 50 | 100 | 100 | 100 | 90 | 54 |
| Rv2377c | *mbtH* | 71 | 0 | 0 | 0 | 0 | 66 | 0 | 66 | 0 | 91 | 88 | 90 | 90 | 88 | 92 | 90 | 92 | 98 | 89 | 90 | 90 | 90 | 88 | 98 | 100 | 98 | 90 | 81 |
| Rv2378c | *mbtG* | 431 | 0 | 0 | 0 | 0 | 55 | 55 | 0 | 0 | 69 | 0 | 0 | 0 | 83 | 93 | 78 | 93 | 100 | 84 | 83 | 83 | 83 | 83 | 100 | 100 | 100 | 78 | 84 |
| Rv2379c | *mbtF* | 1461 | 42 | 44 | 46 | 46 | 45 | 38 | 45 | 40 | 57 | 47 | 48 | 55 | 57 | 70 | 55 | 70 | 99 | 71 | 75 | 75 | 75 | 73 | 100 | 100 | 100 | 55 | 67 |
| Rv2380c | *mbtE* | 1682 | 46 | 41 | 50 | 49 | 49 | 43 | 49 | 42 | 59 | 52 | 53 | 63 | 59 | 77 | 58 | 77 | 100 | 74 | 80 | 80 | 80 | 78 | 100 | 100 | 100 | 58 | 76 |
| Rv2381c | *mbtD* | 1004 | 38 | 41 | 41 | 40 | 36 | 0 | 45 | 0 | 48 | 42 | 46 | 45 | 46 | 60 | 48 | 60 | 100 | 62 | 67 | 67 | 67 | 62 | 100 | 100 | 100 | 48 | 66 |
| Rv2382c | *mbtC* | 444 | 57 | 55 | 58 | 57 | 57 | 0 | 56 | 48 | 71 | 57 | 63 | 63 | 66 | 83 | 67 | 83 | 100 | 81 | 88 | 87 | 87 | 85 | 100 | 100 | 100 | 67 | 86 |
| Rv2383c | *mbtB* | 1414 | 45 | 43 | 45 | 45 | 50 | 40 | 50 | 40 | 64 | 43 | 43 | 64 | 64 | 81 | 64 | 81 | 100 | 69 | 72 | 72 | 72 | 73 | 100 | 100 | 100 | 64 | 71 |
| Rv2384 | *mbtA* | 565 | 41 | 41 | 41 | 41 | 49 | 51 | 43 | 44 | 68 | 59 | 60 | 66 | 72 | 84 | 85 | 85 | 100 | 76 | 75 | 76 | 76 | 78 | 100 | 100 | 100 | 80 | 75 |
| Rv2385 | *mbtJ* | 306 | 42 | 0 | 0 | 44 | 41 | 0 | 0 | 40 | 46 | 43 | 45 | 48 | 61 | 79 | 79 | 79 | 100 | 55 | 56 | 56 | 56 | 47 | 100 | 100 | 100 | 83 | 47 |
| Rv2386c | *mbtI* | 450 | 49 | 45 | 48 | 47 | 45 | 47 | 48 | 44 | 65 | 43 | 45 | 65 | 76 | 83 | 86 | 83 | 100 | 46 | 46 | 46 | 46 | 77 | 100 | 100 | 100 | 86 | 45 |
| Rv2387 | - | 417 | 0 | 0 | 0 | 0 | 0 | 0 | 0 | 0 | 0 | 0 | 0 | 0 | 0 | 85 | 89 | 85 | 100 | 0 | 0 | 0 | 0 | 0 | 100 | 100 | 100 | 89 | 0 |
| Rv2388c | *hemN* | 375 | 72 | 70 | 70 | 73 | 65 | 69 | 62 | 61 | 73 | 73 | 75 | 76 | 76 | 88 | 91 | 88 | 99 | 79 | 78 | 78 | 78 | 83 | 100 | 100 | 100 | 91 | 78 |
| Rv2389c | *rpfD* | 154 | 61 | 57 | 62 | 66 | 59 | 63 | 64 | 66 | 73 | 72 | 72 | 78 | 65 | 62 | 78 | 62 | 100 | 75 | 71 | 71 | 71 | 68 | 100 | 100 | 100 | 77 | 68 |
| Rv2390c | - | 185 | 0 | 0 | 0 | 0 | 0 | 0 | 0 | 0 | 46 | 57 | 52 | 50 | 69 | 70 | 72 | 72 | 99 | 63 | 47 | 48 | 48 | 60 | 100 | 100 | 100 | 64 | 65 |
| Rv2391 | *nirA* | 563 | 70 | 0 | 70 | 69 | 70 | 70 | 70 | 72 | 89 | 89 | 89 | 88 | 90 | 94 | 94 | 94 | 100 | 90 | 89 | 89 | 89 | 89 | 100 | 100 | 100 | 94 | 88 |
| Rv2392 | *cysH* | 254 | 53 | 0 | 58 | 60 | 61 | 63 | 63 | 64 | 77 | 77 | 76 | 79 | 79 | 88 | 89 | 88 | 100 | 81 | 79 | 79 | 79 | 80 | 100 | 100 | 100 | 89 | 81 |
| Rv2393 | - | 281 | 43 | 0 | 43 | 45 | 44 | 0 | 41 | 54 | 56 | 59 | 57 | 55 | 61 | 78 | 80 | 77 | 100 | 66 | 69 | 69 | 69 | 70 | 100 | 100 | 100 | 77 | 67 |
| Rv2394 | *ggtB* | 643 | 0 | 0 | 66 | 66 | 0 | 69 | 0 | 41 | 76 | 0 | 77 | 77 | 80 | 43 | 88 | 43 | 100 | 80 | 80 | 80 | 80 | 82 | 100 | 100 | 100 | 85 | 40 |
| Rv2395 | - | 667 | 73 | 76 | 0 | 0 | 0 | 75 | 78 | 0 | 0 | 0 | 0 | 0 | 0 | 0 | 85 | 0 | 99 | 0 | 0 | 53 | 53 | 78 | 100 | 100 | 100 | 85 | 0 |
| Rv2396 | *PE_PGRS41* | 361 | 43 | 45 | 39 | 40 | 37 | 34 | 37 | 41 | 45 | 45 | 46 | 43 | 45 | 53 | 60 | 54 | 99 | 57 | 56 | 55 | 55 | 53 | 100 | 99 | 100 | 59 | 54 |
| Rv2397c | *cysA1* | 351 | 63 | 67 | 56 | 59 | 58 | 59 | 54 | 71 | 85 | 84 | 83 | 82 | 89 | 95 | 94 | 92 | 100 | 91 | 92 | 92 | 92 | 92 | 100 | 100 | 100 | 94 | 93 |
| Rv2398c | *cysW* | 272 | 51 | 47 | 48 | 45 | 53 | 41 | 51 | 68 | 82 | 83 | 82 | 82 | 88 | 93 | 93 | 93 | 99 | 85 | 89 | 89 | 89 | 87 | 99 | 100 | 99 | 92 | 88 |
| Rv2399c | *cysT* | 283 | 50 | 50 | 44 | 47 | 52 | 45 | 47 | 71 | 77 | 78 | 77 | 77 | 89 | 94 | 92 | 94 | 100 | 87 | 87 | 87 | 87 | 86 | 100 | 100 | 100 | 92 | 87 |
| Rv2400c | *subI* | 356 | 0 | 0 | 0 | 0 | 0 | 0 | 0 | 63 | 74 | 75 | 76 | 76 | 81 | 86 | 88 | 86 | 100 | 77 | 80 | 80 | 80 | 84 | 100 | 100 | 100 | 87 | 76 |
| Rv2401 | - | 109 | 0 | 0 | 0 | 0 | 0 | 0 | 0 | 0 | 0 | 0 | 0 | 0 | 0 | 0 | 51 | 0 | 100 | 0 | 0 | 0 | 0 | 0 | 100 | 100 | 100 | 0 | 0 |
| Rv2401A | - | 67 | 0 | 0 | 0 | 0 | 0 | 0 | 0 | 0 | 0 | 0 | 0 | 0 | 64 | 84 | 83 | 84 | 100 | 74 | 67 | 67 | 67 | 67 | 100 | 100 | 100 | 83 | 75 |
| Rv2402 | - | 642 | 0 | 0 | 52 | 50 | 0 | 0 | 0 | 54 | 85 | 89 | 87 | 88 | 89 | 97 | 97 | 97 | 100 | 94 | 93 | 94 | 94 | 94 | 100 | 100 | 100 | 97 | 94 |
| Rv2403c | *lppR* | 251 | 0 | 0 | 0 | 0 | 0 | 0 | 0 | 0 | 0 | 0 | 0 | 0 | 44 | 62 | 61 | 62 | 100 | 49 | 42 | 42 | 42 | 53 | 100 | 100 | 100 | 62 | 47 |
| Rv2404c | *lepA* | 653 | 86 | 87 | 87 | 87 | 88 | 86 | 85 | 83 | 94 | 91 | 92 | 92 | 93 | 91 | 92 | 92 | 100 | 94 | 94 | 94 | 94 | 94 | 100 | 100 | 100 | 95 | 95 |
| Rv2405 | - | 189 | 65 | 65 | 57 | 68 | 56 | 70 | 52 | 69 | 78 | 85 | 76 | 82 | 76 | 86 | 90 | 86 | 100 | 77 | 76 | 76 | 76 | 73 | 100 | 100 | 100 | 89 | 79 |
| Rv2406c | - | 142 | 0 | 0 | 0 | 0 | 0 | 0 | 0 | 71 | 70 | 75 | 75 | 75 | 0 | 96 | 96 | 96 | 100 | 80 | 80 | 80 | 80 | 87 | 96 | 100 | 100 | 95 | 79 |
| Rv2407 | - | 273 | 0 | 0 | 0 | 0 | 39 | 0 | 41 | 43 | 0 | 0 | 51 | 40 | 41 | 86 | 88 | 86 | 97 | 83 | 79 | 79 | 79 | 83 | 97 | 100 | 97 | 88 | 84 |

| | | | | | | | | | | | | | | | | | | | | | | | | | | | | | |
|---|---|---|---|---|---|---|---|---|---|---|---|---|---|---|---|---|---|---|---|---|---|---|---|---|---|---|---|---|---|
| Rv2408 | *PE24* | 239 | 0 | 0 | 0 | 0 | 0 | 0 | 0 | 0 | 0 | 0 | 0 | 0 | 61 | 55 | 43 | 62 | 100 | 56 | 51 | 51 | 51 | 64 | 100 | 100 | 100 | 55 | 64 |
| Rv2409c | - | 279 | 0 | 0 | 0 | 0 | 0 | 0 | 0 | 0 | 0 | 75 | 75 | 75 | 83 | 87 | 87 | 87 | 100 | 87 | 86 | 86 | 86 | 86 | 100 | 100 | 100 | 92 | 88 |
| Rv2410c | - | 325 | 0 | 0 | 0 | 0 | 0 | 0 | 0 | 0 | 0 | 86 | 87 | 87 | 91 | 95 | 94 | 94 | 100 | 92 | 91 | 91 | 91 | 92 | 100 | 100 | 100 | 94 | 92 |
| Rv2411c | - | 551 | 0 | 0 | 0 | 0 | 0 | 0 | 0 | 0 | 0 | 84 | 84 | 84 | 86 | 90 | 91 | 92 | 100 | 89 | 87 | 87 | 87 | 88 | 100 | 100 | 100 | 91 | 89 |
| Rv2412 | *rpsT* | 86 | 76 | 75 | 76 | 74 | 65 | 72 | 64 | 79 | 84 | 89 | 90 | 90 | 86 | 97 | 97 | 97 | 100 | 88 | 90 | 90 | 90 | 88 | 100 | 100 | 100 | 97 | 87 |
| Rv2413c | - | 316 | 56 | 61 | 63 | 64 | 63 | 65 | 63 | 50 | 78 | 75 | 78 | 78 | 82 | 91 | 91 | 91 | 100 | 91 | 89 | 89 | 89 | 89 | 100 | 100 | 100 | 91 | 91 |
| Rv2414c | - | 514 | 48 | 47 | 56 | 48 | 46 | 47 | 43 | 49 | 55 | 58 | 58 | 59 | 63 | 80 | 79 | 79 | 100 | 71 | 69 | 77 | 77 | 68 | 100 | 100 | 100 | 79 | 69 |
| Rv2415c | - | 297 | 54 | 52 | 54 | 55 | 53 | 58 | 54 | 53 | 62 | 55 | 58 | 63 | 65 | 77 | 76 | 77 | 99 | 68 | 69 | 69 | 69 | 68 | 100 | 100 | 99 | 75 | 69 |
| Rv2416c | *eis* | 402 | 0 | 0 | 0 | 0 | 0 | 0 | 0 | 41 | 0 | 57 | 62 | 62 | 45 | 50 | 83 | 50 | 99 | 66 | 69 | 69 | 69 | 69 | 100 | 100 | 100 | 77 | 67 |
| Rv2417c | - | 280 | 54 | 51 | 52 | 49 | 49 | 46 | 57 | 51 | 58 | 63 | 61 | 61 | 67 | 83 | 83 | 83 | 100 | 75 | 76 | 76 | 76 | 72 | 100 | 100 | 100 | 84 | 75 |
| Rv2418c | - | 247 | 0 | 0 | 0 | 0 | 0 | 0 | 0 | 0 | 73 | 75 | 74 | 73 | 81 | 94 | 92 | 93 | 100 | 85 | 94 | 94 | 94 | 87 | 100 | 100 | 100 | 92 | 88 |
| Rv2419c | - | 223 | 60 | 57 | 66 | 60 | 67 | 57 | 55 | 49 | 74 | 76 | 79 | 80 | 83 | 89 | 91 | 89 | 99 | 81 | 84 | 84 | 84 | 83 | 100 | 100 | 100 | 90 | 84 |
| Rv2420c | - | 126 | 68 | 70 | 70 | 75 | 75 | 81 | 71 | 74 | 86 | 83 | 78 | 78 | 88 | 94 | 93 | 94 | 100 | 90 | 90 | 88 | 88 | 90 | 100 | 100 | 100 | 93 | 91 |
| Rv2421c | *nadD* | 211 | 84 | 79 | 82 | 83 | 82 | 77 | 82 | 80 | 85 | 84 | 85 | 85 | 85 | 91 | 87 | 87 | 99 | 85 | 90 | 90 | 90 | 88 | 100 | 100 | 100 | 90 | 88 |
| Rv2422 | - | 90 | 0 | 0 | 0 | 0 | 0 | 0 | 0 | 0 | 0 | 0 | 0 | 0 | 0 | 0 | 0 | 0 | 100 | 0 | 0 | 0 | 0 | 0 | 100 | 100 | 100 | 0 | 0 |
| Rv2423 | - | 348 | 0 | 0 | 0 | 0 | 0 | 0 | 0 | 0 | 0 | 0 | 0 | 0 | 0 | 73 | 73 | 73 | 100 | 75 | 0 | 0 | 0 | 0 | 100 | 100 | 100 | 87 | 73 |
| Rv2424c | - | 333 | 0 | 0 | 0 | 0 | 0 | 0 | 0 | 59 | 0 | 0 | 59 | 58 | 0 | 0 | 0 | 0 | 99 | 0 | 0 | 0 | 0 | 0 | 99 | 100 | 100 | 0 | 59 |
| Rv2425c | - | 480 | 0 | 0 | 0 | 0 | 0 | 0 | 0 | 56 | 72 | 74 | 74 | 73 | 83 | 94 | 96 | 94 | 100 | 91 | 91 | 91 | 91 | 89 | 100 | 100 | 100 | 95 | 91 |
| Rv2426c | - | 291 | 0 | 0 | 0 | 0 | 0 | 0 | 0 | 76 | 86 | 85 | 89 | 88 | 92 | 97 | 98 | 97 | 99 | 93 | 93 | 94 | 94 | 93 | 100 | 100 | 100 | 98 | 93 |
| Rv2427c | *proA* | 415 | 70 | 69 | 71 | 71 | 68 | 69 | 69 | 71 | 82 | 84 | 83 | 82 | 82 | 89 | 94 | 89 | 100 | 87 | 87 | 87 | 87 | 89 | 100 | 100 | 100 | 94 | 87 |
| Rv2428 | *ahpC* | 195 | 80 | 84 | 0 | 0 | 54 | 84 | 54 | 0 | 92 | 93 | 57 | 57 | 93 | 93 | 94 | 93 | 100 | 0 | 0 | 0 | 0 | 92 | 100 | 100 | 100 | 94 | 47 |
| Rv2429 | *ahpD* | 177 | 74 | 76 | 0 | 0 | 0 | 72 | 0 | 0 | 84 | 85 | 0 | 0 | 78 | 84 | 85 | 85 | 100 | 0 | 0 | 0 | 0 | 79 | 100 | 100 | 100 | 89 | 0 |
| Rv2430c | *PPE41* | 194 | 0 | 0 | 0 | 0 | 0 | 0 | 0 | 0 | 0 | 0 | 0 | 0 | 44 | 52 | 50 | 52 | 100 | 43 | 45 | 45 | 45 | 45 | 100 | 100 | 100 | 50 | 44 |
| Rv2431c | *PE25* | 99 | 0 | 0 | 0 | 0 | 0 | 0 | 0 | 0 | 0 | 0 | 0 | 0 | 0 | 51 | 50 | 50 | 100 | 0 | 0 | 0 | 0 | 0 | 100 | 100 | 100 | 51 | 0 |
| Rv2432c | - | 136 | 0 | 0 | 0 | 0 | 0 | 0 | 0 | 0 | 0 | 0 | 0 | 0 | 0 | 0 | 0 | 0 | 100 | 0 | 0 | 0 | 0 | 0 | 100 | 100 | 100 | 0 | 0 |
| Rv2433c | - | 96 | 0 | 0 | 0 | 0 | 0 | 0 | 0 | 0 | 0 | 0 | 0 | 0 | 0 | 0 | 0 | 0 | 100 | 0 | 0 | 0 | 0 | 0 | 100 | 100 | 100 | 0 | 0 |
| Rv2434c | - | 481 | 0 | 0 | 0 | 0 | 0 | 0 | 0 | 0 | 0 | 0 | 0 | 0 | 0 | 77 | 86 | 78 | 100 | 0 | 0 | 0 | 0 | 75 | 100 | 100 | 100 | 86 | 0 |
| Rv2435c | - | 730 | 0 | 0 | 0 | 43 | 0 | 0 | 43 | 0 | 0 | 0 | 0 | 0 | 0 | 80 | 80 | 80 | 100 | 54 | 51 | 51 | 51 | 75 | 100 | 100 | 100 | 76 | 50 |
| Rv2436 | *rbsK* | 304 | 52 | 50 | 45 | 46 | 44 | 55 | 47 | 45 | 55 | 53 | 58 | 58 | 62 | 80 | 0 | 80 | 99 | 69 | 72 | 71 | 71 | 68 | 99 | 100 | 99 | 0 | 70 |
| Rv2437 | - | 139 | 0 | 0 | 0 | 0 | 0 | 0 | 0 | 0 | 0 | 0 | 0 | 0 | 68 | 0 | 0 | 0 | 100 | 68 | 0 | 0 | 0 | 0 | 100 | 100 | 100 | 0 | 74 |
| Rv2438A | - | 92 | 0 | 0 | 0 | 0 | 0 | 0 | 0 | 0 | 0 | 0 | 0 | 0 | 0 | 0 | 0 | 0 | 100 | 0 | 0 | 0 | 0 | 0 | 100 | 100 | 100 | 0 | 0 |
| Rv2438c | *nadE* | 679 | 0 | 0 | 0 | 0 | 0 | 0 | 0 | 79 | 0 | 0 | 0 | 0 | 0 | 92 | 92 | 92 | 100 | 88 | 90 | 90 | 90 | 92 | 100 | 100 | 99 | 93 | 88 |
| Rv2439c | *proB* | 376 | 70 | 73 | 70 | 70 | 71 | 69 | 72 | 66 | 83 | 83 | 83 | 84 | 85 | 90 | 93 | 91 | 100 | 89 | 89 | 89 | 89 | 88 | 100 | 100 | 100 | 92 | 90 |
| Rv2440c | *obgE* | 479 | 75 | 74 | 73 | 75 | 72 | 70 | 70 | 68 | 81 | 81 | 81 | 81 | 86 | 91 | 91 | 91 | 100 | 89 | 88 | 88 | 88 | 90 | 100 | 100 | 100 | 93 | 88 |
| Rv2441c | *rpmA* | 86 | 88 | 84 | 83 | 85 | 86 | 84 | 87 | 88 | 92 | 88 | 92 | 92 | 93 | 93 | 95 | 93 | 100 | 91 | 93 | 93 | 93 | 93 | 100 | 100 | 100 | 94 | 89 |
| Rv2442c | *rplU* | 104 | 76 | 80 | 81 | 80 | 75 | 77 | 75 | 81 | 86 | 87 | 89 | 89 | 93 | 94 | 94 | 94 | 100 | 92 | 93 | 93 | 93 | 90 | 100 | 100 | 100 | 94 | 92 |
| Rv2443 | *dctA* | 491 | 48 | 48 | 38 | 80 | 78 | 77 | 45 | 0 | 81 | 84 | 83 | 83 | 82 | 0 | 79 | 0 | 100 | 81 | 82 | 82 | 82 | 81 | 100 | 100 | 100 | 79 | 82 |
| Rv2444c | *rne* | 953 | 58 | 60 | 60 | 56 | 58 | 52 | 53 | 62 | 61 | 66 | 68 | 60 | 72 | 81 | 78 | 81 | 100 | 72 | 74 | 73 | 73 | 70 | 100 | 100 | 100 | 80 | 75 |

| | | | | | | | | | | | | | | | | | | | | | | | | | | | | | |
|---|---|---|---|---|---|---|---|---|---|---|---|---|---|---|---|---|---|---|---|---|---|---|---|---|---|---|---|---|---|
| Rv2445c | *ndk* | 136 | 79 | 81 | 78 | 78 | 78 | 80 | 78 | 73 | 89 | 84 | 84 | 85 | 86 | 90 | 91 | 91 | 100 | 86 | 88 | 88 | 88 | 90 | 100 | 100 | 100 | 91 | 87 |
| Rv2446c | - | 123 | 54 | 55 | 55 | 56 | 61 | 58 | 55 | 0 | 66 | 71 | 70 | 70 | 73 | 86 | 78 | 86 | 100 | 78 | 82 | 82 | 82 | 75 | 100 | 100 | 99 | 76 | 82 |
| Rv2447c | *folC* | 487 | 66 | 70 | 70 | 71 | 76 | 72 | 73 | 65 | 81 | 78 | 81 | 82 | 84 | 91 | 91 | 91 | 100 | 84 | 86 | 86 | 86 | 87 | 100 | 100 | 100 | 91 | 87 |
| Rv2448c | *valS* | 876 | 77 | 79 | 81 | 80 | 77 | 76 | 76 | 42 | 83 | 85 | 85 | 86 | 86 | 92 | 94 | 93 | 100 | 87 | 89 | 89 | 89 | 89 | 100 | 100 | 100 | 94 | 88 |
| Rv2449c | - | 419 | 0 | 0 | 0 | 0 | 57 | 0 | 56 | 52 | 68 | 67 | 69 | 68 | 76 | 88 | 88 | 88 | 100 | 81 | 82 | 82 | 82 | 80 | 100 | 100 | 100 | 88 | 81 |
| Rv2450c | *rpfE* | 172 | 69 | 67 | 65 | 67 | 64 | 64 | 64 | 63 | 76 | 88 | 85 | 84 | 78 | 67 | 67 | 67 | 100 | 87 | 80 | 80 | 80 | 87 | 100 | 100 | 100 | 62 | 88 |
| Rv2451 | - | 132 | 0 | 0 | 0 | 0 | 0 | 0 | 0 | 0 | 0 | 0 | 0 | 0 | 0 | 0 | 61 | 0 | 100 | 0 | 0 | 0 | 0 | 0 | 100 | 100 | 100 | 60 | 0 |
| Rv2452c | - | 48 | 0 | 0 | 0 | 0 | 0 | 0 | 0 | 0 | 0 | 0 | 0 | 0 | 0 | 0 | 0 | 0 | 100 | 0 | 0 | 0 | 0 | 0 | 100 | 100 | 100 | 0 | 0 |
| Rv2453c | *mobA* | 201 | 0 | 0 | 0 | 0 | 0 | 0 | 0 | 0 | 49 | 0 | 0 | 0 | 62 | 81 | 87 | 81 | 100 | 71 | 76 | 76 | 76 | 71 | 100 | 100 | 100 | 86 | 73 |
| Rv2454c | - | 373 | 0 | 0 | 0 | 0 | 0 | 0 | 0 | 77 | 82 | 0 | 84 | 84 | 88 | 93 | 93 | 93 | 100 | 89 | 91 | 91 | 91 | 92 | 100 | 100 | 100 | 93 | 91 |
| Rv2455c | - | 653 | 0 | 0 | 0 | 0 | 0 | 0 | 0 | 79 | 81 | 0 | 83 | 82 | 89 | 93 | 95 | 93 | 100 | 89 | 88 | 88 | 88 | 90 | 100 | 100 | 100 | 95 | 89 |
| Rv2456c | - | 418 | 0 | 0 | 0 | 39 | 0 | 0 | 38 | 0 | 0 | 39 | 41 | 40 | 0 | 54 | 90 | 54 | 100 | 42 | 0 | 0 | 0 | 0 | 100 | 100 | 100 | 90 | 39 |
| Rv2457c | *clpX* | 426 | 86 | 84 | 85 | 85 | 81 | 83 | 82 | 89 | 96 | 96 | 96 | 96 | 97 | 99 | 99 | 99 | 100 | 97 | 98 | 98 | 98 | 98 | 99 | 100 | 100 | 98 | 98 |
| Rv2458 | *mmuM* | 302 | 56 | 40 | 41 | 40 | 0 | 41 | 0 | 42 | 38 | 40 | 39 | 39 | 42 | 80 | 42 | 81 | 99 | 43 | 44 | 44 | 44 | 44 | 99 | 100 | 99 | 42 | 42 |
| Rv2459 | - | 508 | 44 | 46 | 51 | 48 | 46 | 50 | 49 | 49 | 53 | 56 | 50 | 51 | 53 | 52 | 88 | 47 | 100 | 51 | 52 | 52 | 52 | 51 | 100 | 100 | 100 | 88 | 51 |
| Rv2460c | *clpP2* | 214 | 85 | 86 | 87 | 87 | 90 | 87 | 90 | 77 | 94 | 88 | 95 | 95 | 94 | 98 | 98 | 98 | 100 | 96 | 95 | 95 | 95 | 97 | 100 | 100 | 100 | 98 | 97 |
| Rv2461c | *clpP* | 200 | 84 | 83 | 81 | 84 | 88 | 87 | 87 | 78 | 84 | 86 | 87 | 87 | 92 | 95 | 98 | 95 | 100 | 93 | 93 | 93 | 93 | 95 | 100 | 100 | 100 | 98 | 94 |
| Rv2462c | *tig* | 466 | 66 | 68 | 68 | 67 | 68 | 69 | 70 | 61 | 78 | 78 | 77 | 77 | 77 | 85 | 88 | 85 | 99 | 79 | 81 | 81 | 81 | 82 | 100 | 100 | 100 | 93 | 79 |
| Rv2463 | *lipP* | 394 | 0 | 0 | 0 | 0 | 0 | 0 | 0 | 0 | 47 | 50 | 52 | 51 | 77 | 87 | 87 | 87 | 100 | 47 | 82 | 82 | 82 | 82 | 100 | 100 | 100 | 90 | 49 |
| Rv2464c | - | 268 | 59 | 55 | 57 | 56 | 41 | 59 | 55 | 65 | 74 | 76 | 80 | 80 | 76 | 89 | 87 | 89 | 100 | 83 | 80 | 81 | 81 | 83 | 100 | 100 | 99 | 86 | 83 |
| Rv2465c | - | 162 | 84 | 84 | 82 | 84 | 86 | 82 | 86 | 66 | 87 | 85 | 82 | 83 | 87 | 96 | 96 | 96 | 100 | 89 | 94 | 94 | 94 | 90 | 100 | 100 | 100 | 95 | 92 |
| Rv2466c | - | 207 | 65 | 68 | 67 | 70 | 66 | 68 | 62 | 73 | 78 | 83 | 85 | 86 | 86 | 91 | 92 | 91 | 100 | 82 | 87 | 87 | 87 | 89 | 100 | 100 | 100 | 90 | 85 |
| Rv2467 | *pepN* | 861 | 61 | 66 | 65 | 65 | 62 | 60 | 62 | 61 | 82 | 81 | 81 | 82 | 85 | 91 | 91 | 91 | 100 | 85 | 86 | 86 | 86 | 85 | 100 | 100 | 100 | 90 | 85 |
| Rv2468c | - | 167 | 0 | 0 | 0 | 0 | 0 | 0 | 0 | 0 | 62 | 63 | 61 | 61 | 77 | 90 | 90 | 90 | 100 | 76 | 77 | 77 | 77 | 83 | 100 | 100 | 100 | 87 | 82 |
| Rv2469c | - | 222 | 0 | 0 | 0 | 0 | 0 | 0 | 0 | 0 | 81 | 80 | 74 | 74 | 81 | 86 | 90 | 86 | 99 | 81 | 78 | 78 | 78 | 79 | 100 | 100 | 100 | 90 | 80 |
| Rv2470 | *glbO* | 128 | 69 | 67 | 70 | 66 | 70 | 0 | 69 | 64 | 85 | 83 | 85 | 85 | 86 | 93 | 93 | 93 | 100 | 87 | 87 | 87 | 87 | 85 | 100 | 100 | 100 | 92 | 87 |
| Rv2471 | *aglA* | 546 | 44 | 56 | 43 | 44 | 43 | 38 | 0 | 62 | 77 | 76 | 76 | 77 | 77 | 85 | 88 | 87 | 99 | 61 | 81 | 81 | 81 | 78 | 99 | 100 | 100 | 87 | 61 |
| Rv2472 | - | 97 | 0 | 0 | 0 | 0 | 0 | 0 | 0 | 0 | 0 | 0 | 0 | 0 | 0 | 0 | 0 | 0 | 100 | 0 | 0 | 0 | 0 | 0 | 100 | 100 | 100 | 0 | 0 |
| Rv2473 | - | 238 | 0 | 0 | 0 | 0 | 0 | 0 | 0 | 0 | 0 | 0 | 0 | 0 | 0 | 0 | 0 | 0 | 100 | 53 | 45 | 45 | 45 | 0 | 100 | 100 | 100 | 0 | 52 |
| Rv2474c | - | 217 | 56 | 54 | 52 | 53 | 48 | 0 | 0 | 0 | 70 | 69 | 70 | 70 | 68 | 84 | 86 | 84 | 99 | 74 | 78 | 78 | 78 | 77 | 100 | 100 | 99 | 87 | 78 |
| Rv2475c | - | 138 | 0 | 0 | 0 | 0 | 0 | 0 | 0 | 0 | 72 | 70 | 70 | 70 | 87 | 91 | 91 | 91 | 100 | 86 | 86 | 86 | 86 | 85 | 100 | 100 | 100 | 90 | 86 |
| Rv2476c | *gdh* | 1624 | 0 | 0 | 0 | 0 | 0 | 0 | 0 | 58 | 68 | 67 | 68 | 68 | 75 | 89 | 90 | 89 | 99 | 81 | 82 | 82 | 82 | 82 | 99 | 100 | 99 | 90 | 82 |
| Rv2477c | - | 558 | 90 | 90 | 90 | 90 | 89 | 89 | 89 | 85 | 94 | 92 | 92 | 92 | 94 | 98 | 98 | 98 | 100 | 94 | 94 | 94 | 94 | 94 | 100 | 100 | 100 | 97 | 95 |
| Rv2478c | - | 161 | 47 | 47 | 47 | 50 | 55 | 47 | 51 | 50 | 45 | 69 | 59 | 61 | 69 | 79 | 79 | 79 | 100 | 74 | 83 | 83 | 83 | 67 | 100 | 100 | 100 | 77 | 73 |
| Rv2479c | - | 346 | 58 | 49 | 59 | 51 | 61 | 0 | 59 | 64 | 0 | 0 | 49 | 52 | 66 | 58 | 65 | 64 | 100 | 65 | 88 | 64 | 64 | 66 | 100 | 100 | 100 | 0 | 61 |
| Rv2480c | - | 108 | 65 | 0 | 66 | 0 | 61 | 0 | 60 | 76 | 0 | 0 | 0 | 0 | 59 | 63 | 82 | 66 | 100 | 82 | 96 | 77 | 77 | 84 | 100 | 100 | 100 | 0 | 62 |
| Rv2481c | - | 107 | 0 | 0 | 0 | 0 | 0 | 0 | 0 | 0 | 0 | 0 | 0 | 0 | 0 | 0 | 0 | 0 | 100 | 0 | 0 | 0 | 0 | 0 | 100 | 100 | 100 | 0 | 0 |
| Rv2482c | *plsB2* | 789 | 0 | 0 | 0 | 0 | 0 | 0 | 0 | 0 | 53 | 54 | 52 | 52 | 0 | 88 | 90 | 89 | 99 | 79 | 79 | 79 | 79 | 79 | 99 | 100 | 99 | 91 | 79 |

| | | | | | | | | | | | | | | | | | | | | | | | | | | | | | |
|---|---|---|---|---|---|---|---|---|---|---|---|---|---|---|---|---|---|---|---|---|---|---|---|---|---|---|---|---|---|
| Rv2483c | *plsC* | 580 | 48 | 46 | 47 | 47 | 47 | 49 | 45 | 47 | 50 | 57 | 58 | 58 | 69 | 87 | 90 | 87 | 99 | 77 | 78 | 78 | 78 | 85 | 100 | 100 | 100 | 90 | 79 |
| Rv2484c | - | 491 | 0 | 0 | 0 | 0 | 0 | 0 | 0 | 43 | 43 | 55 | 57 | 57 | 45 | 92 | 93 | 93 | 99 | 83 | 84 | 84 | 84 | 83 | 100 | 100 | 100 | 91 | 83 |
| Rv2485c | *lipQ* | 421 | 0 | 0 | 0 | 0 | 0 | 0 | 0 | 56 | 45 | 0 | 53 | 52 | 41 | 81 | 85 | 81 | 100 | 55 | 65 | 65 | 65 | 65 | 100 | 100 | 100 | 85 | 58 |
| Rv2486 | *echA14* | 256 | 53 | 49 | 50 | 0 | 48 | 45 | 49 | 54 | 50 | 50 | 51 | 51 | 51 | 87 | 50 | 87 | 100 | 84 | 86 | 86 | 86 | 85 | 100 | 100 | 100 | 49 | 85 |
| Rv2487c | *PE_PGRS42* | 694 | 40 | 36 | 35 | 34 | 34 | 34 | 37 | 33 | 38 | 39 | 42 | 41 | 38 | 48 | 63 | 35 | 65 | 53 | 48 | 47 | 47 | 43 | 100 | 100 | 99 | 55 | 52 |
| Rv2488c | - | 1137 | 0 | 0 | 71 | 56 | 0 | 0 | 0 | 36 | 47 | 46 | 50 | 52 | 39 | 42 | 66 | 50 | 100 | 53 | 38 | 38 | 38 | 55 | 100 | 100 | 99 | 55 | 80 |
| Rv2489c | - | 99 | 0 | 0 | 0 | 0 | 0 | 0 | 0 | 0 | 0 | 0 | 0 | 0 | 0 | 0 | 0 | 0 | 100 | 0 | 0 | 0 | 0 | 0 | 100 | 100 | 100 | 0 | 0 |
| Rv2490c | *PE_PGRS43* | 1660 | 45 | 30 | 32 | 32 | 33 | 37 | 41 | 29 | 36 | 39 | 37 | 37 | 40 | 32 | 54 | 32 | 99 | 48 | 52 | 52 | 52 | 44 | 100 | 100 | 86 | 55 | 51 |
| Rv2491 | - | 207 | 0 | 0 | 0 | 0 | 0 | 0 | 0 | 0 | 0 | 0 | 0 | 0 | 0 | 0 | 0 | 0 | 100 | 0 | 0 | 0 | 0 | 0 | 100 | 100 | 100 | 0 | 0 |
| Rv2492 | - | 250 | 0 | 0 | 0 | 0 | 0 | 0 | 0 | 0 | 0 | 0 | 0 | 0 | 0 | 0 | 0 | 0 | 99 | 0 | 0 | 0 | 0 | 0 | 100 | 100 | 97 | 0 | 0 |
| Rv2493 | - | 73 | 0 | 0 | 0 | 0 | 0 | 0 | 0 | 0 | 0 | 0 | 0 | 0 | 0 | 0 | 0 | 0 | 100 | 0 | 0 | 0 | 0 | 0 | 100 | 100 | 100 | 0 | 0 |
| Rv2494 | - | 141 | 0 | 0 | 0 | 0 | 0 | 0 | 0 | 0 | 0 | 0 | 0 | 0 | 0 | 0 | 0 | 0 | 100 | 0 | 0 | 0 | 0 | 0 | 99 | 100 | 100 | 0 | 0 |
| Rv2495c | *pdhC* | 393 | 46 | 49 | 46 | 47 | 47 | 44 | 45 | 46 | 51 | 57 | 56 | 55 | 57 | 81 | 83 | 82 | 99 | 44 | 73 | 73 | 73 | 71 | 99 | 99 | 99 | 83 | 71 |
| Rv2496c | *pdhB* | 348 | 0 | 0 | 0 | 0 | 0 | 54 | 0 | 71 | 71 | 71 | 72 | 72 | 72 | 90 | 91 | 90 | 99 | 0 | 85 | 85 | 85 | 83 | 100 | 100 | 100 | 90 | 82 |
| Rv2497c | *pdhA* | 367 | 0 | 0 | 0 | 0 | 0 | 47 | 0 | 56 | 59 | 59 | 59 | 60 | 58 | 88 | 89 | 88 | 100 | 0 | 86 | 86 | 86 | 83 | 100 | 100 | 100 | 88 | 83 |
| Rv2498c | *citE* | 273 | 0 | 57 | 59 | 60 | 60 | 61 | 0 | 45 | 77 | 79 | 79 | 78 | 74 | 86 | 86 | 86 | 100 | 74 | 74 | 74 | 74 | 75 | 100 | 100 | 100 | 89 | 75 |
| Rv2499c | - | 185 | 0 | 0 | 0 | 0 | 83 | 0 | 0 | 58 | 78 | 82 | 82 | 79 | 81 | 97 | 95 | 97 | 100 | 80 | 79 | 79 | 79 | 75 | 100 | 100 | 100 | 95 | 82 |
| Rv2500c | *fadE19* | 394 | 46 | 0 | 46 | 42 | 84 | 55 | 55 | 73 | 86 | 87 | 87 | 87 | 82 | 95 | 94 | 95 | 100 | 84 | 86 | 85 | 85 | 84 | 100 | 100 | 100 | 93 | 85 |
| Rv2501c | *accA1* | 654 | 64 | 63 | 60 | 54 | 66 | 56 | 62 | 59 | 75 | 77 | 75 | 75 | 67 | 89 | 90 | 89 | 100 | 73 | 76 | 76 | 76 | 74 | 100 | 100 | 100 | 89 | 73 |
| Rv2502c | *accD1* | 529 | 50 | 53 | 52 | 52 | 79 | 53 | 51 | 83 | 90 | 88 | 91 | 90 | 90 | 93 | 95 | 93 | 99 | 87 | 86 | 86 | 86 | 88 | 100 | 100 | 100 | 94 | 88 |
| Rv2503c | *scoB* | 218 | 0 | 0 | 76 | 79 | 80 | 0 | 0 | 82 | 0 | 86 | 82 | 83 | 0 | 80 | 80 | 80 | 100 | 79 | 79 | 79 | 79 | 78 | 100 | 100 | 100 | 93 | 81 |
| Rv2504c | *scoA* | 248 | 0 | 0 | 76 | 76 | 75 | 0 | 0 | 78 | 40 | 85 | 83 | 83 | 0 | 70 | 96 | 70 | 100 | 72 | 72 | 72 | 72 | 74 | 100 | 100 | 100 | 95 | 76 |
| Rv2505c | *fadD35* | 547 | 47 | 46 | 48 | 47 | 68 | 46 | 45 | 73 | 75 | 75 | 77 | 75 | 72 | 49 | 86 | 49 | 100 | 73 | 74 | 73 | 73 | 75 | 100 | 100 | 100 | 86 | 73 |
| Rv2506 | - | 215 | 50 | 0 | 0 | 0 | 55 | 0 | 0 | 56 | 75 | 77 | 73 | 72 | 73 | 87 | 88 | 87 | 100 | 81 | 84 | 83 | 83 | 79 | 100 | 100 | 100 | 88 | 80 |
| Rv2507 | - | 273 | 0 | 0 | 38 | 0 | 0 | 0 | 0 | 0 | 38 | 0 | 35 | 38 | 44 | 68 | 78 | 68 | 100 | 60 | 58 | 58 | 58 | 59 | 100 | 100 | 100 | 78 | 58 |
| Rv2508c | - | 445 | 62 | 0 | 63 | 65 | 0 | 0 | 0 | 39 | 68 | 62 | 65 | 66 | 68 | 84 | 87 | 84 | 100 | 76 | 78 | 78 | 78 | 75 | 100 | 100 | 100 | 86 | 76 |
| Rv2509 | - | 268 | 65 | 66 | 68 | 69 | 65 | 44 | 63 | 48 | 83 | 81 | 84 | 82 | 87 | 94 | 96 | 94 | 100 | 85 | 88 | 87 | 87 | 86 | 100 | 100 | 100 | 95 | 85 |
| Rv2510c | - | 533 | 0 | 0 | 0 | 0 | 0 | 0 | 0 | 72 | 79 | 79 | 81 | 81 | 83 | 81 | 91 | 81 | 100 | 84 | 84 | 84 | 84 | 84 | 100 | 100 | 100 | 94 | 85 |
| Rv2511 | *orn* | 215 | 69 | 70 | 73 | 73 | 71 | 73 | 71 | 66 | 85 | 84 | 86 | 86 | 85 | 89 | 92 | 89 | 100 | 86 | 84 | 84 | 84 | 86 | 100 | 100 | 100 | 92 | 86 |
| Rv2512c | - | 415 | 45 | 66 | 74 | 0 | 73 | 0 | 69 | 59 | 0 | 40 | 87 | 58 | 0 | 60 | 0 | 79 | 100 | 89 | 42 | 79 | 79 | 59 | 99 | 100 | 99 | 43 | 89 |
| Rv2513 | - | 140 | 0 | 0 | 0 | 0 | 0 | 0 | 0 | 0 | 0 | 0 | 0 | 0 | 0 | 0 | 0 | 0 | 99 | 0 | 0 | 0 | 0 | 0 | 99 | 100 | 99 | 0 | 0 |
| Rv2514c | - | 153 | 0 | 0 | 0 | 0 | 0 | 0 | 0 | 0 | 0 | 0 | 0 | 0 | 0 | 0 | 0 | 0 | 100 | 0 | 48 | 0 | 0 | 0 | 100 | 100 | 100 | 0 | 0 |
| Rv2515c | - | 415 | 0 | 0 | 0 | 0 | 0 | 0 | 0 | 0 | 0 | 0 | 0 | 0 | 0 | 0 | 0 | 0 | 100 | 0 | 45 | 0 | 0 | 0 | 100 | 100 | 100 | 0 | 0 |
| Rv2516c | - | 267 | 0 | 0 | 0 | 0 | 0 | 0 | 0 | 0 | 0 | 0 | 0 | 0 | 0 | 84 | 0 | 0 | 100 | 0 | 0 | 0 | 0 | 0 | 100 | 100 | 100 | 0 | 0 |
| Rv2517c | - | 83 | 0 | 0 | 0 | 0 | 0 | 0 | 0 | 0 | 0 | 0 | 0 | 0 | 0 | 75 | 0 | 0 | 100 | 0 | 0 | 0 | 0 | 0 | 100 | 100 | 100 | 0 | 0 |
| Rv2518c | *lppS* | 408 | 63 | 65 | 61 | 66 | 66 | 63 | 61 | 54 | 75 | 74 | 73 | 75 | 80 | 92 | 91 | 92 | 100 | 80 | 83 | 84 | 84 | 85 | 100 | 100 | 100 | 91 | 83 |
| Rv2519 | *PE26* | 492 | 0 | 0 | 0 | 0 | 0 | 0 | 0 | 0 | 0 | 0 | 49 | 0 | 0 | 51 | 80 | 51 | 99 | 45 | 61 | 60 | 60 | 57 | 100 | 100 | 100 | 73 | 62 |
| Rv2520c | - | 75 | 0 | 70 | 64 | 0 | 0 | 68 | 71 | 0 | 73 | 71 | 65 | 67 | 77 | 81 | 85 | 81 | 100 | 80 | 76 | 76 | 76 | 83 | 100 | 100 | 100 | 85 | 80 |

| | | | | | | | | | | | | | | | | | | | | | | | | | | | | | |
|---|---|---|---|---|---|---|---|---|---|---|---|---|---|---|---|---|---|---|---|---|---|---|---|---|---|---|---|---|---|
| Rv2521 | *bcp* | 157 | 80 | 78 | 76 | 76 | 80 | 76 | 76 | 79 | 89 | 87 | 89 | 89 | 84 | 91 | 92 | 92 | 100 | 90 | 89 | 89 | 89 | 89 | 100 | 100 | 100 | 92 | 90 |
| Rv2522c | - | 470 | 52 | 53 | 53 | 53 | 51 | 49 | 51 | 72 | 57 | 58 | 59 | 58 | 0 | 90 | 94 | 90 | 100 | 87 | 89 | 89 | 89 | 87 | 100 | 100 | 100 | 94 | 87 |
| Rv2523c | *acpS* | 130 | 59 | 57 | 62 | 64 | 0 | 0 | 0 | 51 | 75 | 78 | 79 | 79 | 87 | 96 | 97 | 96 | 100 | 97 | 94 | 94 | 94 | 93 | 97 | 100 | 100 | 97 | 96 |
| Rv2524c | *fas* | 3069 | 61 | 66 | 66 | 66 | 44 | 0 | 43 | 44 | 76 | 75 | 75 | 76 | 86 | 93 | 95 | 93 | 99 | 87 | 90 | 90 | 90 | 90 | 100 | 100 | 100 | 95 | 88 |
| Rv2525c | - | 240 | 62 | 60 | 65 | 64 | 0 | 0 | 0 | 0 | 71 | 72 | 70 | 70 | 78 | 94 | 95 | 94 | 100 | 57 | 81 | 81 | 81 | 56 | 100 | 100 | 100 | 95 | 53 |
| Rv2526 | - | 75 | 0 | 0 | 0 | 0 | 0 | 0 | 0 | 0 | 0 | 0 | 0 | 0 | 0 | 0 | 0 | 0 | 100 | 0 | 0 | 0 | 0 | 0 | 100 | 100 | 100 | 0 | 0 |
| Rv2527 | - | 133 | 0 | 0 | 0 | 0 | 0 | 0 | 0 | 0 | 0 | 0 | 0 | 0 | 0 | 0 | 0 | 0 | 100 | 0 | 0 | 0 | 0 | 0 | 100 | 100 | 100 | 0 | 0 |
| Rv2528c | *mrr* | 306 | 58 | 0 | 0 | 51 | 47 | 0 | 0 | 65 | 60 | 0 | 50 | 50 | 0 | 0 | 0 | 0 | 100 | 0 | 0 | 57 | 57 | 0 | 100 | 100 | 100 | 0 | 0 |
| Rv2529 | - | 463 | 0 | 0 | 0 | 0 | 0 | 0 | 0 | 66 | 0 | 0 | 0 | 54 | 0 | 0 | 0 | 0 | 99 | 67 | 0 | 0 | 0 | 0 | 99 | 100 | 100 | 0 | 0 |
| Rv2530A | - | 74 | 0 | 0 | 0 | 0 | 0 | 0 | 0 | 0 | 0 | 0 | 0 | 0 | 0 | 0 | 0 | 0 | 100 | 0 | 0 | 0 | 0 | 0 | 100 | 100 | 100 | 0 | 0 |
| Rv2530c | - | 139 | 0 | 0 | 0 | 0 | 0 | 0 | 0 | 0 | 0 | 0 | 0 | 0 | 0 | 0 | 0 | 0 | 100 | 0 | 0 | 0 | 0 | 0 | 100 | 100 | 100 | 0 | 0 |
| Rv2531c | - | 947 | 0 | 0 | 0 | 0 | 0 | 0 | 0 | 0 | 0 | 0 | 0 | 0 | 0 | 91 | 91 | 91 | 100 | 0 | 0 | 0 | 0 | 0 | 100 | 100 | 100 | 92 | 0 |
| Rv2532c | - | 133 | 0 | 0 | 0 | 0 | 0 | 0 | 0 | 0 | 0 | 0 | 0 | 0 | 0 | 84 | 89 | 85 | 100 | 0 | 0 | 0 | 0 | 0 | 100 | 100 | 100 | 89 | 0 |
| Rv2533c | *nusB* | 156 | 62 | 60 | 61 | 56 | 63 | 64 | 65 | 66 | 73 | 73 | 74 | 75 | 85 | 91 | 89 | 91 | 99 | 88 | 87 | 87 | 87 | 90 | 94 | 100 | 100 | 88 | 88 |
| Rv2534c | *efp* | 187 | 81 | 83 | 83 | 82 | 83 | 87 | 81 | 77 | 88 | 89 | 89 | 89 | 91 | 97 | 97 | 97 | 100 | 94 | 94 | 94 | 94 | 94 | 100 | 100 | 100 | 96 | 93 |
| Rv2535c | *pepQ* | 372 | 64 | 61 | 63 | 65 | 56 | 56 | 57 | 43 | 71 | 69 | 71 | 71 | 73 | 88 | 88 | 88 | 100 | 79 | 80 | 80 | 80 | 82 | 100 | 100 | 100 | 91 | 80 |
| Rv2536 | - | 230 | 0 | 0 | 0 | 0 | 0 | 0 | 0 | 0 | 55 | 49 | 44 | 46 | 61 | 74 | 80 | 74 | 100 | 69 | 70 | 70 | 70 | 76 | 100 | 100 | 100 | 74 | 70 |
| Rv2537c | *aroD* | 147 | 69 | 75 | 74 | 65 | 76 | 73 | 74 | 69 | 70 | 84 | 70 | 73 | 86 | 93 | 94 | 94 | 100 | 64 | 91 | 91 | 91 | 59 | 100 | 100 | 100 | 95 | 64 |
| Rv2538c | *aroB* | 362 | 66 | 66 | 66 | 65 | 70 | 69 | 67 | 62 | 84 | 83 | 84 | 84 | 87 | 94 | 95 | 94 | 100 | 90 | 90 | 90 | 90 | 90 | 100 | 100 | 100 | 93 | 90 |
| Rv2539c | *aroK* | 176 | 65 | 63 | 66 | 62 | 62 | 69 | 60 | 64 | 70 | 74 | 78 | 77 | 85 | 92 | 95 | 92 | 100 | 91 | 93 | 93 | 93 | 90 | 100 | 100 | 100 | 94 | 91 |
| Rv2540c | *aroF* | 401 | 82 | 78 | 80 | 81 | 81 | 77 | 81 | 75 | 89 | 88 | 88 | 88 | 91 | 95 | 95 | 95 | 100 | 90 | 92 | 92 | 92 | 93 | 100 | 100 | 100 | 94 | 91 |
| Rv2541 | - | 135 | 0 | 0 | 0 | 0 | 0 | 0 | 0 | 0 | 0 | 0 | 0 | 0 | 0 | 57 | 51 | 57 | 100 | 0 | 0 | 0 | 0 | 0 | 100 | 100 | 100 | 50 | 0 |
| Rv2542 | - | 403 | 41 | 50 | 0 | 0 | 40 | 0 | 0 | 0 | 0 | 47 | 47 | 47 | 0 | 59 | 60 | 60 | 99 | 40 | 45 | 44 | 44 | 45 | 99 | 100 | 99 | 56 | 35 |
| Rv2543 | *lppA* | 219 | 0 | 0 | 0 | 0 | 0 | 0 | 0 | 0 | 0 | 0 | 0 | 0 | 0 | 0 | 0 | 0 | 100 | 0 | 0 | 0 | 0 | 0 | 100 | 100 | 100 | 0 | 0 |
| Rv2544 | *lppB* | 220 | 0 | 0 | 0 | 0 | 0 | 0 | 0 | 0 | 0 | 0 | 0 | 0 | 0 | 0 | 45 | 0 | 100 | 0 | 0 | 0 | 0 | 0 | 100 | 100 | 100 | 46 | 0 |
| Rv2545 | - | 92 | 0 | 0 | 0 | 0 | 0 | 0 | 0 | 0 | 0 | 0 | 0 | 0 | 0 | 0 | 0 | 0 | 100 | 0 | 0 | 0 | 0 | 0 | 100 | 100 | 98 | 0 | 0 |
| Rv2546 | - | 137 | 0 | 0 | 0 | 0 | 0 | 0 | 0 | 0 | 0 | 0 | 0 | 0 | 0 | 0 | 0 | 0 | 100 | 0 | 0 | 0 | 0 | 0 | 100 | 100 | 100 | 0 | 0 |
| Rv2547 | - | 85 | 0 | 0 | 0 | 0 | 0 | 0 | 0 | 0 | 0 | 0 | 0 | 0 | 0 | 0 | 0 | 0 | 100 | 0 | 0 | 0 | 0 | 0 | 100 | 100 | 100 | 0 | 0 |
| Rv2548 | - | 125 | 0 | 0 | 0 | 0 | 0 | 0 | 0 | 0 | 0 | 0 | 0 | 0 | 0 | 0 | 0 | 0 | 100 | 0 | 0 | 0 | 0 | 0 | 100 | 100 | 100 | 0 | 0 |
| Rv2549c | - | 131 | 0 | 0 | 0 | 0 | 0 | 0 | 0 | 0 | 0 | 0 | 0 | 0 | 0 | 0 | 0 | 0 | 100 | 0 | 0 | 0 | 0 | 0 | 100 | 100 | 100 | 0 | 0 |
| Rv2550c | - | 81 | 0 | 0 | 0 | 0 | 0 | 0 | 0 | 0 | 0 | 0 | 0 | 0 | 0 | 0 | 0 | 0 | 100 | 0 | 0 | 0 | 0 | 0 | 100 | 100 | 100 | 0 | 0 |
| Rv2551c | - | 139 | 52 | 0 | 52 | 55 | 0 | 0 | 0 | 0 | 58 | 58 | 57 | 54 | 69 | 71 | 74 | 71 | 100 | 67 | 73 | 73 | 73 | 72 | 100 | 100 | 100 | 77 | 69 |
| Rv2552c | *aroE* | 269 | 60 | 57 | 61 | 60 | 57 | 55 | 53 | 51 | 72 | 72 | 72 | 72 | 71 | 87 | 89 | 87 | 100 | 78 | 82 | 83 | 83 | 81 | 100 | 100 | 100 | 89 | 78 |
| Rv2553c | - | 417 | 59 | 59 | 62 | 59 | 61 | 60 | 61 | 50 | 60 | 62 | 64 | 64 | 71 | 84 | 85 | 84 | 100 | 75 | 77 | 77 | 77 | 79 | 100 | 100 | 100 | 84 | 76 |
| Rv2554c | - | 170 | 62 | 62 | 63 | 62 | 63 | 63 | 62 | 63 | 67 | 69 | 71 | 70 | 74 | 87 | 81 | 88 | 100 | 80 | 84 | 84 | 84 | 83 | 100 | 100 | 100 | 81 | 81 |
| Rv2555c | *alaS* | 904 | 72 | 74 | 74 | 74 | 73 | 73 | 74 | 67 | 78 | 79 | 80 | 80 | 87 | 94 | 92 | 94 | 99 | 91 | 90 | 90 | 90 | 91 | 100 | 100 | 100 | 92 | 91 |
| Rv2556c | - | 129 | 0 | 0 | 0 | 0 | 0 | 0 | 0 | 72 | 70 | 0 | 69 | 69 | 0 | 93 | 93 | 93 | 100 | 85 | 92 | 92 | 92 | 92 | 100 | 100 | 100 | 90 | 90 |
| Rv2557 | - | 224 | 0 | 0 | 0 | 0 | 0 | 0 | 0 | 65 | 0 | 0 | 72 | 63 | 0 | 77 | 78 | 78 | 100 | 72 | 79 | 79 | 79 | 75 | 100 | 100 | 100 | 77 | 72 |

| | | | | | | | | | | | | | | | | | | | | | | | | | | | | | |
|---|---|---|---|---|---|---|---|---|---|---|---|---|---|---|---|---|---|---|---|---|---|---|---|---|---|---|---|---|---|
| Rv2558 | - | 236 | 0 | 0 | 0 | 0 | 0 | 0 | 0 | 63 | 0 | 0 | 72 | 65 | 0 | 92 | 93 | 93 | 100 | 67 | 78 | 78 | 78 | 71 | 100 | 100 | 100 | 90 | 66 |
| Rv2559c | - | 452 | 78 | 78 | 77 | 76 | 78 | 78 | 81 | 76 | 81 | 84 | 83 | 86 | 86 | 93 | 92 | 93 | 99 | 87 | 88 | 88 | 88 | 89 | 100 | 100 | 100 | 92 | 88 |
| Rv2560 | - | 325 | 0 | 0 | 0 | 43 | 0 | 0 | 0 | 50 | 45 | 51 | 50 | 49 | 52 | 59 | 69 | 59 | 100 | 54 | 54 | 55 | 55 | 49 | 99 | 100 | 100 | 71 | 56 |
| Rv2561 | - | 97 | 0 | 0 | 0 | 0 | 0 | 0 | 0 | 0 | 0 | 0 | 0 | 0 | 0 | 90 | 92 | 90 | 100 | 0 | 0 | 0 | 0 | 0 | 100 | 100 | 98 | 90 | 0 |
| Rv2562 | - | 129 | 0 | 0 | 0 | 0 | 0 | 0 | 0 | 0 | 0 | 0 | 0 | 0 | 0 | 71 | 78 | 71 | 99 | 0 | 0 | 0 | 0 | 0 | 100 | 100 | 100 | 79 | 0 |
| Rv2563 | - | 349 | 41 | 42 | 0 | 0 | 42 | 0 | 43 | 39 | 0 | 39 | 0 | 0 | 43 | 0 | 88 | 0 | 100 | 0 | 0 | 0 | 0 | 0 | 100 | 100 | 100 | 0 | 0 |
| Rv2564 | *glnQ* | 330 | 60 | 58 | 61 | 60 | 61 | 62 | 62 | 62 | 62 | 63 | 63 | 63 | 60 | 63 | 91 | 62 | 100 | 61 | 59 | 59 | 59 | 60 | 100 | 100 | 100 | 59 | 63 |
| Rv2565 | - | 583 | 0 | 0 | 0 | 0 | 0 | 0 | 0 | 45 | 0 | 0 | 47 | 47 | 43 | 48 | 48 | 48 | 100 | 48 | 47 | 48 | 48 | 53 | 99 | 100 | 100 | 49 | 52 |
| Rv2566 | - | 1140 | 0 | 0 | 0 | 0 | 0 | 0 | 0 | 0 | 0 | 0 | 0 | 0 | 83 | 38 | 91 | 38 | 98 | 84 | 86 | 87 | 87 | 86 | 99 | 100 | 99 | 92 | 86 |
| Rv2567 | - | 884 | 0 | 0 | 0 | 0 | 0 | 0 | 0 | 0 | 0 | 49 | 64 | 63 | 75 | 88 | 88 | 88 | 100 | 78 | 80 | 80 | 80 | 79 | 100 | 100 | 100 | 88 | 78 |
| Rv2568c | - | 341 | 0 | 0 | 0 | 0 | 0 | 0 | 0 | 0 | 0 | 0 | 71 | 0 | 79 | 88 | 87 | 88 | 100 | 79 | 80 | 80 | 80 | 82 | 100 | 100 | 100 | 86 | 81 |
| Rv2569c | - | 314 | 0 | 0 | 0 | 0 | 0 | 0 | 0 | 0 | 0 | 48 | 73 | 73 | 73 | 94 | 92 | 94 | 100 | 82 | 82 | 82 | 82 | 83 | 100 | 100 | 100 | 91 | 81 |
| Rv2570 | - | 129 | 0 | 0 | 0 | 0 | 0 | 0 | 0 | 65 | 0 | 0 | 0 | 0 | 0 | 85 | 82 | 85 | 100 | 0 | 0 | 0 | 0 | 77 | 100 | 100 | 100 | 80 | 52 |
| Rv2571c | - | 355 | 45 | 50 | 47 | 50 | 48 | 48 | 46 | 46 | 47 | 52 | 47 | 51 | 0 | 65 | 72 | 65 | 100 | 0 | 0 | 0 | 0 | 0 | 100 | 100 | 100 | 50 | 0 |
| Rv2572c | *aspS* | 596 | 83 | 83 | 83 | 82 | 82 | 83 | 83 | 82 | 87 | 86 | 86 | 87 | 90 | 93 | 94 | 93 | 99 | 89 | 90 | 90 | 90 | 91 | 100 | 100 | 100 | 94 | 90 |
| Rv2573 | - | 275 | 0 | 0 | 37 | 39 | 0 | 0 | 0 | 0 | 0 | 0 | 38 | 0 | 46 | 82 | 85 | 83 | 100 | 0 | 76 | 76 | 76 | 0 | 100 | 100 | 100 | 84 | 0 |
| Rv2574 | - | 167 | 0 | 0 | 0 | 0 | 0 | 0 | 0 | 0 | 0 | 56 | 44 | 0 | 0 | 89 | 89 | 89 | 100 | 75 | 77 | 77 | 77 | 74 | 100 | 100 | 100 | 83 | 77 |
| Rv2575 | - | 293 | 55 | 60 | 60 | 60 | 51 | 62 | 50 | 59 | 71 | 70 | 70 | 69 | 79 | 89 | 93 | 89 | 100 | 82 | 83 | 83 | 83 | 85 | 100 | 100 | 100 | 93 | 82 |
| Rv2576c | - | 154 | 0 | 0 | 0 | 0 | 0 | 0 | 0 | 0 | 0 | 0 | 0 | 0 | 55 | 69 | 85 | 69 | 100 | 53 | 59 | 59 | 59 | 52 | 100 | 100 | 100 | 83 | 53 |
| Rv2577 | - | 529 | 0 | 0 | 0 | 0 | 0 | 0 | 0 | 0 | 0 | 0 | 0 | 0 | 42 | 86 | 85 | 85 | 99 | 0 | 0 | 0 | 0 | 0 | 100 | 100 | 100 | 84 | 0 |
| Rv2578c | - | 340 | 0 | 0 | 0 | 0 | 0 | 0 | 0 | 0 | 73 | 74 | 80 | 79 | 81 | 89 | 89 | 89 | 100 | 82 | 84 | 84 | 84 | 83 | 100 | 100 | 100 | 88 | 82 |
| Rv2579 | *dhaA* | 300 | 0 | 0 | 0 | 0 | 0 | 0 | 0 | 0 | 43 | 59 | 55 | 0 | 0 | 46 | 75 | 46 | 99 | 43 | 45 | 45 | 45 | 81 | 100 | 100 | 100 | 68 | 45 |
| Rv2580c | *hisS* | 423 | 81 | 80 | 83 | 83 | 80 | 80 | 83 | 39 | 89 | 88 | 89 | 89 | 87 | 94 | 95 | 94 | 100 | 91 | 89 | 89 | 89 | 90 | 100 | 100 | 100 | 95 | 93 |
| Rv2581c | - | 224 | 52 | 51 | 50 | 50 | 52 | 48 | 51 | 63 | 47 | 70 | 70 | 70 | 76 | 91 | 93 | 92 | 100 | 82 | 84 | 84 | 84 | 83 | 100 | 100 | 100 | 93 | 82 |
| Rv2582 | *ppiB* | 308 | 58 | 58 | 60 | 59 | 53 | 56 | 55 | 49 | 65 | 64 | 64 | 64 | 68 | 85 | 82 | 82 | 99 | 73 | 77 | 77 | 77 | 76 | 100 | 100 | 100 | 87 | 71 |
| Rv2583c | *relA* | 790 | 77 | 79 | 79 | 79 | 80 | 80 | 78 | 76 | 88 | 88 | 93 | 90 | 92 | 95 | 95 | 95 | 100 | 93 | 91 | 91 | 91 | 93 | 100 | 100 | 100 | 95 | 93 |
| Rv2584c | *apt* | 223 | 65 | 69 | 73 | 71 | 64 | 67 | 66 | 62 | 61 | 62 | 63 | 64 | 78 | 73 | 83 | 72 | 99 | 77 | 73 | 73 | 73 | 79 | 99 | 100 | 99 | 83 | 73 |
| Rv2585c | - | 557 | 43 | 44 | 45 | 45 | 42 | 39 | 40 | 0 | 53 | 56 | 59 | 58 | 71 | 85 | 85 | 86 | 99 | 78 | 78 | 78 | 78 | 78 | 99 | 100 | 99 | 84 | 76 |
| Rv2586c | *secF* | 442 | 65 | 69 | 66 | 69 | 62 | 66 | 65 | 55 | 71 | 72 | 73 | 74 | 73 | 82 | 84 | 83 | 99 | 78 | 77 | 77 | 77 | 78 | 100 | 100 | 100 | 84 | 76 |
| Rv2587c | *secD* | 573 | 58 | 60 | 59 | 60 | 60 | 60 | 59 | 54 | 72 | 75 | 75 | 74 | 81 | 85 | 85 | 85 | 100 | 77 | 83 | 83 | 83 | 78 | 100 | 100 | 100 | 87 | 79 |
| Rv2588c | *yajC* | 115 | 0 | 0 | 67 | 62 | 54 | 0 | 52 | 0 | 55 | 66 | 60 | 60 | 80 | 84 | 84 | 84 | 100 | 87 | 78 | 78 | 78 | 82 | 100 | 100 | 100 | 94 | 85 |
| Rv2589 | *gabT* | 449 | 72 | 50 | 51 | 73 | 48 | 47 | 47 | 81 | 48 | 77 | 77 | 77 | 74 | 92 | 90 | 92 | 100 | 86 | 86 | 86 | 86 | 88 | 100 | 100 | 100 | 89 | 85 |
| Rv2590 | *fadD9* | 1168 | 40 | 40 | 41 | 40 | 42 | 39 | 41 | 42 | 69 | 41 | 41 | 42 | 72 | 82 | 85 | 82 | 99 | 43 | 79 | 78 | 78 | 82 | 99 | 100 | 100 | 84 | 79 |
| Rv2591 | *PE_PGRS44* | 543 | 35 | 34 | 36 | 37 | 39 | 38 | 34 | 36 | 39 | 38 | 43 | 42 | 45 | 47 | 71 | 37 | 99 | 47 | 50 | 45 | 45 | 50 | 100 | 100 | 100 | 63 | 44 |
| Rv2592c | *ruvB* | 344 | 84 | 86 | 85 | 85 | 88 | 85 | 87 | 81 | 88 | 91 | 93 | 93 | 94 | 96 | 98 | 96 | 100 | 95 | 94 | 94 | 94 | 95 | 100 | 100 | 100 | 98 | 95 |
| Rv2593c | *ruvA* | 196 | 68 | 64 | 65 | 62 | 58 | 56 | 62 | 60 | 78 | 81 | 84 | 84 | 82 | 88 | 91 | 88 | 100 | 84 | 88 | 88 | 88 | 89 | 100 | 100 | 100 | 91 | 84 |
| Rv2594c | *ruvC* | 188 | 76 | 80 | 72 | 73 | 79 | 77 | 76 | 72 | 81 | 80 | 81 | 81 | 87 | 90 | 91 | 90 | 100 | 86 | 86 | 86 | 86 | 86 | 100 | 100 | 100 | 91 | 84 |
| Rv2595 | - | 81 | 0 | 0 | 0 | 0 | 0 | 0 | 0 | 0 | 0 | 0 | 0 | 0 | 0 | 0 | 0 | 0 | 100 | 0 | 0 | 0 | 0 | 0 | 100 | 100 | 100 | 0 | 0 |

| | | | | | | | | | | | | | | | | | | | | | | | | | | | | |
|---|---|---|---|---|---|---|---|---|---|---|---|---|---|---|---|---|---|---|---|---|---|---|---|---|---|---|---|---|---|
| Rv2596 | - | 134 | 0 | 0 | 0 | 0 | 0 | 0 | 0 | 0 | 0 | 0 | 0 | 0 | 0 | 0 | 0 | 0 | 99 | 0 | 47 | 49 | 49 | 0 | 100 | 100 | 100 | 0 | 0 |
| Rv2597 | - | 206 | 0 | 0 | 61 | 61 | 0 | 0 | 0 | 0 | 0 | 62 | 62 | 62 | 0 | 85 | 87 | 85 | 100 | 0 | 75 | 74 | 74 | 0 | 100 | 100 | 100 | 86 | 0 |
| Rv2598 | - | 164 | 0 | 0 | 50 | 46 | 0 | 0 | 0 | 0 | 0 | 55 | 56 | 53 | 0 | 73 | 75 | 73 | 100 | 0 | 64 | 64 | 64 | 0 | 100 | 100 | 100 | 75 | 0 |
| Rv2599 | - | 143 | 0 | 0 | 56 | 47 | 0 | 0 | 0 | 0 | 0 | 50 | 53 | 52 | 0 | 83 | 93 | 82 | 99 | 0 | 68 | 68 | 68 | 0 | 100 | 100 | 100 | 91 | 0 |
| Rv2600 | - | 133 | 0 | 0 | 63 | 64 | 0 | 0 | 0 | 0 | 66 | 66 | 65 | 65 | 55 | 78 | 89 | 77 | 100 | 0 | 81 | 81 | 81 | 0 | 100 | 100 | 100 | 89 | 0 |
| Rv2601 | *speE* | 523 | 0 | 0 | 71 | 71 | 0 | 0 | 0 | 0 | 62 | 75 | 75 | 75 | 0 | 87 | 88 | 88 | 100 | 0 | 83 | 83 | 83 | 0 | 100 | 100 | 100 | 88 | 0 |
| Rv2601A | - | 95 | 0 | 0 | 0 | 0 | 0 | 0 | 0 | 0 | 0 | 0 | 0 | 0 | 0 | 0 | 0 | 0 | 100 | 0 | 0 | 0 | 0 | 0 | 100 | 100 | 100 | 0 | 0 |
| Rv2602 | - | 146 | 0 | 0 | 0 | 0 | 0 | 0 | 0 | 0 | 0 | 0 | 0 | 0 | 0 | 0 | 0 | 0 | 100 | 0 | 0 | 0 | 0 | 0 | 100 | 100 | 100 | 0 | 0 |
| Rv2603c | - | 251 | 84 | 85 | 87 | 86 | 85 | 85 | 82 | 83 | 94 | 94 | 93 | 93 | 96 | 95 | 98 | 95 | 100 | 94 | 94 | 94 | 94 | 95 | 100 | 100 | 100 | 98 | 95 |
| Rv2604c | - | 198 | 47 | 61 | 0 | 62 | 70 | 65 | 0 | 71 | 79 | 79 | 80 | 80 | 80 | 90 | 87 | 91 | 100 | 87 | 90 | 90 | 90 | 86 | 100 | 100 | 100 | 87 | 87 |
| Rv2605c | *tesB2* | 281 | 58 | 65 | 65 | 67 | 65 | 0 | 65 | 62 | 74 | 76 | 75 | 75 | 76 | 94 | 94 | 94 | 99 | 88 | 90 | 90 | 90 | 87 | 100 | 100 | 100 | 90 | 88 |
| Rv2606c | - | 299 | 87 | 89 | 87 | 87 | 91 | 90 | 89 | 91 | 90 | 93 | 95 | 95 | 96 | 95 | 96 | 95 | 100 | 95 | 96 | 96 | 96 | 96 | 100 | 100 | 100 | 96 | 95 |
| Rv2607 | *pdxH* | 224 | 0 | 0 | 0 | 0 | 0 | 0 | 0 | 57 | 68 | 62 | 62 | 64 | 77 | 82 | 81 | 82 | 100 | 81 | 83 | 83 | 83 | 78 | 100 | 100 | 100 | 82 | 78 |
| Rv2608 | *PPE42* | 580 | 0 | 0 | 0 | 0 | 0 | 0 | 0 | 0 | 0 | 48 | 0 | 0 | 47 | 69 | 74 | 74 | 99 | 45 | 47 | 50 | 50 | 46 | 99 | 100 | 100 | 59 | 47 |
| Rv2609c | - | 351 | 57 | 57 | 56 | 56 | 52 | 49 | 50 | 0 | 63 | 63 | 65 | 64 | 70 | 84 | 84 | 84 | 100 | 78 | 76 | 76 | 76 | 77 | 100 | 100 | 100 | 84 | 77 |
| Rv2610c | *pimA* | 378 | 63 | 62 | 64 | 64 | 64 | 66 | 62 | 42 | 78 | 80 | 81 | 81 | 84 | 91 | 91 | 91 | 100 | 85 | 88 | 88 | 88 | 88 | 99 | 100 | 100 | 91 | 86 |
| Rv2611c | - | 316 | 65 | 61 | 64 | 63 | 63 | 59 | 64 | 0 | 71 | 75 | 75 | 76 | 75 | 83 | 86 | 84 | 99 | 78 | 83 | 83 | 83 | 84 | 99 | 100 | 99 | 86 | 82 |
| Rv2612c | *pgsA1* | 217 | 65 | 66 | 67 | 65 | 62 | 65 | 62 | 56 | 71 | 76 | 77 | 77 | 76 | 85 | 91 | 85 | 100 | 80 | 80 | 80 | 80 | 81 | 100 | 100 | 100 | 91 | 80 |
| Rv2613c | - | 195 | 75 | 75 | 74 | 72 | 69 | 76 | 66 | 73 | 84 | 84 | 85 | 84 | 86 | 91 | 94 | 91 | 100 | 87 | 90 | 89 | 89 | 90 | 100 | 100 | 100 | 94 | 88 |
| Rv2614A | - | 75 | 0 | 0 | 0 | 0 | 0 | 0 | 0 | 0 | 0 | 0 | 0 | 0 | 0 | 0 | 0 | 0 | 98 | 0 | 0 | 0 | 0 | 0 | 100 | 100 | 100 | 0 | 0 |
| Rv2614c | *thrS* | 692 | 78 | 79 | 79 | 79 | 78 | 77 | 78 | 73 | 85 | 84 | 84 | 84 | 88 | 91 | 95 | 91 | 100 | 89 | 89 | 89 | 89 | 89 | 100 | 100 | 100 | 94 | 89 |
| Rv2615c | *PE_PGRS45* | 461 | 38 | 50 | 40 | 37 | 37 | 38 | 40 | 38 | 46 | 39 | 36 | 42 | 42 | 43 | 46 | 46 | 98 | 46 | 51 | 53 | 53 | 45 | 100 | 100 | 100 | 60 | 51 |
| Rv2616 | - | 166 | 48 | 0 | 46 | 0 | 47 | 0 | 0 | 0 | 64 | 63 | 57 | 59 | 63 | 82 | 80 | 82 | 100 | 68 | 75 | 75 | 75 | 0 | 100 | 100 | 100 | 78 | 68 |
| Rv2617c | - | 146 | 0 | 0 | 0 | 0 | 0 | 0 | 0 | 75 | 0 | 0 | 81 | 83 | 0 | 0 | 0 | 0 | 100 | 0 | 0 | 0 | 0 | 0 | 100 | 100 | 100 | 0 | 0 |
| Rv2618 | - | 225 | 0 | 0 | 0 | 0 | 0 | 0 | 0 | 61 | 51 | 0 | 60 | 59 | 0 | 44 | 0 | 44 | 100 | 0 | 49 | 49 | 49 | 0 | 99 | 100 | 100 | 0 | 43 |
| Rv2619c | - | 117 | 0 | 0 | 0 | 66 | 0 | 0 | 0 | 73 | 66 | 67 | 70 | 70 | 67 | 92 | 91 | 92 | 100 | 71 | 72 | 72 | 72 | 77 | 100 | 100 | 100 | 90 | 70 |
| Rv2620c | - | 141 | 0 | 0 | 0 | 0 | 0 | 0 | 0 | 0 | 77 | 0 | 0 | 68 | 64 | 82 | 85 | 82 | 100 | 73 | 71 | 71 | 71 | 72 | 100 | 100 | 100 | 85 | 70 |
| Rv2621c | - | 224 | 45 | 43 | 43 | 44 | 0 | 0 | 0 | 56 | 55 | 0 | 45 | 58 | 45 | 76 | 76 | 76 | 99 | 48 | 65 | 65 | 65 | 62 | 100 | 100 | 100 | 79 | 73 |
| Rv2622 | - | 273 | 0 | 66 | 0 | 65 | 0 | 67 | 0 | 0 | 0 | 0 | 0 | 0 | 0 | 84 | 90 | 84 | 100 | 72 | 69 | 0 | 0 | 74 | 100 | 100 | 100 | 91 | 73 |
| Rv2623 | *TB31.7* | 297 | 56 | 54 | 56 | 55 | 56 | 57 | 54 | 48 | 58 | 59 | 56 | 56 | 54 | 71 | 86 | 71 | 100 | 72 | 71 | 71 | 71 | 67 | 100 | 100 | 100 | 86 | 69 |
| Rv2624c | - | 272 | 44 | 43 | 43 | 43 | 43 | 45 | 44 | 43 | 44 | 44 | 45 | 45 | 54 | 55 | 84 | 55 | 100 | 62 | 68 | 68 | 68 | 59 | 100 | 100 | 100 | 75 | 60 |
| Rv2625c | - | 393 | 0 | 0 | 0 | 0 | 0 | 0 | 0 | 46 | 0 | 0 | 0 | 0 | 0 | 0 | 86 | 0 | 100 | 0 | 0 | 0 | 0 | 0 | 100 | 100 | 100 | 86 | 69 |
| Rv2626c | - | 143 | 50 | 0 | 0 | 0 | 0 | 0 | 0 | 0 | 70 | 0 | 0 | 0 | 0 | 0 | 60 | 0 | 100 | 0 | 0 | 0 | 0 | 0 | 100 | 100 | 100 | 60 | 84 |
| Rv2627c | - | 413 | 0 | 0 | 0 | 0 | 0 | 0 | 0 | 0 | 0 | 0 | 0 | 0 | 46 | 46 | 83 | 46 | 99 | 77 | 80 | 80 | 80 | 45 | 99 | 99 | 99 | 87 | 79 |
| Rv2628 | - | 120 | 0 | 0 | 0 | 0 | 0 | 0 | 0 | 0 | 0 | 0 | 0 | 0 | 0 | 71 | 0 | 71 | 98 | 0 | 0 | 0 | 0 | 0 | 100 | 100 | 100 | 0 | 69 |
| Rv2629 | - | 374 | 0 | 0 | 0 | 0 | 0 | 0 | 0 | 0 | 49 | 48 | 0 | 0 | 0 | 66 | 66 | 66 | 100 | 0 | 66 | 66 | 66 | 0 | 100 | 100 | 100 | 81 | 65 |
| Rv2630 | - | 179 | 0 | 0 | 0 | 0 | 0 | 0 | 0 | 0 | 0 | 0 | 0 | 0 | 0 | 0 | 0 | 0 | 100 | 0 | 0 | 0 | 0 | 0 | 100 | 100 | 100 | 0 | 0 |
| Rv2631 | - | 432 | 43 | 43 | 44 | 46 | 0 | 0 | 0 | 0 | 44 | 45 | 44 | 44 | 0 | 0 | 0 | 0 | 100 | 0 | 0 | 0 | 0 | 0 | 99 | 100 | 100 | 0 | 0 |

| | | | | | | | | | | | | | | | | | | | | | | | | | | | | | |
|---|---|---|---|---|---|---|---|---|---|---|---|---|---|---|---|---|---|---|---|---|---|---|---|---|---|---|---|---|---|
| Rv2632c | - | 93 | 0 | 0 | 0 | 0 | 0 | 0 | 0 | 68 | 0 | 67 | 72 | 68 | 0 | 0 | 87 | 0 | 100 | 0 | 0 | 0 | 0 | 0 | 100 | 100 | 100 | 90 | 68 |
| Rv2633c | - | 161 | 0 | 0 | 0 | 0 | 0 | 0 | 0 | 0 | 0 | 0 | 0 | 0 | 0 | 86 | 86 | 86 | 100 | 0 | 0 | 0 | 0 | 0 | 100 | 100 | 100 | 0 | 0 |
| Rv2634c | *PE_PGRS46* | 778 | 41 | 44 | 36 | 35 | 33 | 35 | 35 | 37 | 40 | 38 | 41 | 44 | 39 | 57 | 77 | 35 | 100 | 51 | 48 | 50 | 50 | 43 | 99 | 100 | 100 | 54 | 51 |
| Rv2635 | - | 80 | 0 | 0 | 0 | 0 | 0 | 0 | 0 | 0 | 0 | 0 | 0 | 0 | 0 | 0 | 0 | 0 | 100 | 0 | 0 | 0 | 0 | 0 | 100 | 100 | 100 | 0 | 0 |
| Rv2636 | - | 225 | 0 | 0 | 0 | 0 | 0 | 0 | 0 | 0 | 0 | 0 | 0 | 0 | 0 | 0 | 90 | 0 | 100 | 0 | 0 | 0 | 0 | 0 | 100 | 100 | 100 | 0 | 0 |
| Rv2637 | *dedA* | 218 | 52 | 48 | 0 | 50 | 52 | 50 | 53 | 51 | 50 | 50 | 49 | 50 | 43 | 92 | 91 | 92 | 100 | 0 | 44 | 44 | 44 | 48 | 100 | 100 | 100 | 91 | 43 |
| Rv2638 | - | 148 | 0 | 0 | 0 | 0 | 0 | 0 | 0 | 0 | 52 | 0 | 0 | 0 | 0 | 79 | 79 | 79 | 100 | 0 | 0 | 0 | 0 | 0 | 100 | 100 | 100 | 65 | 0 |
| Rv2639c | - | 110 | 0 | 0 | 0 | 0 | 0 | 0 | 0 | 90 | 82 | 87 | 85 | 85 | 87 | 0 | 90 | 0 | 100 | 83 | 86 | 86 | 86 | 85 | 100 | 100 | 100 | 0 | 83 |
| Rv2640c | - | 119 | 59 | 0 | 54 | 51 | 52 | 0 | 57 | 57 | 79 | 59 | 77 | 78 | 83 | 0 | 92 | 76 | 100 | 83 | 83 | 83 | 83 | 89 | 100 | 100 | 100 | 92 | 87 |
| Rv2641 | *cadI* | 152 | 0 | 0 | 0 | 0 | 0 | 0 | 0 | 0 | 81 | 0 | 83 | 80 | 81 | 0 | 86 | 85 | 100 | 83 | 79 | 79 | 79 | 84 | 100 | 100 | 100 | 85 | 84 |
| Rv2642 | - | 126 | 57 | 59 | 55 | 54 | 55 | 0 | 54 | 62 | 78 | 67 | 68 | 68 | 80 | 0 | 85 | 60 | 100 | 72 | 82 | 82 | 82 | 85 | 100 | 100 | 100 | 85 | 80 |
| Rv2643 | *arsC* | 498 | 77 | 0 | 76 | 77 | 69 | 66 | 76 | 82 | 87 | 90 | 91 | 89 | 89 | 0 | 90 | 90 | 99 | 89 | 91 | 90 | 90 | 91 | 100 | 100 | 100 | 92 | 92 |
| Rv2644c | - | 105 | 0 | 0 | 0 | 0 | 0 | 0 | 0 | 0 | 0 | 0 | 0 | 0 | 0 | 62 | 0 | 0 | 100 | 0 | 0 | 0 | 0 | 65 | 100 | 100 | 100 | 0 | 0 |
| Rv2645 | - | 143 | 0 | 0 | 0 | 0 | 0 | 0 | 0 | 0 | 0 | 0 | 0 | 0 | 0 | 0 | 0 | 0 | 0 | 0 | 0 | 0 | 0 | 0 | 100 | 100 | 99 | 0 | 0 |
| Rv2646 | - | 332 | 40 | 56 | 41 | 43 | 42 | 48 | 54 | 46 | 60 | 48 | 48 | 59 | 48 | 46 | 46 | 46 | 46 | 46 | 47 | 47 | 47 | 48 | 100 | 100 | 100 | 46 | 47 |
| Rv2647 | - | 122 | 0 | 0 | 0 | 0 | 0 | 0 | 0 | 0 | 0 | 0 | 0 | 0 | 0 | 0 | 0 | 0 | 84 | 0 | 0 | 0 | 0 | 0 | 100 | 100 | 100 | 0 | 0 |
| Rv2648 | - | 108 | 65 | 0 | 66 | 0 | 61 | 0 | 60 | 76 | 0 | 0 | 0 | 0 | 59 | 63 | 82 | 66 | 100 | 82 | 96 | 77 | 77 | 84 | 100 | 100 | 100 | 0 | 62 |
| Rv2649 | - | 328 | 58 | 49 | 59 | 51 | 60 | 0 | 59 | 65 | 0 | 0 | 51 | 52 | 66 | 59 | 66 | 66 | 100 | 66 | 89 | 65 | 65 | 67 | 100 | 100 | 100 | 0 | 63 |
| Rv2650c | - | 479 | 0 | 0 | 0 | 0 | 0 | 0 | 0 | 0 | 0 | 60 | 0 | 0 | 0 | 0 | 0 | 0 | 92 | 0 | 0 | 0 | 0 | 0 | 99 | 100 | 99 | 0 | 56 |
| Rv2651c | - | 177 | 0 | 48 | 0 | 0 | 0 | 0 | 0 | 0 | 0 | 43 | 0 | 52 | 0 | 0 | 0 | 0 | 94 | 0 | 0 | 0 | 0 | 0 | 100 | 100 | 100 | 0 | 0 |
| Rv2652c | - | 208 | 0 | 0 | 0 | 0 | 0 | 0 | 0 | 0 | 52 | 0 | 0 | 0 | 0 | 0 | 0 | 0 | 52 | 0 | 0 | 0 | 0 | 0 | 99 | 100 | 100 | 0 | 0 |
| Rv2653c | - | 107 | 0 | 0 | 0 | 0 | 0 | 0 | 0 | 0 | 0 | 0 | 0 | 0 | 0 | 0 | 0 | 0 | 0 | 0 | 0 | 0 | 0 | 0 | 100 | 100 | 100 | 0 | 0 |
| Rv2654c | - | 81 | 0 | 0 | 0 | 0 | 0 | 0 | 0 | 0 | 0 | 0 | 0 | 0 | 0 | 0 | 0 | 0 | 0 | 0 | 0 | 0 | 0 | 0 | 100 | 100 | 100 | 0 | 0 |
| Rv2655c | - | 475 | 0 | 0 | 0 | 0 | 0 | 0 | 0 | 0 | 0 | 45 | 0 | 0 | 0 | 0 | 0 | 0 | 0 | 0 | 0 | 0 | 55 | 0 | 100 | 100 | 100 | 0 | 75 |
| Rv2656c | - | 130 | 0 | 0 | 0 | 0 | 0 | 0 | 0 | 0 | 0 | 0 | 0 | 0 | 0 | 54 | 0 | 0 | 83 | 0 | 0 | 0 | 0 | 0 | 100 | 100 | 100 | 0 | 66 |
| Rv2657c | - | 86 | 0 | 0 | 0 | 0 | 0 | 0 | 0 | 0 | 0 | 0 | 0 | 0 | 0 | 84 | 0 | 0 | 0 | 78 | 69 | 0 | 0 | 0 | 100 | 100 | 100 | 0 | 78 |
| Rv2658c | - | 120 | 0 | 0 | 0 | 0 | 0 | 0 | 0 | 0 | 0 | 0 | 0 | 0 | 0 | 0 | 0 | 0 | 0 | 0 | 0 | 0 | 0 | 0 | 100 | 100 | 100 | 0 | 0 |
| Rv2659c | - | 375 | 39 | 40 | 43 | 46 | 0 | 44 | 42 | 44 | 41 | 54 | 0 | 44 | 0 | 70 | 43 | 55 | 0 | 71 | 67 | 47 | 43 | 0 | 100 | 100 | 100 | 44 | 69 |
| Rv2660c | - | 75 | 0 | 0 | 0 | 0 | 0 | 0 | 0 | 0 | 0 | 0 | 0 | 0 | 0 | 0 | 0 | 0 | 100 | 0 | 0 | 0 | 0 | 0 | 100 | 100 | 100 | 0 | 0 |
| Rv2661c | - | 129 | 0 | 0 | 0 | 0 | 0 | 0 | 0 | 0 | 0 | 0 | 0 | 0 | 0 | 0 | 0 | 0 | 100 | 0 | 0 | 0 | 0 | 0 | 100 | 100 | 100 | 0 | 0 |
| Rv2662 | - | 90 | 0 | 0 | 0 | 0 | 0 | 0 | 0 | 0 | 0 | 0 | 0 | 0 | 0 | 0 | 0 | 0 | 100 | 0 | 0 | 0 | 0 | 0 | 100 | 100 | 100 | 0 | 0 |
| Rv2663 | - | 77 | 0 | 0 | 0 | 0 | 0 | 0 | 0 | 0 | 0 | 0 | 0 | 64 | 0 | 0 | 0 | 0 | 100 | 0 | 0 | 0 | 0 | 0 | 100 | 100 | 100 | 0 | 0 |
| Rv2664 | - | 84 | 0 | 0 | 0 | 0 | 0 | 0 | 0 | 0 | 0 | 0 | 0 | 0 | 0 | 0 | 0 | 67 | 100 | 0 | 0 | 0 | 0 | 0 | 100 | 100 | 100 | 0 | 0 |
| Rv2665 | - | 93 | 0 | 0 | 0 | 0 | 0 | 0 | 0 | 0 | 0 | 0 | 0 | 0 | 0 | 0 | 0 | 0 | 100 | 0 | 0 | 0 | 0 | 0 | 100 | 100 | 100 | 0 | 0 |
| Rv2666 | - | 267 | 50 | 77 | 76 | 0 | 75 | 0 | 73 | 61 | 0 | 40 | 89 | 57 | 0 | 62 | 79 | 79 | 100 | 89 | 46 | 79 | 79 | 60 | 100 | 100 | 100 | 47 | 88 |
| Rv2667 | *clpC2* | 252 | 50 | 49 | 50 | 51 | 54 | 51 | 54 | 54 | 71 | 78 | 75 | 75 | 62 | 79 | 78 | 78 | 100 | 69 | 79 | 79 | 79 | 74 | 100 | 100 | 100 | 85 | 73 |
| Rv2668 | - | 173 | 0 | 0 | 0 | 0 | 0 | 0 | 0 | 0 | 0 | 0 | 0 | 0 | 54 | 79 | 91 | 79 | 98 | 63 | 65 | 64 | 64 | 61 | 100 | 100 | 99 | 90 | 63 |
| Rv2669 | - | 156 | 0 | 0 | 0 | 0 | 0 | 0 | 0 | 0 | 63 | 66 | 65 | 64 | 67 | 73 | 80 | 74 | 100 | 71 | 73 | 72 | 72 | 70 | 100 | 100 | 100 | 78 | 72 |

| | | | | | | | | | | | | | | | | | | | | | | | | | | | | | |
|---|---|---|---|---|---|---|---|---|---|---|---|---|---|---|---|---|---|---|---|---|---|---|---|---|---|---|---|---|---|
| Rv2670c | - | 369 | 0 | 0 | 0 | 0 | 0 | 0 | 0 | 0 | 75 | 73 | 72 | 72 | 82 | 90 | 93 | 90 | 100 | 87 | 88 | 88 | 88 | 85 | 100 | 100 | 100 | 92 | 86 |
| Rv2671 | *ribD* | 258 | 48 | 49 | 48 | 50 | 0 | 0 | 0 | 45 | 56 | 56 | 60 | 59 | 63 | 83 | 80 | 83 | 100 | 68 | 66 | 66 | 66 | 70 | 100 | 100 | 100 | 79 | 68 |
| Rv2672 | - | 528 | 0 | 0 | 0 | 0 | 0 | 0 | 0 | 36 | 54 | 53 | 55 | 55 | 71 | 87 | 88 | 87 | 99 | 80 | 76 | 76 | 76 | 77 | 100 | 100 | 100 | 88 | 82 |
| Rv2673 | - | 433 | 55 | 47 | 62 | 62 | 58 | 58 | 54 | 0 | 70 | 71 | 68 | 68 | 74 | 89 | 93 | 89 | 100 | 81 | 82 | 82 | 82 | 80 | 100 | 100 | 100 | 93 | 81 |
| Rv2674 | - | 136 | 82 | 79 | 80 | 80 | 82 | 76 | 76 | 71 | 84 | 84 | 84 | 85 | 88 | 92 | 91 | 92 | 100 | 89 | 90 | 90 | 90 | 90 | 100 | 100 | 100 | 90 | 90 |
| Rv2675c | - | 250 | 0 | 0 | 0 | 0 | 0 | 0 | 0 | 0 | 0 | 48 | 50 | 49 | 58 | 89 | 88 | 88 | 100 | 51 | 51 | 51 | 51 | 53 | 100 | 100 | 100 | 86 | 51 |
| Rv2676c | - | 231 | 72 | 74 | 70 | 69 | 75 | 74 | 68 | 70 | 86 | 86 | 88 | 88 | 87 | 95 | 96 | 95 | 100 | 90 | 89 | 89 | 89 | 91 | 100 | 100 | 100 | 96 | 89 |
| Rv2677c | *hemY* | 452 | 53 | 54 | 51 | 50 | 51 | 50 | 52 | 50 | 67 | 65 | 67 | 66 | 71 | 82 | 83 | 80 | 100 | 79 | 81 | 81 | 81 | 81 | 100 | 100 | 100 | 83 | 79 |
| Rv2678c | *hemE* | 357 | 68 | 67 | 68 | 68 | 65 | 63 | 66 | 65 | 76 | 75 | 73 | 73 | 78 | 86 | 90 | 86 | 99 | 82 | 83 | 83 | 83 | 81 | 100 | 100 | 100 | 89 | 84 |
| Rv2679 | *echA15* | 276 | 45 | 41 | 48 | 0 | 45 | 46 | 43 | 48 | 51 | 50 | 50 | 78 | 47 | 89 | 91 | 89 | 100 | 52 | 57 | 51 | 51 | 51 | 100 | 100 | 100 | 90 | 50 |
| Rv2680 | - | 210 | 71 | 72 | 72 | 71 | 68 | 72 | 65 | 58 | 82 | 87 | 86 | 86 | 90 | 94 | 94 | 94 | 100 | 92 | 94 | 94 | 94 | 94 | 100 | 100 | 100 | 95 | 91 |
| Rv2681 | - | 438 | 52 | 53 | 56 | 57 | 50 | 50 | 0 | 59 | 70 | 68 | 68 | 68 | 73 | 81 | 84 | 82 | 99 | 78 | 78 | 78 | 78 | 79 | 100 | 100 | 100 | 84 | 80 |
| Rv2682c | *dxs1* | 638 | 74 | 75 | 74 | 74 | 75 | 0 | 75 | 73 | 82 | 83 | 85 | 84 | 86 | 92 | 94 | 92 | 100 | 89 | 89 | 89 | 89 | 89 | 100 | 100 | 100 | 93 | 87 |
| Rv2683 | - | 165 | 0 | 0 | 0 | 0 | 0 | 0 | 0 | 0 | 0 | 0 | 0 | 0 | 0 | 92 | 85 | 92 | 99 | 0 | 53 | 53 | 53 | 55 | 100 | 100 | 100 | 85 | 0 |
| Rv2684 | *arsA* | 429 | 0 | 0 | 41 | 0 | 0 | 0 | 0 | 0 | 69 | 42 | 43 | 43 | 0 | 84 | 87 | 84 | 100 | 44 | 68 | 68 | 68 | 70 | 99 | 100 | 100 | 86 | 44 |
| Rv2685 | *arsB1* | 428 | 0 | 0 | 44 | 0 | 0 | 0 | 0 | 0 | 68 | 42 | 41 | 40 | 0 | 82 | 89 | 82 | 99 | 43 | 66 | 67 | 67 | 68 | 100 | 100 | 100 | 88 | 43 |
| Rv2686c | - | 252 | 0 | 0 | 0 | 0 | 0 | 0 | 0 | 0 | 0 | 0 | 0 | 0 | 88 | 0 | 86 | 0 | 100 | 0 | 87 | 87 | 87 | 88 | 100 | 100 | 100 | 86 | 83 |
| Rv2687c | - | 237 | 0 | 0 | 0 | 0 | 0 | 0 | 0 | 0 | 0 | 0 | 0 | 0 | 78 | 0 | 82 | 0 | 100 | 0 | 85 | 85 | 85 | 85 | 100 | 100 | 99 | 81 | 81 |
| Rv2688c | - | 301 | 46 | 54 | 49 | 47 | 52 | 46 | 50 | 52 | 50 | 50 | 48 | 50 | 86 | 48 | 48 | 48 | 99 | 47 | 85 | 88 | 88 | 89 | 99 | 100 | 99 | 87 | 86 |
| Rv2689c | - | 405 | 51 | 56 | 55 | 58 | 46 | 47 | 51 | 48 | 58 | 56 | 61 | 61 | 70 | 79 | 81 | 81 | 100 | 73 | 75 | 75 | 75 | 78 | 100 | 100 | 100 | 82 | 74 |
| Rv2690c | - | 657 | 0 | 0 | 0 | 0 | 0 | 0 | 0 | 75 | 83 | 82 | 84 | 84 | 88 | 91 | 92 | 91 | 100 | 88 | 88 | 88 | 88 | 88 | 100 | 100 | 100 | 92 | 87 |
| Rv2691 | *ceoB* | 227 | 0 | 0 | 0 | 0 | 0 | 0 | 0 | 67 | 78 | 83 | 84 | 83 | 86 | 97 | 94 | 97 | 99 | 86 | 88 | 88 | 88 | 86 | 99 | 100 | 99 | 94 | 87 |
| Rv2692 | *ceoC* | 220 | 0 | 0 | 0 | 0 | 0 | 0 | 0 | 78 | 82 | 85 | 86 | 86 | 88 | 94 | 94 | 94 | 100 | 92 | 92 | 92 | 92 | 93 | 100 | 100 | 100 | 93 | 94 |
| Rv2693c | - | 223 | 67 | 0 | 0 | 0 | 67 | 63 | 0 | 49 | 69 | 66 | 64 | 65 | 71 | 81 | 86 | 82 | 100 | 77 | 81 | 81 | 81 | 76 | 100 | 100 | 100 | 86 | 82 |
| Rv2694c | - | 122 | 0 | 0 | 0 | 0 | 0 | 0 | 0 | 55 | 73 | 78 | 76 | 76 | 86 | 95 | 93 | 93 | 99 | 92 | 90 | 90 | 90 | 88 | 100 | 100 | 100 | 98 | 91 |
| Rv2695 | - | 235 | 0 | 0 | 0 | 0 | 0 | 0 | 0 | 0 | 61 | 68 | 66 | 68 | 75 | 83 | 82 | 84 | 100 | 73 | 77 | 77 | 77 | 74 | 100 | 100 | 100 | 82 | 78 |
| Rv2696c | - | 259 | 48 | 61 | 57 | 55 | 49 | 58 | 55 | 47 | 56 | 63 | 56 | 57 | 76 | 81 | 80 | 78 | 100 | 73 | 75 | 75 | 75 | 72 | 100 | 100 | 100 | 79 | 73 |
| Rv2697c | *dut* | 154 | 74 | 75 | 80 | 77 | 79 | 72 | 79 | 73 | 81 | 77 | 78 | 78 | 86 | 96 | 96 | 96 | 100 | 91 | 92 | 92 | 92 | 93 | 100 | 100 | 100 | 96 | 94 |
| Rv2698 | - | 161 | 58 | 62 | 58 | 58 | 62 | 60 | 58 | 44 | 72 | 65 | 67 | 68 | 77 | 84 | 91 | 83 | 100 | 75 | 78 | 78 | 78 | 79 | 100 | 100 | 100 | 91 | 77 |
| Rv2699c | - | 100 | 65 | 70 | 69 | 73 | 71 | 67 | 67 | 78 | 91 | 94 | 94 | 94 | 95 | 100 | 100 | 100 | 100 | 97 | 97 | 97 | 97 | 97 | 100 | 100 | 100 | 100 | 97 |
| Rv2700 | - | 216 | 0 | 0 | 0 | 0 | 0 | 0 | 0 | 0 | 56 | 56 | 58 | 58 | 67 | 88 | 91 | 88 | 100 | 79 | 78 | 78 | 78 | 79 | 100 | 100 | 100 | 90 | 79 |
| Rv2701c | *suhB* | 290 | 47 | 60 | 61 | 62 | 58 | 60 | 58 | 54 | 65 | 69 | 73 | 73 | 66 | 83 | 83 | 83 | 100 | 70 | 74 | 74 | 74 | 72 | 100 | 100 | 100 | 83 | 72 |
| Rv2702 | *ppgK* | 265 | 67 | 72 | 71 | 71 | 66 | 70 | 49 | 69 | 80 | 79 | 83 | 84 | 81 | 87 | 88 | 87 | 99 | 84 | 84 | 84 | 84 | 86 | 99 | 100 | 99 | 87 | 84 |
| Rv2703 | *sigA* | 528 | 72 | 73 | 70 | 73 | 72 | 74 | 73 | 69 | 76 | 76 | 77 | 77 | 80 | 87 | 84 | 87 | 100 | 79 | 80 | 81 | 81 | 79 | 100 | 100 | 100 | 85 | 80 |
| Rv2704 | - | 142 | 0 | 0 | 0 | 0 | 0 | 0 | 0 | 58 | 72 | 75 | 78 | 77 | 74 | 76 | 81 | 76 | 100 | 77 | 82 | 81 | 81 | 0 | 100 | 100 | 100 | 80 | 76 |
| Rv2705c | - | 129 | 0 | 0 | 0 | 0 | 0 | 0 | 0 | 0 | 66 | 68 | 71 | 68 | 70 | 79 | 77 | 71 | 100 | 67 | 67 | 67 | 67 | 75 | 100 | 100 | 100 | 76 | 71 |
| Rv2706c | - | 85 | 0 | 0 | 0 | 0 | 0 | 0 | 0 | 0 | 0 | 0 | 0 | 0 | 0 | 76 | 65 | 78 | 100 | 60 | 55 | 55 | 55 | 0 | 100 | 100 | 100 | 65 | 0 |
| Rv2707 | - | 324 | 0 | 0 | 0 | 0 | 0 | 0 | 0 | 54 | 66 | 70 | 65 | 66 | 79 | 90 | 90 | 90 | 100 | 82 | 83 | 83 | 83 | 80 | 100 | 100 | 100 | 89 | 80 |

| | | | | | | | | | | | | | | | | | | | | | | | | | | | | | |
|---|---|---|---|---|---|---|---|---|---|---|---|---|---|---|---|---|---|---|---|---|---|---|---|---|---|---|---|---|---|
| Rv2708c | - | 82 | 78 | 76 | 77 | 77 | 71 | 80 | 75 | 67 | 84 | 82 | 83 | 83 | 84 | 96 | 93 | 96 | 100 | 93 | 94 | 94 | 94 | 97 | 100 | 100 | 100 | 93 | 91 |
| Rv2709 | - | 148 | 0 | 0 | 0 | 62 | 0 | 0 | 0 | 0 | 77 | 61 | 64 | 57 | 75 | 80 | 75 | 80 | 100 | 84 | 77 | 77 | 77 | 73 | 100 | 100 | 100 | 75 | 86 |
| Rv2710 | *sigB* | 323 | 86 | 88 | 87 | 86 | 88 | 89 | 89 | 72 | 93 | 91 | 92 | 92 | 93 | 98 | 95 | 98 | 100 | 96 | 97 | 97 | 97 | 98 | 100 | 100 | 100 | 95 | 97 |
| Rv2711 | *ideR* | 230 | 69 | 71 | 72 | 72 | 68 | 71 | 70 | 73 | 86 | 86 | 86 | 85 | 91 | 97 | 97 | 97 | 100 | 91 | 91 | 91 | 91 | 90 | 100 | 100 | 100 | 97 | 91 |
| Rv2712c | - | 352 | 0 | 0 | 0 | 0 | 0 | 0 | 0 | 41 | 48 | 47 | 52 | 51 | 59 | 82 | 86 | 82 | 100 | 73 | 75 | 74 | 74 | 75 | 100 | 100 | 100 | 78 | 72 |
| Rv2713 | *sthA* | 468 | 51 | 48 | 50 | 51 | 51 | 50 | 49 | 49 | 49 | 91 | 48 | 48 | 91 | 97 | 98 | 97 | 100 | 48 | 47 | 48 | 48 | 95 | 100 | 100 | 100 | 98 | 96 |
| Rv2714 | - | 324 | 63 | 63 | 62 | 65 | 60 | 61 | 62 | 67 | 80 | 82 | 81 | 81 | 85 | 94 | 94 | 94 | 99 | 87 | 89 | 89 | 89 | 90 | 100 | 100 | 100 | 94 | 87 |
| Rv2715 | - | 341 | 0 | 0 | 0 | 0 | 0 | 0 | 37 | 69 | 48 | 80 | 81 | 81 | 84 | 91 | 95 | 92 | 100 | 88 | 89 | 89 | 89 | 89 | 100 | 100 | 100 | 95 | 89 |
| Rv2716 | - | 228 | 0 | 0 | 0 | 0 | 0 | 0 | 0 | 0 | 63 | 74 | 70 | 69 | 77 | 86 | 89 | 81 | 100 | 84 | 83 | 83 | 83 | 85 | 100 | 100 | 99 | 89 | 84 |
| Rv2717c | - | 164 | 0 | 0 | 53 | 0 | 55 | 0 | 0 | 56 | 70 | 56 | 53 | 55 | 0 | 86 | 86 | 86 | 100 | 70 | 75 | 75 | 75 | 76 | 100 | 100 | 100 | 82 | 72 |
| Rv2718c | *nrdR* | 154 | 77 | 79 | 84 | 83 | 76 | 76 | 74 | 80 | 93 | 90 | 96 | 96 | 94 | 97 | 96 | 97 | 100 | 96 | 96 | 96 | 96 | 96 | 100 | 100 | 100 | 96 | 95 |
| Rv2719c | - | 165 | 0 | 0 | 0 | 0 | 0 | 0 | 0 | 0 | 0 | 46 | 48 | 48 | 54 | 67 | 69 | 66 | 100 | 67 | 64 | 64 | 64 | 64 | 100 | 100 | 100 | 68 | 70 |
| Rv2720 | *lexA* | 217 | 72 | 71 | 75 | 72 | 71 | 72 | 69 | 77 | 85 | 87 | 85 | 85 | 92 | 95 | 96 | 96 | 99 | 94 | 95 | 95 | 95 | 94 | 100 | 100 | 100 | 96 | 96 |
| Rv2721c | - | 699 | 46 | 57 | 42 | 44 | 46 | 44 | 40 | 0 | 44 | 46 | 46 | 47 | 54 | 68 | 72 | 68 | 100 | 59 | 61 | 61 | 61 | 70 | 100 | 100 | 100 | 72 | 61 |
| Rv2722 | - | 82 | 0 | 0 | 0 | 0 | 0 | 0 | 0 | 0 | 0 | 0 | 0 | 0 | 0 | 0 | 0 | 65 | 100 | 0 | 0 | 0 | 0 | 0 | 100 | 100 | 100 | 0 | 0 |
| Rv2723 | - | 397 | 63 | 66 | 65 | 64 | 67 | 67 | 64 | 67 | 75 | 70 | 74 | 75 | 68 | 0 | 0 | 0 | 100 | 67 | 76 | 76 | 76 | 67 | 99 | 100 | 100 | 85 | 68 |
| Rv2724c | *fadE20* | 386 | 42 | 0 | 48 | 40 | 67 | 66 | 64 | 74 | 88 | 88 | 87 | 87 | 88 | 70 | 95 | 94 | 99 | 74 | 74 | 74 | 74 | 74 | 99 | 100 | 100 | 94 | 92 |
| Rv2725c | *hflX* | 495 | 76 | 80 | 79 | 76 | 78 | 76 | 78 | 73 | 85 | 86 | 84 | 84 | 87 | 90 | 90 | 90 | 100 | 88 | 89 | 89 | 89 | 89 | 100 | 100 | 100 | 87 | 89 |
| Rv2726c | *dapF* | 289 | 65 | 68 | 63 | 64 | 65 | 60 | 0 | 53 | 71 | 72 | 73 | 74 | 77 | 85 | 87 | 86 | 100 | 82 | 82 | 82 | 82 | 83 | 100 | 100 | 100 | 86 | 86 |
| Rv2727c | *miaA* | 314 | 71 | 71 | 74 | 72 | 69 | 70 | 66 | 62 | 79 | 75 | 79 | 80 | 79 | 87 | 87 | 88 | 100 | 83 | 83 | 84 | 84 | 81 | 100 | 100 | 100 | 87 | 83 |
| Rv2728c | - | 231 | 0 | 0 | 0 | 0 | 0 | 0 | 0 | 0 | 53 | 53 | 58 | 58 | 62 | 76 | 74 | 75 | 100 | 70 | 73 | 73 | 73 | 67 | 100 | 100 | 100 | 73 | 74 |
| Rv2729c | - | 301 | 48 | 48 | 50 | 50 | 50 | 49 | 46 | 0 | 42 | 53 | 59 | 52 | 0 | 69 | 83 | 70 | 99 | 64 | 65 | 65 | 65 | 66 | 99 | 100 | 99 | 68 | 63 |
| Rv2730 | - | 158 | 0 | 0 | 0 | 0 | 0 | 0 | 0 | 0 | 0 | 0 | 0 | 0 | 0 | 0 | 0 | 0 | 100 | 0 | 0 | 0 | 0 | 0 | 100 | 100 | 100 | 0 | 0 |
| Rv2731 | - | 450 | 68 | 63 | 66 | 66 | 65 | 61 | 61 | 60 | 72 | 74 | 72 | 72 | 81 | 89 | 90 | 89 | 100 | 83 | 83 | 83 | 83 | 84 | 100 | 100 | 100 | 90 | 85 |
| Rv2732c | - | 204 | 46 | 42 | 45 | 49 | 49 | 50 | 52 | 0 | 70 | 74 | 74 | 74 | 79 | 82 | 87 | 82 | 100 | 75 | 80 | 80 | 80 | 80 | 94 | 100 | 100 | 86 | 72 |
| Rv2733c | - | 512 | 78 | 80 | 82 | 79 | 80 | 75 | 83 | 79 | 83 | 83 | 84 | 85 | 88 | 94 | 90 | 92 | 100 | 89 | 85 | 86 | 86 | 88 | 99 | 100 | 100 | 90 | 89 |
| Rv2734 | - | 284 | 0 | 0 | 0 | 0 | 0 | 0 | 0 | 72 | 48 | 0 | 0 | 0 | 83 | 0 | 0 | 0 | 100 | 0 | 81 | 0 | 0 | 0 | 100 | 100 | 100 | 0 | 0 |
| Rv2735c | - | 330 | 0 | 0 | 0 | 0 | 0 | 0 | 0 | 0 | 0 | 0 | 0 | 0 | 0 | 0 | 0 | 0 | 100 | 0 | 0 | 0 | 0 | 0 | 100 | 100 | 100 | 0 | 0 |
| Rv2736c | *recX* | 174 | 50 | 54 | 52 | 48 | 50 | 50 | 48 | 61 | 60 | 67 | 64 | 64 | 68 | 88 | 88 | 88 | 100 | 75 | 72 | 72 | 72 | 77 | 100 | 100 | 100 | 88 | 75 |
| Rv2737A | - | 57 | 0 | 0 | 0 | 0 | 0 | 0 | 0 | 0 | 0 | 0 | 0 | 0 | 0 | 73 | 73 | 73 | 100 | 0 | 0 | 0 | 0 | 0 | 100 | 100 | 100 | 71 | 0 |
| Rv2737c | *recA* | 790 | 93 | 92 | 93 | 93 | 92 | 92 | 92 | 94 | 98 | 97 | 98 | 98 | 98 | 99 | 98 | 99 | 100 | 98 | 98 | 98 | 98 | 99 | 100 | 100 | 100 | 98 | 99 |
| Rv2738c | - | 68 | 0 | 0 | 61 | 0 | 0 | 0 | 67 | 0 | 73 | 72 | 73 | 74 | 87 | 88 | 92 | 88 | 100 | 81 | 82 | 82 | 82 | 78 | 100 | 100 | 100 | 92 | 82 |
| Rv2739c | - | 388 | 0 | 0 | 0 | 0 | 0 | 0 | 0 | 43 | 73 | 72 | 75 | 73 | 79 | 89 | 89 | 90 | 100 | 83 | 84 | 84 | 84 | 84 | 100 | 100 | 100 | 89 | 82 |
| Rv2740 | - | 149 | 0 | 0 | 0 | 0 | 0 | 0 | 0 | 46 | 52 | 61 | 61 | 61 | 65 | 72 | 77 | 72 | 100 | 68 | 71 | 71 | 71 | 75 | 100 | 100 | 100 | 76 | 55 |
| Rv2741 | *PE_PGRS47* | 525 | 45 | 48 | 38 | 35 | 36 | 36 | 37 | 40 | 39 | 42 | 39 | 45 | 45 | 56 | 67 | 53 | 84 | 51 | 53 | 53 | 53 | 49 | 77 | 99 | 99 | 60 | 49 |
| Rv2742c | - | 277 | 0 | 0 | 0 | 0 | 0 | 0 | 0 | 0 | 45 | 0 | 0 | 0 | 58 | 85 | 61 | 84 | 100 | 42 | 49 | 58 | 58 | 68 | 100 | 100 | 100 | 61 | 57 |
| Rv2743c | - | 270 | 0 | 0 | 0 | 0 | 0 | 0 | 0 | 0 | 45 | 50 | 51 | 52 | 64 | 81 | 82 | 82 | 100 | 70 | 72 | 72 | 72 | 72 | 100 | 100 | 100 | 81 | 71 |
| Rv2744c | *35kd_ag* | 270 | 84 | 87 | 87 | 82 | 89 | 85 | 83 | 0 | 86 | 89 | 85 | 86 | 92 | 92 | 95 | 93 | 100 | 90 | 90 | 90 | 90 | 95 | 100 | 100 | 100 | 94 | 91 |

| | | | | | | | | | | | | | | | | | | | | | | | | | | | | | |
|---|---|---|---|---|---|---|---|---|---|---|---|---|---|---|---|---|---|---|---|---|---|---|---|---|---|---|---|---|---|
| Rv2745c | - | 112 | 67 | 75 | 72 | 69 | 72 | 72 | 69 | 74 | 67 | 68 | 63 | 62 | 73 | 83 | 83 | 83 | 100 | 77 | 75 | 75 | 75 | 78 | 100 | 100 | 100 | 83 | 78 |
| Rv2746c | *pgsA3* | 209 | 61 | 65 | 64 | 64 | 66 | 68 | 64 | 60 | 72 | 75 | 76 | 75 | 77 | 83 | 89 | 83 | 100 | 78 | 78 | 78 | 78 | 76 | 100 | 100 | 100 | 89 | 77 |
| Rv2747 | - | 174 | 0 | 0 | 0 | 0 | 0 | 0 | 0 | 0 | 88 | 87 | 86 | 86 | 90 | 95 | 95 | 95 | 100 | 90 | 88 | 88 | 88 | 89 | 100 | 100 | 100 | 95 | 88 |
| Rv2748c | *ftsK* | 883 | 85 | 68 | 85 | 85 | 83 | 83 | 87 | 64 | 75 | 70 | 75 | 75 | 80 | 85 | 83 | 83 | 100 | 79 | 82 | 82 | 82 | 79 | 100 | 100 | 99 | 87 | 81 |
| Rv2749 | - | 104 | 0 | 0 | 0 | 0 | 0 | 0 | 0 | 0 | 0 | 0 | 53 | 51 | 59 | 92 | 91 | 91 | 100 | 85 | 83 | 83 | 83 | 79 | 100 | 100 | 100 | 90 | 80 |
| Rv2750 | - | 272 | 46 | 42 | 51 | 50 | 46 | 49 | 43 | 51 | 63 | 70 | 75 | 76 | 76 | 87 | 84 | 87 | 100 | 79 | 81 | 81 | 81 | 83 | 100 | 100 | 100 | 83 | 78 |
| Rv2751 | - | 296 | 0 | 0 | 0 | 0 | 0 | 0 | 0 | 0 | 46 | 0 | 0 | 0 | 65 | 89 | 90 | 90 | 100 | 69 | 44 | 45 | 45 | 64 | 100 | 100 | 100 | 90 | 71 |
| Rv2752c | - | 558 | 72 | 73 | 74 | 73 | 72 | 73 | 72 | 71 | 77 | 77 | 76 | 77 | 83 | 95 | 95 | 95 | 100 | 91 | 91 | 91 | 91 | 92 | 100 | 100 | 99 | 96 | 92 |
| Rv2753c | *dapA* | 300 | 71 | 73 | 74 | 74 | 73 | 73 | 71 | 63 | 77 | 82 | 82 | 82 | 81 | 93 | 93 | 93 | 100 | 86 | 86 | 86 | 86 | 85 | 100 | 100 | 100 | 93 | 87 |
| Rv2754c | *thyX* | 250 | 75 | 74 | 76 | 79 | 77 | 71 | 75 | 0 | 0 | 81 | 81 | 81 | 88 | 96 | 96 | 96 | 100 | 90 | 89 | 89 | 89 | 91 | 100 | 100 | 100 | 96 | 91 |
| Rv2755c | *hsdS.1* | 91 | 0 | 0 | 0 | 0 | 0 | 0 | 0 | 0 | 0 | 0 | 0 | 0 | 0 | 0 | 0 | 0 | 100 | 0 | 0 | 0 | 0 | 0 | 100 | 100 | 100 | 0 | 0 |
| Rv2756c | *hsdM* | 540 | 45 | 76 | 82 | 0 | 45 | 0 | 76 | 42 | 41 | 0 | 0 | 0 | 0 | 0 | 39 | 39 | 99 | 46 | 43 | 55 | 55 | 0 | 99 | 100 | 100 | 81 | 93 |
| Rv2757c | - | 138 | 0 | 0 | 0 | 0 | 0 | 0 | 0 | 0 | 0 | 0 | 0 | 0 | 0 | 0 | 0 | 0 | 100 | 58 | 0 | 0 | 0 | 0 | 100 | 100 | 100 | 0 | 0 |
| Rv2758c | - | 88 | 0 | 0 | 0 | 0 | 0 | 0 | 0 | 0 | 0 | 0 | 0 | 0 | 0 | 0 | 0 | 0 | 100 | 0 | 0 | 0 | 0 | 0 | 100 | 100 | 100 | 0 | 0 |
| Rv2759c | - | 131 | 0 | 0 | 0 | 0 | 0 | 0 | 0 | 0 | 0 | 0 | 0 | 0 | 0 | 0 | 0 | 0 | 100 | 0 | 0 | 0 | 0 | 64 | 100 | 100 | 100 | 0 | 63 |
| Rv2760c | - | 89 | 0 | 0 | 0 | 0 | 0 | 0 | 0 | 0 | 0 | 0 | 0 | 0 | 0 | 0 | 0 | 0 | 100 | 0 | 0 | 0 | 0 | 0 | 100 | 100 | 100 | 0 | 0 |
| Rv2761c | *hsdS* | 364 | 0 | 0 | 0 | 0 | 0 | 0 | 0 | 0 | 0 | 0 | 0 | 0 | 0 | 0 | 73 | 0 | 100 | 0 | 0 | 0 | 0 | 0 | 100 | 100 | 100 | 72 | 0 |
| Rv2762c | - | 139 | 0 | 0 | 0 | 0 | 0 | 0 | 0 | 0 | 53 | 0 | 54 | 53 | 60 | 63 | 67 | 0 | 99 | 55 | 57 | 57 | 57 | 0 | 100 | 100 | 100 | 67 | 57 |
| Rv2763c | *dfrA* | 159 | 58 | 62 | 57 | 56 | 51 | 52 | 56 | 58 | 68 | 68 | 68 | 70 | 70 | 82 | 82 | 82 | 100 | 80 | 80 | 80 | 80 | 76 | 100 | 100 | 100 | 81 | 78 |
| Rv2764c | *thyA* | 263 | 81 | 82 | 81 | 82 | 84 | 81 | 80 | 75 | 85 | 87 | 88 | 88 | 90 | 95 | 95 | 95 | 100 | 93 | 93 | 93 | 93 | 93 | 100 | 100 | 99 | 95 | 92 |
| Rv2765 | - | 245 | 0 | 0 | 0 | 0 | 0 | 0 | 0 | 0 | 43 | 44 | 44 | 45 | 67 | 83 | 85 | 83 | 100 | 70 | 75 | 75 | 75 | 73 | 100 | 100 | 100 | 84 | 70 |
| Rv2766c | *fabG* | 260 | 50 | 48 | 55 | 53 | 67 | 54 | 70 | 65 | 68 | 85 | 90 | 88 | 55 | 95 | 94 | 96 | 100 | 53 | 58 | 58 | 58 | 58 | 100 | 100 | 100 | 94 | 54 |
| Rv2767c | - | 117 | 0 | 0 | 0 | 0 | 0 | 0 | 0 | 0 | 0 | 0 | 0 | 0 | 0 | 0 | 0 | 0 | 100 | 0 | 0 | 0 | 0 | 0 | 100 | 100 | 100 | 0 | 0 |
| Rv2768c | *PPE43* | 394 | 0 | 0 | 0 | 0 | 0 | 0 | 0 | 0 | 40 | 0 | 35 | 36 | 41 | 63 | 82 | 63 | 99 | 41 | 41 | 41 | 41 | 43 | 99 | 100 | 99 | 77 | 43 |
| Rv2769c | *PE27* | 275 | 0 | 0 | 0 | 0 | 0 | 0 | 0 | 0 | 0 | 0 | 0 | 0 | 0 | 60 | 60 | 60 | 99 | 0 | 0 | 0 | 0 | 0 | 99 | 100 | 100 | 72 | 0 |
| Rv2770c | *PPE44* | 382 | 0 | 0 | 0 | 0 | 0 | 0 | 0 | 0 | 39 | 40 | 37 | 39 | 39 | 62 | 62 | 62 | 99 | 42 | 42 | 42 | 42 | 40 | 100 | 100 | 100 | 71 | 43 |
| Rv2771c | - | 150 | 0 | 0 | 0 | 0 | 50 | 0 | 53 | 0 | 89 | 65 | 64 | 66 | 67 | 88 | 90 | 88 | 99 | 87 | 91 | 68 | 68 | 68 | 99 | 100 | 99 | 93 | 87 |
| Rv2772c | - | 157 | 0 | 0 | 0 | 0 | 0 | 0 | 0 | 0 | 70 | 75 | 72 | 73 | 81 | 87 | 90 | 87 | 100 | 80 | 77 | 77 | 77 | 79 | 100 | 100 | 100 | 90 | 79 |
| Rv2773c | *dapB* | 245 | 74 | 75 | 72 | 73 | 69 | 76 | 66 | 63 | 84 | 83 | 86 | 86 | 89 | 95 | 91 | 95 | 99 | 82 | 91 | 90 | 90 | 91 | 99 | 99 | 99 | 93 | 83 |
| Rv2774c | - | 134 | 0 | 0 | 0 | 0 | 0 | 0 | 0 | 0 | 0 | 0 | 0 | 0 | 0 | 0 | 60 | 0 | 100 | 0 | 0 | 0 | 0 | 0 | 100 | 100 | 100 | 60 | 0 |
| Rv2775 | - | 153 | 0 | 0 | 0 | 0 | 0 | 0 | 0 | 0 | 0 | 0 | 0 | 0 | 0 | 0 | 72 | 0 | 100 | 0 | 0 | 0 | 0 | 0 | 100 | 100 | 100 | 72 | 0 |
| Rv2776c | - | 309 | 0 | 0 | 43 | 45 | 0 | 0 | 0 | 41 | 62 | 64 | 64 | 64 | 61 | 86 | 88 | 87 | 100 | 58 | 59 | 59 | 59 | 57 | 100 | 100 | 100 | 87 | 60 |
| Rv2777c | - | 356 | 0 | 0 | 0 | 0 | 0 | 0 | 0 | 0 | 61 | 68 | 68 | 68 | 61 | 84 | 90 | 85 | 100 | 58 | 61 | 61 | 61 | 56 | 100 | 100 | 100 | 83 | 58 |
| Rv2778c | - | 156 | 0 | 0 | 0 | 0 | 0 | 42 | 0 | 0 | 51 | 47 | 49 | 49 | 0 | 77 | 82 | 76 | 100 | 0 | 73 | 74 | 74 | 0 | 100 | 100 | 100 | 82 | 46 |
| Rv2779c | - | 179 | 0 | 0 | 0 | 58 | 43 | 0 | 0 | 59 | 49 | 68 | 72 | 72 | 82 | 49 | 88 | 50 | 95 | 85 | 90 | 89 | 89 | 89 | 100 | 100 | 100 | 88 | 87 |
| Rv2780 | *ald* | 371 | 70 | 0 | 0 | 0 | 0 | 0 | 49 | 74 | 79 | 80 | 81 | 81 | 87 | 91 | 91 | 91 | 100 | 88 | 88 | 88 | 88 | 90 | 99 | 100 | 100 | 90 | 88 |
| Rv2781c | - | 344 | 50 | 0 | 51 | 53 | 47 | 50 | 56 | 44 | 58 | 59 | 63 | 63 | 57 | 86 | 93 | 83 | 100 | 66 | 56 | 56 | 56 | 69 | 100 | 100 | 100 | 93 | 69 |
| Rv2782c | *pepR* | 438 | 0 | 0 | 0 | 0 | 0 | 0 | 0 | 67 | 76 | 77 | 78 | 78 | 83 | 92 | 93 | 92 | 100 | 87 | 86 | 86 | 86 | 87 | 99 | 100 | 100 | 92 | 87 |

| | | | | | | | | | | | | | | | | | | | | | | | | | | | | | |
|---|---|---|---|---|---|---|---|---|---|---|---|---|---|---|---|---|---|---|---|---|---|---|---|---|---|---|---|---|---|
| Rv2783c | *gpsI* | 752 | 80 | 81 | 80 | 81 | 77 | 78 | 79 | 82 | 88 | 88 | 90 | 90 | 91 | 94 | 93 | 94 | 100 | 92 | 92 | 92 | 92 | 92 | 100 | 100 | 100 | 93 | 91 |
| Rv2784c | *lppU* | 171 | 0 | 0 | 0 | 0 | 0 | 0 | 0 | 0 | 54 | 54 | 57 | 60 | 59 | 0 | 77 | 0 | 100 | 0 | 0 | 0 | 0 | 75 | 100 | 100 | 100 | 77 | 0 |
| Rv2785c | *rpsO* | 89 | 87 | 85 | 86 | 87 | 88 | 88 | 86 | 83 | 86 | 91 | 94 | 93 | 93 | 98 | 98 | 98 | 100 | 94 | 96 | 96 | 96 | 95 | 100 | 100 | 100 | 98 | 95 |
| Rv2786c | *ribF* | 331 | 60 | 62 | 58 | 60 | 61 | 61 | 60 | 61 | 84 | 85 | 86 | 86 | 89 | 95 | 95 | 95 | 100 | 93 | 94 | 94 | 94 | 93 | 100 | 100 | 100 | 96 | 94 |
| Rv2787 | - | 587 | 0 | 0 | 0 | 0 | 0 | 0 | 0 | 39 | 55 | 51 | 55 | 56 | 53 | 52 | 52 | 52 | 100 | 54 | 50 | 50 | 50 | 53 | 100 | 100 | 100 | 53 | 52 |
| Rv2788 | *sirR* | 228 | 65 | 60 | 65 | 67 | 61 | 62 | 65 | 52 | 87 | 85 | 86 | 85 | 86 | 91 | 96 | 90 | 99 | 90 | 87 | 87 | 87 | 89 | 100 | 100 | 100 | 95 | 87 |
| Rv2789c | *fadE21* | 410 | 45 | 0 | 44 | 47 | 48 | 48 | 48 | 50 | 83 | 81 | 82 | 81 | 85 | 86 | 91 | 87 | 100 | 77 | 77 | 78 | 78 | 79 | 100 | 100 | 100 | 91 | 86 |
| Rv2790c | *ltp1* | 401 | 0 | 0 | 43 | 0 | 0 | 0 | 0 | 47 | 87 | 83 | 84 | 84 | 85 | 94 | 92 | 94 | 100 | 95 | 91 | 91 | 91 | 49 | 99 | 100 | 100 | 92 | 90 |
| Rv2791c | - | 459 | 0 | 0 | 0 | 0 | 0 | 0 | 0 | 49 | 42 | 0 | 52 | 41 | 0 | 0 | 0 | 0 | 100 | 0 | 0 | 0 | 0 | 0 | 99 | 100 | 99 | 0 | 0 |
| Rv2792c | - | 193 | 0 | 0 | 0 | 0 | 0 | 0 | 0 | 0 | 0 | 0 | 0 | 0 | 0 | 0 | 0 | 0 | 98 | 0 | 0 | 0 | 0 | 0 | 100 | 100 | 100 | 0 | 0 |
| Rv2793c | *truB* | 298 | 70 | 71 | 68 | 66 | 66 | 60 | 67 | 65 | 74 | 74 | 73 | 73 | 82 | 87 | 86 | 84 | 100 | 81 | 81 | 81 | 81 | 82 | 100 | 100 | 100 | 85 | 85 |
| Rv2794c | - | 227 | 56 | 58 | 58 | 58 | 53 | 46 | 55 | 0 | 69 | 71 | 71 | 70 | 77 | 88 | 88 | 88 | 100 | 80 | 81 | 81 | 81 | 82 | 100 | 100 | 100 | 88 | 80 |
| Rv2795c | - | 324 | 66 | 62 | 62 | 64 | 58 | 0 | 58 | 0 | 81 | 81 | 80 | 80 | 88 | 94 | 96 | 94 | 100 | 91 | 88 | 88 | 88 | 93 | 100 | 100 | 100 | 96 | 92 |
| Rv2796c | *lppV* | 187 | 0 | 0 | 0 | 0 | 0 | 0 | 0 | 0 | 0 | 0 | 40 | 39 | 0 | 0 | 81 | 45 | 99 | 0 | 0 | 0 | 0 | 0 | 100 | 100 | 100 | 82 | 0 |
| Rv2797c | - | 562 | 42 | 42 | 0 | 0 | 42 | 43 | 43 | 0 | 0 | 47 | 44 | 45 | 0 | 41 | 90 | 41 | 100 | 47 | 40 | 40 | 40 | 44 | 100 | 100 | 100 | 90 | 41 |
| Rv2798c | - | 108 | 0 | 0 | 0 | 0 | 0 | 0 | 0 | 0 | 0 | 0 | 0 | 0 | 0 | 0 | 86 | 0 | 100 | 0 | 0 | 0 | 0 | 0 | 99 | 100 | 100 | 86 | 0 |
| Rv2799 | - | 209 | 0 | 0 | 0 | 0 | 0 | 0 | 0 | 0 | 0 | 0 | 0 | 0 | 64 | 84 | 83 | 83 | 99 | 71 | 73 | 73 | 73 | 74 | 100 | 100 | 100 | 86 | 70 |
| Rv2800 | - | 549 | 0 | 0 | 0 | 0 | 0 | 0 | 0 | 37 | 38 | 40 | 41 | 41 | 50 | 80 | 85 | 80 | 100 | 67 | 71 | 71 | 71 | 75 | 100 | 100 | 100 | 85 | 70 |
| Rv2801c | - | 118 | 0 | 0 | 0 | 0 | 0 | 0 | 0 | 0 | 0 | 0 | 0 | 0 | 0 | 0 | 0 | 0 | 100 | 0 | 0 | 0 | 0 | 0 | 100 | 100 | 100 | 0 | 87 |
| Rv2802c | - | 347 | 0 | 0 | 0 | 0 | 0 | 0 | 0 | 0 | 0 | 0 | 0 | 0 | 0 | 0 | 0 | 0 | 100 | 0 | 64 | 64 | 64 | 0 | 100 | 100 | 100 | 0 | 0 |
| Rv2803 | - | 155 | 0 | 0 | 0 | 0 | 0 | 0 | 0 | 0 | 0 | 0 | 0 | 0 | 0 | 0 | 0 | 0 | 100 | 0 | 0 | 0 | 0 | 0 | 100 | 100 | 100 | 0 | 0 |
| Rv2804c | - | 209 | 0 | 0 | 0 | 0 | 0 | 0 | 0 | 0 | 0 | 0 | 0 | 0 | 0 | 0 | 0 | 0 | 99 | 0 | 0 | 0 | 0 | 0 | 100 | 100 | 100 | 0 | 0 |
| Rv2805 | - | 134 | 0 | 0 | 0 | 0 | 0 | 0 | 0 | 0 | 0 | 0 | 0 | 0 | 0 | 0 | 0 | 0 | 99 | 0 | 0 | 0 | 0 | 0 | 100 | 100 | 100 | 0 | 0 |
| Rv2806 | - | 63 | 0 | 0 | 0 | 0 | 0 | 0 | 0 | 0 | 0 | 0 | 0 | 0 | 0 | 0 | 69 | 82 | 100 | 0 | 0 | 0 | 0 | 0 | 100 | 100 | 100 | 71 | 0 |
| Rv2807 | - | 384 | 0 | 0 | 0 | 0 | 0 | 0 | 0 | 0 | 0 | 0 | 0 | 42 | 0 | 0 | 0 | 0 | 99 | 0 | 0 | 0 | 0 | 0 | 99 | 100 | 99 | 0 | 51 |
| Rv2808 | - | 85 | 0 | 0 | 0 | 0 | 0 | 0 | 0 | 0 | 0 | 0 | 0 | 0 | 0 | 0 | 0 | 0 | 100 | 0 | 0 | 0 | 0 | 0 | 100 | 100 | 100 | 75 | 0 |
| Rv2809 | - | 103 | 0 | 0 | 0 | 0 | 0 | 0 | 0 | 0 | 0 | 0 | 0 | 0 | 0 | 0 | 0 | 0 | 99 | 0 | 0 | 0 | 0 | 0 | 100 | 100 | 100 | 0 | 0 |
| Rv2810c | - | 133 | 0 | 0 | 55 | 70 | 0 | 0 | 0 | 0 | 0 | 0 | 0 | 0 | 0 | 0 | 0 | 0 | 100 | 0 | 0 | 0 | 0 | 66 | 100 | 100 | 100 | 0 | 0 |
| Rv2811 | - | 202 | 0 | 0 | 0 | 0 | 0 | 0 | 0 | 0 | 0 | 0 | 0 | 0 | 0 | 0 | 0 | 0 | 100 | 0 | 0 | 0 | 0 | 0 | 100 | 100 | 100 | 0 | 61 |
| Rv2812 | - | 469 | 0 | 0 | 0 | 0 | 42 | 0 | 41 | 0 | 0 | 0 | 0 | 0 | 0 | 40 | 0 | 0 | 97 | 0 | 0 | 0 | 0 | 0 | 99 | 100 | 99 | 0 | 70 |
| Rv2813 | - | 270 | 0 | 0 | 0 | 0 | 0 | 0 | 0 | 0 | 0 | 0 | 0 | 0 | 0 | 0 | 0 | 0 | 100 | 0 | 0 | 0 | 0 | 0 | 100 | 100 | 100 | 0 | 78 |
| Rv2814c | - | 312 | 58 | 49 | 61 | 51 | 63 | 0 | 61 | 56 | 0 | 0 | 51 | 52 | 66 | 59 | 66 | 66 | 100 | 66 | 90 | 67 | 67 | 66 | 100 | 100 | 100 | 0 | 65 |
| Rv2815c | - | 108 | 65 | 0 | 66 | 0 | 61 | 0 | 60 | 76 | 0 | 0 | 0 | 0 | 59 | 63 | 82 | 66 | 100 | 82 | 96 | 77 | 77 | 84 | 100 | 100 | 100 | 0 | 62 |
| Rv2816c | - | 113 | 0 | 0 | 0 | 0 | 0 | 0 | 0 | 0 | 0 | 0 | 0 | 0 | 0 | 0 | 0 | 0 | 100 | 0 | 0 | 0 | 0 | 0 | 100 | 100 | 100 | 0 | 0 |
| Rv2817c | - | 338 | 0 | 0 | 0 | 0 | 0 | 0 | 0 | 0 | 0 | 0 | 0 | 0 | 0 | 0 | 0 | 0 | 100 | 0 | 0 | 0 | 0 | 0 | 100 | 100 | 100 | 0 | 0 |
| Rv2818c | - | 382 | 0 | 0 | 0 | 0 | 0 | 0 | 0 | 0 | 0 | 0 | 0 | 0 | 0 | 0 | 0 | 0 | 99 | 0 | 0 | 0 | 0 | 0 | 100 | 100 | 100 | 0 | 0 |
| Rv2819c | - | 375 | 0 | 0 | 0 | 0 | 0 | 0 | 0 | 0 | 0 | 0 | 0 | 0 | 0 | 0 | 0 | 0 | 100 | 0 | 0 | 0 | 0 | 0 | 100 | 100 | 100 | 0 | 0 |
| Rv2820c | - | 302 | 0 | 0 | 0 | 0 | 0 | 0 | 0 | 0 | 0 | 0 | 0 | 0 | 0 | 0 | 0 | 0 | 100 | 0 | 0 | 0 | 0 | 0 | 100 | 100 | 100 | 0 | 0 |

| | | | | | | | | | | | | | | | | | | | | | | | | | | | | | |
|---|---|---|---|---|---|---|---|---|---|---|---|---|---|---|---|---|---|---|---|---|---|---|---|---|---|---|---|---|---|
| Rv2821c | - | 236 | 0 | 0 | 0 | 0 | 0 | 0 | 0 | 0 | 0 | 0 | 0 | 0 | 0 | 0 | 0 | 0 | 100 | 0 | 0 | 0 | 0 | 0 | 100 | 100 | 100 | 0 | 0 |
| Rv2822c | - | 124 | 0 | 0 | 0 | 0 | 0 | 0 | 0 | 0 | 0 | 0 | 0 | 0 | 0 | 0 | 0 | 0 | 100 | 0 | 0 | 0 | 0 | 0 | 100 | 100 | 100 | 0 | 0 |
| Rv2823c | - | 809 | 0 | 0 | 0 | 0 | 0 | 0 | 0 | 0 | 0 | 0 | 0 | 0 | 0 | 0 | 0 | 0 | 99 | 0 | 0 | 0 | 0 | 0 | 99 | 100 | 99 | 0 | 0 |
| Rv2824c | - | 314 | 0 | 0 | 0 | 0 | 0 | 0 | 0 | 0 | 0 | 0 | 0 | 0 | 0 | 0 | 0 | 0 | 99 | 0 | 0 | 0 | 0 | 0 | 100 | 100 | 100 | 0 | 0 |
| Rv2825c | - | 215 | 0 | 0 | 0 | 0 | 0 | 0 | 0 | 0 | 0 | 0 | 0 | 0 | 77 | 88 | 87 | 88 | 98 | 81 | 81 | 81 | 81 | 83 | 100 | 100 | 100 | 88 | 81 |
| Rv2826c | - | 294 | 0 | 0 | 0 | 0 | 0 | 0 | 0 | 0 | 0 | 0 | 0 | 0 | 0 | 0 | 0 | 0 | 100 | 0 | 0 | 0 | 0 | 0 | 100 | 100 | 100 | 0 | 0 |
| Rv2827c | - | 295 | 0 | 0 | 0 | 0 | 0 | 0 | 0 | 0 | 0 | 0 | 0 | 0 | 0 | 0 | 0 | 0 | 100 | 0 | 0 | 0 | 0 | 0 | 99 | 100 | 100 | 0 | 0 |
| Rv2828c | - | 181 | 0 | 0 | 0 | 0 | 0 | 0 | 0 | 0 | 0 | 0 | 0 | 0 | 76 | 89 | 92 | 89 | 99 | 81 | 82 | 82 | 82 | 83 | 100 | 100 | 99 | 88 | 84 |
| Rv2829c | - | 130 | 0 | 0 | 0 | 0 | 0 | 0 | 0 | 0 | 0 | 0 | 0 | 0 | 0 | 0 | 0 | 0 | 100 | 0 | 0 | 0 | 0 | 0 | 100 | 100 | 100 | 0 | 0 |
| Rv2830c | - | 71 | 0 | 0 | 0 | 0 | 0 | 0 | 0 | 0 | 0 | 0 | 0 | 0 | 0 | 0 | 0 | 0 | 98 | 0 | 0 | 0 | 0 | 0 | 98 | 100 | 98 | 0 | 0 |
| Rv2831 | *echA16* | 249 | 54 | 41 | 57 | 37 | 57 | 54 | 57 | 60 | 57 | 56 | 53 | 55 | 76 | 94 | 94 | 93 | 99 | 85 | 89 | 89 | 89 | 88 | 100 | 100 | 100 | 57 | 86 |
| Rv2832c | *ugpC* | 360 | 71 | 65 | 73 | 65 | 52 | 69 | 69 | 69 | 68 | 78 | 75 | 74 | 68 | 72 | 87 | 72 | 100 | 63 | 68 | 68 | 68 | 67 | 100 | 100 | 100 | 71 | 60 |
| Rv2833c | *ugpB* | 436 | 0 | 0 | 67 | 65 | 0 | 66 | 0 | 0 | 0 | 73 | 75 | 38 | 0 | 41 | 86 | 42 | 99 | 0 | 42 | 42 | 42 | 39 | 100 | 100 | 100 | 0 | 0 |
| Rv2834c | *ugpE* | 275 | 49 | 48 | 80 | 79 | 0 | 79 | 48 | 55 | 50 | 84 | 84 | 56 | 56 | 53 | 93 | 53 | 100 | 50 | 49 | 49 | 49 | 53 | 100 | 100 | 100 | 90 | 50 |
| Rv2835c | *ugpA* | 303 | 44 | 43 | 81 | 80 | 0 | 80 | 44 | 55 | 45 | 83 | 84 | 57 | 50 | 56 | 93 | 56 | 100 | 52 | 51 | 51 | 51 | 55 | 100 | 100 | 100 | 93 | 52 |
| Rv2836c | *dinF* | 439 | 66 | 65 | 66 | 65 | 68 | 65 | 67 | 53 | 78 | 78 | 78 | 77 | 82 | 0 | 0 | 0 | 99 | 83 | 85 | 85 | 85 | 84 | 100 | 100 | 100 | 89 | 82 |
| Rv2837c | - | 336 | 54 | 52 | 53 | 57 | 0 | 55 | 0 | 0 | 63 | 69 | 69 | 70 | 78 | 85 | 85 | 85 | 100 | 83 | 86 | 86 | 86 | 83 | 100 | 100 | 100 | 85 | 82 |
| Rv2838c | *rbfA* | 183 | 75 | 74 | 70 | 72 | 72 | 75 | 62 | 63 | 78 | 80 | 82 | 81 | 84 | 82 | 83 | 82 | 100 | 79 | 86 | 86 | 86 | 86 | 100 | 100 | 100 | 83 | 85 |
| Rv2839c | *infB* | 900 | 73 | 74 | 74 | 71 | 78 | 68 | 76 | 73 | 80 | 79 | 79 | 80 | 85 | 89 | 89 | 88 | 100 | 85 | 85 | 85 | 85 | 84 | 100 | 100 | 100 | 89 | 85 |
| Rv2840c | - | 99 | 57 | 56 | 0 | 55 | 57 | 56 | 59 | 61 | 71 | 70 | 66 | 68 | 72 | 85 | 83 | 85 | 100 | 71 | 75 | 75 | 75 | 76 | 100 | 100 | 100 | 83 | 73 |
| Rv2841c | *nusA* | 347 | 74 | 76 | 73 | 74 | 68 | 75 | 70 | 78 | 89 | 87 | 87 | 87 | 91 | 95 | 95 | 95 | 100 | 94 | 91 | 91 | 91 | 91 | 100 | 100 | 100 | 96 | 94 |
| Rv2842c | - | 183 | 51 | 52 | 53 | 51 | 46 | 46 | 0 | 55 | 57 | 63 | 58 | 59 | 64 | 80 | 80 | 80 | 100 | 76 | 76 | 76 | 76 | 74 | 100 | 100 | 100 | 80 | 77 |
| Rv2843 | - | 181 | 0 | 0 | 0 | 0 | 0 | 0 | 0 | 0 | 47 | 54 | 52 | 54 | 55 | 83 | 87 | 83 | 100 | 63 | 62 | 62 | 62 | 58 | 100 | 100 | 100 | 83 | 63 |
| Rv2844 | - | 162 | 0 | 0 | 0 | 0 | 0 | 0 | 0 | 0 | 51 | 54 | 59 | 61 | 66 | 80 | 87 | 80 | 100 | 63 | 70 | 70 | 70 | 69 | 100 | 100 | 100 | 86 | 69 |
| Rv2845c | *proS* | 582 | 77 | 76 | 78 | 78 | 78 | 76 | 76 | 74 | 85 | 87 | 87 | 87 | 89 | 94 | 95 | 95 | 99 | 89 | 90 | 90 | 90 | 89 | 100 | 100 | 100 | 95 | 90 |
| Rv2846c | *efpA* | 530 | 44 | 47 | 49 | 53 | 47 | 46 | 48 | 57 | 63 | 68 | 70 | 69 | 78 | 92 | 92 | 92 | 99 | 84 | 83 | 83 | 83 | 84 | 100 | 100 | 100 | 93 | 83 |
| Rv2847c | *cysG* | 405 | 65 | 68 | 67 | 65 | 64 | 68 | 67 | 71 | 78 | 81 | 81 | 81 | 86 | 92 | 92 | 92 | 100 | 91 | 88 | 88 | 88 | 88 | 100 | 100 | 100 | 92 | 88 |
| Rv2848c | *cobB* | 457 | 0 | 66 | 0 | 0 | 0 | 61 | 0 | 61 | 71 | 74 | 74 | 75 | 73 | 86 | 87 | 87 | 100 | 81 | 81 | 81 | 81 | 82 | 100 | 100 | 100 | 87 | 81 |
| Rv2849c | *cobO* | 207 | 0 | 80 | 0 | 0 | 0 | 80 | 0 | 77 | 84 | 85 | 86 | 86 | 87 | 88 | 87 | 87 | 99 | 89 | 90 | 90 | 90 | 86 | 100 | 100 | 100 | 89 | 88 |
| Rv2850c | - | 629 | 0 | 76 | 74 | 75 | 0 | 68 | 0 | 63 | 73 | 72 | 71 | 71 | 77 | 87 | 86 | 87 | 99 | 81 | 78 | 78 | 78 | 80 | 100 | 100 | 100 | 86 | 80 |
| Rv2851c | - | 156 | 0 | 0 | 46 | 49 | 46 | 52 | 45 | 60 | 74 | 81 | 83 | 82 | 67 | 91 | 90 | 91 | 100 | 82 | 88 | 88 | 88 | 86 | 100 | 100 | 100 | 90 | 85 |
| Rv2852c | *mqo* | 493 | 68 | 68 | 70 | 69 | 69 | 68 | 68 | 0 | 77 | 77 | 78 | 78 | 80 | 86 | 87 | 87 | 100 | 80 | 82 | 82 | 82 | 82 | 100 | 100 | 100 | 89 | 81 |
| Rv2853 | *PE_PGRS48* | 615 | 40 | 45 | 36 | 31 | 33 | 41 | 36 | 35 | 38 | 39 | 40 | 41 | 41 | 44 | 67 | 53 | 99 | 51 | 50 | 50 | 50 | 43 | 99 | 100 | 99 | 63 | 46 |
| Rv2854 | - | 346 | 56 | 53 | 54 | 54 | 50 | 0 | 47 | 58 | 62 | 65 | 66 | 66 | 68 | 79 | 76 | 78 | 100 | 76 | 75 | 75 | 75 | 73 | 99 | 100 | 99 | 75 | 75 |
| Rv2855 | *mtr* | 459 | 69 | 69 | 68 | 68 | 63 | 65 | 63 | 44 | 45 | 77 | 77 | 77 | 72 | 91 | 93 | 91 | 100 | 83 | 81 | 81 | 81 | 85 | 100 | 100 | 100 | 46 | 82 |
| Rv2856 | *nicT* | 372 | 0 | 0 | 0 | 0 | 0 | 0 | 0 | 66 | 72 | 75 | 0 | 0 | 70 | 85 | 86 | 86 | 100 | 0 | 0 | 0 | 0 | 0 | 100 | 100 | 100 | 0 | 0 |
| Rv2857c | - | 258 | 50 | 46 | 57 | 51 | 49 | 52 | 47 | 83 | 55 | 57 | 52 | 53 | 52 | 93 | 57 | 92 | 100 | 87 | 87 | 87 | 87 | 93 | 100 | 100 | 100 | 56 | 87 |
| Rv2858c | *aldC* | 455 | 53 | 51 | 52 | 52 | 52 | 53 | 51 | 76 | 58 | 56 | 58 | 58 | 57 | 91 | 58 | 91 | 100 | 89 | 90 | 90 | 90 | 89 | 99 | 100 | 100 | 57 | 90 |

| | | | | | | | | | | | | | | | | | | | | | | | | | | | | | |
|---|---|---|---|---|---|---|---|---|---|---|---|---|---|---|---|---|---|---|---|---|---|---|---|---|---|---|---|---|---|
| Rv2859c | - | 308 | 0 | 0 | 0 | 0 | 0 | 0 | 0 | 60 | 0 | 0 | 50 | 49 | 0 | 84 | 84 | 83 | 100 | 73 | 78 | 78 | 78 | 80 | 95 | 100 | 100 | 83 | 75 |
| Rv2860c | *glnA4* | 457 | 44 | 43 | 43 | 43 | 43 | 43 | 44 | 72 | 44 | 43 | 50 | 49 | 45 | 85 | 44 | 86 | 100 | 83 | 84 | 84 | 84 | 83 | 100 | 100 | 100 | 44 | 82 |
| Rv2861c | *mapB* | 285 | 80 | 80 | 80 | 79 | 82 | 76 | 81 | 79 | 88 | 89 | 90 | 89 | 87 | 95 | 95 | 95 | 100 | 92 | 92 | 92 | 92 | 91 | 100 | 100 | 100 | 94 | 92 |
| Rv2862c | - | 194 | 0 | 0 | 0 | 0 | 0 | 0 | 0 | 0 | 46 | 43 | 46 | 45 | 41 | 93 | 95 | 94 | 98 | 88 | 84 | 84 | 84 | 86 | 100 | 100 | 100 | 95 | 88 |
| Rv2863 | - | 126 | 0 | 0 | 0 | 0 | 0 | 0 | 0 | 0 | 0 | 0 | 0 | 0 | 0 | 0 | 0 | 0 | 100 | 0 | 0 | 0 | 0 | 0 | 100 | 100 | 100 | 0 | 0 |
| Rv2864c | - | 603 | 58 | 58 | 55 | 58 | 59 | 59 | 57 | 41 | 66 | 70 | 73 | 73 | 78 | 92 | 92 | 92 | 100 | 82 | 83 | 83 | 83 | 85 | 100 | 100 | 100 | 92 | 83 |
| Rv2865 | - | 93 | 0 | 0 | 0 | 0 | 0 | 0 | 0 | 0 | 0 | 0 | 0 | 60 | 0 | 0 | 0 | 0 | 100 | 83 | 0 | 0 | 0 | 0 | 100 | 100 | 100 | 0 | 0 |
| Rv2866 | - | 87 | 0 | 0 | 0 | 0 | 0 | 0 | 0 | 54 | 0 | 0 | 0 | 75 | 0 | 0 | 0 | 0 | 100 | 0 | 0 | 0 | 0 | 0 | 100 | 100 | 100 | 0 | 0 |
| Rv2867c | - | 284 | 0 | 0 | 0 | 0 | 0 | 0 | 0 | 54 | 73 | 75 | 79 | 79 | 75 | 92 | 92 | 92 | 100 | 89 | 88 | 88 | 88 | 85 | 100 | 100 | 100 | 93 | 88 |
| Rv2868c | *ispG* | 387 | 92 | 90 | 91 | 90 | 91 | 0 | 91 | 88 | 93 | 91 | 92 | 92 | 92 | 95 | 98 | 95 | 99 | 93 | 94 | 94 | 94 | 95 | 100 | 100 | 100 | 97 | 94 |
| Rv2869c | - | 404 | 60 | 59 | 61 | 60 | 60 | 59 | 56 | 48 | 66 | 65 | 69 | 68 | 70 | 89 | 93 | 89 | 99 | 78 | 85 | 85 | 85 | 81 | 100 | 100 | 100 | 93 | 79 |
| Rv2870c | *dxr* | 413 | 72 | 72 | 72 | 73 | 74 | 0 | 73 | 67 | 78 | 82 | 84 | 84 | 86 | 89 | 92 | 89 | 100 | 88 | 86 | 86 | 86 | 85 | 100 | 100 | 100 | 91 | 86 |
| Rv2871 | - | 85 | 0 | 0 | 0 | 0 | 0 | 0 | 0 | 0 | 0 | 0 | 0 | 0 | 0 | 0 | 0 | 0 | 100 | 0 | 0 | 0 | 0 | 0 | 100 | 100 | 100 | 0 | 0 |
| Rv2872 | - | 147 | 0 | 0 | 0 | 0 | 0 | 0 | 0 | 0 | 0 | 0 | 0 | 0 | 0 | 0 | 0 | 0 | 100 | 50 | 0 | 0 | 0 | 0 | 100 | 100 | 100 | 0 | 0 |
| Rv2873 | *mpt83* | 220 | 0 | 0 | 50 | 0 | 0 | 0 | 0 | 61 | 0 | 75 | 74 | 75 | 0 | 0 | 76 | 0 | 100 | 75 | 76 | 76 | 76 | 78 | 100 | 100 | 100 | 74 | 72 |
| Rv2874 | *dipZ* | 695 | 55 | 57 | 45 | 44 | 57 | 43 | 48 | 43 | 41 | 45 | 45 | 45 | 42 | 72 | 78 | 71 | 99 | 46 | 45 | 45 | 45 | 47 | 99 | 100 | 99 | 78 | 47 |
| Rv2875 | *mpt70* | 193 | 0 | 0 | 48 | 0 | 0 | 0 | 0 | 61 | 0 | 78 | 79 | 79 | 0 | 0 | 77 | 0 | 100 | 72 | 73 | 73 | 73 | 80 | 100 | 100 | 100 | 74 | 73 |
| Rv2876 | - | 104 | 0 | 0 | 0 | 0 | 55 | 0 | 59 | 0 | 58 | 60 | 60 | 60 | 64 | 85 | 82 | 85 | 99 | 58 | 67 | 67 | 67 | 69 | 100 | 100 | 100 | 81 | 65 |
| Rv2877c | - | 287 | 0 | 0 | 0 | 0 | 0 | 0 | 0 | 58 | 44 | 0 | 0 | 0 | 70 | 82 | 84 | 82 | 100 | 45 | 0 | 0 | 0 | 78 | 100 | 100 | 100 | 83 | 45 |
| Rv2878c | *mpt53* | 173 | 0 | 0 | 0 | 0 | 0 | 0 | 0 | 58 | 0 | 0 | 0 | 0 | 78 | 87 | 84 | 87 | 100 | 0 | 0 | 0 | 0 | 84 | 100 | 100 | 100 | 83 | 0 |
| Rv2879c | - | 189 | 82 | 87 | 85 | 86 | 82 | 82 | 83 | 74 | 85 | 88 | 89 | 89 | 88 | 94 | 96 | 96 | 100 | 91 | 92 | 92 | 92 | 92 | 100 | 100 | 100 | 94 | 92 |
| Rv2880c | - | 275 | 72 | 75 | 71 | 71 | 72 | 67 | 70 | 63 | 72 | 72 | 71 | 71 | 74 | 93 | 80 | 71 | 100 | 72 | 82 | 82 | 82 | 81 | 100 | 100 | 100 | 80 | 76 |
| Rv2881c | *cdsA* | 306 | 62 | 63 | 63 | 63 | 63 | 63 | 61 | 54 | 64 | 68 | 71 | 71 | 71 | 77 | 77 | 77 | 100 | 74 | 74 | 74 | 74 | 77 | 100 | 100 | 100 | 82 | 77 |
| Rv2882c | *frr* | 185 | 80 | 80 | 80 | 80 | 83 | 85 | 83 | 72 | 92 | 94 | 94 | 94 | 92 | 97 | 96 | 97 | 100 | 95 | 93 | 93 | 93 | 92 | 100 | 100 | 100 | 96 | 93 |
| Rv2883c | *pyrH* | 261 | 86 | 88 | 85 | 86 | 85 | 85 | 87 | 81 | 91 | 87 | 91 | 91 | 93 | 93 | 92 | 93 | 100 | 96 | 89 | 89 | 89 | 92 | 100 | 100 | 100 | 92 | 90 |
| Rv2884 | - | 252 | 47 | 47 | 47 | 43 | 43 | 58 | 46 | 59 | 65 | 65 | 66 | 66 | 65 | 78 | 78 | 77 | 100 | 65 | 65 | 65 | 65 | 65 | 100 | 100 | 100 | 76 | 65 |
| Rv2885c | - | 460 | 0 | 0 | 0 | 0 | 0 | 0 | 0 | 48 | 42 | 0 | 51 | 43 | 0 | 0 | 0 | 0 | 99 | 0 | 0 | 0 | 0 | 0 | 100 | 100 | 100 | 0 | 0 |
| Rv2886c | - | 295 | 0 | 0 | 0 | 0 | 0 | 0 | 0 | 0 | 0 | 0 | 0 | 0 | 0 | 0 | 0 | 0 | 100 | 0 | 0 | 0 | 0 | 0 | 100 | 100 | 100 | 0 | 0 |
| Rv2887 | - | 139 | 0 | 0 | 0 | 0 | 0 | 0 | 0 | 0 | 0 | 0 | 53 | 50 | 75 | 86 | 88 | 87 | 100 | 53 | 50 | 50 | 50 | 74 | 100 | 100 | 100 | 87 | 53 |
| Rv2888c | *amiC* | 473 | 40 | 38 | 41 | 39 | 44 | 41 | 45 | 73 | 61 | 64 | 63 | 64 | 60 | 83 | 82 | 80 | 100 | 76 | 79 | 79 | 79 | 76 | 100 | 100 | 100 | 82 | 77 |
| Rv2889c | *tsf* | 271 | 73 | 77 | 75 | 76 | 76 | 76 | 75 | 76 | 85 | 86 | 85 | 85 | 88 | 93 | 93 | 93 | 99 | 90 | 90 | 90 | 90 | 89 | 100 | 100 | 100 | 93 | 89 |
| Rv2890c | *rpsB* | 287 | 83 | 79 | 81 | 81 | 80 | 80 | 79 | 77 | 91 | 89 | 86 | 89 | 89 | 94 | 95 | 94 | 100 | 88 | 90 | 90 | 90 | 89 | 100 | 100 | 100 | 95 | 92 |
| Rv2891 | - | 249 | 46 | 56 | 52 | 58 | 58 | 56 | 49 | 57 | 58 | 66 | 50 | 61 | 69 | 76 | 79 | 77 | 100 | 76 | 78 | 78 | 78 | 72 | 100 | 100 | 100 | 48 | 77 |
| Rv2892c | *PPE45* | 408 | 0 | 0 | 0 | 0 | 0 | 0 | 0 | 0 | 43 | 0 | 39 | 38 | 55 | 56 | 61 | 58 | 100 | 48 | 50 | 50 | 50 | 49 | 100 | 100 | 100 | 61 | 43 |
| Rv2893 | - | 325 | 0 | 0 | 0 | 0 | 0 | 0 | 0 | 44 | 52 | 53 | 51 | 51 | 48 | 49 | 48 | 49 | 100 | 49 | 50 | 50 | 50 | 72 | 100 | 100 | 100 | 50 | 80 |
| Rv2894c | *xerC* | 298 | 62 | 63 | 65 | 63 | 63 | 66 | 55 | 68 | 77 | 78 | 56 | 44 | 83 | 90 | 93 | 90 | 100 | 88 | 88 | 88 | 88 | 85 | 100 | 100 | 100 | 92 | 89 |
| Rv2895c | *viuB* | 283 | 51 | 0 | 50 | 50 | 46 | 44 | 50 | 0 | 63 | 67 | 67 | 67 | 71 | 89 | 88 | 89 | 100 | 82 | 83 | 83 | 83 | 82 | 100 | 100 | 100 | 88 | 83 |
| Rv2896c | - | 389 | 55 | 57 | 51 | 54 | 52 | 54 | 49 | 54 | 58 | 62 | 70 | 62 | 71 | 84 | 84 | 84 | 99 | 74 | 78 | 77 | 77 | 79 | 100 | 100 | 100 | 84 | 76 |

| | | | | | | | | | | | | | | | | | | | | | | | | | | | | | |
|---|---|---|---|---|---|---|---|---|---|---|---|---|---|---|---|---|---|---|---|---|---|---|---|---|---|---|---|---|---|
| Rv2897c | - | 503 | 64 | 61 | 62 | 62 | 62 | 60 | 61 | 59 | 75 | 79 | 78 | 78 | 82 | 92 | 93 | 92 | 100 | 85 | 86 | 86 | 86 | 86 | 99 | 100 | 100 | 92 | 85 |
| Rv2898c | - | 128 | 57 | 0 | 57 | 60 | 58 | 54 | 62 | 58 | 66 | 67 | 64 | 64 | 71 | 85 | 86 | 89 | 100 | 80 | 79 | 80 | 80 | 81 | 100 | 100 | 100 | 86 | 78 |
| Rv2899c | *fdhD* | 276 | 61 | 0 | 61 | 56 | 0 | 0 | 0 | 57 | 69 | 72 | 75 | 75 | 79 | 82 | 84 | 83 | 100 | 80 | 80 | 80 | 80 | 80 | 100 | 100 | 100 | 83 | 77 |
| Rv2900c | *fdhF* | 779 | 0 | 0 | 64 | 63 | 0 | 0 | 0 | 71 | 81 | 82 | 82 | 82 | 86 | 41 | 41 | 41 | 100 | 88 | 88 | 87 | 87 | 86 | 100 | 100 | 100 | 42 | 88 |
| Rv2901c | - | 101 | 89 | 90 | 88 | 91 | 94 | 95 | 93 | 83 | 97 | 98 | 97 | 97 | 98 | 98 | 99 | 98 | 100 | 98 | 99 | 99 | 99 | 99 | 100 | 100 | 100 | 99 | 98 |
| Rv2902c | *rnhB* | 264 | 69 | 71 | 72 | 70 | 71 | 66 | 61 | 64 | 83 | 82 | 77 | 77 | 85 | 87 | 85 | 87 | 99 | 88 | 84 | 84 | 84 | 84 | 100 | 100 | 100 | 85 | 89 |
| Rv2903c | *lepB* | 294 | 60 | 63 | 64 | 61 | 59 | 59 | 54 | 50 | 63 | 68 | 66 | 66 | 72 | 83 | 83 | 83 | 100 | 73 | 76 | 76 | 76 | 78 | 100 | 100 | 100 | 87 | 76 |
| Rv2904c | *rplS* | 113 | 86 | 91 | 86 | 87 | 85 | 88 | 84 | 84 | 93 | 92 | 92 | 92 | 92 | 97 | 99 | 97 | 100 | 97 | 97 | 97 | 97 | 96 | 100 | 100 | 100 | 99 | 97 |
| Rv2905 | *lppW* | 314 | 0 | 0 | 0 | 0 | 0 | 0 | 0 | 0 | 41 | 59 | 53 | 53 | 71 | 81 | 84 | 81 | 100 | 68 | 69 | 69 | 69 | 69 | 100 | 100 | 100 | 84 | 69 |
| Rv2906c | *trmD* | 230 | 67 | 66 | 67 | 67 | 68 | 57 | 73 | 70 | 84 | 80 | 83 | 83 | 86 | 92 | 93 | 92 | 100 | 91 | 87 | 87 | 87 | 89 | 100 | 100 | 100 | 93 | 91 |
| Rv2907c | *rimM* | 176 | 55 | 58 | 61 | 62 | 52 | 51 | 49 | 58 | 73 | 74 | 74 | 75 | 77 | 82 | 82 | 82 | 100 | 76 | 80 | 80 | 80 | 73 | 100 | 100 | 100 | 82 | 77 |
| Rv2908c | - | 80 | 0 | 0 | 0 | 0 | 0 | 62 | 0 | 77 | 96 | 96 | 96 | 96 | 100 | 98 | 98 | 98 | 100 | 100 | 100 | 100 | 100 | 100 | 100 | 100 | 100 | 98 | 100 |
| Rv2909c | *rpsP* | 162 | 76 | 82 | 76 | 75 | 74 | 71 | 66 | 67 | 86 | 78 | 80 | 80 | 87 | 88 | 87 | 89 | 100 | 82 | 86 | 86 | 86 | 79 | 100 | 100 | 100 | 87 | 84 |
| Rv2910c | - | 147 | 0 | 0 | 0 | 0 | 0 | 0 | 0 | 53 | 66 | 0 | 0 | 0 | 75 | 90 | 90 | 90 | 100 | 79 | 86 | 86 | 86 | 85 | 100 | 100 | 100 | 84 | 81 |
| Rv2911 | *dacB2* | 291 | 57 | 56 | 54 | 57 | 0 | 54 | 0 | 0 | 62 | 62 | 58 | 59 | 78 | 88 | 88 | 88 | 100 | 76 | 78 | 78 | 78 | 80 | 100 | 100 | 100 | 88 | 77 |
| Rv2912c | - | 195 | 0 | 0 | 0 | 0 | 0 | 0 | 0 | 46 | 53 | 51 | 47 | 46 | 64 | 87 | 94 | 88 | 100 | 0 | 0 | 0 | 0 | 80 | 100 | 100 | 100 | 93 | 0 |
| Rv2913c | - | 611 | 0 | 0 | 0 | 0 | 0 | 0 | 0 | 39 | 73 | 75 | 74 | 75 | 81 | 90 | 90 | 90 | 100 | 70 | 72 | 72 | 72 | 86 | 100 | 100 | 100 | 90 | 72 |
| Rv2914c | *pknI* | 585 | 44 | 40 | 40 | 39 | 44 | 45 | 45 | 41 | 48 | 41 | 47 | 51 | 54 | 52 | 65 | 52 | 100 | 65 | 54 | 54 | 54 | 61 | 100 | 100 | 100 | 64 | 52 |
| Rv2915c | - | 370 | 0 | 0 | 0 | 0 | 0 | 0 | 0 | 65 | 77 | 79 | 78 | 78 | 82 | 88 | 91 | 88 | 100 | 82 | 82 | 82 | 82 | 81 | 100 | 100 | 99 | 90 | 83 |
| Rv2916c | *ffh* | 525 | 77 | 76 | 79 | 79 | 79 | 77 | 77 | 76 | 85 | 84 | 83 | 83 | 87 | 94 | 96 | 94 | 100 | 88 | 89 | 89 | 89 | 90 | 100 | 100 | 99 | 96 | 89 |
| Rv2917 | - | 626 | 73 | 77 | 75 | 77 | 75 | 74 | 73 | 71 | 79 | 81 | 77 | 78 | 82 | 85 | 84 | 84 | 99 | 85 | 84 | 85 | 85 | 83 | 99 | 100 | 99 | 87 | 83 |
| Rv2918c | *glnD* | 808 | 50 | 49 | 55 | 53 | 0 | 51 | 0 | 52 | 73 | 72 | 74 | 73 | 73 | 83 | 86 | 84 | 99 | 75 | 74 | 74 | 74 | 76 | 100 | 100 | 89 | 86 | 76 |
| Rv2919c | *glnB* | 112 | 83 | 86 | 88 | 86 | 85 | 86 | 0 | 80 | 93 | 94 | 94 | 94 | 94 | 99 | 99 | 99 | 100 | 94 | 94 | 94 | 94 | 94 | 100 | 100 | 100 | 98 | 94 |
| Rv2920c | *amt* | 477 | 66 | 50 | 65 | 67 | 63 | 64 | 60 | 65 | 85 | 69 | 84 | 71 | 84 | 93 | 91 | 93 | 100 | 70 | 71 | 71 | 71 | 68 | 100 | 100 | 100 | 89 | 68 |
| Rv2921c | *ftsY* | 422 | 81 | 75 | 72 | 79 | 76 | 82 | 71 | 72 | 68 | 69 | 73 | 72 | 75 | 88 | 85 | 91 | 100 | 79 | 80 | 80 | 80 | 74 | 100 | 100 | 100 | 86 | 83 |
| Rv2922A | *acyP* | 93 | 71 | 60 | 66 | 68 | 0 | 70 | 60 | 0 | 77 | 75 | 73 | 71 | 76 | 79 | 90 | 79 | 100 | 79 | 82 | 82 | 82 | 80 | 100 | 100 | 100 | 90 | 80 |
| Rv2922c | *smc* | 1205 | 64 | 61 | 64 | 63 | 61 | 64 | 61 | 66 | 76 | 77 | 78 | 78 | 82 | 90 | 91 | 90 | 99 | 85 | 84 | 85 | 85 | 84 | 100 | 100 | 99 | 91 | 86 |
| Rv2923c | - | 137 | 0 | 0 | 49 | 53 | 0 | 0 | 0 | 0 | 78 | 83 | 84 | 84 | 85 | 93 | 94 | 93 | 100 | 88 | 86 | 86 | 86 | 85 | 100 | 100 | 100 | 94 | 88 |
| Rv2924c | *fpg* | 289 | 64 | 63 | 66 | 65 | 60 | 43 | 66 | 66 | 74 | 73 | 74 | 74 | 76 | 90 | 91 | 90 | 100 | 85 | 86 | 85 | 85 | 84 | 100 | 100 | 100 | 91 | 85 |
| Rv2925c | *rnc* | 240 | 70 | 69 | 66 | 68 | 69 | 71 | 72 | 77 | 84 | 79 | 82 | 82 | 88 | 95 | 92 | 94 | 100 | 88 | 88 | 88 | 88 | 88 | 100 | 100 | 100 | 92 | 89 |
| Rv2926c | - | 207 | 50 | 47 | 51 | 51 | 49 | 48 | 52 | 68 | 73 | 73 | 71 | 71 | 75 | 79 | 83 | 80 | 100 | 74 | 78 | 78 | 78 | 77 | 100 | 100 | 100 | 83 | 78 |
| Rv2927c | - | 245 | 63 | 70 | 67 | 67 | 71 | 70 | 71 | 0 | 79 | 81 | 82 | 82 | 89 | 97 | 97 | 97 | 100 | 93 | 92 | 92 | 92 | 92 | 100 | 100 | 100 | 95 | 93 |
| Rv2928 | *tesA* | 261 | 0 | 0 | 0 | 0 | 0 | 0 | 0 | 0 | 52 | 0 | 43 | 50 | 52 | 42 | 85 | 45 | 100 | 64 | 61 | 61 | 61 | 49 | 100 | 100 | 100 | 85 | 66 |
| Rv2929 | - | 103 | 0 | 0 | 0 | 0 | 0 | 0 | 0 | 0 | 0 | 0 | 0 | 0 | 0 | 0 | 0 | 0 | 100 | 0 | 0 | 0 | 0 | 0 | 100 | 100 | 100 | 0 | 0 |
| Rv2930 | *fadD26* | 583 | 49 | 53 | 50 | 50 | 53 | 37 | 53 | 41 | 53 | 54 | 54 | 54 | 67 | 73 | 90 | 71 | 100 | 75 | 77 | 77 | 77 | 70 | 100 | 100 | 100 | 90 | 75 |
| Rv2931 | *ppsA* | 1876 | 53 | 56 | 56 | 55 | 51 | 0 | 54 | 41 | 54 | 56 | 48 | 47 | 48 | 52 | 89 | 48 | 99 | 70 | 75 | 75 | 75 | 47 | 99 | 100 | 98 | 89 | 70 |
| Rv2932 | *ppsB* | 1538 | 55 | 50 | 53 | 53 | 52 | 0 | 52 | 41 | 56 | 55 | 55 | 54 | 50 | 51 | 81 | 51 | 99 | 65 | 68 | 68 | 68 | 48 | 100 | 100 | 100 | 81 | 66 |
| Rv2933 | *ppsC* | 2188 | 53 | 52 | 52 | 55 | 51 | 45 | 50 | 50 | 48 | 50 | 50 | 48 | 52 | 53 | 84 | 53 | 100 | 76 | 53 | 53 | 53 | 54 | 99 | 100 | 100 | 84 | 78 |

| | | | | | | | | | | | | | | | | | | | | | | | | | | | | | |
|---|---|---|---|---|---|---|---|---|---|---|---|---|---|---|---|---|---|---|---|---|---|---|---|---|---|---|---|---|---|
| Rv2934 | *ppsD* | 1827 | 53 | 51 | 53 | 53 | 53 | 0 | 50 | 42 | 49 | 54 | 48 | 49 | 53 | 54 | 80 | 54 | 99 | 70 | 71 | 68 | 68 | 54 | 100 | 100 | 100 | 80 | 71 |
| Rv2935 | *ppsE* | 1488 | 48 | 45 | 47 | 47 | 45 | 0 | 46 | 44 | 48 | 48 | 51 | 51 | 57 | 50 | 88 | 51 | 100 | 72 | 72 | 72 | 72 | 52 | 100 | 100 | 100 | 88 | 72 |
| Rv2936 | *drrA* | 331 | 51 | 49 | 52 | 53 | 51 | 60 | 50 | 62 | 77 | 74 | 75 | 75 | 51 | 69 | 91 | 69 | 99 | 80 | 81 | 81 | 81 | 60 | 100 | 100 | 99 | 91 | 81 |
| Rv2937 | *drrB* | 289 | 0 | 0 | 0 | 0 | 0 | 0 | 0 | 44 | 58 | 54 | 56 | 55 | 0 | 47 | 80 | 50 | 100 | 64 | 67 | 67 | 67 | 0 | 100 | 100 | 100 | 80 | 65 |
| Rv2938 | *drrC* | 276 | 0 | 0 | 0 | 0 | 0 | 0 | 0 | 48 | 68 | 63 | 64 | 65 | 0 | 54 | 85 | 56 | 100 | 71 | 65 | 65 | 65 | 44 | 100 | 100 | 100 | 85 | 72 |
| Rv2939 | *papA5* | 422 | 0 | 0 | 0 | 0 | 0 | 0 | 0 | 0 | 47 | 0 | 0 | 0 | 45 | 0 | 0 | 0 | 100 | 67 | 67 | 67 | 67 | 0 | 100 | 100 | 100 | 72 | 66 |
| Rv2940c | *mas* | 2111 | 54 | 53 | 54 | 55 | 53 | 42 | 53 | 46 | 53 | 53 | 53 | 52 | 74 | 77 | 87 | 76 | 99 | 48 | 76 | 76 | 76 | 74 | 100 | 100 | 100 | 87 | 50 |
| Rv2941 | *fadD28* | 580 | 49 | 52 | 49 | 50 | 49 | 37 | 51 | 40 | 53 | 54 | 54 | 54 | 75 | 79 | 88 | 77 | 99 | 73 | 73 | 73 | 73 | 74 | 100 | 100 | 100 | 88 | 73 |
| Rv2942 | *mmpL7* | 920 | 0 | 0 | 0 | 0 | 0 | 0 | 0 | 39 | 0 | 0 | 0 | 0 | 47 | 47 | 72 | 47 | 100 | 43 | 43 | 43 | 43 | 44 | 100 | 100 | 100 | 72 | 44 |
| Rv2943 | - | 413 | 0 | 0 | 0 | 0 | 0 | 0 | 0 | 0 | 0 | 0 | 0 | 64 | 0 | 0 | 37 | 37 | 100 | 81 | 81 | 0 | 0 | 0 | 100 | 100 | 100 | 0 | 77 |
| Rv2943A | - | 176 | 0 | 0 | 0 | 0 | 0 | 0 | 0 | 0 | 0 | 0 | 52 | 0 | 0 | 0 | 0 | 56 | 100 | 96 | 0 | 0 | 0 | 0 | 100 | 100 | 100 | 0 | 96 |
| Rv2944 | - | 238 | 0 | 0 | 65 | 0 | 0 | 0 | 0 | 46 | 0 | 53 | 54 | 0 | 0 | 63 | 0 | 58 | 100 | 91 | 0 | 0 | 0 | 66 | 99 | 100 | 100 | 0 | 78 |
| Rv2945c | *lppX* | 233 | 0 | 0 | 0 | 0 | 0 | 0 | 0 | 0 | 41 | 0 | 0 | 0 | 51 | 52 | 83 | 52 | 100 | 49 | 50 | 50 | 50 | 45 | 100 | 100 | 100 | 83 | 48 |
| Rv2946c | *pks1* | 1616 | 47 | 45 | 44 | 42 | 43 | 53 | 41 | 45 | 40 | 41 | 49 | 48 | 49 | 69 | 89 | 71 | 99 | 49 | 49 | 48 | 48 | 50 | 100 | 100 | 100 | 88 | 48 |
| Rv2947c | *pks15* | 496 | 61 | 61 | 62 | 63 | 61 | 0 | 62 | 42 | 61 | 63 | 77 | 77 | 70 | 82 | 91 | 82 | 99 | 69 | 70 | 70 | 70 | 70 | 99 | 100 | 99 | 91 | 69 |
| Rv2948c | *fadD22* | 705 | 42 | 41 | 41 | 42 | 45 | 40 | 40 | 40 | 45 | 43 | 45 | 44 | 41 | 39 | 41 | 41 | 100 | 41 | 43 | 41 | 41 | 45 | 100 | 100 | 100 | 82 | 42 |
| Rv2949c | - | 199 | 0 | 0 | 0 | 0 | 0 | 0 | 0 | 0 | 0 | 0 | 0 | 0 | 0 | 0 | 0 | 0 | 100 | 0 | 0 | 0 | 0 | 0 | 100 | 100 | 100 | 75 | 0 |
| Rv2950c | *fadD29* | 582 | 50 | 53 | 50 | 51 | 52 | 0 | 51 | 40 | 54 | 55 | 55 | 55 | 67 | 72 | 70 | 70 | 99 | 73 | 74 | 74 | 74 | 69 | 100 | 100 | 100 | 86 | 74 |
| Rv2951c | - | 381 | 0 | 0 | 0 | 0 | 0 | 0 | 0 | 50 | 42 | 46 | 39 | 39 | 42 | 54 | 93 | 53 | 100 | 85 | 73 | 52 | 52 | 74 | 100 | 100 | 100 | 93 | 46 |
| Rv2952 | - | 270 | 0 | 0 | 0 | 0 | 0 | 0 | 0 | 0 | 0 | 0 | 0 | 0 | 69 | 84 | 0 | 0 | 100 | 0 | 48 | 0 | 0 | 66 | 100 | 100 | 100 | 87 | 0 |
| Rv2953 | - | 418 | 0 | 0 | 0 | 0 | 56 | 0 | 54 | 48 | 67 | 63 | 66 | 65 | 71 | 78 | 89 | 78 | 100 | 79 | 79 | 79 | 79 | 79 | 100 | 100 | 100 | 89 | 78 |
| Rv2954c | - | 241 | 0 | 0 | 0 | 0 | 0 | 0 | 0 | 0 | 0 | 0 | 0 | 0 | 0 | 0 | 0 | 0 | 100 | 0 | 0 | 0 | 0 | 0 | 94 | 100 | 100 | 0 | 0 |
| Rv2955c | - | 321 | 0 | 0 | 0 | 0 | 0 | 0 | 0 | 0 | 0 | 0 | 0 | 0 | 0 | 46 | 46 | 46 | 99 | 0 | 0 | 0 | 0 | 0 | 100 | 100 | 100 | 47 | 0 |
| Rv2956 | - | 243 | 0 | 0 | 0 | 0 | 0 | 0 | 0 | 0 | 0 | 0 | 0 | 0 | 79 | 0 | 0 | 99 | 0 | 0 | 48 | 48 | 0 | 100 | 100 | 100 | 0 | 47 | |
| Rv2957 | - | 275 | 0 | 0 | 0 | 0 | 0 | 0 | 0 | 49 | 0 | 0 | 0 | 0 | 0 | 77 | 0 | 73 | 100 | 0 | 0 | 0 | 0 | 0 | 100 | 100 | 100 | 0 | 0 |
| Rv2958c | - | 428 | 0 | 0 | 0 | 0 | 0 | 0 | 0 | 46 | 0 | 0 | 46 | 46 | 0 | 0 | 83 | 0 | 87 | 52 | 51 | 51 | 51 | 53 | 100 | 100 | 100 | 81 | 46 |
| Rv2959c | - | 245 | 0 | 0 | 0 | 0 | 0 | 0 | 0 | 0 | 0 | 0 | 0 | 0 | 0 | 0 | 0 | 0 | 100 | 0 | 0 | 0 | 0 | 0 | 100 | 100 | 100 | 0 | 0 |
| Rv2960c | - | 82 | 0 | 0 | 0 | 0 | 0 | 0 | 0 | 0 | 0 | 0 | 0 | 0 | 0 | 0 | 0 | 0 | 100 | 0 | 0 | 0 | 0 | 0 | 100 | 100 | 100 | 0 | 0 |
| Rv2961 | - | 129 | 0 | 0 | 0 | 0 | 0 | 0 | 0 | 0 | 0 | 0 | 0 | 0 | 0 | 0 | 0 | 0 | 100 | 0 | 0 | 0 | 0 | 0 | 100 | 100 | 100 | 0 | 0 |
| Rv2962c | - | 449 | 0 | 0 | 44 | 0 | 0 | 0 | 0 | 44 | 0 | 43 | 42 | 41 | 0 | 0 | 86 | 0 | 100 | 49 | 50 | 50 | 50 | 54 | 100 | 100 | 99 | 84 | 51 |
| Rv2963 | - | 406 | 0 | 0 | 0 | 0 | 0 | 0 | 0 | 0 | 0 | 0 | 0 | 0 | 0 | 90 | 89 | 91 | 100 | 0 | 0 | 0 | 0 | 0 | 100 | 100 | 100 | 88 | 0 |
| Rv2964 | *purU* | 310 | 0 | 0 | 57 | 57 | 49 | 47 | 45 | 64 | 59 | 59 | 60 | 60 | 85 | 0 | 94 | 0 | 100 | 89 | 51 | 51 | 51 | 84 | 100 | 100 | 100 | 93 | 88 |
| Rv2965c | *coaD* | 161 | 70 | 73 | 76 | 72 | 72 | 72 | 73 | 71 | 81 | 80 | 80 | 79 | 87 | 96 | 94 | 96 | 100 | 91 | 88 | 88 | 88 | 94 | 100 | 100 | 100 | 94 | 92 |
| Rv2966c | - | 188 | 55 | 57 | 54 | 55 | 51 | 59 | 55 | 65 | 68 | 70 | 70 | 70 | 71 | 87 | 87 | 87 | 100 | 77 | 77 | 77 | 77 | 78 | 100 | 100 | 100 | 87 | 75 |
| Rv2967c | *pca* | 1127 | 75 | 77 | 78 | 78 | 58 | 80 | 60 | 81 | 87 | 88 | 88 | 87 | 89 | 90 | 90 | 90 | 100 | 88 | 90 | 90 | 90 | 90 | 100 | 100 | 100 | 94 | 88 |
| Rv2968c | - | 210 | 0 | 0 | 56 | 55 | 56 | 0 | 60 | 54 | 68 | 0 | 58 | 0 | 77 | 80 | 79 | 79 | 100 | 76 | 77 | 76 | 76 | 74 | 100 | 100 | 100 | 79 | 78 |
| Rv2969c | - | 255 | 0 | 0 | 0 | 0 | 0 | 0 | 0 | 44 | 46 | 49 | 47 | 47 | 61 | 83 | 86 | 83 | 100 | 75 | 75 | 75 | 75 | 75 | 100 | 100 | 100 | 85 | 76 |
| Rv2970A | - | 56 | 0 | 0 | 0 | 0 | 0 | 0 | 0 | 0 | 0 | 0 | 0 | 0 | 0 | 0 | 0 | 0 | 100 | 0 | 85 | 85 | 85 | 88 | 100 | 100 | 100 | 0 | 0 |

| | | | | | | | | | | | | | | | | | | | | | | | | | | | | | |
|---|---|---|---|---|---|---|---|---|---|---|---|---|---|---|---|---|---|---|---|---|---|---|---|---|---|---|---|---|---|
| Rv2970c | *lipN* | 376 | 49 | 0 | 0 | 50 | 53 | 0 | 0 | 58 | 54 | 57 | 54 | 56 | 66 | 81 | 81 | 81 | 100 | 66 | 65 | 65 | 65 | 67 | 100 | 100 | 100 | 81 | 69 |
| Rv2971 | - | 282 | 62 | 62 | 62 | 60 | 64 | 61 | 60 | 59 | 66 | 68 | 69 | 68 | 69 | 87 | 87 | 87 | 100 | 80 | 79 | 79 | 79 | 81 | 100 | 100 | 100 | 90 | 79 |
| Rv2972c | - | 237 | 50 | 48 | 50 | 52 | 53 | 53 | 50 | 45 | 51 | 55 | 51 | 51 | 71 | 86 | 88 | 87 | 100 | 76 | 76 | 77 | 77 | 76 | 100 | 100 | 100 | 88 | 74 |
| Rv2973c | *recG* | 737 | 58 | 56 | 59 | 58 | 58 | 57 | 54 | 60 | 66 | 67 | 64 | 65 | 71 | 88 | 91 | 88 | 100 | 76 | 79 | 79 | 79 | 79 | 100 | 100 | 100 | 91 | 76 |
| Rv2974c | - | 470 | 49 | 48 | 50 | 50 | 45 | 43 | 43 | 52 | 54 | 57 | 57 | 57 | 63 | 83 | 87 | 83 | 100 | 70 | 72 | 72 | 72 | 74 | 100 | 100 | 100 | 87 | 72 |
| Rv2975c | - | 84 | 0 | 59 | 0 | 58 | 0 | 66 | 0 | 0 | 0 | 56 | 62 | 63 | 72 | 74 | 75 | 74 | 100 | 71 | 73 | 72 | 72 | 75 | 100 | 100 | 100 | 75 | 74 |
| Rv2976c | *ung* | 227 | 66 | 76 | 72 | 62 | 64 | 76 | 62 | 68 | 82 | 86 | 85 | 84 | 88 | 92 | 92 | 92 | 100 | 86 | 90 | 90 | 90 | 89 | 100 | 100 | 100 | 91 | 85 |
| Rv2977c | *thiL* | 333 | 55 | 52 | 58 | 55 | 53 | 57 | 56 | 57 | 66 | 65 | 67 | 67 | 69 | 86 | 86 | 86 | 100 | 72 | 79 | 79 | 79 | 77 | 100 | 100 | 100 | 84 | 72 |
| Rv2978c | - | 459 | 0 | 0 | 0 | 0 | 0 | 0 | 0 | 48 | 42 | 0 | 50 | 41 | 0 | 0 | 0 | 0 | 100 | 0 | 0 | 0 | 0 | 0 | 100 | 100 | 100 | 0 | 0 |
| Rv2979c | - | 194 | 0 | 0 | 0 | 0 | 0 | 0 | 0 | 0 | 0 | 0 | 0 | 0 | 0 | 0 | 0 | 0 | 98 | 0 | 0 | 0 | 0 | 0 | 100 | 100 | 100 | 0 | 0 |
| Rv2980 | - | 181 | 46 | 50 | 46 | 45 | 47 | 44 | 46 | 0 | 60 | 61 | 60 | 60 | 60 | 76 | 75 | 76 | 100 | 66 | 70 | 70 | 70 | 68 | 100 | 100 | 100 | 75 | 62 |
| Rv2981c | *ddl* | 373 | 70 | 70 | 71 | 70 | 65 | 71 | 66 | 65 | 80 | 79 | 81 | 81 | 83 | 92 | 91 | 92 | 98 | 87 | 88 | 88 | 88 | 88 | 99 | 100 | 99 | 91 | 87 |
| Rv2982c | *gpsA* | 334 | 74 | 72 | 76 | 75 | 69 | 71 | 69 | 69 | 78 | 81 | 83 | 82 | 81 | 88 | 89 | 88 | 99 | 84 | 86 | 86 | 86 | 85 | 99 | 100 | 99 | 89 | 85 |
| Rv2983 | - | 214 | 0 | 0 | 0 | 0 | 0 | 0 | 0 | 46 | 60 | 61 | 60 | 59 | 65 | 86 | 86 | 83 | 100 | 77 | 74 | 74 | 74 | 75 | 100 | 100 | 100 | 87 | 78 |
| Rv2984 | *ppk* | 742 | 0 | 0 | 0 | 0 | 0 | 0 | 0 | 69 | 84 | 85 | 85 | 85 | 87 | 91 | 92 | 91 | 100 | 90 | 89 | 89 | 89 | 89 | 100 | 100 | 100 | 92 | 89 |
| Rv2985 | *mutT1* | 317 | 56 | 57 | 61 | 59 | 51 | 54 | 54 | 50 | 66 | 64 | 69 | 68 | 73 | 86 | 86 | 86 | 100 | 77 | 76 | 76 | 76 | 76 | 100 | 100 | 100 | 85 | 78 |
| Rv2986c | *hupB* | 214 | 0 | 47 | 0 | 0 | 54 | 51 | 51 | 62 | 69 | 76 | 73 | 72 | 78 | 84 | 94 | 84 | 100 | 81 | 78 | 78 | 78 | 85 | 100 | 100 | 100 | 89 | 81 |
| Rv2987c | *leuD* | 198 | 74 | 80 | 81 | 82 | 82 | 80 | 79 | 76 | 85 | 88 | 87 | 87 | 91 | 93 | 93 | 94 | 100 | 89 | 90 | 90 | 90 | 93 | 100 | 100 | 100 | 93 | 90 |
| Rv2988c | *leuC* | 473 | 82 | 84 | 82 | 82 | 83 | 83 | 83 | 84 | 89 | 90 | 90 | 90 | 88 | 95 | 95 | 95 | 100 | 92 | 91 | 91 | 91 | 92 | 100 | 100 | 100 | 94 | 93 |
| Rv2989 | - | 233 | 60 | 65 | 61 | 63 | 62 | 64 | 63 | 66 | 81 | 79 | 81 | 80 | 86 | 91 | 90 | 91 | 100 | 87 | 87 | 87 | 87 | 87 | 100 | 100 | 100 | 89 | 89 |
| Rv2990c | - | 286 | 0 | 0 | 0 | 0 | 0 | 0 | 0 | 0 | 0 | 0 | 0 | 0 | 0 | 0 | 0 | 0 | 99 | 0 | 0 | 0 | 0 | 0 | 100 | 100 | 100 | 0 | 0 |
| Rv2991 | - | 163 | 0 | 0 | 0 | 0 | 0 | 0 | 0 | 72 | 75 | 80 | 82 | 82 | 84 | 91 | 96 | 91 | 99 | 82 | 83 | 83 | 83 | 83 | 99 | 100 | 100 | 96 | 82 |
| Rv2992c | *gltX* | 490 | 75 | 76 | 77 | 77 | 77 | 76 | 76 | 73 | 83 | 84 | 84 | 84 | 85 | 93 | 92 | 93 | 100 | 85 | 86 | 85 | 85 | 89 | 100 | 100 | 100 | 91 | 86 |
| Rv2993c | - | 239 | 66 | 65 | 69 | 70 | 66 | 68 | 70 | 72 | 85 | 86 | 85 | 85 | 89 | 93 | 94 | 93 | 100 | 91 | 91 | 90 | 90 | 88 | 100 | 100 | 100 | 92 | 91 |
| Rv2994 | - | 445 | 0 | 0 | 0 | 0 | 0 | 0 | 0 | 61 | 60 | 62 | 65 | 65 | 71 | 81 | 87 | 81 | 100 | 65 | 63 | 67 | 67 | 72 | 99 | 100 | 99 | 87 | 69 |
| Rv2995c | *leuB* | 336 | 81 | 80 | 79 | 81 | 78 | 79 | 79 | 80 | 87 | 82 | 84 | 84 | 88 | 92 | 93 | 92 | 99 | 90 | 91 | 91 | 91 | 90 | 100 | 100 | 100 | 92 | 90 |
| Rv2996c | *serA1* | 528 | 78 | 79 | 80 | 79 | 78 | 79 | 78 | 74 | 86 | 82 | 89 | 88 | 90 | 94 | 94 | 94 | 100 | 90 | 93 | 93 | 93 | 92 | 100 | 100 | 100 | 93 | 91 |
| Rv2997 | - | 480 | 51 | 0 | 52 | 50 | 0 | 50 | 0 | 42 | 62 | 56 | 58 | 56 | 56 | 88 | 86 | 87 | 99 | 81 | 80 | 81 | 81 | 82 | 100 | 100 | 100 | 62 | 81 |
| Rv2998 | - | 153 | 0 | 0 | 0 | 0 | 0 | 0 | 0 | 0 | 0 | 0 | 0 | 0 | 0 | 0 | 0 | 0 | 100 | 0 | 0 | 0 | 0 | 0 | 100 | 100 | 100 | 0 | 0 |
| Rv2998A | - | 67 | 0 | 0 | 0 | 0 | 0 | 0 | 0 | 72 | 0 | 67 | 67 | 0 | 0 | 65 | 0 | 65 | 100 | 0 | 0 | 0 | 0 | 0 | 100 | 100 | 100 | 0 | 0 |
| Rv2999 | *lppY* | 321 | 0 | 0 | 0 | 0 | 0 | 0 | 0 | 0 | 0 | 0 | 0 | 0 | 0 | 0 | 0 | 0 | 100 | 0 | 0 | 0 | 0 | 0 | 100 | 100 | 100 | 0 | 0 |
| Rv3000 | - | 219 | 58 | 58 | 43 | 55 | 56 | 53 | 0 | 54 | 56 | 49 | 58 | 58 | 45 | 60 | 59 | 60 | 100 | 55 | 56 | 57 | 57 | 59 | 100 | 100 | 100 | 59 | 55 |
| Rv3001c | *ilvC* | 333 | 83 | 80 | 84 | 82 | 85 | 80 | 84 | 78 | 92 | 89 | 89 | 89 | 93 | 95 | 95 | 95 | 100 | 93 | 93 | 93 | 93 | 92 | 100 | 100 | 100 | 94 | 93 |
| Rv3002c | *ilvH* | 168 | 77 | 77 | 76 | 76 | 73 | 78 | 75 | 83 | 88 | 88 | 88 | 88 | 89 | 96 | 96 | 96 | 100 | 91 | 92 | 92 | 92 | 93 | 100 | 100 | 100 | 96 | 91 |
| Rv3003c | *ilvB1* | 618 | 77 | 77 | 82 | 80 | 79 | 77 | 78 | 81 | 85 | 86 | 87 | 87 | 89 | 93 | 94 | 95 | 100 | 90 | 91 | 91 | 91 | 90 | 99 | 100 | 100 | 94 | 89 |
| Rv3004 | *cfp6* | 112 | 63 | 74 | 79 | 78 | 76 | 74 | 73 | 70 | 86 | 91 | 90 | 89 | 84 | 90 | 51 | 91 | 98 | 85 | 85 | 85 | 85 | 86 | 50 | 100 | 52 | 82 | 86 |
| Rv3005c | - | 279 | 48 | 42 | 47 | 48 | 48 | 53 | 52 | 0 | 58 | 66 | 51 | 56 | 65 | 79 | 79 | 79 | 100 | 70 | 72 | 72 | 72 | 69 | 100 | 100 | 100 | 87 | 69 |
| Rv3006 | *lppZ* | 373 | 0 | 0 | 40 | 0 | 0 | 0 | 0 | 37 | 59 | 59 | 62 | 62 | 79 | 96 | 95 | 96 | 99 | 89 | 87 | 87 | 87 | 90 | 100 | 100 | 100 | 95 | 89 |

| | | | | | | | | | | | | | | | | | | | | | | | | | | | | | |
|---|---|---|---|---|---|---|---|---|---|---|---|---|---|---|---|---|---|---|---|---|---|---|---|---|---|---|---|---|---|
| Rv3007c | - | 204 | 0 | 0 | 0 | 0 | 0 | 0 | 0 | 0 | 61 | 0 | 69 | 0 | 60 | 64 | 79 | 64 | 100 | 66 | 0 | 0 | 0 | 0 | 100 | 100 | 100 | 80 | 67 |
| Rv3008 | - | 207 | 0 | 0 | 0 | 0 | 0 | 0 | 0 | 0 | 0 | 0 | 0 | 0 | 0 | 0 | 0 | 0 | 100 | 0 | 0 | 0 | 0 | 0 | 100 | 100 | 100 | 0 | 0 |
| Rv3009c | *gatB* | 509 | 79 | 80 | 79 | 79 | 79 | 76 | 80 | 78 | 85 | 85 | 85 | 86 | 88 | 95 | 95 | 95 | 100 | 92 | 90 | 90 | 90 | 91 | 100 | 100 | 100 | 95 | 91 |
| Rv3010c | *pfkA* | 343 | 76 | 79 | 77 | 77 | 77 | 80 | 71 | 40 | 88 | 88 | 87 | 87 | 94 | 97 | 96 | 97 | 100 | 95 | 94 | 94 | 94 | 95 | 100 | 100 | 100 | 96 | 96 |
| Rv3011c | *gatA* | 494 | 83 | 84 | 82 | 83 | 82 | 82 | 81 | 80 | 86 | 87 | 87 | 87 | 90 | 95 | 95 | 95 | 99 | 92 | 93 | 93 | 93 | 93 | 99 | 100 | 100 | 95 | 93 |
| Rv3012c | *gatC* | 99 | 63 | 63 | 68 | 65 | 65 | 67 | 65 | 60 | 69 | 65 | 67 | 67 | 74 | 93 | 93 | 93 | 100 | 81 | 84 | 84 | 84 | 85 | 100 | 100 | 100 | 90 | 83 |
| Rv3013 | - | 218 | 59 | 62 | 61 | 61 | 57 | 61 | 55 | 51 | 81 | 80 | 80 | 80 | 86 | 93 | 91 | 93 | 100 | 83 | 84 | 84 | 84 | 86 | 100 | 100 | 100 | 90 | 84 |
| Rv3014c | *ligA* | 691 | 73 | 73 | 73 | 73 | 70 | 72 | 71 | 75 | 83 | 83 | 83 | 83 | 86 | 88 | 95 | 88 | 100 | 87 | 87 | 87 | 87 | 88 | 100 | 100 | 100 | 94 | 88 |
| Rv3015c | - | 337 | 51 | 47 | 49 | 49 | 0 | 44 | 44 | 54 | 67 | 65 | 66 | 66 | 71 | 84 | 84 | 84 | 100 | 81 | 80 | 80 | 80 | 82 | 100 | 100 | 100 | 87 | 78 |
| Rv3016 | *lpqA* | 209 | 0 | 0 | 0 | 0 | 0 | 0 | 0 | 0 | 0 | 0 | 0 | 0 | 0 | 74 | 72 | 72 | 100 | 63 | 66 | 66 | 66 | 0 | 100 | 100 | 100 | 74 | 66 |
| Rv3017c | *esxQ* | 120 | 0 | 0 | 0 | 0 | 0 | 0 | 0 | 0 | 0 | 0 | 0 | 0 | 67 | 78 | 80 | 78 | 100 | 74 | 75 | 75 | 75 | 75 | 100 | 100 | 100 | 80 | 74 |
| Rv3018c | *PPE46* | 434 | 0 | 0 | 0 | 0 | 0 | 0 | 0 | 0 | 39 | 37 | 39 | 40 | 48 | 53 | 56 | 52 | 99 | 58 | 50 | 50 | 50 | 51 | 86 | 100 | 100 | 50 | 55 |
| Rv3019c | *esxR* | 96 | 0 | 0 | 0 | 0 | 0 | 0 | 0 | 0 | 0 | 0 | 0 | 0 | 80 | 91 | 94 | 91 | 100 | 85 | 86 | 86 | 86 | 85 | 100 | 100 | 100 | 93 | 83 |
| Rv3020c | *esxS* | 97 | 0 | 0 | 0 | 0 | 0 | 0 | 0 | 0 | 0 | 0 | 0 | 0 | 78 | 90 | 93 | 90 | 100 | 84 | 83 | 84 | 84 | 83 | 100 | 100 | 100 | 93 | 86 |
| Rv3021c | *PPE47* | 358 | 0 | 0 | 0 | 0 | 0 | 0 | 0 | 0 | 0 | 0 | 0 | 0 | 42 | 47 | 52 | 46 | 99 | 51 | 43 | 43 | 43 | 44 | 99 | 99 | 99 | 46 | 43 |
| Rv3022A | *PE29* | 104 | 0 | 0 | 0 | 0 | 0 | 0 | 0 | 0 | 0 | 0 | 0 | 0 | 75 | 87 | 87 | 87 | 100 | 81 | 80 | 80 | 80 | 84 | 100 | 100 | 100 | 87 | 84 |
| Rv3022c | *PPE48* | 81 | 0 | 0 | 0 | 0 | 0 | 0 | 0 | 0 | 0 | 0 | 0 | 0 | 81 | 85 | 82 | 83 | 98 | 80 | 82 | 82 | 82 | 82 | 100 | 100 | 100 | 83 | 83 |
| Rv3023c | - | 415 | 45 | 66 | 74 | 0 | 73 | 0 | 69 | 59 | 0 | 40 | 87 | 58 | 0 | 60 | 0 | 79 | 99 | 89 | 42 | 79 | 79 | 59 | 100 | 100 | 100 | 43 | 89 |
| Rv3024c | *mnmA* | 367 | 69 | 72 | 70 | 73 | 70 | 73 | 69 | 68 | 83 | 82 | 82 | 82 | 84 | 92 | 90 | 92 | 100 | 87 | 86 | 86 | 86 | 86 | 100 | 100 | 100 | 90 | 88 |
| Rv3025c | *iscS* | 393 | 62 | 63 | 62 | 60 | 61 | 58 | 60 | 71 | 77 | 78 | 77 | 77 | 79 | 91 | 93 | 91 | 100 | 84 | 86 | 86 | 86 | 85 | 100 | 100 | 100 | 93 | 82 |
| Rv3026c | - | 304 | 0 | 0 | 0 | 0 | 0 | 0 | 0 | 0 | 51 | 57 | 59 | 58 | 71 | 80 | 80 | 80 | 100 | 72 | 70 | 70 | 70 | 73 | 100 | 100 | 100 | 80 | 71 |
| Rv3027c | - | 246 | 0 | 0 | 0 | 0 | 0 | 0 | 0 | 0 | 70 | 71 | 71 | 71 | 77 | 90 | 90 | 90 | 100 | 80 | 85 | 85 | 85 | 82 | 100 | 100 | 100 | 87 | 81 |
| Rv3028c | *fixB* | 318 | 65 | 65 | 67 | 67 | 72 | 74 | 73 | 72 | 87 | 88 | 89 | 89 | 93 | 95 | 95 | 95 | 99 | 93 | 91 | 91 | 91 | 93 | 100 | 100 | 100 | 95 | 92 |
| Rv3029c | *fixA* | 266 | 62 | 68 | 69 | 69 | 79 | 74 | 77 | 64 | 86 | 84 | 84 | 84 | 91 | 95 | 95 | 95 | 100 | 94 | 95 | 95 | 95 | 93 | 100 | 100 | 100 | 97 | 93 |
| Rv3030 | - | 274 | 0 | 0 | 0 | 0 | 0 | 0 | 0 | 52 | 76 | 76 | 77 | 77 | 79 | 82 | 85 | 83 | 100 | 79 | 82 | 82 | 82 | 82 | 100 | 100 | 100 | 85 | 81 |
| Rv3031 | - | 526 | 0 | 0 | 0 | 0 | 0 | 0 | 0 | 0 | 75 | 78 | 77 | 77 | 83 | 91 | 93 | 91 | 100 | 87 | 88 | 88 | 88 | 86 | 100 | 100 | 100 | 92 | 86 |
| Rv3032 | - | 414 | 41 | 42 | 39 | 40 | 41 | 40 | 42 | 43 | 79 | 81 | 79 | 79 | 86 | 92 | 92 | 93 | 100 | 86 | 89 | 89 | 89 | 89 | 100 | 100 | 97 | 92 | 86 |
| Rv3033 | - | 182 | 0 | 0 | 0 | 0 | 57 | 0 | 0 | 0 | 64 | 58 | 0 | 0 | 0 | 0 | 88 | 0 | 100 | 52 | 0 | 0 | 0 | 0 | 100 | 100 | 100 | 87 | 50 |
| Rv3034c | - | 300 | 50 | 52 | 52 | 53 | 0 | 0 | 0 | 49 | 90 | 86 | 81 | 85 | 89 | 96 | 96 | 96 | 100 | 94 | 91 | 91 | 91 | 94 | 100 | 100 | 100 | 96 | 91 |
| Rv3035 | - | 360 | 0 | 0 | 0 | 0 | 0 | 0 | 0 | 0 | 52 | 51 | 55 | 55 | 70 | 87 | 89 | 87 | 100 | 74 | 75 | 74 | 74 | 75 | 100 | 100 | 100 | 89 | 74 |
| Rv3036c | *TB22.2* | 227 | 0 | 0 | 0 | 0 | 0 | 0 | 0 | 0 | 0 | 0 | 0 | 0 | 49 | 82 | 85 | 83 | 100 | 63 | 70 | 70 | 70 | 64 | 100 | 100 | 100 | 85 | 70 |
| Rv3037c | - | 358 | 57 | 57 | 58 | 59 | 56 | 52 | 55 | 52 | 70 | 70 | 69 | 69 | 72 | 84 | 82 | 83 | 99 | 76 | 76 | 76 | 76 | 77 | 100 | 100 | 100 | 82 | 75 |
| Rv3038c | - | 327 | 0 | 0 | 0 | 0 | 0 | 0 | 50 | 54 | 80 | 83 | 84 | 82 | 88 | 91 | 92 | 91 | 100 | 90 | 90 | 90 | 90 | 91 | 100 | 100 | 100 | 93 | 90 |
| Rv3039c | *echA17* | 254 | 52 | 0 | 48 | 0 | 51 | 49 | 51 | 60 | 64 | 64 | 66 | 67 | 70 | 92 | 86 | 92 | 100 | 77 | 74 | 74 | 74 | 79 | 100 | 100 | 100 | 85 | 81 |
| Rv3040c | - | 288 | 54 | 51 | 51 | 51 | 56 | 53 | 54 | 57 | 60 | 64 | 65 | 65 | 71 | 83 | 88 | 84 | 100 | 78 | 79 | 79 | 79 | 79 | 99 | 100 | 100 | 88 | 80 |
| Rv3041c | - | 287 | 81 | 80 | 79 | 81 | 81 | 79 | 79 | 77 | 86 | 86 | 84 | 84 | 87 | 92 | 92 | 92 | 100 | 93 | 87 | 87 | 87 | 88 | 100 | 100 | 100 | 93 | 89 |
| Rv3042c | *serB2* | 409 | 70 | 70 | 70 | 69 | 73 | 71 | 72 | 70 | 80 | 79 | 79 | 79 | 82 | 90 | 92 | 90 | 99 | 87 | 87 | 87 | 87 | 88 | 99 | 100 | 99 | 92 | 88 |
| Rv3043c | *ctaD* | 573 | 84 | 86 | 86 | 85 | 85 | 87 | 85 | 82 | 92 | 90 | 91 | 91 | 94 | 96 | 97 | 96 | 100 | 95 | 95 | 95 | 95 | 92 | 100 | 100 | 100 | 97 | 95 |

| | | | | | | | | | | | | | | | | | | | | | | | | | | | | | |
|---|---|---|---|---|---|---|---|---|---|---|---|---|---|---|---|---|---|---|---|---|---|---|---|---|---|---|---|---|---|
| Rv3044 | *fecB* | 359 | 45 | 47 | 0 | 46 | 0 | 41 | 0 | 0 | 53 | 54 | 50 | 50 | 70 | 84 | 89 | 84 | 100 | 71 | 74 | 74 | 74 | 71 | 100 | 100 | 100 | 89 | 72 |
| Rv3045 | *adhC* | 346 | 70 | 44 | 70 | 67 | 69 | 41 | 43 | 47 | 76 | 80 | 79 | 79 | 83 | 90 | 94 | 89 | 100 | 85 | 87 | 87 | 87 | 85 | 100 | 100 | 100 | 94 | 85 |
| Rv3046c | - | 124 | 0 | 0 | 0 | 0 | 0 | 0 | 0 | 0 | 47 | 62 | 57 | 57 | 64 | 84 | 84 | 84 | 100 | 78 | 55 | 55 | 55 | 47 | 100 | 100 | 100 | 85 | 79 |
| Rv3047c | - | 94 | 0 | 0 | 0 | 0 | 0 | 0 | 0 | 0 | 0 | 0 | 0 | 0 | 0 | 0 | 0 | 0 | 100 | 0 | 0 | 0 | 0 | 0 | 100 | 100 | 100 | 0 | 0 |
| Rv3048c | *nrdF2* | 324 | 88 | 88 | 88 | 87 | 85 | 87 | 84 | 0 | 93 | 93 | 94 | 94 | 94 | 96 | 98 | 96 | 100 | 97 | 97 | 97 | 97 | 95 | 100 | 100 | 100 | 97 | 96 |
| Rv3049c | - | 524 | 53 | 0 | 42 | 0 | 41 | 0 | 41 | 61 | 66 | 67 | 68 | 69 | 76 | 89 | 90 | 89 | 100 | 78 | 63 | 63 | 63 | 79 | 100 | 100 | 100 | 89 | 78 |
| Rv3050c | - | 246 | 0 | 0 | 0 | 0 | 0 | 0 | 0 | 0 | 47 | 47 | 48 | 45 | 63 | 88 | 93 | 88 | 100 | 84 | 86 | 86 | 86 | 89 | 100 | 100 | 100 | 91 | 85 |
| Rv3051c | *nrdE* | 693 | 88 | 88 | 88 | 88 | 90 | 87 | 88 | 39 | 96 | 95 | 96 | 95 | 97 | 98 | 98 | 98 | 100 | 97 | 98 | 98 | 98 | 97 | 100 | 100 | 100 | 98 | 97 |
| Rv3052c | *nrdI* | 150 | 72 | 72 | 74 | 77 | 75 | 68 | 72 | 0 | 76 | 79 | 79 | 79 | 75 | 92 | 92 | 92 | 100 | 85 | 86 | 86 | 86 | 84 | 100 | 100 | 100 | 91 | 88 |
| Rv3053c | *nrdH* | 79 | 86 | 84 | 85 | 84 | 87 | 89 | 90 | 0 | 88 | 86 | 86 | 86 | 90 | 94 | 94 | 94 | 100 | 97 | 95 | 95 | 95 | 93 | 100 | 100 | 100 | 93 | 93 |
| Rv3054c | - | 184 | 53 | 52 | 0 | 0 | 0 | 0 | 56 | 63 | 71 | 73 | 71 | 77 | 67 | 87 | 87 | 87 | 100 | 80 | 77 | 77 | 77 | 77 | 100 | 100 | 100 | 86 | 81 |
| Rv3055 | - | 204 | 0 | 0 | 0 | 0 | 0 | 0 | 0 | 61 | 65 | 64 | 65 | 66 | 67 | 86 | 88 | 85 | 100 | 75 | 77 | 77 | 77 | 79 | 100 | 100 | 100 | 88 | 82 |
| Rv3056 | *dinP* | 346 | 50 | 49 | 50 | 49 | 46 | 49 | 48 | 64 | 65 | 49 | 65 | 64 | 51 | 86 | 50 | 86 | 100 | 80 | 83 | 83 | 83 | 82 | 100 | 100 | 100 | 50 | 81 |
| Rv3057c | - | 287 | 48 | 50 | 53 | 50 | 64 | 56 | 46 | 68 | 74 | 74 | 77 | 77 | 81 | 90 | 93 | 89 | 100 | 85 | 83 | 84 | 84 | 86 | 100 | 100 | 99 | 93 | 84 |
| Rv3058c | - | 216 | 0 | 0 | 0 | 0 | 0 | 0 | 0 | 0 | 0 | 0 | 0 | 0 | 69 | 84 | 84 | 84 | 100 | 69 | 69 | 69 | 69 | 71 | 100 | 100 | 100 | 86 | 70 |
| Rv3059 | *cyp136* | 492 | 0 | 0 | 0 | 0 | 43 | 0 | 0 | 61 | 60 | 60 | 57 | 61 | 81 | 85 | 91 | 85 | 99 | 79 | 87 | 84 | 84 | 80 | 100 | 100 | 100 | 90 | 79 |
| Rv3060c | - | 490 | 0 | 0 | 0 | 0 | 0 | 0 | 0 | 48 | 0 | 0 | 46 | 55 | 44 | 80 | 81 | 80 | 100 | 47 | 46 | 44 | 44 | 43 | 100 | 100 | 100 | 77 | 44 |
| Rv3061c | *fadE22* | 721 | 0 | 0 | 0 | 0 | 42 | 44 | 42 | 54 | 54 | 57 | 56 | 65 | 55 | 90 | 91 | 90 | 100 | 65 | 65 | 65 | 65 | 58 | 100 | 100 | 100 | 91 | 65 |
| Rv3062 | *ligB* | 507 | 0 | 0 | 0 | 0 | 0 | 0 | 0 | 66 | 62 | 46 | 64 | 63 | 40 | 85 | 85 | 85 | 99 | 79 | 80 | 80 | 80 | 79 | 99 | 100 | 99 | 84 | 77 |
| Rv3063 | *cstA* | 758 | 46 | 77 | 0 | 77 | 0 | 0 | 0 | 0 | 79 | 83 | 82 | 82 | 79 | 83 | 86 | 84 | 99 | 85 | 84 | 84 | 84 | 86 | 99 | 100 | 99 | 87 | 86 |
| Rv3064c | - | 141 | 0 | 0 | 0 | 0 | 0 | 0 | 0 | 0 | 0 | 0 | 60 | 54 | 64 | 0 | 0 | 0 | 100 | 68 | 65 | 65 | 65 | 68 | 100 | 100 | 100 | 0 | 64 |
| Rv3065 | *mmr* | 107 | 0 | 0 | 0 | 0 | 0 | 0 | 0 | 64 | 68 | 56 | 0 | 0 | 58 | 91 | 91 | 91 | 100 | 58 | 60 | 60 | 60 | 62 | 100 | 100 | 100 | 83 | 80 |
| Rv3066 | - | 202 | 0 | 0 | 0 | 0 | 0 | 0 | 0 | 0 | 0 | 48 | 0 | 0 | 0 | 0 | 43 | 43 | 100 | 46 | 49 | 49 | 49 | 0 | 100 | 100 | 100 | 80 | 46 |
| Rv3067 | - | 136 | 0 | 0 | 0 | 0 | 0 | 0 | 0 | 0 | 0 | 0 | 0 | 0 | 0 | 49 | 56 | 56 | 100 | 0 | 0 | 0 | 0 | 0 | 100 | 100 | 100 | 62 | 0 |
| Rv3068c | *pgmA* | 547 | 80 | 80 | 79 | 79 | 76 | 79 | 0 | 80 | 79 | 83 | 83 | 83 | 84 | 91 | 92 | 90 | 100 | 85 | 85 | 85 | 85 | 87 | 100 | 100 | 100 | 91 | 85 |
| Rv3069 | *ccrB* | 132 | 0 | 0 | 0 | 0 | 0 | 50 | 0 | 50 | 45 | 52 | 50 | 51 | 48 | 91 | 93 | 91 | 100 | 82 | 83 | 83 | 83 | 81 | 100 | 100 | 100 | 92 | 84 |
| Rv3070 | *ccrB* | 126 | 0 | 0 | 0 | 0 | 0 | 57 | 0 | 59 | 60 | 54 | 57 | 59 | 57 | 89 | 88 | 89 | 100 | 79 | 75 | 75 | 75 | 83 | 100 | 100 | 100 | 87 | 80 |
| Rv3071 | - | 369 | 0 | 0 | 0 | 0 | 0 | 0 | 0 | 0 | 0 | 0 | 0 | 0 | 0 | 67 | 67 | 67 | 100 | 61 | 58 | 58 | 58 | 69 | 100 | 100 | 100 | 83 | 65 |
| Rv3072c | - | 174 | 0 | 0 | 0 | 0 | 0 | 0 | 0 | 56 | 83 | 62 | 82 | 83 | 63 | 57 | 87 | 56 | 100 | 71 | 60 | 60 | 60 | 85 | 100 | 100 | 100 | 90 | 57 |
| Rv3073c | - | 118 | 55 | 0 | 57 | 59 | 0 | 61 | 46 | 58 | 63 | 0 | 56 | 56 | 57 | 80 | 81 | 81 | 100 | 0 | 0 | 0 | 0 | 66 | 100 | 100 | 100 | 81 | 0 |
| Rv3074 | - | 424 | 0 | 0 | 0 | 0 | 0 | 0 | 0 | 63 | 0 | 63 | 65 | 66 | 74 | 87 | 91 | 86 | 100 | 70 | 83 | 80 | 80 | 74 | 100 | 100 | 100 | 89 | 73 |
| Rv3075c | - | 307 | 0 | 0 | 40 | 0 | 44 | 39 | 0 | 44 | 45 | 62 | 66 | 66 | 64 | 89 | 92 | 88 | 100 | 87 | 90 | 90 | 90 | 88 | 100 | 100 | 100 | 92 | 89 |
| Rv3076 | - | 158 | 0 | 0 | 0 | 0 | 0 | 0 | 0 | 0 | 0 | 0 | 0 | 0 | 0 | 0 | 0 | 0 | 100 | 52 | 66 | 66 | 66 | 69 | 100 | 100 | 100 | 77 | 60 |
| Rv3077 | - | 603 | 0 | 0 | 0 | 0 | 0 | 0 | 0 | 0 | 0 | 45 | 39 | 39 | 42 | 0 | 92 | 0 | 99 | 84 | 87 | 87 | 87 | 89 | 99 | 100 | 99 | 92 | 85 |
| Rv3078 | *hab* | 133 | 0 | 0 | 0 | 0 | 0 | 0 | 0 | 0 | 0 | 0 | 0 | 0 | 0 | 0 | 0 | 0 | 100 | 0 | 0 | 0 | 0 | 56 | 100 | 100 | 100 | 0 | 0 |
| Rv3079c | - | 275 | 0 | 0 | 45 | 38 | 0 | 0 | 0 | 55 | 56 | 60 | 55 | 55 | 55 | 56 | 87 | 56 | 100 | 66 | 54 | 54 | 54 | 65 | 100 | 100 | 100 | 87 | 65 |
| Rv3080c | *pknK* | 1110 | 46 | 47 | 46 | 47 | 46 | 45 | 48 | 47 | 61 | 60 | 70 | 70 | 51 | 50 | 76 | 49 | 99 | 49 | 72 | 72 | 72 | 67 | 100 | 100 | 100 | 73 | 66 |
| Rv3081 | - | 412 | 0 | 0 | 0 | 0 | 0 | 0 | 0 | 0 | 0 | 0 | 0 | 0 | 0 | 0 | 0 | 0 | 100 | 0 | 0 | 0 | 0 | 0 | 100 | 100 | 100 | 0 | 0 |

| | | | | | | | | | | | | | | | | | | | | | | | | | | | | | |
|---|---|---|---|---|---|---|---|---|---|---|---|---|---|---|---|---|---|---|---|---|---|---|---|---|---|---|---|---|---|
| Rv3082c | *virS* | 340 | 0 | 0 | 0 | 0 | 0 | 0 | 0 | 0 | 50 | 44 | 47 | 80 | 50 | 0 | 0 | 0 | 99 | 0 | 0 | 0 | 0 | 0 | 100 | 100 | 100 | 50 | 0 |
| Rv3083 | - | 495 | 42 | 0 | 0 | 0 | 51 | 0 | 0 | 68 | 71 | 73 | 68 | 86 | 66 | 63 | 65 | 63 | 100 | 65 | 63 | 63 | 63 | 66 | 100 | 100 | 100 | 65 | 65 |
| Rv3084 | *lipR* | 308 | 44 | 0 | 0 | 40 | 42 | 0 | 0 | 46 | 48 | 47 | 50 | 82 | 49 | 47 | 45 | 47 | 100 | 49 | 48 | 47 | 47 | 46 | 100 | 100 | 100 | 45 | 46 |
| Rv3085 | - | 276 | 49 | 45 | 52 | 49 | 53 | 53 | 48 | 75 | 86 | 86 | 86 | 86 | 83 | 81 | 81 | 81 | 100 | 78 | 79 | 79 | 79 | 82 | 100 | 100 | 100 | 80 | 81 |
| Rv3086 | *adhD* | 368 | 50 | 44 | 47 | 48 | 50 | 48 | 49 | 52 | 73 | 95 | 93 | 93 | 69 | 66 | 64 | 64 | 100 | 68 | 69 | 69 | 69 | 83 | 100 | 100 | 100 | 65 | 68 |
| Rv3087 | - | 472 | 0 | 0 | 0 | 0 | 0 | 0 | 0 | 47 | 47 | 50 | 47 | 48 | 49 | 49 | 59 | 48 | 99 | 51 | 52 | 52 | 52 | 50 | 100 | 100 | 100 | 59 | 50 |
| Rv3088 | - | 474 | 0 | 0 | 0 | 0 | 0 | 0 | 0 | 46 | 49 | 47 | 47 | 48 | 43 | 47 | 81 | 47 | 100 | 45 | 45 | 45 | 45 | 46 | 100 | 100 | 99 | 78 | 48 |
| Rv3089 | *fadD13* | 503 | 47 | 45 | 46 | 53 | 46 | 46 | 46 | 51 | 53 | 55 | 55 | 54 | 54 | 52 | 54 | 54 | 100 | 55 | 55 | 55 | 55 | 52 | 100 | 100 | 100 | 84 | 53 |
| Rv3090 | - | 295 | 0 | 0 | 0 | 0 | 0 | 0 | 0 | 0 | 0 | 0 | 0 | 0 | 0 | 0 | 92 | 0 | 100 | 0 | 0 | 0 | 0 | 0 | 99 | 100 | 100 | 96 | 0 |
| Rv3091 | - | 563 | 0 | 0 | 0 | 0 | 0 | 0 | 0 | 0 | 0 | 0 | 0 | 0 | 0 | 0 | 85 | 0 | 100 | 0 | 0 | 0 | 0 | 0 | 100 | 100 | 100 | 85 | 0 |
| Rv3092c | - | 306 | 65 | 69 | 66 | 67 | 68 | 0 | 68 | 79 | 0 | 76 | 0 | 72 | 0 | 0 | 0 | 0 | 100 | 84 | 81 | 81 | 81 | 79 | 100 | 100 | 99 | 82 | 84 |
| Rv3093c | - | 334 | 0 | 0 | 0 | 0 | 0 | 0 | 0 | 39 | 41 | 54 | 40 | 40 | 41 | 41 | 85 | 41 | 99 | 41 | 40 | 43 | 43 | 41 | 100 | 100 | 99 | 84 | 42 |
| Rv3094c | - | 376 | 0 | 0 | 0 | 0 | 0 | 0 | 0 | 44 | 49 | 45 | 49 | 49 | 45 | 46 | 90 | 47 | 100 | 46 | 46 | 45 | 45 | 43 | 100 | 100 | 100 | 87 | 45 |
| Rv3095 | - | 158 | 0 | 0 | 58 | 0 | 0 | 0 | 0 | 0 | 61 | 61 | 56 | 60 | 61 | 54 | 87 | 54 | 100 | 52 | 45 | 45 | 45 | 61 | 100 | 100 | 100 | 87 | 51 |
| Rv3096 | - | 379 | 0 | 0 | 0 | 0 | 0 | 0 | 0 | 0 | 0 | 0 | 0 | 0 | 0 | 82 | 87 | 82 | 99 | 79 | 80 | 81 | 81 | 82 | 100 | 100 | 100 | 86 | 79 |
| Rv3097c | *lipY* | 437 | 0 | 0 | 0 | 0 | 40 | 0 | 0 | 42 | 42 | 44 | 47 | 44 | 39 | 58 | 56 | 56 | 99 | 49 | 39 | 43 | 43 | 42 | 99 | 100 | 100 | 74 | 50 |
| Rv3098c | - | 150 | 0 | 0 | 0 | 0 | 0 | 0 | 0 | 0 | 0 | 0 | 0 | 0 | 0 | 0 | 0 | 0 | 100 | 0 | 0 | 0 | 0 | 0 | 100 | 100 | 100 | 0 | 0 |
| Rv3099c | - | 283 | 0 | 0 | 0 | 0 | 0 | 0 | 0 | 48 | 56 | 57 | 60 | 61 | 56 | 80 | 87 | 79 | 100 | 0 | 51 | 49 | 49 | 0 | 100 | 100 | 100 | 87 | 48 |
| Rv3100c | *smpB* | 160 | 78 | 80 | 82 | 82 | 82 | 82 | 81 | 73 | 88 | 87 | 89 | 89 | 86 | 95 | 94 | 95 | 100 | 90 | 93 | 93 | 93 | 89 | 100 | 100 | 100 | 94 | 91 |
| Rv3101c | *ftsX* | 297 | 61 | 60 | 61 | 60 | 65 | 64 | 65 | 50 | 65 | 76 | 76 | 76 | 82 | 92 | 94 | 92 | 100 | 91 | 92 | 92 | 92 | 92 | 100 | 100 | 100 | 94 | 92 |
| Rv3102c | *ftsE* | 229 | 78 | 85 | 84 | 83 | 83 | 81 | 82 | 78 | 87 | 89 | 89 | 89 | 93 | 97 | 99 | 97 | 100 | 95 | 96 | 96 | 96 | 96 | 100 | 100 | 100 | 99 | 95 |
| Rv3103c | - | 145 | 0 | 0 | 0 | 0 | 0 | 0 | 0 | 0 | 0 | 0 | 41 | 45 | 45 | 67 | 65 | 67 | 100 | 53 | 56 | 57 | 57 | 57 | 100 | 100 | 100 | 64 | 54 |
| Rv3104c | - | 308 | 52 | 50 | 47 | 48 | 52 | 47 | 48 | 56 | 75 | 74 | 76 | 76 | 78 | 84 | 94 | 84 | 100 | 82 | 80 | 80 | 80 | 82 | 100 | 100 | 100 | 94 | 81 |
| Rv3105c | *prfB* | 378 | 83 | 84 | 82 | 82 | 83 | 82 | 84 | 79 | 88 | 86 | 88 | 89 | 91 | 97 | 97 | 97 | 100 | 93 | 93 | 93 | 93 | 94 | 99 | 100 | 100 | 97 | 94 |
| Rv3106 | *fprA* | 456 | 52 | 52 | 54 | 57 | 53 | 54 | 51 | 54 | 63 | 62 | 63 | 63 | 73 | 89 | 88 | 88 | 100 | 82 | 85 | 85 | 85 | 85 | 100 | 100 | 100 | 88 | 83 |
| Rv3107c | *agpS* | 527 | 0 | 0 | 0 | 0 | 0 | 39 | 0 | 49 | 50 | 50 | 49 | 49 | 47 | 50 | 87 | 50 | 100 | 49 | 49 | 49 | 49 | 48 | 100 | 100 | 100 | 83 | 50 |
| Rv3108 | - | 146 | 0 | 0 | 0 | 0 | 0 | 0 | 0 | 0 | 0 | 0 | 0 | 0 | 0 | 0 | 82 | 0 | 100 | 0 | 0 | 0 | 0 | 0 | 100 | 100 | 100 | 82 | 0 |
| Rv3109 | *moaA1* | 359 | 55 | 56 | 58 | 56 | 54 | 0 | 55 | 56 | 58 | 60 | 57 | 57 | 56 | 52 | 54 | 52 | 99 | 55 | 53 | 53 | 53 | 54 | 100 | 100 | 100 | 53 | 57 |
| Rv3110 | *moaB1* | 131 | 0 | 0 | 0 | 0 | 0 | 0 | 0 | 0 | 0 | 55 | 51 | 52 | 0 | 0 | 0 | 0 | 100 | 0 | 0 | 0 | 0 | 0 | 100 | 100 | 100 | 0 | 0 |
| Rv3111 | *moaC* | 170 | 66 | 68 | 62 | 63 | 59 | 0 | 61 | 66 | 62 | 62 | 64 | 63 | 63 | 67 | 67 | 67 | 100 | 65 | 66 | 66 | 66 | 66 | 100 | 100 | 100 | 65 | 66 |
| Rv3112 | *moaD1* | 83 | 0 | 0 | 0 | 0 | 0 | 0 | 0 | 0 | 0 | 0 | 0 | 0 | 0 | 0 | 0 | 0 | 100 | 0 | 0 | 0 | 0 | 0 | 100 | 100 | 100 | 0 | 0 |
| Rv3113 | - | 222 | 0 | 0 | 0 | 0 | 0 | 0 | 0 | 0 | 0 | 0 | 0 | 0 | 46 | 0 | 0 | 0 | 98 | 0 | 0 | 0 | 0 | 0 | 99 | 100 | 100 | 0 | 0 |
| Rv3114 | - | 176 | 0 | 0 | 0 | 0 | 0 | 50 | 0 | 43 | 0 | 0 | 0 | 0 | 0 | 0 | 0 | 0 | 99 | 0 | 0 | 0 | 0 | 0 | 100 | 100 | 100 | 0 | 0 |
| Rv3115 | - | 415 | 45 | 66 | 74 | 0 | 73 | 0 | 69 | 59 | 0 | 40 | 87 | 58 | 0 | 60 | 0 | 79 | 99 | 89 | 42 | 79 | 79 | 59 | 100 | 100 | 100 | 43 | 89 |
| Rv3116 | *moeB2* | 389 | 54 | 55 | 59 | 58 | 62 | 49 | 55 | 73 | 73 | 71 | 71 | 71 | 72 | 70 | 70 | 70 | 100 | 71 | 71 | 72 | 72 | 71 | 100 | 100 | 100 | 70 | 70 |
| Rv3117 | *cysA3* | 277 | 59 | 61 | 64 | 63 | 59 | 60 | 43 | 86 | 90 | 87 | 86 | 89 | 92 | 93 | 93 | 93 | 100 | 90 | 91 | 91 | 91 | 93 | 100 | 100 | 100 | 93 | 92 |
| Rv3118 | *sseC1* | 100 | 0 | 0 | 0 | 0 | 0 | 0 | 0 | 69 | 83 | 82 | 79 | 79 | 91 | 96 | 95 | 96 | 100 | 94 | 93 | 93 | 93 | 92 | 100 | 100 | 100 | 95 | 93 |
| Rv3119 | *moaE1* | 147 | 58 | 55 | 55 | 55 | 51 | 0 | 49 | 57 | 60 | 64 | 63 | 64 | 59 | 56 | 57 | 56 | 86 | 58 | 57 | 57 | 57 | 60 | 100 | 100 | 100 | 57 | 58 |

| | | | | | | | | | | | | | | | | | | | | | | | | | | | | | |
|---|---|---|---|---|---|---|---|---|---|---|---|---|---|---|---|---|---|---|---|---|---|---|---|---|---|---|---|---|---|
| Rv3120 | - | 200 | 0 | 0 | 0 | 0 | 0 | 0 | 0 | 0 | 0 | 0 | 0 | 0 | 0 | 0 | 0 | 0 | 67 | 0 | 0 | 0 | 0 | 51 | 100 | 100 | 100 | 0 | 0 |
| Rv3121 | *cyp141* | 400 | 0 | 0 | 44 | 45 | 41 | 0 | 0 | 46 | 46 | 51 | 51 | 51 | 42 | 49 | 49 | 49 | 98 | 50 | 49 | 52 | 52 | 51 | 100 | 100 | 100 | 46 | 51 |
| Rv3122 | - | 156 | 0 | 0 | 0 | 0 | 0 | 0 | 0 | 0 | 0 | 0 | 0 | 0 | 0 | 0 | 0 | 0 | 100 | 0 | 0 | 0 | 0 | 0 | 100 | 100 | 100 | 0 | 0 |
| Rv3123 | - | 164 | 0 | 0 | 0 | 0 | 0 | 0 | 0 | 0 | 0 | 0 | 0 | 0 | 0 | 0 | 0 | 0 | 100 | 0 | 0 | 0 | 0 | 0 | 100 | 100 | 100 | 0 | 0 |
| Rv3124 | - | 289 | 0 | 0 | 0 | 0 | 0 | 0 | 0 | 43 | 57 | 47 | 66 | 47 | 43 | 69 | 73 | 69 | 100 | 48 | 48 | 49 | 49 | 50 | 100 | 100 | 100 | 73 | 50 |
| Rv3125c | *PPE49* | 391 | 0 | 0 | 0 | 0 | 0 | 0 | 0 | 0 | 40 | 41 | 36 | 37 | 55 | 56 | 58 | 58 | 99 | 41 | 42 | 43 | 43 | 43 | 100 | 100 | 100 | 74 | 54 |
| Rv3126c | - | 104 | 0 | 0 | 0 | 0 | 0 | 0 | 0 | 0 | 0 | 0 | 0 | 0 | 0 | 0 | 0 | 0 | 100 | 0 | 0 | 0 | 0 | 0 | 100 | 100 | 100 | 0 | 0 |
| Rv3127 | - | 344 | 0 | 0 | 0 | 0 | 0 | 0 | 0 | 46 | 55 | 52 | 56 | 56 | 66 | 65 | 80 | 65 | 99 | 63 | 66 | 66 | 66 | 64 | 100 | 100 | 100 | 80 | 66 |
| Rv3129 | - | 110 | 0 | 58 | 56 | 58 | 0 | 67 | 64 | 0 | 0 | 0 | 0 | 0 | 68 | 77 | 80 | 77 | 100 | 68 | 73 | 73 | 73 | 76 | 100 | 100 | 100 | 80 | 66 |
| Rv3130c | *tgs1* | 463 | 0 | 0 | 0 | 0 | 0 | 0 | 0 | 59 | 50 | 61 | 58 | 49 | 49 | 58 | 78 | 59 | 100 | 69 | 71 | 71 | 71 | 70 | 99 | 100 | 100 | 78 | 68 |
| Rv3131 | - | 332 | 0 | 0 | 0 | 0 | 0 | 0 | 0 | 51 | 63 | 48 | 49 | 49 | 48 | 76 | 75 | 75 | 100 | 75 | 76 | 76 | 76 | 77 | 100 | 100 | 100 | 82 | 77 |
| Rv3132c | *devS* | 578 | 45 | 0 | 0 | 0 | 46 | 0 | 0 | 58 | 70 | 67 | 73 | 72 | 66 | 62 | 89 | 62 | 99 | 76 | 76 | 76 | 76 | 78 | 100 | 100 | 100 | 89 | 76 |
| Rv3133c | *devR* | 217 | 59 | 53 | 60 | 57 | 55 | 53 | 50 | 75 | 85 | 86 | 86 | 87 | 85 | 93 | 95 | 93 | 100 | 92 | 92 | 92 | 92 | 94 | 100 | 100 | 100 | 95 | 94 |
| Rv3134c | - | 268 | 45 | 41 | 41 | 41 | 42 | 47 | 44 | 45 | 41 | 42 | 44 | 42 | 52 | 66 | 79 | 66 | 100 | 53 | 55 | 55 | 55 | 52 | 100 | 100 | 100 | 78 | 55 |
| Rv3135 | *PPE50* | 132 | 0 | 0 | 0 | 0 | 0 | 0 | 0 | 0 | 0 | 0 | 0 | 0 | 59 | 72 | 74 | 72 | 90 | 57 | 61 | 61 | 61 | 57 | 71 | 100 | 100 | 74 | 59 |
| Rv3136 | *PPE51* | 380 | 0 | 0 | 0 | 0 | 0 | 0 | 0 | 0 | 0 | 39 | 0 | 0 | 55 | 63 | 80 | 63 | 100 | 43 | 52 | 52 | 52 | 49 | 100 | 100 | 100 | 80 | 49 |
| Rv3137 | - | 260 | 75 | 76 | 77 | 79 | 70 | 70 | 75 | 71 | 81 | 80 | 82 | 82 | 81 | 86 | 88 | 86 | 99 | 76 | 83 | 83 | 83 | 81 | 99 | 100 | 99 | 88 | 77 |
| Rv3138 | *pflA* | 362 | 0 | 0 | 0 | 0 | 0 | 0 | 0 | 0 | 0 | 0 | 0 | 0 | 0 | 0 | 0 | 0 | 99 | 0 | 0 | 0 | 0 | 0 | 100 | 100 | 100 | 0 | 0 |
| Rv3139 | *fadE24* | 468 | 43 | 0 | 45 | 41 | 66 | 65 | 67 | 67 | 76 | 77 | 77 | 77 | 80 | 93 | 93 | 93 | 100 | 90 | 86 | 88 | 88 | 88 | 100 | 100 | 100 | 92 | 89 |
| Rv3140 | *fadE23* | 401 | 44 | 0 | 46 | 44 | 81 | 80 | 81 | 78 | 83 | 83 | 82 | 82 | 87 | 94 | 94 | 94 | 100 | 89 | 88 | 88 | 88 | 90 | 100 | 100 | 100 | 94 | 90 |
| Rv3141 | *fadB4* | 323 | 49 | 45 | 49 | 48 | 48 | 43 | 44 | 67 | 82 | 66 | 73 | 73 | 62 | 93 | 92 | 94 | 100 | 85 | 85 | 86 | 86 | 86 | 100 | 100 | 100 | 92 | 84 |
| Rv3142c | - | 142 | 0 | 0 | 0 | 0 | 0 | 0 | 0 | 0 | 0 | 0 | 0 | 0 | 72 | 0 | 67 | 69 | 99 | 67 | 67 | 71 | 71 | 0 | 100 | 100 | 100 | 0 | 67 |
| Rv3143 | - | 133 | 0 | 0 | 0 | 0 | 0 | 0 | 0 | 86 | 84 | 82 | 86 | 87 | 74 | 93 | 94 | 93 | 100 | 84 | 86 | 86 | 86 | 83 | 100 | 100 | 100 | 94 | 85 |
| Rv3144c | *PPE52* | 409 | 0 | 0 | 0 | 0 | 36 | 0 | 36 | 0 | 39 | 37 | 36 | 37 | 48 | 49 | 64 | 48 | 99 | 36 | 38 | 38 | 38 | 48 | 99 | 99 | 99 | 66 | 39 |
| Rv3145 | *nuoA* | 128 | 0 | 0 | 0 | 0 | 0 | 0 | 0 | 71 | 81 | 76 | 76 | 77 | 74 | 87 | 89 | 87 | 100 | 80 | 86 | 86 | 86 | 78 | 100 | 100 | 100 | 89 | 81 |
| Rv3146 | *nuoB* | 184 | 0 | 0 | 0 | 0 | 0 | 0 | 0 | 85 | 89 | 90 | 91 | 91 | 92 | 98 | 100 | 98 | 100 | 96 | 95 | 95 | 95 | 95 | 100 | 100 | 100 | 100 | 96 |
| Rv3147 | *nuoC* | 236 | 0 | 0 | 0 | 0 | 0 | 0 | 0 | 77 | 80 | 83 | 83 | 85 | 83 | 86 | 92 | 86 | 100 | 86 | 86 | 86 | 86 | 85 | 100 | 100 | 100 | 92 | 86 |
| Rv3148 | *nuoD* | 440 | 0 | 0 | 0 | 0 | 0 | 0 | 0 | 78 | 87 | 88 | 87 | 87 | 89 | 95 | 96 | 95 | 100 | 87 | 90 | 90 | 90 | 90 | 100 | 100 | 100 | 96 | 89 |
| Rv3149 | *nuoE* | 252 | 0 | 0 | 0 | 0 | 0 | 0 | 0 | 69 | 81 | 72 | 77 | 76 | 73 | 91 | 93 | 91 | 100 | 75 | 75 | 75 | 75 | 77 | 100 | 100 | 100 | 93 | 75 |
| Rv3150 | *nuoF* | 445 | 0 | 0 | 0 | 0 | 0 | 0 | 0 | 82 | 87 | 88 | 90 | 89 | 86 | 94 | 95 | 94 | 100 | 89 | 90 | 90 | 90 | 90 | 100 | 100 | 100 | 95 | 88 |
| Rv3151 | *nuoG* | 806 | 0 | 0 | 0 | 0 | 0 | 0 | 0 | 70 | 75 | 78 | 81 | 81 | 75 | 89 | 91 | 89 | 99 | 79 | 78 | 78 | 78 | 80 | 99 | 100 | 99 | 91 | 80 |
| Rv3152 | *nuoH* | 410 | 0 | 0 | 0 | 0 | 0 | 0 | 0 | 72 | 76 | 81 | 80 | 80 | 78 | 87 | 89 | 85 | 100 | 80 | 80 | 80 | 80 | 81 | 100 | 100 | 100 | 89 | 80 |
| Rv3153 | *nuoI* | 211 | 0 | 0 | 0 | 0 | 0 | 0 | 0 | 78 | 85 | 80 | 74 | 77 | 80 | 82 | 91 | 82 | 99 | 81 | 87 | 87 | 87 | 84 | 100 | 100 | 100 | 91 | 89 |
| Rv3154 | *nuoJ* | 262 | 0 | 0 | 0 | 0 | 0 | 0 | 0 | 73 | 75 | 74 | 78 | 76 | 79 | 88 | 93 | 88 | 100 | 84 | 85 | 85 | 85 | 85 | 100 | 100 | 100 | 93 | 86 |
| Rv3155 | *nuoK* | 99 | 0 | 0 | 0 | 0 | 0 | 0 | 0 | 84 | 92 | 92 | 93 | 93 | 92 | 96 | 97 | 96 | 100 | 94 | 91 | 91 | 91 | 92 | 100 | 100 | 100 | 97 | 92 |
| Rv3156 | *nuoL* | 633 | 50 | 51 | 48 | 49 | 49 | 49 | 49 | 72 | 81 | 81 | 80 | 82 | 80 | 91 | 93 | 91 | 100 | 81 | 86 | 83 | 83 | 86 | 100 | 100 | 100 | 92 | 83 |
| Rv3157 | *nuoM* | 553 | 44 | 44 | 45 | 45 | 43 | 44 | 42 | 67 | 77 | 76 | 79 | 80 | 78 | 88 | 89 | 88 | 99 | 80 | 81 | 81 | 81 | 82 | 100 | 100 | 100 | 89 | 80 |
| Rv3158 | *nuoN* | 531 | 44 | 45 | 47 | 47 | 44 | 46 | 45 | 65 | 75 | 78 | 78 | 78 | 73 | 87 | 88 | 88 | 100 | 77 | 78 | 78 | 78 | 78 | 99 | 100 | 100 | 89 | 78 |

| | | | | | | | | | | | | | | | | | | | | | | | | | | | | | |
|---|---|---|---|---|---|---|---|---|---|---|---|---|---|---|---|---|---|---|---|---|---|---|---|---|---|---|---|---|---|
| Rv3159c | *PPE53* | 590 | 0 | 0 | 0 | 0 | 0 | 46 | 0 | 0 | 41 | 38 | 37 | 39 | 43 | 65 | 74 | 65 | 99 | 33 | 38 | 44 | 44 | 38 | 99 | 100 | 100 | 73 | 33 |
| Rv3160c | - | 213 | 0 | 0 | 0 | 0 | 0 | 0 | 0 | 0 | 50 | 42 | 46 | 45 | 56 | 0 | 0 | 0 | 100 | 46 | 48 | 48 | 48 | 46 | 100 | 100 | 100 | 0 | 0 |
| Rv3161c | - | 382 | 0 | 0 | 47 | 46 | 0 | 0 | 0 | 44 | 52 | 0 | 52 | 46 | 45 | 46 | 48 | 46 | 100 | 51 | 48 | 48 | 48 | 48 | 100 | 100 | 100 | 46 | 48 |
| Rv3162c | - | 145 | 0 | 0 | 0 | 0 | 0 | 0 | 0 | 0 | 53 | 0 | 0 | 0 | 55 | 69 | 73 | 66 | 86 | 57 | 61 | 62 | 62 | 56 | 100 | 100 | 100 | 72 | 63 |
| Rv3163c | - | 423 | 0 | 0 | 0 | 0 | 0 | 0 | 0 | 40 | 67 | 0 | 65 | 63 | 65 | 89 | 87 | 86 | 100 | 77 | 73 | 73 | 73 | 74 | 100 | 100 | 100 | 86 | 76 |
| Rv3164c | *moxR3* | 320 | 0 | 47 | 44 | 46 | 0 | 0 | 0 | 74 | 85 | 61 | 86 | 86 | 86 | 96 | 96 | 96 | 99 | 90 | 89 | 89 | 89 | 91 | 100 | 100 | 100 | 96 | 89 |
| Rv3165c | - | 160 | 0 | 0 | 0 | 0 | 0 | 0 | 0 | 0 | 56 | 0 | 59 | 60 | 58 | 85 | 83 | 85 | 100 | 72 | 71 | 69 | 69 | 63 | 100 | 100 | 100 | 84 | 74 |
| Rv3166c | - | 319 | 0 | 0 | 0 | 0 | 0 | 0 | 0 | 0 | 51 | 0 | 51 | 48 | 48 | 72 | 72 | 72 | 100 | 55 | 60 | 61 | 61 | 52 | 100 | 100 | 100 | 71 | 54 |
| Rv3167c | - | 208 | 0 | 0 | 0 | 0 | 0 | 0 | 0 | 44 | 57 | 53 | 59 | 59 | 50 | 92 | 90 | 92 | 100 | 83 | 86 | 86 | 86 | 82 | 100 | 100 | 100 | 90 | 82 |
| Rv3168 | - | 378 | 0 | 0 | 0 | 0 | 0 | 0 | 0 | 76 | 57 | 60 | 58 | 57 | 60 | 90 | 90 | 90 | 99 | 77 | 80 | 81 | 81 | 82 | 100 | 100 | 100 | 89 | 80 |
| Rv3169 | - | 374 | 0 | 0 | 0 | 0 | 0 | 0 | 0 | 81 | 70 | 57 | 57 | 57 | 53 | 94 | 95 | 94 | 100 | 90 | 87 | 88 | 88 | 92 | 100 | 100 | 100 | 95 | 91 |
| Rv3170 | *aofH* | 448 | 0 | 0 | 0 | 0 | 0 | 0 | 0 | 56 | 59 | 51 | 54 | 55 | 63 | 89 | 89 | 89 | 100 | 78 | 77 | 77 | 77 | 77 | 100 | 100 | 100 | 88 | 76 |
| Rv3171c | *hpx* | 299 | 0 | 43 | 39 | 0 | 0 | 0 | 37 | 42 | 45 | 54 | 53 | 53 | 44 | 81 | 85 | 81 | 99 | 72 | 73 | 73 | 73 | 69 | 100 | 100 | 100 | 85 | 75 |
| Rv3172c | - | 160 | 0 | 0 | 0 | 0 | 0 | 0 | 0 | 0 | 0 | 0 | 0 | 0 | 0 | 0 | 68 | 0 | 100 | 0 | 0 | 0 | 0 | 0 | 100 | 100 | 100 | 78 | 0 |
| Rv3173c | - | 200 | 41 | 46 | 45 | 43 | 0 | 43 | 43 | 0 | 45 | 53 | 55 | 0 | 0 | 48 | 77 | 77 | 100 | 0 | 67 | 67 | 67 | 70 | 100 | 100 | 100 | 0 | 50 |
| Rv3174 | - | 235 | 0 | 46 | 47 | 0 | 50 | 50 | 50 | 48 | 65 | 65 | 67 | 69 | 58 | 51 | 51 | 51 | 99 | 53 | 66 | 65 | 65 | 67 | 100 | 100 | 99 | 51 | 65 |
| Rv3175 | - | 495 | 41 | 41 | 44 | 41 | 43 | 41 | 42 | 51 | 41 | 43 | 47 | 48 | 42 | 44 | 45 | 44 | 100 | 41 | 43 | 43 | 43 | 50 | 100 | 100 | 100 | 46 | 41 |
| Rv3176c | *mesT* | 318 | 0 | 0 | 0 | 0 | 0 | 0 | 0 | 0 | 0 | 0 | 0 | 0 | 42 | 0 | 43 | 0 | 100 | 0 | 0 | 0 | 0 | 41 | 100 | 100 | 100 | 43 | 0 |
| Rv3177 | - | 286 | 0 | 0 | 0 | 0 | 0 | 0 | 0 | 40 | 41 | 66 | 39 | 39 | 0 | 41 | 41 | 41 | 100 | 36 | 42 | 42 | 42 | 54 | 100 | 100 | 100 | 41 | 41 |
| Rv3178 | - | 119 | 0 | 0 | 0 | 0 | 0 | 0 | 0 | 49 | 50 | 64 | 66 | 66 | 63 | 60 | 60 | 60 | 100 | 58 | 82 | 82 | 82 | 54 | 100 | 100 | 100 | 57 | 58 |
| Rv3179 | - | 429 | 53 | 43 | 0 | 0 | 0 | 0 | 0 | 43 | 54 | 0 | 0 | 0 | 0 | 0 | 0 | 0 | 100 | 0 | 0 | 0 | 0 | 0 | 100 | 100 | 100 | 0 | 0 |
| Rv3180c | - | 144 | 0 | 0 | 0 | 0 | 0 | 0 | 0 | 0 | 0 | 0 | 0 | 0 | 0 | 0 | 97 | 0 | 100 | 99 | 0 | 0 | 0 | 0 | 100 | 100 | 100 | 90 | 0 |
| Rv3181c | - | 150 | 0 | 0 | 0 | 0 | 0 | 0 | 0 | 0 | 0 | 0 | 0 | 0 | 0 | 0 | 92 | 0 | 100 | 82 | 0 | 0 | 0 | 0 | 100 | 100 | 100 | 90 | 0 |
| Rv3182 | - | 114 | 0 | 0 | 0 | 0 | 0 | 0 | 0 | 0 | 0 | 0 | 0 | 0 | 0 | 63 | 0 | 0 | 100 | 0 | 0 | 0 | 0 | 0 | 100 | 100 | 100 | 0 | 0 |
| Rv3183 | - | 109 | 0 | 0 | 0 | 0 | 0 | 0 | 0 | 0 | 0 | 0 | 0 | 0 | 0 | 0 | 0 | 0 | 100 | 0 | 0 | 0 | 0 | 0 | 100 | 100 | 100 | 0 | 0 |
| Rv3184 | - | 108 | 65 | 0 | 66 | 0 | 61 | 0 | 60 | 76 | 0 | 0 | 0 | 0 | 59 | 63 | 82 | 66 | 100 | 82 | 96 | 77 | 77 | 84 | 100 | 100 | 100 | 0 | 62 |
| Rv3185 | - | 312 | 58 | 49 | 61 | 51 | 63 | 0 | 61 | 56 | 0 | 0 | 51 | 52 | 66 | 59 | 66 | 66 | 100 | 66 | 90 | 67 | 67 | 66 | 100 | 100 | 100 | 0 | 65 |
| Rv3186 | - | 108 | 65 | 0 | 66 | 0 | 61 | 0 | 60 | 76 | 0 | 0 | 0 | 0 | 59 | 63 | 82 | 66 | 100 | 82 | 96 | 77 | 77 | 84 | 100 | 100 | 100 | 0 | 62 |
| Rv3187 | - | 312 | 58 | 49 | 61 | 51 | 63 | 0 | 61 | 56 | 0 | 0 | 51 | 52 | 66 | 59 | 66 | 66 | 100 | 66 | 90 | 67 | 67 | 66 | 100 | 100 | 100 | 0 | 65 |
| Rv3188 | - | 115 | 0 | 0 | 0 | 0 | 0 | 0 | 0 | 0 | 0 | 0 | 0 | 0 | 0 | 0 | 0 | 0 | 100 | 0 | 0 | 0 | 0 | 0 | 100 | 100 | 100 | 0 | 0 |
| Rv3189 | - | 206 | 0 | 0 | 0 | 0 | 0 | 0 | 0 | 0 | 0 | 0 | 0 | 0 | 0 | 0 | 0 | 0 | 100 | 0 | 0 | 0 | 0 | 0 | 100 | 100 | 100 | 0 | 0 |
| Rv3190c | - | 421 | 0 | 0 | 0 | 0 | 0 | 0 | 0 | 0 | 0 | 0 | 0 | 0 | 0 | 0 | 0 | 0 | 99 | 0 | 0 | 0 | 0 | 0 | 99 | 100 | 99 | 0 | 0 |
| Rv3191c | - | 344 | 0 | 53 | 0 | 59 | 55 | 0 | 61 | 53 | 0 | 0 | 59 | 0 | 0 | 57 | 73 | 0 | 100 | 58 | 62 | 62 | 62 | 52 | 100 | 100 | 100 | 88 | 58 |
| Rv3192 | - | 153 | 0 | 0 | 0 | 0 | 0 | 0 | 0 | 0 | 53 | 56 | 0 | 0 | 55 | 75 | 76 | 76 | 100 | 71 | 72 | 72 | 72 | 57 | 100 | 100 | 100 | 76 | 72 |
| Rv3193c | - | 992 | 62 | 63 | 63 | 63 | 62 | 62 | 61 | 55 | 69 | 70 | 68 | 69 | 82 | 94 | 94 | 94 | 100 | 87 | 88 | 88 | 88 | 87 | 100 | 100 | 100 | 94 | 88 |
| Rv3194c | - | 340 | 61 | 58 | 62 | 61 | 60 | 60 | 57 | 51 | 66 | 67 | 68 | 67 | 80 | 88 | 91 | 88 | 100 | 80 | 81 | 81 | 81 | 80 | 100 | 100 | 100 | 91 | 80 |
| Rv3195 | - | 472 | 59 | 62 | 59 | 61 | 52 | 55 | 52 | 59 | 73 | 75 | 75 | 73 | 82 | 87 | 90 | 87 | 100 | 85 | 86 | 85 | 85 | 84 | 100 | 100 | 99 | 90 | 84 |
| Rv3196 | - | 299 | 37 | 39 | 41 | 43 | 42 | 0 | 39 | 39 | 56 | 55 | 57 | 58 | 60 | 82 | 82 | 81 | 100 | 72 | 71 | 71 | 71 | 71 | 100 | 100 | 100 | 82 | 71 |

| | | | | | | | | | | | | | | | | | | | | | | | | | | | | | |
|---|---|---|---|---|---|---|---|---|---|---|---|---|---|---|---|---|---|---|---|---|---|---|---|---|---|---|---|---|---|
| Rv3196A | - | 66 | 0 | 0 | 0 | 0 | 0 | 0 | 0 | 0 | 0 | 0 | 0 | 0 | 0 | 0 | 0 | 0 | 100 | 0 | 0 | 0 | 0 | 0 | 100 | 100 | 100 | 90 | 0 |
| Rv3197 | - | 447 | 0 | 0 | 0 | 0 | 0 | 0 | 0 | 61 | 73 | 72 | 72 | 71 | 75 | 94 | 95 | 94 | 100 | 88 | 89 | 89 | 89 | 88 | 100 | 100 | 100 | 95 | 89 |
| Rv3197A | *whiB7* | 92 | 0 | 0 | 0 | 0 | 0 | 0 | 0 | 77 | 77 | 79 | 81 | 80 | 84 | 77 | 85 | 78 | 100 | 80 | 89 | 89 | 89 | 77 | 100 | 100 | 100 | 84 | 83 |
| Rv3198A | - | 84 | 58 | 68 | 61 | 62 | 0 | 63 | 0 | 68 | 74 | 80 | 77 | 77 | 78 | 83 | 83 | 84 | 100 | 81 | 82 | 82 | 82 | 81 | 100 | 100 | 100 | 84 | 79 |
| Rv3198c | *uvrD2* | 700 | 69 | 71 | 73 | 72 | 68 | 67 | 66 | 61 | 74 | 77 | 77 | 77 | 83 | 89 | 92 | 89 | 100 | 85 | 87 | 87 | 87 | 86 | 100 | 100 | 100 | 91 | 85 |
| Rv3199c | *nudC* | 313 | 49 | 42 | 50 | 48 | 0 | 0 | 0 | 49 | 70 | 71 | 72 | 72 | 79 | 92 | 89 | 91 | 99 | 82 | 83 | 83 | 83 | 86 | 100 | 100 | 100 | 89 | 84 |
| Rv3200c | - | 355 | 75 | 80 | 77 | 78 | 78 | 77 | 76 | 67 | 83 | 84 | 84 | 84 | 81 | 85 | 92 | 85 | 100 | 80 | 81 | 81 | 81 | 81 | 100 | 100 | 100 | 92 | 80 |
| Rv3201c | - | 1101 | 55 | 56 | 56 | 55 | 50 | 47 | 50 | 50 | 61 | 66 | 67 | 67 | 70 | 83 | 85 | 83 | 100 | 76 | 77 | 77 | 77 | 77 | 100 | 100 | 100 | 84 | 76 |
| Rv3202c | - | 1055 | 47 | 47 | 49 | 49 | 46 | 45 | 44 | 47 | 59 | 62 | 61 | 62 | 63 | 83 | 82 | 82 | 99 | 74 | 75 | 75 | 75 | 73 | 100 | 100 | 100 | 83 | 75 |
| Rv3203 | *lipV* | 224 | 0 | 0 | 0 | 0 | 0 | 0 | 0 | 0 | 65 | 67 | 67 | 67 | 74 | 79 | 88 | 79 | 100 | 75 | 77 | 77 | 77 | 74 | 100 | 100 | 100 | 88 | 73 |
| Rv3204 | - | 101 | 0 | 0 | 0 | 0 | 0 | 0 | 0 | 0 | 76 | 73 | 76 | 76 | 88 | 88 | 90 | 89 | 99 | 83 | 86 | 87 | 87 | 85 | 99 | 100 | 99 | 90 | 85 |
| Rv3205c | - | 292 | 47 | 54 | 51 | 51 | 63 | 57 | 60 | 0 | 83 | 80 | 81 | 80 | 89 | 92 | 90 | 92 | 100 | 88 | 90 | 90 | 90 | 91 | 100 | 100 | 100 | 90 | 89 |
| Rv3206c | *moeB1* | 392 | 54 | 55 | 62 | 58 | 60 | 54 | 56 | 73 | 87 | 84 | 88 | 87 | 93 | 87 | 94 | 87 | 99 | 91 | 92 | 92 | 92 | 91 | 100 | 100 | 100 | 94 | 89 |
| Rv3207c | - | 285 | 56 | 59 | 59 | 60 | 57 | 57 | 55 | 0 | 68 | 73 | 72 | 70 | 77 | 90 | 92 | 90 | 100 | 79 | 81 | 81 | 81 | 79 | 100 | 100 | 100 | 92 | 80 |
| Rv3208 | - | 228 | 49 | 50 | 50 | 48 | 46 | 48 | 0 | 59 | 76 | 81 | 82 | 82 | 78 | 92 | 90 | 92 | 100 | 85 | 87 | 87 | 87 | 86 | 100 | 100 | 100 | 90 | 86 |
| Rv3208A | *TB9.4* | 90 | 62 | 70 | 69 | 70 | 0 | 62 | 0 | 63 | 80 | 75 | 72 | 80 | 86 | 82 | 91 | 82 | 100 | 90 | 89 | 89 | 89 | 85 | 100 | 100 | 100 | 91 | 89 |
| Rv3209 | - | 186 | 0 | 0 | 0 | 0 | 0 | 0 | 0 | 0 | 0 | 0 | 0 | 0 | 48 | 68 | 68 | 68 | 100 | 56 | 59 | 59 | 59 | 58 | 100 | 100 | 100 | 76 | 58 |
| Rv3210c | - | 231 | 0 | 0 | 0 | 0 | 0 | 0 | 0 | 59 | 73 | 75 | 72 | 72 | 78 | 93 | 94 | 94 | 100 | 88 | 86 | 86 | 86 | 85 | 100 | 100 | 100 | 94 | 85 |
| Rv3211 | *rhlE* | 527 | 69 | 68 | 71 | 71 | 68 | 65 | 63 | 63 | 74 | 73 | 74 | 76 | 77 | 80 | 85 | 81 | 99 | 78 | 78 | 79 | 79 | 79 | 100 | 100 | 100 | 84 | 79 |
| Rv3212 | - | 407 | 45 | 43 | 45 | 46 | 39 | 48 | 47 | 0 | 54 | 56 | 58 | 59 | 63 | 83 | 87 | 83 | 99 | 72 | 74 | 74 | 74 | 73 | 100 | 100 | 100 | 87 | 75 |
| Rv3213c | - | 266 | 61 | 63 | 61 | 61 | 62 | 60 | 63 | 75 | 62 | 83 | 85 | 84 | 91 | 95 | 95 | 94 | 100 | 90 | 92 | 92 | 92 | 93 | 100 | 100 | 100 | 95 | 92 |
| Rv3214 | *gpm2* | 203 | 52 | 49 | 52 | 48 | 49 | 50 | 53 | 50 | 65 | 64 | 67 | 67 | 77 | 85 | 85 | 85 | 100 | 76 | 78 | 78 | 78 | 80 | 100 | 100 | 100 | 86 | 76 |
| Rv3215 | *entC* | 372 | 60 | 59 | 60 | 58 | 54 | 57 | 54 | 50 | 62 | 61 | 66 | 65 | 67 | 84 | 87 | 84 | 100 | 71 | 72 | 72 | 72 | 74 | 100 | 100 | 100 | 87 | 73 |
| Rv3216 | - | 110 | 0 | 0 | 0 | 0 | 0 | 0 | 57 | 0 | 74 | 69 | 72 | 71 | 68 | 85 | 85 | 85 | 100 | 85 | 83 | 84 | 84 | 84 | 100 | 100 | 100 | 89 | 84 |
| Rv3217c | - | 143 | 0 | 53 | 45 | 49 | 0 | 57 | 53 | 0 | 71 | 62 | 65 | 70 | 67 | 79 | 78 | 79 | 100 | 57 | 68 | 69 | 69 | 70 | 96 | 100 | 99 | 78 | 61 |
| Rv3218 | - | 321 | 50 | 51 | 49 | 48 | 0 | 0 | 0 | 0 | 63 | 70 | 68 | 68 | 73 | 88 | 83 | 88 | 100 | 78 | 77 | 77 | 77 | 79 | 100 | 100 | 100 | 83 | 79 |
| Rv3219 | *whiB1* | 84 | 90 | 92 | 90 | 90 | 91 | 91 | 92 | 82 | 94 | 95 | 96 | 96 | 96 | 98 | 98 | 98 | 100 | 96 | 98 | 98 | 98 | 98 | 100 | 100 | 100 | 98 | 97 |
| Rv3220c | - | 501 | 0 | 0 | 0 | 0 | 0 | 0 | 0 | 60 | 74 | 73 | 74 | 74 | 80 | 89 | 89 | 89 | 100 | 84 | 85 | 85 | 85 | 83 | 100 | 100 | 100 | 90 | 84 |
| Rv3221A | - | 101 | 0 | 0 | 0 | 0 | 0 | 0 | 0 | 0 | 73 | 71 | 70 | 64 | 88 | 87 | 88 | 95 | 100 | 85 | 85 | 85 | 85 | 88 | 100 | 100 | 100 | 89 | 87 |
| Rv3221c | *TB7.3* | 71 | 67 | 62 | 0 | 0 | 0 | 0 | 0 | 76 | 78 | 84 | 85 | 85 | 88 | 94 | 95 | 94 | 100 | 95 | 91 | 91 | 91 | 91 | 100 | 100 | 100 | 95 | 94 |
| Rv3222c | - | 183 | 0 | 0 | 0 | 0 | 0 | 0 | 0 | 44 | 0 | 0 | 0 | 0 | 0 | 0 | 0 | 0 | 100 | 0 | 0 | 0 | 0 | 0 | 100 | 100 | 100 | 0 | 0 |
| Rv3223c | *sigH* | 216 | 76 | 78 | 82 | 83 | 81 | 81 | 77 | 78 | 86 | 89 | 93 | 93 | 91 | 97 | 94 | 97 | 100 | 88 | 90 | 90 | 90 | 90 | 100 | 100 | 100 | 94 | 89 |
| Rv3224 | - | 282 | 40 | 0 | 42 | 45 | 44 | 47 | 45 | 48 | 71 | 69 | 46 | 69 | 74 | 94 | 96 | 94 | 100 | 93 | 92 | 92 | 92 | 92 | 100 | 100 | 100 | 96 | 93 |
| Rv3224A | - | 62 | 0 | 0 | 0 | 0 | 0 | 0 | 0 | 0 | 0 | 0 | 0 | 0 | 0 | 0 | 0 | 0 | 100 | 0 | 0 | 0 | 0 | 0 | 100 | 100 | 100 | 0 | 0 |
| Rv3224B | - | 72 | 0 | 0 | 0 | 0 | 0 | 0 | 0 | 0 | 71 | 71 | 67 | 69 | 88 | 0 | 93 | 0 | 100 | 69 | 89 | 89 | 89 | 84 | 100 | 100 | 100 | 93 | 66 |
| Rv3225c | - | 474 | 0 | 0 | 0 | 0 | 0 | 0 | 0 | 0 | 54 | 59 | 0 | 51 | 43 | 0 | 71 | 0 | 99 | 0 | 0 | 0 | 0 | 0 | 100 | 100 | 100 | 76 | 0 |
| Rv3226c | - | 252 | 51 | 48 | 51 | 51 | 41 | 0 | 39 | 53 | 69 | 66 | 63 | 62 | 68 | 83 | 83 | 83 | 100 | 70 | 73 | 71 | 71 | 73 | 100 | 100 | 100 | 80 | 73 |
| Rv3227 | *aroA* | 450 | 65 | 66 | 66 | 67 | 66 | 60 | 63 | 65 | 68 | 69 | 69 | 69 | 75 | 89 | 88 | 89 | 99 | 80 | 78 | 78 | 78 | 78 | 100 | 100 | 100 | 87 | 79 |

| | | | | | | | | | | | | | | | | | | | | | | | | | | | | | |
|---|---|---|---|---|---|---|---|---|---|---|---|---|---|---|---|---|---|---|---|---|---|---|---|---|---|---|---|---|---|
| Rv3228 | - | 330 | 75 | 72 | 74 | 74 | 75 | 69 | 76 | 64 | 82 | 81 | 82 | 82 | 83 | 93 | 91 | 93 | 100 | 86 | 86 | 86 | 86 | 87 | 100 | 100 | 100 | 90 | 87 |
| Rv3229c | - | 427 | 0 | 66 | 0 | 0 | 0 | 0 | 0 | 73 | 78 | 78 | 80 | 79 | 84 | 90 | 94 | 90 | 100 | 85 | 88 | 88 | 88 | 88 | 100 | 100 | 100 | 94 | 87 |
| Rv3230c | - | 380 | 0 | 65 | 40 | 0 | 0 | 0 | 0 | 70 | 75 | 77 | 78 | 77 | 79 | 91 | 91 | 91 | 99 | 82 | 86 | 86 | 86 | 86 | 100 | 100 | 100 | 90 | 84 |
| Rv3231c | - | 169 | 61 | 65 | 65 | 65 | 63 | 61 | 60 | 0 | 74 | 76 | 79 | 80 | 83 | 89 | 90 | 89 | 100 | 82 | 83 | 83 | 83 | 83 | 100 | 100 | 100 | 90 | 84 |
| Rv3232c | *pvdS* | 295 | 75 | 78 | 77 | 77 | 79 | 76 | 77 | 84 | 49 | 73 | 70 | 70 | 73 | 89 | 89 | 90 | 100 | 87 | 70 | 70 | 70 | 73 | 100 | 100 | 100 | 89 | 73 |
| Rv3233c | - | 196 | 0 | 0 | 0 | 0 | 0 | 0 | 0 | 65 | 72 | 73 | 73 | 73 | 76 | 91 | 91 | 91 | 100 | 89 | 90 | 90 | 90 | 87 | 100 | 100 | 100 | 90 | 90 |
| Rv3234c | - | 271 | 0 | 0 | 0 | 0 | 0 | 0 | 0 | 55 | 68 | 69 | 72 | 73 | 79 | 90 | 92 | 90 | 100 | 87 | 86 | 86 | 86 | 86 | 100 | 100 | 99 | 82 | 86 |
| Rv3235 | - | 213 | 0 | 0 | 0 | 0 | 0 | 0 | 0 | 0 | 49 | 45 | 50 | 52 | 54 | 63 | 66 | 62 | 100 | 54 | 59 | 59 | 59 | 50 | 100 | 100 | 100 | 55 | 56 |
| Rv3236c | - | 385 | 0 | 41 | 0 | 0 | 0 | 0 | 0 | 0 | 67 | 66 | 66 | 66 | 40 | 88 | 89 | 89 | 100 | 0 | 86 | 86 | 86 | 82 | 100 | 100 | 100 | 89 | 42 |
| Rv3237c | - | 160 | 0 | 0 | 0 | 0 | 0 | 0 | 0 | 0 | 60 | 61 | 62 | 61 | 0 | 93 | 88 | 93 | 100 | 0 | 91 | 91 | 91 | 88 | 100 | 100 | 100 | 88 | 0 |
| Rv3238c | - | 244 | 0 | 0 | 0 | 0 | 0 | 0 | 0 | 0 | 53 | 0 | 67 | 68 | 68 | 82 | 81 | 83 | 100 | 0 | 81 | 81 | 81 | 0 | 100 | 100 | 100 | 81 | 80 |
| Rv3239c | - | 1048 | 46 | 48 | 53 | 50 | 49 | 48 | 55 | 49 | 50 | 58 | 58 | 49 | 50 | 49 | 48 | 48 | 99 | 50 | 51 | 51 | 51 | 51 | 99 | 100 | 100 | 82 | 49 |
| Rv3240c | *secA1* | 949 | 78 | 82 | 74 | 80 | 82 | 76 | 76 | 76 | 84 | 84 | 84 | 84 | 86 | 93 | 94 | 91 | 100 | 87 | 88 | 88 | 88 | 89 | 100 | 100 | 100 | 93 | 86 |
| Rv3241c | - | 214 | 65 | 66 | 66 | 65 | 66 | 69 | 65 | 63 | 81 | 81 | 83 | 82 | 87 | 80 | 88 | 81 | 100 | 86 | 84 | 84 | 84 | 86 | 100 | 100 | 100 | 88 | 82 |
| Rv3242c | - | 213 | 48 | 61 | 56 | 57 | 50 | 52 | 47 | 51 | 62 | 59 | 61 | 61 | 71 | 79 | 79 | 79 | 100 | 75 | 74 | 73 | 73 | 72 | 100 | 100 | 100 | 83 | 76 |
| Rv3243c | - | 280 | 0 | 0 | 0 | 0 | 0 | 0 | 0 | 0 | 0 | 0 | 0 | 0 | 0 | 0 | 86 | 0 | 100 | 65 | 0 | 0 | 0 | 0 | 100 | 100 | 100 | 85 | 0 |
| Rv3244c | *lpqB* | 583 | 49 | 45 | 49 | 49 | 48 | 48 | 50 | 42 | 59 | 61 | 63 | 63 | 74 | 92 | 89 | 89 | 99 | 86 | 84 | 84 | 84 | 86 | 100 | 100 | 100 | 93 | 86 |
| Rv3245c | *mtrB* | 567 | 67 | 70 | 66 | 69 | 70 | 69 | 68 | 58 | 74 | 80 | 77 | 78 | 81 | 92 | 95 | 92 | 100 | 89 | 91 | 91 | 91 | 91 | 100 | 100 | 100 | 94 | 92 |
| Rv3246c | *mtrA* | 228 | 82 | 83 | 83 | 84 | 85 | 80 | 83 | 86 | 95 | 93 | 94 | 94 | 98 | 100 | 100 | 100 | 100 | 98 | 98 | 98 | 98 | 98 | 100 | 100 | 100 | 99 | 98 |
| Rv3247c | *tmk* | 214 | 60 | 59 | 64 | 64 | 61 | 57 | 60 | 0 | 68 | 68 | 70 | 70 | 72 | 84 | 85 | 84 | 100 | 73 | 75 | 75 | 75 | 72 | 100 | 100 | 100 | 85 | 74 |
| Rv3248c | *sahH* | 495 | 0 | 83 | 84 | 84 | 84 | 0 | 81 | 81 | 93 | 90 | 91 | 91 | 94 | 92 | 92 | 92 | 100 | 94 | 93 | 93 | 93 | 94 | 100 | 100 | 100 | 96 | 94 |
| Rv3249c | - | 211 | 0 | 0 | 0 | 0 | 0 | 0 | 0 | 45 | 71 | 75 | 75 | 75 | 83 | 92 | 96 | 92 | 100 | 84 | 83 | 83 | 83 | 87 | 99 | 100 | 100 | 95 | 86 |
| Rv3250c | *rubB* | 60 | 0 | 0 | 0 | 0 | 64 | 0 | 0 | 0 | 91 | 91 | 93 | 93 | 93 | 94 | 96 | 94 | 100 | 94 | 94 | 94 | 94 | 93 | 100 | 100 | 100 | 96 | 93 |
| Rv3251c | *rubA* | 55 | 0 | 0 | 0 | 0 | 79 | 0 | 0 | 66 | 85 | 88 | 88 | 86 | 83 | 80 | 89 | 80 | 100 | 85 | 83 | 81 | 81 | 88 | 100 | 100 | 100 | 89 | 88 |
| Rv3252c | *alkB* | 416 | 0 | 0 | 0 | 0 | 66 | 0 | 0 | 70 | 80 | 83 | 84 | 82 | 81 | 90 | 91 | 90 | 100 | 88 | 88 | 89 | 89 | 89 | 100 | 100 | 100 | 82 | 89 |
| Rv3253c | - | 495 | 40 | 39 | 39 | 39 | 39 | 43 | 41 | 67 | 75 | 78 | 78 | 78 | 81 | 91 | 91 | 91 | 100 | 84 | 86 | 86 | 86 | 82 | 100 | 100 | 100 | 91 | 85 |
| Rv3254 | - | 462 | 0 | 0 | 0 | 0 | 0 | 0 | 0 | 0 | 0 | 0 | 45 | 49 | 47 | 81 | 86 | 82 | 100 | 75 | 79 | 79 | 79 | 73 | 100 | 100 | 99 | 85 | 58 |
| Rv3255c | *manA* | 408 | 59 | 62 | 60 | 62 | 57 | 58 | 55 | 55 | 73 | 69 | 70 | 70 | 74 | 90 | 92 | 90 | 100 | 83 | 83 | 82 | 82 | 84 | 100 | 100 | 100 | 93 | 83 |
| Rv3256c | - | 346 | 45 | 0 | 48 | 45 | 0 | 42 | 0 | 40 | 59 | 54 | 59 | 59 | 64 | 79 | 79 | 79 | 100 | 70 | 72 | 72 | 72 | 74 | 100 | 100 | 100 | 78 | 70 |
| Rv3257c | *manB* | 465 | 77 | 76 | 76 | 76 | 76 | 77 | 77 | 72 | 81 | 82 | 80 | 80 | 83 | 91 | 91 | 90 | 99 | 85 | 86 | 86 | 86 | 87 | 100 | 100 | 100 | 90 | 86 |
| Rv3258c | - | 163 | 50 | 52 | 55 | 72 | 63 | 48 | 45 | 55 | 64 | 66 | 63 | 62 | 70 | 80 | 80 | 80 | 100 | 75 | 75 | 75 | 75 | 77 | 100 | 100 | 100 | 80 | 76 |
| Rv3259 | - | 139 | 68 | 68 | 66 | 65 | 66 | 69 | 69 | 53 | 80 | 83 | 82 | 82 | 88 | 97 | 97 | 97 | 100 | 95 | 96 | 96 | 96 | 92 | 100 | 100 | 100 | 97 | 94 |
| Rv3260c | *whiB2* | 89 | 91 | 91 | 92 | 92 | 89 | 91 | 93 | 95 | 98 | 98 | 98 | 98 | 91 | 91 | 91 | 91 | 100 | 84 | 94 | 94 | 94 | 93 | 100 | 100 | 100 | 89 | 84 |
| Rv3261 | *fbiA* | 331 | 0 | 0 | 0 | 0 | 0 | 0 | 0 | 69 | 78 | 79 | 80 | 81 | 82 | 87 | 89 | 87 | 100 | 85 | 85 | 85 | 85 | 85 | 100 | 100 | 100 | 89 | 85 |
| Rv3262 | *fbiB* | 448 | 0 | 0 | 0 | 0 | 0 | 0 | 0 | 60 | 79 | 80 | 79 | 79 | 80 | 89 | 88 | 89 | 100 | 84 | 87 | 86 | 86 | 87 | 100 | 100 | 100 | 88 | 84 |
| Rv3263 | - | 553 | 0 | 0 | 0 | 0 | 0 | 0 | 0 | 0 | 0 | 0 | 0 | 0 | 0 | 80 | 83 | 80 | 100 | 48 | 0 | 0 | 0 | 45 | 100 | 100 | 99 | 82 | 49 |
| Rv3264c | *manB* | 359 | 76 | 79 | 78 | 80 | 79 | 79 | 80 | 40 | 86 | 86 | 87 | 88 | 86 | 92 | 93 | 92 | 100 | 89 | 89 | 89 | 89 | 89 | 100 | 100 | 100 | 93 | 89 |
| Rv3265c | *wbbL1* | 301 | 64 | 67 | 65 | 67 | 64 | 66 | 63 | 0 | 73 | 72 | 74 | 74 | 78 | 89 | 89 | 89 | 98 | 80 | 85 | 85 | 85 | 85 | 100 | 100 | 100 | 90 | 84 |

| | | | | | | | | | | | | | | | | | | | | | | | | | | | | | |
|---|---|---|---|---|---|---|---|---|---|---|---|---|---|---|---|---|---|---|---|---|---|---|---|---|---|---|---|---|---|
| Rv3266c | *rmlD* | 304 | 51 | 51 | 48 | 49 | 47 | 49 | 51 | 0 | 66 | 64 | 61 | 65 | 72 | 79 | 82 | 80 | 99 | 72 | 72 | 72 | 72 | 71 | 100 | 100 | 100 | 82 | 74 |
| Rv3267 | - | 498 | 57 | 63 | 60 | 61 | 67 | 58 | 59 | 43 | 68 | 68 | 66 | 66 | 74 | 91 | 90 | 91 | 100 | 81 | 82 | 82 | 82 | 81 | 100 | 100 | 100 | 90 | 80 |
| Rv3268 | - | 229 | 42 | 42 | 45 | 45 | 43 | 51 | 42 | 44 | 66 | 69 | 73 | 73 | 69 | 86 | 87 | 86 | 100 | 80 | 79 | 79 | 79 | 81 | 100 | 100 | 100 | 87 | 81 |
| Rv3269 | - | 93 | 0 | 0 | 0 | 0 | 0 | 0 | 0 | 0 | 0 | 0 | 0 | 72 | 0 | 87 | 84 | 87 | 100 | 0 | 0 | 0 | 0 | 77 | 100 | 100 | 100 | 84 | 0 |
| Rv3270 | *ctpC* | 718 | 52 | 50 | 58 | 58 | 50 | 55 | 51 | 58 | 50 | 49 | 50 | 85 | 52 | 94 | 95 | 94 | 100 | 49 | 49 | 49 | 49 | 86 | 100 | 100 | 100 | 95 | 49 |
| Rv3271c | - | 222 | 0 | 0 | 0 | 0 | 0 | 47 | 0 | 46 | 51 | 51 | 52 | 51 | 53 | 0 | 0 | 0 | 100 | 49 | 50 | 50 | 50 | 53 | 100 | 100 | 100 | 81 | 71 |
| Rv3272 | - | 394 | 44 | 0 | 47 | 0 | 52 | 0 | 44 | 48 | 46 | 79 | 49 | 52 | 51 | 84 | 89 | 85 | 100 | 47 | 52 | 46 | 46 | 52 | 100 | 100 | 100 | 89 | 45 |
| Rv3273 | - | 764 | 45 | 46 | 52 | 50 | 45 | 41 | 43 | 44 | 71 | 72 | 72 | 71 | 80 | 75 | 84 | 75 | 100 | 48 | 51 | 51 | 51 | 50 | 100 | 100 | 100 | 79 | 48 |
| Rv3274c | *fadE25* | 389 | 48 | 0 | 47 | 45 | 84 | 84 | 84 | 77 | 92 | 93 | 94 | 94 | 94 | 94 | 95 | 94 | 100 | 95 | 94 | 94 | 94 | 95 | 100 | 100 | 100 | 95 | 95 |
| Rv3275c | *purE* | 174 | 75 | 78 | 79 | 79 | 74 | 77 | 79 | 84 | 81 | 80 | 82 | 81 | 83 | 89 | 89 | 89 | 99 | 84 | 84 | 84 | 84 | 84 | 100 | 100 | 100 | 89 | 84 |
| Rv3276c | *purK* | 429 | 64 | 67 | 67 | 69 | 67 | 63 | 65 | 63 | 79 | 77 | 78 | 78 | 79 | 83 | 83 | 83 | 100 | 80 | 80 | 80 | 80 | 77 | 100 | 100 | 100 | 86 | 79 |
| Rv3277 | - | 272 | 0 | 0 | 0 | 0 | 0 | 0 | 0 | 57 | 75 | 80 | 76 | 74 | 74 | 92 | 91 | 92 | 100 | 84 | 84 | 84 | 84 | 85 | 100 | 100 | 100 | 91 | 77 |
| Rv3278c | - | 172 | 0 | 0 | 0 | 0 | 0 | 0 | 0 | 52 | 71 | 68 | 70 | 69 | 84 | 93 | 95 | 93 | 100 | 83 | 84 | 84 | 84 | 85 | 100 | 100 | 100 | 95 | 84 |
| Rv3279c | *birA* | 266 | 45 | 54 | 52 | 54 | 54 | 53 | 53 | 53 | 57 | 61 | 63 | 62 | 69 | 79 | 80 | 79 | 100 | 65 | 65 | 65 | 65 | 64 | 100 | 100 | 100 | 80 | 71 |
| Rv3280 | *accD5* | 548 | 78 | 85 | 85 | 84 | 67 | 85 | 64 | 79 | 87 | 87 | 88 | 88 | 91 | 94 | 95 | 94 | 99 | 91 | 92 | 92 | 92 | 91 | 99 | 100 | 100 | 95 | 91 |
| Rv3281 | - | 177 | 0 | 0 | 0 | 0 | 0 | 0 | 0 | 0 | 0 | 0 | 0 | 0 | 63 | 67 | 60 | 67 | 88 | 52 | 60 | 60 | 60 | 63 | 88 | 100 | 88 | 59 | 55 |
| Rv3282 | *maf* | 222 | 63 | 60 | 60 | 58 | 52 | 58 | 57 | 60 | 69 | 71 | 69 | 68 | 72 | 77 | 81 | 77 | 100 | 70 | 74 | 74 | 74 | 75 | 100 | 100 | 100 | 81 | 69 |
| Rv3283 | *sseA* | 297 | 65 | 73 | 74 | 73 | 62 | 65 | 40 | 68 | 76 | 84 | 83 | 83 | 85 | 91 | 90 | 91 | 99 | 89 | 88 | 88 | 88 | 90 | 100 | 100 | 100 | 90 | 91 |
| Rv3284 | - | 143 | 0 | 0 | 0 | 0 | 0 | 0 | 0 | 0 | 0 | 80 | 80 | 79 | 85 | 93 | 94 | 93 | 100 | 88 | 92 | 92 | 92 | 91 | 100 | 100 | 100 | 93 | 87 |
| Rv3285 | *accA3* | 600 | 81 | 83 | 82 | 81 | 78 | 81 | 77 | 77 | 87 | 85 | 88 | 88 | 91 | 94 | 95 | 94 | 100 | 91 | 92 | 92 | 92 | 91 | 100 | 100 | 100 | 95 | 91 |
| Rv3286c | *sigF* | 261 | 51 | 53 | 54 | 54 | 53 | 49 | 51 | 71 | 74 | 76 | 77 | 78 | 75 | 91 | 93 | 91 | 100 | 88 | 89 | 89 | 89 | 89 | 100 | 100 | 100 | 93 | 89 |
| Rv3287c | *rsbW* | 145 | 0 | 0 | 0 | 0 | 0 | 0 | 0 | 0 | 50 | 53 | 57 | 57 | 61 | 91 | 88 | 91 | 100 | 80 | 77 | 76 | 76 | 75 | 100 | 100 | 100 | 88 | 81 |
| Rv3288c | *usfY* | 137 | 0 | 0 | 0 | 0 | 0 | 0 | 0 | 0 | 56 | 0 | 0 | 0 | 53 | 87 | 85 | 87 | 100 | 60 | 65 | 65 | 65 | 65 | 100 | 100 | 100 | 85 | 58 |
| Rv3289c | - | 125 | 0 | 0 | 0 | 0 | 0 | 0 | 0 | 0 | 0 | 0 | 56 | 0 | 52 | 78 | 78 | 78 | 100 | 48 | 56 | 56 | 56 | 53 | 100 | 100 | 100 | 79 | 54 |
| Rv3290c | *lat* | 449 | 41 | 41 | 41 | 41 | 39 | 45 | 41 | 42 | 76 | 74 | 77 | 78 | 81 | 83 | 83 | 83 | 100 | 82 | 86 | 86 | 86 | 86 | 100 | 100 | 100 | 84 | 84 |
| Rv3291c | - | 150 | 0 | 0 | 0 | 52 | 0 | 0 | 0 | 73 | 89 | 86 | 89 | 88 | 91 | 92 | 92 | 92 | 99 | 90 | 88 | 89 | 89 | 91 | 100 | 100 | 100 | 92 | 87 |
| Rv3292 | - | 415 | 0 | 0 | 64 | 0 | 0 | 0 | 0 | 0 | 0 | 68 | 63 | 68 | 76 | 84 | 84 | 84 | 100 | 80 | 0 | 0 | 0 | 0 | 100 | 100 | 100 | 84 | 81 |
| Rv3293 | *pcd* | 494 | 48 | 49 | 51 | 51 | 47 | 51 | 47 | 50 | 78 | 79 | 83 | 83 | 85 | 87 | 89 | 87 | 99 | 86 | 87 | 88 | 88 | 87 | 100 | 100 | 100 | 89 | 87 |
| Rv3294c | - | 269 | 56 | 45 | 0 | 0 | 0 | 0 | 0 | 45 | 59 | 0 | 0 | 0 | 0 | 0 | 0 | 0 | 100 | 0 | 0 | 0 | 0 | 0 | 100 | 100 | 100 | 0 | 0 |
| Rv3295 | - | 221 | 0 | 56 | 0 | 0 | 0 | 0 | 0 | 47 | 49 | 51 | 54 | 54 | 65 | 90 | 91 | 91 | 100 | 79 | 0 | 0 | 0 | 0 | 100 | 100 | 100 | 91 | 77 |
| Rv3296 | *lhr* | 1513 | 62 | 62 | 63 | 63 | 56 | 43 | 56 | 72 | 71 | 74 | 75 | 74 | 78 | 89 | 89 | 89 | 100 | 84 | 86 | 85 | 85 | 85 | 100 | 100 | 100 | 91 | 85 |
| Rv3297 | *nei* | 255 | 57 | 57 | 60 | 57 | 55 | 40 | 52 | 62 | 57 | 62 | 64 | 62 | 67 | 85 | 84 | 85 | 100 | 74 | 78 | 79 | 79 | 79 | 100 | 100 | 100 | 84 | 74 |
| Rv3298c | *lpqC* | 304 | 48 | 44 | 43 | 40 | 40 | 42 | 39 | 0 | 0 | 0 | 0 | 0 | 0 | 80 | 82 | 80 | 100 | 0 | 0 | 0 | 0 | 69 | 100 | 100 | 100 | 82 | 0 |
| Rv3299c | *atsB* | 970 | 0 | 0 | 45 | 0 | 0 | 0 | 0 | 55 | 0 | 57 | 59 | 57 | 53 | 51 | 51 | 51 | 99 | 50 | 56 | 56 | 56 | 51 | 100 | 100 | 100 | 92 | 56 |
| Rv3300c | - | 305 | 59 | 59 | 59 | 59 | 58 | 49 | 49 | 41 | 67 | 66 | 66 | 66 | 73 | 83 | 84 | 84 | 100 | 77 | 78 | 78 | 78 | 78 | 99 | 100 | 100 | 85 | 79 |
| Rv3301c | *phoY1* | 221 | 61 | 60 | 61 | 64 | 59 | 60 | 55 | 59 | 79 | 79 | 80 | 80 | 81 | 81 | 89 | 81 | 100 | 79 | 79 | 79 | 79 | 77 | 100 | 100 | 100 | 89 | 80 |
| Rv3302c | *glpD2* | 585 | 71 | 71 | 0 | 49 | 0 | 72 | 0 | 67 | 85 | 88 | 88 | 88 | 90 | 93 | 95 | 93 | 100 | 91 | 91 | 91 | 91 | 91 | 100 | 100 | 100 | 95 | 90 |
| Rv3303c | *lpdA* | 493 | 76 | 77 | 75 | 76 | 44 | 74 | 45 | 71 | 80 | 85 | 83 | 83 | 85 | 91 | 94 | 91 | 99 | 86 | 85 | 85 | 85 | 87 | 99 | 100 | 99 | 93 | 86 |

| | | | | | | | | | | | | | | | | | | | | | | | | | | | | | |
|---|---|---|---|---|---|---|---|---|---|---|---|---|---|---|---|---|---|---|---|---|---|---|---|---|---|---|---|---|---|
| Rv3304 | - | 159 | 0 | 0 | 0 | 0 | 0 | 0 | 0 | 76 | 85 | 86 | 87 | 87 | 94 | 98 | 98 | 98 | 100 | 91 | 95 | 95 | 95 | 95 | 100 | 100 | 100 | 98 | 91 |
| Rv3305c | *amiA1* | 389 | 68 | 64 | 71 | 69 | 66 | 46 | 66 | 65 | 81 | 83 | 82 | 82 | 86 | 94 | 96 | 94 | 100 | 91 | 92 | 92 | 92 | 88 | 100 | 100 | 100 | 96 | 91 |
| Rv3306c | *amiB1* | 394 | 50 | 41 | 50 | 50 | 42 | 38 | 42 | 40 | 73 | 70 | 70 | 70 | 81 | 89 | 91 | 90 | 100 | 84 | 86 | 86 | 86 | 85 | 100 | 100 | 100 | 90 | 83 |
| Rv3307 | *deoD* | 268 | 0 | 0 | 0 | 0 | 70 | 0 | 70 | 70 | 76 | 75 | 77 | 77 | 81 | 89 | 92 | 89 | 100 | 83 | 86 | 86 | 86 | 85 | 100 | 100 | 100 | 92 | 84 |
| Rv3308 | *pmmB* | 534 | 58 | 59 | 52 | 52 | 55 | 52 | 56 | 56 | 63 | 66 | 69 | 69 | 71 | 82 | 83 | 82 | 99 | 75 | 74 | 74 | 74 | 73 | 100 | 100 | 100 | 83 | 75 |
| Rv3309c | *upp* | 207 | 77 | 78 | 77 | 77 | 77 | 77 | 73 | 67 | 81 | 82 | 82 | 81 | 82 | 86 | 89 | 85 | 100 | 87 | 86 | 86 | 86 | 87 | 100 | 100 | 100 | 88 | 87 |
| Rv3310 | - | 299 | 0 | 0 | 0 | 0 | 0 | 0 | 0 | 47 | 0 | 0 | 0 | 0 | 0 | 83 | 85 | 82 | 99 | 0 | 0 | 0 | 0 | 0 | 100 | 100 | 100 | 84 | 0 |
| Rv3311 | - | 420 | 53 | 52 | 53 | 54 | 54 | 50 | 54 | 0 | 67 | 66 | 66 | 65 | 73 | 83 | 83 | 83 | 100 | 78 | 77 | 76 | 76 | 77 | 99 | 100 | 100 | 83 | 79 |
| Rv3312A | - | 103 | 0 | 0 | 0 | 0 | 0 | 0 | 0 | 0 | 0 | 0 | 0 | 0 | 61 | 52 | 70 | 52 | 100 | 0 | 0 | 0 | 0 | 0 | 100 | 100 | 100 | 53 | 0 |
| Rv3312c | - | 308 | 0 | 0 | 0 | 0 | 0 | 0 | 0 | 45 | 0 | 43 | 45 | 45 | 0 | 45 | 86 | 46 | 100 | 44 | 45 | 45 | 45 | 46 | 100 | 100 | 100 | 85 | 43 |
| Rv3313c | *add* | 365 | 0 | 0 | 0 | 0 | 62 | 61 | 63 | 76 | 84 | 84 | 82 | 82 | 86 | 91 | 91 | 91 | 100 | 88 | 89 | 89 | 89 | 90 | 100 | 100 | 100 | 91 | 89 |
| Rv3314c | *deoA* | 427 | 73 | 0 | 0 | 0 | 0 | 73 | 0 | 73 | 75 | 76 | 76 | 78 | 77 | 88 | 91 | 88 | 99 | 82 | 83 | 81 | 81 | 82 | 100 | 100 | 100 | 91 | 83 |
| Rv3315c | *cdd* | 133 | 51 | 0 | 0 | 0 | 0 | 57 | 0 | 72 | 71 | 77 | 72 | 73 | 79 | 84 | 84 | 84 | 100 | 80 | 82 | 81 | 81 | 79 | 100 | 100 | 100 | 84 | 82 |
| Rv3316 | *sdhC* | 112 | 0 | 0 | 0 | 0 | 0 | 0 | 0 | 0 | 84 | 81 | 87 | 87 | 90 | 90 | 89 | 90 | 100 | 84 | 84 | 84 | 84 | 85 | 100 | 100 | 100 | 88 | 85 |
| Rv3317 | *sdhD* | 144 | 0 | 0 | 0 | 0 | 0 | 0 | 0 | 0 | 85 | 85 | 86 | 86 | 95 | 95 | 96 | 95 | 100 | 92 | 92 | 93 | 93 | 88 | 100 | 100 | 100 | 95 | 93 |
| Rv3318 | *sdhA* | 590 | 44 | 44 | 44 | 43 | 44 | 45 | 45 | 54 | 92 | 90 | 91 | 91 | 95 | 96 | 96 | 96 | 100 | 95 | 95 | 95 | 95 | 96 | 100 | 100 | 100 | 96 | 95 |
| Rv3319 | *sdhB* | 263 | 0 | 0 | 0 | 0 | 0 | 0 | 0 | 41 | 91 | 90 | 91 | 91 | 93 | 95 | 95 | 95 | 100 | 93 | 89 | 89 | 89 | 93 | 100 | 100 | 100 | 98 | 93 |
| Rv3320c | - | 142 | 0 | 0 | 0 | 0 | 0 | 0 | 0 | 0 | 0 | 0 | 0 | 0 | 0 | 0 | 0 | 0 | 100 | 0 | 0 | 0 | 0 | 0 | 100 | 100 | 100 | 0 | 0 |
| Rv3321c | - | 80 | 0 | 0 | 0 | 0 | 0 | 0 | 0 | 0 | 0 | 0 | 0 | 0 | 0 | 0 | 0 | 0 | 100 | 0 | 0 | 0 | 0 | 0 | 100 | 100 | 100 | 0 | 0 |
| Rv3322c | - | 204 | 0 | 0 | 0 | 0 | 0 | 0 | 0 | 0 | 0 | 0 | 0 | 0 | 0 | 0 | 0 | 0 | 100 | 49 | 46 | 46 | 46 | 0 | 100 | 100 | 100 | 0 | 51 |
| Rv3323c | *moaX* | 221 | 56 | 56 | 55 | 55 | 50 | 0 | 52 | 59 | 56 | 61 | 56 | 60 | 58 | 56 | 60 | 57 | 100 | 60 | 61 | 61 | 61 | 58 | 100 | 100 | 100 | 60 | 61 |
| Rv3324c | *moaC* | 184 | 62 | 67 | 64 | 62 | 63 | 0 | 60 | 69 | 63 | 63 | 67 | 66 | 64 | 65 | 67 | 66 | 100 | 67 | 67 | 67 | 67 | 68 | 100 | 100 | 100 | 65 | 69 |
| Rv3325 | - | 108 | 65 | 0 | 66 | 0 | 61 | 0 | 60 | 76 | 0 | 0 | 0 | 0 | 59 | 63 | 82 | 66 | 100 | 82 | 96 | 77 | 77 | 84 | 100 | 100 | 100 | 0 | 62 |
| Rv3326 | - | 312 | 58 | 49 | 61 | 51 | 63 | 0 | 61 | 56 | 0 | 0 | 51 | 52 | 66 | 59 | 66 | 66 | 100 | 66 | 90 | 67 | 67 | 66 | 100 | 100 | 100 | 0 | 65 |
| Rv3327 | - | 570 | 0 | 0 | 45 | 38 | 40 | 0 | 0 | 58 | 54 | 76 | 75 | 68 | 77 | 80 | 90 | 81 | 100 | 74 | 85 | 86 | 86 | 78 | 100 | 100 | 99 | 83 | 84 |
| Rv3328c | *sigJ* | 312 | 0 | 0 | 0 | 0 | 0 | 0 | 0 | 51 | 52 | 63 | 68 | 68 | 79 | 83 | 77 | 84 | 99 | 78 | 79 | 81 | 81 | 80 | 99 | 100 | 91 | 78 | 77 |
| Rv3329 | - | 438 | 48 | 48 | 51 | 50 | 52 | 53 | 50 | 79 | 54 | 53 | 87 | 87 | 88 | 90 | 88 | 90 | 99 | 88 | 88 | 88 | 88 | 88 | 99 | 100 | 99 | 88 | 86 |
| Rv3330 | *dacB1* | 405 | 56 | 57 | 63 | 68 | 0 | 57 | 0 | 0 | 62 | 65 | 62 | 62 | 75 | 85 | 84 | 84 | 100 | 77 | 79 | 79 | 79 | 78 | 100 | 100 | 100 | 84 | 78 |
| Rv3331 | *sugI* | 502 | 49 | 43 | 44 | 52 | 0 | 44 | 44 | 43 | 39 | 45 | 51 | 52 | 71 | 76 | 78 | 74 | 99 | 47 | 50 | 50 | 50 | 51 | 95 | 99 | 99 | 78 | 49 |
| Rv3332 | *nagA* | 383 | 58 | 52 | 0 | 49 | 0 | 53 | 0 | 46 | 0 | 54 | 54 | 55 | 66 | 81 | 80 | 79 | 100 | 0 | 0 | 0 | 0 | 67 | 100 | 100 | 100 | 80 | 0 |
| Rv3333c | - | 281 | 0 | 0 | 0 | 0 | 0 | 0 | 0 | 46 | 43 | 0 | 49 | 0 | 0 | 49 | 49 | 49 | 100 | 48 | 46 | 46 | 46 | 48 | 100 | 100 | 100 | 62 | 62 |
| Rv3334 | - | 146 | 0 | 0 | 0 | 0 | 61 | 0 | 61 | 59 | 0 | 54 | 0 | 60 | 0 | 0 | 0 | 0 | 100 | 61 | 50 | 50 | 50 | 0 | 100 | 100 | 100 | 0 | 51 |
| Rv3335c | - | 289 | 57 | 58 | 59 | 61 | 59 | 61 | 58 | 41 | 67 | 68 | 66 | 66 | 73 | 83 | 83 | 83 | 100 | 75 | 77 | 73 | 73 | 76 | 100 | 100 | 100 | 82 | 75 |
| Rv3336c | *trpS* | 336 | 75 | 78 | 77 | 77 | 77 | 78 | 78 | 74 | 79 | 81 | 81 | 81 | 83 | 92 | 92 | 92 | 100 | 90 | 89 | 88 | 88 | 90 | 100 | 100 | 100 | 94 | 90 |
| Rv3337 | - | 128 | 0 | 0 | 0 | 0 | 0 | 0 | 0 | 0 | 54 | 0 | 0 | 0 | 86 | 83 | 91 | 85 | 100 | 78 | 72 | 87 | 87 | 70 | 100 | 100 | 100 | 89 | 67 |
| Rv3338 | - | 214 | 0 | 0 | 0 | 0 | 0 | 0 | 0 | 0 | 55 | 0 | 0 | 0 | 66 | 87 | 85 | 87 | 100 | 71 | 77 | 77 | 77 | 78 | 100 | 100 | 100 | 85 | 77 |
| Rv3339c | *icd1* | 409 | 0 | 0 | 0 | 0 | 0 | 0 | 0 | 0 | 89 | 88 | 87 | 87 | 0 | 94 | 95 | 94 | 100 | 0 | 0 | 0 | 0 | 0 | 100 | 100 | 100 | 95 | 0 |
| Rv3340 | *metC* | 449 | 73 | 74 | 73 | 74 | 73 | 70 | 71 | 69 | 87 | 86 | 88 | 89 | 85 | 93 | 93 | 93 | 100 | 90 | 91 | 91 | 91 | 91 | 100 | 100 | 100 | 95 | 90 |

| | | | | | | | | | | | | | | | | | | | | | | | | | | | | | |
|---|---|---|---|---|---|---|---|---|---|---|---|---|---|---|---|---|---|---|---|---|---|---|---|---|---|---|---|---|---|
| Rv3341 | *metX* | 379 | 62 | 64 | 62 | 63 | 61 | 66 | 58 | 51 | 79 | 79 | 79 | 79 | 83 | 89 | 89 | 89 | 100 | 87 | 89 | 89 | 89 | 87 | 99 | 100 | 100 | 94 | 86 |
| Rv3342 | - | 243 | 50 | 49 | 50 | 51 | 0 | 45 | 0 | 56 | 0 | 0 | 47 | 53 | 82 | 90 | 94 | 89 | 100 | 86 | 87 | 87 | 87 | 86 | 100 | 100 | 100 | 94 | 86 |
| Rv3343c | *PPE54* | 2523 | 0 | 0 | 0 | 0 | 0 | 0 | 0 | 0 | 43 | 35 | 36 | 36 | 50 | 63 | 54 | 46 | 99 | 51 | 52 | 52 | 52 | 53 | 62 | 91 | 94 | 56 | 52 |
| Rv3344c | *PE_PGRS49* | 484 | 39 | 38 | 36 | 32 | 32 | 41 | 34 | 35 | 39 | 38 | 41 | 42 | 36 | 46 | 34 | 34 | 100 | 53 | 51 | 52 | 51 | 51 | 100 | 100 | 100 | 55 | 52 |
| Rv3345c | *PE_PGRS50* | 1538 | 43 | 48 | 31 | 33 | 35 | 35 | 36 | 35 | 36 | 38 | 37 | 37 | 42 | 29 | 53 | 32 | 84 | 50 | 50 | 51 | 51 | 45 | 85 | 100 | 77 | 54 | 47 |
| Rv3346c | - | 85 | 0 | 0 | 0 | 0 | 0 | 0 | 0 | 0 | 0 | 0 | 67 | 63 | 61 | 74 | 73 | 74 | 100 | 64 | 65 | 65 | 65 | 68 | 100 | 100 | 100 | 73 | 67 |
| Rv3347c | *PPE55* | 3157 | 0 | 0 | 0 | 0 | 0 | 0 | 0 | 0 | 34 | 39 | 37 | 36 | 50 | 70 | 46 | 70 | 99 | 50 | 49 | 49 | 49 | 50 | 100 | 100 | 99 | 53 | 50 |
| Rv3348 | - | 163 | 0 | 0 | 0 | 0 | 0 | 0 | 0 | 0 | 0 | 0 | 0 | 0 | 0 | 0 | 0 | 0 | 100 | 0 | 0 | 0 | 0 | 0 | 100 | 100 | 100 | 0 | 0 |
| Rv3349c | - | 246 | 0 | 0 | 0 | 0 | 0 | 0 | 0 | 0 | 0 | 0 | 0 | 0 | 0 | 0 | 0 | 0 | 100 | 0 | 0 | 0 | 0 | 58 | 100 | 100 | 100 | 0 | 0 |
| Rv3350c | *PPE56* | 3716 | 0 | 0 | 0 | 0 | 0 | 0 | 0 | 0 | 35 | 39 | 36 | 39 | 48 | 67 | 50 | 67 | 99 | 47 | 49 | 49 | 49 | 36 | 99 | 100 | 100 | 52 | 48 |
| Rv3351c | - | 264 | 0 | 0 | 0 | 0 | 0 | 0 | 0 | 0 | 0 | 0 | 0 | 0 | 0 | 70 | 83 | 70 | 100 | 0 | 0 | 0 | 0 | 0 | 100 | 100 | 100 | 82 | 0 |
| Rv3352c | - | 123 | 0 | 0 | 0 | 0 | 0 | 0 | 0 | 62 | 59 | 64 | 67 | 67 | 59 | 77 | 84 | 76 | 100 | 52 | 56 | 56 | 56 | 53 | 100 | 100 | 100 | 84 | 57 |
| Rv3353c | - | 86 | 0 | 0 | 0 | 0 | 0 | 0 | 0 | 0 | 0 | 0 | 0 | 0 | 0 | 67 | 76 | 67 | 100 | 0 | 0 | 0 | 0 | 0 | 100 | 100 | 100 | 77 | 0 |
| Rv3354 | - | 129 | 0 | 0 | 0 | 0 | 0 | 0 | 0 | 0 | 0 | 0 | 0 | 0 | 0 | 78 | 77 | 78 | 99 | 57 | 64 | 64 | 64 | 55 | 100 | 100 | 100 | 78 | 60 |
| Rv3355c | - | 97 | 0 | 0 | 0 | 0 | 0 | 0 | 0 | 0 | 59 | 55 | 61 | 59 | 67 | 82 | 82 | 82 | 100 | 70 | 73 | 73 | 73 | 77 | 100 | 100 | 100 | 83 | 75 |
| Rv3356c | *folD* | 281 | 81 | 82 | 82 | 82 | 83 | 81 | 83 | 77 | 57 | 89 | 87 | 87 | 89 | 94 | 95 | 95 | 99 | 91 | 91 | 91 | 91 | 92 | 100 | 100 | 100 | 95 | 91 |
| Rv3357 | - | 91 | 0 | 0 | 0 | 0 | 0 | 0 | 0 | 0 | 0 | 0 | 0 | 0 | 0 | 0 | 0 | 0 | 100 | 0 | 0 | 0 | 0 | 0 | 100 | 100 | 100 | 0 | 0 |
| Rv3358 | - | 85 | 70 | 0 | 0 | 0 | 0 | 0 | 0 | 0 | 0 | 0 | 0 | 71 | 0 | 0 | 0 | 0 | 100 | 0 | 0 | 0 | 0 | 0 | 100 | 100 | 100 | 0 | 0 |
| Rv3359 | - | 396 | 47 | 44 | 44 | 48 | 46 | 0 | 42 | 68 | 75 | 77 | 76 | 77 | 82 | 93 | 93 | 93 | 100 | 89 | 89 | 89 | 89 | 89 | 100 | 100 | 100 | 91 | 90 |
| Rv3360 | - | 122 | 0 | 0 | 0 | 0 | 0 | 0 | 0 | 0 | 0 | 0 | 0 | 0 | 66 | 77 | 77 | 77 | 100 | 72 | 78 | 78 | 78 | 79 | 100 | 100 | 100 | 78 | 76 |
| Rv3361c | - | 183 | 0 | 0 | 0 | 0 | 0 | 0 | 0 | 49 | 0 | 74 | 72 | 72 | 71 | 60 | 90 | 82 | 100 | 75 | 77 | 77 | 77 | 81 | 100 | 100 | 100 | 90 | 79 |
| Rv3362c | - | 193 | 0 | 0 | 0 | 0 | 0 | 0 | 0 | 0 | 83 | 82 | 80 | 80 | 0 | 95 | 92 | 95 | 100 | 89 | 88 | 88 | 88 | 87 | 100 | 100 | 100 | 89 | 86 |
| Rv3363c | - | 122 | 0 | 0 | 0 | 0 | 0 | 0 | 0 | 0 | 66 | 63 | 68 | 68 | 0 | 87 | 88 | 88 | 100 | 74 | 80 | 80 | 80 | 80 | 100 | 100 | 100 | 89 | 73 |
| Rv3364c | - | 130 | 0 | 0 | 0 | 0 | 0 | 0 | 0 | 0 | 82 | 87 | 83 | 83 | 0 | 92 | 92 | 92 | 100 | 85 | 88 | 88 | 88 | 88 | 100 | 100 | 100 | 92 | 88 |
| Rv3365c | - | 876 | 0 | 0 | 42 | 0 | 0 | 0 | 0 | 42 | 51 | 48 | 50 | 49 | 0 | 74 | 74 | 74 | 100 | 63 | 67 | 67 | 67 | 61 | 100 | 100 | 99 | 78 | 62 |
| Rv3366 | *spoU* | 154 | 69 | 72 | 76 | 74 | 70 | 65 | 69 | 0 | 81 | 85 | 83 | 82 | 83 | 90 | 90 | 90 | 100 | 87 | 86 | 86 | 86 | 87 | 100 | 100 | 100 | 89 | 87 |
| Rv3367 | *PE_PGRS51* | 588 | 41 | 45 | 35 | 41 | 36 | 45 | 34 | 38 | 37 | 35 | 39 | 39 | 42 | 41 | 81 | 34 | 93 | 50 | 44 | 48 | 48 | 46 | 99 | 99 | 99 | 59 | 48 |
| Rv3368c | - | 214 | 0 | 0 | 0 | 0 | 0 | 0 | 0 | 46 | 66 | 61 | 0 | 0 | 73 | 89 | 93 | 90 | 100 | 79 | 79 | 79 | 79 | 79 | 100 | 100 | 100 | 92 | 78 |
| Rv3369 | - | 144 | 0 | 0 | 0 | 0 | 0 | 0 | 0 | 0 | 51 | 0 | 53 | 54 | 0 | 0 | 0 | 0 | 100 | 0 | 0 | 0 | 0 | 0 | 100 | 100 | 100 | 86 | 0 |
| Rv3370c | *dnaE2* | 1091 | 65 | 62 | 66 | 66 | 64 | 66 | 64 | 62 | 78 | 83 | 83 | 83 | 84 | 93 | 93 | 93 | 100 | 88 | 90 | 90 | 90 | 89 | 100 | 100 | 100 | 92 | 88 |
| Rv3371 | - | 446 | 0 | 0 | 0 | 0 | 0 | 0 | 0 | 47 | 41 | 51 | 51 | 43 | 43 | 63 | 73 | 65 | 99 | 61 | 58 | 58 | 58 | 56 | 99 | 100 | 100 | 72 | 57 |
| Rv3372 | *otsB2* | 391 | 0 | 45 | 46 | 0 | 40 | 40 | 43 | 43 | 46 | 68 | 61 | 51 | 48 | 78 | 83 | 78 | 100 | 64 | 65 | 65 | 65 | 46 | 100 | 100 | 100 | 83 | 64 |
| Rv3373 | *echA18* | 213 | 55 | 48 | 53 | 0 | 53 | 51 | 50 | 54 | 54 | 55 | 54 | 54 | 53 | 53 | 53 | 53 | 100 | 54 | 51 | 51 | 51 | 50 | 100 | 100 | 100 | 55 | 55 |
| Rv3374 | *echA18.1* | 82 | 0 | 0 | 0 | 0 | 0 | 0 | 0 | 0 | 0 | 0 | 0 | 0 | 0 | 0 | 0 | 0 | 100 | 0 | 0 | 0 | 0 | 0 | 100 | 100 | 100 | 0 | 0 |
| Rv3375 | *amiD* | 475 | 45 | 46 | 52 | 45 | 46 | 45 | 46 | 43 | 51 | 45 | 48 | 46 | 47 | 45 | 61 | 45 | 100 | 47 | 45 | 45 | 45 | 53 | 100 | 100 | 100 | 63 | 43 |
| Rv3376 | - | 217 | 0 | 0 | 50 | 0 | 0 | 0 | 0 | 0 | 0 | 0 | 0 | 0 | 0 | 0 | 0 | 0 | 100 | 0 | 0 | 0 | 0 | 0 | 100 | 100 | 100 | 0 | 0 |
| Rv3377c | - | 501 | 0 | 0 | 0 | 0 | 0 | 0 | 0 | 0 | 0 | 0 | 0 | 0 | 0 | 0 | 0 | 0 | 98 | 0 | 0 | 0 | 0 | 0 | 100 | 100 | 100 | 0 | 0 |
| Rv3378c | - | 296 | 0 | 0 | 0 | 0 | 0 | 0 | 0 | 0 | 0 | 0 | 0 | 0 | 0 | 0 | 0 | 0 | 100 | 0 | 0 | 0 | 0 | 0 | 100 | 100 | 100 | 0 | 0 |

| | | | | | | | | | | | | | | | | | | | | | | | | | | | | | |
|---|---|---|---|---|---|---|---|---|---|---|---|---|---|---|---|---|---|---|---|---|---|---|---|---|---|---|---|---|---|
| Rv3379c | *dxs2* | 536 | 58 | 58 | 59 | 58 | 58 | 0 | 56 | 60 | 62 | 61 | 62 | 62 | 63 | 62 | 62 | 62 | 99 | 61 | 61 | 61 | 61 | 63 | 100 | 100 | 100 | 62 | 61 |
| Rv3380c | - | 294 | 58 | 49 | 61 | 51 | 63 | 0 | 61 | 56 | 0 | 0 | 53 | 52 | 66 | 60 | 68 | 68 | 100 | 66 | 90 | 67 | 67 | 66 | 100 | 100 | 100 | 0 | 65 |
| Rv3381c | - | 108 | 65 | 0 | 66 | 0 | 61 | 0 | 60 | 76 | 0 | 0 | 0 | 0 | 59 | 63 | 82 | 66 | 100 | 82 | 96 | 77 | 77 | 84 | 100 | 100 | 100 | 0 | 62 |
| Rv3382c | *lytB1* | 329 | 68 | 65 | 66 | 67 | 68 | 0 | 67 | 69 | 68 | 68 | 68 | 68 | 68 | 68 | 84 | 68 | 100 | 68 | 66 | 67 | 67 | 69 | 100 | 100 | 100 | 67 | 67 |
| Rv3383c | *idsB* | 350 | 45 | 45 | 48 | 45 | 46 | 50 | 47 | 45 | 45 | 47 | 48 | 48 | 45 | 43 | 72 | 45 | 99 | 45 | 46 | 46 | 46 | 44 | 99 | 100 | 99 | 71 | 44 |
| Rv3384c | - | 130 | 0 | 0 | 0 | 0 | 0 | 0 | 0 | 0 | 0 | 0 | 0 | 0 | 0 | 0 | 0 | 0 | 100 | 0 | 0 | 0 | 0 | 0 | 100 | 100 | 100 | 0 | 61 |
| Rv3385c | - | 102 | 0 | 0 | 0 | 0 | 0 | 0 | 0 | 0 | 0 | 0 | 0 | 0 | 0 | 0 | 0 | 0 | 100 | 0 | 0 | 0 | 0 | 0 | 100 | 100 | 100 | 0 | 0 |
| Rv3386 | - | 234 | 0 | 0 | 0 | 0 | 0 | 0 | 0 | 0 | 0 | 0 | 0 | 0 | 0 | 0 | 0 | 0 | 100 | 0 | 0 | 0 | 0 | 0 | 100 | 100 | 100 | 0 | 0 |
| Rv3387 | - | 225 | 0 | 0 | 0 | 0 | 0 | 0 | 0 | 0 | 0 | 0 | 0 | 0 | 0 | 0 | 0 | 0 | 100 | 0 | 0 | 0 | 0 | 0 | 100 | 100 | 100 | 0 | 0 |
| Rv3388 | *PE_PGRS52* | 731 | 45 | 33 | 33 | 33 | 35 | 35 | 38 | 35 | 38 | 39 | 40 | 42 | 38 | 48 | 58 | 35 | 93 | 48 | 50 | 50 | 50 | 49 | 99 | 87 | 100 | 52 | 51 |
| Rv3389c | - | 290 | 0 | 0 | 43 | 42 | 0 | 0 | 0 | 55 | 54 | 53 | 53 | 81 | 53 | 91 | 91 | 91 | 99 | 84 | 83 | 84 | 84 | 55 | 100 | 100 | 100 | 91 | 86 |
| Rv3390 | *lpqD* | 236 | 0 | 44 | 43 | 0 | 0 | 0 | 0 | 0 | 0 | 0 | 0 | 60 | 0 | 81 | 83 | 81 | 100 | 55 | 64 | 64 | 64 | 59 | 99 | 100 | 100 | 82 | 57 |
| Rv3391 | *acrA1* | 650 | 50 | 48 | 48 | 47 | 42 | 49 | 48 | 59 | 70 | 68 | 68 | 68 | 77 | 86 | 86 | 86 | 100 | 80 | 59 | 59 | 59 | 80 | 100 | 100 | 100 | 85 | 82 |
| Rv3392c | *cmaA1* | 287 | 46 | 0 | 0 | 47 | 0 | 47 | 41 | 46 | 44 | 48 | 49 | 50 | 82 | 82 | 88 | 82 | 100 | 81 | 92 | 91 | 91 | 86 | 99 | 100 | 100 | 87 | 81 |
| Rv3393 | *iunH* | 308 | 50 | 49 | 49 | 49 | 44 | 48 | 40 | 49 | 63 | 63 | 65 | 64 | 68 | 73 | 80 | 74 | 100 | 69 | 70 | 70 | 70 | 70 | 100 | 100 | 100 | 79 | 69 |
| Rv3394c | - | 527 | 52 | 48 | 53 | 52 | 48 | 51 | 51 | 49 | 71 | 72 | 73 | 73 | 76 | 88 | 88 | 88 | 100 | 81 | 86 | 86 | 86 | 85 | 100 | 100 | 100 | 90 | 84 |
| Rv3395A | - | 208 | 0 | 0 | 0 | 0 | 0 | 0 | 0 | 0 | 0 | 0 | 0 | 0 | 52 | 0 | 0 | 0 | 100 | 0 | 0 | 0 | 0 | 0 | 100 | 100 | 100 | 67 | 0 |
| Rv3395c | - | 204 | 49 | 0 | 45 | 48 | 46 | 47 | 48 | 51 | 65 | 69 | 69 | 60 | 65 | 79 | 84 | 79 | 99 | 71 | 72 | 73 | 73 | 71 | 100 | 100 | 100 | 83 | 71 |
| Rv3396c | *guaA* | 525 | 86 | 86 | 86 | 85 | 85 | 85 | 86 | 79 | 89 | 89 | 90 | 90 | 88 | 93 | 93 | 93 | 100 | 92 | 92 | 92 | 92 | 92 | 100 | 100 | 100 | 93 | 92 |
| Rv3397c | *phyA* | 302 | 41 | 0 | 0 | 44 | 0 | 0 | 0 | 0 | 42 | 43 | 38 | 44 | 0 | 45 | 44 | 43 | 100 | 43 | 42 | 42 | 42 | 42 | 100 | 100 | 100 | 44 | 42 |
| Rv3398c | *idsA1* | 359 | 45 | 50 | 48 | 46 | 50 | 51 | 51 | 43 | 47 | 52 | 50 | 50 | 46 | 50 | 62 | 50 | 100 | 48 | 48 | 48 | 48 | 48 | 100 | 100 | 100 | 60 | 43 |
| Rv3399 | - | 348 | 0 | 0 | 0 | 0 | 0 | 0 | 0 | 0 | 48 | 57 | 58 | 0 | 66 | 73 | 72 | 73 | 99 | 69 | 70 | 70 | 70 | 70 | 99 | 100 | 99 | 71 | 66 |
| Rv3400 | - | 262 | 0 | 0 | 59 | 0 | 65 | 56 | 60 | 61 | 59 | 54 | 69 | 70 | 43 | 84 | 85 | 85 | 100 | 76 | 86 | 86 | 86 | 82 | 100 | 100 | 100 | 89 | 76 |
| Rv3401 | - | 786 | 0 | 0 | 41 | 0 | 56 | 55 | 55 | 56 | 40 | 41 | 68 | 68 | 0 | 92 | 95 | 91 | 99 | 86 | 89 | 89 | 89 | 91 | 100 | 100 | 100 | 93 | 87 |
| Rv3402c | - | 412 | 0 | 0 | 0 | 45 | 0 | 0 | 0 | 44 | 36 | 43 | 44 | 43 | 0 | 0 | 38 | 0 | 99 | 0 | 0 | 0 | 0 | 0 | 100 | 100 | 100 | 0 | 0 |
| Rv3403c | - | 533 | 0 | 0 | 0 | 0 | 0 | 0 | 0 | 0 | 0 | 0 | 0 | 0 | 0 | 0 | 0 | 0 | 100 | 0 | 0 | 0 | 0 | 0 | 100 | 100 | 100 | 0 | 0 |
| Rv3404c | - | 234 | 0 | 0 | 0 | 0 | 52 | 0 | 52 | 0 | 0 | 0 | 0 | 0 | 0 | 0 | 0 | 0 | 100 | 0 | 0 | 0 | 0 | 0 | 100 | 100 | 100 | 0 | 0 |
| Rv3405c | - | 188 | 0 | 0 | 0 | 0 | 0 | 0 | 0 | 0 | 0 | 0 | 0 | 0 | 68 | 0 | 80 | 82 | 100 | 0 | 70 | 0 | 0 | 66 | 100 | 100 | 100 | 79 | 0 |
| Rv3406 | - | 295 | 0 | 0 | 0 | 0 | 0 | 0 | 0 | 0 | 66 | 68 | 70 | 70 | 66 | 48 | 89 | 87 | 100 | 67 | 71 | 71 | 71 | 76 | 100 | 100 | 100 | 89 | 43 |
| Rv3407 | - | 99 | 0 | 0 | 0 | 0 | 0 | 0 | 0 | 0 | 0 | 0 | 0 | 0 | 0 | 0 | 0 | 0 | 100 | 0 | 0 | 0 | 0 | 0 | 100 | 100 | 100 | 0 | 67 |
| Rv3408 | - | 136 | 0 | 0 | 0 | 0 | 0 | 0 | 0 | 0 | 0 | 0 | 0 | 0 | 0 | 0 | 0 | 0 | 99 | 0 | 0 | 0 | 0 | 0 | 99 | 100 | 99 | 0 | 58 |
| Rv3409c | *choD* | 578 | 0 | 0 | 0 | 0 | 75 | 0 | 75 | 72 | 81 | 83 | 81 | 82 | 87 | 98 | 94 | 93 | 100 | 89 | 88 | 88 | 88 | 89 | 100 | 100 | 100 | 94 | 89 |
| Rv3410c | *guaB3* | 375 | 70 | 72 | 72 | 75 | 74 | 76 | 71 | 69 | 83 | 87 | 87 | 88 | 92 | 97 | 96 | 96 | 100 | 93 | 95 | 94 | 94 | 95 | 100 | 100 | 100 | 95 | 93 |
| Rv3411c | *guaB2* | 529 | 81 | 83 | 82 | 82 | 84 | 84 | 80 | 77 | 91 | 89 | 90 | 90 | 92 | 96 | 96 | 96 | 100 | 93 | 94 | 93 | 94 | 94 | 100 | 100 | 100 | 96 | 92 |
| Rv3412 | - | 136 | 65 | 61 | 62 | 61 | 66 | 61 | 63 | 0 | 94 | 93 | 94 | 94 | 94 | 97 | 96 | 97 | 100 | 96 | 96 | 96 | 96 | 95 | 100 | 100 | 100 | 95 | 95 |
| Rv3413c | - | 299 | 40 | 0 | 0 | 0 | 0 | 0 | 0 | 0 | 41 | 44 | 41 | 41 | 50 | 62 | 63 | 63 | 100 | 55 | 53 | 58 | 58 | 52 | 100 | 100 | 100 | 68 | 55 |
| Rv3414c | *sigD* | 212 | 70 | 66 | 72 | 72 | 62 | 69 | 0 | 50 | 77 | 77 | 77 | 77 | 81 | 92 | 98 | 92 | 100 | 87 | 87 | 87 | 87 | 88 | 100 | 100 | 100 | 99 | 90 |
| Rv3415c | - | 275 | 0 | 0 | 0 | 0 | 0 | 0 | 0 | 0 | 52 | 52 | 56 | 56 | 65 | 78 | 78 | 78 | 100 | 64 | 65 | 64 | 64 | 67 | 100 | 100 | 100 | 0 | 64 |

| | | | | | | | | | | | | | | | | | | | | | | | | | | | | | |
|---|---|---|---|---|---|---|---|---|---|---|---|---|---|---|---|---|---|---|---|---|---|---|---|---|---|---|---|---|---|
| Rv3416 | *whiB3* | 102 | 78 | 55 | 78 | 79 | 81 | 68 | 57 | 62 | 88 | 88 | 84 | 84 | 87 | 95 | 95 | 95 | 100 | 89 | 88 | 88 | 88 | 88 | 100 | 100 | 100 | 97 | 89 |
| Rv3417c | *groEL* | 539 | 76 | 76 | 77 | 73 | 76 | 76 | 77 | 77 | 87 | 85 | 85 | 85 | 87 | 92 | 93 | 92 | 100 | 89 | 92 | 92 | 92 | 90 | 100 | 100 | 100 | 93 | 90 |
| Rv3418c | *groES* | 100 | 89 | 91 | 90 | 88 | 90 | 91 | 89 | 88 | 96 | 92 | 92 | 92 | 98 | 99 | 99 | 99 | 100 | 98 | 99 | 99 | 99 | 99 | 100 | 100 | 100 | 99 | 98 |
| Rv3419c | *gcp* | 344 | 73 | 75 | 75 | 75 | 67 | 70 | 70 | 71 | 81 | 82 | 81 | 81 | 86 | 92 | 92 | 92 | 100 | 89 | 89 | 89 | 89 | 90 | 100 | 100 | 100 | 92 | 89 |
| Rv3420c | *rimI* | 158 | 56 | 63 | 66 | 66 | 56 | 56 | 58 | 46 | 65 | 68 | 70 | 70 | 75 | 90 | 87 | 90 | 100 | 81 | 82 | 83 | 83 | 81 | 100 | 100 | 100 | 87 | 80 |
| Rv3421c | - | 211 | 60 | 62 | 61 | 63 | 50 | 58 | 49 | 55 | 62 | 64 | 67 | 68 | 70 | 87 | 86 | 86 | 100 | 81 | 80 | 80 | 80 | 78 | 100 | 100 | 100 | 86 | 82 |
| Rv3422c | - | 168 | 68 | 63 | 59 | 61 | 56 | 58 | 62 | 67 | 56 | 65 | 66 | 64 | 81 | 81 | 91 | 81 | 100 | 84 | 83 | 83 | 83 | 81 | 100 | 100 | 100 | 91 | 82 |
| Rv3423c | *alr* | 408 | 56 | 57 | 60 | 58 | 59 | 55 | 56 | 61 | 72 | 69 | 71 | 72 | 71 | 90 | 90 | 90 | 100 | 81 | 79 | 79 | 79 | 78 | 100 | 100 | 100 | 90 | 79 |
| Rv3424c | - | 120 | 0 | 0 | 0 | 0 | 0 | 0 | 0 | 0 | 0 | 0 | 0 | 0 | 0 | 0 | 0 | 0 | 100 | 0 | 0 | 0 | 0 | 0 | 100 | 100 | 100 | 0 | 0 |
| Rv3425 | *PPE57* | 176 | 0 | 0 | 0 | 0 | 0 | 0 | 0 | 0 | 0 | 0 | 0 | 0 | 45 | 54 | 52 | 54 | 93 | 0 | 43 | 43 | 43 | 46 | 77 | 100 | 77 | 51 | 43 |
| Rv3426 | *PPE58* | 232 | 0 | 0 | 0 | 0 | 0 | 0 | 0 | 0 | 0 | 0 | 0 | 0 | 0 | 52 | 52 | 53 | 75 | 0 | 0 | 0 | 0 | 0 | 80 | 100 | 80 | 50 | 0 |
| Rv3427c | - | 251 | 51 | 0 | 50 | 0 | 0 | 0 | 0 | 48 | 0 | 49 | 55 | 54 | 0 | 0 | 45 | 45 | 43 | 46 | 0 | 0 | 0 | 48 | 43 | 100 | 43 | 0 | 49 |
| Rv3428c | - | 410 | 42 | 0 | 45 | 0 | 0 | 0 | 0 | 0 | 0 | 0 | 78 | 0 | 0 | 0 | 0 | 0 | 0 | 0 | 0 | 0 | 0 | 0 | 0 | 100 | 0 | 0 | 0 |
| Rv3429 | *PPE59* | 178 | 0 | 0 | 0 | 0 | 0 | 0 | 0 | 0 | 0 | 0 | 0 | 0 | 43 | 50 | 50 | 50 | 84 | 39 | 42 | 42 | 42 | 42 | 100 | 100 | 99 | 47 | 0 |
| Rv3430c | - | 387 | 49 | 46 | 41 | 49 | 42 | 0 | 41 | 43 | 0 | 0 | 41 | 42 | 0 | 0 | 42 | 42 | 99 | 50 | 46 | 42 | 42 | 61 | 100 | 100 | 100 | 0 | 51 |
| Rv3431c | - | 281 | 49 | 61 | 59 | 0 | 60 | 0 | 58 | 72 | 0 | 44 | 59 | 90 | 0 | 74 | 0 | 74 | 100 | 96 | 49 | 60 | 60 | 95 | 100 | 100 | 100 | 52 | 73 |
| Rv3432c | *gadB* | 460 | 0 | 0 | 0 | 0 | 0 | 0 | 0 | 41 | 67 | 69 | 83 | 82 | 87 | 93 | 96 | 94 | 100 | 86 | 89 | 89 | 89 | 88 | 100 | 100 | 100 | 95 | 85 |
| Rv3433c | - | 473 | 51 | 53 | 47 | 48 | 50 | 43 | 55 | 55 | 72 | 71 | 70 | 70 | 79 | 93 | 93 | 93 | 100 | 84 | 85 | 85 | 85 | 85 | 100 | 100 | 100 | 91 | 85 |
| Rv3434c | - | 237 | 0 | 0 | 0 | 0 | 0 | 0 | 0 | 0 | 0 | 60 | 60 | 0 | 42 | 86 | 88 | 86 | 99 | 0 | 54 | 56 | 56 | 0 | 100 | 100 | 100 | 87 | 0 |
| Rv3435c | - | 284 | 0 | 0 | 0 | 0 | 0 | 0 | 0 | 0 | 0 | 0 | 49 | 47 | 52 | 67 | 67 | 67 | 100 | 51 | 0 | 0 | 0 | 0 | 99 | 100 | 100 | 73 | 0 |
| Rv3436c | *glmS* | 624 | 73 | 74 | 73 | 75 | 80 | 81 | 79 | 76 | 86 | 87 | 88 | 88 | 91 | 94 | 95 | 94 | 100 | 92 | 90 | 90 | 90 | 91 | 100 | 100 | 100 | 95 | 91 |
| Rv3437 | - | 158 | 0 | 0 | 0 | 0 | 0 | 0 | 0 | 0 | 0 | 0 | 0 | 0 | 61 | 0 | 70 | 0 | 99 | 0 | 0 | 0 | 0 | 0 | 100 | 100 | 100 | 69 | 0 |
| Rv3438 | - | 280 | 54 | 58 | 56 | 56 | 53 | 54 | 54 | 0 | 67 | 66 | 64 | 64 | 67 | 86 | 86 | 86 | 100 | 78 | 80 | 80 | 80 | 77 | 100 | 100 | 100 | 87 | 76 |
| Rv3439c | - | 467 | 0 | 0 | 35 | 0 | 37 | 0 | 36 | 0 | 39 | 0 | 0 | 0 | 40 | 60 | 60 | 63 | 100 | 49 | 53 | 53 | 53 | 45 | 100 | 100 | 99 | 60 | 50 |
| Rv3440c | - | 103 | 0 | 0 | 0 | 0 | 0 | 0 | 0 | 0 | 0 | 0 | 0 | 0 | 54 | 73 | 69 | 73 | 100 | 64 | 66 | 66 | 66 | 57 | 99 | 100 | 100 | 69 | 63 |
| Rv3441c | *mrsA* | 448 | 74 | 75 | 75 | 75 | 75 | 76 | 76 | 68 | 77 | 78 | 79 | 79 | 80 | 91 | 89 | 91 | 100 | 88 | 88 | 88 | 88 | 86 | 100 | 100 | 100 | 90 | 87 |
| Rv3442c | *rpsI* | 151 | 72 | 74 | 78 | 79 | 81 | 80 | 77 | 70 | 94 | 90 | 86 | 86 | 91 | 84 | 90 | 84 | 100 | 86 | 89 | 89 | 89 | 93 | 100 | 100 | 100 | 89 | 92 |
| Rv3443c | *rplM* | 147 | 76 | 79 | 79 | 78 | 80 | 78 | 81 | 80 | 88 | 88 | 89 | 89 | 91 | 93 | 96 | 93 | 100 | 87 | 90 | 90 | 90 | 91 | 100 | 100 | 100 | 95 | 91 |
| Rv3444c | *esxT* | 100 | 59 | 0 | 0 | 61 | 0 | 60 | 56 | 0 | 0 | 0 | 0 | 0 | 52 | 87 | 91 | 87 | 100 | 84 | 80 | 80 | 80 | 84 | 100 | 100 | 100 | 89 | 83 |
| Rv3445c | *esxU* | 125 | 0 | 0 | 0 | 0 | 0 | 0 | 0 | 0 | 0 | 0 | 0 | 0 | 0 | 89 | 73 | 82 | 100 | 74 | 70 | 70 | 70 | 74 | 100 | 100 | 100 | 72 | 75 |
| Rv3446c | - | 404 | 0 | 0 | 0 | 0 | 0 | 0 | 0 | 0 | 0 | 0 | 0 | 38 | 49 | 67 | 66 | 66 | 100 | 51 | 53 | 53 | 53 | 50 | 99 | 100 | 100 | 66 | 53 |
| Rv3447c | - | 1236 | 54 | 54 | 54 | 54 | 49 | 50 | 47 | 58 | 61 | 65 | 44 | 63 | 67 | 72 | 75 | 72 | 99 | 63 | 65 | 64 | 64 | 64 | 99 | 100 | 99 | 75 | 71 |
| Rv3448 | - | 467 | 40 | 0 | 39 | 0 | 0 | 0 | 0 | 42 | 46 | 45 | 0 | 46 | 47 | 59 | 65 | 61 | 99 | 45 | 46 | 46 | 46 | 44 | 100 | 100 | 100 | 64 | 45 |
| Rv3449 | *mycP4* | 455 | 47 | 50 | 44 | 48 | 41 | 42 | 40 | 44 | 57 | 62 | 0 | 58 | 63 | 76 | 75 | 76 | 100 | 65 | 66 | 66 | 66 | 64 | 100 | 100 | 100 | 75 | 68 |
| Rv3450c | - | 470 | 37 | 47 | 39 | 46 | 42 | 54 | 40 | 37 | 52 | 56 | 0 | 55 | 60 | 68 | 70 | 69 | 100 | 60 | 60 | 60 | 60 | 56 | 100 | 100 | 100 | 70 | 60 |
| Rv3451 | *cut3* | 262 | 0 | 0 | 0 | 0 | 0 | 0 | 0 | 0 | 0 | 0 | 50 | 0 | 60 | 73 | 76 | 73 | 100 | 64 | 58 | 57 | 57 | 75 | 99 | 100 | 99 | 75 | 66 |
| Rv3452 | *cut4* | 226 | 0 | 0 | 0 | 0 | 0 | 0 | 0 | 0 | 0 | 0 | 53 | 0 | 57 | 84 | 84 | 84 | 100 | 70 | 65 | 64 | 64 | 67 | 100 | 100 | 100 | 81 | 73 |
| Rv3453 | - | 110 | 0 | 0 | 0 | 0 | 0 | 76 | 0 | 0 | 0 | 80 | 0 | 0 | 85 | 0 | 0 | 0 | 100 | 0 | 0 | 0 | 0 | 0 | 100 | 100 | 100 | 88 | 0 |

| | | | | | | | | | | | | | | | | | | | | | | | | | | | | | |
|---|---|---|---|---|---|---|---|---|---|---|---|---|---|---|---|---|---|---|---|---|---|---|---|---|---|---|---|---|---|
| Rv3454 | - | 422 | 0 | 0 | 0 | 0 | 0 | 75 | 0 | 0 | 0 | 83 | 0 | 0 | 83 | 0 | 0 | 0 | 100 | 0 | 0 | 0 | 0 | 0 | 100 | 100 | 100 | 90 | 0 |
| Rv3455c | *truA* | 297 | 63 | 66 | 65 | 64 | 65 | 64 | 65 | 62 | 68 | 72 | 72 | 71 | 77 | 89 | 84 | 89 | 100 | 81 | 86 | 86 | 86 | 77 | 100 | 100 | 100 | 84 | 85 |
| Rv3456c | *rplQ* | 180 | 73 | 71 | 68 | 73 | 69 | 72 | 72 | 69 | 78 | 78 | 77 | 78 | 85 | 82 | 82 | 82 | 100 | 87 | 83 | 83 | 83 | 82 | 99 | 100 | 100 | 82 | 83 |
| Rv3457c | *rpoA* | 347 | 86 | 86 | 88 | 86 | 87 | 88 | 87 | 84 | 93 | 94 | 94 | 94 | 96 | 99 | 99 | 99 | 100 | 95 | 96 | 96 | 96 | 95 | 100 | 100 | 100 | 99 | 96 |
| Rv3458c | *rpsD* | 201 | 84 | 85 | 85 | 85 | 87 | 85 | 86 | 85 | 93 | 91 | 92 | 92 | 92 | 99 | 97 | 99 | 100 | 94 | 94 | 94 | 94 | 95 | 100 | 100 | 100 | 96 | 94 |
| Rv3459c | *rpsK* | 139 | 84 | 85 | 85 | 87 | 87 | 88 | 86 | 82 | 92 | 92 | 92 | 92 | 93 | 97 | 97 | 97 | 99 | 94 | 97 | 97 | 97 | 95 | 100 | 100 | 100 | 95 | 94 |
| Rv3460c | *rpsM* | 124 | 87 | 88 | 87 | 87 | 87 | 85 | 88 | 90 | 92 | 92 | 92 | 92 | 94 | 99 | 99 | 99 | 100 | 94 | 96 | 96 | 96 | 97 | 100 | 100 | 100 | 99 | 95 |
| Rv3461c | *rpmJ* | 37 | 0 | 0 | 0 | 0 | 0 | 0 | 0 | 94 | 89 | 89 | 89 | 89 | 97 | 100 | 100 | 100 | 100 | 100 | 100 | 100 | 100 | 100 | 100 | 100 | 100 | 100 | 100 |
| Rv3462c | *infA* | 73 | 100 | 100 | 100 | 100 | 100 | 100 | 100 | 93 | 100 | 100 | 100 | 100 | 100 | 100 | 100 | 100 | 100 | 100 | 100 | 100 | 100 | 100 | 100 | 100 | 100 | 100 | 100 |
| Rv3463 | - | 285 | 0 | 0 | 0 | 0 | 0 | 0 | 0 | 0 | 73 | 71 | 72 | 72 | 74 | 90 | 90 | 90 | 100 | 84 | 83 | 83 | 83 | 85 | 100 | 100 | 100 | 88 | 85 |
| Rv3464 | *rmlB* | 331 | 74 | 76 | 75 | 76 | 64 | 67 | 70 | 45 | 77 | 80 | 79 | 79 | 85 | 95 | 96 | 95 | 100 | 85 | 87 | 87 | 87 | 89 | 100 | 100 | 100 | 96 | 87 |
| Rv3465 | *rmlC* | 202 | 46 | 46 | 46 | 45 | 60 | 54 | 59 | 0 | 69 | 73 | 70 | 69 | 70 | 89 | 89 | 89 | 100 | 0 | 80 | 80 | 80 | 77 | 100 | 100 | 100 | 90 | 75 |
| Rv3466 | - | 222 | 0 | 0 | 0 | 0 | 0 | 0 | 0 | 0 | 0 | 0 | 50 | 49 | 60 | 66 | 74 | 66 | 98 | 62 | 56 | 56 | 56 | 52 | 99 | 100 | 99 | 74 | 58 |
| Rv3467 | - | 317 | 0 | 51 | 41 | 45 | 48 | 0 | 0 | 0 | 0 | 38 | 54 | 48 | 68 | 74 | 75 | 74 | 98 | 62 | 62 | 61 | 61 | 56 | 99 | 100 | 100 | 74 | 61 |
| Rv3468c | - | 364 | 0 | 45 | 0 | 0 | 0 | 0 | 0 | 0 | 39 | 0 | 0 | 42 | 66 | 40 | 47 | 41 | 100 | 38 | 42 | 41 | 41 | 48 | 100 | 100 | 100 | 40 | 41 |
| Rv3469c | *mhpE* | 336 | 0 | 0 | 0 | 0 | 0 | 0 | 0 | 50 | 52 | 52 | 53 | 53 | 50 | 50 | 50 | 50 | 100 | 50 | 50 | 50 | 50 | 49 | 100 | 100 | 100 | 51 | 50 |
| Rv3470c | *ilvB2* | 552 | 44 | 44 | 43 | 43 | 44 | 42 | 44 | 44 | 43 | 42 | 42 | 41 | 42 | 43 | 42 | 42 | 99 | 42 | 43 | 43 | 43 | 42 | 100 | 100 | 100 | 43 | 42 |
| Rv3471c | - | 177 | 0 | 0 | 0 | 0 | 0 | 0 | 0 | 0 | 0 | 0 | 0 | 0 | 0 | 0 | 0 | 0 | 99 | 0 | 0 | 0 | 0 | 0 | 100 | 100 | 100 | 0 | 0 |
| Rv3472 | - | 168 | 0 | 0 | 0 | 0 | 0 | 0 | 0 | 0 | 0 | 0 | 0 | 0 | 0 | 0 | 0 | 0 | 100 | 0 | 0 | 0 | 0 | 0 | 100 | 100 | 100 | 0 | 0 |
| Rv3473c | *bpoA* | 261 | 0 | 0 | 0 | 0 | 0 | 0 | 0 | 41 | 56 | 0 | 60 | 58 | 57 | 88 | 89 | 89 | 100 | 83 | 58 | 58 | 58 | 61 | 100 | 100 | 100 | 86 | 86 |
| Rv3474 | - | 108 | 65 | 0 | 66 | 0 | 61 | 0 | 60 | 76 | 0 | 0 | 0 | 0 | 59 | 63 | 82 | 66 | 100 | 82 | 96 | 77 | 77 | 84 | 100 | 100 | 100 | 0 | 62 |
| Rv3475 | - | 346 | 58 | 49 | 59 | 51 | 61 | 0 | 59 | 64 | 0 | 0 | 49 | 52 | 66 | 58 | 65 | 64 | 100 | 65 | 88 | 64 | 64 | 66 | 100 | 100 | 100 | 0 | 61 |
| Rv3476c | *kgtP* | 449 | 48 | 46 | 49 | 50 | 0 | 46 | 48 | 0 | 67 | 54 | 84 | 84 | 90 | 48 | 46 | 48 | 99 | 51 | 82 | 82 | 82 | 90 | 100 | 100 | 100 | 46 | 51 |
| Rv3477 | *PE31* | 98 | 0 | 0 | 0 | 0 | 0 | 0 | 0 | 0 | 0 | 0 | 0 | 0 | 0 | 75 | 77 | 75 | 98 | 0 | 0 | 0 | 0 | 0 | 100 | 100 | 100 | 75 | 0 |
| Rv3478 | *PPE60* | 393 | 0 | 0 | 0 | 0 | 0 | 0 | 0 | 0 | 37 | 40 | 0 | 0 | 51 | 55 | 55 | 55 | 100 | 44 | 43 | 43 | 43 | 49 | 92 | 100 | 100 | 68 | 50 |
| Rv3479 | - | 1021 | 0 | 0 | 0 | 0 | 0 | 0 | 0 | 0 | 0 | 0 | 0 | 0 | 0 | 0 | 0 | 0 | 99 | 0 | 0 | 0 | 0 | 0 | 99 | 99 | 99 | 0 | 0 |
| Rv3480c | - | 497 | 0 | 0 | 0 | 0 | 0 | 0 | 0 | 50 | 53 | 51 | 53 | 52 | 50 | 51 | 95 | 51 | 100 | 75 | 51 | 51 | 51 | 52 | 100 | 100 | 100 | 94 | 73 |
| Rv3481c | - | 229 | 0 | 0 | 0 | 0 | 0 | 0 | 0 | 0 | 0 | 61 | 60 | 58 | 93 | 81 | 82 | 81 | 100 | 72 | 74 | 74 | 74 | 73 | 100 | 100 | 100 | 82 | 73 |
| Rv3482c | - | 260 | 0 | 0 | 0 | 0 | 0 | 0 | 0 | 0 | 0 | 0 | 0 | 0 | 0 | 0 | 72 | 0 | 100 | 0 | 0 | 0 | 0 | 0 | 100 | 100 | 100 | 72 | 0 |
| Rv3483c | - | 220 | 44 | 0 | 0 | 0 | 0 | 0 | 51 | 0 | 0 | 43 | 45 | 0 | 43 | 47 | 73 | 50 | 100 | 43 | 46 | 46 | 46 | 50 | 100 | 100 | 100 | 72 | 46 |
| Rv3484 | *cpsA* | 512 | 48 | 54 | 51 | 50 | 47 | 49 | 47 | 43 | 52 | 57 | 52 | 52 | 71 | 87 | 87 | 87 | 100 | 48 | 50 | 50 | 50 | 53 | 100 | 100 | 100 | 87 | 49 |
| Rv3485c | - | 314 | 47 | 42 | 50 | 48 | 51 | 47 | 50 | 51 | 51 | 75 | 73 | 73 | 50 | 86 | 92 | 87 | 100 | 84 | 87 | 87 | 87 | 86 | 100 | 100 | 100 | 92 | 85 |
| Rv3486 | - | 149 | 0 | 0 | 51 | 50 | 0 | 54 | 0 | 0 | 0 | 0 | 0 | 0 | 0 | 0 | 0 | 0 | 100 | 0 | 67 | 0 | 0 | 0 | 100 | 100 | 100 | 0 | 0 |
| Rv3487c | *lipF* | 277 | 47 | 0 | 0 | 0 | 41 | 0 | 0 | 42 | 57 | 55 | 60 | 60 | 70 | 85 | 83 | 85 | 100 | 77 | 80 | 79 | 79 | 75 | 100 | 100 | 100 | 82 | 76 |
| Rv3488 | - | 107 | 0 | 0 | 0 | 0 | 0 | 0 | 0 | 0 | 0 | 0 | 0 | 0 | 0 | 0 | 0 | 0 | 99 | 0 | 83 | 0 | 0 | 0 | 100 | 100 | 100 | 97 | 0 |
| Rv3489 | - | 54 | 0 | 0 | 0 | 0 | 0 | 0 | 0 | 0 | 76 | 0 | 89 | 89 | 79 | 96 | 96 | 96 | 100 | 84 | 88 | 88 | 88 | 84 | 100 | 100 | 100 | 96 | 86 |
| Rv3490 | *otsA* | 500 | 67 | 65 | 66 | 65 | 67 | 68 | 60 | 70 | 83 | 85 | 87 | 86 | 88 | 89 | 92 | 90 | 100 | 86 | 87 | 87 | 87 | 87 | 100 | 100 | 100 | 92 | 85 |
| Rv3491 | - | 192 | 0 | 0 | 0 | 0 | 0 | 0 | 0 | 0 | 0 | 0 | 0 | 0 | 63 | 90 | 90 | 90 | 100 | 50 | 47 | 47 | 47 | 48 | 100 | 100 | 100 | 89 | 46 |

| | | | | | | | | | | | | | | | | | | | | | | | | | | | | | |
|---|---|---|---|---|---|---|---|---|---|---|---|---|---|---|---|---|---|---|---|---|---|---|---|---|---|---|---|---|---|
| Rv3492c | - | 160 | 0 | 0 | 0 | 0 | 0 | 0 | 0 | 0 | 52 | 53 | 52 | 45 | 70 | 84 | 84 | 84 | 100 | 79 | 74 | 75 | 75 | 77 | 100 | 100 | 100 | 89 | 76 |
| Rv3493c | - | 242 | 0 | 0 | 0 | 0 | 0 | 0 | 0 | 0 | 0 | 0 | 45 | 45 | 61 | 80 | 83 | 80 | 100 | 56 | 72 | 70 | 70 | 70 | 100 | 100 | 100 | 83 | 72 |
| Rv3494c | *mce4F* | 564 | 0 | 0 | 0 | 0 | 39 | 0 | 0 | 45 | 63 | 67 | 57 | 62 | 67 | 90 | 91 | 91 | 99 | 75 | 72 | 72 | 72 | 76 | 100 | 100 | 100 | 91 | 74 |
| Rv3495c | *lprN* | 384 | 0 | 0 | 0 | 0 | 0 | 0 | 0 | 52 | 60 | 59 | 58 | 58 | 73 | 91 | 92 | 91 | 100 | 77 | 79 | 79 | 79 | 78 | 100 | 100 | 100 | 92 | 78 |
| Rv3496c | *mce4D* | 451 | 0 | 0 | 0 | 0 | 0 | 0 | 0 | 53 | 56 | 61 | 60 | 60 | 71 | 85 | 89 | 86 | 100 | 76 | 75 | 77 | 77 | 75 | 100 | 100 | 100 | 88 | 74 |
| Rv3497c | *mce4C* | 357 | 0 | 0 | 0 | 0 | 0 | 0 | 0 | 55 | 62 | 60 | 62 | 62 | 78 | 90 | 92 | 90 | 100 | 81 | 82 | 82 | 82 | 80 | 100 | 100 | 100 | 91 | 82 |
| Rv3498c | *mce4B* | 350 | 0 | 0 | 0 | 0 | 0 | 0 | 0 | 53 | 64 | 65 | 68 | 68 | 82 | 92 | 94 | 92 | 100 | 81 | 84 | 83 | 83 | 85 | 100 | 100 | 100 | 94 | 82 |
| Rv3499c | *mce4A* | 400 | 0 | 0 | 0 | 0 | 0 | 0 | 0 | 47 | 57 | 59 | 57 | 57 | 74 | 91 | 91 | 91 | 100 | 79 | 80 | 79 | 79 | 81 | 100 | 100 | 100 | 91 | 79 |
| Rv3500c | *yrbE4B* | 280 | 0 | 0 | 0 | 0 | 0 | 0 | 0 | 74 | 82 | 84 | 84 | 84 | 90 | 97 | 98 | 97 | 100 | 93 | 94 | 94 | 94 | 95 | 100 | 100 | 100 | 97 | 93 |
| Rv3501c | *yrbE4A* | 254 | 0 | 0 | 0 | 0 | 0 | 0 | 0 | 70 | 89 | 87 | 88 | 88 | 93 | 98 | 100 | 98 | 100 | 96 | 96 | 95 | 95 | 96 | 100 | 100 | 100 | 100 | 96 |
| Rv3502c | *fabG* | 317 | 49 | 0 | 51 | 54 | 49 | 48 | 47 | 71 | 75 | 73 | 75 | 74 | 84 | 90 | 90 | 90 | 100 | 86 | 84 | 83 | 83 | 83 | 100 | 100 | 100 | 90 | 82 |
| Rv3503c | *fdxD* | 63 | 0 | 0 | 0 | 0 | 0 | 0 | 0 | 71 | 72 | 70 | 71 | 71 | 81 | 95 | 95 | 95 | 100 | 90 | 91 | 93 | 93 | 93 | 100 | 100 | 100 | 93 | 88 |
| Rv3504 | *fadE26* | 400 | 42 | 0 | 43 | 0 | 47 | 47 | 47 | 77 | 84 | 87 | 85 | 85 | 90 | 92 | 93 | 92 | 100 | 87 | 90 | 88 | 88 | 89 | 100 | 100 | 100 | 93 | 89 |
| Rv3505 | *fadE27* | 373 | 0 | 0 | 40 | 0 | 43 | 41 | 40 | 59 | 57 | 66 | 66 | 65 | 75 | 88 | 90 | 89 | 99 | 81 | 82 | 84 | 84 | 81 | 100 | 100 | 100 | 89 | 81 |
| Rv3506 | *fadD17* | 502 | 43 | 46 | 43 | 41 | 40 | 36 | 45 | 42 | 61 | 65 | 67 | 67 | 70 | 88 | 88 | 88 | 100 | 75 | 82 | 78 | 78 | 76 | 99 | 100 | 100 | 88 | 76 |
| Rv3507 | *PE_PGRS53* | 1381 | 44 | 46 | 31 | 32 | 32 | 32 | 38 | 30 | 35 | 37 | 37 | 37 | 36 | 30 | 73 | 30 | 86 | 51 | 49 | 49 | 49 | 47 | 98 | 100 | 98 | 53 | 50 |
| Rv3508 | *PE_PGRS54* | 1901 | 46 | 31 | 35 | 33 | 38 | 36 | 41 | 37 | 38 | 38 | 38 | 39 | 38 | 30 | 32 | 32 | 74 | 53 | 51 | 51 | 51 | 46 | 87 | 97 | 87 | 56 | 53 |
| Rv3509c | *ilvX* | 515 | 39 | 0 | 0 | 46 | 38 | 0 | 51 | 39 | 40 | 72 | 44 | 71 | 0 | 88 | 89 | 89 | 100 | 80 | 39 | 39 | 39 | 68 | 100 | 100 | 100 | 90 | 82 |
| Rv3510c | - | 278 | 0 | 0 | 0 | 46 | 0 | 0 | 0 | 0 | 0 | 0 | 47 | 48 | 0 | 91 | 88 | 90 | 100 | 55 | 56 | 56 | 56 | 52 | 100 | 100 | 100 | 88 | 55 |
| Rv3511 | *PE_PGRS55* | 714 | 41 | 41 | 37 | 33 | 33 | 32 | 36 | 35 | 38 | 37 | 42 | 41 | 38 | 44 | 75 | 34 | 99 | 54 | 49 | 49 | 49 | 46 | 99 | 99 | 99 | 57 | 52 |
| Rv3512 | *PE_PGRS56* | 1079 | 42 | 45 | 35 | 31 | 36 | 43 | 37 | 30 | 38 | 38 | 39 | 40 | 41 | 30 | 30 | 30 | 96 | 52 | 54 | 52 | 52 | 47 | 98 | 100 | 97 | 55 | 53 |
| Rv3513c | *fadD18* | 218 | 48 | 52 | 47 | 48 | 56 | 44 | 47 | 74 | 85 | 88 | 87 | 88 | 81 | 94 | 94 | 94 | 100 | 92 | 90 | 89 | 89 | 92 | 100 | 100 | 100 | 94 | 93 |
| Rv3514 | *PE_PGRS57* | 1489 | 46 | 29 | 33 | 33 | 35 | 36 | 38 | 35 | 37 | 38 | 39 | 38 | 39 | 29 | 57 | 32 | 68 | 51 | 53 | 53 | 53 | 45 | 69 | 70 | 68 | 55 | 52 |
| Rv3515c | *fadD19* | 548 | 38 | 37 | 41 | 39 | 42 | 38 | 41 | 68 | 79 | 83 | 83 | 84 | 81 | 95 | 95 | 95 | 100 | 91 | 89 | 89 | 89 | 91 | 100 | 100 | 100 | 95 | 92 |
| Rv3516 | *echA19* | 263 | 51 | 41 | 50 | 42 | 50 | 49 | 46 | 82 | 85 | 85 | 86 | 86 | 86 | 95 | 96 | 95 | 99 | 89 | 93 | 93 | 93 | 91 | 99 | 100 | 99 | 96 | 90 |
| Rv3517 | - | 279 | 0 | 0 | 49 | 43 | 0 | 0 | 0 | 46 | 0 | 46 | 63 | 63 | 70 | 79 | 69 | 79 | 100 | 79 | 79 | 78 | 78 | 75 | 100 | 100 | 100 | 67 | 62 |
| Rv3518c | *cyp142* | 398 | 0 | 0 | 45 | 46 | 45 | 0 | 0 | 55 | 51 | 74 | 76 | 77 | 49 | 88 | 88 | 88 | 100 | 86 | 85 | 85 | 85 | 85 | 100 | 100 | 100 | 94 | 87 |
| Rv3519 | - | 236 | 0 | 0 | 0 | 0 | 0 | 0 | 0 | 0 | 59 | 61 | 62 | 64 | 0 | 82 | 92 | 82 | 100 | 76 | 83 | 83 | 83 | 78 | 97 | 100 | 100 | 92 | 76 |
| Rv3520c | - | 347 | 0 | 0 | 0 | 0 | 0 | 0 | 0 | 77 | 81 | 83 | 84 | 83 | 85 | 93 | 93 | 93 | 100 | 92 | 94 | 94 | 94 | 93 | 100 | 100 | 100 | 93 | 91 |
| Rv3521 | - | 303 | 0 | 0 | 0 | 0 | 0 | 0 | 0 | 70 | 78 | 76 | 76 | 74 | 85 | 90 | 88 | 90 | 100 | 85 | 83 | 83 | 83 | 84 | 100 | 100 | 100 | 88 | 83 |
| Rv3522 | *ltp4* | 354 | 0 | 0 | 0 | 0 | 0 | 0 | 0 | 74 | 80 | 82 | 82 | 82 | 88 | 93 | 92 | 93 | 99 | 87 | 90 | 90 | 90 | 89 | 99 | 100 | 99 | 91 | 89 |
| Rv3523 | *ltp3* | 394 | 0 | 0 | 41 | 0 | 0 | 0 | 0 | 83 | 90 | 87 | 88 | 88 | 93 | 94 | 95 | 94 | 100 | 93 | 94 | 94 | 94 | 94 | 100 | 100 | 99 | 95 | 93 |
| Rv3524 | - | 343 | 0 | 0 | 0 | 0 | 0 | 0 | 0 | 0 | 0 | 0 | 57 | 52 | 0 | 54 | 67 | 54 | 100 | 0 | 0 | 0 | 0 | 0 | 100 | 100 | 100 | 67 | 0 |
| Rv3525c | - | 174 | 63 | 60 | 61 | 59 | 61 | 60 | 62 | 72 | 62 | 60 | 64 | 62 | 80 | 91 | 91 | 91 | 100 | 58 | 57 | 57 | 57 | 60 | 100 | 100 | 100 | 97 | 61 |
| Rv3526 | - | 386 | 0 | 0 | 0 | 0 | 0 | 0 | 0 | 73 | 75 | 80 | 81 | 82 | 86 | 95 | 94 | 96 | 100 | 92 | 93 | 93 | 93 | 90 | 100 | 100 | 100 | 93 | 90 |
| Rv3527 | - | 149 | 0 | 0 | 0 | 0 | 0 | 0 | 0 | 0 | 55 | 56 | 55 | 55 | 70 | 86 | 87 | 86 | 100 | 73 | 77 | 79 | 79 | 77 | 100 | 100 | 100 | 87 | 77 |
| Rv3528c | - | 237 | 0 | 0 | 0 | 0 | 0 | 0 | 0 | 0 | 0 | 0 | 0 | 0 | 0 | 0 | 0 | 0 | 100 | 0 | 0 | 0 | 0 | 0 | 100 | 100 | 100 | 0 | 0 |
| Rv3529c | - | 384 | 0 | 0 | 0 | 0 | 0 | 0 | 0 | 67 | 78 | 77 | 79 | 79 | 83 | 93 | 93 | 93 | 100 | 83 | 83 | 84 | 84 | 84 | 100 | 100 | 100 | 92 | 84 |

| | | | | | | | | | | | | | | | | | | | | | | | | | | | | | |
|---|---|---|---|---|---|---|---|---|---|---|---|---|---|---|---|---|---|---|---|---|---|---|---|---|---|---|---|---|---|
| Rv3530c | - | 260 | 51 | 49 | 47 | 50 | 46 | 48 | 48 | 65 | 71 | 81 | 82 | 82 | 82 | 91 | 91 | 92 | 100 | 88 | 90 | 90 | 90 | 86 | 100 | 100 | 100 | 89 | 89 |
| Rv3531c | - | 375 | 0 | 0 | 0 | 0 | 0 | 0 | 0 | 64 | 75 | 74 | 76 | 76 | 79 | 94 | 94 | 94 | 100 | 87 | 87 | 87 | 87 | 90 | 100 | 100 | 100 | 93 | 88 |
| Rv3532 | *PPE61* | 406 | 0 | 0 | 0 | 0 | 0 | 0 | 0 | 0 | 0 | 0 | 0 | 0 | 40 | 57 | 57 | 57 | 100 | 43 | 41 | 41 | 41 | 54 | 99 | 100 | 100 | 73 | 42 |
| Rv3533c | *PPE62* | 582 | 0 | 0 | 0 | 0 | 0 | 49 | 0 | 0 | 36 | 38 | 37 | 36 | 43 | 66 | 64 | 66 | 100 | 35 | 39 | 39 | 39 | 41 | 100 | 100 | 100 | 66 | 37 |
| Rv3534c | - | 346 | 0 | 0 | 0 | 0 | 0 | 0 | 0 | 64 | 90 | 90 | 90 | 91 | 94 | 97 | 97 | 96 | 100 | 97 | 95 | 96 | 96 | 96 | 100 | 100 | 100 | 97 | 96 |
| Rv3535c | - | 303 | 0 | 0 | 0 | 0 | 0 | 0 | 0 | 70 | 91 | 93 | 93 | 92 | 92 | 96 | 94 | 97 | 100 | 92 | 92 | 92 | 92 | 92 | 100 | 100 | 99 | 94 | 90 |
| Rv3536c | - | 261 | 60 | 0 | 0 | 0 | 0 | 0 | 0 | 57 | 83 | 80 | 80 | 80 | 82 | 90 | 93 | 90 | 100 | 86 | 86 | 86 | 86 | 85 | 100 | 100 | 100 | 93 | 85 |
| Rv3537 | - | 563 | 0 | 0 | 0 | 52 | 0 | 0 | 0 | 69 | 77 | 78 | 78 | 78 | 88 | 94 | 94 | 94 | 100 | 91 | 92 | 92 | 92 | 91 | 100 | 100 | 100 | 93 | 91 |
| Rv3538 | - | 286 | 0 | 0 | 0 | 0 | 0 | 0 | 0 | 67 | 71 | 73 | 75 | 74 | 75 | 90 | 91 | 91 | 100 | 84 | 86 | 86 | 86 | 84 | 99 | 100 | 100 | 90 | 83 |
| Rv3539 | *PPE63* | 479 | 0 | 0 | 0 | 0 | 0 | 0 | 0 | 0 | 0 | 40 | 0 | 0 | 48 | 67 | 58 | 67 | 100 | 46 | 49 | 49 | 49 | 48 | 100 | 100 | 100 | 68 | 51 |
| Rv3540c | *ltp2* | 386 | 0 | 0 | 37 | 0 | 0 | 0 | 0 | 82 | 85 | 87 | 87 | 87 | 89 | 92 | 96 | 92 | 100 | 89 | 90 | 90 | 90 | 89 | 100 | 100 | 100 | 96 | 90 |
| Rv3541c | - | 129 | 0 | 0 | 0 | 0 | 0 | 0 | 0 | 72 | 88 | 86 | 85 | 86 | 85 | 93 | 92 | 93 | 100 | 90 | 91 | 91 | 91 | 87 | 100 | 100 | 100 | 94 | 92 |
| Rv3542c | - | 311 | 0 | 0 | 0 | 0 | 0 | 0 | 0 | 54 | 74 | 77 | 75 | 75 | 80 | 90 | 90 | 90 | 100 | 84 | 84 | 84 | 84 | 83 | 100 | 100 | 100 | 89 | 86 |
| Rv3543c | *fadE29* | 387 | 0 | 0 | 45 | 0 | 46 | 45 | 45 | 77 | 81 | 84 | 84 | 84 | 87 | 93 | 94 | 93 | 100 | 89 | 92 | 92 | 92 | 89 | 99 | 100 | 100 | 93 | 91 |
| Rv3544c | *fadE28* | 339 | 0 | 0 | 0 | 0 | 38 | 0 | 47 | 62 | 60 | 67 | 67 | 66 | 72 | 90 | 87 | 90 | 100 | 83 | 83 | 83 | 83 | 85 | 100 | 100 | 100 | 86 | 81 |
| Rv3545c | *cyp125* | 433 | 0 | 0 | 44 | 46 | 38 | 0 | 0 | 72 | 82 | 82 | 82 | 83 | 87 | 88 | 95 | 88 | 100 | 82 | 83 | 83 | 83 | 83 | 100 | 100 | 99 | 95 | 84 |
| Rv3546 | *fadA5* | 391 | 49 | 0 | 55 | 49 | 54 | 51 | 53 | 78 | 82 | 82 | 84 | 84 | 84 | 91 | 94 | 91 | 100 | 90 | 90 | 90 | 90 | 89 | 100 | 100 | 100 | 93 | 90 |
| Rv3547 | - | 151 | 0 | 0 | 0 | 0 | 0 | 0 | 0 | 56 | 60 | 68 | 70 | 68 | 68 | 86 | 86 | 86 | 100 | 69 | 82 | 82 | 82 | 72 | 100 | 100 | 100 | 93 | 66 |
| Rv3548c | - | 304 | 46 | 0 | 50 | 55 | 50 | 50 | 49 | 74 | 84 | 82 | 83 | 84 | 87 | 94 | 93 | 94 | 100 | 90 | 91 | 91 | 91 | 93 | 100 | 100 | 100 | 92 | 90 |
| Rv3549c | - | 259 | 48 | 47 | 51 | 45 | 49 | 45 | 46 | 72 | 76 | 63 | 76 | 78 | 81 | 85 | 92 | 85 | 100 | 84 | 84 | 85 | 85 | 86 | 100 | 100 | 100 | 92 | 82 |
| Rv3550 | *echA20* | 247 | 47 | 44 | 49 | 52 | 46 | 44 | 45 | 83 | 80 | 83 | 84 | 83 | 87 | 95 | 97 | 95 | 100 | 91 | 90 | 90 | 90 | 93 | 100 | 100 | 100 | 97 | 91 |
| Rv3551 | - | 292 | 0 | 0 | 0 | 0 | 0 | 0 | 0 | 71 | 84 | 84 | 84 | 84 | 87 | 93 | 93 | 93 | 100 | 88 | 87 | 87 | 87 | 88 | 100 | 100 | 100 | 92 | 88 |
| Rv3552 | - | 250 | 0 | 0 | 0 | 0 | 0 | 0 | 0 | 68 | 83 | 82 | 82 | 82 | 86 | 91 | 91 | 91 | 100 | 89 | 89 | 89 | 89 | 90 | 100 | 100 | 100 | 93 | 88 |
| Rv3553 | - | 355 | 50 | 0 | 39 | 0 | 47 | 42 | 44 | 79 | 85 | 82 | 83 | 83 | 91 | 93 | 94 | 94 | 100 | 90 | 93 | 93 | 93 | 91 | 100 | 100 | 100 | 94 | 91 |
| Rv3554 | *fdxB* | 685 | 0 | 44 | 51 | 41 | 0 | 0 | 0 | 61 | 58 | 57 | 72 | 57 | 58 | 46 | 93 | 46 | 100 | 50 | 47 | 47 | 47 | 49 | 100 | 100 | 100 | 92 | 49 |
| Rv3555c | - | 289 | 43 | 0 | 44 | 0 | 0 | 0 | 42 | 47 | 0 | 45 | 57 | 57 | 71 | 70 | 69 | 69 | 99 | 59 | 67 | 67 | 67 | 67 | 94 | 100 | 100 | 60 | 65 |
| Rv3556c | *fadA6* | 386 | 50 | 0 | 60 | 52 | 56 | 53 | 55 | 80 | 84 | 86 | 84 | 85 | 88 | 97 | 96 | 97 | 100 | 92 | 91 | 91 | 91 | 90 | 100 | 100 | 100 | 96 | 91 |
| Rv3557c | - | 200 | 45 | 0 | 45 | 0 | 47 | 0 | 0 | 73 | 77 | 75 | 75 | 76 | 83 | 94 | 94 | 94 | 100 | 88 | 92 | 92 | 92 | 89 | 100 | 100 | 100 | 94 | 92 |
| Rv3558 | *PPE64* | 552 | 0 | 0 | 0 | 0 | 0 | 51 | 0 | 0 | 36 | 34 | 0 | 39 | 42 | 62 | 69 | 62 | 100 | 46 | 48 | 53 | 53 | 52 | 99 | 100 | 99 | 63 | 48 |
| Rv3559c | - | 262 | 48 | 48 | 52 | 52 | 46 | 51 | 47 | 72 | 83 | 84 | 85 | 83 | 90 | 95 | 97 | 95 | 99 | 92 | 91 | 91 | 91 | 90 | 100 | 100 | 100 | 97 | 91 |
| Rv3560c | *fadE30* | 385 | 40 | 0 | 41 | 0 | 43 | 47 | 43 | 77 | 85 | 79 | 80 | 80 | 86 | 95 | 96 | 95 | 100 | 92 | 91 | 91 | 91 | 90 | 100 | 100 | 100 | 95 | 91 |
| Rv3561 | *fadD3* | 507 | 44 | 48 | 47 | 48 | 49 | 45 | 46 | 67 | 72 | 70 | 71 | 71 | 76 | 89 | 90 | 90 | 99 | 84 | 85 | 85 | 85 | 85 | 100 | 100 | 100 | 89 | 82 |
| Rv3562 | *fadE31* | 377 | 0 | 0 | 40 | 0 | 42 | 44 | 42 | 75 | 79 | 79 | 78 | 79 | 84 | 94 | 96 | 94 | 100 | 89 | 86 | 87 | 87 | 89 | 100 | 100 | 100 | 95 | 89 |
| Rv3563 | *fadE32* | 319 | 0 | 0 | 0 | 0 | 48 | 0 | 0 | 62 | 61 | 70 | 68 | 69 | 75 | 86 | 87 | 85 | 99 | 79 | 81 | 81 | 81 | 80 | 100 | 100 | 100 | 86 | 80 |
| Rv3564 | *fadE33* | 318 | 0 | 0 | 0 | 0 | 37 | 0 | 0 | 57 | 57 | 56 | 56 | 57 | 74 | 90 | 91 | 90 | 100 | 81 | 82 | 82 | 82 | 81 | 100 | 100 | 100 | 91 | 82 |
| Rv3565 | *aspB* | 388 | 42 | 64 | 63 | 62 | 41 | 61 | 38 | 76 | 70 | 71 | 69 | 70 | 73 | 92 | 93 | 93 | 100 | 89 | 87 | 87 | 87 | 88 | 99 | 100 | 100 | 92 | 89 |
| Rv3566A | - | 88 | 0 | 0 | 0 | 0 | 0 | 0 | 0 | 0 | 0 | 0 | 0 | 0 | 0 | 0 | 0 | 0 | 100 | 0 | 0 | 0 | 0 | 0 | 100 | 100 | 100 | 0 | 0 |
| Rv3566c | *nat* | 283 | 0 | 0 | 0 | 0 | 0 | 0 | 0 | 0 | 47 | 0 | 57 | 0 | 44 | 77 | 84 | 78 | 100 | 72 | 74 | 73 | 73 | 72 | 100 | 100 | 100 | 84 | 72 |

| | | | | | | | | | | | | | | | | | | | | | | | | | | | | | |
|---|---|---|---|---|---|---|---|---|---|---|---|---|---|---|---|---|---|---|---|---|---|---|---|---|---|---|---|---|---|
| Rv3567c | - | 187 | 0 | 0 | 51 | 55 | 46 | 0 | 0 | 69 | 77 | 85 | 85 | 84 | 88 | 95 | 95 | 95 | 99 | 88 | 92 | 92 | 92 | 91 | 100 | 100 | 100 | 95 | 89 |
| Rv3568c | *bphC* | 300 | 0 | 0 | 0 | 0 | 0 | 0 | 0 | 69 | 88 | 86 | 88 | 89 | 82 | 95 | 96 | 95 | 100 | 91 | 93 | 92 | 92 | 90 | 100 | 100 | 100 | 96 | 90 |
| Rv3569c | *bphD* | 291 | 0 | 0 | 48 | 0 | 0 | 0 | 0 | 67 | 85 | 85 | 84 | 84 | 57 | 92 | 92 | 92 | 100 | 88 | 87 | 87 | 87 | 90 | 100 | 100 | 100 | 93 | 87 |
| Rv3570c | - | 394 | 0 | 0 | 0 | 0 | 0 | 0 | 40 | 70 | 86 | 85 | 87 | 86 | 86 | 95 | 96 | 95 | 100 | 90 | 91 | 91 | 91 | 92 | 100 | 100 | 100 | 96 | 89 |
| Rv3571 | *hmp* | 358 | 0 | 48 | 45 | 44 | 0 | 55 | 0 | 61 | 68 | 71 | 69 | 70 | 79 | 92 | 91 | 92 | 100 | 81 | 81 | 81 | 81 | 81 | 100 | 100 | 100 | 92 | 83 |
| Rv3572 | - | 176 | 0 | 0 | 0 | 0 | 0 | 0 | 0 | 0 | 0 | 0 | 0 | 0 | 55 | 75 | 91 | 76 | 100 | 51 | 54 | 54 | 54 | 46 | 100 | 100 | 100 | 90 | 54 |
| Rv3573c | *fadE34* | 711 | 40 | 0 | 40 | 0 | 43 | 44 | 43 | 62 | 65 | 67 | 68 | 68 | 71 | 88 | 89 | 89 | 100 | 79 | 81 | 81 | 81 | 81 | 99 | 100 | 100 | 88 | 81 |
| Rv3574 | - | 199 | 0 | 0 | 0 | 0 | 0 | 0 | 0 | 72 | 86 | 84 | 84 | 84 | 87 | 98 | 98 | 98 | 100 | 95 | 96 | 96 | 96 | 96 | 99 | 100 | 100 | 98 | 95 |
| Rv3575c | - | 359 | 62 | 61 | 64 | 61 | 60 | 62 | 65 | 50 | 43 | 77 | 77 | 76 | 0 | 94 | 96 | 94 | 100 | 87 | 85 | 86 | 86 | 85 | 100 | 100 | 100 | 95 | 87 |
| Rv3576 | *lppH* | 237 | 0 | 0 | 0 | 0 | 0 | 0 | 0 | 0 | 0 | 0 | 0 | 0 | 0 | 50 | 87 | 50 | 100 | 49 | 52 | 51 | 51 | 49 | 100 | 100 | 100 | 87 | 52 |
| Rv3577 | - | 288 | 0 | 0 | 0 | 0 | 0 | 0 | 0 | 0 | 0 | 0 | 57 | 0 | 0 | 90 | 88 | 90 | 100 | 77 | 77 | 76 | 76 | 80 | 100 | 100 | 100 | 88 | 77 |
| Rv3578 | *arsB2* | 413 | 0 | 0 | 45 | 0 | 0 | 0 | 0 | 0 | 43 | 62 | 63 | 64 | 0 | 84 | 88 | 84 | 100 | 72 | 75 | 75 | 75 | 72 | 100 | 100 | 100 | 87 | 73 |
| Rv3579c | - | 322 | 73 | 72 | 74 | 74 | 74 | 73 | 73 | 72 | 83 | 80 | 82 | 81 | 83 | 93 | 92 | 93 | 99 | 83 | 87 | 87 | 87 | 84 | 100 | 100 | 100 | 92 | 82 |
| Rv3580c | *cysS* | 469 | 74 | 75 | 76 | 75 | 73 | 72 | 74 | 66 | 79 | 81 | 80 | 80 | 88 | 94 | 94 | 95 | 100 | 87 | 89 | 89 | 89 | 88 | 100 | 100 | 100 | 95 | 89 |
| Rv3581c | *ispF* | 159 | 74 | 71 | 69 | 71 | 73 | 0 | 74 | 69 | 81 | 84 | 81 | 81 | 83 | 87 | 94 | 87 | 100 | 86 | 86 | 86 | 86 | 84 | 100 | 100 | 100 | 94 | 87 |
| Rv3582c | *ispD* | 231 | 60 | 58 | 62 | 62 | 50 | 0 | 55 | 0 | 67 | 71 | 69 | 68 | 70 | 84 | 87 | 84 | 99 | 47 | 80 | 79 | 79 | 76 | 100 | 100 | 100 | 86 | 43 |
| Rv3583c | - | 162 | 84 | 82 | 84 | 83 | 83 | 83 | 82 | 86 | 98 | 98 | 98 | 98 | 97 | 100 | 100 | 100 | 100 | 99 | 96 | 96 | 96 | 100 | 100 | 100 | 100 | 100 | 99 |
| Rv3584 | *lpqE* | 182 | 0 | 0 | 0 | 0 | 0 | 0 | 0 | 0 | 43 | 48 | 53 | 51 | 57 | 81 | 86 | 81 | 100 | 68 | 65 | 64 | 64 | 67 | 100 | 100 | 100 | 86 | 68 |
| Rv3585 | *radA* | 480 | 67 | 67 | 67 | 66 | 65 | 0 | 67 | 68 | 77 | 74 | 75 | 74 | 81 | 89 | 86 | 89 | 100 | 83 | 82 | 81 | 81 | 81 | 100 | 100 | 100 | 83 | 79 |
| Rv3586 | - | 358 | 0 | 0 | 73 | 73 | 0 | 0 | 0 | 72 | 0 | 83 | 81 | 81 | 87 | 94 | 94 | 94 | 99 | 92 | 92 | 92 | 92 | 91 | 100 | 100 | 100 | 94 | 92 |
| Rv3587c | - | 264 | 40 | 43 | 43 | 39 | 40 | 45 | 43 | 0 | 51 | 47 | 53 | 52 | 71 | 90 | 90 | 90 | 100 | 75 | 78 | 79 | 79 | 77 | 100 | 100 | 100 | 94 | 76 |
| Rv3588c | - | 207 | 62 | 62 | 71 | 68 | 66 | 69 | 69 | 0 | 80 | 78 | 82 | 81 | 80 | 90 | 94 | 90 | 99 | 88 | 88 | 88 | 88 | 85 | 100 | 100 | 100 | 94 | 89 |
| Rv3589 | *mutY* | 304 | 66 | 64 | 67 | 66 | 55 | 55 | 59 | 64 | 76 | 73 | 71 | 72 | 77 | 85 | 86 | 84 | 100 | 79 | 79 | 79 | 79 | 78 | 100 | 100 | 100 | 86 | 79 |
| Rv3590c | *PE_PGRS58* | 584 | 38 | 43 | 38 | 34 | 34 | 38 | 36 | 35 | 39 | 40 | 40 | 42 | 43 | 35 | 62 | 35 | 100 | 54 | 52 | 54 | 54 | 42 | 100 | 100 | 100 | 57 | 52 |
| Rv3591c | - | 257 | 0 | 0 | 0 | 0 | 0 | 0 | 0 | 36 | 64 | 66 | 66 | 67 | 74 | 88 | 90 | 88 | 100 | 86 | 85 | 86 | 86 | 82 | 100 | 100 | 100 | 90 | 84 |
| Rv3592 | *TB11.2* | 105 | 0 | 73 | 69 | 71 | 0 | 0 | 0 | 79 | 86 | 85 | 84 | 84 | 85 | 95 | 95 | 95 | 100 | 88 | 87 | 87 | 87 | 87 | 100 | 100 | 100 | 94 | 91 |
| Rv3593 | *lpqF* | 452 | 0 | 0 | 0 | 0 | 0 | 0 | 0 | 0 | 0 | 44 | 0 | 0 | 71 | 89 | 89 | 89 | 99 | 75 | 76 | 76 | 76 | 77 | 99 | 100 | 99 | 85 | 75 |
| Rv3594 | - | 275 | 0 | 55 | 0 | 0 | 0 | 0 | 50 | 0 | 50 | 0 | 0 | 0 | 65 | 0 | 0 | 0 | 100 | 67 | 65 | 65 | 65 | 61 | 100 | 100 | 100 | 0 | 66 |
| Rv3595c | *PE_PGRS59* | 439 | 41 | 42 | 39 | 37 | 36 | 39 | 38 | 39 | 40 | 39 | 39 | 44 | 43 | 61 | 70 | 58 | 79 | 47 | 52 | 52 | 52 | 46 | 99 | 100 | 100 | 60 | 50 |
| Rv3596c | *clpC1* | 848 | 88 | 89 | 88 | 88 | 88 | 89 | 89 | 88 | 93 | 95 | 95 | 95 | 95 | 99 | 98 | 99 | 100 | 97 | 97 | 97 | 97 | 97 | 100 | 100 | 100 | 98 | 97 |
| Rv3597c | *lsr2* | 112 | 0 | 0 | 0 | 0 | 50 | 54 | 52 | 61 | 75 | 61 | 75 | 75 | 85 | 97 | 97 | 97 | 100 | 91 | 92 | 92 | 92 | 90 | 100 | 100 | 100 | 99 | 93 |
| Rv3598c | *lysS* | 505 | 75 | 75 | 75 | 75 | 74 | 71 | 76 | 72 | 86 | 84 | 84 | 84 | 85 | 94 | 92 | 94 | 100 | 88 | 89 | 89 | 89 | 90 | 100 | 100 | 100 | 92 | 89 |
| Rv3599c | - | 27 | 0 | 0 | 0 | 0 | 0 | 0 | 0 | 0 | 0 | 0 | 0 | 0 | 0 | 0 | 0 | 0 | 0 | 0 | 0 | 0 | 0 | 0 | 0 | 0 | 0 | 0 | 0 |
| Rv3600c | - | 272 | 0 | 0 | 0 | 0 | 0 | 0 | 0 | 70 | 77 | 84 | 83 | 83 | 88 | 94 | 94 | 94 | 100 | 89 | 92 | 92 | 92 | 92 | 100 | 100 | 100 | 94 | 90 |
| Rv3601c | *panD* | 139 | 0 | 0 | 76 | 77 | 81 | 0 | 80 | 79 | 82 | 83 | 83 | 83 | 84 | 92 | 90 | 92 | 100 | 91 | 89 | 89 | 89 | 78 | 100 | 100 | 100 | 90 | 92 |
| Rv3602c | *panC* | 309 | 59 | 60 | 58 | 59 | 72 | 65 | 69 | 66 | 77 | 79 | 77 | 75 | 77 | 86 | 86 | 86 | 100 | 81 | 81 | 82 | 82 | 81 | 100 | 100 | 100 | 87 | 77 |
| Rv3603c | - | 303 | 0 | 0 | 0 | 0 | 55 | 0 | 55 | 55 | 81 | 78 | 78 | 79 | 81 | 92 | 92 | 92 | 100 | 85 | 85 | 85 | 85 | 88 | 100 | 100 | 100 | 93 | 85 |
| Rv3604c | - | 397 | 37 | 44 | 0 | 0 | 0 | 47 | 47 | 0 | 44 | 45 | 47 | 51 | 48 | 62 | 62 | 62 | 99 | 54 | 53 | 54 | 54 | 46 | 99 | 100 | 100 | 64 | 55 |

| | | | | | | | | | | | | | | | | | | | | | | | | | | | | | |
|---|---|---|---|---|---|---|---|---|---|---|---|---|---|---|---|---|---|---|---|---|---|---|---|---|---|---|---|---|---|
| Rv3605c | - | 158 | 49 | 50 | 55 | 51 | 48 | 52 | 50 | 0 | 61 | 67 | 63 | 64 | 71 | 90 | 85 | 90 | 100 | 76 | 75 | 75 | 75 | 77 | 99 | 100 | 100 | 85 | 76 |
| Rv3606c | *folK* | 188 | 60 | 62 | 56 | 60 | 51 | 52 | 51 | 52 | 64 | 69 | 64 | 64 | 69 | 82 | 74 | 82 | 100 | 66 | 74 | 73 | 73 | 74 | 100 | 100 | 100 | 74 | 70 |
| Rv3607c | *folB* | 133 | 70 | 68 | 71 | 71 | 65 | 66 | 63 | 56 | 67 | 72 | 76 | 76 | 73 | 86 | 87 | 86 | 100 | 78 | 75 | 82 | 82 | 82 | 100 | 100 | 100 | 85 | 75 |
| Rv3608c | *folP1* | 280 | 63 | 66 | 65 | 66 | 61 | 60 | 62 | 66 | 77 | 76 | 73 | 74 | 76 | 90 | 90 | 90 | 100 | 82 | 84 | 84 | 84 | 85 | 100 | 100 | 100 | 85 | 83 |
| Rv3609c | *folE* | 202 | 84 | 87 | 87 | 85 | 84 | 85 | 84 | 81 | 90 | 92 | 93 | 93 | 93 | 96 | 93 | 96 | 100 | 91 | 92 | 92 | 92 | 95 | 100 | 100 | 100 | 93 | 90 |
| Rv3610c | *ftsH* | 760 | 76 | 72 | 73 | 73 | 75 | 69 | 70 | 66 | 80 | 79 | 81 | 81 | 86 | 89 | 92 | 89 | 100 | 86 | 88 | 88 | 88 | 88 | 100 | 100 | 100 | 92 | 89 |
| Rv3611 | - | 217 | 0 | 0 | 0 | 0 | 0 | 0 | 37 | 38 | 41 | 0 | 40 | 39 | 0 | 38 | 42 | 42 | 100 | 39 | 0 | 0 | 0 | 37 | 100 | 97 | 100 | 36 | 41 |
| Rv3612c | - | 109 | 0 | 0 | 0 | 0 | 0 | 0 | 0 | 0 | 0 | 0 | 0 | 0 | 0 | 0 | 0 | 0 | 100 | 0 | 0 | 0 | 0 | 0 | 100 | 100 | 100 | 0 | 0 |
| Rv3613c | - | 53 | 0 | 0 | 0 | 0 | 0 | 0 | 0 | 0 | 0 | 0 | 0 | 0 | 0 | 0 | 0 | 0 | 100 | 0 | 0 | 0 | 0 | 0 | 100 | 100 | 100 | 0 | 0 |
| Rv3614c | - | 184 | 0 | 0 | 0 | 0 | 0 | 0 | 0 | 0 | 0 | 0 | 0 | 0 | 0 | 0 | 74 | 0 | 100 | 62 | 57 | 54 | 54 | 54 | 100 | 100 | 100 | 70 | 55 |
| Rv3615c | - | 103 | 0 | 0 | 0 | 0 | 0 | 0 | 0 | 0 | 0 | 0 | 0 | 0 | 0 | 0 | 79 | 0 | 100 | 0 | 0 | 0 | 0 | 0 | 100 | 100 | 100 | 0 | 0 |
| Rv3616c | - | 392 | 0 | 0 | 0 | 0 | 0 | 0 | 0 | 0 | 0 | 0 | 0 | 0 | 0 | 0 | 77 | 0 | 99 | 0 | 0 | 0 | 0 | 0 | 99 | 100 | 99 | 0 | 0 |
| Rv3617 | *ephA* | 322 | 52 | 52 | 55 | 53 | 40 | 39 | 38 | 42 | 53 | 60 | 63 | 61 | 76 | 89 | 91 | 89 | 48 | 72 | 76 | 77 | 77 | 81 | 100 | 100 | 100 | 91 | 75 |
| Rv3618 | - | 395 | 54 | 0 | 0 | 0 | 51 | 0 | 48 | 0 | 41 | 62 | 59 | 59 | 84 | 92 | 93 | 92 | 48 | 66 | 93 | 92 | 92 | 90 | 100 | 100 | 100 | 89 | 66 |
| Rv3619c | *esxV* | 94 | 0 | 0 | 0 | 0 | 0 | 0 | 0 | 0 | 0 | 0 | 0 | 0 | 0 | 91 | 93 | 91 | 98 | 0 | 0 | 0 | 0 | 0 | 100 | 100 | 100 | 93 | 0 |
| Rv3620c | *esxW* | 98 | 0 | 0 | 0 | 0 | 0 | 0 | 0 | 0 | 0 | 0 | 0 | 0 | 0 | 92 | 92 | 92 | 98 | 0 | 0 | 0 | 0 | 0 | 100 | 100 | 100 | 95 | 0 |
| Rv3621c | *PPE65* | 413 | 0 | 0 | 0 | 0 | 0 | 0 | 35 | 0 | 36 | 0 | 34 | 35 | 41 | 66 | 83 | 69 | 59 | 53 | 43 | 43 | 43 | 43 | 100 | 100 | 100 | 64 | 44 |
| Rv3622c | *PE32* | 99 | 0 | 0 | 0 | 0 | 0 | 0 | 0 | 0 | 0 | 0 | 0 | 0 | 0 | 73 | 88 | 76 | 76 | 0 | 0 | 0 | 0 | 0 | 100 | 100 | 100 | 81 | 0 |
| Rv3623 | *lpqG* | 240 | 0 | 0 | 0 | 0 | 0 | 0 | 0 | 46 | 50 | 64 | 48 | 48 | 62 | 79 | 79 | 79 | 96 | 70 | 75 | 75 | 75 | 74 | 100 | 100 | 100 | 78 | 73 |
| Rv3624c | *hpt* | 216 | 75 | 75 | 81 | 80 | 79 | 79 | 73 | 77 | 80 | 81 | 82 | 82 | 78 | 92 | 89 | 92 | 100 | 88 | 83 | 83 | 83 | 80 | 100 | 100 | 100 | 89 | 87 |
| Rv3625c | *mesJ* | 323 | 53 | 56 | 53 | 53 | 54 | 52 | 52 | 54 | 66 | 66 | 64 | 64 | 72 | 81 | 85 | 82 | 100 | 74 | 75 | 74 | 74 | 75 | 100 | 100 | 100 | 84 | 78 |
| Rv3626c | - | 350 | 0 | 0 | 0 | 0 | 0 | 0 | 0 | 58 | 69 | 69 | 73 | 72 | 79 | 85 | 88 | 85 | 100 | 79 | 81 | 80 | 80 | 80 | 100 | 100 | 100 | 88 | 80 |
| Rv3627c | - | 461 | 51 | 50 | 52 | 50 | 55 | 51 | 51 | 47 | 59 | 64 | 62 | 62 | 73 | 89 | 88 | 89 | 99 | 73 | 73 | 73 | 73 | 74 | 99 | 100 | 100 | 88 | 73 |
| Rv3628 | *ppa* | 162 | 78 | 82 | 79 | 79 | 79 | 79 | 80 | 76 | 86 | 88 | 90 | 91 | 92 | 92 | 93 | 92 | 100 | 87 | 85 | 85 | 85 | 88 | 100 | 100 | 100 | 93 | 87 |
| Rv3629c | - | 365 | 0 | 0 | 0 | 0 | 0 | 0 | 0 | 0 | 77 | 75 | 75 | 74 | 0 | 85 | 86 | 85 | 100 | 0 | 0 | 0 | 0 | 0 | 100 | 100 | 100 | 86 | 0 |
| Rv3630 | - | 431 | 0 | 0 | 55 | 53 | 54 | 0 | 53 | 0 | 0 | 0 | 0 | 0 | 74 | 88 | 89 | 88 | 99 | 76 | 82 | 83 | 83 | 82 | 99 | 100 | 99 | 89 | 80 |
| Rv3631 | - | 241 | 44 | 0 | 73 | 76 | 44 | 0 | 0 | 42 | 0 | 0 | 46 | 42 | 77 | 91 | 91 | 91 | 100 | 41 | 42 | 42 | 42 | 44 | 100 | 100 | 100 | 91 | 0 |
| Rv3632 | - | 114 | 0 | 0 | 81 | 81 | 0 | 0 | 0 | 0 | 0 | 0 | 0 | 0 | 89 | 93 | 93 | 93 | 100 | 0 | 0 | 0 | 0 | 0 | 100 | 100 | 100 | 97 | 0 |
| Rv3633 | - | 291 | 0 | 0 | 0 | 0 | 0 | 0 | 0 | 0 | 52 | 0 | 49 | 53 | 57 | 55 | 55 | 55 | 100 | 0 | 0 | 0 | 0 | 0 | 100 | 100 | 100 | 50 | 0 |
| Rv3634c | *galE1* | 314 | 45 | 46 | 75 | 74 | 45 | 46 | 47 | 42 | 47 | 48 | 48 | 48 | 88 | 94 | 93 | 94 | 100 | 46 | 52 | 52 | 52 | 84 | 100 | 100 | 100 | 93 | 44 |
| Rv3635 | - | 591 | 0 | 0 | 0 | 0 | 44 | 0 | 43 | 43 | 60 | 59 | 60 | 61 | 73 | 86 | 86 | 86 | 100 | 82 | 80 | 80 | 80 | 80 | 100 | 100 | 100 | 86 | 82 |
| Rv3636 | - | 115 | 0 | 0 | 0 | 0 | 0 | 0 | 0 | 0 | 0 | 0 | 54 | 59 | 0 | 0 | 0 | 0 | 100 | 72 | 72 | 0 | 0 | 0 | 100 | 100 | 99 | 0 | 72 |
| Rv3637 | - | 166 | 0 | 0 | 0 | 0 | 0 | 0 | 0 | 0 | 0 | 0 | 0 | 0 | 0 | 0 | 0 | 0 | 100 | 69 | 0 | 0 | 0 | 0 | 100 | 100 | 100 | 0 | 69 |
| Rv3638 | - | 248 | 0 | 0 | 52 | 0 | 0 | 0 | 0 | 45 | 0 | 47 | 45 | 0 | 0 | 68 | 53 | 53 | 100 | 81 | 0 | 0 | 0 | 53 | 100 | 100 | 100 | 0 | 78 |
| Rv3639c | - | 188 | 0 | 0 | 0 | 0 | 0 | 0 | 0 | 0 | 0 | 0 | 0 | 0 | 0 | 0 | 0 | 0 | 99 | 0 | 0 | 0 | 0 | 0 | 100 | 100 | 100 | 0 | 0 |
| Rv3640c | - | 409 | 48 | 58 | 59 | 0 | 63 | 0 | 55 | 86 | 0 | 44 | 63 | 71 | 0 | 73 | 0 | 72 | 99 | 72 | 45 | 62 | 62 | 72 | 99 | 100 | 100 | 46 | 74 |
| Rv3641c | *fic* | 211 | 0 | 0 | 0 | 0 | 0 | 0 | 0 | 0 | 0 | 46 | 0 | 0 | 0 | 0 | 0 | 0 | 100 | 0 | 0 | 0 | 0 | 43 | 100 | 100 | 100 | 91 | 85 |
| Rv3642c | - | 64 | 0 | 0 | 0 | 0 | 0 | 0 | 0 | 0 | 0 | 0 | 0 | 0 | 0 | 0 | 0 | 86 | 100 | 0 | 0 | 0 | 0 | 0 | 100 | 100 | 100 | 92 | 81 |

| | | | | | | | | | | | | | | | | | | | | | | | | | | | | |
|---|---|---|---|---|---|---|---|---|---|---|---|---|---|---|---|---|---|---|---|---|---|---|---|---|---|---|---|---|---|
| Rv3643 | - | 63 | 0 | 0 | 0 | 0 | 0 | 0 | 0 | 0 | 0 | 0 | 0 | 0 | 0 | 0 | 0 | 0 | 100 | 0 | 0 | 0 | 0 | 0 | 91 | 100 | 100 | 74 | 0 |
| Rv3644c | - | 401 | 55 | 60 | 59 | 58 | 62 | 66 | 57 | 58 | 78 | 74 | 79 | 79 | 82 | 93 | 93 | 93 | 100 | 88 | 90 | 90 | 90 | 89 | 100 | 100 | 100 | 91 | 87 |
| Rv3645 | - | 549 | 62 | 62 | 61 | 61 | 61 | 60 | 62 | 0 | 61 | 62 | 62 | 62 | 79 | 90 | 92 | 90 | 100 | 83 | 86 | 86 | 86 | 86 | 100 | 100 | 100 | 92 | 85 |
| Rv3646c | *topA* | 934 | 73 | 74 | 75 | 74 | 75 | 74 | 74 | 72 | 84 | 81 | 81 | 81 | 86 | 92 | 92 | 92 | 100 | 87 | 87 | 87 | 87 | 89 | 100 | 100 | 100 | 91 | 86 |
| Rv3647c | - | 192 | 0 | 0 | 0 | 0 | 0 | 0 | 0 | 0 | 0 | 72 | 74 | 75 | 83 | 86 | 91 | 86 | 100 | 84 | 86 | 86 | 86 | 83 | 100 | 100 | 100 | 91 | 85 |
| Rv3648c | *cspA* | 67 | 82 | 89 | 86 | 85 | 89 | 89 | 88 | 80 | 94 | 92 | 94 | 94 | 97 | 100 | 100 | 100 | 100 | 98 | 98 | 98 | 98 | 98 | 100 | 100 | 100 | 100 | 98 |
| Rv3649 | - | 771 | 66 | 69 | 67 | 68 | 67 | 65 | 66 | 66 | 72 | 75 | 75 | 76 | 79 | 90 | 90 | 90 | 99 | 85 | 86 | 86 | 86 | 86 | 99 | 100 | 100 | 82 | 86 |
| Rv3650 | *PE33* | 94 | 0 | 0 | 0 | 0 | 0 | 0 | 0 | 0 | 0 | 0 | 0 | 0 | 0 | 75 | 81 | 75 | 100 | 0 | 0 | 0 | 0 | 0 | 100 | 100 | 100 | 80 | 0 |
| Rv3651 | - | 345 | 0 | 0 | 0 | 0 | 0 | 0 | 0 | 0 | 0 | 0 | 0 | 0 | 52 | 87 | 88 | 87 | 100 | 70 | 76 | 76 | 76 | 74 | 100 | 100 | 100 | 87 | 70 |
| Rv3652 | *PE_PGRS60* | 104 | 0 | 0 | 0 | 0 | 0 | 0 | 0 | 0 | 0 | 0 | 0 | 0 | 0 | 77 | 88 | 77 | 100 | 0 | 0 | 0 | 0 | 0 | 100 | 100 | 100 | 88 | 0 |
| Rv3653 | *PE_PGRS61* | 195 | 45 | 47 | 36 | 38 | 41 | 0 | 45 | 39 | 50 | 38 | 45 | 44 | 52 | 48 | 62 | 45 | 82 | 56 | 55 | 54 | 54 | 57 | 97 | 100 | 100 | 63 | 57 |
| Rv3654c | - | 84 | 0 | 0 | 0 | 0 | 0 | 0 | 0 | 0 | 58 | 61 | 59 | 60 | 60 | 72 | 71 | 74 | 100 | 64 | 63 | 65 | 65 | 73 | 85 | 100 | 100 | 64 | 66 |
| Rv3655c | - | 125 | 0 | 0 | 0 | 0 | 0 | 0 | 0 | 48 | 69 | 72 | 74 | 61 | 72 | 80 | 82 | 81 | 100 | 64 | 77 | 77 | 77 | 73 | 100 | 100 | 100 | 80 | 78 |
| Rv3656c | - | 68 | 0 | 0 | 0 | 0 | 81 | 0 | 84 | 0 | 77 | 74 | 85 | 80 | 82 | 83 | 91 | 83 | 100 | 84 | 89 | 89 | 89 | 87 | 100 | 100 | 100 | 91 | 81 |
| Rv3657c | - | 191 | 52 | 52 | 46 | 52 | 48 | 55 | 52 | 44 | 69 | 70 | 70 | 68 | 64 | 77 | 73 | 77 | 100 | 75 | 75 | 76 | 76 | 71 | 100 | 100 | 100 | 72 | 77 |
| Rv3658c | - | 266 | 46 | 53 | 49 | 49 | 50 | 48 | 43 | 43 | 56 | 55 | 55 | 54 | 62 | 80 | 75 | 79 | 100 | 67 | 73 | 72 | 72 | 67 | 100 | 100 | 100 | 75 | 66 |
| Rv3659c | - | 352 | 72 | 68 | 72 | 70 | 60 | 63 | 64 | 69 | 78 | 82 | 79 | 81 | 84 | 93 | 89 | 95 | 100 | 82 | 86 | 86 | 86 | 86 | 100 | 100 | 100 | 88 | 86 |
| Rv3660c | - | 350 | 48 | 47 | 45 | 48 | 0 | 49 | 0 | 48 | 57 | 58 | 58 | 58 | 64 | 83 | 83 | 83 | 99 | 71 | 73 | 73 | 73 | 71 | 100 | 100 | 99 | 82 | 69 |
| Rv3661 | - | 287 | 58 | 58 | 63 | 63 | 57 | 0 | 55 | 74 | 78 | 83 | 82 | 84 | 87 | 91 | 92 | 91 | 99 | 86 | 88 | 93 | 93 | 86 | 100 | 100 | 100 | 92 | 90 |
| Rv3662c | - | 256 | 43 | 42 | 0 | 0 | 59 | 0 | 58 | 51 | 75 | 72 | 73 | 73 | 72 | 84 | 84 | 84 | 99 | 76 | 77 | 77 | 77 | 77 | 100 | 100 | 100 | 84 | 76 |
| Rv3663c | *dppD* | 548 | 72 | 74 | 74 | 74 | 58 | 76 | 63 | 61 | 66 | 74 | 75 | 75 | 63 | 85 | 85 | 84 | 100 | 61 | 61 | 61 | 61 | 61 | 100 | 100 | 100 | 85 | 61 |
| Rv3664c | *dppC* | 266 | 74 | 75 | 74 | 74 | 0 | 76 | 53 | 51 | 54 | 81 | 81 | 82 | 66 | 93 | 93 | 94 | 100 | 55 | 55 | 55 | 55 | 56 | 100 | 100 | 100 | 92 | 56 |
| Rv3665c | *dppB* | 308 | 77 | 77 | 78 | 79 | 0 | 80 | 51 | 50 | 49 | 82 | 83 | 83 | 73 | 96 | 95 | 95 | 100 | 56 | 58 | 58 | 58 | 59 | 100 | 100 | 100 | 95 | 57 |
| Rv3666c | *dppA* | 541 | 63 | 64 | 63 | 65 | 0 | 60 | 0 | 0 | 0 | 68 | 68 | 68 | 51 | 90 | 90 | 90 | 99 | 39 | 38 | 38 | 38 | 0 | 100 | 100 | 100 | 91 | 38 |
| Rv3667 | *acs* | 651 | 41 | 42 | 41 | 39 | 40 | 40 | 42 | 77 | 79 | 81 | 82 | 81 | 84 | 88 | 91 | 88 | 99 | 83 | 86 | 86 | 86 | 87 | 100 | 100 | 100 | 91 | 84 |
| Rv3668c | - | 232 | 55 | 53 | 52 | 50 | 56 | 50 | 53 | 0 | 55 | 57 | 58 | 58 | 72 | 87 | 92 | 87 | 100 | 82 | 79 | 79 | 79 | 75 | 100 | 100 | 100 | 91 | 80 |
| Rv3669 | - | 172 | 64 | 68 | 67 | 65 | 68 | 62 | 66 | 0 | 74 | 80 | 81 | 82 | 74 | 83 | 87 | 83 | 100 | 82 | 86 | 86 | 86 | 86 | 100 | 100 | 100 | 86 | 82 |
| Rv3670 | *ephE* | 327 | 50 | 43 | 50 | 45 | 50 | 45 | 51 | 40 | 68 | 71 | 70 | 70 | 80 | 88 | 90 | 88 | 100 | 85 | 82 | 81 | 81 | 79 | 100 | 100 | 100 | 90 | 84 |
| Rv3671c | - | 397 | 59 | 56 | 59 | 60 | 59 | 57 | 62 | 54 | 71 | 74 | 74 | 74 | 79 | 92 | 93 | 92 | 100 | 77 | 81 | 81 | 81 | 79 | 100 | 100 | 100 | 92 | 78 |
| Rv3672c | - | 273 | 49 | 53 | 57 | 53 | 54 | 50 | 59 | 52 | 60 | 64 | 58 | 57 | 67 | 85 | 87 | 85 | 100 | 78 | 81 | 81 | 81 | 79 | 100 | 100 | 100 | 85 | 82 |
| Rv3673c | - | 227 | 46 | 46 | 46 | 47 | 45 | 46 | 49 | 43 | 59 | 58 | 57 | 58 | 69 | 78 | 79 | 78 | 100 | 69 | 73 | 73 | 73 | 74 | 100 | 100 | 100 | 79 | 72 |
| Rv3674c | *nth* | 245 | 70 | 76 | 79 | 79 | 80 | 71 | 77 | 75 | 88 | 85 | 88 | 88 | 88 | 91 | 93 | 90 | 100 | 90 | 90 | 90 | 90 | 90 | 100 | 100 | 100 | 94 | 91 |
| Rv3675 | - | 125 | 0 | 0 | 0 | 0 | 0 | 0 | 0 | 0 | 0 | 0 | 0 | 0 | 73 | 63 | 66 | 63 | 100 | 0 | 49 | 49 | 49 | 0 | 100 | 100 | 100 | 66 | 52 |
| Rv3676 | - | 224 | 87 | 87 | 87 | 87 | 89 | 84 | 85 | 71 | 95 | 97 | 97 | 97 | 100 | 99 | 99 | 99 | 100 | 99 | 100 | 100 | 100 | 100 | 100 | 100 | 100 | 99 | 100 |
| Rv3677c | - | 264 | 60 | 59 | 61 | 59 | 60 | 60 | 58 | 59 | 74 | 73 | 74 | 74 | 82 | 87 | 89 | 88 | 100 | 84 | 87 | 87 | 87 | 82 | 100 | 100 | 100 | 89 | 83 |
| Rv3678A | - | 53 | 75 | 85 | 76 | 78 | 91 | 95 | 93 | 72 | 95 | 77 | 94 | 92 | 96 | 100 | 96 | 100 | 100 | 94 | 98 | 98 | 98 | 98 | 100 | 100 | 100 | 98 | 90 |
| Rv3678c | - | 151 | 78 | 80 | 84 | 85 | 78 | 80 | 75 | 78 | 82 | 82 | 82 | 82 | 86 | 92 | 92 | 92 | 100 | 83 | 90 | 90 | 90 | 84 | 100 | 100 | 100 | 95 | 87 |
| Rv3679 | - | 340 | 0 | 0 | 0 | 0 | 0 | 0 | 0 | 66 | 79 | 79 | 82 | 82 | 85 | 95 | 96 | 95 | 100 | 87 | 90 | 90 | 90 | 87 | 100 | 100 | 100 | 95 | 87 |

| | | | | | | | | | | | | | | | | | | | | | | | | | | | | | |
|---|---|---|---|---|---|---|---|---|---|---|---|---|---|---|---|---|---|---|---|---|---|---|---|---|---|---|---|---|---|
| Rv3680 | - | 386 | 41 | 0 | 0 | 0 | 0 | 46 | 0 | 68 | 82 | 85 | 83 | 83 | 88 | 94 | 92 | 94 | 100 | 91 | 90 | 89 | 89 | 90 | 100 | 100 | 100 | 92 | 91 |
| Rv3681c | *whiB4* | 118 | 85 | 80 | 71 | 70 | 74 | 71 | 74 | 69 | 88 | 88 | 81 | 81 | 76 | 94 | 94 | 94 | 100 | 83 | 81 | 81 | 81 | 79 | 100 | 100 | 100 | 90 | 84 |
| Rv3682 | *ponA2* | 810 | 62 | 65 | 64 | 65 | 65 | 59 | 66 | 48 | 75 | 75 | 76 | 76 | 84 | 94 | 94 | 94 | 100 | 90 | 89 | 89 | 89 | 90 | 100 | 100 | 100 | 96 | 90 |
| Rv3683 | - | 319 | 66 | 65 | 66 | 64 | 63 | 66 | 63 | 66 | 80 | 84 | 80 | 79 | 80 | 93 | 93 | 93 | 100 | 85 | 86 | 86 | 86 | 88 | 100 | 100 | 100 | 95 | 88 |
| Rv3684 | - | 346 | 46 | 42 | 43 | 44 | 43 | 47 | 45 | 43 | 82 | 82 | 82 | 83 | 86 | 93 | 94 | 93 | 100 | 92 | 89 | 89 | 89 | 90 | 100 | 100 | 100 | 93 | 89 |
| Rv3685c | *cyp137* | 476 | 0 | 0 | 42 | 0 | 46 | 0 | 0 | 53 | 50 | 43 | 43 | 42 | 50 | 49 | 88 | 48 | 100 | 52 | 51 | 51 | 51 | 49 | 100 | 100 | 100 | 89 | 70 |
| Rv3686c | - | 110 | 0 | 0 | 0 | 0 | 0 | 0 | 0 | 0 | 0 | 0 | 0 | 0 | 0 | 0 | 0 | 0 | 100 | 0 | 0 | 0 | 0 | 0 | 100 | 100 | 100 | 0 | 0 |
| Rv3687c | *rsfB* | 122 | 0 | 0 | 0 | 0 | 0 | 0 | 0 | 0 | 61 | 0 | 0 | 0 | 0 | 84 | 85 | 84 | 99 | 62 | 56 | 56 | 56 | 61 | 100 | 100 | 100 | 0 | 64 |
| Rv3688c | - | 154 | 68 | 73 | 72 | 71 | 0 | 68 | 0 | 67 | 85 | 88 | 89 | 89 | 0 | 94 | 94 | 94 | 100 | 0 | 94 | 94 | 94 | 94 | 100 | 100 | 100 | 94 | 0 |
| Rv3689 | - | 451 | 0 | 0 | 0 | 0 | 0 | 0 | 0 | 51 | 51 | 0 | 41 | 42 | 56 | 68 | 79 | 67 | 100 | 65 | 64 | 64 | 64 | 67 | 100 | 100 | 100 | 78 | 62 |
| Rv3690 | - | 217 | 0 | 0 | 0 | 0 | 0 | 0 | 0 | 46 | 0 | 0 | 0 | 0 | 70 | 78 | 78 | 78 | 100 | 68 | 77 | 77 | 77 | 73 | 100 | 100 | 100 | 90 | 72 |
| Rv3691 | - | 333 | 0 | 0 | 0 | 0 | 0 | 0 | 0 | 47 | 51 | 0 | 0 | 0 | 64 | 79 | 79 | 79 | 100 | 72 | 71 | 71 | 71 | 70 | 100 | 100 | 100 | 83 | 70 |
| Rv3692 | *moxR2* | 358 | 0 | 42 | 0 | 0 | 0 | 0 | 0 | 75 | 79 | 57 | 64 | 64 | 89 | 92 | 89 | 93 | 100 | 92 | 92 | 92 | 92 | 93 | 100 | 100 | 100 | 89 | 94 |
| Rv3693 | - | 440 | 0 | 0 | 0 | 0 | 0 | 0 | 0 | 66 | 60 | 38 | 39 | 40 | 77 | 90 | 92 | 89 | 100 | 82 | 83 | 83 | 83 | 85 | 100 | 100 | 100 | 92 | 83 |
| Rv3694c | - | 330 | 0 | 0 | 0 | 0 | 0 | 0 | 0 | 66 | 62 | 0 | 0 | 0 | 78 | 90 | 92 | 90 | 100 | 84 | 86 | 86 | 86 | 85 | 100 | 100 | 100 | 92 | 84 |
| Rv3695 | - | 310 | 0 | 0 | 0 | 0 | 0 | 0 | 0 | 60 | 56 | 0 | 0 | 0 | 73 | 82 | 82 | 82 | 100 | 75 | 78 | 77 | 77 | 74 | 100 | 100 | 100 | 82 | 73 |
| Rv3696c | *glpK* | 517 | 69 | 70 | 67 | 67 | 68 | 69 | 68 | 60 | 71 | 75 | 74 | 74 | 90 | 92 | 96 | 93 | 98 | 91 | 91 | 91 | 91 | 91 | 100 | 100 | 100 | 95 | 91 |
| Rv3697c | - | 145 | 0 | 0 | 0 | 0 | 0 | 0 | 0 | 0 | 0 | 0 | 0 | 0 | 0 | 0 | 0 | 0 | 99 | 51 | 0 | 0 | 0 | 0 | 100 | 100 | 100 | 0 | 0 |
| Rv3698 | - | 509 | 0 | 0 | 64 | 63 | 62 | 0 | 0 | 68 | 0 | 69 | 72 | 72 | 83 | 88 | 88 | 87 | 99 | 86 | 86 | 86 | 86 | 85 | 100 | 100 | 100 | 85 | 88 |
| Rv3699 | - | 233 | 0 | 0 | 0 | 0 | 0 | 0 | 0 | 0 | 0 | 0 | 72 | 72 | 72 | 84 | 89 | 84 | 100 | 76 | 81 | 81 | 81 | 80 | 100 | 100 | 99 | 88 | 77 |
| Rv3700c | - | 390 | 40 | 40 | 40 | 0 | 0 | 0 | 0 | 41 | 42 | 44 | 44 | 44 | 65 | 83 | 85 | 83 | 100 | 70 | 76 | 76 | 76 | 75 | 100 | 100 | 100 | 85 | 71 |
| Rv3701c | - | 321 | 0 | 0 | 0 | 0 | 0 | 0 | 0 | 66 | 62 | 65 | 64 | 64 | 77 | 88 | 88 | 88 | 100 | 82 | 80 | 80 | 80 | 81 | 100 | 100 | 100 | 90 | 83 |
| Rv3702c | - | 233 | 0 | 0 | 0 | 0 | 0 | 0 | 0 | 0 | 51 | 51 | 53 | 54 | 78 | 87 | 86 | 87 | 100 | 83 | 79 | 79 | 79 | 82 | 99 | 100 | 100 | 86 | 83 |
| Rv3703c | - | 425 | 0 | 0 | 38 | 0 | 0 | 0 | 0 | 42 | 66 | 64 | 64 | 64 | 78 | 88 | 84 | 88 | 100 | 83 | 83 | 83 | 83 | 84 | 99 | 100 | 100 | 84 | 83 |
| Rv3704c | *gshA* | 432 | 0 | 0 | 0 | 0 | 0 | 0 | 0 | 0 | 45 | 46 | 47 | 47 | 61 | 81 | 80 | 80 | 100 | 78 | 77 | 77 | 77 | 76 | 100 | 100 | 100 | 84 | 78 |
| Rv3705A | - | 129 | 0 | 0 | 0 | 0 | 0 | 0 | 0 | 0 | 0 | 0 | 0 | 0 | 0 | 53 | 63 | 53 | 100 | 53 | 46 | 46 | 46 | 53 | 100 | 100 | 100 | 64 | 48 |
| Rv3705c | - | 214 | 0 | 0 | 0 | 0 | 0 | 0 | 0 | 0 | 0 | 0 | 0 | 0 | 75 | 89 | 85 | 89 | 100 | 81 | 80 | 80 | 80 | 81 | 100 | 100 | 100 | 85 | 83 |
| Rv3706c | - | 106 | 0 | 0 | 0 | 0 | 0 | 0 | 0 | 0 | 0 | 0 | 0 | 0 | 0 | 60 | 74 | 60 | 100 | 51 | 50 | 50 | 50 | 59 | 100 | 100 | 100 | 73 | 54 |
| Rv3707c | - | 336 | 0 | 42 | 44 | 0 | 0 | 0 | 0 | 0 | 0 | 0 | 42 | 41 | 75 | 90 | 93 | 90 | 100 | 82 | 84 | 85 | 85 | 83 | 100 | 100 | 100 | 92 | 83 |
| Rv3708c | *asd* | 345 | 79 | 77 | 79 | 79 | 75 | 77 | 75 | 77 | 89 | 87 | 87 | 87 | 88 | 93 | 93 | 93 | 100 | 90 | 92 | 92 | 92 | 91 | 100 | 100 | 100 | 91 | 90 |
| Rv3709c | *ask* | 421 | 86 | 86 | 87 | 86 | 87 | 81 | 86 | 81 | 90 | 91 | 92 | 92 | 93 | 97 | 97 | 97 | 100 | 94 | 95 | 95 | 95 | 95 | 100 | 100 | 99 | 97 | 94 |
| Rv3710 | *leuA* | 604 | 72 | 74 | 73 | 73 | 74 | 73 | 74 | 68 | 84 | 83 | 84 | 84 | 85 | 88 | 89 | 88 | 99 | 85 | 85 | 85 | 85 | 83 | 100 | 100 | 100 | 89 | 83 |
| Rv3711c | *dnaQ* | 329 | 45 | 42 | 43 | 46 | 48 | 45 | 41 | 51 | 48 | 42 | 54 | 50 | 76 | 89 | 87 | 89 | 99 | 80 | 77 | 77 | 77 | 76 | 100 | 100 | 99 | 86 | 77 |
| Rv3712 | - | 413 | 72 | 72 | 71 | 69 | 69 | 70 | 65 | 38 | 81 | 81 | 81 | 81 | 82 | 90 | 91 | 90 | 99 | 89 | 89 | 89 | 89 | 88 | 100 | 100 | 100 | 90 | 88 |
| Rv3713 | *cobQ2* | 231 | 68 | 69 | 65 | 67 | 57 | 64 | 54 | 0 | 83 | 84 | 83 | 84 | 84 | 93 | 93 | 93 | 100 | 87 | 90 | 91 | 91 | 91 | 100 | 100 | 100 | 93 | 86 |
| Rv3714c | - | 296 | 0 | 0 | 43 | 0 | 0 | 0 | 0 | 44 | 0 | 49 | 56 | 56 | 70 | 73 | 73 | 65 | 100 | 70 | 68 | 67 | 67 | 73 | 100 | 100 | 100 | 72 | 68 |
| Rv3715c | *recR* | 203 | 75 | 74 | 76 | 75 | 65 | 74 | 74 | 83 | 91 | 92 | 92 | 92 | 91 | 98 | 96 | 98 | 100 | 95 | 95 | 95 | 95 | 95 | 100 | 100 | 100 | 96 | 95 |
| Rv3716c | - | 133 | 70 | 77 | 73 | 75 | 78 | 75 | 78 | 56 | 69 | 77 | 76 | 75 | 69 | 87 | 90 | 87 | 100 | 81 | 78 | 78 | 78 | 80 | 100 | 100 | 100 | 90 | 80 |

| | | | | | | | | | | | | | | | | | | | | | | | | | | | | | |
|---|---|---|---|---|---|---|---|---|---|---|---|---|---|---|---|---|---|---|---|---|---|---|---|---|---|---|---|---|---|
| Rv3717 | - | 241 | 0 | 0 | 0 | 0 | 0 | 0 | 0 | 52 | 60 | 70 | 58 | 59 | 78 | 90 | 95 | 90 | 100 | 83 | 83 | 83 | 83 | 87 | 100 | 100 | 100 | 95 | 85 |
| Rv3718c | - | 147 | 0 | 0 | 0 | 0 | 0 | 0 | 0 | 0 | 76 | 76 | 78 | 79 | 86 | 94 | 94 | 94 | 100 | 89 | 90 | 90 | 90 | 88 | 100 | 100 | 100 | 91 | 87 |
| Rv3719 | - | 470 | 67 | 0 | 0 | 71 | 0 | 70 | 70 | 70 | 78 | 80 | 78 | 79 | 84 | 88 | 90 | 88 | 99 | 87 | 87 | 86 | 86 | 85 | 99 | 100 | 99 | 89 | 85 |
| Rv3720 | - | 420 | 68 | 0 | 0 | 67 | 0 | 67 | 63 | 76 | 80 | 83 | 83 | 82 | 86 | 92 | 91 | 92 | 100 | 86 | 85 | 85 | 85 | 87 | 100 | 100 | 99 | 88 | 88 |
| Rv3721c | *dnaZX* | 578 | 73 | 68 | 62 | 63 | 65 | 83 | 80 | 61 | 69 | 72 | 85 | 79 | 73 | 84 | 80 | 84 | 100 | 74 | 77 | 78 | 78 | 85 | 100 | 100 | 100 | 81 | 77 |
| Rv3722c | - | 435 | 69 | 70 | 72 | 72 | 70 | 74 | 69 | 71 | 78 | 78 | 80 | 80 | 84 | 92 | 94 | 92 | 100 | 85 | 88 | 88 | 88 | 84 | 100 | 100 | 100 | 94 | 86 |
| Rv3723 | - | 254 | 0 | 0 | 0 | 0 | 0 | 0 | 0 | 0 | 0 | 0 | 0 | 0 | 60 | 74 | 73 | 73 | 100 | 70 | 69 | 72 | 72 | 64 | 100 | 100 | 100 | 77 | 71 |
| Rv3724A | *cut5a* | 80 | 0 | 0 | 0 | 0 | 0 | 0 | 0 | 0 | 0 | 0 | 0 | 0 | 61 | 69 | 69 | 69 | 100 | 66 | 65 | 65 | 65 | 66 | 100 | 100 | 100 | 70 | 66 |
| Rv3724B | *cut5b* | 187 | 0 | 0 | 0 | 0 | 0 | 0 | 0 | 0 | 0 | 0 | 69 | 0 | 61 | 76 | 83 | 77 | 100 | 70 | 73 | 73 | 73 | 65 | 100 | 100 | 100 | 83 | 74 |
| Rv3725 | - | 309 | 0 | 0 | 0 | 0 | 0 | 0 | 0 | 0 | 56 | 0 | 74 | 76 | 83 | 92 | 89 | 80 | 100 | 79 | 76 | 76 | 76 | 53 | 100 | 100 | 100 | 89 | 80 |
| Rv3726 | - | 397 | 0 | 41 | 0 | 0 | 0 | 0 | 0 | 45 | 42 | 45 | 74 | 44 | 54 | 55 | 57 | 55 | 99 | 75 | 73 | 73 | 73 | 53 | 100 | 100 | 100 | 40 | 76 |
| Rv3727 | - | 602 | 0 | 0 | 0 | 0 | 0 | 0 | 0 | 0 | 42 | 0 | 0 | 0 | 0 | 0 | 79 | 0 | 100 | 0 | 40 | 40 | 40 | 0 | 100 | 100 | 99 | 0 | 0 |
| Rv3728 | - | 1065 | 51 | 48 | 51 | 50 | 48 | 48 | 45 | 50 | 50 | 54 | 59 | 50 | 50 | 49 | 48 | 48 | 99 | 49 | 50 | 50 | 50 | 50 | 100 | 100 | 99 | 76 | 50 |
| Rv3729 | - | 776 | 0 | 0 | 0 | 0 | 0 | 0 | 0 | 46 | 44 | 55 | 0 | 0 | 46 | 44 | 0 | 44 | 99 | 0 | 45 | 45 | 45 | 46 | 100 | 100 | 100 | 0 | 52 |
| Rv3730c | - | 346 | 0 | 0 | 0 | 0 | 0 | 0 | 0 | 66 | 62 | 79 | 83 | 82 | 86 | 93 | 92 | 93 | 100 | 86 | 87 | 87 | 87 | 89 | 100 | 100 | 100 | 92 | 87 |
| Rv3731 | *ligC* | 358 | 0 | 0 | 0 | 0 | 0 | 0 | 0 | 69 | 67 | 63 | 66 | 66 | 88 | 92 | 91 | 92 | 99 | 83 | 83 | 83 | 83 | 83 | 99 | 100 | 99 | 89 | 84 |
| Rv3732 | - | 352 | 0 | 0 | 0 | 0 | 0 | 0 | 0 | 0 | 60 | 0 | 0 | 0 | 0 | 0 | 77 | 0 | 100 | 0 | 0 | 0 | 0 | 0 | 100 | 100 | 100 | 77 | 59 |
| Rv3733c | - | 166 | 0 | 0 | 0 | 0 | 0 | 0 | 0 | 0 | 66 | 0 | 60 | 63 | 0 | 86 | 88 | 86 | 100 | 75 | 78 | 78 | 78 | 79 | 100 | 100 | 100 | 90 | 80 |
| Rv3734c | - | 454 | 0 | 0 | 0 | 0 | 0 | 0 | 0 | 61 | 74 | 67 | 73 | 74 | 67 | 84 | 91 | 84 | 100 | 57 | 80 | 80 | 80 | 80 | 100 | 100 | 100 | 89 | 56 |
| Rv3735 | - | 162 | 0 | 0 | 0 | 0 | 0 | 0 | 0 | 0 | 0 | 0 | 85 | 85 | 0 | 0 | 93 | 0 | 100 | 0 | 0 | 0 | 0 | 0 | 100 | 100 | 100 | 92 | 89 |
| Rv3736 | - | 353 | 0 | 0 | 0 | 0 | 0 | 0 | 0 | 0 | 51 | 44 | 47 | 48 | 66 | 0 | 85 | 0 | 100 | 0 | 39 | 39 | 39 | 0 | 100 | 100 | 100 | 85 | 0 |
| Rv3737 | - | 529 | 49 | 53 | 55 | 53 | 51 | 51 | 52 | 51 | 53 | 57 | 56 | 56 | 0 | 0 | 83 | 0 | 99 | 80 | 82 | 83 | 83 | 82 | 99 | 100 | 99 | 83 | 80 |
| Rv3738c | *PPE66* | 315 | 0 | 0 | 0 | 0 | 0 | 0 | 0 | 0 | 0 | 0 | 0 | 0 | 40 | 47 | 68 | 46 | 56 | 50 | 42 | 42 | 42 | 48 | 100 | 100 | 100 | 78 | 50 |
| Rv3739c | *PPE67* | 77 | 0 | 0 | 0 | 0 | 0 | 0 | 0 | 0 | 0 | 0 | 0 | 0 | 78 | 84 | 85 | 88 | 89 | 81 | 80 | 80 | 80 | 85 | 100 | 100 | 100 | 85 | 85 |
| Rv3740c | - | 448 | 0 | 0 | 0 | 0 | 0 | 0 | 0 | 61 | 72 | 64 | 72 | 73 | 64 | 78 | 88 | 78 | 99 | 55 | 75 | 75 | 75 | 76 | 100 | 100 | 100 | 88 | 54 |
| Rv3741c | - | 224 | 0 | 0 | 0 | 0 | 56 | 0 | 0 | 66 | 65 | 66 | 68 | 69 | 70 | 55 | 65 | 55 | 100 | 64 | 49 | 63 | 63 | 62 | 99 | 100 | 100 | 65 | 65 |
| Rv3742c | - | 131 | 0 | 0 | 0 | 0 | 57 | 0 | 0 | 65 | 61 | 60 | 64 | 65 | 61 | 58 | 76 | 58 | 100 | 61 | 61 | 60 | 60 | 53 | 100 | 100 | 100 | 76 | 58 |
| Rv3743c | *ctpJ* | 660 | 51 | 48 | 55 | 56 | 51 | 64 | 53 | 57 | 77 | 54 | 51 | 51 | 55 | 50 | 69 | 49 | 100 | 55 | 54 | 55 | 55 | 71 | 100 | 100 | 100 | 69 | 74 |
| Rv3744 | - | 120 | 0 | 0 | 0 | 0 | 0 | 0 | 0 | 60 | 89 | 60 | 61 | 54 | 59 | 67 | 60 | 67 | 100 | 58 | 51 | 51 | 51 | 85 | 100 | 100 | 100 | 60 | 60 |
| Rv3745c | - | 70 | 0 | 0 | 0 | 0 | 0 | 0 | 0 | 73 | 84 | 0 | 0 | 0 | 0 | 87 | 82 | 87 | 100 | 81 | 87 | 87 | 87 | 80 | 100 | 100 | 100 | 82 | 82 |
| Rv3746c | *PE34* | 111 | 0 | 0 | 0 | 0 | 0 | 0 | 0 | 0 | 0 | 0 | 0 | 0 | 0 | 0 | 71 | 0 | 100 | 0 | 58 | 58 | 58 | 59 | 100 | 100 | 100 | 71 | 50 |
| Rv3747 | - | 127 | 0 | 0 | 0 | 0 | 0 | 0 | 0 | 0 | 0 | 0 | 0 | 0 | 0 | 0 | 86 | 0 | 100 | 0 | 0 | 0 | 0 | 0 | 100 | 100 | 100 | 84 | 0 |
| Rv3748 | - | 119 | 0 | 0 | 0 | 0 | 0 | 0 | 0 | 0 | 0 | 0 | 0 | 0 | 0 | 0 | 87 | 0 | 99 | 0 | 0 | 0 | 0 | 0 | 100 | 100 | 100 | 87 | 0 |
| Rv3749c | - | 169 | 0 | 0 | 0 | 0 | 0 | 0 | 0 | 0 | 60 | 0 | 59 | 60 | 0 | 0 | 0 | 0 | 100 | 0 | 0 | 0 | 0 | 0 | 100 | 100 | 100 | 0 | 0 |
| Rv3750c | - | 130 | 61 | 62 | 0 | 0 | 0 | 0 | 0 | 0 | 67 | 0 | 65 | 66 | 0 | 0 | 0 | 0 | 100 | 0 | 0 | 0 | 0 | 0 | 100 | 100 | 100 | 57 | 0 |
| Rv3751 | - | 71 | 0 | 75 | 0 | 0 | 0 | 76 | 0 | 70 | 79 | 0 | 0 | 82 | 0 | 71 | 0 | 0 | 100 | 65 | 66 | 75 | 75 | 0 | 100 | 100 | 100 | 0 | 0 |
| Rv3752c | - | 152 | 67 | 67 | 70 | 68 | 71 | 69 | 71 | 72 | 82 | 82 | 83 | 83 | 84 | 96 | 94 | 95 | 100 | 89 | 92 | 92 | 92 | 87 | 100 | 100 | 100 | 94 | 86 |
| Rv3753c | - | 166 | 58 | 63 | 63 | 62 | 67 | 67 | 57 | 56 | 85 | 83 | 80 | 79 | 84 | 98 | 96 | 98 | 100 | 90 | 91 | 91 | 91 | 92 | 100 | 100 | 100 | 96 | 90 |

| | | | | | | | | | | | | | | | | | | | | | | | | | | | | | |
|---|---|---|---|---|---|---|---|---|---|---|---|---|---|---|---|---|---|---|---|---|---|---|---|---|---|---|---|---|---|
| Rv3754 | *tyrA* | 301 | 54 | 64 | 54 | 62 | 53 | 61 | 58 | 52 | 62 | 66 | 62 | 63 | 73 | 88 | 88 | 88 | 100 | 80 | 83 | 83 | 83 | 77 | 100 | 100 | 100 | 89 | 80 |
| Rv3755c | - | 199 | 0 | 0 | 0 | 0 | 0 | 0 | 0 | 0 | 67 | 74 | 74 | 74 | 81 | 86 | 86 | 86 | 100 | 82 | 84 | 84 | 84 | 83 | 100 | 100 | 100 | 93 | 82 |
| Rv3756c | *proZ* | 239 | 0 | 66 | 63 | 50 | 0 | 66 | 0 | 53 | 56 | 77 | 78 | 77 | 81 | 92 | 87 | 92 | 100 | 85 | 83 | 83 | 83 | 86 | 100 | 100 | 100 | 86 | 82 |
| Rv3757c | *proW* | 229 | 0 | 63 | 57 | 0 | 0 | 59 | 0 | 60 | 49 | 71 | 72 | 75 | 84 | 84 | 88 | 84 | 100 | 80 | 83 | 83 | 83 | 80 | 100 | 100 | 100 | 88 | 83 |
| Rv3758c | *proV* | 376 | 53 | 61 | 63 | 55 | 57 | 66 | 55 | 63 | 58 | 74 | 73 | 72 | 75 | 86 | 87 | 85 | 100 | 78 | 77 | 78 | 78 | 78 | 100 | 100 | 100 | 93 | 78 |
| Rv3759c | *proX* | 315 | 0 | 52 | 56 | 0 | 0 | 57 | 0 | 47 | 42 | 66 | 65 | 66 | 74 | 85 | 88 | 85 | 99 | 73 | 77 | 77 | 77 | 74 | 99 | 100 | 100 | 89 | 76 |
| Rv3760 | - | 100 | 58 | 62 | 57 | 55 | 60 | 56 | 63 | 0 | 63 | 65 | 54 | 67 | 68 | 70 | 84 | 71 | 100 | 75 | 76 | 76 | 76 | 78 | 100 | 100 | 100 | 84 | 76 |
| Rv3761c | *fadE36* | 351 | 0 | 0 | 0 | 0 | 48 | 0 | 49 | 58 | 59 | 72 | 76 | 75 | 49 | 90 | 93 | 90 | 100 | 77 | 79 | 79 | 79 | 81 | 100 | 100 | 100 | 91 | 79 |
| Rv3762c | - | 626 | 0 | 0 | 0 | 0 | 0 | 0 | 0 | 0 | 65 | 73 | 71 | 71 | 70 | 87 | 87 | 87 | 99 | 85 | 86 | 85 | 85 | 85 | 100 | 100 | 100 | 87 | 84 |
| Rv3763 | *lpqH* | 159 | 0 | 0 | 0 | 0 | 0 | 0 | 0 | 0 | 44 | 0 | 45 | 45 | 66 | 86 | 90 | 86 | 100 | 69 | 73 | 73 | 73 | 65 | 99 | 100 | 100 | 90 | 73 |
| Rv3764c | - | 475 | 54 | 59 | 58 | 53 | 56 | 52 | 57 | 57 | 55 | 57 | 59 | 58 | 65 | 89 | 88 | 89 | 99 | 69 | 71 | 71 | 71 | 68 | 99 | 100 | 99 | 90 | 68 |
| Rv3765c | - | 234 | 72 | 72 | 73 | 72 | 70 | 71 | 70 | 82 | 73 | 72 | 74 | 74 | 87 | 95 | 96 | 95 | 100 | 91 | 92 | 92 | 92 | 94 | 100 | 100 | 100 | 96 | 93 |
| Rv3766 | - | 229 | 57 | 0 | 0 | 0 | 0 | 0 | 0 | 0 | 0 | 0 | 0 | 0 | 0 | 0 | 65 | 0 | 100 | 0 | 0 | 0 | 0 | 0 | 99 | 100 | 100 | 0 | 0 |
| Rv3767c | - | 314 | 0 | 0 | 0 | 0 | 0 | 0 | 0 | 0 | 50 | 55 | 58 | 0 | 65 | 88 | 83 | 88 | 100 | 65 | 61 | 62 | 62 | 61 | 100 | 100 | 100 | 83 | 62 |
| Rv3768 | - | 119 | 0 | 0 | 0 | 0 | 0 | 0 | 0 | 0 | 0 | 0 | 0 | 0 | 0 | 0 | 84 | 0 | 100 | 0 | 0 | 0 | 0 | 0 | 100 | 100 | 100 | 83 | 0 |
| Rv3769 | - | 90 | 0 | 0 | 0 | 0 | 0 | 0 | 0 | 0 | 0 | 0 | 0 | 0 | 0 | 0 | 0 | 0 | 100 | 68 | 0 | 0 | 0 | 0 | 100 | 100 | 100 | 0 | 0 |
| Rv3770A | - | 60 | 0 | 0 | 0 | 0 | 0 | 0 | 0 | 0 | 0 | 0 | 0 | 0 | 0 | 0 | 0 | 0 | 100 | 0 | 0 | 0 | 0 | 0 | 100 | 100 | 100 | 0 | 0 |
| Rv3770B | - | 63 | 0 | 0 | 0 | 0 | 0 | 0 | 0 | 0 | 0 | 0 | 0 | 0 | 0 | 0 | 0 | 0 | 100 | 0 | 0 | 0 | 0 | 0 | 100 | 100 | 100 | 0 | 78 |
| Rv3770c | - | 191 | 0 | 0 | 0 | 0 | 0 | 0 | 0 | 0 | 0 | 0 | 0 | 0 | 0 | 0 | 0 | 0 | 99 | 0 | 0 | 0 | 0 | 0 | 100 | 100 | 100 | 0 | 0 |
| Rv3771c | - | 108 | 0 | 0 | 0 | 0 | 0 | 0 | 0 | 0 | 0 | 0 | 0 | 0 | 0 | 0 | 0 | 0 | 100 | 0 | 0 | 0 | 0 | 0 | 100 | 100 | 100 | 0 | 0 |
| Rv3772 | *hisC2* | 353 | 65 | 67 | 66 | 66 | 63 | 58 | 59 | 60 | 74 | 72 | 73 | 72 | 83 | 87 | 88 | 87 | 99 | 84 | 83 | 84 | 84 | 84 | 100 | 100 | 100 | 88 | 87 |
| Rv3773c | - | 194 | 0 | 0 | 0 | 0 | 0 | 0 | 0 | 0 | 0 | 0 | 60 | 60 | 61 | 73 | 72 | 72 | 100 | 0 | 71 | 71 | 71 | 57 | 100 | 100 | 100 | 74 | 0 |
| Rv3774 | *echA21* | 274 | 46 | 42 | 46 | 0 | 47 | 45 | 51 | 46 | 81 | 84 | 83 | 81 | 82 | 89 | 95 | 89 | 100 | 87 | 87 | 87 | 87 | 87 | 100 | 100 | 100 | 95 | 86 |
| Rv3775 | *lipE* | 415 | 0 | 0 | 0 | 0 | 0 | 0 | 0 | 0 | 60 | 44 | 42 | 40 | 85 | 90 | 92 | 89 | 100 | 79 | 87 | 87 | 87 | 81 | 100 | 100 | 100 | 92 | 79 |
| Rv3776 | - | 519 | 43 | 56 | 54 | 48 | 54 | 0 | 0 | 41 | 0 | 41 | 36 | 44 | 49 | 56 | 55 | 56 | 100 | 63 | 64 | 64 | 64 | 61 | 100 | 100 | 100 | 56 | 54 |
| Rv3777 | - | 328 | 69 | 68 | 67 | 67 | 64 | 62 | 64 | 72 | 70 | 75 | 73 | 73 | 75 | 87 | 92 | 87 | 99 | 80 | 80 | 80 | 80 | 81 | 99 | 100 | 100 | 92 | 78 |
| Rv3778c | - | 398 | 51 | 52 | 52 | 51 | 55 | 56 | 54 | 51 | 74 | 74 | 75 | 75 | 84 | 93 | 94 | 93 | 100 | 88 | 90 | 90 | 90 | 89 | 100 | 100 | 100 | 94 | 89 |
| Rv3779 | - | 666 | 0 | 0 | 41 | 42 | 0 | 0 | 0 | 0 | 0 | 0 | 0 | 0 | 65 | 84 | 87 | 86 | 100 | 0 | 0 | 0 | 0 | 0 | 99 | 100 | 100 | 87 | 0 |
| Rv3780 | - | 178 | 0 | 0 | 0 | 0 | 0 | 0 | 0 | 85 | 83 | 75 | 72 | 82 | 86 | 92 | 92 | 92 | 100 | 85 | 83 | 83 | 83 | 82 | 100 | 100 | 100 | 94 | 87 |
| Rv3781 | *rfbE* | 273 | 89 | 88 | 87 | 86 | 87 | 86 | 87 | 55 | 88 | 90 | 89 | 89 | 92 | 96 | 96 | 97 | 100 | 92 | 94 | 94 | 94 | 94 | 100 | 100 | 100 | 95 | 93 |
| Rv3782 | - | 304 | 69 | 73 | 71 | 71 | 72 | 73 | 68 | 46 | 81 | 80 | 82 | 82 | 85 | 92 | 89 | 92 | 100 | 86 | 85 | 85 | 85 | 85 | 100 | 100 | 100 | 89 | 86 |
| Rv3783 | *rfbD* | 280 | 74 | 73 | 73 | 74 | 70 | 74 | 72 | 0 | 77 | 76 | 75 | 75 | 82 | 93 | 93 | 93 | 100 | 88 | 89 | 89 | 89 | 88 | 100 | 100 | 100 | 93 | 89 |
| Rv3784 | - | 326 | 50 | 51 | 50 | 50 | 43 | 46 | 50 | 43 | 50 | 51 | 51 | 50 | 52 | 50 | 91 | 50 | 100 | 49 | 50 | 50 | 50 | 50 | 100 | 100 | 100 | 50 | 50 |
| Rv3785 | - | 357 | 0 | 0 | 0 | 0 | 0 | 0 | 0 | 0 | 0 | 0 | 0 | 0 | 0 | 0 | 72 | 0 | 100 | 0 | 0 | 0 | 0 | 0 | 100 | 100 | 100 | 76 | 0 |
| Rv3786c | - | 407 | 52 | 0 | 48 | 55 | 0 | 0 | 0 | 0 | 52 | 0 | 0 | 52 | 48 | 51 | 91 | 51 | 100 | 0 | 49 | 0 | 0 | 52 | 100 | 100 | 99 | 92 | 46 |
| Rv3787c | - | 308 | 0 | 0 | 0 | 0 | 0 | 0 | 0 | 0 | 48 | 56 | 52 | 0 | 61 | 73 | 73 | 73 | 100 | 67 | 69 | 69 | 69 | 68 | 100 | 100 | 100 | 72 | 68 |
| Rv3788 | - | 161 | 0 | 0 | 0 | 0 | 0 | 0 | 0 | 0 | 0 | 0 | 0 | 0 | 0 | 87 | 91 | 87 | 100 | 0 | 0 | 0 | 0 | 0 | 100 | 100 | 100 | 90 | 0 |
| Rv3789 | - | 121 | 58 | 64 | 68 | 75 | 67 | 66 | 68 | 0 | 77 | 80 | 85 | 84 | 85 | 92 | 89 | 93 | 100 | 82 | 80 | 80 | 80 | 82 | 100 | 100 | 100 | 88 | 81 |

| | | | | | | | | | | | | | | | | | | | | | | | | | | | | | |
|---|---|---|---|---|---|---|---|---|---|---|---|---|---|---|---|---|---|---|---|---|---|---|---|---|---|---|---|---|---|
| Rv3790 | - | 461 | 77 | 78 | 77 | 77 | 80 | 77 | 78 | 0 | 81 | 84 | 84 | 84 | 79 | 83 | 92 | 83 | 100 | 89 | 90 | 90 | 90 | 90 | 100 | 100 | 100 | 91 | 88 |
| Rv3791 | - | 254 | 70 | 71 | 71 | 71 | 73 | 73 | 71 | 0 | 85 | 84 | 90 | 90 | 83 | 93 | 93 | 93 | 100 | 85 | 92 | 92 | 92 | 92 | 100 | 100 | 100 | 95 | 85 |
| Rv3792 | - | 643 | 50 | 52 | 51 | 51 | 50 | 52 | 52 | 0 | 62 | 68 | 68 | 68 | 73 | 85 | 85 | 74 | 100 | 78 | 78 | 78 | 78 | 79 | 100 | 100 | 100 | 84 | 76 |
| Rv3793 | *embC* | 1094 | 55 | 54 | 58 | 59 | 55 | 54 | 54 | 0 | 64 | 65 | 67 | 66 | 80 | 91 | 92 | 91 | 99 | 82 | 84 | 84 | 84 | 84 | 99 | 100 | 100 | 91 | 82 |
| Rv3794 | *embA* | 1094 | 53 | 52 | 53 | 56 | 53 | 50 | 51 | 0 | 61 | 63 | 63 | 63 | 77 | 89 | 91 | 89 | 100 | 78 | 78 | 77 | 77 | 79 | 100 | 100 | 100 | 90 | 79 |
| Rv3795 | *embB* | 1098 | 53 | 53 | 55 | 55 | 53 | 50 | 53 | 0 | 63 | 66 | 65 | 65 | 79 | 88 | 92 | 87 | 99 | 80 | 82 | 82 | 82 | 80 | 100 | 100 | 100 | 92 | 81 |
| Rv3796 | - | 375 | 0 | 0 | 0 | 0 | 0 | 0 | 0 | 50 | 0 | 0 | 60 | 58 | 0 | 39 | 87 | 39 | 99 | 40 | 0 | 0 | 0 | 37 | 100 | 100 | 100 | 83 | 39 |
| Rv3797 | *fadE35* | 593 | 42 | 0 | 46 | 39 | 46 | 46 | 49 | 45 | 48 | 49 | 47 | 48 | 49 | 88 | 88 | 89 | 100 | 48 | 86 | 86 | 86 | 48 | 100 | 100 | 100 | 88 | 47 |
| Rv3798 | - | 444 | 0 | 0 | 0 | 0 | 0 | 0 | 0 | 65 | 0 | 0 | 58 | 59 | 0 | 0 | 0 | 0 | 98 | 48 | 0 | 0 | 0 | 46 | 100 | 100 | 100 | 0 | 0 |
| Rv3799c | *accD4* | 522 | 73 | 72 | 73 | 73 | 73 | 65 | 72 | 66 | 84 | 79 | 82 | 82 | 85 | 92 | 92 | 92 | 100 | 90 | 93 | 93 | 93 | 90 | 100 | 100 | 100 | 94 | 90 |
| Rv3800c | *pks13* | 1733 | 57 | 57 | 58 | 58 | 60 | 0 | 59 | 42 | 68 | 69 | 69 | 69 | 80 | 87 | 89 | 87 | 99 | 81 | 80 | 79 | 79 | 79 | 100 | 100 | 100 | 89 | 78 |
| Rv3801c | *fadD32* | 637 | 57 | 59 | 59 | 57 | 58 | 37 | 56 | 37 | 75 | 76 | 77 | 77 | 80 | 91 | 91 | 91 | 99 | 85 | 86 | 86 | 86 | 86 | 99 | 100 | 100 | 96 | 85 |
| Rv3802c | - | 336 | 54 | 55 | 55 | 56 | 58 | 54 | 52 | 0 | 64 | 63 | 56 | 56 | 75 | 85 | 92 | 85 | 100 | 80 | 80 | 80 | 80 | 78 | 100 | 100 | 99 | 92 | 81 |
| Rv3803c | *fbpD* | 299 | 44 | 49 | 46 | 48 | 44 | 51 | 42 | 0 | 48 | 49 | 48 | 48 | 51 | 88 | 91 | 88 | 100 | 76 | 80 | 80 | 80 | 78 | 100 | 100 | 100 | 90 | 77 |
| Rv3804c | *fbpA* | 338 | 48 | 49 | 52 | 50 | 53 | 50 | 49 | 0 | 50 | 54 | 53 | 54 | 73 | 89 | 90 | 89 | 100 | 80 | 77 | 77 | 77 | 79 | 100 | 100 | 100 | 90 | 83 |
| Rv3805c | - | 627 | 54 | 50 | 54 | 53 | 0 | 49 | 0 | 0 | 65 | 67 | 64 | 64 | 76 | 88 | 92 | 88 | 100 | 81 | 82 | 82 | 82 | 81 | 100 | 100 | 100 | 92 | 80 |
| Rv3806c | - | 302 | 75 | 74 | 75 | 76 | 75 | 71 | 78 | 0 | 80 | 82 | 83 | 83 | 82 | 89 | 92 | 89 | 100 | 88 | 85 | 85 | 85 | 85 | 100 | 100 | 100 | 92 | 89 |
| Rv3807c | - | 165 | 70 | 71 | 63 | 66 | 66 | 71 | 66 | 0 | 73 | 74 | 77 | 77 | 80 | 80 | 84 | 80 | 100 | 81 | 84 | 84 | 84 | 78 | 100 | 100 | 100 | 83 | 84 |
| Rv3808c | *glfT* | 637 | 62 | 67 | 67 | 68 | 66 | 65 | 68 | 54 | 79 | 79 | 79 | 79 | 84 | 92 | 92 | 92 | 100 | 82 | 88 | 88 | 88 | 85 | 100 | 100 | 100 | 92 | 83 |
| Rv3809c | *glf* | 399 | 86 | 86 | 87 | 86 | 86 | 84 | 85 | 78 | 87 | 89 | 89 | 88 | 91 | 95 | 95 | 95 | 100 | 93 | 93 | 93 | 93 | 93 | 100 | 100 | 100 | 95 | 93 |
| Rv3810 | *pirG* | 284 | 0 | 0 | 0 | 0 | 0 | 0 | 0 | 0 | 0 | 0 | 0 | 0 | 55 | 65 | 64 | 64 | 100 | 60 | 66 | 66 | 66 | 68 | 100 | 100 | 100 | 81 | 62 |
| Rv3811 | - | 539 | 54 | 52 | 52 | 49 | 45 | 52 | 57 | 45 | 58 | 53 | 51 | 51 | 63 | 84 | 88 | 84 | 100 | 70 | 70 | 70 | 70 | 71 | 99 | 100 | 100 | 87 | 71 |
| Rv3812 | *PE_PGRS62* | 504 | 0 | 0 | 0 | 0 | 0 | 0 | 0 | 0 | 0 | 0 | 0 | 0 | 0 | 62 | 49 | 57 | 100 | 0 | 0 | 0 | 0 | 0 | 100 | 100 | 100 | 57 | 0 |
| Rv3813c | - | 273 | 59 | 59 | 59 | 58 | 60 | 58 | 52 | 52 | 73 | 75 | 78 | 77 | 79 | 88 | 92 | 88 | 99 | 86 | 87 | 87 | 87 | 86 | 100 | 100 | 100 | 92 | 87 |
| Rv3814c | - | 261 | 56 | 56 | 61 | 61 | 61 | 59 | 61 | 57 | 64 | 68 | 69 | 66 | 73 | 80 | 89 | 80 | 100 | 75 | 78 | 78 | 78 | 75 | 100 | 100 | 100 | 88 | 77 |
| Rv3815c | - | 251 | 59 | 59 | 59 | 59 | 60 | 56 | 57 | 60 | 67 | 67 | 69 | 68 | 76 | 88 | 89 | 88 | 100 | 74 | 78 | 78 | 78 | 76 | 100 | 100 | 100 | 88 | 77 |
| Rv3816c | - | 259 | 62 | 60 | 62 | 63 | 69 | 63 | 64 | 66 | 76 | 75 | 76 | 76 | 83 | 92 | 93 | 92 | 100 | 86 | 89 | 89 | 89 | 85 | 100 | 100 | 100 | 92 | 89 |
| Rv3817 | - | 251 | 0 | 0 | 0 | 0 | 0 | 0 | 43 | 0 | 43 | 0 | 0 | 0 | 68 | 0 | 0 | 0 | 100 | 0 | 0 | 0 | 0 | 0 | 100 | 100 | 100 | 78 | 0 |
| Rv3818 | - | 516 | 0 | 0 | 0 | 0 | 0 | 0 | 0 | 0 | 84 | 83 | 84 | 84 | 88 | 93 | 94 | 93 | 100 | 90 | 91 | 91 | 91 | 89 | 100 | 100 | 100 | 94 | 89 |
| Rv3819 | - | 111 | 0 | 0 | 0 | 0 | 0 | 0 | 0 | 0 | 0 | 52 | 0 | 0 | 0 | 83 | 83 | 83 | 100 | 78 | 80 | 80 | 80 | 78 | 100 | 100 | 100 | 82 | 81 |
| Rv3820c | *papA2* | 468 | 0 | 0 | 0 | 0 | 0 | 40 | 0 | 0 | 53 | 45 | 45 | 44 | 66 | 66 | 68 | 66 | 99 | 61 | 70 | 70 | 70 | 68 | 100 | 100 | 100 | 65 | 62 |
| Rv3821 | - | 237 | 0 | 0 | 0 | 0 | 0 | 0 | 0 | 0 | 0 | 0 | 0 | 0 | 60 | 62 | 53 | 62 | 99 | 57 | 58 | 58 | 58 | 60 | 100 | 100 | 100 | 52 | 62 |
| Rv3822 | - | 404 | 0 | 0 | 0 | 0 | 0 | 0 | 0 | 0 | 0 | 0 | 0 | 0 | 53 | 48 | 48 | 48 | 99 | 45 | 47 | 47 | 47 | 44 | 100 | 100 | 100 | 49 | 47 |
| Rv3823c | *mmpL8* | 1089 | 45 | 46 | 63 | 61 | 40 | 61 | 42 | 51 | 51 | 53 | 47 | 46 | 70 | 68 | 67 | 71 | 99 | 71 | 71 | 71 | 71 | 70 | 100 | 100 | 100 | 47 | 70 |
| Rv3824c | *papA1* | 511 | 0 | 0 | 0 | 0 | 0 | 40 | 0 | 0 | 50 | 45 | 46 | 45 | 70 | 70 | 67 | 70 | 100 | 68 | 68 | 68 | 68 | 67 | 100 | 100 | 99 | 65 | 68 |
| Rv3825c | *pks2* | 2126 | 49 | 51 | 54 | 54 | 50 | 44 | 50 | 44 | 48 | 51 | 46 | 50 | 73 | 79 | 80 | 79 | 100 | 48 | 77 | 78 | 78 | 77 | 100 | 100 | 100 | 77 | 47 |
| Rv3826 | *fadD23* | 584 | 50 | 53 | 50 | 51 | 52 | 37 | 50 | 40 | 53 | 54 | 54 | 54 | 71 | 76 | 79 | 72 | 100 | 69 | 72 | 72 | 72 | 71 | 100 | 100 | 100 | 78 | 70 |
| Rv3827c | - | 408 | 0 | 0 | 0 | 0 | 0 | 0 | 0 | 51 | 42 | 0 | 55 | 41 | 0 | 0 | 0 | 0 | 99 | 0 | 0 | 0 | 0 | 0 | 99 | 100 | 99 | 0 | 0 |

| | | | | | | | | | | | | | | | | | | | | | | | | | | | | | |
|---|---|---|---|---|---|---|---|---|---|---|---|---|---|---|---|---|---|---|---|---|---|---|---|---|---|---|---|---|---|
| Rv3828c | - | 203 | 0 | 0 | 0 | 0 | 0 | 0 | 0 | 0 | 0 | 0 | 0 | 0 | 0 | 0 | 0 | 0 | 100 | 0 | 0 | 0 | 0 | 0 | 100 | 100 | 100 | 0 | 0 |
| Rv3829c | - | 536 | 0 | 0 | 0 | 0 | 0 | 0 | 0 | 41 | 41 | 38 | 40 | 39 | 0 | 90 | 91 | 90 | 100 | 82 | 81 | 81 | 81 | 43 | 100 | 100 | 100 | 73 | 81 |
| Rv3830c | - | 209 | 0 | 0 | 0 | 0 | 0 | 0 | 0 | 0 | 47 | 46 | 46 | 60 | 0 | 88 | 87 | 88 | 100 | 79 | 79 | 79 | 79 | 0 | 100 | 100 | 100 | 86 | 79 |
| Rv3831 | - | 160 | 0 | 0 | 0 | 0 | 0 | 0 | 0 | 0 | 0 | 0 | 0 | 0 | 0 | 86 | 89 | 86 | 100 | 0 | 0 | 0 | 0 | 0 | 100 | 100 | 100 | 88 | 0 |
| Rv3832c | - | 191 | 0 | 0 | 0 | 0 | 0 | 0 | 0 | 45 | 0 | 0 | 0 | 0 | 72 | 80 | 81 | 81 | 100 | 0 | 0 | 0 | 0 | 0 | 100 | 100 | 100 | 72 | 58 |
| Rv3833 | - | 263 | 0 | 0 | 0 | 0 | 0 | 0 | 0 | 0 | 52 | 48 | 60 | 45 | 69 | 84 | 0 | 85 | 100 | 44 | 45 | 45 | 45 | 50 | 100 | 100 | 100 | 0 | 45 |
| Rv3834c | *serS* | 419 | 79 | 78 | 80 | 81 | 78 | 78 | 78 | 72 | 84 | 85 | 85 | 85 | 88 | 93 | 93 | 93 | 100 | 88 | 91 | 91 | 91 | 90 | 100 | 100 | 100 | 93 | 89 |
| Rv3835 | - | 449 | 47 | 50 | 50 | 48 | 43 | 48 | 45 | 0 | 49 | 52 | 52 | 52 | 66 | 77 | 83 | 79 | 100 | 71 | 72 | 72 | 72 | 66 | 100 | 100 | 100 | 82 | 73 |
| Rv3836 | - | 137 | 63 | 70 | 70 | 71 | 75 | 70 | 66 | 70 | 79 | 77 | 77 | 76 | 83 | 85 | 91 | 85 | 100 | 79 | 83 | 82 | 82 | 80 | 99 | 100 | 100 | 91 | 87 |
| Rv3837c | - | 232 | 51 | 48 | 50 | 52 | 41 | 43 | 44 | 43 | 61 | 60 | 64 | 64 | 72 | 82 | 80 | 82 | 100 | 71 | 76 | 76 | 76 | 76 | 100 | 100 | 100 | 78 | 71 |
| Rv3838c | *pheA* | 321 | 63 | 62 | 63 | 59 | 59 | 58 | 57 | 46 | 69 | 70 | 70 | 69 | 74 | 85 | 84 | 85 | 100 | 80 | 81 | 81 | 81 | 80 | 100 | 100 | 100 | 84 | 79 |
| Rv3839 | - | 258 | 0 | 0 | 0 | 0 | 0 | 0 | 0 | 0 | 60 | 62 | 64 | 64 | 65 | 83 | 84 | 84 | 99 | 73 | 73 | 73 | 73 | 76 | 100 | 100 | 100 | 83 | 74 |
| Rv3840 | - | 137 | 67 | 67 | 67 | 70 | 75 | 74 | 73 | 60 | 78 | 74 | 73 | 73 | 71 | 73 | 73 | 73 | 100 | 74 | 76 | 76 | 76 | 73 | 100 | 100 | 100 | 76 | 76 |
| Rv3841 | *bfrB* | 181 | 50 | 0 | 46 | 45 | 49 | 0 | 46 | 65 | 81 | 80 | 80 | 80 | 79 | 0 | 93 | 0 | 100 | 81 | 85 | 85 | 85 | 83 | 100 | 100 | 100 | 93 | 82 |
| Rv3842c | *glpQ1* | 274 | 49 | 52 | 51 | 53 | 50 | 51 | 52 | 55 | 82 | 81 | 79 | 78 | 87 | 95 | 94 | 95 | 100 | 89 | 86 | 86 | 86 | 90 | 100 | 100 | 99 | 93 | 90 |
| Rv3843c | - | 342 | 0 | 0 | 0 | 0 | 0 | 0 | 0 | 0 | 45 | 45 | 43 | 43 | 50 | 73 | 73 | 73 | 99 | 65 | 66 | 65 | 65 | 61 | 100 | 100 | 99 | 72 | 65 |
| Rv3844 | - | 163 | 0 | 0 | 0 | 0 | 0 | 0 | 0 | 0 | 0 | 0 | 0 | 0 | 0 | 0 | 0 | 0 | 100 | 0 | 0 | 0 | 0 | 0 | 100 | 100 | 100 | 0 | 0 |
| Rv3845 | - | 119 | 0 | 0 | 0 | 0 | 0 | 0 | 0 | 0 | 0 | 0 | 0 | 0 | 0 | 60 | 0 | 0 | 100 | 0 | 0 | 0 | 0 | 0 | 94 | 100 | 100 | 0 | 0 |
| Rv3846 | *sodA* | 207 | 74 | 75 | 73 | 74 | 73 | 74 | 75 | 0 | 84 | 77 | 77 | 77 | 89 | 90 | 98 | 90 | 99 | 0 | 87 | 87 | 87 | 89 | 100 | 100 | 100 | 98 | 0 |
| Rv3847 | - | 177 | 0 | 0 | 0 | 0 | 0 | 0 | 0 | 0 | 91 | 91 | 91 | 91 | 90 | 97 | 98 | 97 | 100 | 95 | 93 | 93 | 93 | 91 | 100 | 100 | 100 | 97 | 94 |
| Rv3848 | - | 302 | 0 | 0 | 0 | 0 | 0 | 0 | 0 | 48 | 63 | 61 | 57 | 63 | 0 | 0 | 0 | 0 | 100 | 57 | 67 | 63 | 63 | 55 | 100 | 100 | 100 | 70 | 60 |
| Rv3849 | - | 132 | 0 | 0 | 0 | 0 | 0 | 0 | 0 | 0 | 71 | 69 | 71 | 71 | 85 | 87 | 93 | 87 | 100 | 87 | 87 | 87 | 87 | 87 | 99 | 100 | 100 | 93 | 87 |
| Rv3850 | - | 218 | 55 | 54 | 54 | 53 | 0 | 49 | 48 | 0 | 62 | 72 | 71 | 71 | 78 | 94 | 93 | 94 | 100 | 89 | 89 | 89 | 89 | 93 | 100 | 100 | 100 | 91 | 89 |
| Rv3851 | - | 94 | 0 | 0 | 0 | 0 | 0 | 0 | 0 | 0 | 0 | 0 | 0 | 0 | 0 | 0 | 0 | 0 | 100 | 0 | 0 | 0 | 0 | 0 | 100 | 100 | 100 | 0 | 0 |
| Rv3852 | *hns* | 134 | 0 | 0 | 0 | 0 | 0 | 0 | 0 | 0 | 0 | 49 | 0 | 0 | 48 | 66 | 65 | 66 | 100 | 51 | 57 | 56 | 56 | 51 | 100 | 100 | 100 | 63 | 53 |
| Rv3853 | *menG* | 157 | 51 | 0 | 79 | 78 | 77 | 80 | 78 | 0 | 81 | 82 | 81 | 81 | 81 | 94 | 96 | 94 | 100 | 89 | 87 | 86 | 86 | 85 | 100 | 100 | 100 | 96 | 87 |
| Rv3854c | *ethA* | 489 | 43 | 0 | 0 | 0 | 56 | 0 | 0 | 71 | 68 | 70 | 75 | 74 | 76 | 79 | 92 | 78 | 100 | 78 | 76 | 76 | 76 | 78 | 100 | 100 | 100 | 92 | 79 |
| Rv3855 | *ethR* | 216 | 0 | 0 | 0 | 0 | 0 | 0 | 0 | 0 | 40 | 0 | 0 | 43 | 73 | 72 | 85 | 72 | 100 | 72 | 78 | 79 | 79 | 73 | 100 | 100 | 100 | 85 | 76 |
| Rv3856c | - | 335 | 0 | 0 | 0 | 0 | 0 | 0 | 0 | 70 | 77 | 78 | 80 | 79 | 83 | 94 | 94 | 94 | 100 | 89 | 91 | 90 | 90 | 89 | 100 | 100 | 100 | 94 | 90 |
| Rv3857c | - | 65 | 0 | 0 | 0 | 0 | 0 | 0 | 0 | 0 | 0 | 0 | 0 | 0 | 0 | 0 | 68 | 0 | 100 | 0 | 0 | 0 | 0 | 0 | 100 | 100 | 100 | 66 | 0 |
| Rv3858c | *gltD* | 488 | 45 | 51 | 68 | 71 | 70 | 71 | 48 | 78 | 88 | 87 | 87 | 88 | 88 | 93 | 94 | 93 | 99 | 91 | 90 | 90 | 90 | 90 | 100 | 100 | 100 | 95 | 91 |
| Rv3859c | *gltB* | 1527 | 0 | 0 | 75 | 75 | 76 | 75 | 45 | 77 | 85 | 85 | 85 | 85 | 89 | 96 | 96 | 96 | 100 | 91 | 91 | 92 | 92 | 91 | 100 | 100 | 100 | 96 | 90 |
| Rv3860 | - | 390 | 0 | 0 | 0 | 0 | 0 | 0 | 0 | 37 | 50 | 45 | 47 | 48 | 47 | 75 | 70 | 75 | 99 | 67 | 64 | 64 | 64 | 62 | 100 | 100 | 100 | 46 | 68 |
| Rv3861 | - | 108 | 0 | 0 | 0 | 0 | 0 | 0 | 0 | 0 | 0 | 0 | 0 | 0 | 0 | 0 | 0 | 0 | 100 | 0 | 0 | 0 | 0 | 0 | 99 | 100 | 100 | 0 | 0 |
| Rv3862c | *whiB6* | 116 | 0 | 0 | 0 | 0 | 0 | 0 | 0 | 0 | 0 | 0 | 0 | 0 | 0 | 0 | 85 | 0 | 100 | 84 | 79 | 79 | 79 | 78 | 100 | 100 | 100 | 0 | 83 |
| Rv3863 | - | 392 | 0 | 0 | 0 | 0 | 0 | 0 | 0 | 0 | 0 | 0 | 0 | 0 | 58 | 0 | 78 | 0 | 100 | 71 | 75 | 75 | 75 | 64 | 100 | 100 | 100 | 0 | 72 |
| Rv3864 | - | 402 | 0 | 0 | 0 | 0 | 0 | 0 | 0 | 0 | 0 | 0 | 0 | 0 | 0 | 0 | 82 | 0 | 96 | 0 | 51 | 0 | 0 | 0 | 100 | 100 | 100 | 47 | 0 |
| Rv3865 | - | 103 | 0 | 0 | 0 | 0 | 0 | 0 | 0 | 0 | 0 | 0 | 0 | 0 | 0 | 0 | 88 | 0 | 100 | 0 | 0 | 0 | 0 | 0 | 100 | 100 | 100 | 56 | 0 |

| | | | | | | | | | | | | | | | | | | | | | | | | | | | | | |
|---|---|---|---|---|---|---|---|---|---|---|---|---|---|---|---|---|---|---|---|---|---|---|---|---|---|---|---|---|---|
| Rv3866 | - | 283 | 0 | 0 | 0 | 0 | 0 | 0 | 0 | 0 | 48 | 0 | 38 | 40 | 43 | 43 | 89 | 44 | 100 | 77 | 79 | 79 | 79 | 78 | 100 | 100 | 100 | 0 | 82 |
| Rv3867 | - | 183 | 0 | 0 | 0 | 0 | 0 | 0 | 0 | 0 | 0 | 0 | 0 | 0 | 0 | 0 | 81 | 0 | 100 | 57 | 59 | 58 | 58 | 60 | 100 | 100 | 100 | 73 | 57 |
| Rv3868 | - | 573 | 0 | 0 | 0 | 0 | 0 | 0 | 0 | 0 | 56 | 0 | 0 | 0 | 52 | 54 | 93 | 53 | 99 | 86 | 87 | 87 | 87 | 86 | 100 | 100 | 100 | 53 | 88 |
| Rv3869 | - | 480 | 42 | 40 | 38 | 39 | 42 | 57 | 39 | 35 | 54 | 54 | 0 | 54 | 54 | 51 | 90 | 51 | 100 | 78 | 81 | 81 | 81 | 82 | 100 | 100 | 100 | 52 | 79 |
| Rv3870 | - | 747 | 51 | 50 | 49 | 50 | 66 | 50 | 65 | 51 | 55 | 51 | 47 | 55 | 54 | 50 | 94 | 49 | 100 | 88 | 89 | 89 | 89 | 88 | 100 | 100 | 100 | 85 | 89 |
| Rv3871 | - | 591 | 47 | 48 | 49 | 49 | 43 | 46 | 48 | 56 | 56 | 57 | 0 | 56 | 57 | 53 | 95 | 53 | 100 | 86 | 88 | 88 | 88 | 89 | 100 | 100 | 100 | 94 | 87 |
| Rv3872 | *PE35* | 99 | 0 | 0 | 0 | 0 | 0 | 0 | 0 | 0 | 0 | 0 | 0 | 0 | 0 | 0 | 0 | 0 | 100 | 61 | 61 | 61 | 61 | 70 | 100 | 100 | 100 | 70 | 65 |
| Rv3873 | *PPE68* | 368 | 0 | 0 | 0 | 0 | 0 | 0 | 0 | 0 | 38 | 39 | 39 | 38 | 41 | 45 | 82 | 47 | 100 | 53 | 57 | 56 | 56 | 56 | 99 | 100 | 100 | 58 | 56 |
| Rv3874 | *esxB* | 100 | 0 | 0 | 0 | 0 | 0 | 0 | 0 | 0 | 0 | 0 | 0 | 0 | 0 | 0 | 0 | 0 | 100 | 64 | 71 | 71 | 71 | 71 | 100 | 100 | 100 | 0 | 66 |
| Rv3875 | *esxA* | 95 | 0 | 60 | 55 | 55 | 56 | 55 | 0 | 0 | 0 | 0 | 0 | 0 | 0 | 0 | 96 | 0 | 100 | 65 | 74 | 74 | 74 | 80 | 100 | 100 | 100 | 0 | 58 |
| Rv3876 | - | 666 | 41 | 37 | 37 | 36 | 38 | 32 | 37 | 35 | 41 | 46 | 51 | 56 | 44 | 51 | 48 | 48 | 100 | 61 | 58 | 58 | 58 | 61 | 100 | 100 | 100 | 57 | 62 |
| Rv3877 | - | 511 | 0 | 0 | 0 | 0 | 0 | 0 | 0 | 37 | 43 | 44 | 0 | 42 | 42 | 39 | 88 | 39 | 100 | 79 | 81 | 81 | 81 | 80 | 100 | 100 | 100 | 87 | 82 |
| Rv3878 | - | 280 | 0 | 0 | 0 | 0 | 0 | 0 | 0 | 0 | 0 | 0 | 0 | 0 | 0 | 0 | 56 | 0 | 100 | 45 | 36 | 40 | 40 | 0 | 99 | 100 | 100 | 52 | 46 |
| Rv3879c | - | 729 | 39 | 48 | 38 | 34 | 37 | 37 | 39 | 39 | 41 | 35 | 38 | 39 | 36 | 36 | 34 | 34 | 97 | 53 | 50 | 49 | 49 | 47 | 99 | 100 | 100 | 59 | 50 |
| Rv3880c | - | 115 | 0 | 0 | 0 | 0 | 0 | 0 | 0 | 0 | 0 | 0 | 0 | 0 | 0 | 0 | 85 | 0 | 100 | 63 | 68 | 68 | 68 | 67 | 100 | 100 | 100 | 77 | 68 |
| Rv3881c | - | 460 | 0 | 0 | 0 | 0 | 0 | 0 | 0 | 0 | 38 | 0 | 0 | 0 | 0 | 0 | 79 | 0 | 98 | 41 | 42 | 42 | 42 | 42 | 100 | 100 | 100 | 50 | 37 |
| Rv3882c | - | 462 | 0 | 0 | 0 | 0 | 0 | 0 | 0 | 0 | 44 | 0 | 0 | 43 | 37 | 0 | 89 | 39 | 100 | 80 | 81 | 81 | 81 | 80 | 100 | 100 | 100 | 0 | 81 |
| Rv3883c | *mycP1* | 446 | 45 | 48 | 44 | 48 | 40 | 47 | 41 | 43 | 59 | 55 | 0 | 53 | 60 | 58 | 90 | 58 | 99 | 82 | 83 | 83 | 83 | 83 | 100 | 100 | 100 | 57 | 84 |
| Rv3884c | - | 619 | 0 | 0 | 0 | 0 | 0 | 0 | 0 | 0 | 49 | 0 | 0 | 0 | 46 | 93 | 94 | 93 | 99 | 46 | 46 | 46 | 46 | 47 | 99 | 100 | 100 | 48 | 46 |
| Rv3885c | - | 537 | 0 | 0 | 0 | 0 | 0 | 0 | 0 | 0 | 42 | 38 | 38 | 37 | 0 | 86 | 0 | 86 | 99 | 41 | 0 | 0 | 0 | 0 | 100 | 100 | 100 | 0 | 0 |
| Rv3886c | *mycP2* | 550 | 50 | 69 | 46 | 46 | 0 | 37 | 0 | 37 | 61 | 62 | 35 | 58 | 62 | 90 | 57 | 91 | 100 | 61 | 51 | 51 | 51 | 57 | 98 | 100 | 100 | 57 | 52 |
| Rv3887c | - | 509 | 0 | 0 | 0 | 0 | 0 | 0 | 0 | 0 | 38 | 40 | 0 | 40 | 40 | 90 | 90 | 90 | 97 | 36 | 36 | 37 | 37 | 37 | 100 | 100 | 100 | 41 | 38 |
| Rv3888c | - | 341 | 0 | 0 | 0 | 0 | 0 | 0 | 0 | 40 | 51 | 51 | 52 | 53 | 50 | 95 | 49 | 95 | 53 | 55 | 58 | 58 | 58 | 56 | 100 | 100 | 100 | 51 | 57 |
| Rv3889c | - | 276 | 0 | 0 | 0 | 0 | 0 | 0 | 0 | 0 | 42 | 0 | 0 | 0 | 0 | 89 | 89 | 89 | 100 | 0 | 0 | 0 | 0 | 0 | 100 | 100 | 100 | 0 | 0 |
| Rv3890c | *esxC* | 95 | 0 | 0 | 0 | 0 | 0 | 0 | 0 | 0 | 0 | 0 | 0 | 0 | 0 | 89 | 0 | 89 | 92 | 0 | 0 | 0 | 0 | 0 | 100 | 100 | 100 | 0 | 0 |
| Rv3891c | *esxD* | 107 | 0 | 0 | 0 | 0 | 0 | 0 | 0 | 0 | 0 | 0 | 0 | 0 | 0 | 84 | 0 | 84 | 99 | 0 | 0 | 0 | 0 | 0 | 100 | 100 | 100 | 0 | 0 |
| Rv3892c | *PPE69* | 399 | 0 | 0 | 0 | 0 | 0 | 0 | 36 | 0 | 0 | 0 | 34 | 0 | 35 | 83 | 38 | 82 | 99 | 0 | 33 | 33 | 33 | 33 | 99 | 100 | 99 | 38 | 35 |
| Rv3893c | *PE36* | 77 | 0 | 0 | 0 | 0 | 0 | 0 | 0 | 0 | 0 | 0 | 0 | 0 | 0 | 89 | 0 | 89 | 100 | 0 | 0 | 0 | 0 | 0 | 100 | 100 | 100 | 0 | 0 |
| Rv3894c | - | 1396 | 43 | 40 | 42 | 43 | 41 | 42 | 41 | 45 | 47 | 48 | 45 | 47 | 46 | 93 | 93 | 93 | 99 | 43 | 44 | 44 | 44 | 45 | 99 | 100 | 99 | 48 | 43 |
| Rv3895c | - | 495 | 0 | 41 | 38 | 37 | 37 | 50 | 50 | 0 | 55 | 51 | 0 | 54 | 52 | 93 | 55 | 93 | 99 | 53 | 53 | 53 | 53 | 53 | 100 | 100 | 100 | 54 | 51 |
| Rv3896c | - | 302 | 0 | 0 | 0 | 0 | 0 | 65 | 0 | 0 | 64 | 0 | 0 | 0 | 0 | 73 | 75 | 72 | 99 | 0 | 0 | 0 | 0 | 0 | 100 | 100 | 100 | 75 | 0 |
| Rv3897c | - | 210 | 0 | 0 | 0 | 0 | 0 | 0 | 0 | 0 | 0 | 0 | 0 | 0 | 0 | 76 | 76 | 76 | 98 | 38 | 0 | 39 | 39 | 0 | 98 | 100 | 98 | 80 | 36 |
| Rv3898c | - | 110 | 0 | 0 | 0 | 0 | 0 | 0 | 0 | 0 | 0 | 0 | 0 | 0 | 0 | 55 | 49 | 55 | 100 | 0 | 0 | 0 | 0 | 0 | 100 | 100 | 100 | 49 | 0 |
| Rv3899c | - | 410 | 46 | 0 | 38 | 38 | 39 | 36 | 40 | 38 | 37 | 0 | 0 | 0 | 43 | 76 | 68 | 76 | 100 | 42 | 38 | 38 | 38 | 43 | 99 | 100 | 100 | 67 | 41 |
| Rv3900c | - | 311 | 0 | 0 | 0 | 0 | 0 | 0 | 0 | 0 | 0 | 0 | 0 | 0 | 0 | 60 | 49 | 60 | 99 | 0 | 0 | 0 | 0 | 0 | 99 | 100 | 99 | 46 | 0 |
| Rv3901c | - | 149 | 0 | 0 | 0 | 0 | 0 | 0 | 0 | 0 | 0 | 0 | 0 | 0 | 0 | 67 | 64 | 64 | 99 | 0 | 0 | 0 | 0 | 0 | 99 | 100 | 100 | 61 | 0 |
| Rv3902c | - | 176 | 0 | 0 | 0 | 0 | 0 | 0 | 0 | 0 | 0 | 0 | 0 | 0 | 0 | 57 | 0 | 58 | 100 | 0 | 0 | 0 | 0 | 0 | 100 | 100 | 100 | 0 | 0 |
| Rv3903c | - | 846 | 0 | 36 | 42 | 0 | 38 | 0 | 36 | 43 | 44 | 35 | 39 | 35 | 37 | 55 | 65 | 62 | 100 | 43 | 39 | 37 | 37 | 35 | 100 | 100 | 100 | 40 | 42 |

| | | | | | | | | | | | | | | | | | | | | | | | | | | | | | |
|---|---|---|---|---|---|---|---|---|---|---|---|---|---|---|---|---|---|---|---|---|---|---|---|---|---|---|---|---|---|
| Rv3904c | *esxE* | 90 | 0 | 0 | 0 | 0 | 0 | 0 | 0 | 50 | 59 | 0 | 0 | 0 | 0 | 82 | 84 | 82 | 100 | 0 | 55 | 55 | 55 | 55 | 100 | 100 | 100 | 78 | 56 |
| Rv3905c | *esxF* | 103 | 0 | 0 | 0 | 0 | 0 | 0 | 0 | 0 | 0 | 0 | 0 | 0 | 0 | 78 | 77 | 78 | 99 | 0 | 0 | 54 | 54 | 54 | 100 | 100 | 100 | 76 | 0 |
| Rv3906c | - | 169 | 0 | 0 | 0 | 0 | 48 | 0 | 0 | 0 | 0 | 0 | 0 | 0 | 66 | 85 | 88 | 85 | 100 | 68 | 73 | 73 | 73 | 72 | 100 | 100 | 100 | 87 | 68 |
| Rv3907c | *pcnA* | 480 | 76 | 77 | 77 | 76 | 71 | 73 | 76 | 71 | 81 | 80 | 83 | 83 | 85 | 93 | 94 | 93 | 100 | 86 | 88 | 88 | 88 | 89 | 100 | 100 | 100 | 94 | 86 |
| Rv3908 | - | 248 | 62 | 61 | 55 | 57 | 60 | 60 | 60 | 0 | 82 | 81 | 80 | 79 | 73 | 86 | 86 | 86 | 100 | 78 | 79 | 79 | 79 | 72 | 99 | 100 | 100 | 87 | 80 |
| Rv3909 | - | 802 | 42 | 40 | 41 | 41 | 38 | 50 | 37 | 0 | 55 | 57 | 59 | 58 | 68 | 83 | 86 | 84 | 99 | 74 | 74 | 74 | 74 | 74 | 100 | 100 | 100 | 86 | 76 |
| Rv3910 | - | 1184 | 66 | 61 | 66 | 64 | 61 | 61 | 61 | 46 | 58 | 59 | 59 | 60 | 65 | 84 | 84 | 84 | 99 | 74 | 78 | 77 | 77 | 78 | 100 | 100 | 99 | 86 | 76 |
| Rv3911 | *sigM* | 222 | 50 | 55 | 53 | 53 | 50 | 43 | 48 | 50 | 65 | 65 | 64 | 64 | 78 | 88 | 81 | 88 | 95 | 76 | 67 | 67 | 67 | 73 | 95 | 95 | 95 | 80 | 78 |
| Rv3912 | - | 254 | 0 | 0 | 0 | 0 | 0 | 0 | 0 | 0 | 0 | 42 | 48 | 45 | 48 | 67 | 71 | 73 | 100 | 51 | 0 | 0 | 0 | 54 | 100 | 100 | 100 | 72 | 55 |
| Rv3913 | *trxB2* | 335 | 79 | 77 | 81 | 80 | 79 | 76 | 76 | 76 | 84 | 86 | 84 | 86 | 87 | 87 | 87 | 87 | 99 | 85 | 88 | 88 | 88 | 87 | 100 | 100 | 100 | 94 | 87 |
| Rv3914 | *trxC* | 116 | 70 | 66 | 66 | 66 | 62 | 68 | 60 | 73 | 83 | 86 | 85 | 85 | 83 | 94 | 94 | 94 | 100 | 91 | 92 | 92 | 92 | 88 | 100 | 100 | 100 | 95 | 88 |
| Rv3915 | - | 406 | 67 | 69 | 66 | 68 | 70 | 71 | 71 | 0 | 77 | 77 | 78 | 79 | 83 | 94 | 95 | 94 | 100 | 90 | 91 | 91 | 91 | 92 | 100 | 100 | 100 | 95 | 90 |
| Rv3916c | - | 244 | 45 | 0 | 49 | 49 | 39 | 40 | 48 | 50 | 62 | 66 | 64 | 64 | 74 | 90 | 90 | 90 | 100 | 79 | 81 | 81 | 81 | 78 | 100 | 100 | 100 | 89 | 79 |
| Rv3917c | *parB* | 344 | 69 | 65 | 61 | 62 | 75 | 71 | 72 | 69 | 78 | 74 | 73 | 73 | 82 | 90 | 90 | 90 | 100 | 85 | 85 | 85 | 85 | 77 | 100 | 100 | 100 | 91 | 84 |
| Rv3918c | *parA* | 347 | 79 | 80 | 76 | 76 | 75 | 70 | 76 | 72 | 86 | 84 | 85 | 85 | 82 | 88 | 89 | 87 | 100 | 89 | 87 | 87 | 87 | 86 | 100 | 100 | 100 | 88 | 85 |
| Rv3919c | *gidB* | 224 | 68 | 66 | 67 | 65 | 70 | 68 | 67 | 62 | 70 | 71 | 70 | 70 | 73 | 83 | 82 | 82 | 99 | 81 | 77 | 76 | 76 | 75 | 99 | 99 | 99 | 88 | 79 |
| Rv3920c | - | 187 | 0 | 0 | 0 | 0 | 0 | 59 | 0 | 72 | 83 | 76 | 74 | 74 | 83 | 95 | 86 | 88 | 99 | 83 | 84 | 84 | 84 | 83 | 100 | 100 | 100 | 86 | 84 |
| Rv3921c | - | 366 | 58 | 65 | 64 | 64 | 64 | 64 | 65 | 46 | 75 | 73 | 75 | 74 | 82 | 88 | 89 | 88 | 100 | 81 | 83 | 84 | 84 | 84 | 100 | 100 | 100 | 88 | 81 |
| Rv3922c | - | 120 | 69 | 67 | 70 | 62 | 69 | 75 | 67 | 65 | 72 | 76 | 83 | 70 | 84 | 74 | 81 | 73 | 100 | 74 | 77 | 77 | 77 | 68 | 100 | 100 | 100 | 81 | 77 |
| Rv3923c | *rnpA* | 116 | 58 | 61 | 59 | 58 | 52 | 56 | 53 | 58 | 54 | 61 | 62 | 62 | 66 | 72 | 74 | 74 | 100 | 63 | 67 | 67 | 67 | 66 | 100 | 100 | 100 | 79 | 70 |
| Rv3924c | *rpmH* | 47 | 87 | 93 | 91 | 93 | 93 | 93 | 93 | 97 | 95 | 95 | 95 | 95 | 91 | 93 | 93 | 93 | 100 | 95 | 95 | 95 | 95 | 93 | 100 | 100 | 100 | 93 | 95 |
