# Supplementary material for: atpE gene as a new useful specific molecular target to quantify Mycobacterium in environmental samples
Source: BMC Microbiol. 2013 Dec 3;13:277. doi: 10.1186/1471-2180-13-277 (PMC4219376; doi:10.1186/1471-2180-13-277)
Supplement: Additional file 2 — Protein sequence alignment of conserved proteins in mycobacterial genomes. Sequences are from genomes of M. abscessus ATCC 19977 (CU458896.1), M. avium 104 (CP000479.1), M. avium subsp. paratuberculosis K10 (AE016958.1), M. bovis subsp. bovis AF2122/97 (BX248333.1), M. bovis BCG Pasteur 1173P2 (AM408590.1), M. bovis BCG Tokyo 172 (AP010918.1), M. gilvum PYR-GCK (CP000656.1), M. intracellulare ATCC 13950 (ABIN00000000), M. kansasii ATCC 12478 (ACBV00000000), M. leprae Br4923 (FM211192.1), M. leprae TN (AL450380.1), M. marinum M (CP000854.1), M. parascrofulaceum BAA-614 (ADNV00000000), M. smegmatis MC2 155 (CP000480.1), Mycobacterium sp. JLS (CP000580.1), Mycobacterium sp. KMS (CP000518.1), Mycobacterium sp. MCS (CP000384.1), M. tuberculosis CDC1551 (AE000516.2), M. tuberculosis H37Ra (CP000611.1), M. tuberculosis H37Rv (AL123456.2), M. tuberculosis KZN 1435 (CP001658.1), M. ulcerans Agy99 (CP000325.1) and M. vanbaalenii PYR-1 (CP000511.1). [file 1471-2180-13-277-S2.pdf]

Additional file 2 - Protein sequence alignment of conserved proteins in genomes of *M. abscessus* ATCC 19977 (CU458896.1), *M. avium* 104 (CP000479.1), *M. avium* subsp. *paratuberculosis* K10 (AE016958.1), *M. bovis* subsp. *bovis* AF2122/97 (BX248333.1), *M. bovis* BCG Pasteur 1173P2 (AM408590.1), *M. bovis* BCG Tokyo 172 (AP010918.1), *M. gilvum* PYR-GCK (CP000656.1), *M. intracellulare* ATCC 13950 (ABIN00000000), *M. kansasii* ATCC 12478 (ACBV00000000), *M. leprae* Br4923 (FM211192.1), *M. leprae* TN (AL450380.1), *M. marinum* M (CP000854.1), *M. parascrofulaceum* BAA-614 (ADNV00000000), *M. smegmatis* MC2 155 (CP000480.1), *Mycobacterium* sp. JLS (CP000580.1), *Mycobacterium* sp. KMS (CP000518.1), *Mycobacterium* sp. MCS (CP000384.1), *M. tuberculosis* CDC1551 (AE000516.2), *M. tuberculosis* H37Ra (CP000611.1), *M. tuberculosis* H37Rv (AL123456.2), *M. tuberculosis* KZN 1435 (CP001658.1), *M. ulcerans* Agy99 (CP000325.1) and *M. vanbaalenii* PYR-1 (CP000511.1).

1/ Protein sequence alignment of *atpE* gene coding ATP synthase subunit C in *Mycobacterium* genome (locus tag Rv1305 in genome of *M. tuberculosis* H37Rv).

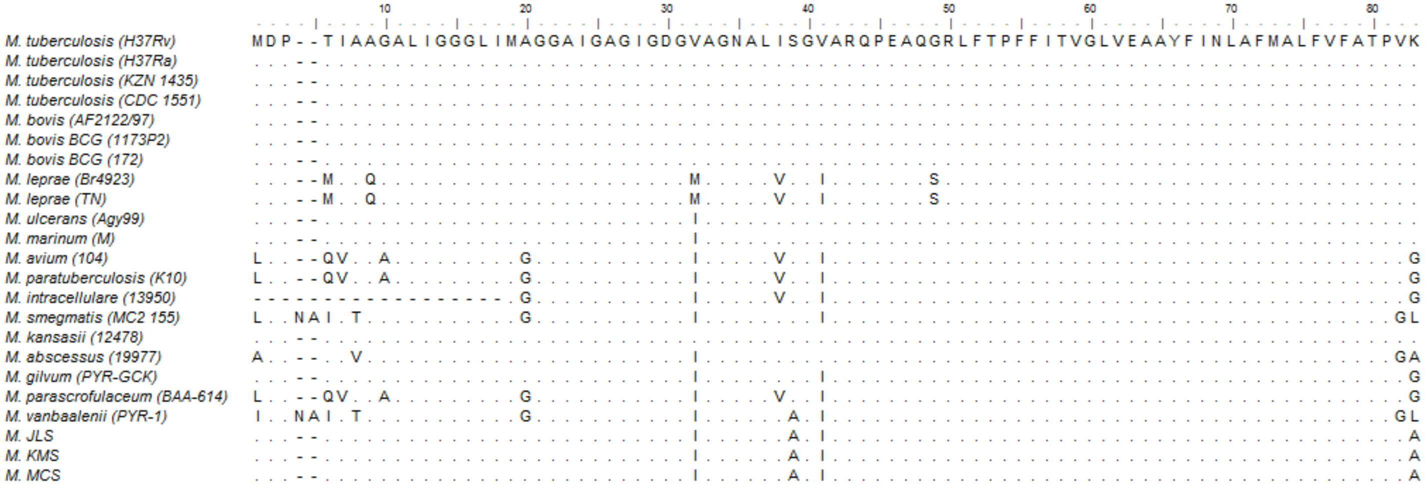

2 /Protein sequence alignment of unnamed gene coding small secreted protein in *Mycobacterium* genome (locus tag Rv0236A in genome of *M. tuberculosis* H37Rv).

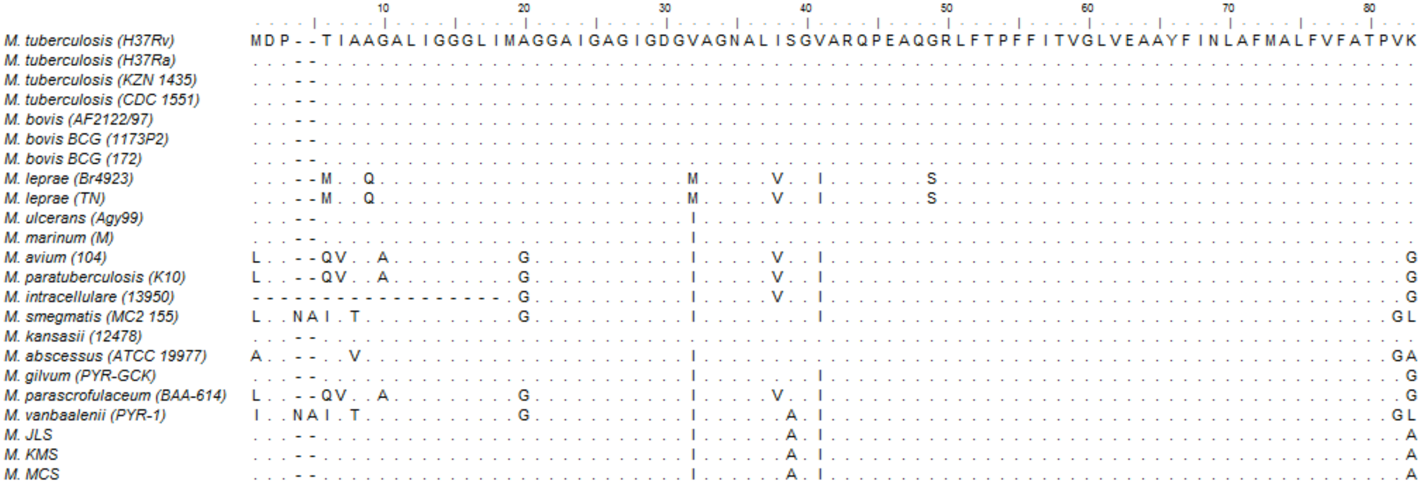

3.a/ Protein sequence alignment of unnamed gene coding hypothetical oxidoreductase in *Mycobacterium* genome (locus tag Rv0197 in genome of *M. tuberculosis* H37Rv).

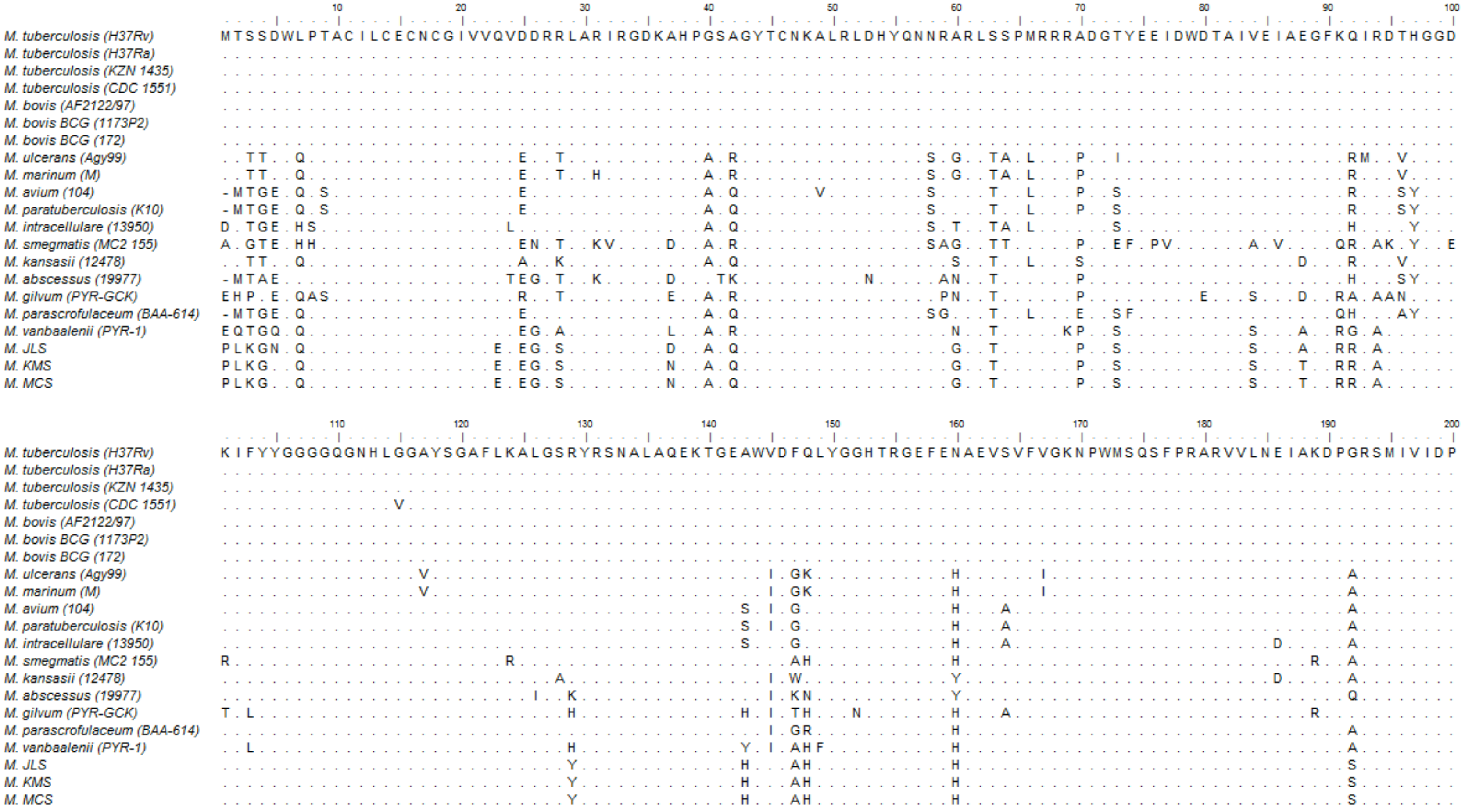

3.b/ Protein sequence alignment of unnamed gene coding hypothetical oxidoreductase in *Mycobacterium* genome (locus tag Rv0197 in genome of *M. tuberculosis* H37Rv).

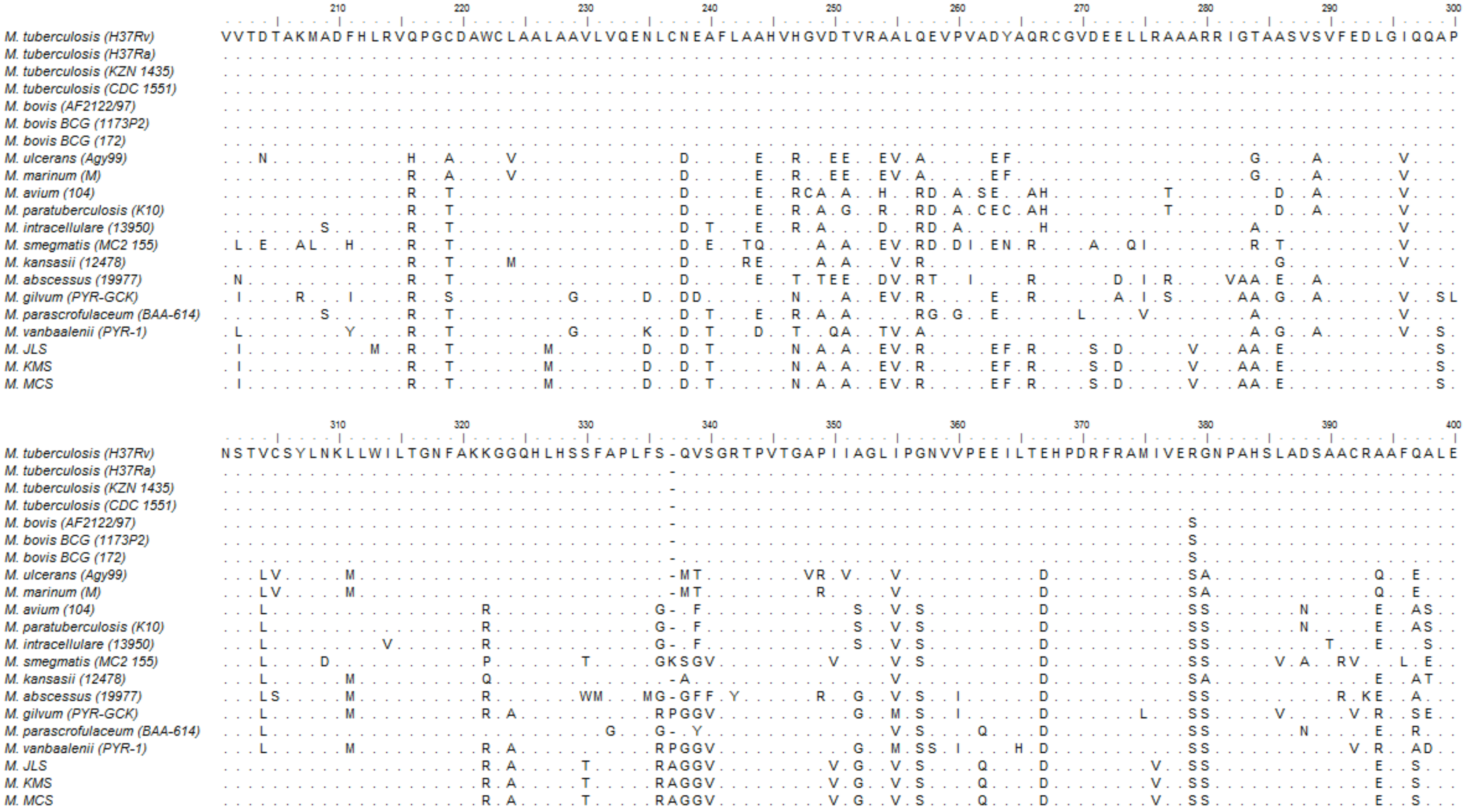

3.c/ Protein sequence alignment of unnamed gene coding hypothetical oxidoreductase in *Mycobacterium* genome (locus tag Rv0197 in genome of *M. tuberculosis* H37Rv).

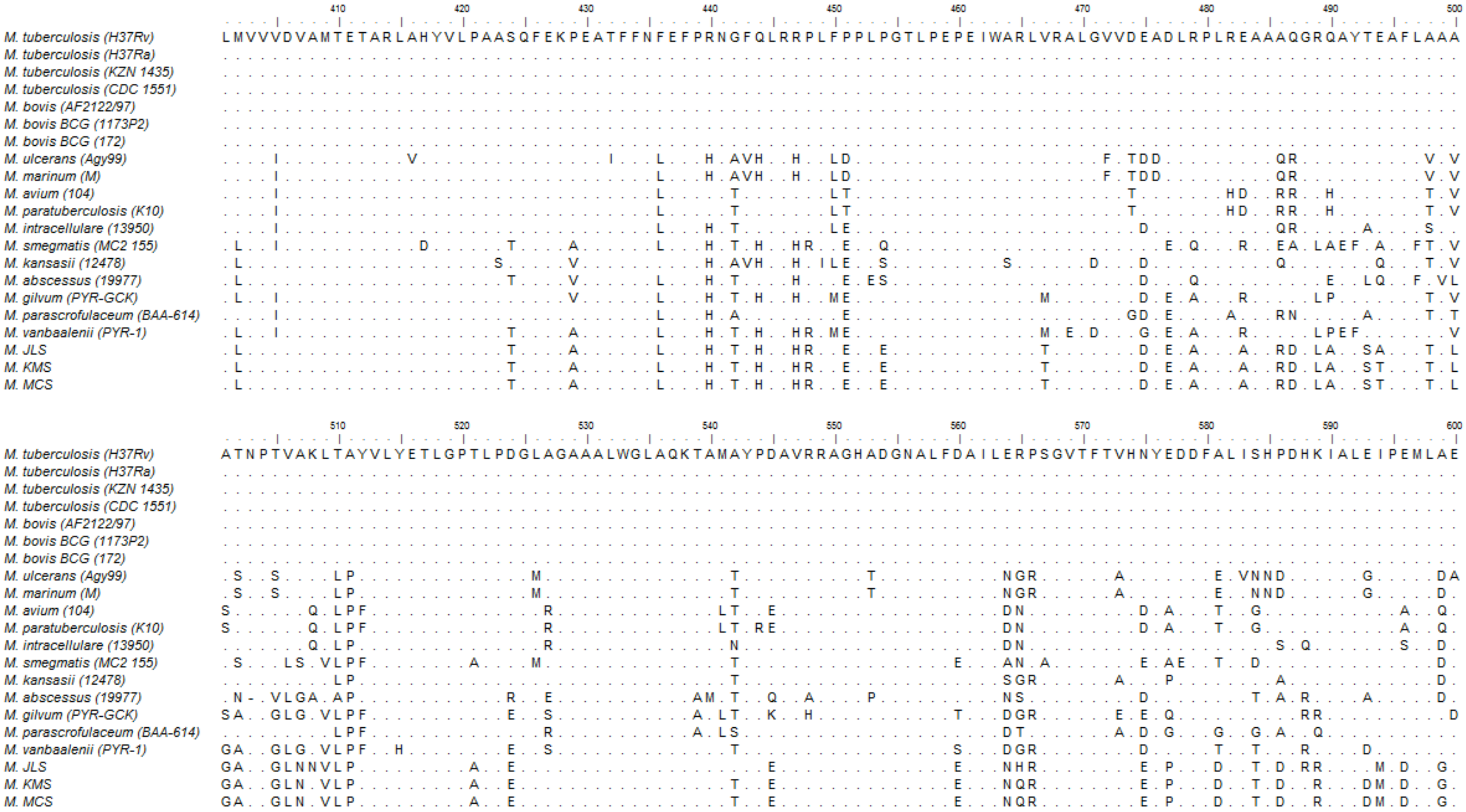

3.d/ Protein sequence alignment of unnamed gene coding hypothetical oxidoreductase in *Mycobacterium* genome (locus tag Rv0197 in genome of *M. tuberculosis* H37Rv).

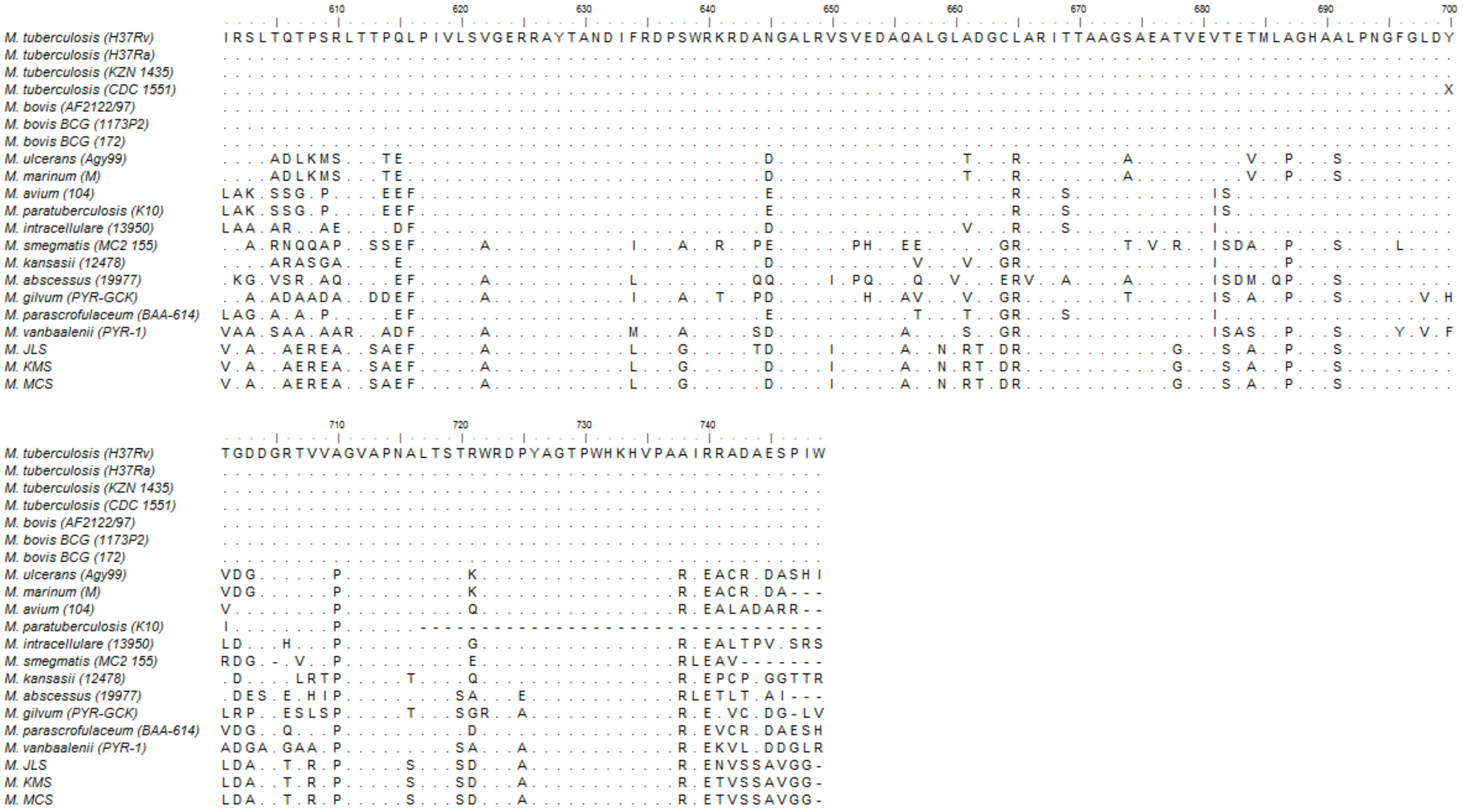

4.a/ Protein sequence alignment of *lppM* gene coding hypothetical lipoprotein in *Mycobacterium* genome (locus tag Rv2172c in genome of *M. tuberculosis* H37Rv).

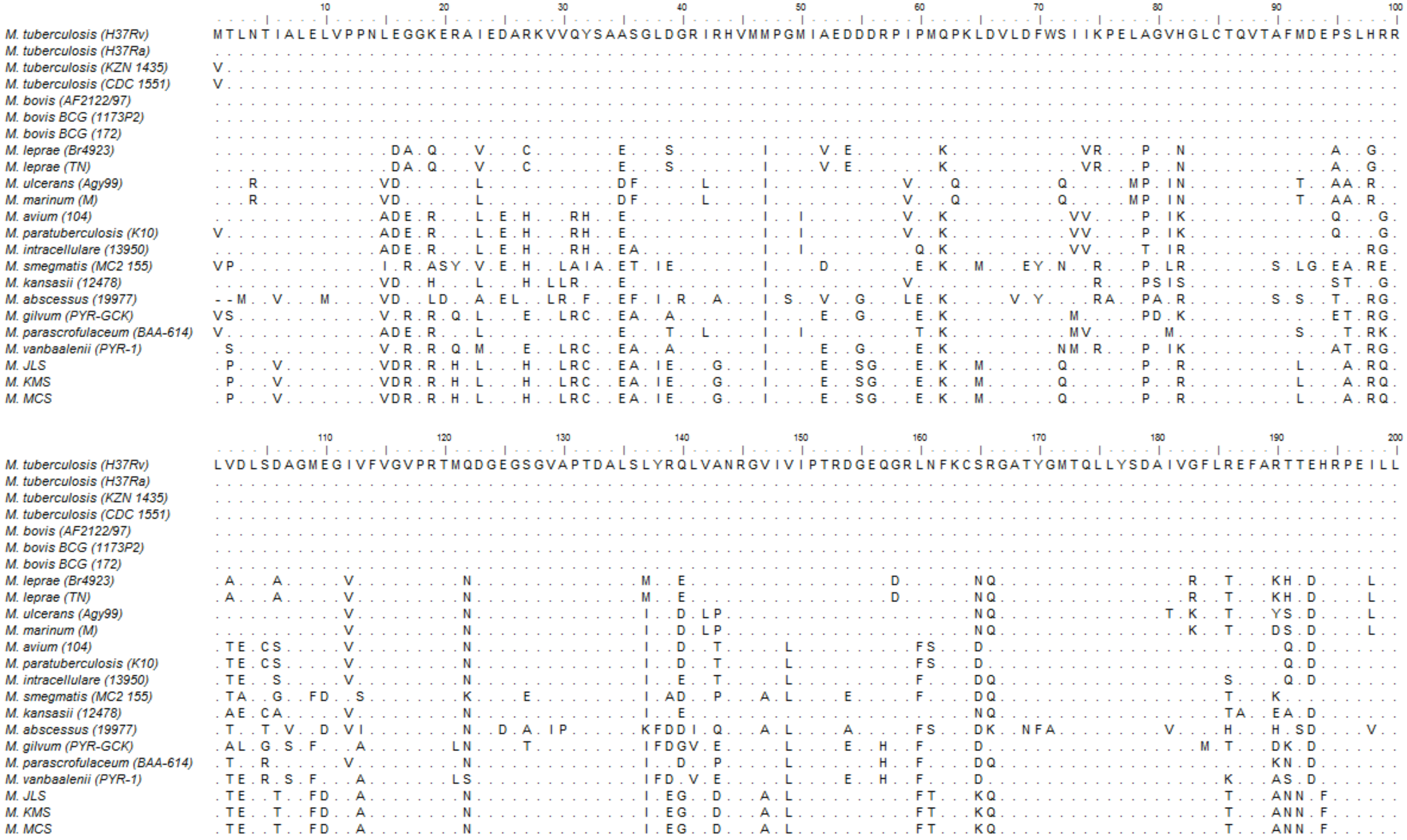

4.b/ Protein sequence alignment of *lppM* gene coding hypothetical lipoprotein in *Mycobacterium* genome (locus tag Rv2172c in genome of *M. tuberculosis* H37Rv).

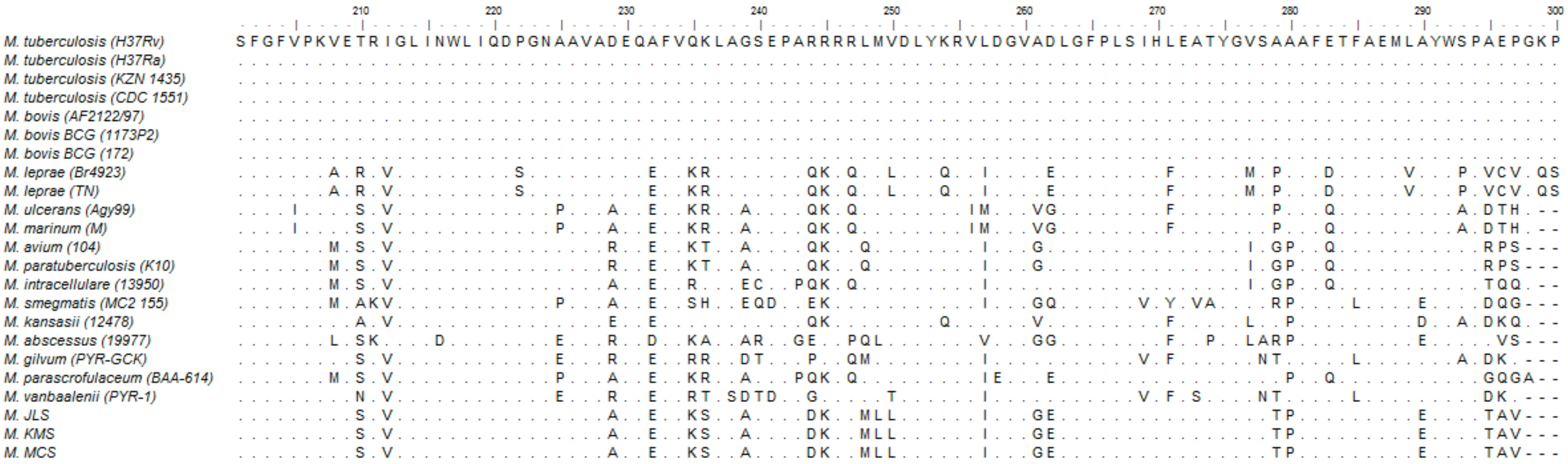

5/ Protein sequence alignment of *esxG* gene coding hypothetical PE family protein in *Mycobacterium* genome (locus tag Rv0287 in genome of *M. tuberculosis* H37Rv).

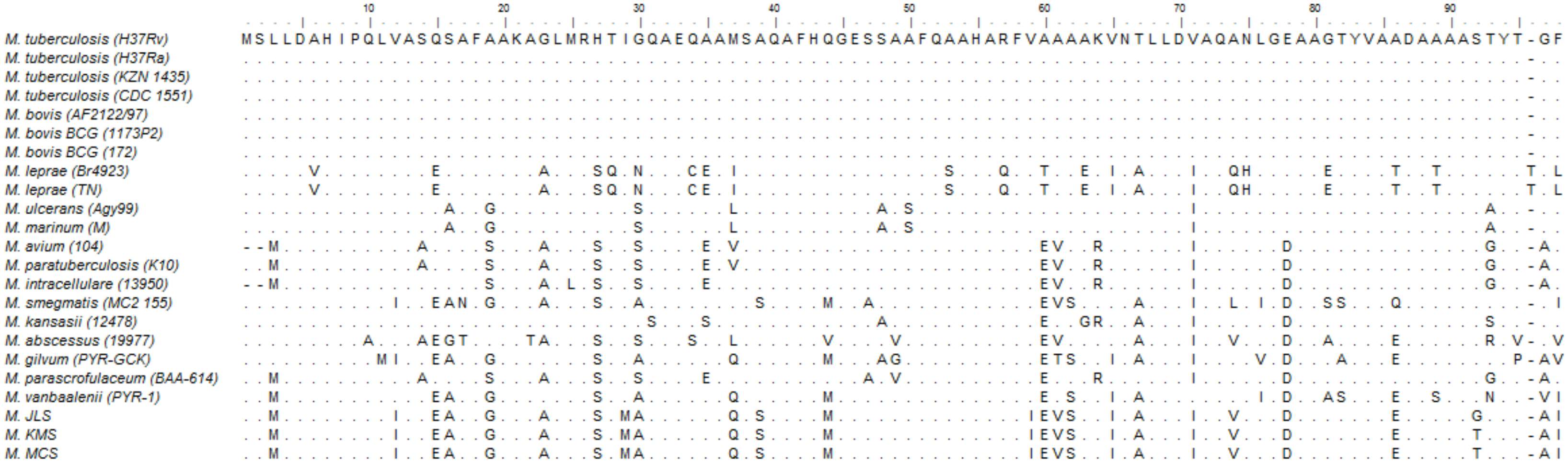

6/ Protein sequence alignment of *esxH* gene coding hypothetical PE family protein in *Mycobacterium* genome (locus tag Rv0288 in genome of *M. tuberculosis* H37Rv).

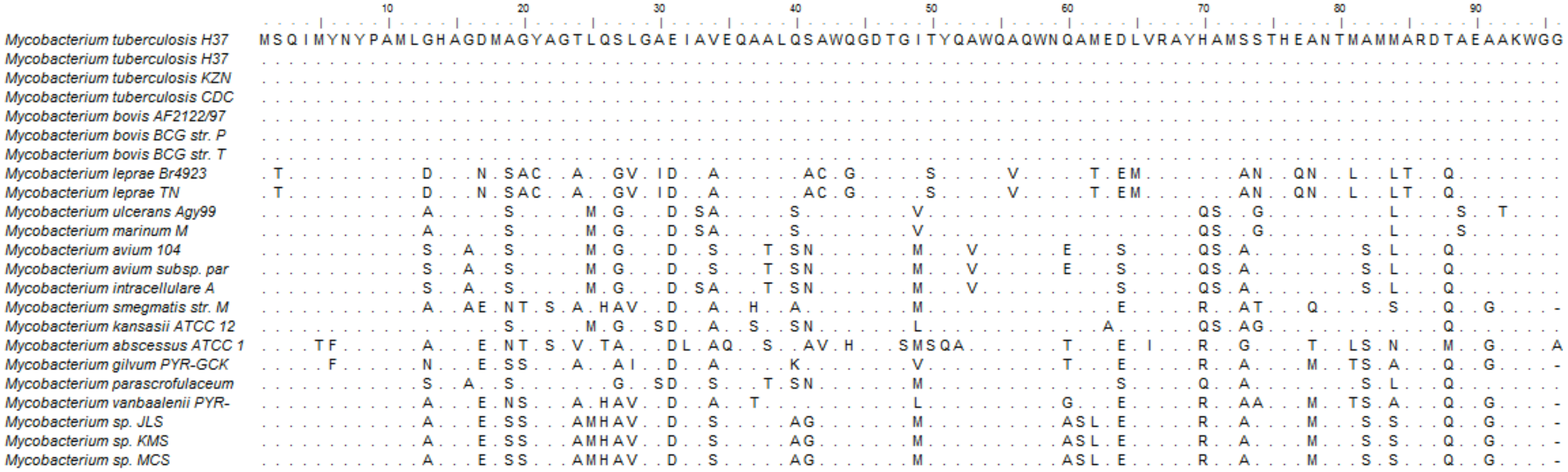

7/ Protein sequence alignment of *esxR* gene coding hypothetical PE family protein in *Mycobacterium* genome (locus tag Rv3019c in genome of *M. tuberculosis* H37Rv).

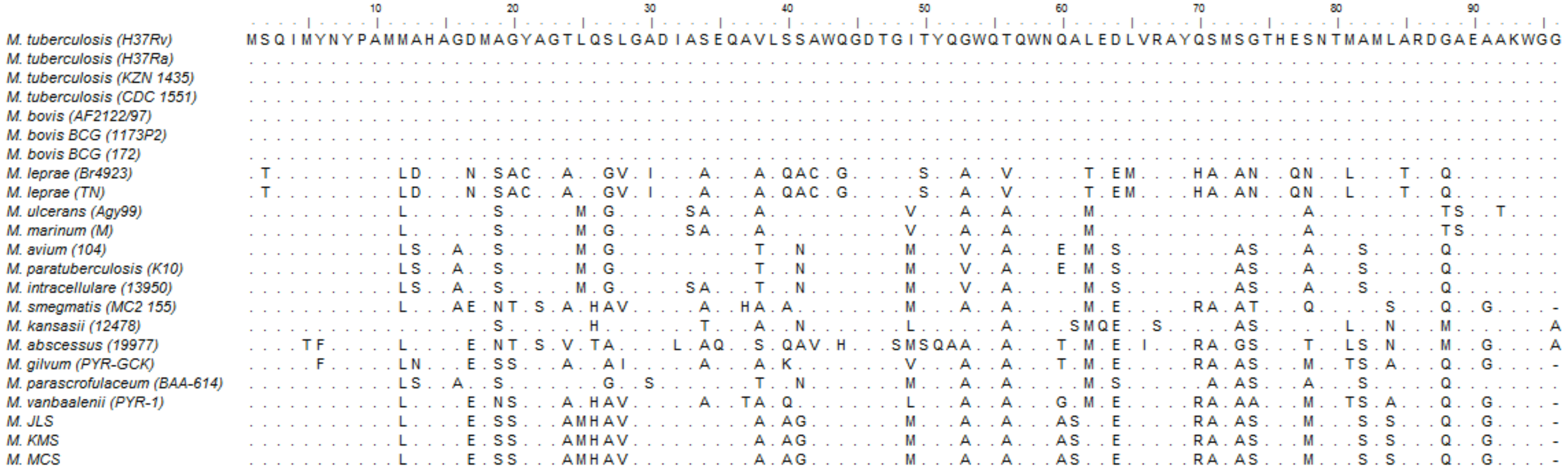

8/ Protein sequence alignment of *PE5* gene coding hypothetical PE family protein in *Mycobacterium* genome (locus tag Rv0285 in genome of *M. tuberculosis* H37Rv).

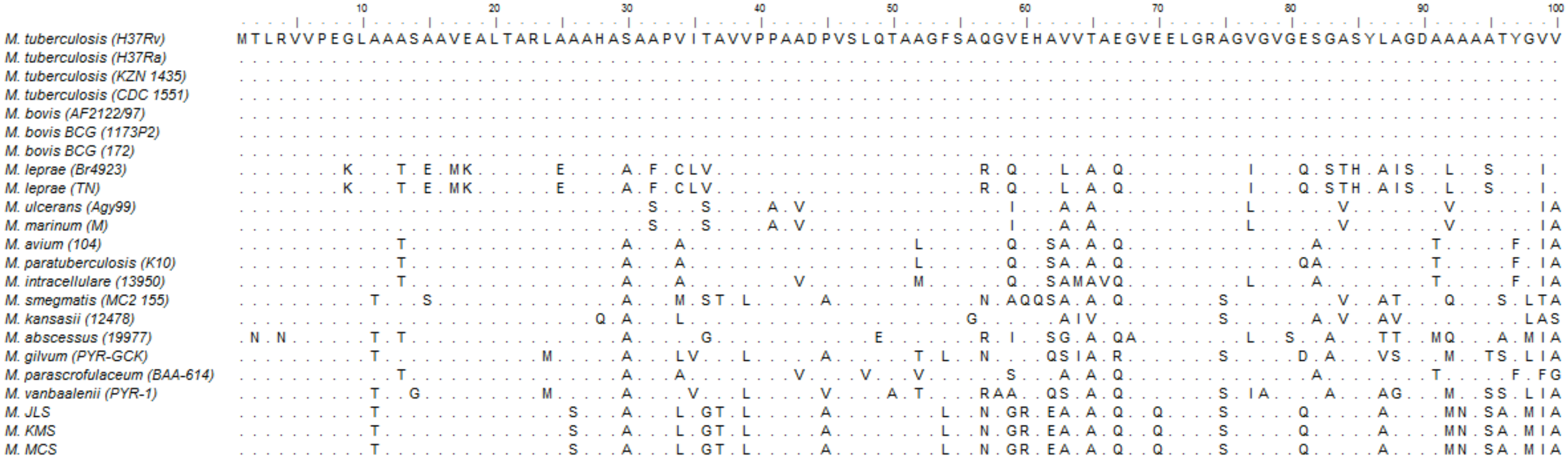

9/ Protein sequence alignment of *PPE48* gene coding hypothetical PPE family protein in *Mycobacterium* genome (locus tag Rv3022c in genome of *M. tuberculosis* H37Rv).

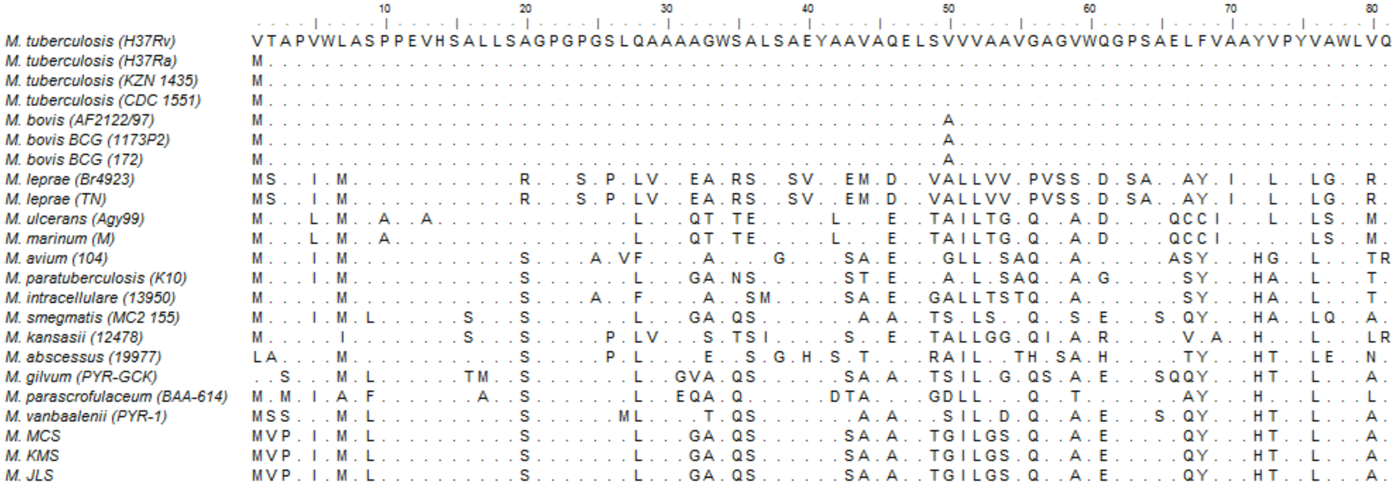

10.a/ Protein sequence alignment of *atpB* gene coding ATP synthase subunit A in *Mycobacterium* genome (locus tag Rv1304 in genome of *M. tuberculosis* H37Rv).

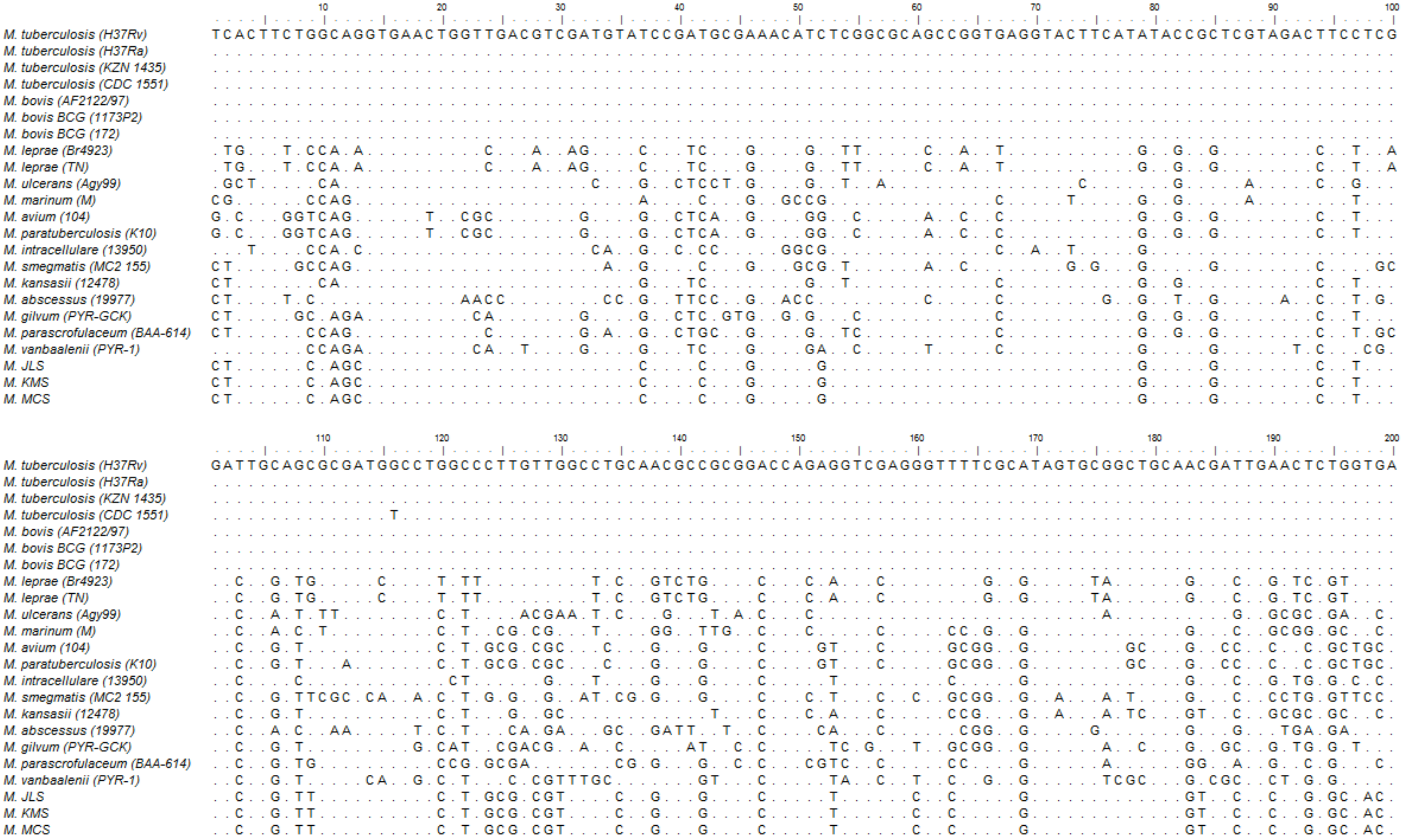

10.b/ Protein sequence alignment of *atpB* gene coding ATP synthase subunit A in *Mycobacterium* genome (locus tag Rv1304 in genome of *M. tuberculosis* H37Rv).

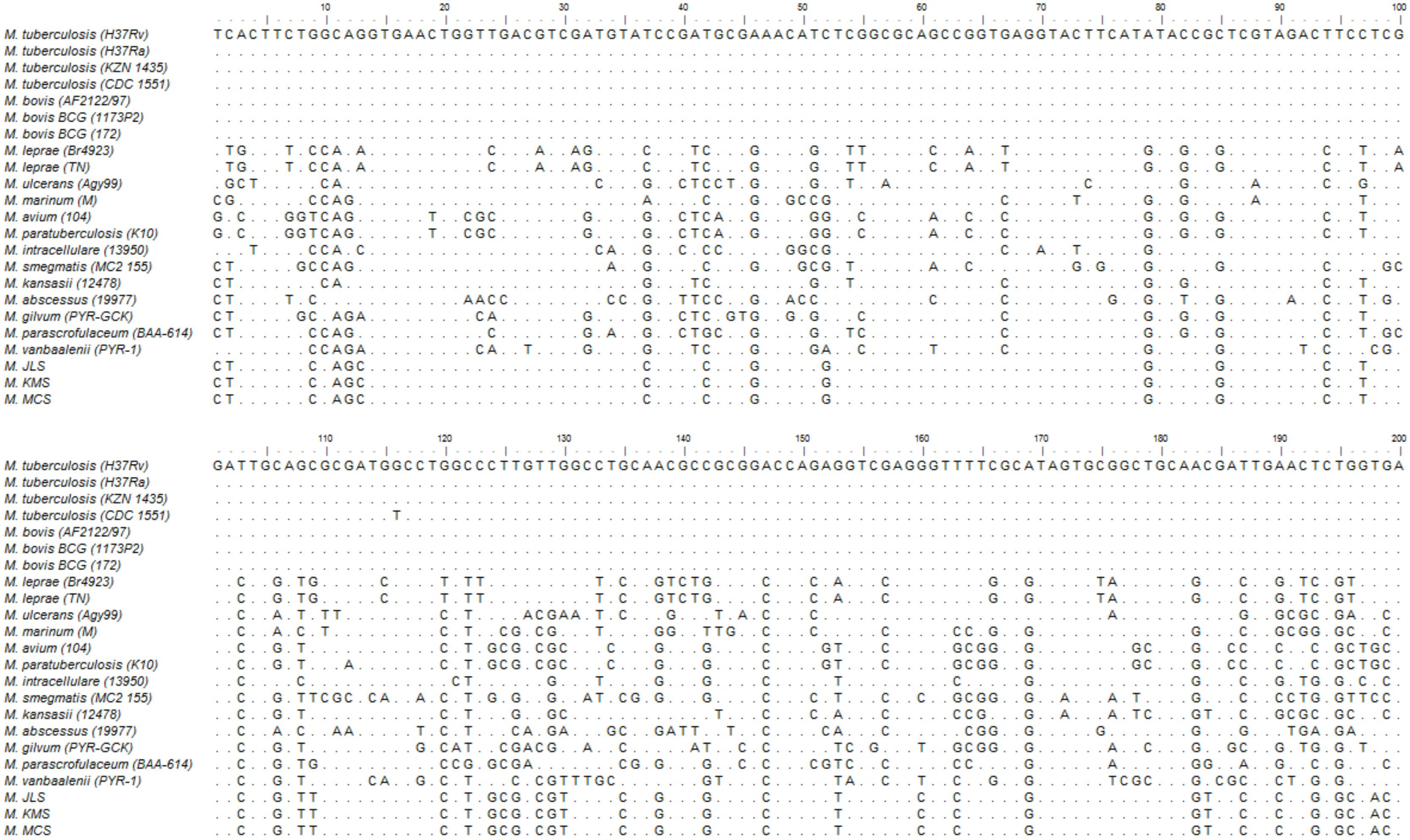

10.c/ Protein sequence alignment of *atpB* gene coding ATP synthase subunit A in *Mycobacterium* genome (locus tag Rv1304 in genome of *M. tuberculosis* H37Rv).

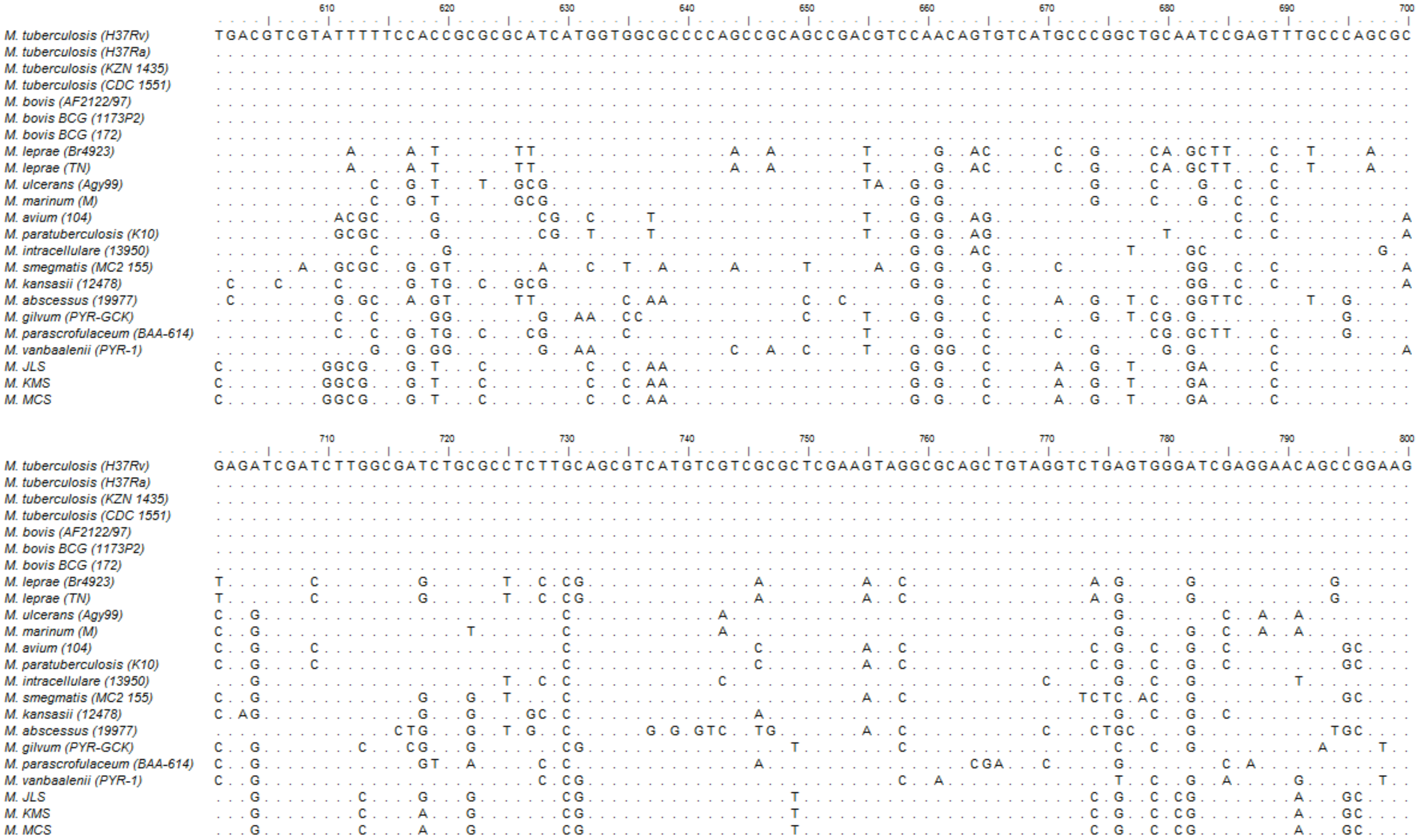

10.d/ Protein sequence alignment of *atpB* gene coding ATP synthase subunit A in *Mycobacterium* genome (locus tag Rv1304 in genome of *M. tuberculosis* H37Rv).

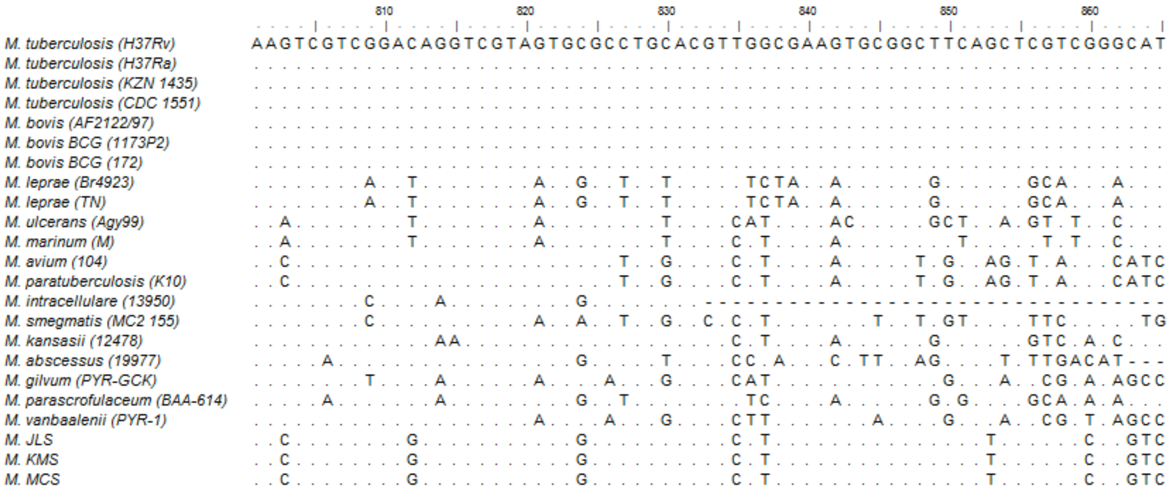

11.a/ Protein sequence alignment of *cmaA1* gene coding cyclopropane mycolic acid synthase in *Mycobacterium* genome (locus tag Rv3392c in genome of *M. tuberculosis* H37Rv).

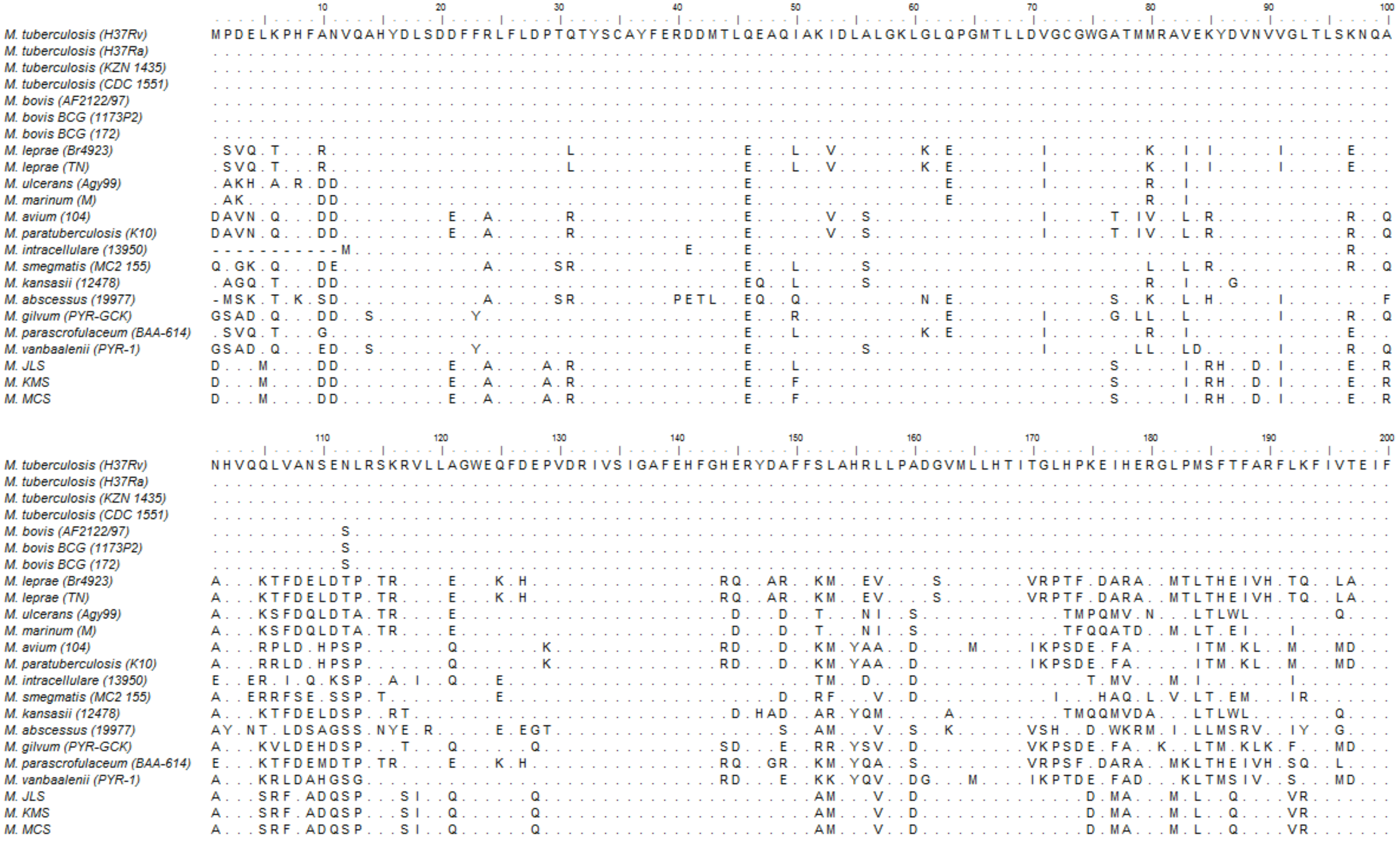

11.b/ Protein sequence alignment of *cmaA1* gene coding cyclopropane mycolic acid synthase in *Mycobacterium* genome (locus tag Rv3392c in genome of *M. tuberculosis* H37Rv).

```
                                              210       220       230       240       250       260       270       280
                                      ....|....|....|....|....|....|....|....|....|....|....|....|....|....|....|....|....|..
M. tuberculosis (H37Rv)               PGGRLPSIPMVQECASANGFTVTRVQSLQPHYAKTLDLWSAALQANKGQAIALQSEEVYERYMKYLTGCAEMFRIGYIDVNQFTCQK
M. tuberculosis (H37Ra)               .......................................................................................
M. tuberculosis (KZN 1435)            .......................................................................................
M. tuberculosis (CDC 1551)            .................................................D.....................................
M. bovis (AF2122/97)                  .......................................................................................
M. bovis BCG (1173P2)                 .......................................................................................
M. bovis BCG (172)                    .......................................................................................
M. leprae (Br4923)                    ...W..TV.T.E.H.T.A..KF..I....L..........A.R.E...NK...I......D..........KL..D..T..D...LE.
M. leprae (TN)                        ...W..TV.T.E.H.T.A..KF..I....L..........A.R.E...NK...I......D..........KL..D..T..D...LE.
M. ulcerans (Agy99)                   ...HP.T.E..E.QSVKP...L..RH...L..........AE..EVR.DE..KI..D.......R.....SKL.QE..V......L..
M. marinum (M)                        ........EK.ADHS.KA...L..R........R.....AQ....RRDE..EV..................DG..V.........LE.
M. avium (104)                        ...D..QAKG.I.H.QRA..G.Q...A.R....R...T.A...R.RRDE...I......D..........DL..E..T..A...LT.
M. paratuberculosis (K10)             ...D..QAKG.I.H.QRA..G.Q...A.R....R...T.A...R.RRDE...I......D..........DL..E..T..A...LT.
M. intracellulare (13950)             .............R.T......S.I............I.A.....H.E....V.................DA..V..V......LE.
M. smegmatis (MC2 155)                ......TVD..IDH.TRAS..LE.R....L...R...M.A...ESHHDD.VRI..Q...D...H.......NA..V.........LA.
M. kansasii (12478)                   ...HP.T.E..G.QSAKA...L..R...RL...R.....AE....HQDE...I.................KL..D.........L..
M. abscessus (19977)                  .......V...DKH.ALA..HI..HH.......R...C.ATN.A.HEDE..SV..Q...D..I........G..E.W...V....E.
M. gilvum (PYR-GCK)                   ...M..KVDD.EKH.TKAS.K.N.I.P.RL...R..QI.A.S.ESRRDD...I......D............L.TE..T..C...LA.
M. parascrofulaceum (BAA-614)         ...W..TPQT.G.Y.VTA..EL...H...L...R..ET.A...E..RAE...I..Q...D..........KL..Q..T..D...LE.
M. vanbaalenii (PYR-1)                ...D..KPTT.A.H.TKA..RL..E.R.RE.......I.AD..RKREDD.V.I..R.D.D..........DL..D..T.IC...LE.
M. JLS                                ...........E.R.A.G....S..............I.A...E.RRDE...I......D..........D...V.........LE.
M. KMS                                ...........E.R.A.G....S..............I.A...E.RRDE...I......D..........D...V.........LE.
M. MCS                                ...........E.R.A.G....S..............I.A...E.RRDE...I......D..........D...V.........LE.
```
